# Supplementary material for: Asymmetric total synthesis of polycyclic xanthenes and discovery of a WalK activator active against MRSA
Source: Nat Commun. 2024 Jul 13;15:5879. doi: 10.1038/s41467-024-49629-8 (PMC11245619; doi:10.1038/s41467-024-49629-8)
Supplement: Supplementary file 1 — Supplementary Information [file 41467_2024_49629_MOESM1_ESM.pdf]

Supplementary Information

Asymmetric Total Synthesis of Polycyclic Xanthenes and Discovery of a WalK Activator Active against MRSA

Min-Jing Cheng,[1,2,‡] Yan-Yi Wu,[1,2,‡] Hao Zeng,[3,‡] Tian-Hong Zhang,[1,2] Yan-Xia Hu,[1,2] Shi-Yi Liu,[4] Rui-Qin Cui,[4] Chun-Xia Hu,[4] Quan-Ming Zou,[3,*] Chuang-Chuang Li,[5,*] Wen-Cai Ye,[1,2,*] Wei Huang,[4,*] and Lei Wang[1,2*]

[1]State Key Laboratory of Bioactive Molecules and Druggability Assessment, Jinan University, Guangzhou 510632, P. R. China;

[2]Center for Bioactive Natural Molecules and Innovative Drugs, and Guangdong Province Key Laboratory of Pharmacodynamic Constituents of TCM and New Drugs Research, College of Pharmacy, Jinan University, Guangzhou 510632, P. R. China;

[3]National Engineering Research Center of Immunological Products, Department of Microbiology and Biochemical Pharmacy, College of Pharmacy, Army Medical University, Chongqing 400038, P. R. China;

[4]Department of Medical Laboratory, Shenzhen People's Hospital (The Second Clinical Medical College, Jinan University; The First Affiliated Hospital, Southern University of Science and Technology), Shenzhen 518020, Guangdong, P. R. China;

[5]Department of Chemistry, Shenzhen Grubbs Institute, Southern University of Science and Technology, Shenzhen 518055, P. R. China

*Correspondence: cpuwanglei@126.com

*Correspondence: whuang_sz@163.com

*Correspondence: chywc@aliyun.com

*Correspondence: ccli@sustech.edu.cn

*Correspondence: qmzou2007@163.com

‡These authors contributed equally.

Table of Contents

1. Supplementary Methods 2

2. Supplementary Discussion 4

2.1 Selected experimentation to asymmetric synthesis of isomyrtucommulone B (**14**) 4

2.2 Selected experimentation to synthesis of myrtucommulones D-E (**1** and **5**) 6

3. Supplementary Notes 14

3.1 Supplementary Table 1. Substrate scope of the Mitsunobu-mediated resolution 14

3.2 Synthesis of *rac*-**8-9**, *rac*-**11**, *rac*-**14**, *rac*-**17a-17o** 15

3.3 Synthesis of (+)- and (-)-**8-9**, (+)- and (-)-**11**, (+)- and (-)-**14**, (+)- and (-)-**17a-17o** 18

3.5 Synthesis of (+)- and (-)-**18**, (+)- and (-)-**18a-18b** 78

3.6 Synthesis of (+)- and (-)-**1-3** 81

3.7 Synthesis of (+)- and (-)-**5-7** 91

3.8 X-ray crystal structures of (+)-**1**, **1a**, (+)-**2**, (+)-**5**, *rac*-**5a**, (-)-**8**, (+)-**14**, (-)-**14**, **15**, (-)-**17n**, (-)-**17o** 99

3.9 Computational details 101

3.10 Synthetic ^{1}H, ^{13}C and 2D-NMR spectra 104

3.11 The tables and figures of antibacterial activity assay 196

4. Supplementary References 209

1. Supplementary Methods

Unless otherwise mentioned, all reactions were carried out under a nitrogen atmosphere under anhydrous conditions and all reagents were purchased from commercial suppliers without further purification. Solvent purification was conducted according to *Purification of Laboratory Chemicals* (Peerrin, D. D.; Armarego, W. L. and Perrins, D. R., Pergamon Press: Oxford, 1980). Yields refer to chromatographically and spectroscopically (^{1}H NMR) homogeneous materials, unless otherwise stated. Reactions were monitored by thin layer chromatography on plates (GF254) supplied by Yantai Chemicals (China) using UV light as visualizing agent, an ethanolic solution of phosphomolybdic acid, or basic aqueous potassium permanganate ($KMnO_4$), and heat as developing agents. If not specially mentioned, flash column chromatography uses silica gel (200-300 mesh) supplied by Tsingtao Haiyang Chemicals (China), preparative thin layer chromatography (PTLC) separations were carried out 0.50 mm Yantai (China) silica gel plates. NMR spectra was recorded on Bruker AV600, AV500, Bruker ARX400, and calibrated using residual undeuterated solvent as an internal reference ($CHCl_3$, δ 7.26 ^{1}H NMR, δ 77.20 ^{13}C NMR). The following abbreviations were used to explain the multiplicities: s = singlet, d = doublet, t = triplet, q = quartet, b = broad, m = multiplet. High-resolution mass spectra (HRMS) was recorded on a Bruker Apex IV FTMS mass spectrometer using ESI (electrospray ionization). Infrared spectra was recorded on a Shimadzu IR Prestige 21, using thin KBrs of the sample on KBr plates. Optical rotations were measured with a Rudolph autopol I automatic polarimeter using 10 cm glass cells with a sodium 589 nm filter.

2. Supplementary Discussion

2.1 Selected experimentation to asymmetric synthesis of isomyrtucommulone B (14)

Attempt 1:

We initially tried to treat phloroglucinol with **23**[1] and the following chiral phosphoric acids (**CPA1-9**) to afford (*R*)-**14a** via an asymmetric Friedel-Crafts-type Michael addition reaction (Supplementary Figure 1). However, the product was a *rac*-**14a**. Furthermore, we have also used other catalysts (Supplementary Figure 2). To our disappointment, the enantiomeric excess (*ee*) value of **14** was almost 0.

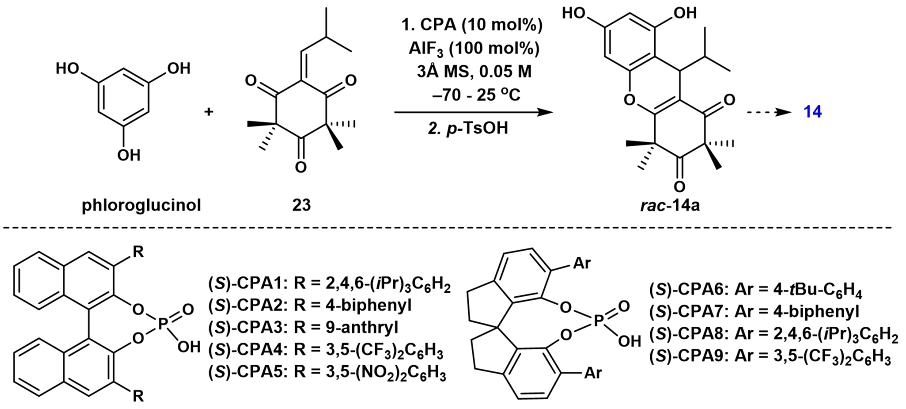

Supplementary Figure 1. **Attempts to asymmetric synthesis of 14.**

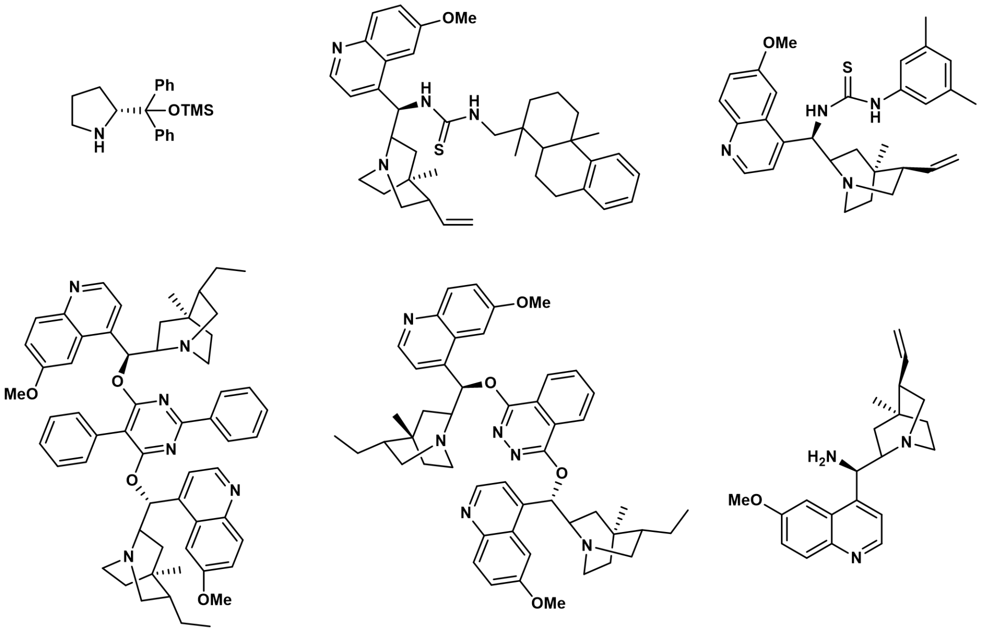

Supplementary Figure 2. The structures of six chiral reagents.

Attempt 2:

We tried to convert the **8**[1] to (*R*)**-14a** via a typical *retro*-Friedel-Crafts with different acids (Supplementary Figure 3a). After an extensive attempt, we failed to obtain the corresponding (*R*)**-14a** because of the racemization in this reaction.

Note: The reason of the racemization in this reaction may be attributed to the formation of intermediate II (Supplementary Figure 3c), followed by 1,3-hydrogen shift to afford III, resulting in the racemization. The detail reaction process was provided as follows.

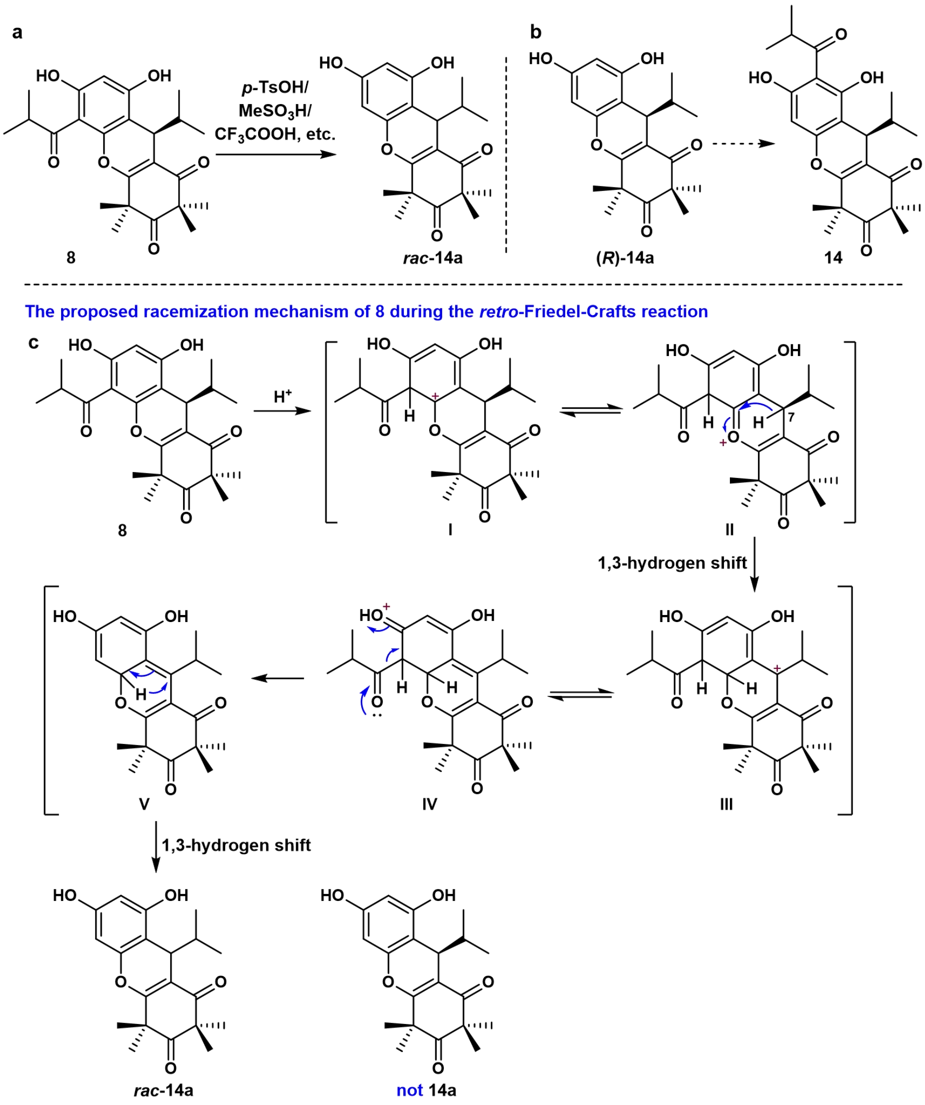

Supplementary Figure 3. Attempts to asymmetric synthesis of 14 and the reason for their failure. a, Synthesis of *rac*-14a. b, Imagining asymmetric synthesis of 14 from (*R*)-14a. c, The proposed mechanism for racemization of the *retro*-Friedel-Crafts reaction.

*All these results showed that compound **14** would be difficult to be synthesized.*

2.2 Selected experimentation to synthesis of myrtucommulones D-E (1 and 5)

Attempt 1:

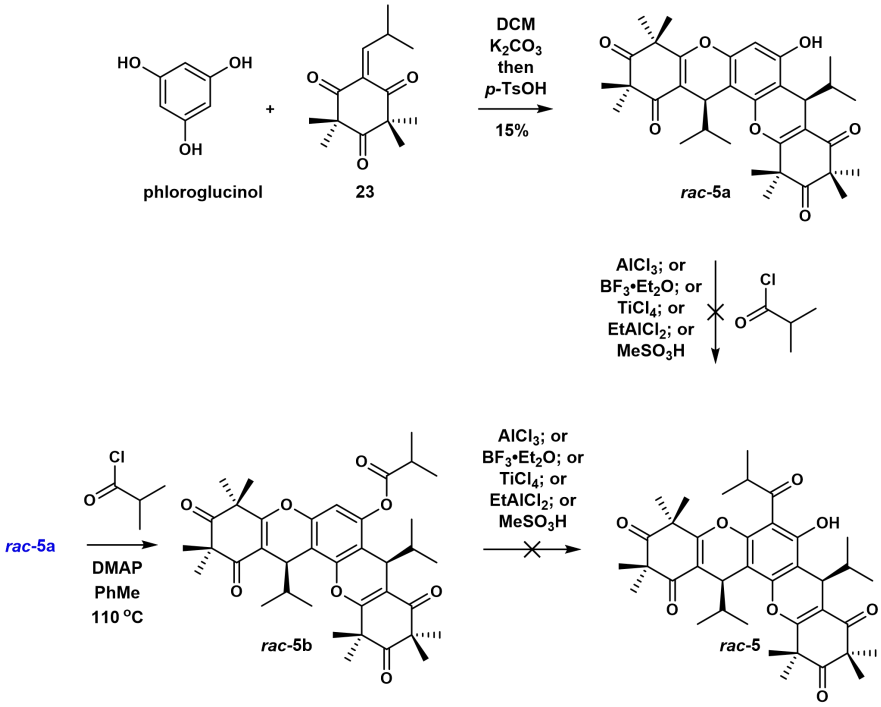

Supplementary Figure 4. Attempts to synthesis of *rac*-5.

We tried to treat *rac*-**5a** with Lewis acid to give *rac*-myrtucommulone E (**5**) via a Friedel-Crafts reaction (Supplementary Figure 4). Unfortunately, we recovered *rac*-**5a**, but not obtain *rac*-**5**. Subsequently, treatment of *rac*-**5a** with DMAP in PhMe provided *rac*-**5b**. Then, we tried to treat *rac*-**5b** with Lewis acid to afford *rac*-**5** via a Fries rearrangement (Supplementary Figure 4). However, only *rac*-**5a** was obtained since the conformation of *rac*-**5a** is probably more stable than that of *rac*-**5** under acid condition.

Note: The relative configuration of compound *rac*-**5a** was determined by X-ray diffraction analysis.

Compound *rac*-**5a**

R*f* = 0.6 (ethyl acetate/*n*-hexane = 1/4);

IR (KBr): 3305, 2966, 2878, 1717, 1650, 1549, 1460, 1323, 1186, 794, 760 cm^{-1}; ^{1}**H NMR** (400

MHz, $CDCl_3$) δ 6.50 (s, 1H), 4.33 (d, *J* = 3.5 Hz, 1H), 4.29 (d, *J* = 3.4 Hz, 1H), 1.96 (m, 2H), 1.64 (s, 3H), 1.59 (s, 3H), 1.50 (s, 3H), 1.44 (s, 3H), 1.42 (s, 3H), 1.36 (s, 6H), 1.25 (s, 3H), 0.92 (d, *J* = 6.9 Hz, 3H), 0.85 (d, *J* = 6.9 Hz, 3H), 0.79 (d, *J* = 6.8 Hz, 3H), 0.77 (d, *J* = 6.8 Hz, 3H); **^{13}C NMR** (101 MHz, $CDCl_3$) δ 212.5, 212.2, 198.5, 197.8, 169.3, 168.6, 152.6, 151.1, 149.9, 111.5, 111.3, 108.8, 104.8, 99.9, 56.2 (2 carbons overlapped), 47.8, 47.6, 35.6, 35.0, 32.8, 32.6, 25.7, 25.3 (2 carbons overlapped), 25.1, 24.9, 24.6, 24.6, 24.1, 19.9, 19.2, 19.0, 18.6; **HRMS** (ESI-TOF) m/z: $[M-H]^-$ calcd. for $C_{34}H_{41}O_7$ 561.2852; found, 561.2853.

Attempt 2:

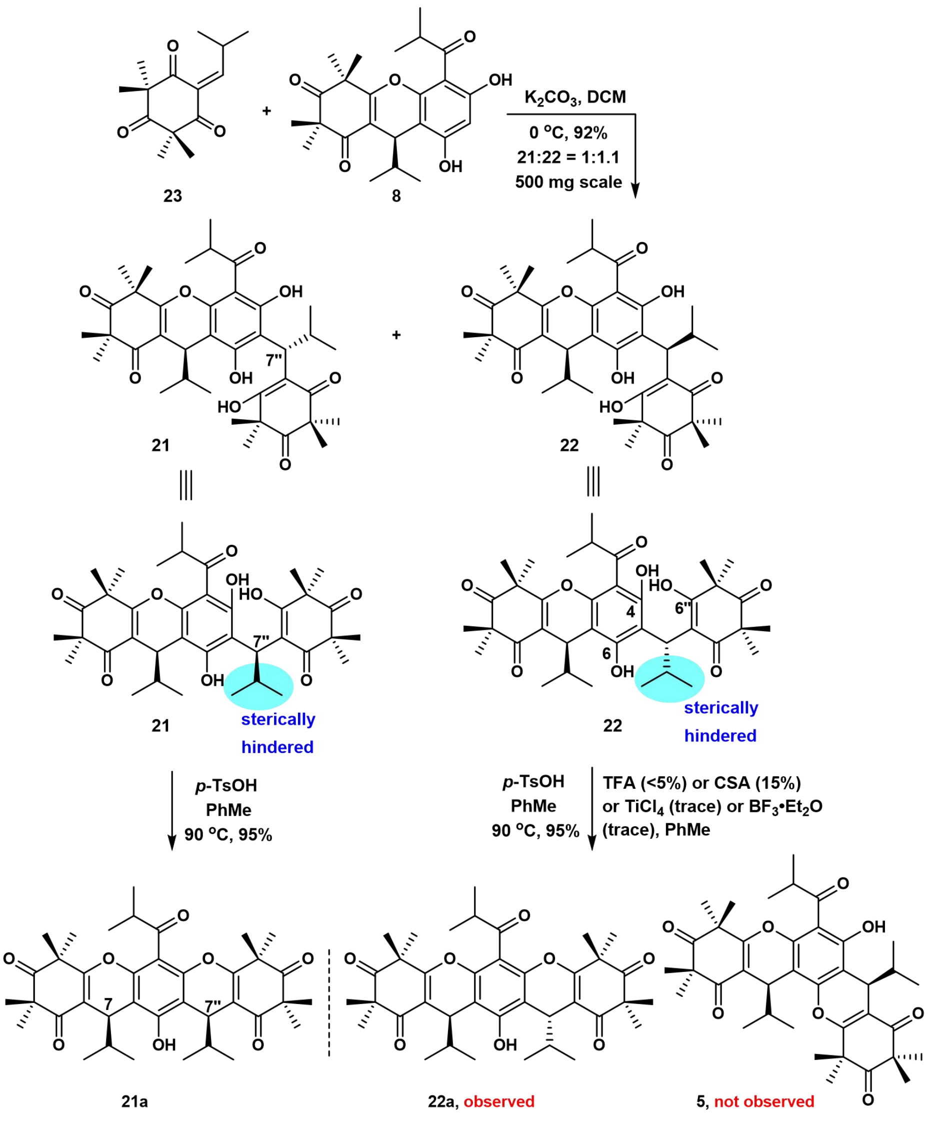

Supplementary Figure 5. Attempts to asymmetric synthesis of 5.

We tried to treat **22**, which could be provided from compounds **10** (500 mg, 1.0 equiv.) and **20**[1]

(1.5 equiv.) by Michael addition reaction, with different acids to give myrtucommulone E (**5**) via an intramolecular cyclization (Supplementary Figure 5). To our disappointment, we only obtained **22a**, but not **5**. The reason might be that compound **22** was existed in the form of linear one under acid conditions, and then the intramolecular exclusive attack of the less hindered free phenolic hydroxyl group (C4-OH) on the less hindered C6″ could give the linear product.

Note: 1. Since both ^{1}H and ^{13}C NMR spectra of product **21** and **22** showed doubled signal patterns, which was probably because of the presence of keto-enol tautomers, further cyclization of **21** and **22** was carried out to afford specify **21a** and **22a**, respectively.

2. The C7 and C7″ absolute configurations of **21** and **22** were determined to be (*R*, *R*) and (*R, S*) based on the reference[2], respectively.

3. To test the antibacterial activity of **22** *in vivo* (see Supplementary Figure 254), synthesis of 378.3 mg of **22** was achieved.

Compound **21**

R*f* = 0.45 (ethyl acetate/*n*-hexane = 1/3); $[\alpha]_D^{29}$ = +120.1 (*c* = 0.1 in MeOH); **IR (KBr)**: 3118, 2973, 2937, 2871, 1715, 1661, 1599, 1573, 1467, 1381, 1183 cm^{-1}; **^{1}H NMR** (500 MHz, $CDCl_3$) δ 11.91 (s, 1H), 4.43 (d, *J* = 3.4 Hz, 1H), 3.97 (m, 1H), 3.82 (d, *J* = 10.8 Hz, 1H), 3.07 (m, 1H), 1.90 (m, 1H), 1.58 (s, 3H), 1.47 (s, 3H), 1.41 (s, 3H), 1.41 (s, 3H), 1.38 (s, 3H), 1.33 (s, 3H), 1.31 (s, 3H), 1.30 (s, 3H), 1.27 (d, *J* = 7.2 Hz, 3H), 1.25 (d, *J* = 6.6 Hz, 3H), 0.85 (d, *J* = 6.3 Hz, 3H), 0.80 (d, *J* = 6.9 Hz, 3H), 0.76 (d, *J* = 4.0 Hz, 3H), 0.75 (d, *J* = 4.5 Hz, 3H) (Note: keto-enol tautomers ratio: 2.5:1; ^{1}H NMR date of the main tautomer was listed); **^{13}C NMR** (126 MHz, $CDCl_3$) δ 212.4, 212.3, 210.1, 203.3, 197.7, 178.4, 166.9, 161.8, 161.5, 152.4, 113.9, 112.7, 112.6, 106.4, 102.1, 56.3, 55.2, 49.0, 47.4, 39.5, 39.5, 35.1, 31.8, 27.0, 26.1, 25.7, 25.5, 25.2, 25.1, 25.0, 24.1, 23.4, 22.1, 21.9, 21.3, 19.2, 19.0, 18.1 (Note: ^{13}C NMR date of the main tautomer was listed); **HRMS** (ESI-TOF) m/z: $[M+H]^+$ calcd. for $C_{38}H_{51}O_9$ 651.3528; found, 651.3526.

Compound **22**

R*f* = 0.40 (ethyl acetate/*n*-hexane = 1/3); $[\alpha]_D^{29}$ = +62.3 (*c* = 0.1 in MeOH); **^{1}H NMR** (500 MHz, $CDCl_3$) δ 11.97 (s, 1H), 10.99 (s, 0.55H), 10.93 (s, 0.45H), 10.24 (s, 1H), 4.42 (m, 1H), 3.97 (m, 1H), 3.86 (d, *J* = 11.3 Hz, 0.45 H), 3.76 (d, *J* = 10.8 Hz, 0.55H), 3.09 (m, 0.45H), 3.01 (m, 0.55H),

1.90 (m, 1H), 1.59 (s, 1.6H), 1.58 (s, 1.4H), 1.49 (s, 1.6H), 1.49 (s, 1.4H), 1.42 (s, 4.4H), 1.41 (s, 1.6H), 1.38 (t, *J* = 2.8 Hz, 6H), 1.32 (m, 6H), 1.26 (m, 6H), 0.90 (d, *J* = 6.4 Hz, 1.5H), 0.83 (d, *J* = 6.3 Hz, 1.9H), 0.80 (d, *J* = 4.7 Hz, 1.3H), 0.78 (d, *J* = 4.1 Hz, 1.3H), 0.72 (m, 6H) (Note: keto-enol tautomers ratio: 1.2:1); **^{13}C NMR** (126 MHz, $CDCl_3$) δ 212.7, 212.5, 212.3, 212.2, 210.6, 209.9, 204.0, 203.3, 197.8, 197.7, 178.6, 176.9, 167.2, 166.8, 161.8, 162.1, 162.1, 161.5, 152.6, 152.2, 114.1, 113.9, 112.8, 112.8, 112.6, 112.4, 106.8, 106.3, 102.3, 102.1, 56.4, 56.3, 55.1, 54.6, 49.2, 49.0, 47.4, 47.3, 41.5, 39.9, 39.6, 39.4, 35.1, 34.6, 32.1, 31.7, 27.0, 26.5, 26.3, 26.2, 25.9, 25.8, 25.5, 25.4, 25.4, 25.4, 25.4, 25.3, 25.1, 24.9, 24.2, 24.1, 24.0, 24.0, 22.2, 22.2, 22.1, 22.1, 21.3, 21.2, 19.8, 19.1, 19.0, 18.5, 18.2, 18.0; **IR (KBr)**: 3117, 2974, 2936, 2872, 1718, 1658, 1610, 1572, 1468, 1384, 1298, 1016 cm^{-1}; **HRMS** (ESI-TOF) m/z: $[M+H]^+$ calcd. for $C_{38}H_{51}O_9$ 651.3528; found, 651.3528.

Compound **21a**

R*f* = 0.25 (ethyl acetate/*n*-hexane = 1/8); $[\alpha]_D^{26}$ = 0 (*c* = 0.1 in MeOH); **IR (KBr)**: 3422, 2972, 2876, 1715, 1669, 1470, 1437, 1319, 1174, 984 cm^{-1}; **^{1}H NMR** (500 MHz, $CDCl_3$) δ 7.86 (s, 1H), 4.60 (d, *J* = 3.5 Hz, 2H), 3.19 (m, 1H), 1.97 (m, 2H), 1.52 (s, 6H), 1.44 (s, 6H), 1.38 (s, 6H), 1.36 (s, 6H), 1.29 (d, *J* = 6.9 Hz, 6H), 0.86 (d, *J* = 6.8 Hz, 6H), 0.77 (d, *J* = 6.8 Hz, 6H); **^{13}C NMR** (126 MHz, $CDCl_3$) δ 211.8 (2 carbons overlapped), 205.4, 198.6 (2 carbons overlapped), 169.1 (2 carbons overlapped), 152.4, 147.5 (2 carbons overlapped), 111.2 (2 carbons overlapped), 110.4, 108.5 (2 carbons overlapped), 56.0 (2 carbons overlapped), 47.5 (2 carbons overlapped), 43.3, 35.1 (2 carbons overlapped), 32.4 (2 carbons overlapped), 24.9 (2 carbons overlapped), 24.8 (2 carbons overlapped), 24.7 (2 carbons overlapped), 24.2 (2 carbons overlapped), 19.1 (2 carbons overlapped), 18.8 (2 carbons overlapped), 17.7 (2 carbons overlapped); **HRMS** (ESI-TOF) m/z: $[M+H]^+$ calcd. for $C_{38}H_{49}O_8$ 633.3422; found, 633.3423.

Compound **22a**

R*f* = 0.2 (ethyl acetate/*n*-hexane = 1/8); $[\alpha]_D^{26}$ = +81.6 (*c* = 0.1 in MeOH); **IR (KBr)**: 3410, 2972, 2876, 1714, 1667, 1437, 1389, 1321, 1175, 984 cm^{-1}; **^{1}H NMR** (500 MHz, $CDCl_3$) δ 7.60 (s, 1H), 4.57 (d, *J* = 3.4 Hz, 2H), 3.18 (m, 1H), 1.95 (m, 2H), 1.55 (s, 6H), 1.45 (s, 6H), 1.43 (s, 6H), 1.42 (s, 6H), 1.25 (d, *J* = 7.1 Hz, 3H), 1.24 (d, *J* = 7.0 Hz, 3H), 0.80 (d, *J* = 7.3 Hz, 6H), 0.77 (d, *J* =

7.3 Hz, 6H); **^{13}C NMR** (126 MHz, $CDCl_3$) δ 211.8 (2 carbons overlapped), 204.4, 198.7 (2 carbons overlapped), 168.8 (2 carbons overlapped), 152.0, 147.5 (2 carbons overlapped), 110.8 (2 carbons overlapped), 110.2, 108.6 (2 carbons overlapped), 55.8 (2 carbons overlapped), 47.5 (2 carbons overlapped), 42.7, 35.7 (2 carbons overlapped), 32.2 (2 carbons overlapped), 25.2 (2 carbons overlapped), 24.8 (2 carbons overlapped), 24.8 (2 carbons overlapped), 24.4 (2 carbons overlapped), 19.2 (2 carbons overlapped), 18.6 (2 carbons overlapped), 18.0 (2 carbons overlapped); **HRMS** (ESI-TOF) m/z: $[M+H]^+$ calcd. for $C_{38}H_{49}O_8$ 633.3422; found, 633.3419.

Attempt 3:

We tried to treat *rac*-**14** with different bases or acids to give ***rac*-1b** via a Michael addition reaction (Supplementary Figure 6). However, only *rac*-**14** was recovered probably because of the steric effect from *rac*-**14** or a very weak nucleophile for *rac*-**14**.

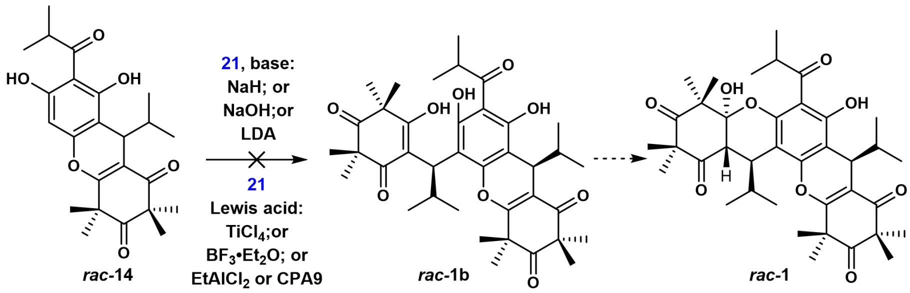

Supplementary Figure 6. Attempts to synthesis of *rac*-1.

All these results showed that compounds myrtucommulones D-E would be difficult to be synthesized.

Note: We have also compared the differences between the this work and the previous work in 'Cheng, M. J. et al. Catalytic asymmetric total syntheses of myrtucommuacetalone, myrtucommuacetalone B, and callistrilones A, C, D and E. *Chem. Sci.* **9**, 1488-1495 (2018)'. Additionally, we have discussed the reasons why the previous work was unable to achieve the synthesis of myrtucommulone D (**1**) and its analogues. The details are described as follows.

1. The differences between this work and the previous work.

(1) In this work, asymmetric synthesis of 2- and 4-substituted xanthenes (Supplementary Figure 7a) can be readily realized within 12 h from the *rac*-xanthenes and inexpensive chiral alcohol

(~40$/50 g) by a Mitsunobu-mediated chiral resolution. However, in our previous work, the asymmetric Friedel-Crafts-type Michael addition could only give 2-substituted xanthenes, and need an expensive chiral phosphoric acid [(S)-CPA9; ~400$/100 mg] with long time (up to 7 days) (Supplementary Figure 7b).

(2) In this work, the chiral products (4- and 2-substituted xanthenes) were not only translated into the natural products **1** and its analogues (Supplementary Figure 7a) but also served as precursors for the asymmetric synthesis of myrtucommuacetalone B (Supplementary Figure 7b) and other related natural products. However, the previous work could hardly achieve the asymmetric synthesis of **1** and its analogues.

(3) In this work, the three contiguous stereocenters in **1** were constructed using a novel *retro*-hemiketalization-double Michael addition cascade reaction (Supplementary Figure 7a, *dr* > 20:1), which provided a single product **1** in 81% yield. The diastereoselective reaction mechanism was elucidated through experiments and Quantum mechanical calculations. However, constructing the consecutive three stereocenters in myrtucommuacetalone B, as referenced in 'Cheng, M. J. et al. Catalytic asymmetric total syntheses of myrtucommuacetalone, myrtucommuacetalone B, and callistrilones A, C, D and E. *Chem. Sci.* **9**, 1488-1495 (2018)', using a Michael-ketalization-annulation cascade reaction resulted in poorer diastereoselective (*dr* 11:1) compared to the result in this work. Meanwhile, the reaction mechanism has not yet been explained through experiments or Quantum mechanical calculations (Supplementary Figure 7b).

2. The possible reasons for the reaction in the previous work not working.

(1) Although the 2-substituted xanthene **8** obtained from the previous work (Supplementary Figure 7b) can undergo a Michael addition reaction to yield **22**, the transformation of **22** to **5** presents a challenge (Supplementary Figures 5 and 8a). This is due to the likelihood of **22** existing in a linear form under acidic conditions, leading to the intramolecular attack of the less hindered free phenolic hydroxyl group (C4-OH) on the less hindered C6″ and resulting in the formation of the linear product **22a**.

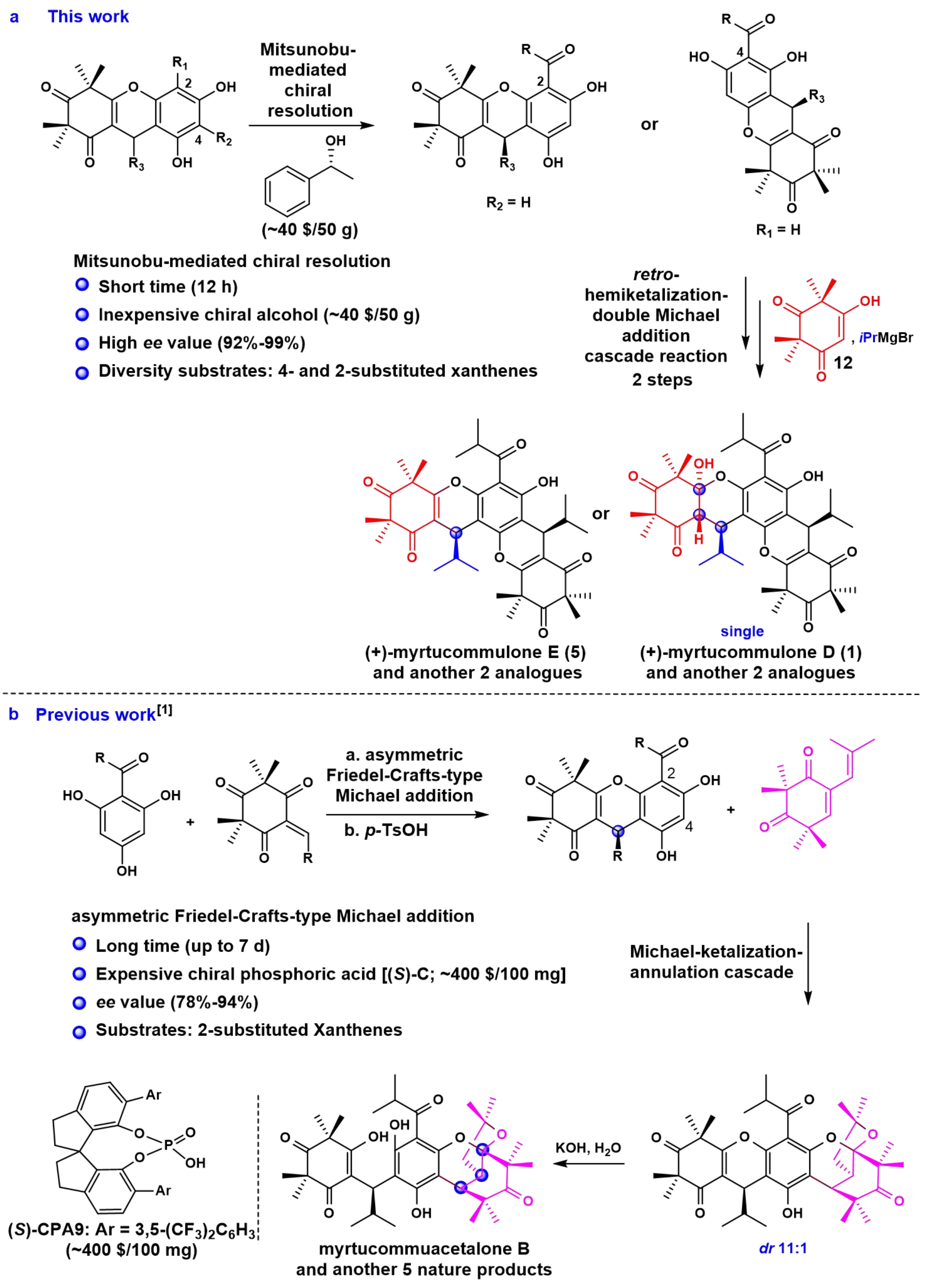

Supplementary Figure 7. Comparison between the this work and previous work. a, This work. b, Previous work. (**Note:** Supplementary Figure 7b is reproduced from Ref. 1 with permission from the Royal Society of Chemistry).

(2) An optically pure 2-substituted xanthene, exemplified by compound **9** (Supplementary Figure 8b) and obtainable according to the procedures outlined in the previous work[1], proved

challenging to convert into optically pure compound **11** through *retro*-Friedel-Crafts, followed by Friedel-Crafts reactions [Deng, L. M. et al. Discovery and biomimetic synthesis of a polycyclic polymethylated phloroglucinol collection from *Rhodomyrtus tomentosa*. *J. Org. Chem.* **87**, 4788-4800 (2022).]. This hindrance was attributed to the issue of racemization (Supplementary Figure 8b and Supplementary Figure 3a), making it difficult to achieve the asymmetric synthesis of compound **1** and its analogues. Additionally, the proposed racemization mechanism has been discussed in Scheme S3c.

(3) The novel *retro*-hemiketalization-double Michael addition cascade reaction, crucial for the synthesis of compound **1** and its analogues, is absent in the previous work[1]. Consequently, the absence of this key reaction in this reference may be a contributing factor to the challenge of achieving compound **1** and its analogues.

a

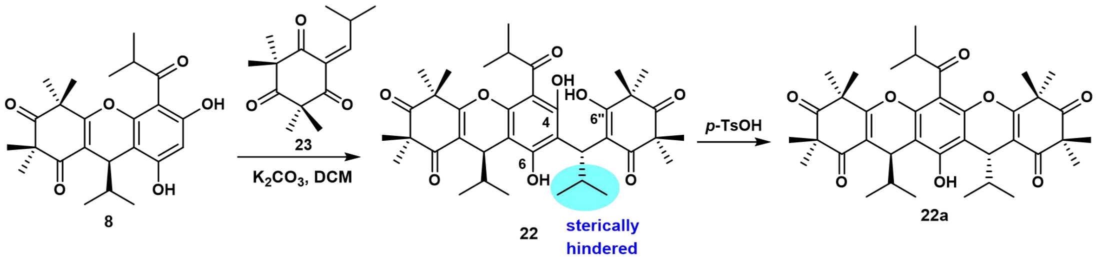

b Deng, L. M., Tang, W., Wang, S. Q., Song, J. G., Huang, X. J., Zhu, H. Y., Li, Y. L., Ye, W. C., Hu, L. J., Wang, Y. (2022), Discovery and Biomimetic Synthesis of a Polycyclic Polymethylated Phloroglucinol Collection from *Rhodomyrtus tomentosa*† The Journal of Organic Chemistry, 87: 4788-4800. https://doi.org/10.1021/acs.joc.2c00071

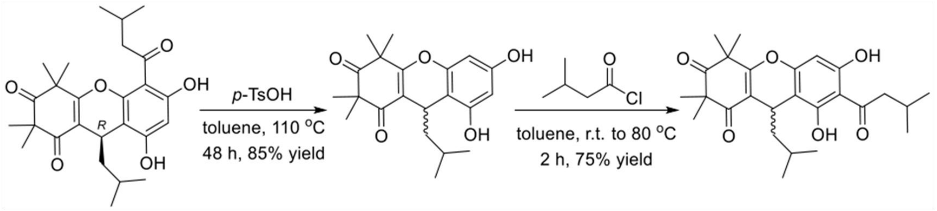

rhodomyrtone B (9) ***rac*-10** ***rac*-rhodomyrtone (11)**

Supplementary Figure 8. The possible reasons for the reaction in previous work not succeeding in the asymmetric synthesis of myrtucommulones D-E. a, Our attempts to synthesis of myrtucommulone E. b, Reported attempts to asymmetric synthesis of rhodomyrtone. [**Note:** Supplementary Figure 8b is reprinted (adapted) with permission from {Deng, L. M. et al. Discovery and biomimetic synthesis of a polycyclic polymethylated phloroglucinol collection from *Rhodomyrtus tomentosa*. *J. Org. Chem.* **87**, 4788-4800 (2022).}. Copyright {2022} American Chemical Society.]

3. Supplementary Notes

3.1 Supplementary Table 1. Substrate scope of the Mitsunobu-mediated resolution[a,b]

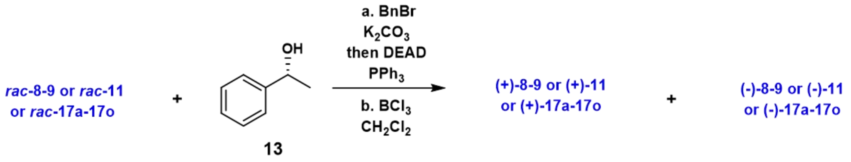

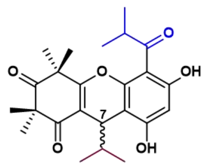

(7*R*)-(+)-8, 33%, 97% ee
(7*S*)-(-)-8, 37%, 96% ee, X-ray

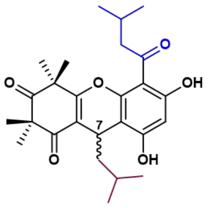

(7*R*)-(+)-9, 30%, 98.5% ee
(7*S*)-(-)-9, 35.5%, 99% ee

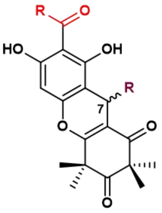

R = *i*Bu, (7*R*)-(+)-11, 38%, 97.5% ee
(7*S*)-(-)-11, 35.5%, 95.5% ee;
R = *i*Pr, (7*R*)-(+)-14, 40%, 99% ee, X-ray
(7*S*)-(-)-14, 36.5%, 99% ee, X-ray

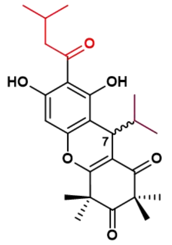

(7*R*)-(+)-17a, 37%, 96.5% ee
(7*S*)-(-)-17a, 34.5%, 92% ee

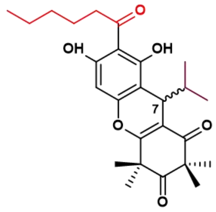

(7*R*)-(+)-17b, 36%, 96% ee
(7*S*)-(-)-17b, 33.5%, 93.5% ee

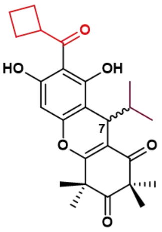

(7*R*)-(+)-17c, 37%, 99% ee
(7*S*)-(-)-17c, 34%, 95% ee

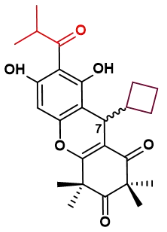

(7*R*)-(+)-17d, 35%, 97% ee
(7*S*)-(-)-17d, 32%, 98% ee

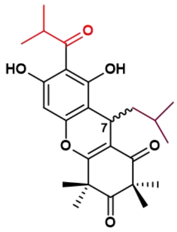

(7*R*)-(+)-17e, 36%, 99% ee
(7*S*)-(-)-17e, 32%, 95% ee

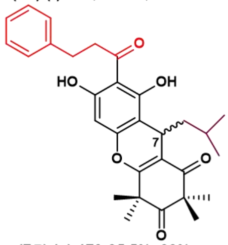

(7*R*)-(+)-17f, 35.5%, 99% ee
(7*S*)-(-)-17f, 32.5%, 95.5% ee

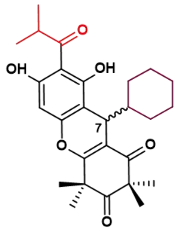

(7*R*)-(+)-17g, 37%, 96% ee
(7*S*)-(-)-17g, 33.5, 98% ee

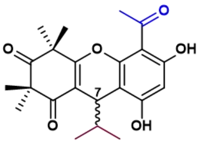

(7*R*)-(+)-17h, 32%, 99% ee
(7*S*)-(-)-17h, 36%, 99% ee

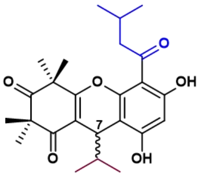

(7*R*)-(+)-17i, 31.5%, 98.5% ee
(7*S*)-(-)-17i, 34.5%, 99% ee

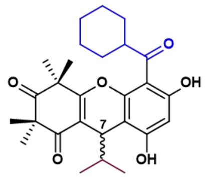

(7*R*)-(+)-17j, 33%, 96% ee
(7*S*)-(-)-17j, 38%, 99% ee

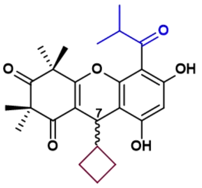

(7*R*)-(+)-17k, 31%, 98.5% ee
(7*S*)-(-)-17k, 35.5%, 99% ee

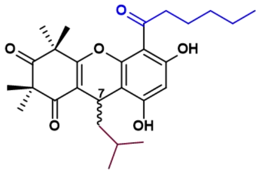

(7*R*)-(+)-17l, 32.5%, 96% ee
(7*S*)-(-)-17l, 36%, 99% ee

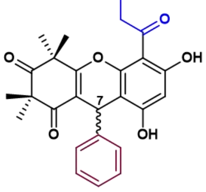

(7*R*)-(+)-17m, 32.5%, 99% ee
(7*S*)-(-)-17m, 38%, 99% ee

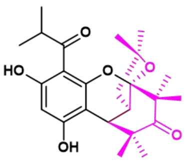

(+)-17n, 37%, 98.5% ee, X-ray

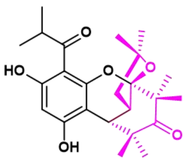

(-)-17n, 33.5%, 99% ee

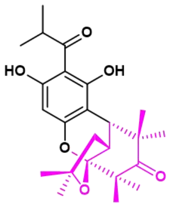

(+)-17o, 31%, 99% ee

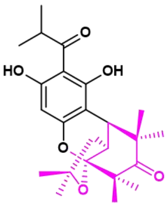

(-)-17o, 34%, 97% ee, X-ray

Reaction conditions:[a]*rac*-**8-9** (1.0 equiv.), or *rac*-**11** (1.0 equiv.), or *rac*-**17a-17o** (1.0 equiv.), K_2CO_3 (1.5 equiv.), BnBr (1.05 equiv.), 60 °C, acetone (0.05 M), 10 h, then filtered and

concentrated, then DEAD (1.5 equiv.), PPh_3 (1.5 equiv.), **13** (1.2 equiv.), 0-25 °C,THF/PhMe (V/V=1:1, 0.1 M), 1 h. [b]BCl_3 (10.0 equiv.), CH_2Cl_2 (0.1 M), −50 °C, 1 h. [c]Isolated yields for 2 steps.[d]The *ee* values were determined by chiral HPLC analysis.

Note: (1) The detailed structures of (+)-**8-9**, (+)-**11**, (+)-**14**, (+)-**17a-17o** and (-)-**8-9**, (-)-**11**, (-)-**14**, (-)-**17a-17o** were as follows (see Supplementary Pages 22-77).

(2) The synthesis of 2.5 g of (+)-**8** and (-)-**8**, 1.3 g of (+)-**11** and (-)-**11**, 13 g of (+)- and (-)-**14** as well as 2.6 g of (+)-**17a** and (-)-**17a** could be achieved (see Supplementary Pages 23, 28, 32, 35).

3.2 Synthesis of *rac*-8-9, *rac*-11, *rac*-14, *rac*-17a-17o

Note: we used three approaches to provide *rac*-**8-9**, *rac*-**11**, *rac*-**14**, and *rac*-**17a-17o**, respectively. The detailed procedures were as follows.

3.2.1 Procedure A: General procedure for synthesis of *rac*-11, *rac*-14, *rac*-17a-17g

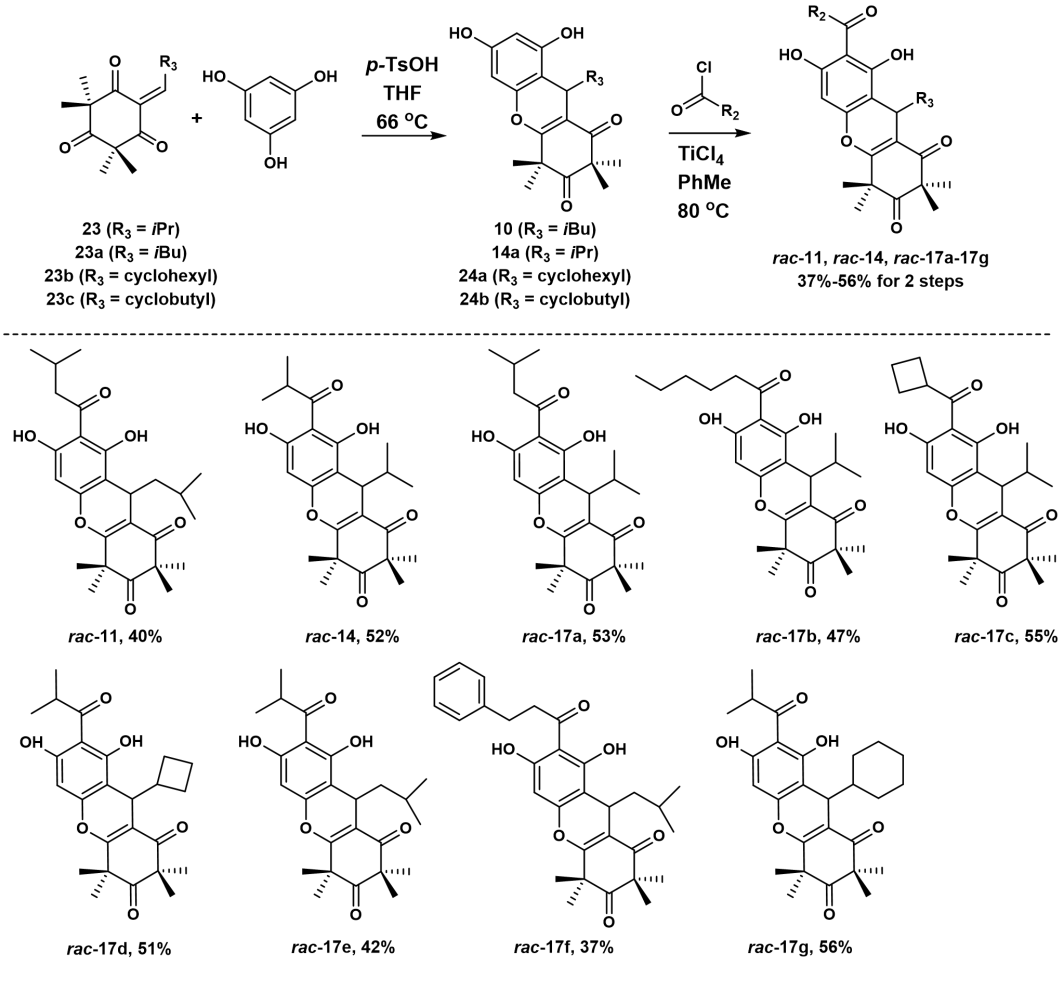

Supplementary Figure 9. Synthesis of *rac*-11, *rac*-14, *rac*-17a-17g.

A suspension of **23** or **23a-23c** (1.1 equiv.) with phloroglucinol (1.0 equiv.) in tetrahydrofuran (THF; 0.1 M) was stirred for 10 h at 66 °C. To the resulting mixture was added *p*-toluenesulfonic acid (*p*-TsOH; 3.0 equiv.) and stirred for 5 h at 66 °C. The mixture was quenched with saturated aqueous sodium bicarbonate, and extracted with diethyl ether. The combined organic extracts were washed with brine, dried over Na_2SO_4, filtered and concentrated in *vacuo*. The residue was purified by flash-column chromatography on silica gel (10%-50% ethyl acetate/*n*-hexane) to afford the corresponding products **10** or **14a** or **24a-24b**.

To a solution of **10** (1.0 equiv.) or **14a** (1.0 equiv.) or **24a-24b** (1.0 equiv.) and titanium tetrachloride ($TiCl_4$; 4.0 equiv.) in methylbenzene (PhMe) was added acyl chloride (1.05 equiv.) at 0 ºC. The resulting mixture was allowed to warm up to 80 °C. After the reaction was finished according to TLC, the mixture was quenched with saturated aqueous ammonium chloride, and extracted with ethyl acetate. The combined organic layers were dried over Na_2SO_4, filtered and concentrated in *vacuo*. The residue was purified by flash-column chromatography on silica gel (2%-10% ethyl acetate/*n*-hexane) to afford the corresponding products *rac*-**11**, *rac*-**14**, *rac*-**17a-17g** (37-56% for 2 steps).

3.2.2 Procedure B: General procedure for the synthesis of *rac*-8-9, *rac*-17h-17m

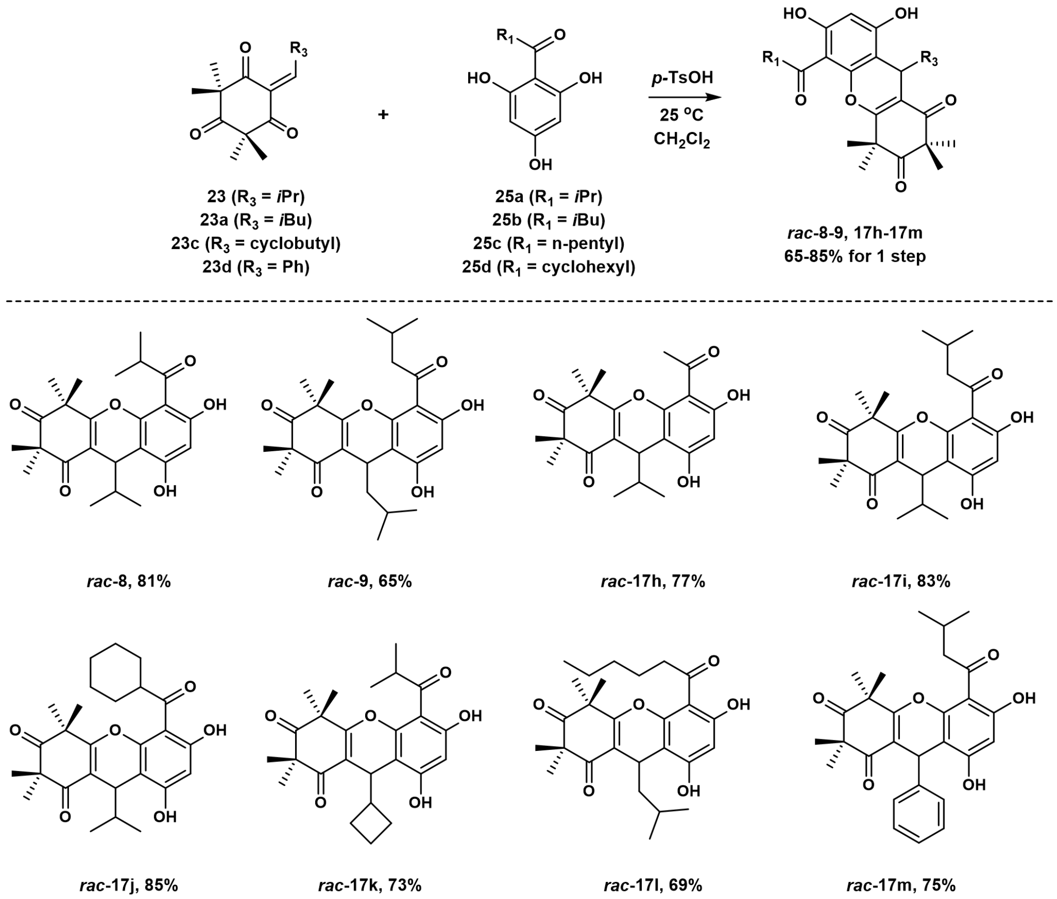

Supplementary Figure 10. Synthesis of *rac*-8-9, *rac*-17h-17m.

To a solution of **23** or **23a**, **23c-23d** (1.5 equiv.) and **25a-25d** (1.0 equiv.) in dichloromethane (CH_2Cl_2) was added *p*-toluenesulfonic acid (*p*-TsOH; 1.0 equiv.) at 25 °C. The resulting mixture was stirred for 12 h, and quenched with saturated aqueous sodium bicarbonate, and extracted with ethyl acetate. The combined organic layers were dried over Na_2SO_4, filtered and concentrated *in vacuo*. The residue was purified by flash-column chromatography on silica gel (2%-10% ethyl acetate/*n*-hexane) to afford the corresponding products *rac*-**8-9**, *rac*-**17h-17m** (65-85%).

3.2.3 Procedure C: Synthesis of *rac*-17n and *rac*-17o

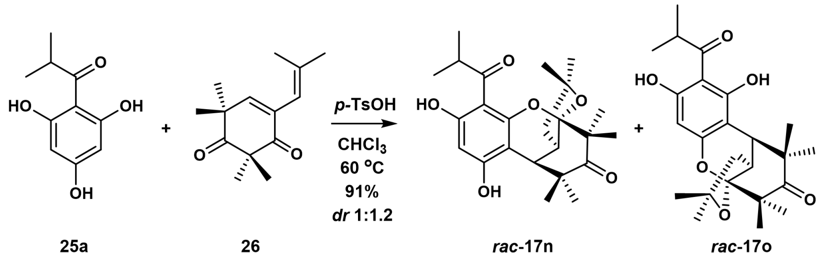

Supplementary Figure 11. Synthesis of *rac*-17n and *rac*-17o.

To a solution of **25a** (98 mg, 0.5 mmol, 1.0 equiv.) and **26**[1] (110 mg, 0.55 mmol, 1.1 equiv.) in trichloromethane ($CHCl_3$; 5 mL) was added *p*-toluenesulfonic acid (*p*-TsOH; 114 mg, 0.6 mmol, 1.2 equiv.) at 25 °C. The resulting mixture was allowed to warm up to 60 °C and stirred for overnight. The resulting mixture was quenched with saturated aqueous sodium bicarbonate (10 mL), and extracted with ethyl acetate (10 mL × 3). The combined organic layers were dried over Na_2SO_4, filtered and concentrated *in vacuo*. The residue was purified by flash-column chromatography on silica gel (2%-10% ethyl acetate/*n*-hexane) to afford *rac*-**17n** (85 mg, 41%) as white solids, and *rac*-**17o** (104 mg, 50%) as white solids.

3.3 Synthesis of (+)- and (-)-8-9, (+)- and (-)-11, (+)- and (-)-14, (+)- and (-)-17a-17o

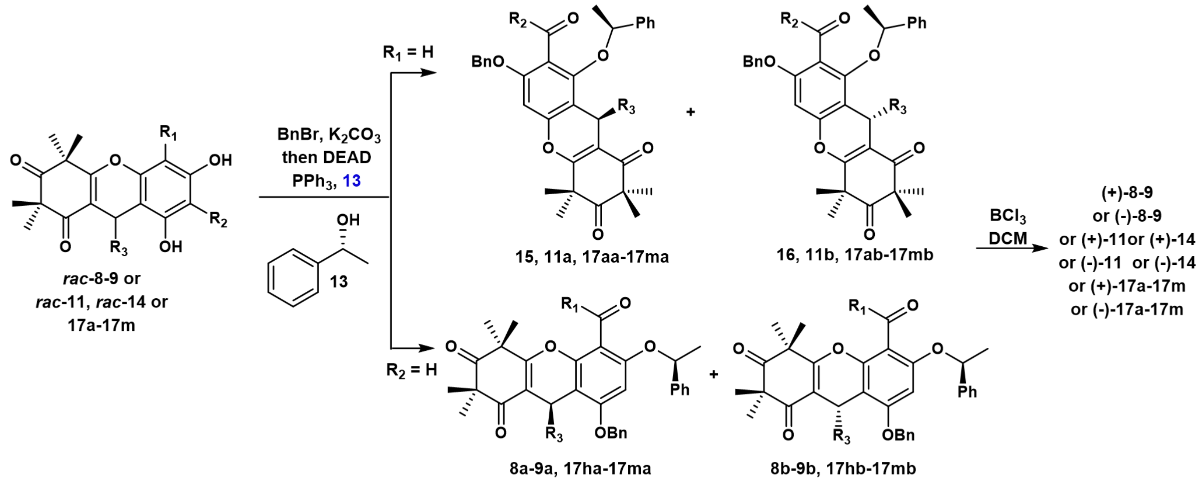

Supplementary Figure 12. Synthesis of (+)- and (-)-8-9, (+)- and (-)-11, (+)- and (-)-14, and (+)- and (-)-17a-17m.

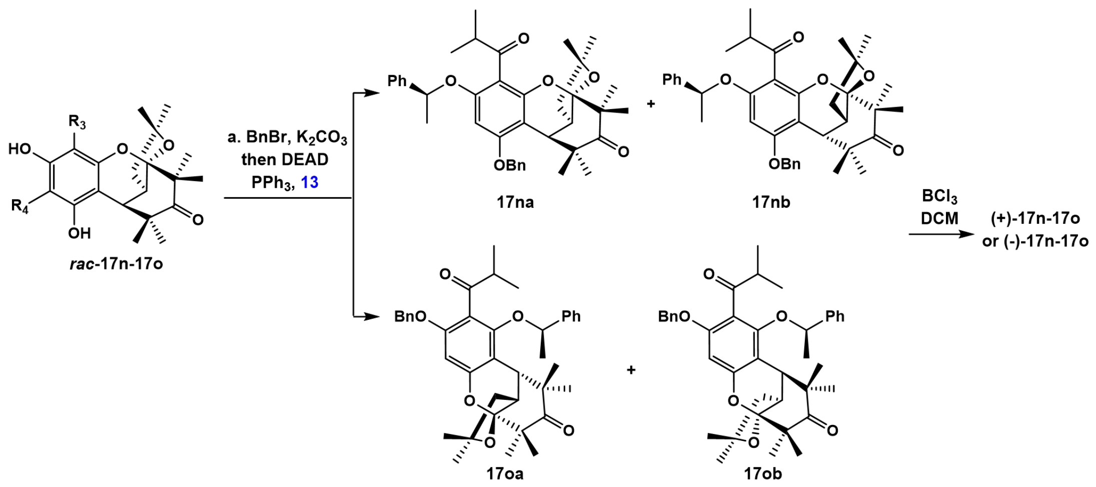

Supplementary Figure 13. Synthesis of (+)- and (-)-17n-17o.

To a solution of *rac*-**8-9** or *rac*-**11** or *rac*-**14** or *rac*-**17a-17o** (1.0 equiv.) and potassium carbonate (K_2CO_3; 1.5 equiv.) in acetone (0.05 M) was added benzyl bromide (BnBr; 1.05 equiv.) at 25 °C. The resulting mixture was allowed to warm up to 60 °C. After the reaction was finished according to TLC, the resulting mixture was directly filtered and concentrated *in vacuo* to afford the crude. Subsequently, to a solution of the crude and commercially available inexpensive compound **13** (1.2 equiv.) as well as triphenylphosphine (PPh_3; 1.5 equiv.) in tetrahydrofuran/methylbenzene (THF/PhMe; v/v 1:1; 0.1 M) were slowly added diethyl azodicarboxylate (DEAD; 1.5 equiv.) at 0 °C. The resulting mixture was allowed to warm up to 25 °C. After the reaction was finished according to TLC, the resulting mixture was quenched with saturated aqueous sodium bicarbonate, and extracted with ethyl acetate. The combined organic layers were dried over Na_2SO_4, filtered and concentrated *in vacuo*. The residue was purified by flash-column chromatography on silica gel (1%-2% ethyl acetate/*n*-hexane) to afford the corresponding products **8a-9a**, **8b-9b**, **11a**, **11b**, **15**, **16**, **17aa-17oa** and **17ab-17ob**.

To a solution of **8a-8a** or **9b-9b** or **11a** or **11b** or **15** or **16** or **17aa-17oa** or **17ab-17ob** in dichloromethane (CH_2Cl_2; 0.1 M) was added boron trichloride (BCl_3; 10 equiv.) at −78 ºC. The resulting mixture was allowed to warm up to −50 ºC. After the reaction was finished according to TLC, the resulting mixture was quenched with saturated aqueous sodium bicarbonate, and extracted with ethyl acetate. The combined organic layers were dried over Na_2SO_4, filtered and concentrated *in vacuo*. The residue was purified by flash-column chromatography on silica gel

(2%-10% ethyl acetate/*n*-hexane) to afford the corresponding products (+)-**8-9** or (+)-**11** or (+)-**14** or (+)-**17a-17o** or (-)-**8-9** or (-)-**11** or (-)-**14** or (-)-**17a-17o**.

Note: (1) When we treated *rac*-**14** (1.0 equiv.) with the following selected chiral reagents (1.0 equiv. or 3.0 equiv.) (Supplementary Figure 14), the diastereoisomers could not be separated by silica gel column chromatography or recrystallization.

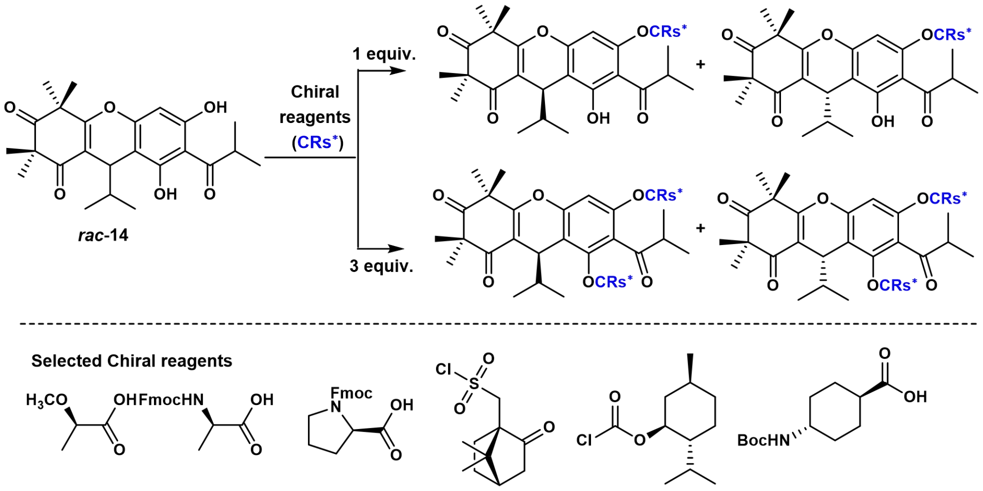

Supplementary Figure 14. Attempts to the chiral resolution of *rac*-14 using different chiral reagents.

(2) When we treated *rac*-**14** (1.0 equiv.) with the chiral reagent **13** (1.05 equiv.) (Supplementary Figure 15) under a standard mitsunobu reaction, the diastereoisomers **A** and **B** could be separated by silica gel column chromatography. However, the compound **A** or **B** was not pure because of the presence of byproducts **C**, **D**, **E**, resulting in the poor *ee* value (*ee*<40%).

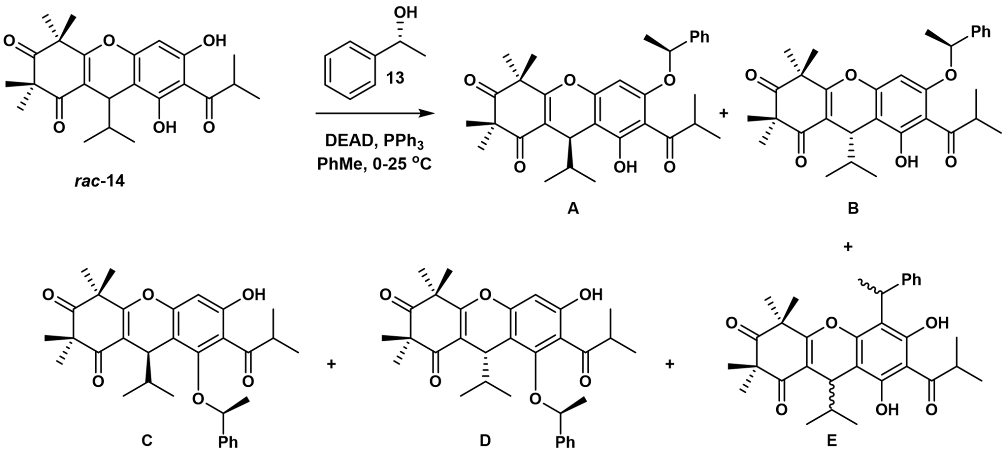

Supplementary Figure 15. Attempts to the chiral resolution of *rac*-14 using chiral 13.

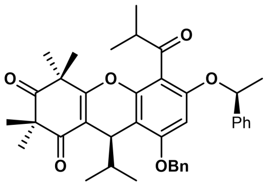

8a

Compound **8a**: 1.03 g, 35% yield, 11 h, white solids (2.0 g scale); $\mathbf{R}_f$ = 0.21 (ethyl acetate/*n*-hexane = 1/12); $[\alpha]_D^{26}$ = −17.6 (*c* = 0.34 in CH_2Cl_2); **IR** (KBr): 3066, 3032, 2967, 2929, 2871, 1707, 1651, 1581, 1466, 1383, 1181, 1105, 804 cm^{-1}; **^{1}H NMR** (400 MHz, $CDCl_3$) δ 7.32 (m, 10H), 6.13 (s, 1H), 5.08 (q, *J* = 6.4 Hz, 1H), 4.84 (s, 2H), 4.31(d, *J* = 3.4 Hz, 1H), 3.21 (m, 1H), 1.88 (m, 1H), 1.54 (d, *J* = 6.4 Hz, 3H), 1.49 (s, 3H), 1.41 (s, 3H), 1.38 (s, 3H), 1.35 (s, 3H), 1.23 (d, *J* = 6.7 Hz, 3H), 1.22 (d, *J* = 6.9 Hz, 3H), 0.72 (d, *J* = 7.0 Hz, 3H), 0.69 (d, *J* = 6.9 Hz, 3H); **^{13}C NMR** (101 MHz, $CDCl_3$) δ 212.7, 207.1, 198.0, 168.6, 157.0, 154.8, 149.3, 142.8, 136.4, 129.0 (2 carbons overlapped), 128.8 (2 carbons overlapped), 128.2, 128.0, 127.1 (2 carbons overlapped), 125.6 (2 carbons overlapped), 113.0, 111.2, 106.9, 96.2, 78.2, 70.4, 56.1, 47.7, 42.6, 35.0, 32.3, 25.0, 24.9, 24.7 (2 carbons overlapped), 24.3, 19.6, 18.8, 18.4, 17.8; **HRMS** (ESI-TOF) m/z: $[M+Na]^+$ calcd. for $C_{39}H_{44}O_6Na$ 631.3030; found, 631.3033.

Note: According to the above procedure, a total of 2.0 g of compound **8a** was prepared readily after 2 simple parallel operations.

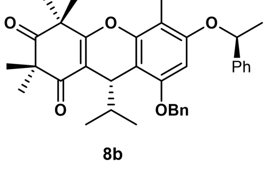

8b

Compound **8b**: 1.17 g, 40% yield, 11 h, white solids (2.0 g scale); $\mathbf{R}_f$ = 0.30 (ethyl acetate/*n*-hexane = 1/12); $[\alpha]_D^{26}$ = −23.9 (*c* = 0.56 in CH_2Cl_2); **IR** (KBr): 3063, 3033, 2971, 2932, 2874, 1711, 1646, 1584, 1466, 1389, 1189, 1070, 814 cm^{-1}; **^{1}H NMR** (400 MHz, $CDCl_3$) δ 7.30 (m, 5H), 7.26 (m, 5H), 6.14 (s, 1H), 5.18 (q, *J* = 6.4 Hz, 1H), 4.83 (d, *J* = 12.3 Hz, 1H), 4.79 (d, *J*

= 12.3 Hz, 1H), 4.27 (d, J = 3.3 Hz, 1H), 3.24 (m, 1H), 1.92 (m, 1H), 1.58 (d, J = 6.4 Hz, 3H), 1.49 (s, 3H), 1.40 (s, 3H), 1.37 (s, 3H), 1.33 (s, 3H), 1.25 (d, J = 4.0 Hz, 3H), 1.23 (d, J = 4.1 Hz, 3H), 0.76 (d, J = 8.1 Hz, 3H), 0.74 (d, J = 7.3 Hz, 3H); **^{13}C NMR** (101 MHz, $CDCl_3$) δ 212.7, 206.8, 197.8, 168.6, 157.0, 154.6, 149.3, 142.4, 136.4, 128.9 (2 carbons overlapped), 128.7 (2 carbons overlapped), 128.1, 127.9, 127.1 (2 carbons overlapped), 125.4 (2 carbons overlapped), 112.7, 111.2, 106.5, 95.8, 77.3, 70.4, 56.0, 47.6, 42.4, 35.0, 32.3, 25.0, 24.9, 24.9, 24.6, 24.4, 19.3, 19.0, 18.4, 17.8; **HRMS** (ESI-TOF) m/z: $[M+Na]^+$ calcd. for $C_{39}H_{44}O_6Na$ 631.3030; found, 631.3031.

Note: According to the above procedure, a total of 2.3 g of compound **8b** was prepared readily after 2 simple parallel operations.

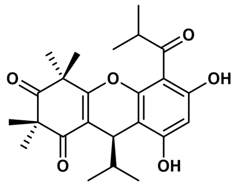

(+)-8

Compound (+)-**8**: 647 mg, 95% yield, 3 h, white solids (1.0 g scale); $[\alpha]_D^{26}$ = +188.9 (c = 0.1 in MeOH); Compound (-)-**8**: 633 mg, 93% yield, 3 h, white solids (1.0 g scale); $[\alpha]_D^{26}$ = −192.1 (c = 0.1 in MeOH); **R_f** = 0.31 (ethyl acetate/n-hexane = 1/5); **IR** (KBr): 3238, 2974, 2927, 2870, 1718, 1626, 1596, 1466, 1399, 1230, 1110, 1038, 830 cm^{-1}; **^{1}H NMR** (400 MHz, $CDCl_3$) δ 13.32 (s, 1H), 6.55 (s, 1H), 6.27 (s, 1H), 4.31 (s, 1H), 3.90 (m, 1H), 1.91 (m, 1H), 1.61 (s, 3H), 1.44 (s, 3H), 1.42 (s, 3H), 1.39 (s, 3H), 1.26 (d, J = 5.8 Hz, 3H), 1.24 (d, J = 6.6 Hz, 3H), 0.83 (d, J = 6.7 Hz, 3H), 0.79 (d, J = 6.8 Hz, 3H); **^{13}C NMR** (101 MHz, $CDCl_3$) δ 212.1, 209.0, 199.5, 168.7, 164.8, 160.5, 153.4, 112.3, 104.1, 103.6, 100.7, 56.2, 47.5, 39.7, 34.9, 31.5, 25.2, 25.1, 25.1, 24.3, 21.0, 19.0, 18.7, 17.8; **HRMS** (ESI-TOF) m/z: $[M+H]^+$ calcd. for $C_{24}H_{31}O_6$ 415.2115; found, 415.2112.

HPLC condition of (+)-8: Phenomenex Lux Cellulose-4 column; acetonitrile/H_2O = 45:55, 1.0 mL/min, λ = 254 nm; major enantiomer: t_R = 18.5 min, minor enantiomer: t_R = 15.7 min, 97% *ee*.

HPLC condition of (-)-8: Phenomenex Lux Cellulose-4 column; acetonitrile/H_2O= 45:55, 1.0 mL/min, λ = 254 nm; minor enantiomer: t_R = 19.0 min, major enantiomer: t_R = 16.1 min, 96% *ee*.

Note: (1) According to the above procedure, a total of 2.5 g of compounds (+)-**8** and (-)-**8** was prepared readily after 2 simple parallel operations.

(2) The absolute configuration of compound (-)-**8** was determined by X-ray diffraction analysis.

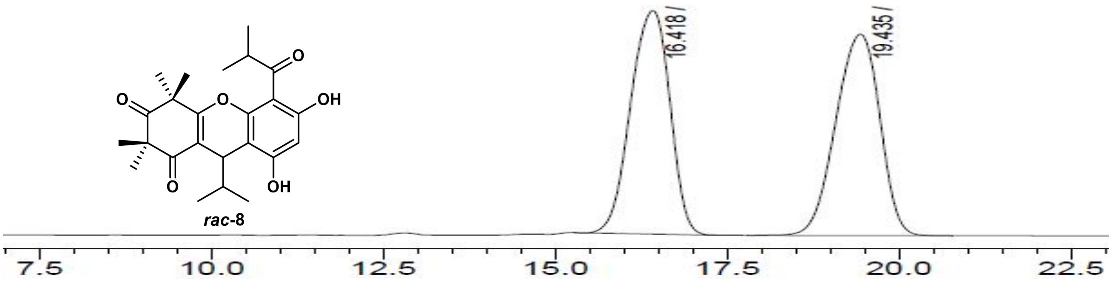

Peak#	Ret. Time	Area	Area%	Height
1	16.418	12903533	49.704	324103
2	19.435	13057301	50.296	291996
Total		25960833	100.000	616100

Supplementary Figure 16. HPLC spectra of *rac*-8.

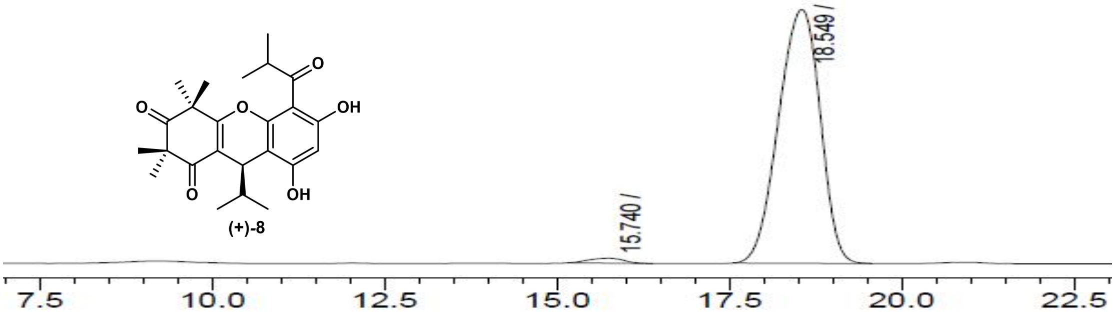

Peak#	Ret. Time	Area	Area%	Height
1	15.740	246310	1.583	7078
2	18.549	15312500	98.417	364798
Total		15558810	100.000	371875

Supplementary Figure 17. HPLC spectra of (+)-8.

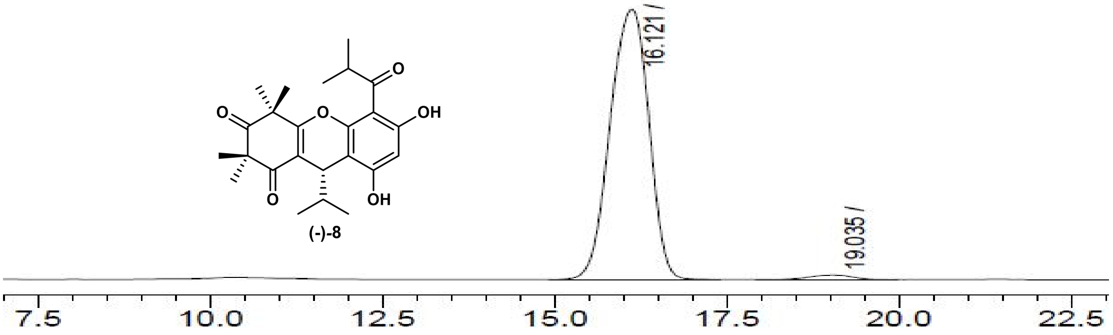

Peak#	Ret. Time	Area	Area%	Height
1	16.121	17549103	98.121	448366
2	19.035	335977	1.879	7892
Total		17885081	100.000	456258

Supplementary Figure 18. HPLC spectra of (-)-8.

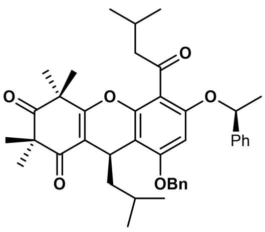

9a

Compound **9a**: 20.4 mg, 32% yield, 11 h, white solids (0.1 mmol scale); **R*f*** = 0.39 (ethyl acetate/*n*-hexane = 1/10); $[\alpha]_D^{26}$ = −23.5 (*c* = 0.4 in CH_2Cl_2); **IR** (KBr): 3068, 3033, 2959, 2927, 2855, 1715, 1649, 1581, 1464, 1385, 1260, 1087, 807 cm^{-1}; **^{1}H NMR** (400 MHz, $CDCl_3$) δ 7.32 (m, 10H), 6.10 (s, 1H), 5.11 (q, *J* = 6.4 Hz, 1H), 4.84 (s, 2H), 4.24 (t, *J* = 5.9 Hz, 1H), 2.86 (dd, *J* = 16.7, 6.8 Hz, 1H), 2.74 (dd, *J* = 16.7, 6.9 Hz, 1H), 2.28 (m, 1H), 1.56 (d, *J* = 6.4 Hz, 3H), 1.48 (s, 3H), 1.43 (s, 3H), 1.36 (s, 3H), 1.35 (s, 3H), 1.29 (m, 3H), 1.03 (d, *J* = 4.8 Hz, 3H), 1.02 (d, *J* = 4.8 Hz, 3H), 0.79 (d, *J* = 6.1 Hz, 3H), 0.68 (d, *J* = 6.1 Hz, 3H); **^{13}C NMR** (101 MHz, $CDCl_3$) δ 212.4, 202.6, 197.8, 167.7, 156.8, 154.7, 148.6, 142.5, 136.1, 128.9 (2 carbons overlapped), 128.6 (2 carbons overlapped), 128.1, 127.9, 127.2 (2 carbons overlapped), 125.4 (2 carbons overlapped), 113.7, 113.7, 108.7, 95.6, 78.0, 70.3, 56.0, 54.3, 47.4, 46.8, 25.4, 25.0, 24.8, 24.6, 24.6 (2 carbons overlapped), 24.3, 24.3, 23.2, 23.1, 22.9, 22.8; **HRMS** (ESI-TOF) m/z: $[M+Na]^+$ calcd. for $C_{41}H_{48}O_6Na$ 659.3343; found, 659.3357.

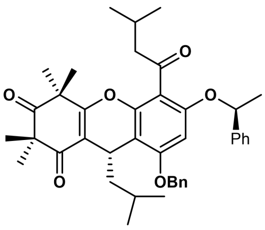

9b

Compound **9b**: 24.2 mg, 38% yield, 11 h, white solids (0.1 mmol scale); **R*f*** = 0.48 (ethyl acetate/*n*-hexane = 1/10); $[\alpha]_D^{26}$ = −39.5 (*c* = 0.2 in CH_2Cl_2); **IR** (KBr): 3063, 3033, 2962, 2932, 2870, 1711, 1649, 1586, 1461, 1382, 1181, 1082, 802 cm^{-1}; **^{1}H NMR** (400 MHz, $CDCl_3$) δ 7.30 (m, 10H), 6.10 (s, 1H), 5.17 (q, *J* = 6.3 Hz, 1H), 4.81 (s, 2H), 4.21 (t, *J* = 5.5 Hz, 1H), 2.87 (dd, *J* = 16.6, 6.8 Hz, 1H), 2.75 (dd, *J* = 16.6, 6.9 Hz, 1H), 2.31 (m, 1H), 1.59 (d, *J* = 6.4 Hz, 3H), 1.48 (s, 3H), 1.43 (s, 3H), 1.39 (m, 3H), 1.35 (s, 3H), 1.33 (s, 3H), 1.03 (d, *J* = 6.3 Hz, 3H), 1.02 (d, *J* =

5.5 Hz, 3H), 0.82 (d, J = 5.9 Hz, 3H), 0.74 (d, J = 5.8 Hz, 3H); **^{13}C NMR** (101 MHz, $CDCl_3$) δ 212.5, 202.4, 197.7, 167.7, 156.9, 154.7, 148.6, 142.4, 136.3, 129.0 (2 carbons overlapped), 128.7 (2 carbons overlapped), 128.2, 128.0, 127.3 (2 carbons overlapped), 125.4 (2 carbons overlapped), 113.6, 113.5, 108.5, 95.7, 77.5, 70.5, 56.1, 54.4, 47.5, 46.6, 25.6, 25.2, 24.9, 24.8, 24.8, 24.7, 24.6, 24.1, 23.5, 23.3, 23.0, 22.9; **HRMS** (ESI-TOF) m/z: $[M+Na]^+$ calcd. for $C_{41}H_{48}O_6Na$ 659.3343; found, 659.3341.

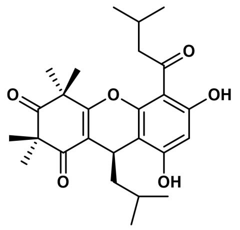

(+)-9

Compound (+)-**9**: 13.2 mg, 95% yield, 3 h, white solids (20 mg scale); $[\alpha]_D^{26}$ = +162.8 (c = 0.1 in MeOH); Compound (-)-**9**: 13.1 mg, 94% yield, 3 h, white solids (20 mg scale); $[\alpha]_D^{26}$ = −160.3 (c = 0.1 in MeOH); **R_f** = 0.40 (ethyl acetate/n-hexane = 1/5); **IR** (KBr): 3385, 2957, 2936, 2860, 1718, 1628, 1466, 1382, 1243, 1119, 1004, 837 cm^{-1}; **^{1}H NMR** (400 MHz, $CDCl_3$) δ 13.48 (s, 1H), 7.33 (s, 1H), 6.27 (s, 1H), 4.30 (t, J = 6.0 Hz, 1H), 3.18 (dd, J = 17.2, 7.4 Hz, 1H), 2.95 (dd, J = 17.2, 6.2 Hz, 1H), 2.37 (m, 1H), 1.64 (s, 3H), 1.47 (s, 3H), 1.43 (s, 3H), 1.41 (m, 3H), 1.39 (s, 3H), 1.03 (d, J = 6.7 Hz, 3H), 1.00 (d, J = 6.6 Hz, 3H), 0.87 (d, J = 6.6 Hz, 3H), 0.86 (d, J = 6.5 Hz, 3H); **^{13}C NMR** (101 MHz, $CDCl_3$) δ 211.9, 204.1, 198.5, 167.4, 164.4, 159.8, 153.2, 114.7, 106.1, 105.7, 100.3, 56.3, 53.6, 47.4, 47.0, 27.1, 25.5, 25.2, 24.9, 24.9, 24.6, 24.6, 24.4, 23.5, 23.6, 23.0, 22.8; **HRMS** (ESI-TOF) m/z: $[M+H]^+$ calcd. for $C_{26}H_{35}O_6$ 443.2428; found, 443.2426.

HPLC condition of (+)-9: Phenomenex Lux Cellulose-4 column; acetonitrile/H_2O = 50:50, 1.0 mL/min, λ = 254 nm; major enantiomer: t_R = 73.6 min, minor enantiomer: t_R = 54.5 min, 98.5% *ee*.

HPLC condition of (-)-9: Phenomenex Lux Cellulose-4 column; acetonitrile/H_2O= 50:50, 1.0 mL/min, λ = 254 nm; minor enantiomer: t_R = 74.2 min, major enantiomer: t_R = 54.1min, 99% *ee*.

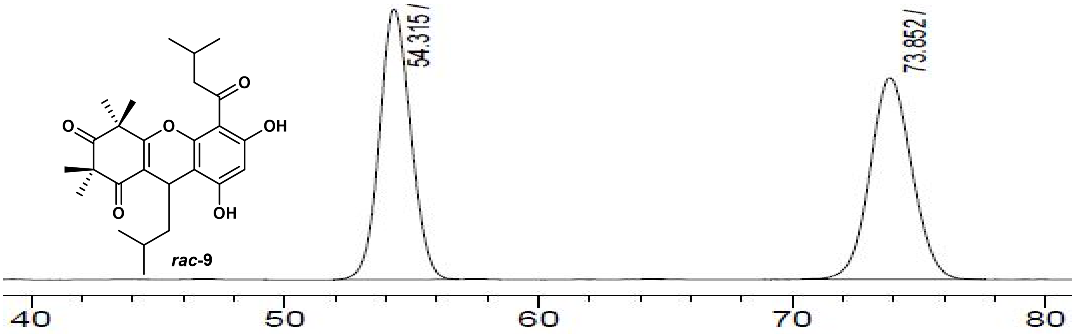

Peak#	Ret. Time	Area	Area%	Height
1	54.315	9177653	49.995	107188
2	73.852	9179344	50.005	79788
Total		18356998	100.000	186976

Supplementary Figure 19. HPLC spectra of *rac*-9.

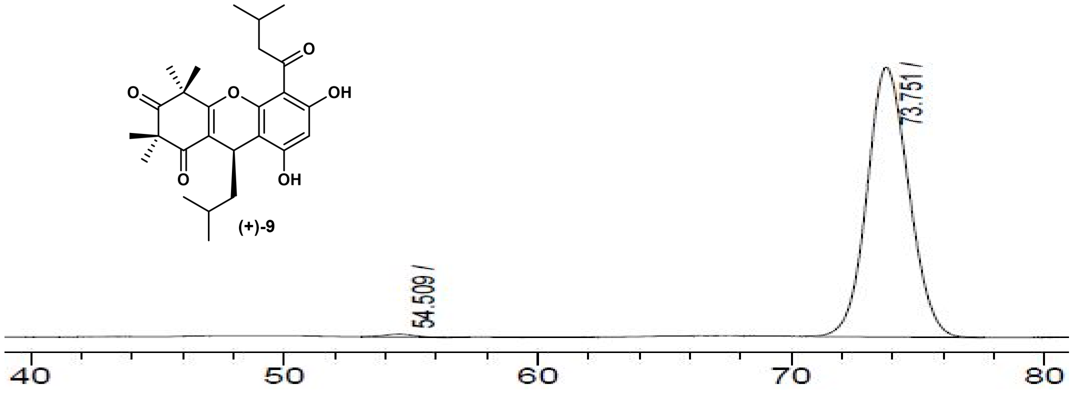

Peak#	Ret. Time	Area	Area%	Height
1	54.509	94488	0.766	1223
2	73.751	12236159	99.234	106606
Total		12330647	100.000	107829

Supplementary Figure 20. HPLC spectra of (+)-9.

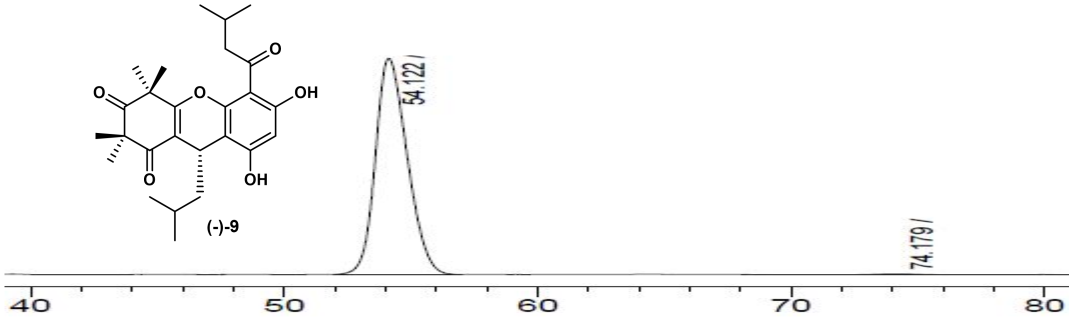

Peak#	Ret. Time	Area	Area%	Height
1	54.122	19801726	99.635	226846
2	74.179	72559	0.365	688
Total		19874285	100.000	227534

Supplementary Figure 21. HPLC spectra of (-)-9.

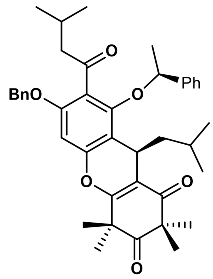

11a

Compound **11a**: 1.18 g, 41% yield, 11 h, white foams (2.0 g scale); **R$_f$** = 0.29 (ethyl acetate/*n*-hexane = 1/12); $[\alpha]_D^{26}$ = +28.0 (*c* = 0.3 in CH_2Cl_2); **IR** (KBr): 3066, 3032, 2959, 2923, 2874, 1715, 1646, 1581, 1459, 1381, 1262,1092, 800 cm^{-1}; **^{1}H NMR** (400 MHz, $CDCl_3$) δ 7.35 (m, 7H), 7.15 (m, 3H), 6.47 (s, 1H), 5.05 (m, 3H), 3.71 (t, *J* = 6.4 Hz, 1H), 2.75 (m, 2H), 2.21 (m, 1H), 1.64 (d, *J* = 6.5 Hz, 3H), 1.47 (s, 3H), 1.35 (s, 3H), 1.33 (s, 3H), 1.33 (m, 3H), 1.29 (s, 3H), 0.98 (d, *J* = 6.7 Hz, 3H), 0.94 (d, *J* = 6.7 Hz, 3H), 0.83 (d, *J* = 6.5 Hz, 3H), 0.78 (d, *J* = 6.6 Hz, 3H); **^{13}C NMR** (101 MHz, $CDCl_3$) δ 212.5, 204.6, 196.6, 167.2, 154.7, 152.9, 152.5, 140.1, 136.2, 128.7 (2 carbons overlapped), 128.3 (2 carbons overlapped), 128.3, 128.2, 127.5 (2 carbons overlapped), 127.4 (2 carbons overlapped), 123.4, 114.1, 113.7, 96.9, 84.0, 70.9, 55.9, 54.1, 47.5, 47.1, 26.2, 25.2, 24.9, 24.8, 24.6, 24.5, 23.9, 23.4, 23.0, 22.9, 22.8, 21.4; **HRMS** (ESI-TOF) m/z: $[M+Na]^+$ calcd. for $C_{41}H_{48}O_6Na$ 659.3343; found, 659.3339.

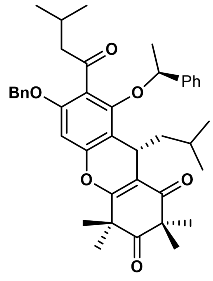

11b

Compound **11b**: 1.06 g, 37% yield, 11 h, white foams (2.0 g scale); **R$_f$** = 0.23 (ethyl acetate/*n*-hexane = 1/12); $[\alpha]_D^{26}$ = −33.0 (*c* = 0.2 in CH_2Cl_2); **IR** (KBr): 3066, 3035, 2957, 2925, 2867, 1711, 1649, 1579, 1461, 1383, 1261, 1082, 800 cm^{-1}; **^{1}H NMR** (400 MHz, $CDCl_3$) δ 7.37 (m, 10H), 6.57 (s, 1H), 5.07 (m, 3H), 4.47 (t, *J* = 6.1 Hz, 1H), 2.49 (dd, *J* = 17.5, 6.4 Hz, 1H), 2.38 (dd, *J* = 17.5, 6.7 Hz, 1H), 1.93 (m, 1H), 1.61 (s, 3H), 1.54 (d, *J* = 6.4 Hz, 3H), 1.49 (s, 3H), 1.44 (s, 3H), 1.43 (m, 3H), 1.41 (s, 3H), 0.96 (d, *J* = 6.3 Hz, 3H), 0.87 (d, *J* = 6.2 Hz, 3H), 0.78 (d, *J* =

6.6 Hz, 3H), 0.69 (d, J = 6.7 Hz, 3H); **^{13}C NMR** (101 MHz, $CDCl_3$) δ 212.4, 204.3, 197.6, 167.7, 154.9, 153.1, 152.6, 142.3, 136.1, 128.7 (2 carbons overlapped), 128.5 (2 carbons overlapped), 128.2, 128.0, 127.4 (2 carbons overlapped), 126.4 (2 carbons overlapped), 123.5, 114.2, 113.8, 96.9, 84.5, 70.9, 56.2, 53.6, 47.4, 47.2, 26.6, 25.1, 25.1, 25.1, 24.7 (2 carbons overlapped), 24.0, 23.6, 23.4, 22.7, 22.6, 22.5; **HRMS** (ESI-TOF) m/z: $[M+Na]^+$ calcd. for $C_{41}H_{48}O_6Na$ 659.3343; found, 659.3332.

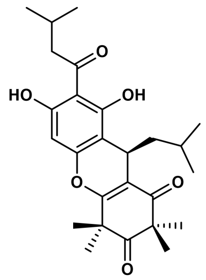

(+)-11

Compound (+)-**11**: 646 mg, 93% yield, 2 h, yellow solids (1.0 g scale); **$[\alpha]_D^{26}$** = +143.0 (c = 0.1 in MeOH); Compound (-)-**11**: 667 mg, 96% yield, 2 h, yellow solids (1.0 g scale); **$[\alpha]_D^{26}$** =−129.4 (c = 0.1 in MeOH); **R_f** = 0.31 (ethyl acetate/n-hexane = 1/5); **IR** (KBr): 3440, 2957, 2924, 2871, 1718, 1631, 1429, 1285, 1170, 1087, 827 cm^{-1}; **^{1}H NMR** (500 MHz, $CDCl_3$) δ 13.03 (s, 1H), 7.56 (s, 1H), 6.11 (s, 1H), 4.27 (t, J = 5.3 Hz, 1H), 2.99 (m, 3H), 2.27 (m, 1H), 1.71 (s, 3H), 1.55 (s, 3H), 1.43 (m, 3H), 1.43 (s, 3H), 1.41 (s, 3H), 1.37 (s, 3H), 0.99 (s, 3H), 0.97 (s, 3H), 0.87 (d, J = 5.8 Hz, 3H), 0.83 (d, J = 5.6 Hz, 3H); **^{13}C NMR** (126 MHz, $CDCl_3$) δ 212.2, 206.5, 197.9, 167.1, 162.6, 158.5, 155.6, 114.2, 107.6, 106.6, 94.8, 56.1, 53.2, 47.2, 45.7, 25.2, 25.1 (2 carbons overlapped), 24.7, 24.6, 24.6, 24.2, 23.5, 23.2, 22.8, 22.8; **HRMS** (ESI-TOF) m/z: $[M+H]^+$ calcd. for $C_{26}H_{35}O_6$ 443.2428; found, 443.2431.

HPLC condition of (+)-**11**: Phenomenex Lux Cellulose-3 column; acetonitrile/H_2O = 50:50, 1.0 mL/min, λ = 254 nm; major enantiomer: t_R = 23.0 min, minor enantiomer: t_R = 26.6 min, 97.5% *ee*.

HPLC condition of (-)-11: Phenomenex Lux Cellulose-3 column; acetonitrile/H_2O= 50:50, 1.0 mL/min, λ = 254 nm; minor enantiomer: t_R = 22.0 min, major enantiomer: t_R = 26.4 min, 95.5% *ee*.

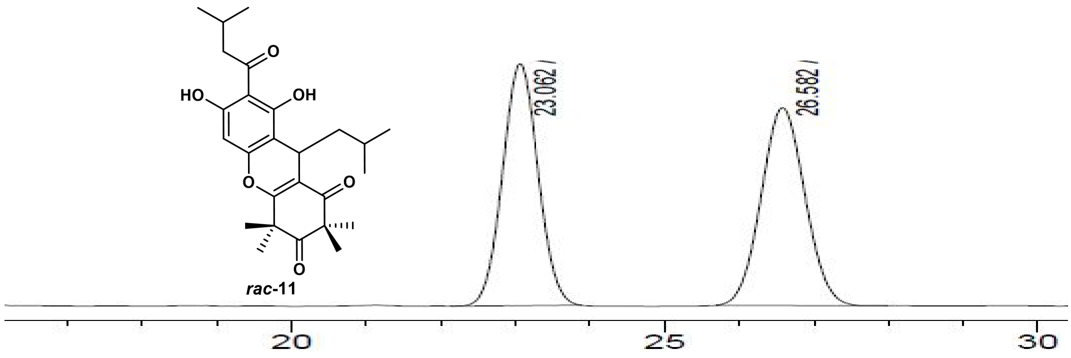

Peak#	Ret. Time	Area	Area%	Height
1	23.062	6092873	50.004	182153
2	26.582	6091859	49.996	148945
Total		12184733	100.000	331098

Supplementary Figure 22. HPLC spectra of *rac*-11.

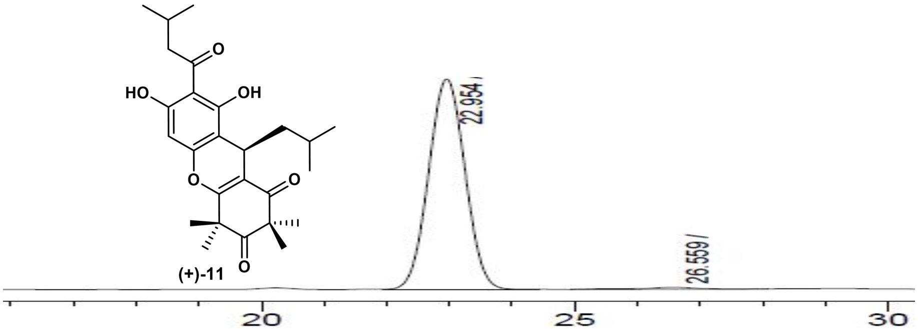

Peak#	Ret. Time	Area	Area%	Height
1	22.954	7821542	98.766	193941
2	26.559	97736	1.234	1638
Total		7919278	100.000	195579

Supplementary Figure 23. HPLC spectra of (+)-11.

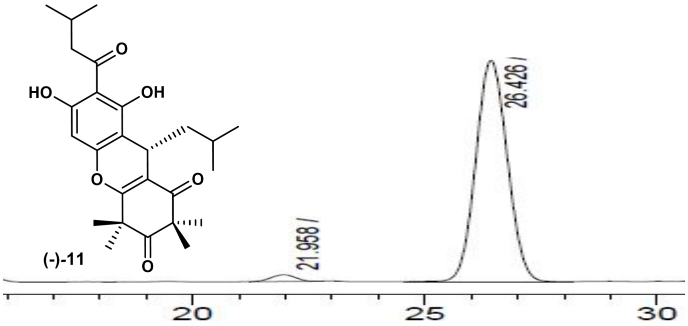

Peak#	Ret. Time	Area	Area%	Height
1	21.958	276823	2.283	7594
2	26.426	11849570	97.717	251070
Total		12126393	100.000	258664

Supplementary Figure 24. HPLC spectra of (-)-11.

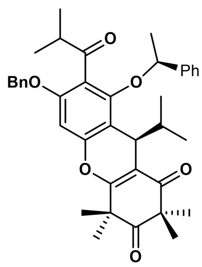

15

Compound **15**: 1.23 g, 42% yield, 11 h, white solids (2.0 g scale); **R***f* = 0.33 (ethyl acetate/*n*-hexane = 1/12); $[\alpha]_D^{26}$ = +40.0 (*c* = 0.2 in CH_2Cl_2); **IR** (KBr): 3066, 3033, 2962, 2925, 2853, 1707, 1651, 1579, 1459, 1378, 1260, 1040, 804 cm^{-1}; **^{1}H NMR** (400 MHz, $CDCl_3$) δ 7.35 (m, 7H), 7.19 (m, 3H), 6.48 (s, 1H), 5.06 (m, 3H), 3.79 (d, *J* = 3.2 Hz, 1H), 3.13 (m, 1H), 1.70 (m, 1H), 1.58 (s, 3H), 1.51 (s, 3H), 1.35 (s, 6H), 1.30 (s, 3H), 1.23 (d, *J* = 6.8 Hz, 3H), 1.13 (d, *J* = 7.0 Hz, 3H), 0.69 (d, *J* = 6.8 Hz, 3H), 0.52 (d, *J* = 6.8 Hz, 3H); **^{13}C NMR** (101 MHz, $CDCl_3$) δ 212.5, 208.7, 196.7, 167.7, 154.7, 153.3, 140.6, 136.2, 128.6 (2 carbons overlapped), 128.3 (2 carbons overlapped), 128.2, 128.1, 127.3 (2 carbons overlapped), 127.2 (2 carbons overlapped), 123.0, 111.5, 111.3, 111.3, 96.4, 83.9, 70.7, 55.9, 47.2, 42.6, 35.4, 33.1, 25.2, 24.8, 24.6, 24.4, 21.7, 19.4, 18.7, 18.2, 17.7; **HRMS** (ESI-TOF) m/z: $[M+Na]^+$ calcd. for $C_{39}H_{44}O_6Na$ 631.3030; found, 631.3036.

Note: 1. According to the above procedure, a total of 10 g of compound **15** was prepared readily after 9 simple parallel operations.

2. Compounds **15** and **16** could be separated by recrystallization in CH_2Cl_2/*n*-hexane.

3. The absolute configuration of compound **15** was determined by X-ray diffraction analysis.

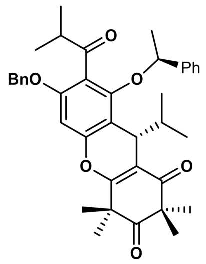

16

Compound **16**: 1.12 g, 38% yield, 11 h, white foams (2.0 g scale); **R***f* = 0.26 (ethyl acetate/*n*-hexane = 1/12); $[\alpha]_D^{26}$ = −27.1 (*c* = 0.1 in CH_2Cl_2); **IR** (KBr): 3063, 3033, 2962, 2925, 2851, 1704, 1651, 1579, 1461, 1381, 1260, 1070, 804 cm^{-1}; **^{1}H NMR** (400 MHz, $CDCl_3$) δ 7.24

(m, 10H), 6.46 (s, 1H), 4.96 (m, 3H), 4.36 (d, *J* = 3.9 Hz, 1H), 2.79 (m, 1H), 1.81 (m, 1H), 1.51 (s, 3H), 1.36 (s, 3H), 1.35 (d, *J* = 8.5 Hz, 3H), 1.34 (s, 3H), 1.28 (s, 3H), 0.87 (d, *J* = 7.1 Hz, 3H), 0.79 (d, *J* = 6.8 Hz, 3H), 0.72 (d, *J* = 6.8 Hz, 3H), 0.65 (d, *J* = 6.9 Hz, 3H); **^{13}C NMR** (101 MHz, $CDCl_3$) δ 212.5, 208.5, 197.8, 168.3, 155.0, 153.3, 153.1, 142.5, 136.3, 128.7 (2 carbons overlapped), 128.5 (2 carbons overlapped), 128.1, 127.9, 127.1 (2 carbons overlapped), 126.3 (2 carbons overlapped), 122.7, 112.2, 111.7, 96.5, 84.3, 70.8, 56.2, 47.5, 42.3, 35.6, 33.4, 25.4, 25.3, 24.7, 24.0, 22.5, 19.2 (2 carbons overlapped), 18.2, 17.1; **HRMS** (ESI-TOF) m/z: $[M+Na]^+$ calcd. for $C_{39}H_{44}O_6Na$ 631.3030; found, 631.3033.

Note: According to the above procedure, a total of 9.9 g of compound **16** was prepared readily after 9 simple parallel operations.

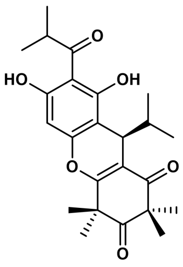

(+)-14

Compound (+)-**14**: 654 mg, 96% yield, 2 h, yellow solids (1.0 g scale); $[\alpha]_D^{26}$ = +97.3 (*c* = 0.1 in MeOH); Compound (-)-**14**: 654 mg, 96% yield, 2 h, yellow solids (1.0 g scale); $[\alpha]_D^{26}$ = −108.6 (*c* = 0.1 in MeOH); **R*f*** = 0.30 (ethyl acetate/*n*-hexane = 1/5); **IR** (KBr): 3300, 2975, 2873, 1718, 1631, 1469, 1261, 1106, 963, 826 cm^{-1}; **^{1}H NMR** (400 MHz, $CDCl_3$) δ 12.87 (s, 1H), 8.61 (s, 1H), 6.22 (s, 1H), 4.39 (d, *J* = 3.5 Hz, 1H), 3.95 (m, 1H), 2.01 (m, 1H), 1.59 (s, 3H), 1.45 (s, 3H), 1.43 (s, 3H), 1.39 (s, 3H), 1.21 (d, *J* = 5.8 Hz, 3H), 1.20 (d, *J* = 5.8 Hz, 3H), 0.79 (d, *J* = 6.1 Hz, 3H), 0.78 (d, *J* = 6.1 Hz, 3H); **^{13}C NMR** (101 MHz, $CDCl_3$) δ 212.4, 211.5, 198.9, 168.8, 162.6, 159.2, 156.6, 112.0, 107.2, 104.7, 95.0 56.2, 47.6, 39.9, 34.7, 32.0, 25.3, 25.3, 24.7, 24.2, 19.5, 19.5, 19.2, 18.9; **HRMS** (ESI-TOF) m/z: $[M-H]^-$ calcd. for $C_{24}H_{29}O_6$ 413.1964; found, 413.1960.

HPLC condition of (+)-14: Phenomenex Lux Cellulose-3 column; acetonitrile/H_2O= 45:55, 1.0 mL/min, λ = 254 nm; major enantiomer: t_R = 52.2 min, >99 *ee*.

HPLC condition of (-)-14: Phenomenex Lux Cellulose-3 column; acetonitrile/H_2O= 45:55, 1.0 mL/min, λ = 254 nm; major enantiomer: t_R = 58.2 min, >99 *ee*.

Note: (1) The absolute configurations of (+)**-14** and (-)**-14** were determined to be respectively *R* and *S* based on X-ray diffraction analysis.

(2) According to the above procedure, a total of 13.0 g of compound (+)**-14** and (-)**-14** was prepared readily after 10 simple parallel operations.

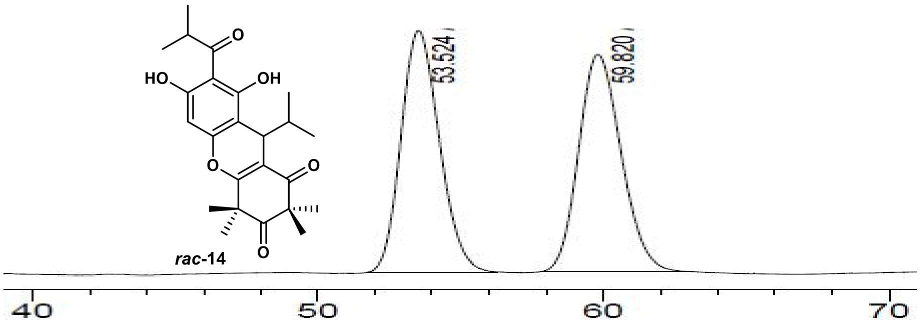

Peak#	Ret. Time	Area	Area%	Height
1	53.524	2615812	50.074	27942
2	59.820	2608119	49.926	25125
Total		5223931	100.000	53067

Supplementary Figure 25. HPLC spectra of *rac*-14.

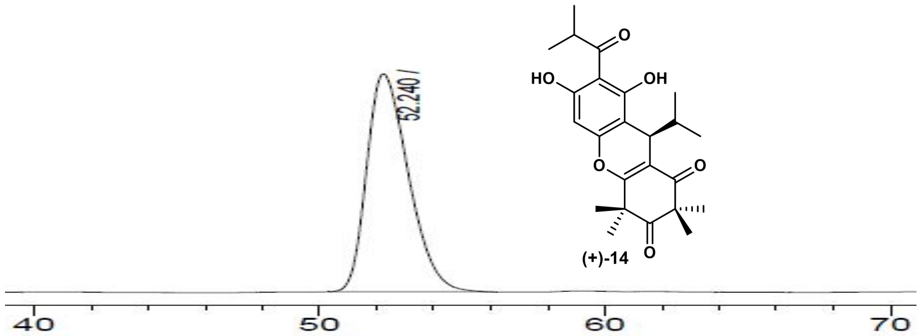

Peak#	Ret. Time	Area	Area%	Height
1	52.240	10104297	100.000	99321
Total		10104297	100.000	99321

Supplementary Figure 26. HPLC spectra of (+)-14.

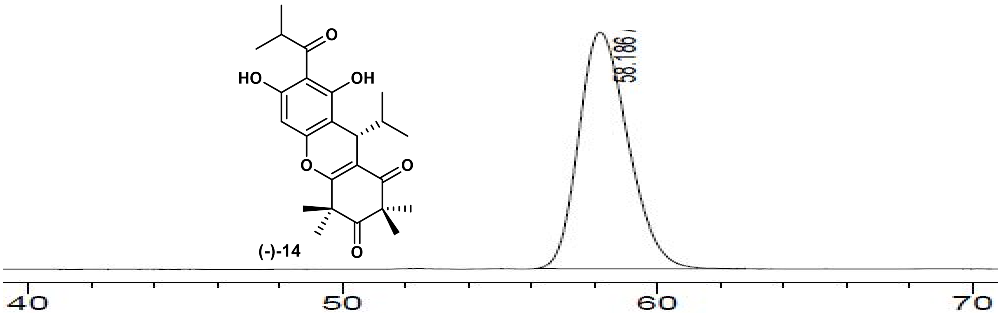

Peak#	Ret. Time	Area	Area%	Height
1	58.186	4825463	100.000	44312
Total		4825463	100.000	44312

Supplementary Figure 27. HPLC spectra of (-)-14.

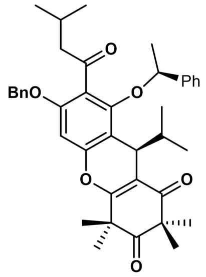

17aa

Compound **17aa**: 1.13 g, 39% yield, 11 h, white foams (2.0 g scale); **R*f*** = 0.33 (ethyl acetate/*n*-hexane = 1/12); $[\alpha]_D^{26}$ = +32.1 (*c* = 0.2 in CH_2Cl_2); **IR** (KBr): 3068, 3033, 2957, 2925, 2853, 1718, 1651, 1581, 1461, 1383, 1260, 1027, 804 cm^{-1}; **^{1}H NMR** (400 MHz, $CDCl_3$) δ 7.36 (m, 7H), 7.20 (m, 3H), 6.47 (s, 1H), 5.06 (m, 3H), 3.84 (d, *J* = 2.8 Hz, 1H), 2.75 (m, 2H), 2.23 (m, 1H), 1.62 (m, 1H), 1.61 (d, *J* = 6.4 Hz, 3H), 1.51 (s, 3H), 1.35 (s, 6H), 1.31 (s, 3H), 0.98 (d, *J* = 6.5 Hz, 3H), 0.94 (d, *J* = 6.6 Hz, 3H), 0.68 (d, *J* = 6.7 Hz, 3H), 0.53 (d, *J* = 6.8 Hz, 3H); **^{13}C NMR** (101 MHz, $CDCl_3$) δ 212.6, 204.5, 196.9, 167.8, 154.7, 153.3 (2 carbons overlapped), 140.7, 136.2, 128.7 (2 carbons overlapped), 128.4 (2 carbons overlapped), 128.2 (2 carbons overlapped), 127.4 (2 carbons overlapped), 127.4 (2 carbons overlapped), 123.8, 111.5 (2 carbons overlapped), 96.6, 83.9, 71.0, 56.0, 54.2, 47.3, 35.4, 33.2, 25.2, 24.7, 24.7, 24.6, 24.5, 22.9, 22.9, 21.8, 19.2, 18.9; **HRMS** (ESI-TOF) m/z: $[M+Na]^+$ calcd. for $C_{40}H_{46}O_6Na$ 645.3187; found, 645.3176.

Note: According to the above procedure, a total of 2.2 g of compound **17aa** was prepared readily after 2 simple parallel operations.

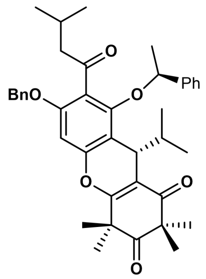

17ab

Compound **17ab**: 1.04 g, 36% yield, 11 h, white foams (2.0 g scale); **R*f*** = 0.24 (ethyl acetate/*n*-hexane = 1/12); **[α]$_D^{26}$** = −67.3 (*c* = 0.1 in CH_2Cl_2); **IR** (KBr): 3063, 3033, 2957, 2923, 2871, 1713, 1646, 1584, 1459, 1383, 1260, 1027, 802 cm^{-1}; **^{1}H NMR** (400 MHz, $CDCl_3$) δ 7.35 (m, 10H), 6.54 (s, 1H), 5.04 (m, 3H), 4.46 (d, *J* = 3.3 Hz, 1H), 2.51 (dd, *J* = 17.5, 6.4 Hz, 1H), 2.31 (dd, *J* = 17.6, 6.6 Hz, 1H), 1.95 (m, 2H), 1.63 (s, 3H), 1.50 (d, *J* = 6.3 Hz, 3H), 1.46 (s, 3H), 1.44 (s, 3H), 1.38 (s, 3H), 0.80 (d, *J* = 7.0 Hz, 3H), 0.77 (d, *J* = 7.0 Hz, 3H), 0.76 (d, *J* = 6.6 Hz, 3H), 0.68 (d, *J* = 6.6 Hz, 3H); **^{13}C NMR** (101 MHz, $CDCl_3$) δ 212.5, 204.1, 197.8, 168.3, 155.0, 153.3, 153.2, 142.4, 136.1, 128.7 (2 carbons overlapped), 128.6 (2 carbons overlapped), 128.2, 128.0, 127.4 (2 carbons overlapped), 126.4 (2 carbons overlapped), 123.7, 112.3, 111.6, 96.6, 84.4, 71.0, 56.2, 53.6, 47.6, 35.6, 33.4, 25.5, 25.4, 24.7, 24.0, 23.9, 22.7, 22.7, 22.5, 19.5, 18.9; **HRMS** (ESI-TOF) m/z: [M+Na]$^+$ calcd. for $C_{40}H_{46}O_6Na$ 645.3187; found, 645.3187.

Note: According to the above procedure, a total of 2.0 g of compound **17ab** was prepared readily after 2 simple parallel operations

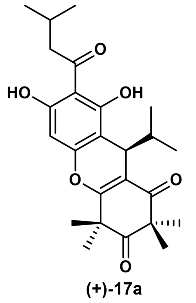

(+)-17a

Compound (+)**-17a**: 654 mg, 95% yield, 2 h, yellow solids (1.0 g scale); **[α]$_D^{26}$** = +106.1 (*c* = 0.1 in MeOH); Compound **(-)-17a**: 660 mg, 96% yield, 2 h, yellow solids (1.0 g scale); **[α]$_D^{26}$** = −95.9 (*c* = 0.1 in MeOH); **R*f*** = 0.30 (ethyl acetate/*n*-hexane = 1/5); **IR** (KBr): 3246, 2959, 2934, 2871, 1718, 1631, 1389, 1246.1163, 1008, 832 cm^{-1}; **^{1}H NMR** (400 MHz, $CDCl_3$) δ 12.96 (s, 1H), 8.74

(s, 1H), 6.23 (s, 1H), 4.39 (d, J = 3.4 Hz, 1H), 3.02 (m, J = 15.7, 6.7 Hz, 2H), 2.29 (m, 1H), 1.99 (m, 1H), 1.59 (s, 3H), 1.44 (d, J = 8.7 Hz, 6H), 1.39 (s, 3H), 0.98 (d, J = 6.6 Hz, 6H), 0.79 (t, J = 6.5 Hz, 6H); **^{13}C NMR** (101 MHz, $CDCl_3$) δ 212.3, 206.9, 199.0, 168.9, 162.5, 159.4, 156.6, 112.0, 108.1, 104.6, 94.9, 56.2, 53.4, 47.6, 34.7, 31.9, 25.3 (3 carbons overlapped), 24.7, 24.2, 23.0, 22.9, 19.5, 18.9; **HRMS** (ESI-TOF) m/z: $[M+H]^+$ calcd. for $C_{25}H_{33}O_6$ 429.2272; found, 429.2285.

HPLC condition of (+)**-17a**: Phenomenex Lux Cellulose-3 column; acetonitrile/H_2O = 48:52, 1.0 mL/min, λ = 254 nm; major enantiomer: t_R = 27.5 min, minor enantiomer: t_R = 30.3 min, 96.5% *ee*.

HPLC condition of (-)**-17a**: Phenomenex Lux Cellulose-3 column; acetonitrile/H_2O= 48:52, 1.0 mL/min, λ = 254 nm; minor enantiomer: t_R = 30.1 min, major enantiomer: t_R = 27.8 min, 92% *ee*.

Note: According to the above procedure, a total of 2.6 g of compounds (+)**-17a** and **(-)-17a** was prepared readily after 2 simple parallel operations.

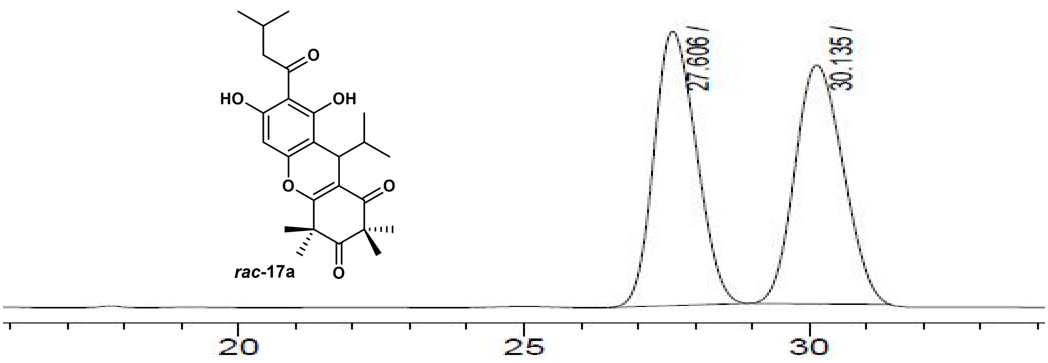

Peak#	Ret. Time	Area	Area%	Height
1	27.606	47728616	50.121	924046
2	30.135	47498263	49.879	804835
Total		95226879	100.000	1728881

Supplementary Figure 28. HPLC spectra of *rac*-17a.

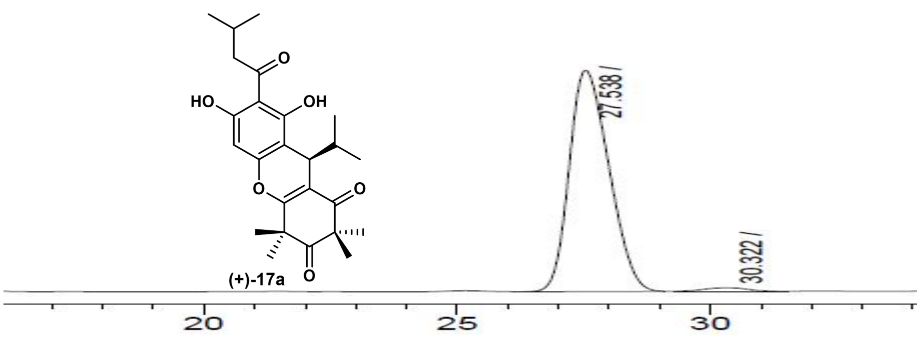

Peak#	Ret. Time	Area	Area%	Height
1	27.538	69993840	98.261	1282600
2	30.322	1238858	1.739	21391
Total		71232699	100.000	1303990

Supplementary Figure 29. HPLC spectra of (+)-17a.

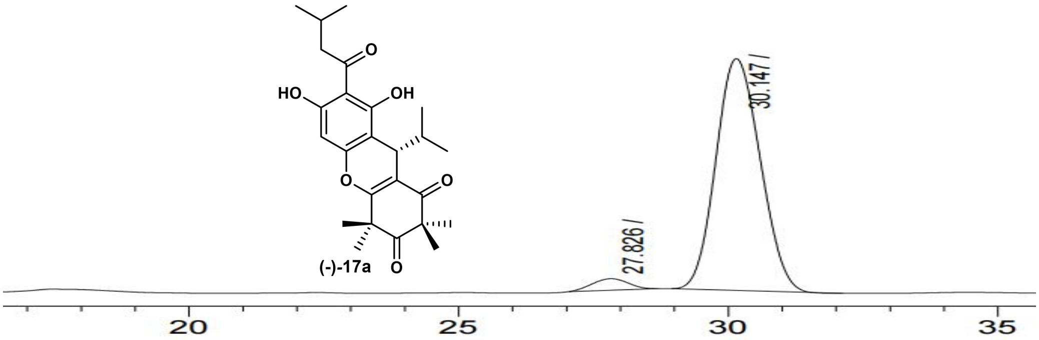

Peak#	Ret. Time	Area	Area%	Height
1	27.826	1250538	3.830	27085
2	30.147	31399238	96.170	548182
Total		32649776	100.000	575267

Supplementary Figure 30. HPLC spectra of (-)-17a.

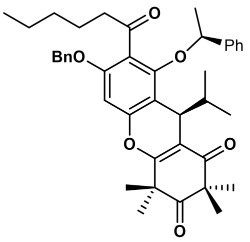

17ba

Compound **17ba**: 24.1 mg, 38% yield, 11 h, white foams (0.1 mmol scale); **R*f*** = 0.48 (ethyl acetate/*n*-hexane = 1/10); $[\alpha]_D^{26}$ = +19.3 (*c* = 0.15 in CH_2Cl_2); **IR** (KBr): 3063, 3033, 2962, 2929, 2858, 1713, 1649, 1609, 1459, 1378, 1262, 1103, 802 cm^{-1}; **^{1}H NMR** (400 MHz, $CDCl_3$) δ 7.36 (m, 7H), 7.20 (m, 3H), 6.47 (s, 1H), 5.05 (m, 3H), 3.82 (d, *J* = 3.5 Hz, 1H), 2.91 (m, 1H), 2.77 (m, 1H), 1.75 (m, 1H), 1.60 (d, *J* = 6.4 Hz, 3H), 1.51 (s, 3H), 1.35 (s, 6H), 1.33 (m, 6H), 1.30 (s, 3H), 0.86 (t, *J* = 6.5 Hz, 3H), 0.69 (d, *J* = 6.8 Hz, 3H), 0.54 (d, *J* = 6.8 Hz, 3H); **^{13}C NMR** (101 MHz,

$CDCl_3$) δ 212.6, 205.2, 196.9, 167.8, 154.6, 153.3 (2 carbons overlapped), 140.7, 136.2, 128.7 (2 carbons overlapped), 128.4 (2 carbons overlapped), 128.2, 128.2, 127.3 (2 carbons overlapped), 127.3 (2 carbons overlapped), 123.6, 111.5, 111.4, 96.5, 83.9, 70.9, 56.0, 47.3, 45.2, 35.4, 33.1, 31.5, 25.2, 24.7, 24.6, 24.5, 23.6, 22.6, 21.9, 19.2, 18.9, 14.1; **HRMS** (ESI-TOF) m/z: $[M+Na]^+$ calcd. for $C_{41}H_{48}O_6Na$ 659.3343; found, 659.3338.

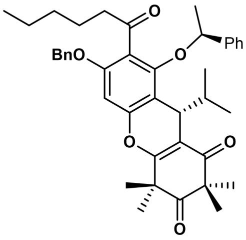

17bb

Compound **17bb**: 22.3 mg, 35% yield, 11 h, white foams (0.1 mmol scale); $\mathbf{R}_f$ = 0.40 (ethyl acetate/*n*-hexane = 1/10); $[\alpha]_D^{26}$ = −18.4 (*c* = 0.25 in CH_2Cl_2); **IR** (KBr): 3066, 3033, 2959, 2929, 2855, 1713, 1646, 1581, 1459, 1383, 1161, 1031, 804 cm^{-1}; **^{1}H NMR** (400 MHz, $CDCl_3$) δ 7.37 (m, 10H), 6.54 (s, 1H), 5.03 (m, 3H), 4.45 (d, *J* = 3.9 Hz, 1H), 2.62 (m, 1H), 2.31 (m, 1H), 1.94 (m, 1H), 1.61 (s, 3H), 1.52 (d, *J* = 6.4 Hz, 3H), 1.46 (s, 3H), 1.44 (s, 3H), 1.38 (s, 3H), 1.32 (m, 2H), 1.07 (m, 4H), 0.79 (t, *J* = 5.8 Hz, 3H), 0.77 (d, *J* = 6.9 Hz, 3H), 0.75 (d, *J* = 7.0 Hz, 3H); **^{13}C NMR** (101 MHz, $CDCl_3$) δ 212.5, 204.8, 197.9, 168.3, 154.9, 153.3 (2 carbons overlapped), 142.4, 136.2, 128.7 (2 carbons overlapped), 128.6 (2 carbons overlapped), 128.2, 128.0, 127.3 (2 carbons overlapped), 126.4 (2 carbons overlapped), 123.5, 112.2, 111.4, 96.5, 84.4, 70.9, 56.2, 47.5, 44.8, 35.5, 33.4, 31.2, 25.6, 25.4, 24.7, 23.8, 23.2, 22.8, 22.5, 19.5, 18.8, 14.0; **HRMS** (ESI-TOF) m/z: $[M+Na]^+$ calcd. for $C_{41}H_{48}O_6Na$ 659.3343; found, 659.3341.

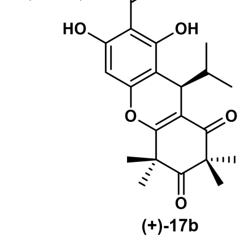

(+)-17b

Compound (+)-**17b**: 13.2 mg, 95% yield, 2 h, yellow solids (20 mg scale); $[\alpha]_D^{26}$ = +89.0 (*c* = 0.1

in MeOH); Compound (-)-**17b**: 13.3 mg, 96% yield, 2 h, yellow solids (20 mg scale); $[\alpha]_D^{26}$ = −81.9 (c = 0.1 in MeOH); **R$_f$** = 0.33 (ethyl acetate/n-hexane = 1/5); **IR** (KBr): 3253, 2957, 2929, 2870, 1718, 1631, 1424, 1385, 1269, 1163, 1103, 823 cm^{-1}; **^{1}H NMR** (400 MHz, $CDCl_3$) δ 12.80 (s, 1H), 8.57 (s, 1H), 6.18 (s, 1H), 4.37 (d, J = 3.4 Hz, 1H), 3.12 (m, 2H), 1.97 (m, 1H), 1.70 (m, 2H), 1.58 (s, 3H), 1.44 (s, 3H), 1.42 (s, 3H), 1.38 (s, 3H), 1.36 (m, 4H), 0.91 (t, J = 0.90 Hz, 3H), 0.79 (d, J = 6.8 Hz, 3H), 0.77 (d, J = 7.0 Hz, 3H); **^{13}C NMR** (101 MHz, $CDCl_3$) δ 212.3, 207.3, 198.6, 168.6, 162.3, 159.4, 156.6, 112.0, 107.8, 104.6, 94.9, 56.2, 47.6, 44.7, 34.7, 31.9, 31.8, 25.3 (2 carbons overlapped), 24.7, 24.4, 24.1, 22.7, 19.5, 18.9, 14.1; **HRMS** (ESI-TOF) m/z: $[M+H]^+$ calcd. for $C_{26}H_{35}O_6$ 443.2428; found, 443.2423.

HPLC condition of (+)-17b: Phenomenex Lux Cellulose-3 column; acetonitrile/H_2O= 45:55, 1.0 mL/min, λ = 254 nm; major enantiomer: t_R = 74.0 min, minor enantiomer: t_R = 79.4 min, 96% *ee*.

HPLC condition of (-)-17b: Phenomenex Lux Cellulose-3 column; acetonitrile/H_2O= 45:55, 1.0 mL/min, λ = 254 nm; minor enantiomer: t_R = 74.8 min, major enantiomer: t_R = 79.3 min, 93.5% *ee*.

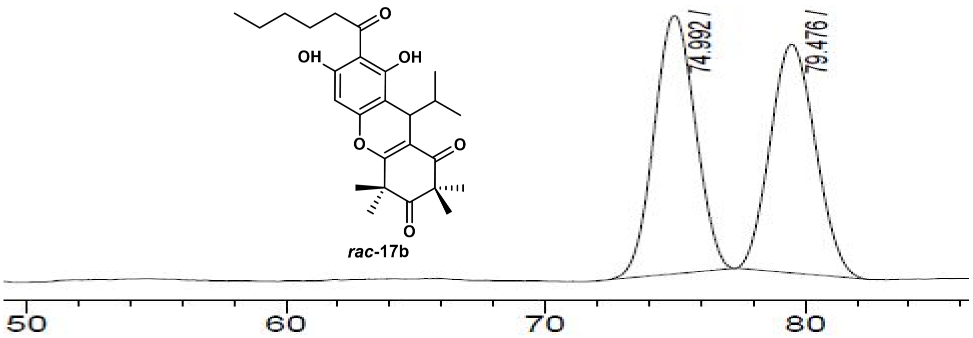

Peak#	Ret. Time	Area	Area%	Height
1	74.992	6826331	50.663	61657
2	79.476	6647670	49.337	54548
Total		13474001	100.000	116205

Supplementary Figure 31. HPLC spectra of *rac*-17b.

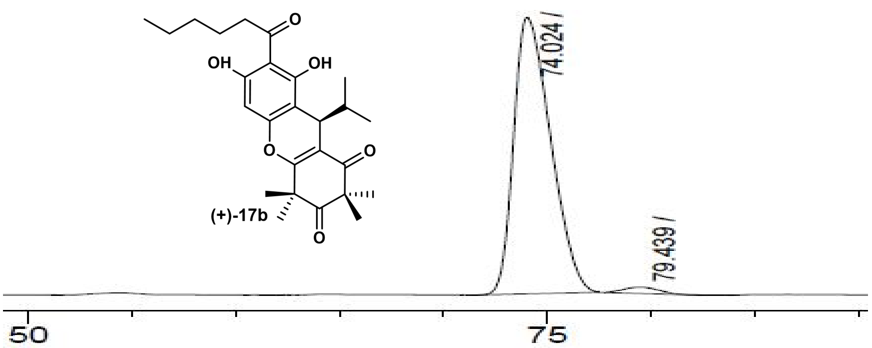

Peak#	Ret. Time	Area	Area%	Height
1	74.024	45348957	97.922	365116
2	79.439	962177	2.078	8210
Total		46311134	100.000	373325

Supplementary Figure 32. HPLC spectra of (+)-17b.

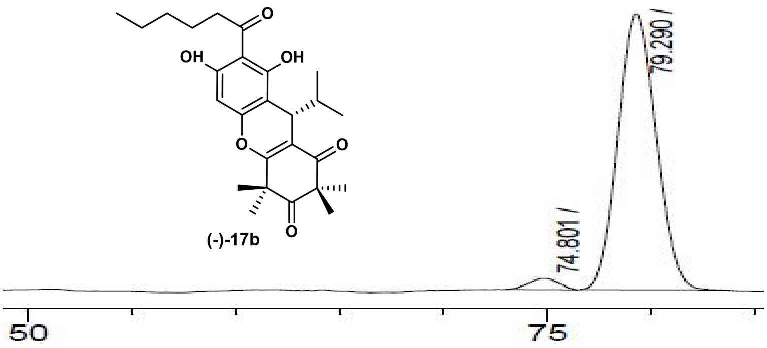

Peak#	Ret. Time	Area	Area%	Height
1	74.801	306571	3.233	2965
2	79.290	9176315	96.767	71172
Total		9482886	100.000	74137

Supplementary Figure 33. HPLC spectra of (-)-17b.

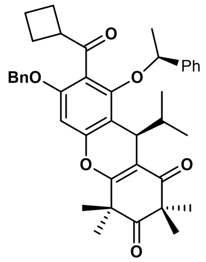

17ca

Compound **17ca**: 24.2 mg, 39% yield, 11 h, white foams (0.1 mmol scale); $\mathbf{R}_f$ = 0.29 (ethyl acetate/*n*-hexane = 1/12); $[\alpha]_D^{26}$ = +29.1 (*c* = 0.12 in CH_2Cl_2); **IR** (KBr): 3066, 3029, 2962, 2932, 2871, 1715, 1649, 1581, 1457, 1378, 1260, 1022, 800 cm^{-1}; **^{1}H NMR** (400 MHz, $CDCl_3$) δ 7.35 (m, 7H), 7.18 (m, 3H), 6.46 (s, 1H), 5.00 (m, 3H), 3.77 (m, 2H), 2.49 (m, 1H), 2.31 (m, 1H), 2.11

(m, 2H), 1.95 (m, 1H), 1.83 (m, 1H), 1.72 (m, 1H), 1.59 (d, *J* = 6.5 Hz, 3H), 1.50 (s, 3H), 1.35 (s, 3H), 1.35 (s, 3H), 0.69 (d, *J* = 6.9 Hz, 3H), 0.53 (d, *J* = 6.9 Hz, 3H); **^{13}C NMR** (101 MHz, $CDCl_3$) δ 212.6, 206.2, 196.8, 167.7, 154.8, 153.6, 153.6, 140.7, 136.3, 128.7 (2 carbons overlapped), 128.4 (2 carbons overlapped), 128.2, 128.2, 127.3 (2 carbons overlapped), 127.3 (2 carbons overlapped), 122.5, 111.5, 111.4, 96.4, 84.0, 70.8, 55.9, 47.4, 47.3, 35.4, 33.1, 25.3, 25.2, 24.8, 24.6, 24.4, 24.4, 21.8, 19.3, 18.8, 17.9; **HRMS** (ESI-TOF) m/z: $[M+Na]^+$ calcd. for $C_{40}H_{44}O_6Na$ 643.3030; found, 643.3029.

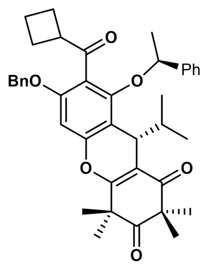

17cb

Compound **17cb**: 22.3 mg, 36% yield, 11 h, white foams (0.1 mmol scale); **R_f** = 0.23 (ethyl acetate/*n*-hexane = 1/12); $[\alpha]_D^{26}$ = −15.0 (*c* = 0.2 in CH_2Cl_2); **IR** (KBr): 3063, 3033, 2962, 2932, 2863, 1718, 1644, 1581, 1457, 1383, 1257, 1023, 797 cm^{-1}; **^{1}H NMR** (400 MHz, $CDCl_3$) δ 7.33 (m, 10H), 6.54 (s, 1H), 5.03 (m, 3H), 4.45 (d, *J* = 3.9 Hz, 1H), 3.53 (m, 1H), 225 (m, 1H), 2.06 (m, 1H), 1.93 (m, 2H), 1.74 (m, 3H), 1.60 (s, 3H), 1.46 (d, *J* = 6.7 Hz, 3H), 1.46 (s, 3H), 1.44 (s, 3H), 1.38 (s, 3H), 0.80 (d, *J* = 6.8 Hz, 3H), 0.75 (d, *J* = 6.9 Hz, 3H); **^{13}C NMR** (101 MHz, $CDCl_3$) δ 212.5, 205.8, 197.8, 168.3, 155.0, 153.5, 153.3, 142.3, 136.3, 128.7 (2 carbons overlapped), 128.5 (2 carbons overlapped), 128.1, 128.0, 127.2 (2 carbons overlapped), 126.3 (2 carbons overlapped), 122.1, 112.2, 111.6, 96.5, 84.3, 70.8, 56.2, 47.5, 47.1, 35.6, 33.3, 25.4, 25.4, 25.2, 24.7, 23.9, 23.7, 22.4, 19.3, 19.1, 17.7; **HRMS** (ESI-TOF) m/z: $[M+Na]^+$ calcd. for $C_{40}H_{44}O_6Na$ 643.3030; found, 643.3020.

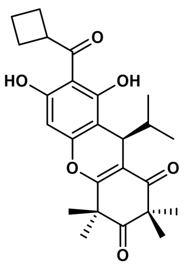

(+)-17c

Compound (+)**-17c**: 13.2 mg, 96% yield, 2 h, yellow solids (20 mg scale); $[\alpha]_D^{26}$ = +92.7 (*c* = 0.1 in MeOH); Compound (-)**-17c**: 12.9 mg, 94% yield, 2 h, yellow solids; $[\alpha]_D^{26}$ = −87.8 (*c* = 0.1 in MeOH); R_f = 0.31 (ethyl acetate/*n*-hexane = 1/5); **IR** (KBr): 3285, 2959, 2929, 2853, 1718, 1631, 1586, 1429, 1362, 1163, 1105, 823 cm^{-1}; **^{1}H NMR** (400 MHz, $CDCl_3$) δ 12.80 (s, 1H), 8.52 (s, 1H), 6.18 (s, 1H), 4.36 (d, *J* = 3.4 Hz, 1H), 4.34 – 4.13 (m, 1H), 2.32 (m, 4H), 2.01 (m, 3H), 1.59 (s, 3H), 1.45 (s, 3H), 1.42 (s, 3H), 1.39 (s, 3H), 0.79 (d, *J* = 7.1 Hz, 3H), 0.77 (d, *J* = 7.2 Hz, 3H); **^{13}C NMR** (101 MHz, $CDCl_3$) δ 212.4, 207.2, 198.8, 168.7, 162.5, 159.3, 156.6, 112.0, 106.8, 104.5, 94.8, 56.2, 47.6, 46.8, 34.7, 31.9, 25.3, 25.3, 25.1, 24.8, 24.7, 24.2, 19.5, 18.9, 17.8; **HRMS** (ESI-TOF) m/z: $[M+H]^+$ calcd. for $C_{25}H_{31}O_6$ 427.2115; found, 427.2119.

HPLC condition of (+)-17c: Phenomenex Lux Cellulose-4 column; acetonitrile/H_2O = 45:55, 1.0 mL/min, λ = 254 nm; major enantiomer: t_R = 77.2 min, >99% *ee*.

HPLC condition of (-)-17c: Phenomenex Lux Cellulose-4 column; acetonitrile/H_2O= 45:55, 1.0 mL/min, λ = 254 nm; minor enantiomer: t_R = 77.7 min, major enantiomer: t_R = 87.8 min, 96.5% *ee*.

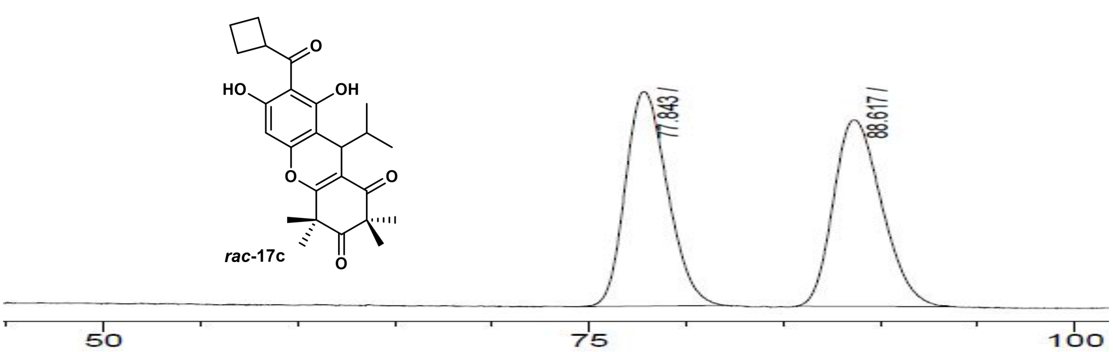

Peak#	Ret. Time	Area	Area%	Height
1	77.843	5072144	50.103	33576
2	88.617	5051268	49.897	29184
Total		10123412	100.000	62760

Supplementary Figure 34. HPLC spectra of *rac*-17c.

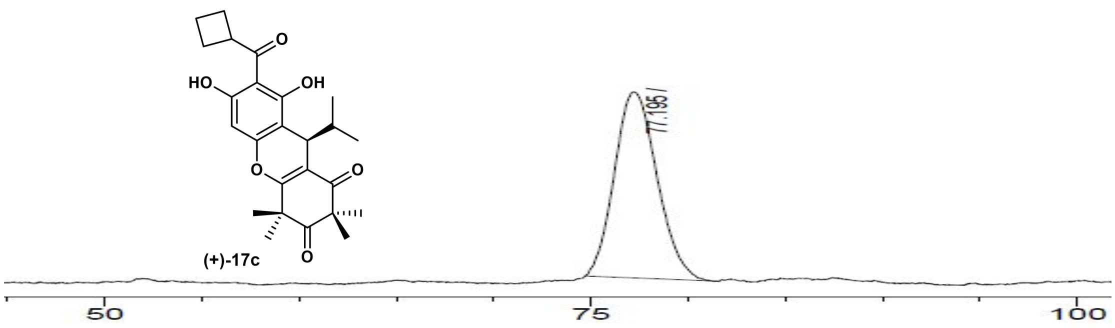

Peak#	Ret. Time	Area	Area%	Height
1	77.195	1544001	100.000	10132
Total		1544001	100.000	10132

Supplementary Figure 35. HPLC spectra of (+)-17c.

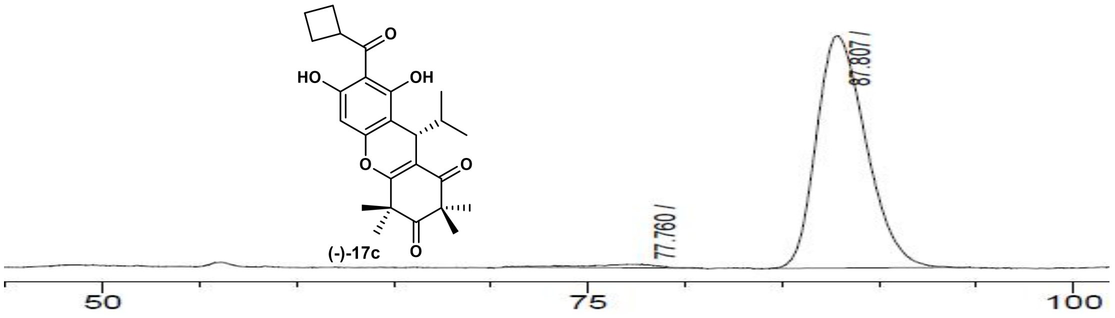

Peak#	Ret. Time	Area	Area%	Height	Height%
1	77.760	50696	0.916	381	1.227
2	87.807	5481842	99.084	30636	98.773
Total		5532539	100.000	31016	100.000

Supplementary Figure 36. HPLC spectra of (+)-17c.

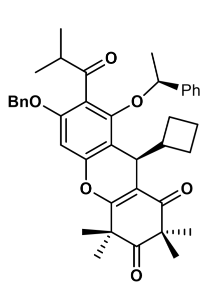

17da

Compound **17da**: 23.5 mg, 38% yield, 11 h, white foams (0.1 mmol scale); $\mathbf{R}_f$ = 0.43 (ethyl acetate/*n*-hexane = 1/10); $[\alpha]_D^{26}$ = +62.3 (*c* = 0.13 in CH_2Cl_2); **IR** (KBr): 3063, 3032, 2969, 2934, 2870, 1702, 1642, 1586, 1466, 1383, 1181, 1029, 812 cm^{-1}; **^{1}H NMR** (400 MHz, $CDCl_3$) δ 7.36 (m, 7H), 7.18 (m, 3H), 6.49 (s, 1H), 5.06 (m, 3H), 3.74 (d, *J* = 6.6 Hz, 1H), 3.13 (m, 1H), 2.29 (m, 1H), 1.59 (d, *J* = 6.4 Hz, 3H), 1.58 (m, 2H), 1.50 (d, *J* = 17.9 Hz, 3H), 1.48 (m, 3H), 1.41 (m, 1H),

1.36 (s, 3H), 1.33 (s, 3H), 1.29 (s, 3H), 1.23 (d, J = 6.9 Hz, 3H), 1.14 (d, J = 7.1 Hz, 3H); **^{13}C NMR** (101 MHz, $CDCl_3$) δ 212.6, 208.8, 196.8, 167.4, 154.8, 153.2, 152.7, 140.5, 136.3, 128.7 (2 carbons overlapped), 128.3 (2 carbons overlapped), 128.2, 128.2, 127.4 (2 carbons overlapped), 127.3 (2 carbons overlapped), 123.0, 111.4 (2 carbons overlapped), 96.7, 83.9, 70.8, 56.0, 47.2, 42.7, 42.7, 31.2, 25.5, 25.4, 25.0, 25.0, 24.5, 24.2, 21.7, 18.2, 17.8, 17.7; **HRMS** (ESI-TOF) m/z: $[M+Na]^+$ calcd. for $C_{40}H_{44}O_6Na$ 643.3030; found, 643.3025.

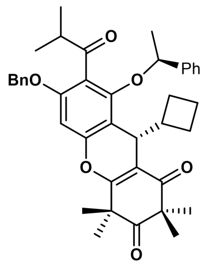

17db

Compound **17db**: 21.1 mg, 34% yield, 11 h, white foams (0.1 mmol scale); **R*f*** = 0.33 (ethyl acetate/*n*-hexane = 1/10); $[\alpha]_D^{26}$ = −45.2 (c = 0.15 in CH_2Cl_2); **IR** (KBr): 3061, 3033, 2969, 2929, 2867, 1709, 1649, 1579, 1461, 1381, 1179, 1029, 804 cm^{-1}; **^{1}H NMR** (400 MHz, $CDCl_3$) δ 7.34 (m, 10H), 6.57 (s, 1H), 5.06 (m, 3H), 4.43 (d, J = 6.5 Hz, 1H), 2.89 (m, 1H), 2.53 (m, 1H), 1.71 (m, 8H), 1.58 (s, 3H), 1.53 (m, 1H), 1.46 (s, 3H), 1.42 (s, 3H), 1.38 (s, 3H), 0.97 (d, J = 7.1 Hz, 3H), 0.88 (d, J = 6.8 Hz, 3H); **^{13}C NMR** (101 MHz, $CDCl_3$) δ 212.5, 208.5, 197.7, 167.9, 155.1, 153.1, 152.7, 142.6, 136.3, 128.7 (2 carbons overlapped), 128.5 (2 carbons overlapped), 128.2, 127.9, 127.2 (2 carbons overlapped), 126.2 (2 carbons overlapped), 122.6, 111.7, 111.5, 96.7, 84.2, 70.8, 56.3, 47.4, 42.7, 42.3, 31.4, 25.6 (2 carbons overlapped), 25.2, 25.1, 24.5, 24.1, 22.7, 18.1, 17.9, 17.1; **HRMS** (ESI-TOF) m/z: $[M+Na]^+$ calcd. for $C_{40}H_{44}O_6Na$ 643.3030; found, 643.3033.

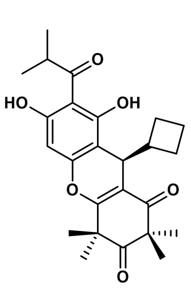

(+)-17d

Compound (+)-**17d**: 12.6 mg, 92% yield, 2 h, yellow solids (20 mg scale); $[\alpha]_D^{26}$ = +137.8 (c = 0.1

in MeOH); Compound (-)-**17d**: 13.1 mg, 95% yield, 2 h, yellow solids (20 mg scale); $[\alpha]_D^{26}$ = −142.5 (c = 0.1 in MeOH); **R$_f$** = 0.29 (ethyl acetate/n-hexane = 1/5); **IR** (KBr): 3323, 2982, 2939, 2870, 1718, 1631, 1588, 1427, 1360, 1262, 1166, 1098, 819 cm^{-1}; **^{1}H NMR** (400 MHz, $CDCl_3$) δ 12.04 (s, 1H), 9.44 (s, 1H), 6.23 (s, 1H), 4.40 (d, J = 5.6 Hz, 1H), 3.92 (m, 1H), 2.69 (m, 1H), 1.65 (m, 6H), 1.57 (s, 3H), 1.45 (s, 3H), 1.44 (s, 3H), 1.39 (s, 3H), 1.22 (s, 3H), 1.20 (s, 3H); **^{13}C NMR** (101 MHz, $CDCl_3$) δ 212.2, 211.5, 198.9, 168.7, 161.7, 160.2, 156.1, 111.8, 107.2, 103.3, 100.1, 95.6, 56.2, 47.5, 41.5, 39.9, 29.5, 25.6, 25.2, 24.7, 24.7, 24.6, 19.5, 19.2, 17.9; **HRMS** (ESI-TOF) m/z: $[M+H]^+$ calcd. for $C_{25}H_{31}O_6$ 427.2115; found, 427.2121.

HPLC condition of (+)-17d: Phenomenex Lux Cellulose-3 column; acetonitrile/H_2O = 45:55, 1.0 mL/min, λ = 254 nm; major enantiomer: t_R = 19.5 min, minor enantiomer: t_R = 21.4 min, 97% *ee*.

HPLC condition of (-)-17d: Phenomenex Lux Cellulose-3 column; acetonitrile/H_2O= 45:55, 1.0 mL/min, λ = 254 nm; minor enantiomer: t_R = 19.7 min, major enantiomer: t_R = 21.5 min, 98% *ee*.

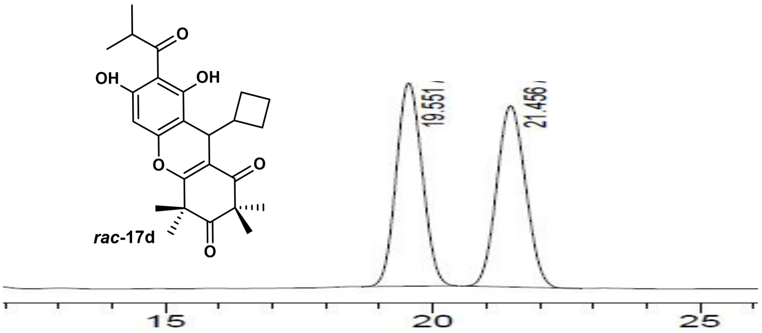

Peak#	Ret. Time	Area	Area%	Height
1	19.551	24436598	50.247	720947
2	21.456	24196509	49.753	642789
Total		48633107	100.000	1363736

Supplementary Figure 37. HPLC spectra of *rac*-17d.

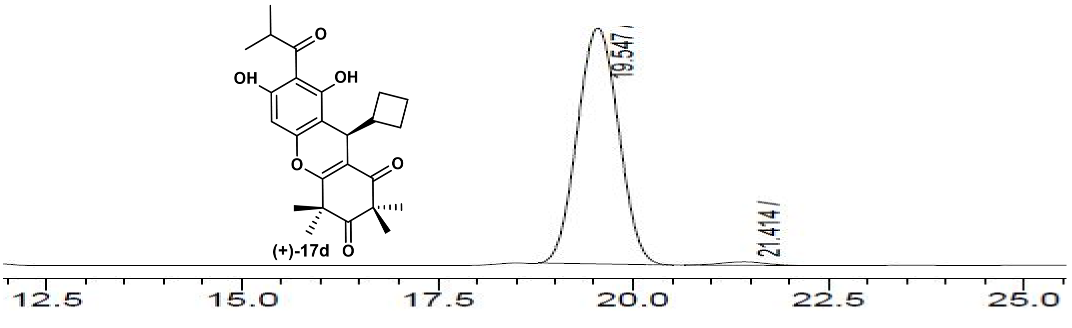

Peak#	Ret. Time	Area	Area%	Height
1	19.547	10290052	98.463	279425
2	21.414	160590	1.537	4012
Total		10450642	100.000	283437

Supplementary Figure 38. HPLC spectra of (+)-17d.

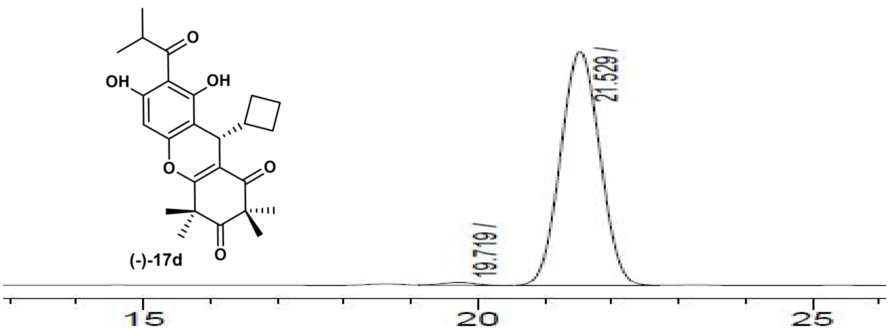

Peak#	Ret. Time	Area	Area%	Height
1	19.719	119096	1.008	3441
2	21.529	11697099	98.992	286002
Total		11816195	100.000	289443

Supplementary Figure 39. HPLC spectra of (-)-17d.

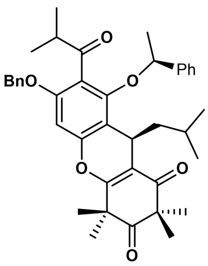

17ea

Compound **17ea**: 23.6 mg, 38% yield, 11 h, white foams (0.1 mmol scale); $\mathbf{R}_f$ = 0.39 (ethyl acetate/*n*-hexane = 1/10); $[\alpha]_D^{26}$ = +19.2 (*c* = 0.1 in CH_2Cl_2); **IR** (KBr): 3066, 3035, 2971, 2932, 2870, 1713, 1651, 1604, 1459, 1378, 1257, 1031, 804 cm^{-1}; **^{1}H NMR** (400 MHz, $CDCl_3$) δ 7.34 (m, 7H), 7.16 (m, 3H), 6.48 (s, 1H), 5.05 (m, 3H), 3.67 (t, *J* = 6.3 Hz, 1H), 3.13 (m, 1H), 1.62 (d, *J* = 6.5 Hz, 3H), 1.47 (s, 3H), 1.36 (s, 3H), 1.43 – 1.24 (m, 3H), 1.33 (s, 3H), 1.29 (s, 3H), 1.23 (d, *J* = 6.9 Hz, 3H), 1.13 (d, *J* = 7.1 Hz, 3H), 0.82 (d, *J* = 6.4 Hz, 3H), 0.76 (d, *J* = 6.6 Hz, 3H); **^{13}C NMR** (101 MHz, $CDCl_3$) δ 212.6, 208.9, 196.5, 167.2, 154.8, 152.9, 152.5, 140.2, 136.3, 128.7 (2 carbons overlapped), 128.3 (2 carbons overlapped), 128.3, 128.2, 127.5 (2 carbons overlapped), 127.3 (2 carbons overlapped), 122.8, 114.0, 113.7, 96.9, 84.0, 70.8, 55.9, 47.3, 47.1, 42.7, 26.2, 25.3, 24.9, 24.8, 24.6, 23.8, 23.5, 23.0, 21.4, 18.3, 17.7; **HRMS** (ESI-TOF) m/z: $[M+Na]^+$ calcd. for $C_{41}H_{48}O_6Na$ 645.3187; found, 645.3180.

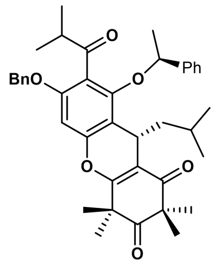

17eb

Compound **17eb**: 21.8 mg, 35% yield, 11 h, white foams (0.1 mmol scale); **R*f*** = 0.30 (ethyl acetate/*n*-hexane = 1/10); **[α]$_D^{26}$** = −27.8 (*c* = 0.1 in CH_2Cl_2); **IR** (KBr): 3068, 3038, 2969, 2929, 2870, 1711, 1665, 1604, 1459, 1382, 1260, 1031, 800 cm^{-1}; **^{1}H NMR** (400 MHz, $CDCl_3$) δ 7.35 (m, 10H), 6.56 (s, 1H), 5.06 (m, 3H), 4.46 (t, *J* = 5.5 Hz, 1H), 2.87 (m, 1H), 1.58 (s, 3H), 1.47 (s, 6H), 1.42 (s, 3H), 1.40 (m, 3H), 1.39 (s, 3H), 0.96 (d, *J* = 7.1 Hz, 3H), 0.92 (d, *J* = 5.9 Hz, 3H), 0.87 (d, *J* = 5.7 Hz, 3H), 0.85 (d, *J* = 4.6 Hz, 3H); **^{13}C NMR** (101 MHz, $CDCl_3$) δ 212.4, 208.5, 197.6, 167.7, 155.0, 153.0, 152.5, 142.4, 136.3, 128.7 (2 carbons overlapped), 128.5 (2 carbons overlapped), 128.1, 127.9, 127.1 (2 carbons overlapped), 126.3 (2 carbons overlapped), 122.7, 114.2, 113.8, 96.9, 84.4, 70.8, 56.2, 47.4, 47.1, 42.4, 26.6, 25.1, 25.0 (2 carbons overlapped), 24.7, 24.1, 23.6, 23.5, 22.5, 18.1, 17.0; **HRMS** (ESI-TOF) m/z: [M+Na]$^+$ calcd. for $C_{41}H_{48}O_6Na$ 645.3187; found, 645.3192.

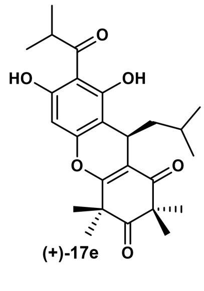

(+)-17e

Compound (+)-**17e**: 13.1 mg, 95% yield, 2 h, yellow solids (20 mg scale); **[*α*]$_D^{26}$** = +137.1 (*c* = 0.1 in MeOH); Compound (-)-**17e**: 12.5 mg, 91% yield, 2 h, yellow solids (20 mg scale); **[*α*]$_D^{26}$** = −125.9 (*c* = 0.1 in MeOH); **R*f*** = 0.33 (ethyl acetate/*n*-hexane = 1/5); **IR** (KBr): 3271, 2979, 2934, 2874, 1722, 1626, 1424, 1385, 1174, 1096, 825 cm^{-1}; **^{1}H NMR** (400 MHz, $CDCl_3$) δ 13.24 (s, 1H), 7.90 (s, 1H), 6.15 (s, 1H), 4.28 (d, *J* = 6.0 Hz, 1H), 3.92 (m, 1H), 1.55 (s, 3H), 1.44 (s, 3H), 1.42 (s, 3H), 1.40 (m, 3H),1.38 (s, 3H), 1.21 (d, *J* = 6.6 Hz, 3H), 1.17 (d, *J* = 6.7 Hz, 3H), 0.86 (d, *J* = 5.9 Hz, 3H), 0.82 (d, *J* = 6.1 Hz, 3H); **^{13}C NMR** (101 MHz, $CDCl_3$) δ 212.3, 211.4, 198.4, 167.6,

163.1, 158.5, 155.7, 114.4, 106.9, 106.7, 95.0, 56.2, 47.4, 46.0, 39.9, 25.4, 25.3, 24.9, 24.8, 24.7, 24.4, 23.7, 23.3, 19.4, 19.2; **HRMS** (ESI-TOF) m/z: $[M+H]^+$ calcd. for $C_{25}H_{33}O_6$ 429.2272; found, 429.2274.

HPLC condition of (+)-17e: Phenomenex Lux Cellulose-3 column; acetonitrile/H_2O = 40:60, 1.0 mL/min, λ = 254 nm; major enantiomer: t_R = 95.6 min, >99% *ee*.

HPLC condition of (-)-17e: Phenomenex Lux Cellulose-3 column; acetonitrile/H_2O= 40:60, 1.0 mL/min, λ = 254 nm; minor enantiomer: t_R = 95.9 min, major enantiomer: t_R = 101.5 min, 95.5% *ee*.

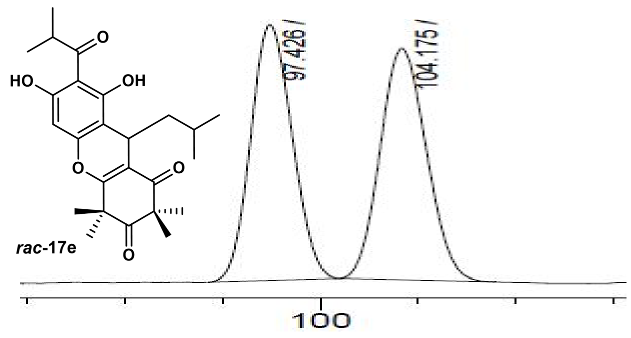

Peak#	Ret. Time	Area	Area%	Height
1	97.426	10218621	49.843	68945
2	104.175	10282791	50.157	62348
Total		20501412	100.000	131292

Supplementary Figure 40. HPLC spectra of *rac*-17e.

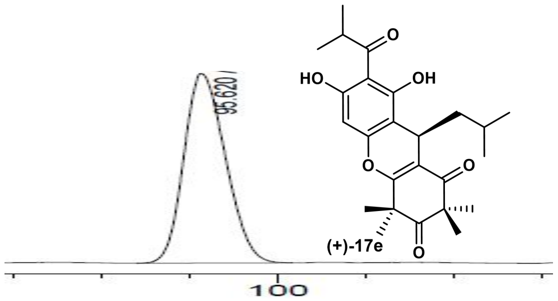

Peak#	Ret. Time	Area	Area%	Height
1	95.620	22313012	100.000	135400
Total		22313012	100.000	135400

Supplementary Figure 41. HPLC spectra of *rac*-17e.

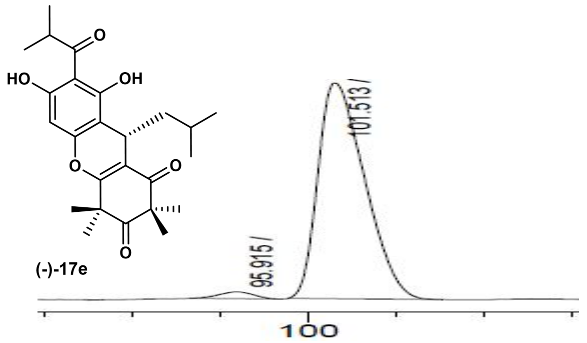

Peak#	Ret. Time	Area	Area%	Height
1	95.915	1192253	2.257	8608
2	101.513	51627369	97.743	278315
Total		52819623	100.000	286923

Supplementary Figure 42. HPLC spectra of (-)-17e.

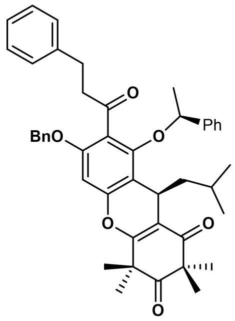

17fa

Compound **17fa**: 26.7 mg, 39% yield, 11 h, white foams (0.1 mmol scale); $\mathbf{R}_f$ = 0.43 (ethyl acetate/*n*-hexane = 1/10); $[\alpha]_D^{26}$ = +25.2 (c = 0.1 in CH_2Cl_2); **IR** (KBr): 3063, 3029, 2959, 2927, 2851, 1715, 1656, 1604, 1459, 1381, 1262, 1087, 804 cm^{-1}; ^{1}**H NMR** (400 MHz, $CDCl_3$) δ 7.37 (m, 4H), 7.25 (m, 5H), 7.15 (m, 6H), 6.47 (s, 1H), 5.01 (m, 1H), 3.73 (t, J = 6.4 Hz, 1H), 3.27 (m, 1H), 3.06 (m, 3H), 1.58 (d, J = 6.5 Hz, 3H), 1.48 (s, 3H), 1.36 (s, 3H), 1.34 (s, 3H), 1.29 (d, J = 2.7 Hz, 3H), 0.82 (d, J = 6.5 Hz, 3H), 0.78 (d, J = 6.5 Hz, 3H); ^{13}**C NMR** (101 MHz, $CDCl_3$) δ 212.5, 203.8, 196.6, 167.2, 154.8, 153.1, 152.8, 141.3, 140.2, 136.2, 128.8 (2 carbons overlapped), 128.6 (2 carbons overlapped), 128.6 (2 carbons overlapped), 128.4 (2 carbons overlapped), 128.3, 127.4 (2 carbons overlapped), 127.4 (2 carbons overlapped), 126.1, 126.1, 122.9, 114.2, 113.8, 96.9, 84.0, 71.0, 55.9, 47.5, 47.2, 46.7, 30.0, 26.2, 25.2, 24.9, 24.9, 24.6, 24.0, 23.3, 23.1, 21.6; **HRMS** (ESI-TOF) m/z: $[M+Na]^+$ calcd. for $C_{45}H_{48}O_6Na$ 707.3343; found,707.3334.

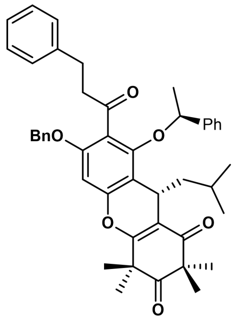

17fb

Compound **17fb**: 23.9 mg, 35% yield, 11 h, white foams (0.1 mmol scale); $\mathbf{R}_f$ = 0.36 (ethyl acetate/*n*-hexane = 1/10); $[\alpha]_D^{26}$ = −31.0 (*c* = 0.1 in CH_2Cl_2); **IR** (KBr): 3066, 3023, 2959, 2925, 2851, 1718, 1651, 1584, 1464, 1381, 1265, 1089, 800 cm^{-1}; **^{1}H NMR** (400 MHz, $CDCl_3$) δ 7.36 (m, 10H), 7.16 (m, 3H), 6.91 (m, 2H), 6.56 (s, 1H), 5.05 (m, 3H), 4.47 (t, *J* = 6.0 Hz, 1H), 2.97 (m, 1H), 2.75 (m, 1H), 2.58 (m, 2H), 1.58 (d, *J* = 6.3 Hz, 3H), 1.52 (m, 3H), 1.49 (s, 3H), 1.44 (s, 3H), 1.41 (s, 3H), 1.36 (s, 3H), 1.31 (s, 3H), 0.96 (d, *J* = 6.1 Hz, 3H), 0.86 (d, *J* = 6.1 Hz, 3H); **^{13}C NMR** (101 MHz, $CDCl_3$) δ 212.4, 203.5, 197.7, 167.7, 154.9, 153.4, 152.8, 142.3, 141.2, 136.1, 128.8 (2 carbons overlapped), 128.7 (2 carbons overlapped), 128.4 (2 carbons overlapped), 128.4 (2 carbons overlapped), 128.3, 128.2, 127.3 (2 carbons overlapped), 126.4 (2 carbons overlapped), 125.9, 123.0, 114.2, 113.9, 97.0, 84.8, 71.0, 68.3, 56.2, 47.4, 47.4, 46.4, 30.5, 26.5, 25.2, 25.1, 25.1, 24.7, 24.0, 23.5, 23.4, 22.9; **HRMS** (ESI-TOF) m/z: $[M+Na]^+$ calcd. for $C_{45}H_{48}O_6Na$ 707.3343; found,707.3341.

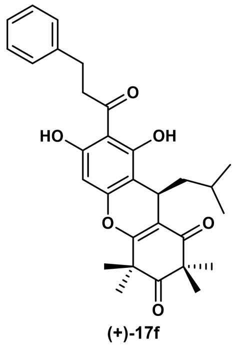

(+)-17f

Compound (+)**-17f**: 12.8 mg, 91% yield, 2 h, yellow solids (20 mg scale); $[\alpha]_D^{26}$ = +110.5 (*c* = 0.1 in MeOH); Compound (-)**-17f**: 13.1 mg, 93% yield, 2 h, yellow solids (20 mg scale); $[\alpha]_D^{26}$ = −104.3 (*c* = 0.1 in MeOH); $\mathbf{R}_f$ = 0.28 (ethyl acetate/*n*-hexane = 1/5); **IR** (KBr): 3343, 2976, 2946, 2871, 1715, 1646, 1591, 1436, 1385, 1170, 1085, 823 cm^{-1}; **^{1}H NMR** (400 MHz, $CDCl_3$) δ 13.15

(s, 1H), 7.95 (s, 1H), 7.25 (m, 5H), 6.16 (s, 1H), 4.30 (d, J = 5.3 Hz, 1H), 3.48 (t, J = 7.7 Hz, 2H), 3.06 (t, J = 7.7 Hz, 2H), 1.56 (s, 3H), 1.46 (m, 3H), 1.44 (s, 3H), 1.41 (s, 3H), 1.38 (s, 3H), 0.87 (d, J = 5.8 Hz, 3H), 0.85 (d, J = 5.7 Hz, 3H); **^{13}C NMR** (101 MHz, $CDCl_3$) δ 212.2, 205.6, 198.5, 167.6, 162.8, 158.9, 155.9, 141.6, 128.6 (2 carbons overlapped), 128.6 (2 carbons overlapped), 126.1, 114.4, 107.5, 106.6, 100.1, 94.9, 56.2, 47.4, 46.2, 46.0, 30.6, 25.3, 25.3, 24.9, 24.7, 24.3, 23.7, 23.3; **HRMS** (ESI-TOF) m/z: $[M+H]^+$ calcd. for $C_{30}H_{35}O_6$ 491.2428; found, 491.2423.

HPLC condition of (+)-17f: Phenomenex Lux Cellulose-3 column; acetonitrile/H_2O = 50:50, 1.0 mL/min, λ = 254 nm; major enantiomer: t_R = 50.1 min, minor enantiomer: t_R = 62.7 min, 99% *ee*.

HPLC condition of (-)-17f: Phenomenex Lux Cellulose-3 column; acetonitrile/H_2O= 50:50, 1.0 mL/min, λ = 254 nm; minor enantiomer: t_R = 52.7 min, major enantiomer: t_R = 64.7 min, 95% *ee*.

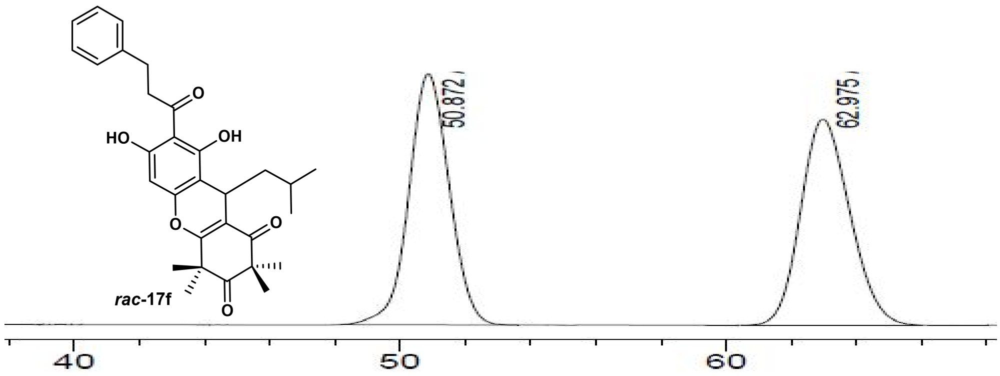

Peak#	Ret. Time	Area	Area%	Height
1	50.872	6433222	49.968	74580
2	62.975	6441520	50.032	61200
Total		12874743	100.000	135780

Supplementary Figure 43. HPLC spectra of *rac*-17f.

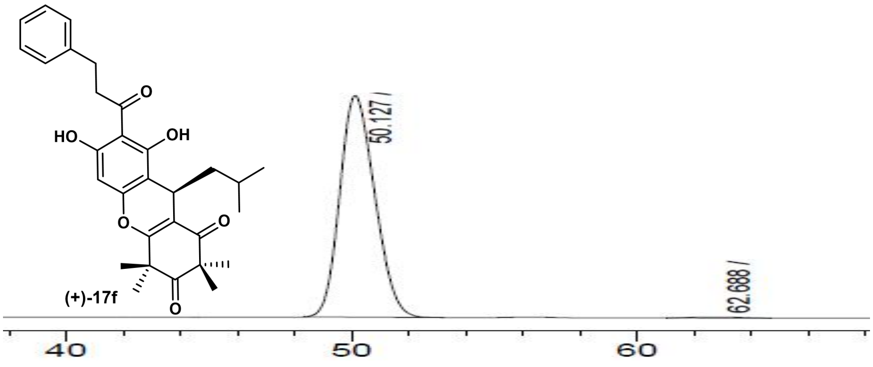

Peak#	Ret. Time	Area	Area%	Height
1	50.127	11305644	99.417	132571
2	62.688	66270	0.583	637
Total		11371914	100.000	133209

Supplementary Figure 44. HPLC spectra of (+)-17f.

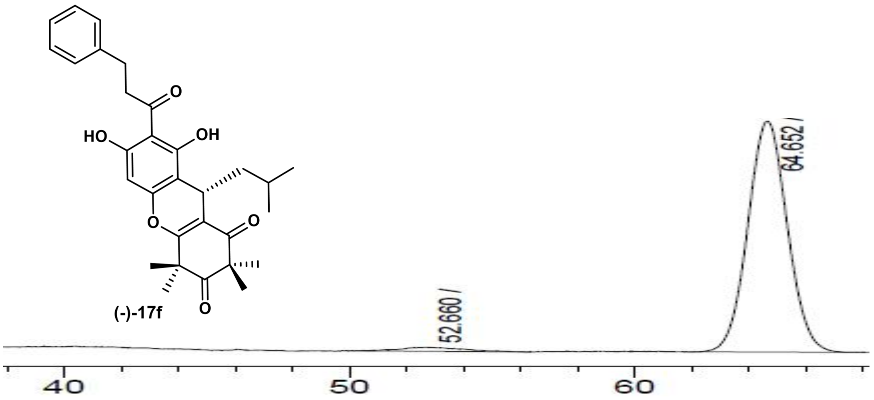

Peak#	Ret. Time	Area	Area%	Height
1	52.660	24303	2.323	169
2	64.652	1022046	97.677	10606
Total		1046349	100.000	10775

Supplementary Figure 45. HPLC spectra of (-)-17f.

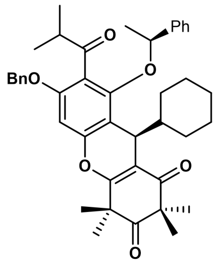

17ga

Compound **17ga**: 25.3 mg, 39% yield, 11 h, white foams (0.1 mmol scale); $\mathbf{R_f}$ = 0.39 (EtOAc/*n*-hexane = 1/10); $[\alpha]_D^{26}$ = +49.3 (*c* = 0.15 in CH_2Cl_2); **IR** (KBr): 3063, 3035, 2962, 2929, 2851, 1709, 1651, 1591, 1454, 1262, 1096, 804 cm^{-1}; ^{1}**H NMR** (400 MHz, $CDCl_3$) δ 7.35 (m, 7H), 7.19 (m, 3H), 6.47 (s, 1H), 5.04 (m, 3H), 3.76 (d, *J* = 3.7 Hz, 1H), 3.13 (m, 1H), 1.58 (d, *J* = 6.5 Hz, 3H), 1.51 (s, 3H), 1.36 (m, 6H), 1.35 (s, 3H), 1.35 (s, 3H), 1.30 (s, 3H), 1.23 (d, *J* = 6.9 Hz, 3H), 1.14 (d, *J* = 7.1 Hz, 3H), 1.00 (m, 2H), 0.85 (m, 2H), 0.51 (m, 1H); ^{13}**C NMR** (101 MHz, $CDCl_3$) δ 212.6, 208.9, 196.8, 167.7, 154.7, 153.4, 153.2, 140.7, 136.3, 128.7 (2 carbons overlapped), 128.3 (2 carbons overlapped), 128.2, 128.2, 127.3 (2 carbons overlapped), 127.3 (2 carbons overlapped), 122.9, 111.6, 111.6, 96.4, 83.9, 70.8, 56.0, 47.3, 45.7, 42.6, 32.9, 29.8, 29.2, 26.7, 26.6, 26.5, 25.2, 24.9, 24.7, 24.7, 21.9, 18.2, 17.7; **HRMS** (ESI-TOF) m/z: $[M+Na]^+$ calcd. for $C_{42}H_{48}O_6Na$ 671.3343; found, 671.3335.

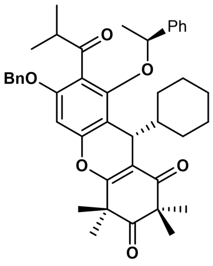

17gb

Compound **17gb**: 21.7 mg, 35% yield, 11 h, white foams (0.1 mmol scale); **R*f*** = 0.31 (ethyl acetate/*n*-hexane = 1/10); **[α]$^{26}_{D}$** = −49.3 (*c* = 0.15 in CH_2Cl_2); **IR** (KBr): 3063, 3033, 2964, 2925, 2853, 1711, 1646, 1607, 1461, 1260, 1027, 802 cm^{-1}; **^{1}H NMR** (400 MHz, $CDCl_3$) δ 7.35 (m, 10H), 6.55 (s, 1H), 5.05 (m, 3H), 4.43 (d, *J* = 4.1 Hz, 1H), 2.91 (m, 1H), 1.62 (d, *J* = 5.8 Hz, 3H), 1.48 (m, 6H), 1.45 (s, 3H), 1.44 (s, 3H), 1.43 (s, 3H), 1.38 (s, 3H), 1.19 (m, 4H), 0.98 (d, *J* = 7.1 Hz, 3H), 0.91 (d, *J* = 6.8 Hz, 3H), 0.75 (m, 1H); **^{13}C NMR** (101 MHz, $CDCl_3$) δ 212.5, 208.5, 197.8, 168.4, 155.0, 153.4, 153.0, 142.5, 136.3, 128.7 (2 carbons overlapped), 128.5 (2 carbons overlapped), 128.1, 127.9, 127.2 (2 carbons overlapped), 126.3 (2 carbons overlapped), 122.7, 112.4, 112.0, 96.5, 84.3, 70.8, 56.3, 47.5, 45.9, 42.3, 33.2, 29.7, 29.7, 26.7, 26.7, 26.6, 25.4, 25.1, 24.7, 24.2, 22.6, 18.2, 17.8; **HRMS** (ESI-TOF) m/z: $[M+Na]^+$ calcd. for $C_{42}H_{48}O_6Na$ 671.3343; found, 671.3330.

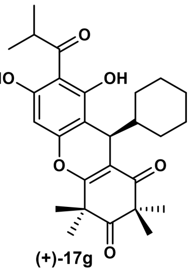

(+)-17g

Compound (+)-**17g**: 13.5 mg, 96% yield, 2 h, yellow solids (20 mg scale); **[*α*]$^{26}_{D}$** = +93.2 (*c* = 0.1 in MeOH); Compound (-)-**17g**: 13.5 mg, 96% yield, 2 h, yellow solids (20 mg scale); **[*α*]$^{26}_{D}$** = −91.8 (*c* = 0.1 in MeOH); **R*f*** = 0.35 (ethyl acetate/*n*-hexane = 1/5); **IR** (KBr): 3223, 2962, 2929, 2851, 1718, 1626, 1584, 1464, 1392, 1151, 1087, 823 cm^{-1}; **^{1}H NMR** (400 MHz, $CDCl_3$) δ 12.92 (s, 1H), 8.68 (s, 1H), 6.21 (s, 1H), 4.33 (d, *J* = 3.2 Hz, 1H), 3.95 (m, 1H), 1.60 (m, 6H), 1.59 (s, 3H), 1.45 (s, 3H), 1.42 (s, 3H), 1.38 (s, 3H), 1.21 (d, *J* = 4 Hz, 3H), 1.19 (d, *J* = 4 Hz, 3H), 1.08

(m, 2H), 0.93 (m, 2H), 0.78 (t, J = 11.0 Hz, 1H); **^{13}C NMR** (101 MHz, $CDCl_3$) δ 212.4, 211.6, 198.8, 168.8, 162.6, 159.2, 156.7, 112.3, 107.2, 104.8, 94.9, 56.3, 47.6, 45.0, 39.9, 31.8, 30.0, 29.3, 26.8, 26.6, 26.5, 25.3, 25.2, 24.8, 24.3, 19.5, 19.2; **HRMS** (ESI-TOF) m/z: $[M+H]^+$ calcd. for $C_{27}H_{35}O_6$ 455.2428; found, 455.2431.

HPLC condition of (+)-17g: Phenomenex Lux Cellulose-4 column; acetonitrile/H_2O = 50:50, 1.0 mL/min, λ = 254 nm; major enantiomer: t_R = 137.6 min, minor enantiomer: t_R = 147.2 min, 96% *ee*.

HPLC condition of (-)-17g: Phenomenex Lux Cellulose-4 column; acetonitrile/H_2O= 50:50, 1.0 mL/min, λ = 254 nm; minor enantiomer: t_R = 138.1 min, major enantiomer: t_R = 146.3 min, 97% *ee*.

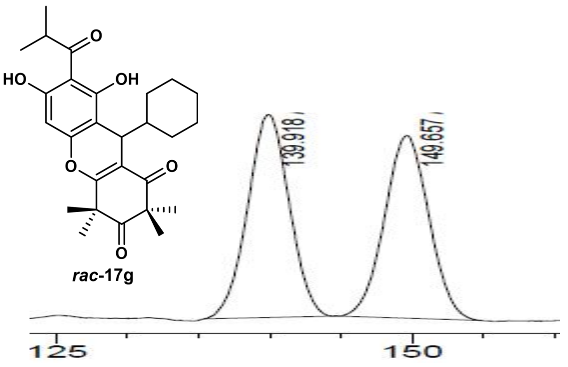

Peak#	Ret. Time	Area	Area%	Height
1	139.918	4930842	51.045	24158
2	149.657	4728984	48.955	21742
Total		9659827	100.000	45901

Supplementary Figure 46. HPLC spectra of *rac*-17g.

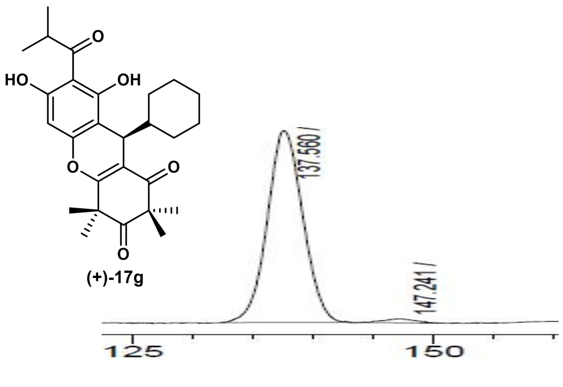

Peak#	Ret. Time	Area	Area%	Height
1	139.918	4930842	51.045	24158
2	149.657	4728984	48.955	21742
Total		9659827	100.000	45901

Supplementary Figure 47. HPLC spectra of (+)-17g.

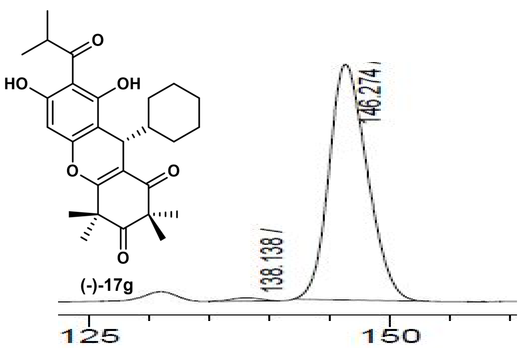

Peak#	Ret. Time	Area	Area%	Height
1	138.138	273316	1.056	1563
2	146.274	25605716	98.944	115311
Total		25879032	100.000	116874

Supplementary Figure 48. HPLC spectra of (-)-17g.

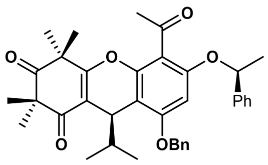

17ha

Compound **17ha**: 20.9 mg, 36% yield, 11 h, white solids (0.1 mmol scale); $\mathbf{R}_f$ = 0.27 (ethyl acetate/*n*-hexane = 1/10); $[\alpha]_D^{26}$ = −42.5 (*c* = 0.1 in CH_2Cl_2); **IR** (KBr): 3068, 3038, 2969, 2929, 2870, 1711, 1665, 1604, 1459, 1382, 1260, 1110, 800 cm^{-1}; ^{1}**H NMR** (400 MHz, $CDCl_3$) δ 7.33 (m, 10H), 6.15 (s, 1H), 5.14 (q, *J* = 6.4 Hz, 1H), 4.86 (s, 2H), 4.30 (d, *J* = 3.6 Hz, 1H), 2.60 (s, 3H), 1.87 (m, 1H), 1.59 (d, *J* = 6.4 Hz, 3H), 1.53 (s, 3H), 1.41 (s, 3H), 1.39 (s, 3H), 1.34 (s, 3H), 0.72 (d, *J* = 6.9 Hz, 3H), 0.69 (d, *J* = 6.9 Hz, 3H); ^{13}**C NMR** (101 MHz, $CDCl_3$) δ 212.7, 199.8, 198.0, 168.7, 157.4, 155.2, 149.4, 142.6, 136.3, 129.0 (2 carbons overlapped), 128.8 (2 carbons overlapped), 128.2, 128.1, 127.1 (2 carbons overlapped), 125.6 (2 carbons overlapped), 113.6, 111.4, 107.2, 96.0, 78.2, 70.4, 56.1, 47.7, 34.9, 32.9, 32.4, 25.1, 25.0, 24.8, 24.4, 24.3, 19.7, 18.9; **HRMS** (ESI-TOF) m/z: [M+Na]$^+$ calcd. for $C_{37}H_{40}O_6Na$ 603.2717; found, 603.2715.

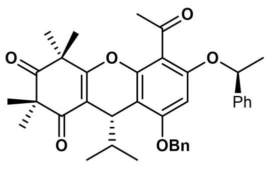

17hb

Compound **17hb**: 22.6 mg, 39% yield, 11 h, white solids (0.1 mmol scale); **R*f*** = 0.37 (EtOAc/*n*-hexane = 1/10); $[\alpha]_D^{26}$ = −45.1 (*c* = 0.5 in CH_2Cl_2); **IR** (KBr): 3065, 3035, 2971, 2932, 2870, 1713, 1651, 1604, 1459, 1378, 1257, 1105, 804 cm^{-1}; **^{1}H NMR** (400 MHz, $CDCl_3$) δ 7.30 (m, 10H), 6.14 (s, 1H), 5.19 (q, *J* = 6.3 Hz, 1H), 4.83 (d, *J* = 12.4 Hz, 1H), 4.79 (d, *J* = 12.4 Hz, 1H), 4.26 (d, *J* = 3.6 Hz, 1H), 2.61 (s, 3H), 1.88 (m, 1H), 1.61 (d, *J* = 6.4 Hz, 3H), 1.53 (s, 3H), 1.41 (s, 3H), 1.38 (s, 3H), 1.33 (s, 3H), 0.76 (d, *J* = 4.7 Hz, 3H), 0.74 (d, *J* = 4.8 Hz, 3H); **^{13}C NMR** (101 MHz, $CDCl_3$) δ 212.7, 199.6, 197.8, 168.7, 157.3, 155.0, 149.5, 142.4, 136.3, 129.0 (2 carbons overlapped), 128.8 (2 carbons overlapped), 128.2, 128.0, 127.1 (2 carbons overlapped), 125.5 (2 carbons overlapped), 113.3, 111.5, 106.9, 95.9, 77.6, 70.5, 56.1, 47.7, 35.0, 32.8, 32.4, 25.2, 24.8, 24.7, 24.6, 24.6, 19.5, 19.1; **HRMS** (ESI-TOF) m/z: $[M+Na]^+$ calcd. for $C_{37}H_{40}O_6Na$ 603.2717; found, 603.2714.

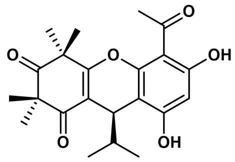

(+)-17h

Compound (+)**-17h**: 12.0 mg, 90% yield, 3 h, white solids (20 mg scale); $[\alpha]_D^{26}$ = +128.7 (*c* = 0.1 in MeOH); Compound (-)**-17h**: 12.2 mg, 92% yield, 3 h, white solids (20 mg scale); $[\alpha]_D^{26}$ = −137.0 (*c* = 0.1 in MeOH); **R*f*** = 0.30 (ethyl acetate/*n*-hexane = 1/5); **IR** (KBr): 3267 2967, 2936, 2871, 1718, 1642, 1503, 1460, 1385, 1253, 1166, 1036, 844 cm^{-1}; **^{1}H NMR** (400 MHz, $CDCl_3$) δ 13.48 (s, 1H), 7.95 (s, 1H), 6.33 (s, 1H), 4.39 (d, *J* = 2.5 Hz, 1H), 2.78 (s, 3H), 1.87 (m, 1H), 1.65 (s, 3H), 1.46 (s, 6H), 1.39 (s, 3H), 0.81 (d, *J* = 6.8 Hz, 3H), 0.78 (d, *J* = 6.8 Hz, 3H); **^{13}C NMR** (101 MHz, $CDCl_3$) δ 211.9, 201.6, 199.5, 168.6, 164.7, 160.7, 154.0, 112.4, 105.2, 104.1, 100.4, 56.3, 47.5, 35.0, 33.1, 31.5, 25.5, 25.3, 25.3, 24.2, 19.1, 18.7; **HRMS** (ESI-TOF) m/z: $[M+H]^+$ calcd. for $C_{22}H_{27}O_6$ 387.1802; found, 387.1800.

HPLC condition of (+)-17h: Phenomenex Lux Cellulose-4 column; acetonitrile/H_2O = 45:55, 1.0 mL/min, λ = 254 nm; major enantiomer: t_R = 39.7 min, minor enantiomer: t_R = 34.2 min, 99% *ee*.

HPLC condition of (-)-17h: Phenomenex Lux Cellulose-4 column; acetonitrile/H_2O= 45:55, 1.0 mL/min, λ = 254 nm; minor enantiomer: t_R = 39.6 min, major enantiomer: t_R = 34.0 min, 99% *ee*.

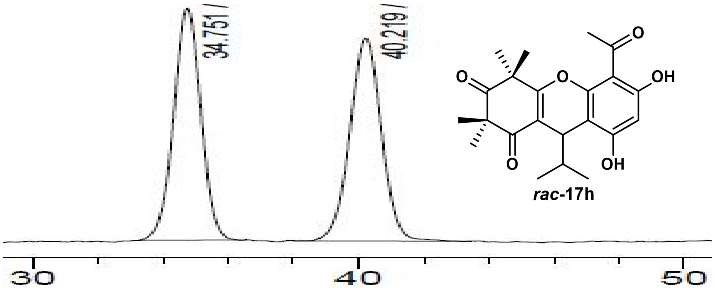

Peak#	Ret. Time	Area	Area%	Height
1	34.751	3647142	49.663	62776
2	40.219	3696618	50.337	54841
Total		7343760	100.000	117617

Supplementary Figure 49. HPLC spectra of *rac*-17h.

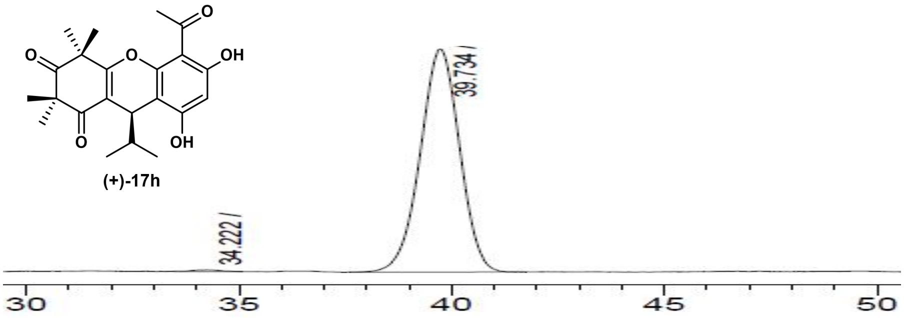

Peak#	Ret. Time	Area	Area%	Height
1	34.222	10818	0.584	236
2	39.734	1842165	99.416	28580
Total		1852983	100.000	28816

Supplementary Figure 50. HPLC spectra of (+)-17h.

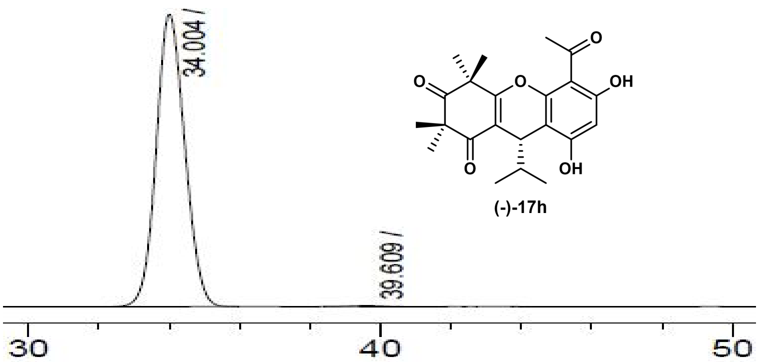

Peak#	Ret. Time	Area	Area%	Height
1	34.004	14570415	99.632	257932
2	39.609	53773	0.368	886
Total		14624188	100.000	258818

Supplementary Figure 51. HPLC spectra of (-)-17h.

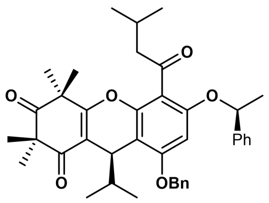

17ia

Compound **17ia**: 21.1 mg, 34% yield, 11 h, white solids (0.1 mmol scale); **R*f*** = 0.30 (ethyl acetate/*n*-hexane = 1/10); $[\alpha]_D^{26}$ = −11.0 (*c* = 0.5 in CH_2Cl_2); **IR** (KBr): 3066, 3033, 2962, 2929, 2867, 1715, 1651, 1581, 1464, 1388, 1184, 1075, 804 cm^{-1}; **^{1}H NMR** (400 MHz, $CDCl_3$) δ 7.33 (m, 10H), 6.13 (s, 1H), 5.09 (q, *J* = 6.4 Hz, 1H), 4.84 (s, 2H), 4.30 (d, *J* = 3.4 Hz, 1H), 2.87 (dd, *J* = 16.4, 6.6 Hz, 1H), 2.71 (dd, *J* = 16.4, 7.1 Hz, 1H), 2.26 (m, 1H), 1.86 (m, 1H), 1.56 (d, *J* = 6.4 Hz, 3H), 1.51 (s, 3H), 1.44 (s, 3H), 1.39 (s, 3H), 1.35 (s, 3H), 1.02 (d, *J* = 6.5 Hz, 3H), 1.00 (d, *J* = 6.5 Hz, 3H), 0.71 (d, *J* = 6.9 Hz, 3H), 0.68 (d, *J* = 6.9 Hz, 3H); **^{13}C NMR** (101 MHz, $CDCl_3$) δ 212.7, 202.8, 198.0, 168.7, 157.1, 154.9, 149.4, 142.7, 136.4, 129.0 (2 carbons overlapped), 128.8 (2 carbons overlapped), 128.2, 128.1, 127.1 (2 carbons overlapped), 125.6 (2 carbons overlapped), 113.5, 111.3, 107.1, 96.1, 78.2, 70.4, 56.1, 54.5, 47.8, 34.9, 32.3, 25.0, 25.0, 24.9, 24.8, 24.6, 24.3, 23.0, 22.8, 19.6, 18.8; **HRMS** (ESI-TOF) m/z: $[M+Na]^+$ calcd. for $C_{40}H_{46}O_6Na$ 645.3187; found, 645.3191.

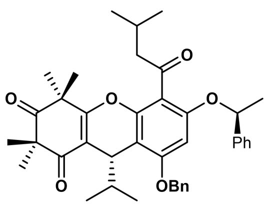

17ib

Compound **17ib**: 23.6 mg, 38% yield, 11 h, white solids (0.1 mmol scale); **R$_f$** = 0.39 (ethyl acetate/*n*-hexane = 1/10); **$[\alpha]_D^{26}$** = −10.1 (*c* = 0.2 in CH_2Cl_2); **IR** (KBr): 3055, 3029, 2967, 2932, 2871, 1715, 1651, 1581, 1459, 1381, 1161, 1073, 809 cm^{-1}; **^{1}H NMR** (400 MHz, $CDCl_3$) δ 7.31 (m, 10H), 6.13 (s, 1H), 5.18 (q, *J* = 6.3 Hz, 1H), 4.82 (d, *J* = 12.3 Hz, 1H), 4.78 (d, *J* = 12.3 Hz, 1H), 4.26 (d, *J* = 3.4 Hz, 1H), 2.88 (dd, *J* = 16.4, 6.7 Hz, 1H), 2.73 (dd, *J* = 16.4, 7.0 Hz, 1H), 2.29 (m, 1H), 1.90 (m, 1H), 1.60 (d, *J* = 6.4 Hz, 3H), 1.51 (s, 3H), 1.43 (s, 3H), 1.38 (s, 3H), 1.33 (s, 3H), 1.03 (d, *J* = 6.4 Hz, 3H), 1.02 (d, *J* = 6.4 Hz, 3H), 0.75 (d, *J* = 6.7 Hz, 3H), 0.73 (d, *J* = 6.7 Hz, 3H); **^{13}C NMR** (101 MHz, $CDCl_3$) δ 212.7, 202.5, 197.9, 168.6, 157.1, 154.7, 149.4, 142.4, 136.4, 129.0 (2 carbons overlapped), 128.8 (2 carbons overlapped), 128.1, 128.0, 127.1 (2 carbons overlapped), 125.4 (2 carbons overlapped), 113.2, 111.3, 106.7, 95.8, 77.4, 70.4, 56.1, 54.4, 47.7, 35.0, 32.3, 25.1, 25.0, 24.9, 24.8, 24.6, 24.5, 23.0, 22.8, 19.4, 19.1; **HRMS** (ESI-TOF) m/z: $[M+Na]^+$ calcd. for $C_{40}H_{46}O_6Na$ 645.3187; found, 645.3178.

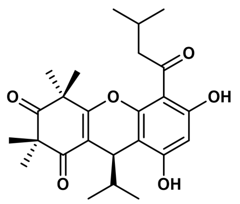

(+)-17i

Compound (+)**-17i**: 12.8 mg, 93% yield, 3 h, white solids (20 mg scale); **$[\alpha]_D^{26}$** = +123.4 (*c* = 0.1 in MeOH); Compound **(-)-17i**: 12.5 mg, 91% yield, 3 h, white solids (20 mg scale); **$[\alpha]_D^{26}$** = −133.2 (*c* = 0.1 in MeOH); **R$_f$** = 0.39 (ethyl acetate/*n*-hexane = 1/5); **IR** (KBr): 3253, 2955, 2932, 2871, 1718, 1630, 1466, 1389, 1249, 1186, 1011, 830 cm^{-1}; **^{1}H NMR** (400 MHz, $CDCl_3$) δ 13.49 (s, 1H), 8.39 (s, 1H), 6.35 (s, 1H), 4.39 (d, *J* = 3.7 Hz, 1H), 3.19 (dd, *J* = 16.7, 7.4 Hz, 1H), 2.89 (dd, *J* = 16.7, 6.4 Hz, 1H), 2.40 – 2.23 (m, 1H), 1.90 (dd, *J* = 6.8, 3.0 Hz, 1H), 1.66 (s, 3H), 1.46 (s, 3H), 1.45 (s, 3H), 1.39 (s, 3H), 1.01 (d, *J* = 6.7 Hz, 3H), 0.98 (d, *J* = 6.6 Hz, 3H), 0.81 (d, *J* = 6.9 Hz,

3H), 0.78 (d, J = 6.9 Hz, 3H); **^{13}C NMR** (101 MHz, $CDCl_3$) δ 212.1, 204.2, 199.6, 168.8, 164.3, 160.5, 153.8, 112.4, 105.2, 104.2, 100.5, 56.2, 53.4, 47.5, 34.9, 31.5, 25.4, 25.3, 25.2, 24.9, 24.2, 22.9, 22.8, 19.1, 18.7; **HRMS** (ESI-TOF) m/z: $[M+H]^+$ calcd. for $C_{25}H_{33}O_6$ 429.2272; found, 429.2272.

HPLC condition of (+)-17i: Phenomenex Lux Cellulose-4 column; acetonitrile/H_2O = 50:50, 1.0 mL/min, λ = 254 nm; major enantiomer: t_R = 46.7 min, minor enantiomer: t_R = 39.3 min, 98.5% *ee*.

HPLC condition of (-)-17i: Phenomenex Lux Cellulose-4 column; acetonitrile/H_2O= 50:50, 1.0 mL/min, λ = 254 nm; minor enantiomer: t_R = 46.9 min, major enantiomer: t_R = 39.0 min, 99% *ee*.

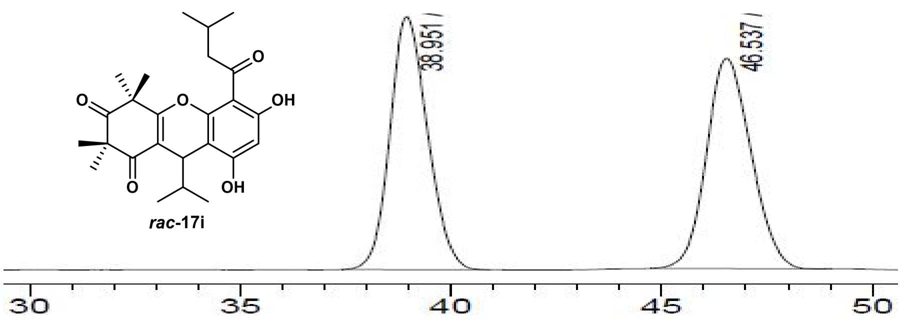

Peak#	Ret. Time	Area	Area%	Height
1	38.951	15988194	50.339	257198
2	46.537	15772553	49.661	213768
Total		31760747	100.000	470966

Supplementary Figure 52. HPLC spectra of *rac*-17i.

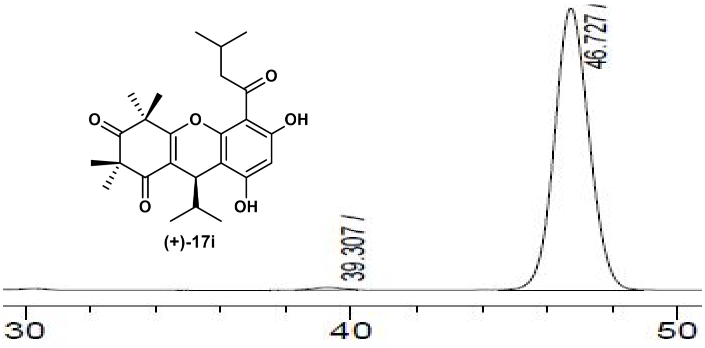

Peak#	Ret. Time	Area	Area%	Height
1	39.307	73068	0.720	1315
2	46.727	10080367	99.280	136688
Total		10153435	100.000	138003

Supplementary Figure 53. HPLC spectra of (+)-17i.

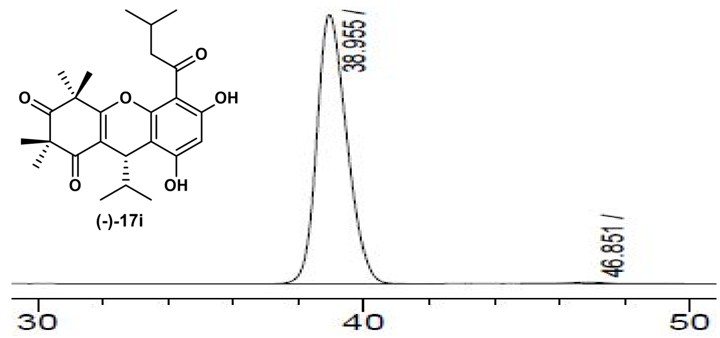

Peak#	Ret. Time	Area	Area%	Height
1	38.955	22629587	99.465	355095
2	46.851	121711	0.535	1502
Total		22751297	100.000	356597

Supplementary Figure 54. HPLC spectra of (-)-17i.

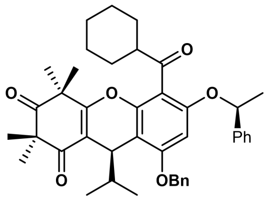

17ja

Compound **17ja**: 22.7 mg, 35% yield, 11 h, white solids (0.1 mmol scale); **R*f*** = 0.26 (ethyl acetate/*n*-hexane = 1/10); **$[\alpha]_D^{26}$** = −17.9 (*c* = 0.1 in CH_2Cl_2); **IR** (KBr): 3057, 3006, 2971, 2925, 2855, 1702, 1646, 1584, 1454, 1381, 1186, 1075, 812 cm^{-1}; **^{1}H NMR** (400 MHz, $CDCl_3$) δ 7.36 (m, 10H), 6.16 (s, 1H), 5.11 (m, 1H), 4.88 (s, 2H), 4.33 (s, 1H), 2.97 (t, *J* = 10.8 Hz, 1H), 1.87 (m, 8H), 1.57 (d, *J* = 6.3 Hz, 3H), 1.51 (s, 3H), 1.44 (s, 3H), 1.40 (s, 3H), 1.38 (m, 3H), 1.37 (s, 3H), 0.74 (d, *J* = 7.2 Hz, 3H), 0.71 (d, *J* = 6.9 Hz, 3H); **^{13}C NMR** (101 MHz, $CDCl_3$) δ 212.7, 206.2, 198.0, 168.7, 157.1, 154.9, 149.4, 142.8, 136.4, 129.0 (2 carbons overlapped), 128.8 (2 carbons overlapped), 128.2, 128.1, 127.1 (2 carbons overlapped), 125.7 (2 carbons overlapped), 113.2, 111.2, 100.1, 96.1, 78.2, 70.4, 56.1, 52.2, 47.8, 35.0, 32.4, 30.3, 28.8, 28.1, 26.1, 26.1, 25.8, 25.0, 25.0, 24.7, 24.7, 24.2, 19.6, 18.8; **HRMS** (ESI-TOF) m/z: $[M+H]^+$ calcd. for $C_{42}H_{49}O_6$ 649.3524; found, 621.3216.

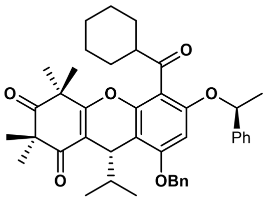

17jb

Compound **17jb**: 25.9 mg, 40% yield, 11 h, white solids (0.1 mmol scale); $\mathbf{R}_f$ = 0.36 (ethyl acetate/*n*-hexane = 1/10); $[\alpha]_D^{26}$ = −35.2 (*c* = 0.2 in CH_2Cl_2); **IR** (KBr): 3066, 3035, 2962, 2923, 2851, 1711, 1649, 1579, 1461, 1388, 1260, 1100, 807 cm^{-1}; **^{1}H NMR** (400 MHz, $CDCl_3$) δ 7.33 (m, 10H), 6.15 (s, 1H), 5.20 (q, *J* = 6.0 Hz, 1H), 4.83 (s, 2H), 4.29 (s, 1H), 3.01 (t, *J* = 10.8 Hz, 1H), 2.01 (m, 2H), 1.93 (m, 1H), 1.85 (m, 2H), 1.65 (m, 3H), 1.61 (d, *J* = 6.3 Hz, 3H), 1.52 (s, 3H), 1.44 (s, 3H), 1.40 (s, 3H), 1.35 (s, 3H), 1.33 (m, 3H), 0.77 (d, *J* = 6.7 Hz, 3H), 0.76 (d, *J* = 6.7 Hz, 3H); **^{13}C NMR** (101 MHz, $CDCl_3$) δ 212.7, 205.9, 197.9, 168.6, 157.1, 154.7, 149.5, 142.5, 136.5, 129.0 (2 carbons overlapped), 128.8 (2 carbons overlapped), 128.1, 128.0, 127.1 (2 carbons overlapped), 125.5 (2 carbons overlapped), 112.8, 111.3, 106.7, 95.9, 77.5, 70.5, 56.0, 52.2, 47.7, 35.1, 32.4, 28.9, 28.1, 26.2 (2 carbons overlapped), 25.8, 25.1, 25.0, 25.0, 24.6, 24.4, 19.4, 19.1; **HRMS** (ESI-TOF) m/z: $[M+H]^+$ calcd. for $C_{42}H_{49}O_6$ 649.3524; found, 649.3517.

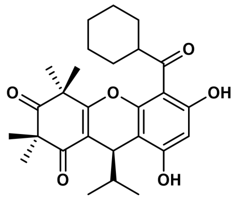

(+)-17j

Compound (+)**-17j**: 13.2 mg, 94% yield, 3 h, white solids (20 mg scale); $[\alpha]_D^{26}$ = +75.9 (*c* = 0.1 in MeOH); Compound **(-)-17j**: 13.3 mg, 95% yield, 3 h, white solids (20 mg scale); $[\alpha]_D^{26}$ = −81.2 (*c* = 0.1 in MeOH); $\mathbf{R}_f$ = 0.34 (ethyl acetate/*n*-hexane = 1/5); **IR** (KBr): 3235, 2957, 2934, 2853, 1713, 1631, 1389, 1257, 1161, 839, 740 cm^{-1}; **^{1}H NMR** (400 MHz, $CDCl_3$) δ 13.13 (s, 1H), 7.33 (s, 1H), 6.31 (s, 1H), 4.36 (d, *J* = 3.7 Hz, 1H), 3.75 (dd, *J* = 14.3, 7.0 Hz, 1H), 1.91 (m, 4H), 1.74 (m, 3H), 1.70 (s, 3H), 1.47 (s, 3H), 1.42 (s, 3H), 1.38 (s, 3H), 1.33 (m, 4H), 0.84 (d, *J* = 6.9 Hz, 3H), 0.80 (d, *J* = 6.9 Hz, 3H); **^{13}C NMR** (101 MHz, $CDCl_3$) δ 211.9, 208.5, 199.0, 168.7, 164.5,

159.6, 153.7, 112.5, 104.3, 104.1, 100.7, 56.4, 49.4, 47.6, 35.0, 31.9, 31.7, 27.5, 26.6, 26.0, 25.7, 25.5, 25.3, 25.0, 24.1, 19.0, 18.9; **HRMS** (ESI-TOF) m/z: $[M+H]^+$ calcd. for $C_{27}H_{35}O_6$ 455.2428; found, 455.2436.

HPLC condition of (+)-17j: Phenomenex Lux Cellulose-4 column; acetonitrile/H_2O = 60:40, 1.0 mL/min, λ = 254 nm; major enantiomer: t_R = 24.3 min, minor enantiomer: t_R = 18.3 min, 96% *ee*.

HPLC condition of (-)-17j: Phenomenex Lux Cellulose-4 column; acetonitrile/H_2O= 60:40, 1.0 mL/min, λ = 254 nm; minor enantiomer: t_R = 24.3 min, major enantiomer: t_R = 18.0 min, 99% *ee*.

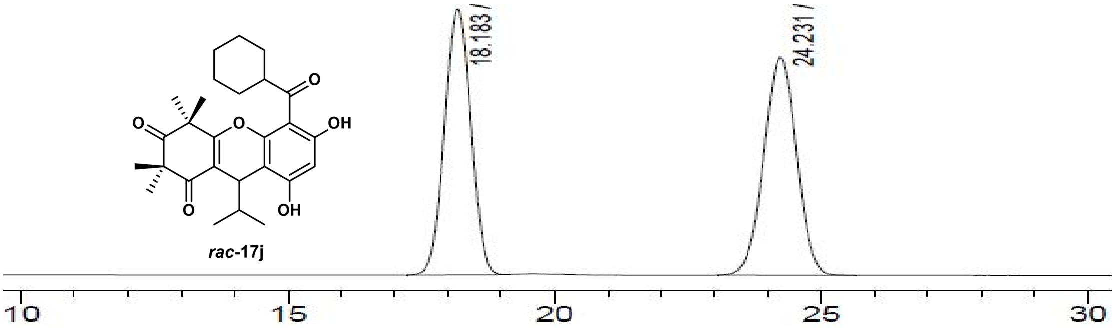

Peak#	Ret. Time	Area	Area%	Height
1	18.183	6175087	49.602	178392
2	24.231	6274222	50.398	146284
Total		12449309	100.000	324676

Supplementary Figure 55. HPLC spectra of *rac*-17j.

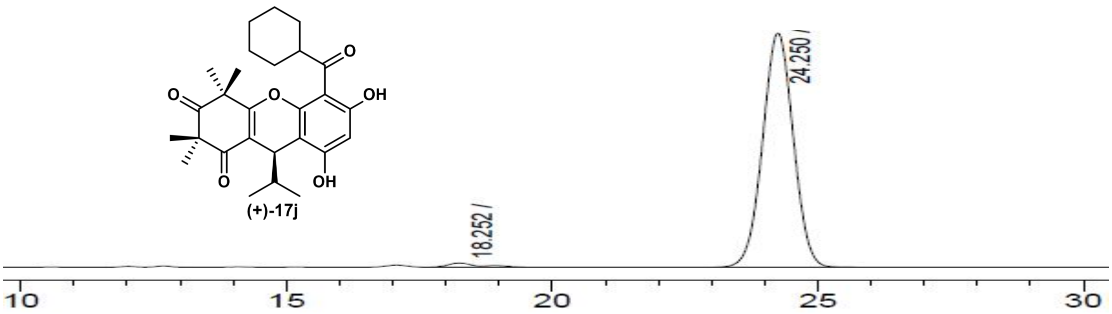

Peak#	Ret. Time	Area	Area%	Height
1	18.252	467872	1.855	11001
2	24.250	24748232	98.145	616305
Total		25216104	100.000	627305

Supplementary Figure 56. HPLC spectra of (+)-17j.

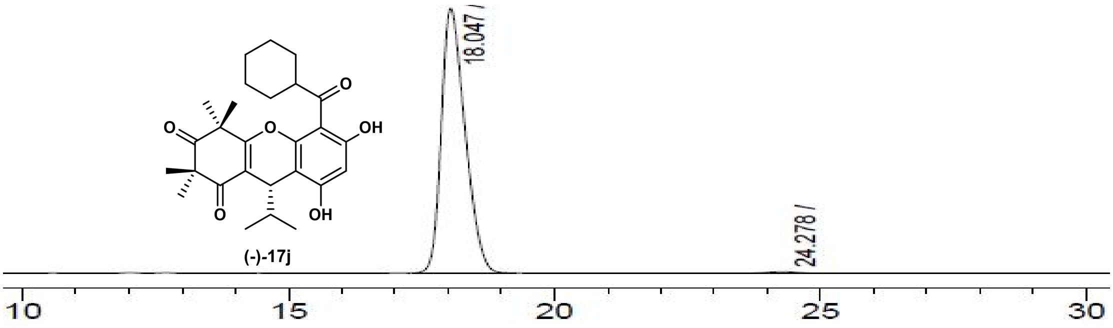

Peak#	Ret. Time	Area	Area%	Height
1	18.047	38351897	99.430	1236683
2	24.278	219984	0.570	6114
Total		38571881	100.000	1242797

Supplementary Figure 57. HPLC spectra of (-)-17j.

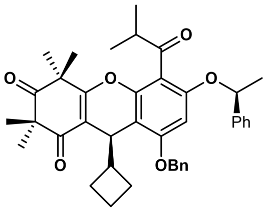

17ka

Compound **17ka**: 21.1 mg, 34% yield, 11 h, white foams (0.1 mmol scale); $\mathbf{R}_f$ = 0.34 (ethyl acetate/*n*-hexane = 1/10); $[\alpha]_D^{26}$ = −20.5 (*c* = 0.1 in CH_2Cl_2); **IR** (KBr): 3066, 3038, 2967, 2929, 2848, 1699, 1651, 1593, 1459, 1385, 1260, 1024, 804 cm^{-1}; ^{1}**H NMR** (400 MHz, $CDCl_3$) δ 7.32 (m, 10H), 6.14 (s, 1H), 5.09 (q, *J* = 6.2 Hz, 1H), 4.84 (s, 2H), 4.23 (d, *J* = 5.5 Hz, 1H), 3.22 (m, 1H), 2.56 (m, 1H), 1.54 (d, *J* = 6.6 Hz, 3H), 1.54 (m, 2H), 1.47 (s, 3H), 1.41 (s, 3H), 1.37 (s, 3H), 1.34 (s, 3H), 1.25 (m, 4H), 1.24 (d, *J* = 6.9 Hz, 3H), 1.23 (d, *J* = 6.8 Hz, 3H); ^{13}**C NMR** (101 MHz, $CDCl_3$) δ 212.6, 207.2, 197.9, 168.3, 157.2, 154.9, 148.9, 142.8, 136.4, 129.0 (2 carbons overlapped), 128.8 (2 carbons overlapped), 128.2, 128.1, 127.2 (2 carbons overlapped), 125.6 (2 carbons overlapped), 113.2, 111.1, 106.1, 96.0, 78.3, 70.4, 56.1, 47.6, 42.5, 41.8, 30.0, 25.3, 25.3, 25.1, 24.9, 24.7, 24.3, 24.2, 18.4, 17.9, 17.9; **HRMS** (ESI-TOF) m/z: $[M+Na]^+$ calcd. for $C_{40}H_{44}O_6Na$ 643.3030; found, 643.3025.

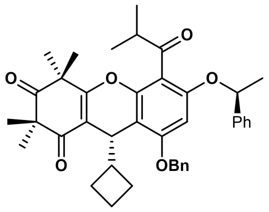

17kb

Compound **17kb**: 24.2 mg, 39% yield, 11 h, white foams (0.1 mmol scale); **R*f*** = 0.43 (ethyl acetate/*n*-hexane = 1/10); $[\alpha]_D^{26}$ = −37.0 (c = 0.1 in CH_2Cl_2); **IR** (KBr): 3063, 3031, 2959, 2923, 2865, 1707, 1646, 1584, 1461, 1385, 1262, 1093, 807 cm^{-1}; **^{1}H NMR** (400 MHz, $CDCl_3$) δ 7.30 (m, 10H), 6.14 (s, 1H), 5.19 (q, J = 6.1 Hz, 1H), 4.80 (m, 2H), 4.19 (d, J = 5.2 Hz, 1H), 3.26 (m, 1H), 2.59 (m, 1H), 1.59 (d, J = 6.0 Hz, 3H), 1.50 (m, 2H), 1.47 (s, 3H), 1.40 (s, 3H), 1.36 (s, 3H), 1.32 (s, 3H), 1.25 (m, 4H), 1.25 (s, 3H), 1.23 (s, 3H); **^{13}C NMR** (101 MHz, $CDCl_3$) δ 212.6, 206.9, 197.8, 168.3, 157.2, 154.7, 148.9, 142.5, 136.4, 129.0 (2 carbons overlapped), 128.8 (2 carbons overlapped), 128.2, 128.0, 127.2 (2 carbons overlapped), 125.4 (2 carbons overlapped), 112.9, 111.0, 105.7, 95.7, 77.4, 70.5, 56.1, 47.6, 42.4, 41.8, 30.0, 25.4, 25.4, 25.4, 24.8, 24.7, 24.7, 23.9, 18.5, 17.9, 17.9; **HRMS** (ESI-TOF) m/z: $[M+Na]^+$ calcd. for $C_{40}H_{44}O_6Na$ 643.3030; found, 643.3033.

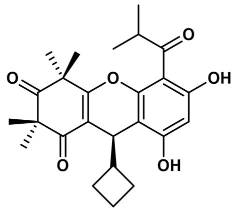

(+)-17k

Compound (+)-**17k**: 12.5 mg, 91% yield, 3 h, white solids (20 mg scale); $[\alpha]_D^{26}$ = +120.6 (c = 0.1 in MeOH); Compound (-)-**17k**: 12.5 mg, 91% yield, 3 h, white solids (20 mg scale); $[\alpha]_D^{26}$ = −131.0 (c = 0.1 in MeOH); **R*f*** = 0.38 (ethyl acetate/*n*-hexane = 1/5); **IR** (KBr): 3389, 2980, 2939, 2870, 1718, 1660, 1639, 1584, 1392, 1219, 1154, 1040, 839 cm^{-1}; **^{1}H NMR** (400 MHz, $CDCl_3$) δ 13.47 (s, 1H), 8.09 (s, 1H), 6.38 (s, 1H), 4.36 (d, J = 5.6 Hz, 1H), 3.90 (m, 1H), 2.60 (d, J = 5.8 Hz, 1H), 1.61 (m, 6H), 1.60 (s, 3H), 1.45 (s, 6H), 1.39 (s, 3H), 1.26 (d, J = 1.8 Hz, 3H), 1.25 (d, J = 1.8 Hz, 3H); **^{13}C NMR** (101 MHz, $CDCl_3$) δ 212.0, 209.1, 199.4, 168.5, 164.9, 160.3, 152.9,

112.0, 103.9, 103.5, 100.8, 56.3, 47.4, 41.8, 39.7, 29.3, 25.3, 25.2, 25.0, 24.7 (2 carbons overlapped), 24.6, 21.0, 17.9, 17.9; **HRMS** (ESI-TOF) m/z: $[M+H]^+$ calcd. for $C_{25}H_{31}O_6$ 427.2115; found, 427.2120.

HPLC condition of (+)-17k: Phenomenex Lux Cellulose-4 column; acetonitrile/H_2O = 43:57, 1.0 mL/min, λ = 254 nm; major enantiomer: t_R = 26.9 min, minor enantiomer: t_R = 24.5 min, 98.5% *ee*.

HPLC condition of (-)-17k: Phenomenex Lux Cellulose-4 column; acetonitrile/H_2O= 43:57, 1.0 mL/min, λ = 254 nm; minor enantiomer: t_R = 26.8 min, major enantiomer: t_R = 24.1min, 99% *ee*.

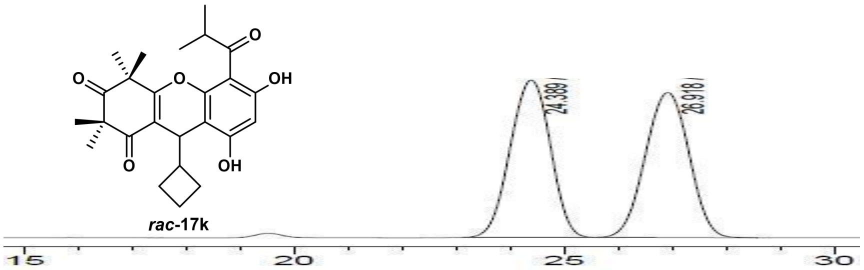

Peak#	Ret. Time	Area	Area%	Height
1	24.389	9252711	50.006	178586
2	26.918	9250594	49.994	164529
Total		18503304	100.000	343114

Supplementary Figure 58. HPLC spectra of *rac*-17k.

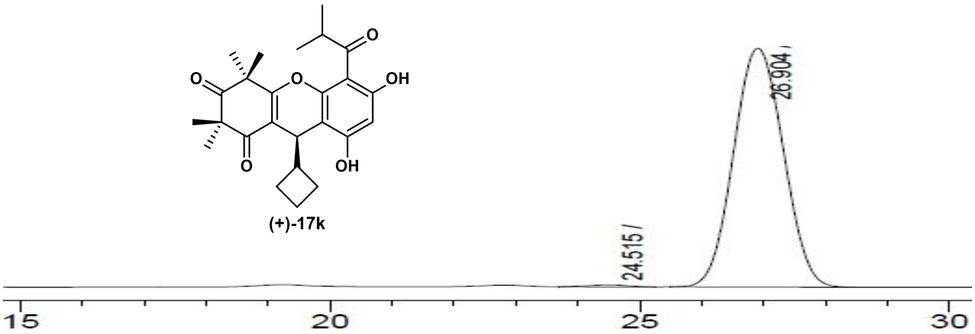

Peak#	Ret. Time	Area	Area%	Height
1	24.515	123206	0.659	2715
2	26.904	18575027	99.341	332010
Total		18698233	100.000	334725

Supplementary Figure 59. HPLC spectra of (+)-17k.

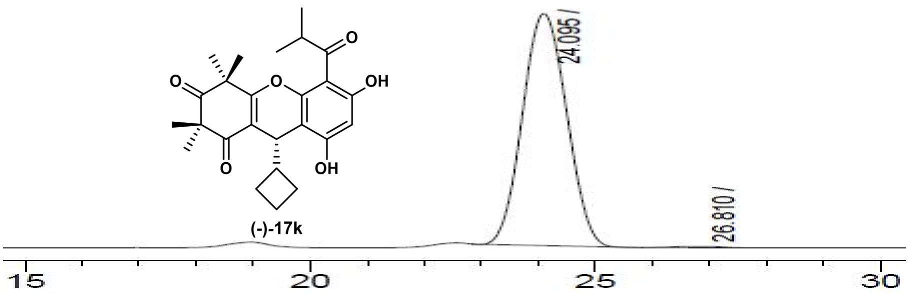

Peak#	Ret. Time	Area	Area%	Height
1	24.095	30612170	99.411	568386
2	26.810	181289	0.589	3299
Total		30793459	100.000	571685

Supplementary Figure 60. HPLC spectra of (-)-17k.

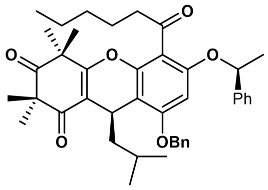

17la

Compound **17la**: 22.7 mg, 35% yield, 11 h, white solids (0.1 mmol scale); **R*f*** = 0.40 (ethyl acetate/*n*-hexane = 1/10); $[\alpha]_D^{26}$ = −22.7 (*c* = 0.18 in CH_2Cl_2); **IR** (KBr): 3066, 3032, 2959, 2929, 2855, 1715, 1649, 1584, 1461, 1383, 1181, 1027, 802 cm^{-1}; **^{1}H NMR** (400 MHz, $CDCl_3$) δ 7.35 (m, 10H), 6.14 (s, 1H), 5.14 (q, *J* = 6.4 Hz, 1H), 4.87 (s, 2H), 4.27 (t, *J* = 5.9 Hz, 1H), 3.90 (m, 2H), 1.75 (m, 2H), 1.59 (d, *J* = 6.4 Hz, 3H), 1.50 (s, 3H), 1.44 (s, 3H), 1.39 (s, 3H), 1.37 (s, 3H), 1.35 (m, 7H), 0.93 (t, *J* = 7.0 Hz, 3H), 0.82 (d, *J* = 6.1 Hz, 3H), 0.71 (d, *J* = 6.1 Hz, 3H); **^{13}C NMR** (101 MHz, $CDCl_3$) δ 212.5, 203.3, 197.8, 167.8, 157.0, 154.8, 148.6, 142.6, 136.2, 129.0 (2 carbons overlapped), 128.7 (2 carbons overlapped), 128.3, 128.0, 127.4 (2 carbons overlapped), 125.6 (2 carbons overlapped), 113.9, 113.8, 108.8, 95.9, 78.0, 70.5, 56.1, 47.5, 46.9, 45.4, 31.7, 25.9, 25.2, 24.9, 24.7, 24.6, 24.5, 24.4, 24.0, 23.3, 23.2, 22.7, 14.1; **HRMS** (ESI-TOF) m/z: $[M+Na]^+$ calcd. for $C_{42}H_{50}O_6Na$ 673.3500; found,673.3502.

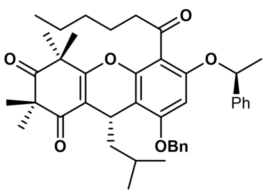

17lb

Compound **17lb**: 25.4 mg, 39% yield, 11 h, white solids (0.1 mmol scale); **R$_f$** = 0.50 (ethyl acetate/*n*-hexane = 1/10); **$[\alpha]_D^{26}$** = −31.1 (*c* = 0.5 in CH_2Cl_2); **IR** (KBr): 3063, 3033, 2959, 2936, 2860, 1711, 1646, 1579, 1472, 1388, 1260, 1080, 802 cm^{-1}; **^{1}H NMR** (400 MHz, $CDCl_3$) δ 7.31 (m, 10H), 6.11 (s, 1H), 5.16 (q, *J* = 6.4 Hz, 1H), 4.82 (s, 2H) 4.21 (dd, *J* = 7.3, 3.9 Hz, 1H), 2.88 (m, 2H), 1.73 (m, 2H), 1.58 (d, *J* = 6.4 Hz, 3H), 1.48 (s, 3H), 1.41 (s, 3H), 1.38 (m, 7H), 1.36 (s, 3H), 1.33 (s, 3H), 0.90 (t, *J* = 6.9 Hz, 3H), 0.81 (d, *J* = 6.0 Hz, 3H), 0.75 (d, *J* = 5.9 Hz, 3H); **^{13}C NMR** (101 MHz, $CDCl_3$) δ 212.5, 203.0, 197.7, 167.7, 156.9, 154.6, 148.6, 142.4, 136.3, 129.0 (2 carbons overlapped), 128.7 (2 carbons overlapped), 128.2, 128.0, 127.3 (2 carbons overlapped), 125.5 (2 carbons overlapped), 113.7, 113.5, 108.5, 95.7, 77.5, 70.5, 56.0, 47.5, 46.6, 45.3, 31.7, 25.6, 25.2, 24.8, 24.8, 24.8, 24.5, 24.2, 24.0, 23.5, 23.3, 22.7, 14.1; **HRMS** (ESI-TOF) m/z: $[M+Na]^+$ calcd. for $C_{42}H_{50}O_6Na$ 673.3500; found,673.3498.

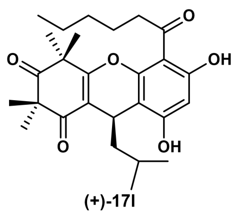

(+)-17l

Compound (+)-**17l**: 13.0 mg, 93% yield, 3 h, white solids (20 mg scale); **$[\alpha]_D^{26}$** = +116.4 (*c* = 0.1 in MeOH); Compound (-)-**17l**: 13.0 mg, 93% yield, 3 h, white solids (20 mg scale); **$[\alpha]_D^{26}$** = −129.1 (*c* = 0.1 in MeOH); **R$_f$** = 0.36 (ethyl acetate/*n*-hexane = 1/5); **IR** (KBr): 3293, 2957, 2936, 2867, 1704, 1658, 1593, 1381, 1251, 1158, 839 cm^{-1}; **^{1}H NMR** (400 MHz, $CDCl_3$) δ 13.55 (s, 1H), 7.54 (s, 1H), 6.29 (s, 1H), 4.32 (t, *J* = 5.5 Hz, 1H), 3.27 (m, 1H), 3.07 (m, 1H), 1.76 (m, 2H), 1.63 (s, 3H), 1.47 (s, 3H), 1.43 (m, 7H), 1.43 (s, 3H), 1.40 (s, 3H), 0.92 (t, *J* = 5.3 Hz, 3H), 0.87 (d, *J* = 4.9 Hz, 3H), 0.85 (d, *J* = 5.0 Hz, 3H); **^{13}C NMR** (101 MHz, $CDCl_3$) δ 211.9, 204.6, 198.8, 167.6, 164.5, 160.0, 153.2, 114.7, 106.1, 105.3, 100.4, 56.2, 47.4, 47.0, 44.9, 31.6, 25.5, 25.2, 24.9, 24.8,

24.6, 24.4, 23.9, 23.6, 23.3, 22.8, 14.1; **HRMS** (ESI-TOF) m/z: $[M+H]^+$ calcd. for $C_{27}H_{37}O_6$ 457.2585; found, 457.2589.

HPLC condition of (+)-17l: Phenomenex Lux Cellulose-4 column; acetonitrile/H_2O = 45:55, 1.0 mL/min, λ = 254 nm; major enantiomer: t_R = 123.8 min, minor enantiomer: t_R = 114.8 min, 96% *ee*.

HPLC condition of (-)-17l: Phenomenex Lux Cellulose-4 column; acetonitrile/H_2O= 45:55, 1.0 mL/min, λ = 254 nm; major enantiomer: t_R = 115.6 min, >99% *ee*.

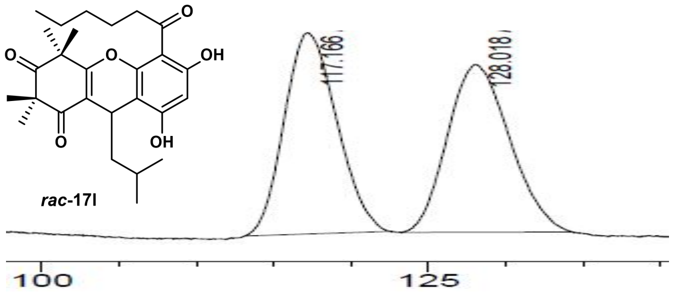

Peak#	Ret. Time	Area	Area%	Height
1	117.166	2488038	50.325	10542
2	128.018	2455874	49.675	8779
Total		4943912	100.000	19321

Supplementary Figure 61. HPLC spectra of *rac*-17l.

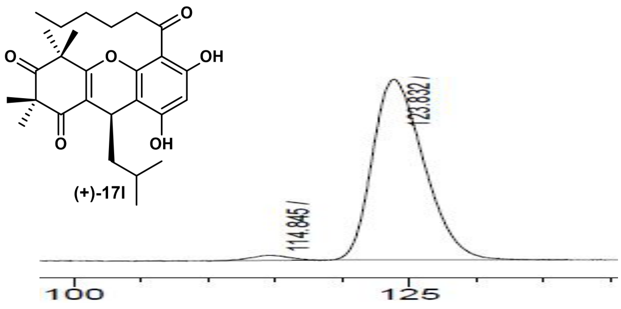

Peak#	Ret. Time	Area	Area%	Height
1	114.845	161784	2.081	796
2	123.832	7612970	97.919	28598
Total		7774755	100.000	29395

Supplementary Figure 62. HPLC spectra of (-)-17l.

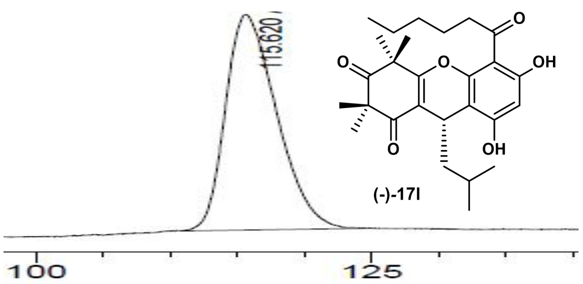

Peak#	Ret. Time	Area	Area%	Height
1	115.620	9127807	100.000	34441
Total		9127807	100.000	34441

Supplementary Figure 63. HPLC spectra of (-)-17l.

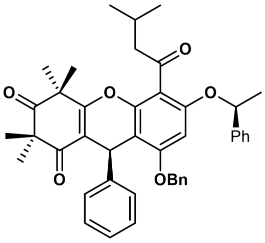

17ma

Compound **17ma**: 21.8 mg, 34% yield, 11 h, white solids (0.1 mmol scale); **R*f*** = 0.25 (ethyl acetate/*n*-hexane = 1/10); $[\alpha]_D^{26}$ = −17.0 (*c* = 0.1 in CH_2Cl_2); **IR** (KBr): 3063, 3029, 2957, 2927, 2851, 1715, 1654, 1581, 1457, 1378, 1184, 1034, 804 cm^{-1}; **^{1}H NMR** (400 MHz, $CDCl_3$) δ 7.31 (m, 8H), 7.15 (m, 5H), 7.01 (d, *J* = 6.4 Hz, 2H), 6.04 (s, 1H), 5.19 (s, 1H), 5.12 (q, *J* = 6.3 Hz, 1H), 4.78 (d, *J* = 12.0 Hz, 1H), 4.68 (d, *J* = 12.0 Hz, 1H), 2.91 (dd, *J* = 16.6, 6.7 Hz, 1H), 2.81 (dd, *J* = 16.6, 6.9 Hz, 1H), 2.34 (m, 1H), 1.59 (d, *J* = 6.4 Hz, 3H), 1.55 (s, 3H), 1.52 (s, 3H), 1.33 (s, 3H), 1.11 (s, 3H), 1.08 (s, 3H), 1.06 (s, 3H); **^{13}C NMR** (101 MHz, $CDCl_3$) δ 212.0, 202.6, 197.2, 165.7, 157.2, 155.5, 147.4, 144.5, 142.5, 135.9, 129.0 (2 carbons overlapped), 128.6 (2 carbons overlapped), 128.5 (2 carbons overlapped), 128.2, 128.1, 128.1 (2 carbons overlapped), 127.4 (2 carbons overlapped), 126.4, 125.4 (2 carbons overlapped), 113.4, 112.5, 107.2, 95.8, 78.1, 70.2, 56.4, 54.48, 47.4, 33.8, 24.9, 24.8, 24.9, 24.5, 24.2, 23.8, 23.0, 22.9; **HRMS** (ESI-TOF) m/z: $[M+H]^+$ calcd. for $C_{43}H_{45}O_6$ 657.3211; found, 657.3204.

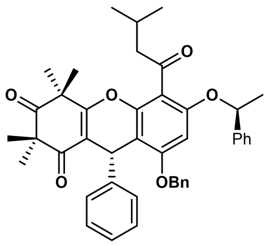

17mb

Compound **17mb**: 25.7 mg, 40% yield, 11 h, white solids (0.1 mmol scale); **R*f*** = 0.33 (EtOAc/*n*-hexane = 1/10); **[*α*]$_D^{26}$** = −26.3 (*c* = 0.1 in CH_2Cl_2); **IR** (KBr): 3063, 3029, 2959, 2929, 2853, 1715, 1656, 1581, 1461, 1378, 1260, 1089, 804 cm^{-1}; **^{1}H NMR** (400 MHz, $CDCl_3$) δ 7.25 (m, 13H), 6.93 (d, *J* = 6.6 Hz, 2H), 5.99 (s, 1H), 5.15 (s, 1H), 5.08 (q, *J* = 6.2 Hz, 1H), 4.75 (d, *J* = 12.2 Hz, 1H), 4.67 (d, *J* = 12.2 Hz, 1H), 2.91 (dd, *J* = 16.5, 6.8 Hz, 1H), 2.81 (dd, *J* = 16.5, 6.9 Hz, 1H), 2.35 (m, 1H), 1.56 (d, *J* = 6.4 Hz, 3H), 1.54 (s, 3H), 1.50 (s, 3H), 1.31 (s, 3H), 1.10 (s, 3H), 1.08 (s, 3H), 1.06 (s, 3H); **^{13}C NMR** (101 MHz, $CDCl_3$) δ 212.0, 202.4, 197.2, 165.6, 157.0, 155.3, 147.3, 144.8, 142.4, 136.0, 129.0 (2 carbons overlapped), 128.6 (2 carbons overlapped), 128.5 (2 carbons overlapped), 128.2 (2 carbons overlapped), 128.1 (2 carbons overlapped), 128.0, 127.3 (2 carbons overlapped), 126.4, 125.4 (2 carbons overlapped), 113.1, 112.3, 107.1, 95.8, 77.6, 70.1, 56.3, 54.4, 47.4, 33.8, 24.9, 24.8, 24.8, 24.5, 24.0, 23.9, 23.0, 22.9; **HRMS** (ESI-TOF) m/z: $[M+H]^+$ calcd. for $C_{43}H_{45}O_6$ 657.3211; found, 657.3214.

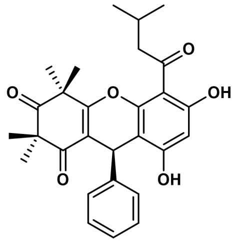

(+)-17m

Compound (+)**-17m**: 13.5 mg, 96% yield, 3 h, white solids (20 mg scale); **[*α*]$_D^{26}$** = +55.3 (*c* = 0.1 in MeOH); Compound **(-)-17m**: 13.4 mg, 95% yield, 3 h, white solids (20 mg scale); **[*α*]$_D^{26}$** = −53.8 (*c* = 0.1 in MeOH); **R*f*** = 0.33 (ethyl acetate/*n*-hexane = 1/5); **IR** (KBr): 3316, 2980, 2955, 2867, 1720, 1649, 1626, 1464, 1366, 1156, 1008, 844 cm^{-1}; **^{1}H NMR** (400 MHz, $CDCl_3$) δ 13.58 (s, 1H), 7.23 (m, 5H), 6.85 (s, 1H), 6.17 (s, 1H), 5.28 (s, 1H), 3.23 (dd, *J* = 17.4, 7.1 Hz, 1H), 3.05 (dd, *J* =

17.4, 6.1 Hz, 1H), 2.41 (m, 1H), 1.68 (s, 3H), 1.56 (s, 3H), 1.35 (s, 3H), 1.11 (s, 3H), 1.06 (s, 6H);

^{13}C NMR (101 MHz, $CDCl_3$) δ 211.3, 204.1, 197.8, 165.1, 159.9, 151.7, 143.9, 128.6 (2 carbons overlapped), 128.2 (2 carbons overlapped), 127.1, 113.3, 105.5, 104.7, 100.9, 100.1, 56.5, 53.9, 47.4, 33.2, 25.5, 25.1, 24.5, 24.5, 23.2, 23.1, 22.9; **HRMS** (ESI-TOF) m/z: $[M+H]^+$ calcd. for $C_{28}H_{31}O_6$ 463.2115; found, 463.2111.

HPLC condition of (+)-17m: Phenomenex Lux Cellulose-4 column; acetonitrile/H_2O = 60:40, 1.0 mL/min, λ = 254 nm; major enantiomer: t_R = 18.6 min, minor enantiomer: t_R = 16.9 min, 99% *ee*.

HPLC condition of (-)-17m: Phenomenex Lux Cellulose-4 column; acetonitrile/H_2O= 60:40, 1.0 mL/min, λ = 254 nm; minor enantiomer: t_R = 18.6 min, major enantiomer: t_R = 16.6 min, 99% *ee*.

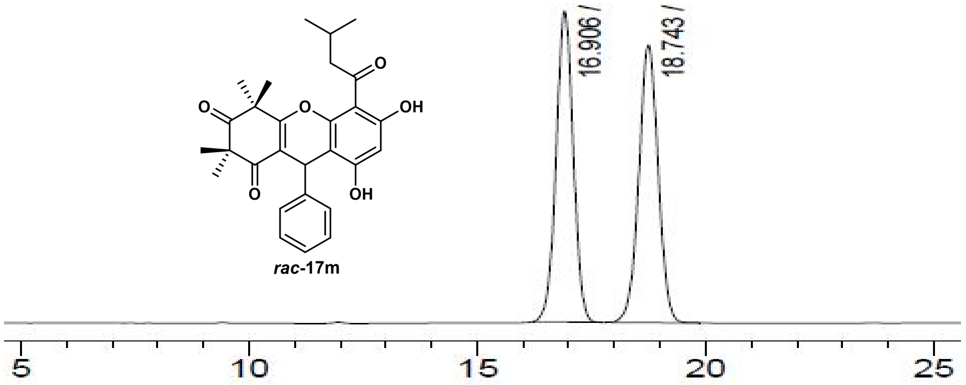

Peak#	Ret. Time	Area	Area%	Height
1	16.906	5831607	49.862	222076
2	18.743	5863913	50.138	198154
Total		11695520	100.000	420230

Supplementary Figure 64. HPLC spectra of *rac*-17m.

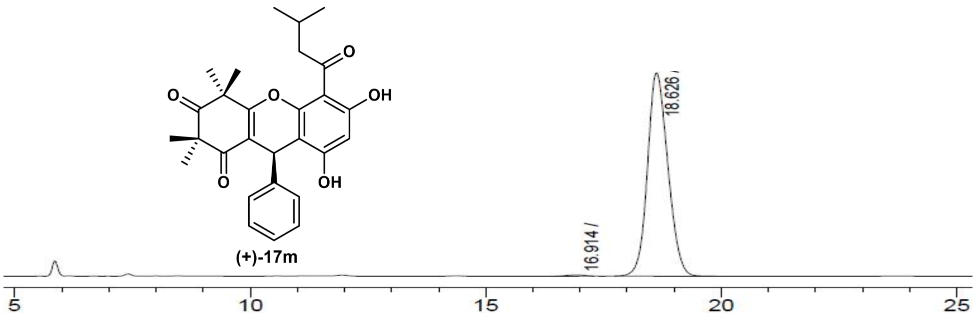

Peak#	Ret. Time	Area	Area%	Height
1	16.914	104182	0.491	4402
2	18.626	21095287	99.509	700897
Total		21199468	100.000	705299

Supplementary Figure 65. HPLC spectra of (+)-17m.

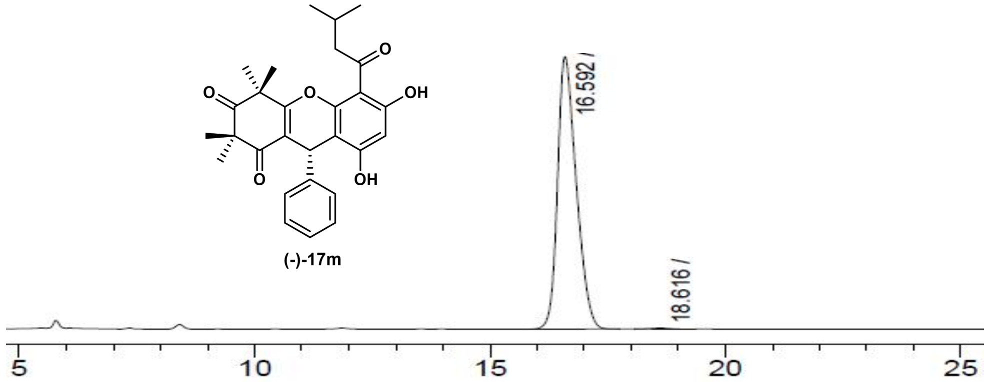

Peak#	Ret. Time	Area	Area%	Height
1	16.592	40655110	99.678	1470799
2	18.616	131128	0.322	4771
Total		40786238	100.000	1475570

Supplementary Figure 66. HPLC spectra of (-)-17m.

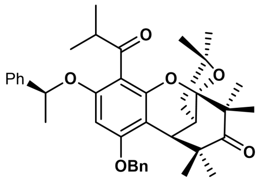

17na

Compound **17na**: 23.1 mg, 35% yield, 11 h, white foams (0.1 mmol scale); **R*f*** = 0.24 (ethyl acetate/*n*-hexane = 1/12); $[\alpha]_D^{26}$ = −32.5 (*c* = 0.3 in CH_2Cl_2); **IR** (KBr): 3089, 3066, 3032, 2969, 2934, 2871, 1699, 1611, 1459, 1364, 1262, 1093, 797 cm^{-1}; **^{1}H NMR** (400 MHz, $CDCl_3$) δ 7.33 (m, 5H), 7.25 (m, 5H), 5.85 (s, 1H), 5.08 (q, *J* = 6.4 Hz, 1H), 4.76 (s, 2H), 3.29 (m, 1H), 3.24 (d, *J* = 3.1 Hz, 1H), 3.09 (m, 1H), 1.77 (dd, *J* = 12.0, 7.4 Hz, 1H), 1.52 (d, *J* = 6.4 Hz, 3H), 1.46 (t, *J* = 12.1 Hz, 1H), 1.35 (s, 3H), 1.33 (s, 3H), 1.30 (s, 3H), 1.23 (s, 3H), 1.23 (s, 3H), 1.15 (*J* = 1.0 Hz, 3H), 1.13 (*J* = 1.0 Hz, 3H), 0.90 (s, 3H); **^{13}C NMR** (101 MHz, $CDCl_3$) δ 218.1, 208.2, 157.6, 155.2, 150.3, 143.1, 136.7, 128.7 (2 carbons overlapped), 128.6 (2 carbons overlapped), 128.0, 127.6, 127.0 (2 carbons overlapped), 125.4 (2 carbons overlapped), 113.1, 111.7, 103.2, 92.0, 84.5, 70.0, 53.8, 50.1, 41.9, 38.9, 38.1, 35.1, 30.0, 29.7, 29.0, 28.8, 24.7, 24.4, 24.2, 19.2, 18.2, 18.0; **HRMS** (ESI-TOF) m/z: $[M+H]^+$ calcd. for $C_{39}H_{47}O_6$ 661.3367; found, 661.3359.

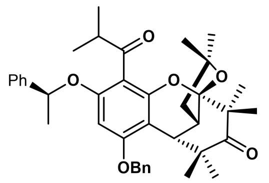

17nb

Compound **17nb**: 25.1 mg, 38% yield, 11 h, white foams (0.1 mmol scale); **R*f*** = 0.33 (ethyl acetate/*n*-hexane = 1/10); **$[\alpha]_D^{26}$** = +22.9 (*c* = 0.3 in CH_2Cl_2); **IR** (KBr): 3087, 3063, 3033, 2969, 2871, 2830, 1703, 1609, 1469, 1257, 1098, 802 cm^{-1}; **^{1}H NMR** (400 MHz, $CDCl_3$) δ 7.34 (m, 10H), 5.90 (s, 1H), 5.13 (q, *J* = 6.3 Hz, 1H), 4.82 (d, *J* = 12.1 Hz, 1H), 4.74 (d, *J* = 12.1 Hz, 1H), 3.29 (m, 1H), 3.23 (d, *J* = 3.0 Hz, 1H), 3.11 (m, 1H), 1.73 (dd, *J* = 12.0, 7.4 Hz, 1H), 1.53 (d, *J* = 6.4 Hz, 3H), 1.45 (t, *J* = 12.1 Hz, 1H), 1.36 (s, 3H), 1.31 (s, 3H), 1.28 (s, 6H), 1.25 (s, 3H), 1.18 (d, *J* = 3.1 Hz, 3H), 1.16 (d, *J* = 3.2 Hz, 3H), 1.01 (s, 3H); **^{13}C NMR** (101 MHz, $CDCl_3$) δ 218.1, 207.9, 157.7, 155.3, 150.3, 143.3, 136.8, 128.8 (2 carbons overlapped), 128.7 (2 carbons overlapped), 128.1, 127.6, 127.2 (2 carbons overlapped), 125.6 (2 carbons overlapped), 111.7, 103.3, 92.0, 84.6, 70.3, 54.0, 50.3, 42.0, 39.0, 38.2, 35.1, 30.0, 29.8, 29.1, 28.9, 24.8, 24.5, 24.3, 19.3, 18.3, 18.1; **HRMS** (ESI-TOF) m/z: $[M+H]^+$ calcd. for $C_{39}H_{47}O_6$ 661.3367; found, 661.3360.

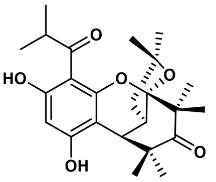

(+)-17n

Compound (+)-**17n**: 13.2 mg, 97% yield, 4 h, white solids (20 mg scale); **$[\alpha]_D^{26}$** = +63.3 (*c* = 0.1 in CH_2Cl_2); Compound **(-)-17n**: 13.1 mg, 96% yield, 4 h, white solids (20 mg scale); **$[\alpha]_D^{26}$** = −51.7 (*c* = 0.1 in CH_2Cl_2); **R*f*** = 0.31 (EtOAc/*n*-hexane = 1/5); **IR** (KBr): 3226, 2979, 2932, 2871, 1711, 1619, 1496, 1419, 1383, 1237, 1057, 916, 878 cm^{-1}; **^{1}H NMR** (400 MHz, $CDCl_3$) δ 13.91 (s, 1H), 6.49 (s, 1H), 5.95 (s, 1H), 3.98 (dd, *J* = 13.3, 6.7 Hz, 1H), 3.41 (t, *J* = 8.5 Hz, 1H), 3.26 (d, *J* = 2.2 Hz, 1H), 1.88 (dd, *J* = 12.5, 8.0 Hz, 1H), 1.48 (t, *J* = 12.3 Hz, 1H), 1.40 (s, 3H), 1.34 (s, 6H), 1.30 (s, 6H), 1.14 (d, *J* = 6.9 Hz, 3H), 1.12 (d, *J* = 6.9 Hz, 3H), 1.01 (s, 3H); **^{13}C NMR** (101 MHz, $CDCl_3$) δ 218.0, 211.3, 165.7, 160.5, 155.7, 114.2, 104.4, 101.4, 96.4, 85.2, 54.1, 50.0, 39.2, 38.3,

38.0, 35.3, 30.2, 29.3, 28.7, 24.6, 24.0, 20.7, 19.6, 19.1; **HRMS** (ESI-TOF) m/z: $[M+H]^+$ calcd. for $C_{24}H_{33}O_6$ 417.2272; found, 417.2265.

HPLC condition of (+)-17n: Phenomenex Lux Cellulose-3 column; acetonitrile/H_2O = 50:50, 1.0 mL/min, λ = 254 nm; major enantiomer: t_R = 9.2 min, minor enantiomer: t_R = 8.1 min, 98.5% *ee*.

HPLC condition of (-)-17n: Phenomenex Lux Cellulose-3 column; acetonitrile/H_2O= 50:50, 1.0 mL/min, λ = 254 nm, >99% *ee*.

Note: The absolute configuration of (+)-**17n** was determined based on X-ray diffraction analysis.

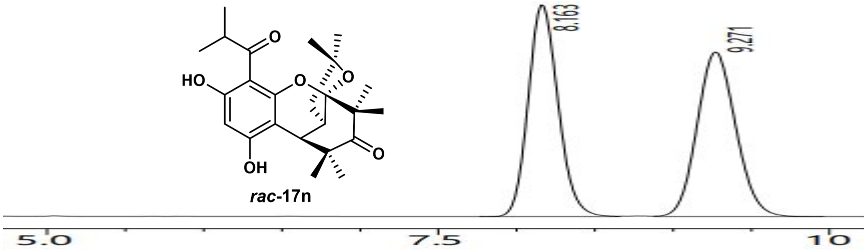

Peak#	Ret. Time	Area	Area%	Height
1	8.163	2561982	49.908	192516
2	9.271	2571456	50.092	149813
Total		5133438	100.000	342329

Supplementary Figure 67. HPLC spectra of *rac*-17n.

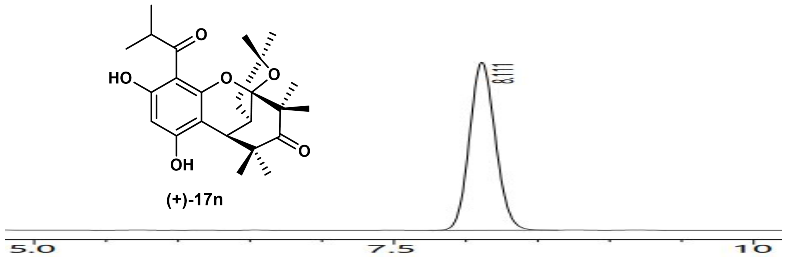

Peak#	Ret. Time	Area	Area%	Height
1	8.111	1890594	100.000	150961
Total		1890594	100.000	150961

Supplementary Figure 69. HPLC spectra of (+)-17n.

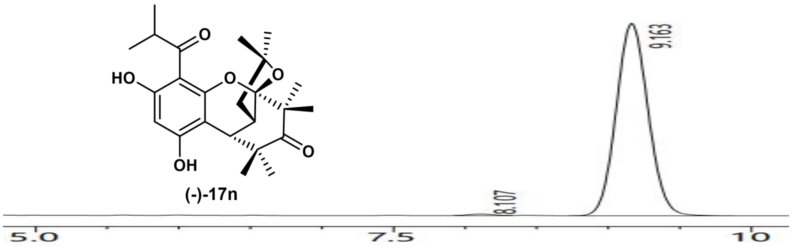

Peak#	Ret. Time	Area	Area%	Height
1	8.107	9136	0.700	736
2	9.163	1296931	99.300	83853
Total		1306068	100.000	84589

Supplementary Figure 68. HPLC spectra of (-)-17n.

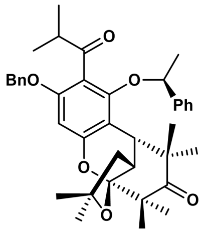

17oa

Compound **17oa**: 21.8 mg, 33% yield, 11 h, white foams (0.1 mmol scale); $\mathbf{R}_f$ = 0.32 (ethyl acetate/*n*-hexane = 1/10); $[\alpha]_D^{26}$ = −24.5 (*c* = 0.2 in CH_2Cl_2); **IR** (KBr): 3063, 3029, 2962, 2925, 2853, 1702, 1608, 1454, 1364, 1257, 1031, 802 cm^{-1}; ^{1}**H NMR** (400 MHz, $CDCl_3$) δ 7.35 (m, 10H), 6.30 (s, 1H), 5.12 (q, *J* = 6.3 Hz, 1H), 5.04 (d, *J* = 11.7 Hz, 1H), 4.93 (d, *J* = 11.7 Hz, 1H), 3.46 (m, 2H), 2.87 (m, 1H), 1.91 (dd, *J* = 11.9, 7.0 Hz, 1H), 1.62 (t, *J* = 12.1 Hz, 1H), 1.48 (s, 3H), 1.38 (s, 6H), 1.36 (d, *J* = 6.5 Hz, 3H), 1.34 (s, 3H), 1.32 (s, 3H), 1.06 (s, 3H), 0.93 (d, *J* = 7.2 Hz, 3H), 0.84 (d, *J* = 6.8 Hz, 3H); ^{13}**C NMR** (101 MHz, $CDCl_3$) δ 217.9, 209.3, 156.0, 153.8, 153.5, 143.0, 136.5, 128.6 (2 carbons overlapped), 128.5 (2 carbons overlapped), 127.9, 127.8, 127.1 (2 carbons overlapped), 125.7 (2 carbons overlapped), 117.8, 111.7, 108.8, 96.2, 84.8, 83.3, 70.4, 54.1, 50.6, 42.4, 39.6, 39.2, 35.3, 30.5, 29.5, 28.8, 24.6, 24.5, 22.9, 19.2, 18.3, 17.1; **HRMS** (ESI-TOF) m/z: $[M+H]^+$ calcd. for $C_{39}H_{47}O_6$ 661.3367; found, 661.3361.

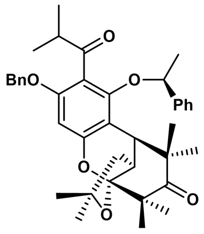

17ob

Compound **17ob**: 23.8 mg, 36% yield, 11 h, white foams (0.1 mmol scale); $\mathbf{R}_f$ = 0.22 (ethyl acetate/*n*-hexane = 1/10); $[\alpha]_D^{26}$ = −56.2 (*c* = 0.3 in CH_2Cl_2); **IR** (KBr): 3089, 3063, 3032, 2967, 2932, 2870, 1702, 1604, 1459, 1366, 1260, 1093, 807 cm^{-1}; ^{1}**H NMR** (400 MHz, $CDCl_3$) δ 7.33 (m, 10H), 6.18 (s, 1H), 4.99 (d, *J* = 11.6 Hz, 1H), 4.90 (d, *J* = 11.6 Hz, 1H), 4.87 (q, 5.7 Hz, 1H), 3.05 (m, 1H), 2.90 (m, 1H), 2.56 (d, *J* = 3.2 Hz, 1H), 1.55 (d, *J* = 6.5 Hz, 3H), 1.36 (m, 2H), 1.23 (s, 3H), 1.21 (s, 6H), 1.17 (d, *J* = 6.9 Hz, 3H), 1.14 (s, 6H), 1.08 (d, *J* = 7.1 Hz, 3H), 0.92 (s, 3H); ^{13}**C NMR** (101 MHz, $CDCl_3$) δ 218.3, 209.6, 155.7, 154.9, 153.8, 142.6, 136.5, 128.6 (4 carbons

overlapped), 128.3, 128.0, 127.3 (2 carbons overlapped), 127.3 (2 carbons overlapped), 118.3, 111.3, 108.3, 96.2, 84.7, 84.5, 70.4, 53.9, 50.4, 42.7, 38.9, 38.8, 34.7, 30.4, 28.9, 28.8, 24.5, 24.2, 23.0, 19.1, 18.2, 17.6; **HRMS** (ESI-TOF) m/z: $[M+H]^+$ calcd. for $C_{39}H_{47}O_6$ 661.3367; found, 661.3358.

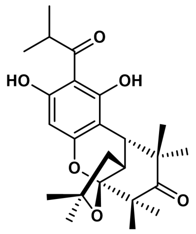

(+)-17o

Compound (+)-**17o**: 13.0 mg, 95% yield, 4 h, white solids (20 mg scale); $[\alpha]_D^{26}$ = +77.5 (*c* = 0.4 in CH_2Cl_2); Compound (-)-**17o**: 13.0 mg, 95% yield, 4 h, white solids (20 mg scale); $[\alpha]_D^{26}$ = −83.3 (*c* = 0.4 in CH_2Cl_2); $\mathbf{R_f}$ = 0.46 (ethyl acetate/*n*-hexane = 1/5); **IR** (KBr): 3229, 2985, 2932, 2867, 1699, 1621, 1468, 1424, 1381, 1226, 1158, 883, 837 cm^{-1}; ^{1}**H NMR** (400 MHz, $CDCl_3$) δ 14.42 (s, 1H), 7.54 (s, 1H), 6.36 (s, 1H), 3.92 (m, 1H), 3.42 (m, 1H), 3.32 (s, 1H), 1.91 (dd, *J* = 11.4, 7.9 Hz, 1H), 1.58 (t, *J* = 12.3 Hz, 1H), 1.40 (s, 3H), 1.38 (s, 3H), 1.34 (s, 3H), 1.29 (s, 3H), 1.26 (s, 3H), 1.18 (d, *J* = 2.9 Hz, 4H), 1.17 (d, *J* = 2.9 Hz, 4H), 1.03 (s, 3H); ^{13}**C NMR** (101 MHz, $CDCl_3$) δ 218.0, 210.9, 165.3, 159.3, 157.8, 112.5, 104.0, 101.3, 94.3, 86.4, 54.1, 50.7, 39.3, 39.0, 37.4, 35.3, 30.1, 29.0, 28.8, 24.7, 24.0, 19.5, 19.3, 19.2; **HRMS** (ESI-TOF) m/z: $[M+H]^+$ calcd. for $C_{24}H_{33}O_6$ 417.2272; found, 417.2270.

HPLC condition of (+)-**17o**: Phenomenex Lux Cellulose-3 column; acetonitrile/H_2O = 50:50, 1.0 mL/min, λ = 254 nm; major enantiomer: t_R = 11.9 min, >99% *ee*.

HPLC condition of (-)-17o: Phenomenex Lux Cellulose-3 column; acetonitrile/H_2O= 50:50, 1.0 mL/min, λ = 254 nm; major enantiomer: t_R = 14.1 min, minor enantiomer: t_R = 11.9 min, 97% *ee*.

Note: The absolute configuration of **(-)-17o** was determined by X-ray diffraction analysis.

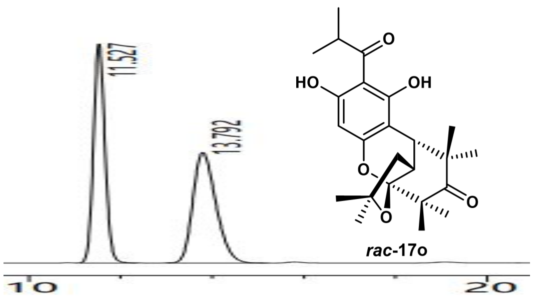

Peak#	Ret. Time	Area	Area%	Height
1	11.527	2392596	49.623	137690
2	13.792	2428964	50.377	69364
Total		4821560	100.000	207054

Supplementary Figure 70. HPLC spectra of *rac*-17o.

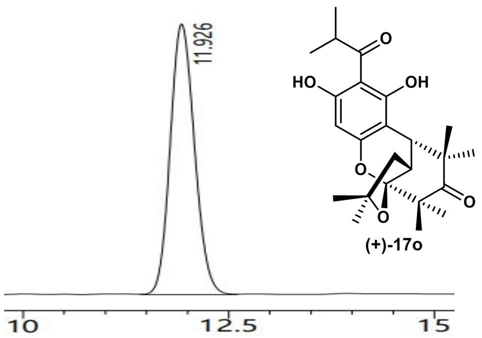

Peak#	Ret. Time	Area	Area%	Height
1	11.926	4121232	100.000	200550
Total	11.926	4121232	100.000	200550

Supplementary Figure 71. HPLC spectra of (+)-17o.

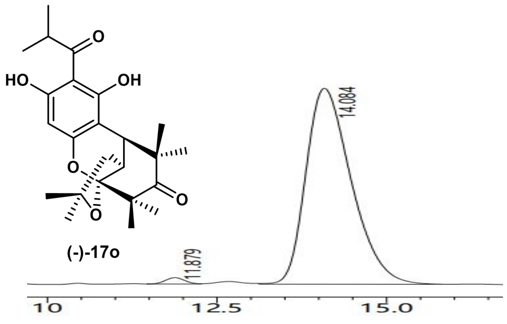

Peak#	Ret. Time	Area	Area%	Height
1	11.879	47977	1.365	2410
2	14.084	3467764	98.635	74520
Total		3515741	100.000	76930

Supplementary Figure 72. HPLC spectra of (-)-17o.

3.5 Synthesis of (+)- and (-)-18, (+)- and (-)-18a-18b

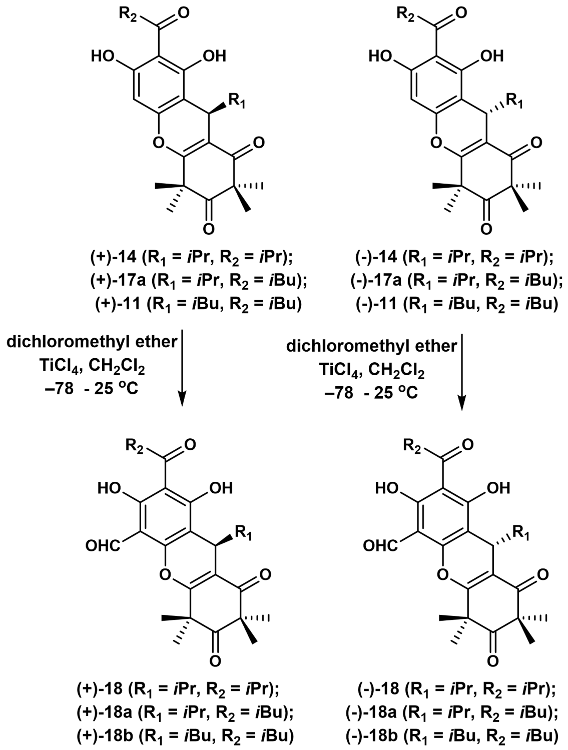

Supplementary Figure 73. Synthesis of (+)- and (-)-18, (+)- and (-)-18a-18b.

To a solution of (+)-**14** or (-)-**14** or (+)-**17a** or (-)-**17a** or (+)-**11** or (-)-**11** (1.0 equiv.) and dichloromethyl ether (9.5 equiv.) in dichloromethane (CH_2Cl_2; 0.15 M) was added titanium tetrachloride ($TiCl_4$, 5.0 equiv.) at −78 ºC. The resulting mixture was stirred for 1 h at −78 ºC, and allowed to warm up to 25 °C. After the reaction was finished according to TLC, the mixture was quenched with saturated aqueous ammonium chloride, and extracted with ethyl acetate. The combined organic layers were dried over Na_2SO_4, filtered and concentrated *in vacuo*. The residue was purified by flash-column chromatography on silica gel (1%-3% ethyl acetate/*n*-hexane) to afford the corresponding products (+)-**18**, (-)-**18**, (+)-**18a**, (-)-**18a**, (+)-**18b**, and (-)-**18b**.

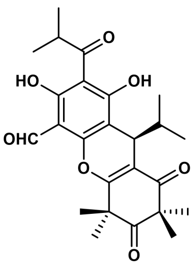

(+)-18

Compound **18**: 999 mg, 78% yield, 12 h, yellow solids (1.2 g scale); $[\alpha]_D^{26}$ = +170.0 (c = 0.1 in CH_2Cl_2); Compound (-)-**18**: 999 mg, 78% yield, 12 h, yellow solids (1.2 g scale); $[\alpha]_D^{26}$ = −163.6 (c = 0.1 in CH_2Cl_2); R_f = 0.32 (ethyl acetate/n-hexane = 1/10); **IR (KBr)**: 3205, 2979, 2875, 1720, 1660, 1466, 1291, 1119, 997, 869 cm^{-1}; ^{1}**H NMR** (400 MHz, $CDCl_3$) δ 15.53 (s, 1H), 14.27 (s, 1H), 10.16 (s, 1H), 4.30 (s, 1H), 3.94 (m, 1H), 2.00 (m, 1H), 1.63 (s, 3H), 1.44 (s, 6H), 1.37 (s, 3H), 1.21 (s, 6H), 0.79 (s, 6H); ^{13}**C NMR** (101 MHz, $CDCl_3$) δ 211.7, 211.4, 197.5, 190.3, 171.8, 167.4, 166.7, 157.4, 112.6, 105.6, 105.1, 102.8, 56.5, 47.5, 39.9, 34.3, 31.5, 25.4 (2 carbons overlapped), 25.0, 23.6, 19.6, 19.1, 18.9, 18.7; **HRMS** (ESI-TOF) m/z: $[M-H]^-$ calcd. for $C_{25}H_{29}O_7$ 441.1913; found, 441.1918.

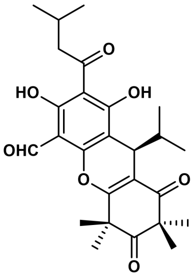

(+)-18a

Compound (+)-**18a**: 410 mg, 77% yield, 12 h, yellow solids, according to procedure E (500 mg scale); $[\alpha]_D^{26}$ = +173.0 (c = 0.1 in CH_2Cl_2); Compound (-)-**18a**: 405 mg, 76% yield, 12 h, yellow solids, according to procedure E (500 mg scale); $[\alpha]_D^{26}$ = −162.9 (c = 0.1 in CH_2Cl_2); R_f = 0.33 (ethyl acetate/n-hexane = 1/10); **IR (KBr)**: 3164, 2871, 1720, 1662, 1422, 1291, 1162, 1119, 999 cm^{-1}; ^{1}**H NMR** (400 MHz, $CDCl_3$) δ 15.46 (s, 1H), 14.16 (s, 1H), 10.14 (s, 1H), 4.26 (d, J = 3.4 Hz, 1H), 2.96 (m, 2H), 2.25 (m, 1H), 1.95 (m, 1H), 1.59 (s, 3H), 1.42 (s, 3H), 1.39 (s, 3H), 1.33 (s, 3H), 0.95 (d, J = 6.9 Hz, 3H),0.94 (d, J = 6.9 Hz, 3H), 0.75 (d, J = 7.0 Hz, 3H), 0.74 (d, J = 7.0 Hz, 3H); ^{13}**C NMR** (101 MHz, $CDCl_3$) δ 211.3, 206.8, 197.3, 190.3, 171.3, 167.5, 166.6, 157.4, 112.5,

106.5, 104.9, 102.7, 56.4, 53.1, 47.5, 34.2, 31.4, 25.3 (2 carbons overlapped), 25.0, 24.8, 23.5, 22.8, 22.7, 19.5, 18.7; **HRMS** (ESI-TOF) m/z: $[M-H]^-$ calcd. for $C_{26}H_{31}O_7$ 455.2070; found, 455.2073.

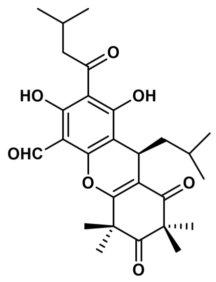

(+)-18b

Compound (+)**-18b**: 425 mg, 80% yield, 12 h, yellow solids (500 mg scale); $[\alpha]_D^{26}$ = +168.6 (*c* = 0.1 in CH_2Cl_2); Compound (**-**)**-18b**: 425 mg, 80% yield, 12 h, yellow solids (500 mg scale); $[\alpha]_D^{26}$ = −151.7 (*c* = 0.1 in CH_2Cl_2); R_f = 0.33 (ethyl acetate/*n*-hexane = 1/10); **IR (KBr)**: 3257, 2964, 2925, 2848, 1707, 1631, 1461, 1385, 1160, 1033, 846 cm^{-1}; ^{1}**H NMR** (400 MHz, $CDCl_3$) δ 15.46 (s, 1H), 14.17 (s, 1H), 10.16 (s, 1H), 4.24 (t, *J* = 5.7 Hz, 1H), 3.01 (dd, *J* = 8.5, 6.7 Hz, 2H), 2.25 (m, 1H), 1.58 (s, 3H), 1.45 (s, 3H), 1.40 (s, 3H), 1.40 (m, 3H), 1.37 (s, 3H), 0.99 (s, 3H), 0.98 (s, 3H), 0.88 (d, *J* = 6.0 Hz, 3H), 0.85 (d, *J* = 5.9 Hz, 3H); ^{13}**C NMR** (101 MHz, $CDCl_3$) δ 211.3, 207.0, 197.4, 190.3, 171.3, 167.6, 165.7, 156.7, 115.1, 106.7, 106.4, 103.0, 56.4, 53.2, 47.4, 46.1, 25.3, 25.1, 25.0, 25.0, 24.8, 24.8, 23.9, 23.4, 23.2, 22.9, 22.8; **HRMS** (ESI-TOF) m/z: $[M+H]^+$ calcd. for $C_{27}H_{35}O_7$ 471.2377; found, 471.2374.

3.6 Synthesis of (+)- and (-)-1-3

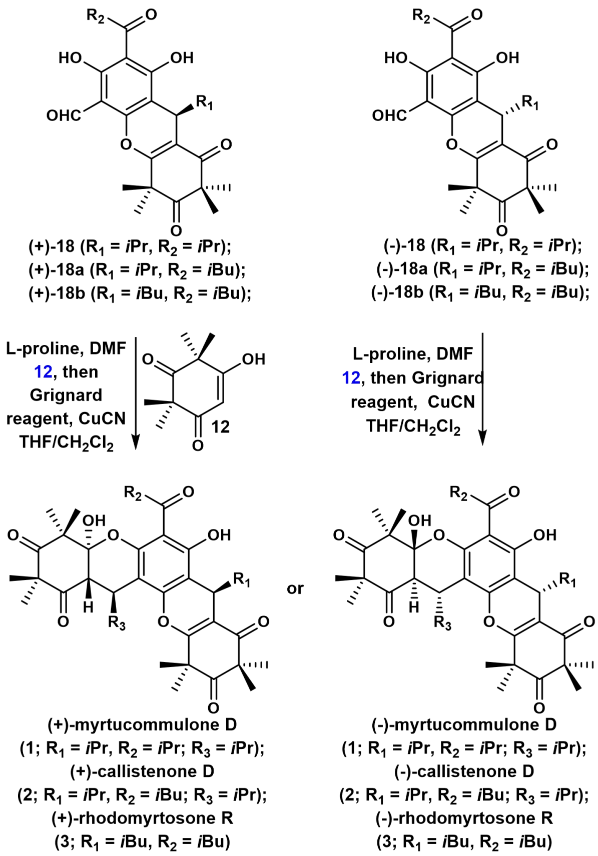

Supplementary Figure 74. Synthesis of (+)- and (-)-1-3.

To a solution of (+)-**18** or (+)-**18a-18b** or (-)-**18** or (-)-**18a-18b** (1.0 equiv.) in dimethyl formamide (DMF) under argon were added L-proline (1.0 equiv.) and **12**. The resulting mixture was stirred for 12 h at 25 °C. The mixture was poured into hexane and directly filtered, then concentrated *in vacuo* to afford crude.

To a solution of the crude in tetrahydrofuran-dichloromethane (THF-CH_2Cl_2; v/v =1:1; 0.05M) under argon were added copper(I) cyanide (CuCN; 1.1 equiv.) and Grignard reagent (*i*PrMgBr or *i*BuMgBr; 3.5 equiv.) at −78 °C. The resulting mixture was allowed to warm up to −50 °C over 1 h. The mixture was quenched with saturated aqueous ammonium chloride, and extracted with ethyl acetate. The combined organic layers were dried over Na_2SO_4, filtered and concentrated *in vacuo*. The residue was purified by flash-column chromatography on silica gel (1.5%-5% ethyl acetate/*n*-hexane) to afford the corresponding products (+)- and (-)-**1-3**.

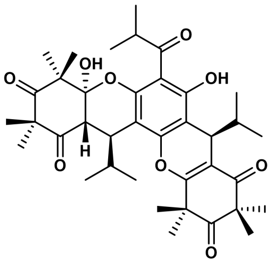

(+)-myrtucommulone D (1)

Compound (+)-myrtucommulone D (**1**): 116.9 mg, 66% yield, 5 h (120 mg scale), white solids; **[*α*]$_D^{26}$** = +188.7 (*c* = 0.1 in CH_2Cl_2); Compound (-)-myrtucommulone D (**1**): 116.9 mg, 66% yield, 5 h (120 mg scale), white solids; **[*α*]$_D^{26}$** = −193.2 (*c* = 0.1 in CH_2Cl_2); **R_f** = 0.42 (ethyl acetate/*n*-hexane = 1/8); **IR (KBr)**: 3247, 2975, 2874, 1725, 1612, 1466, 1267, 1078, 999, 775 cm^{-1}; **^{1}H NMR** (600 MHz, $CDCl_3$) δ 13.37 (s, 1H), 4.35 (d, *J* = 3.2 Hz, 1H), 4.17 (dd, *J* = 5.9, 3.6 Hz, 1H), 3.90 (m, 1H), 3.66 (d, *J* = 3.2 Hz, 1H), 2.34 (m, 1H), 2.01 (m, 1H), 1.62 (s, 3H), 1.58 (s, 3H), 1.55 (s, 3H), 1.41 (s, 3H), 1.40 (s, 6H), 1.33 (s, 3H), 1.29 (s, 3H),1.21 (d, *J* = 7.0 Hz, 3H), 1.17 (d, *J* = 6.6 Hz, 3H), 0.90 (d, *J* = 6.9 Hz, 3H), 0.75 (d, *J* = 6.8 Hz, 3H), 0.71 (d, *J* = 6.8 Hz, 3H), 0.66 (d, *J* = 6.8 Hz, 3H); **^{13}C NMR** (101 MHz, $CDCl_3$) δ 214.4, 212.2, 210.5, 205.2, 197.8, 167.6, 161.2, 153.5, 150.9, 111.5, 108.6, 108.3, 107.1, 100.2, 57.9, 56.3, 55.1, 47.5, 45.8, 40.0, 34.2, 32.3, 32.0, 29.1, 27.0, 25.4, 25.4, 25.0, 24.4, 24.2, 22.2, 20.6, 20.2, 19.6, 19.3, 18.3, 18.0, 16.0; **HRMS** (ESI-TOF) m/z: $[M-H]^-$ calcd. for $C_{38}H_{49}O_9$ 649.3377; found, 649.3377.

Supplementary Table 2. Compared NMR data [$CDCl_3$] between synthetic myrtucommulone D (**1**) and the isolated natural product reported by Shaheen, F. and co-workers [3].

	^1H & ppm (J)			^{13}C & ppm		
position	isolated (400M)	synthesized (600M)	error (iso. - syn.)	isolated (101M)	synthesized (101M)	error (iso. - syn.)
1	-	-	-	106.7	107.1	-0.4
2	-	-	-	150.6	150.9	-0.3
3	-	-	-	108.5	108.3	0.2
4-OH	-	13.37*	-	161.4	161.2	0.2
5	-	-	-	108.6	108.6	0
6	-	-	-	153.5	153.5	0
7	4.18 (dd, 3.3, 3.5)	4.17 (dd, 5.9, 3.6)	0.01	28.9	29.1	-0.2
8	2.35 (m)	2.34 (m)	0.01	32.2	32.3	-0.1
9	0.66 (d, 6.8)	0.66 (d, 6.8)	0	16.0	16.0	0
10	0.89 (d, 7.0)	0.90 (d, 6.9)	-0.01	20.2	20.2	0
1'	3.67(d, 5.9)	3.66 (d, 5.9)	0.01	45.7	45.8	-0.1
2'	-	-	-	204.9	205.2	-0.3
3'	-	-	-	56.2	56.3	-0.1
4'	-	-	-	211.9	212.2	-0.3
5'	-	-	-	54.9	55.1	-0.2
6'	-	-	-	100.1	100.2	-0.1
7'	-	-	-	210.2	210.5	-0.3
8'	3.89 (m)	3.90 (m)	-0.01	39.9	40.0	-0.1
9'	1.20 (d, 7.0)	1.21 (d, 7.0)	-0.01	20.4	20.6	-0.2
10'	1.16 (d, 6.6)	1.17 (d, 6.6)	-0.01	17.9	18.0	-0.1
11'	1.57 (s)	1.58 (s)	-0.01	25.4	25.6	-0.2
12'	1.29 (s)	1.29 (s)	0	24.2	24.2	0
13'	1.56 (s)	1.55 (s)	0.01	24.7	25.0	-0.3
14'	1.40 (s)	1.40 (s)	0	19.2	19.3	-0.1
1"	-	-	-	111.6	111.5	0.1
2"	-	-	-	197.5	197.8	-0.3
3"	-	-	-	57.3	57.9	-0.6
4"	-	-	-	213.8	214.4	-0.6
5"	-	-	-	47.4	47.5	-0.1
6"	-	-	-	167.4	167.6	-0.2
7"	4.37 (d, 3.2)	4.35 (d, 3.1)	0.02	32.0	32.0	0
8"	2.01 (m)	2.01 (m)	0	34.2	34.2	0
9"	0.88 (d,)	0.71 (d, 6.8)	0.17	20.0	19.7	0.3
10"	0.75 (d,)	0.75 (d, 6.8)	0	18.3	18.3	0
11"	1.47 (s)	1.41 (s)	0.06	24.9	27.0	-2.1
12"	1.32 (s)	1.33 (s)	-0.01	22.1	22.2	-0.1
13"	1.39 (s)	1.40 (s)	-0.01	25.3	25.4	-0.1
14"	1.62 (s)	1.62 (s)	0	24.3	24.4	-0.1

Supplementary Table 3. Compared NMR data [$CDCl_3$] between our synthetic myrtucommulone D (**1**) and the isolated natural product reported by Ni, W. and co-workers.[4]

	^1H & ppm (J)			^{13}C & ppm		
position	isolated (600M)	synthesized (600M)	error (iso. - syn.)	isolated (151M)	synthesized (101M)	error (iso. - syn.)
1	-	-	-	106.8	107.1	-0.3
2	-	-	-	150.6	150.9	-0.3
3	-	-	-	108.3	108.3	0
4-OH	13.36*	13.37*	-0.01	161.1	161.2	-0.1
5	-	-	-	108.4	108.6	-0.2
6	-	-	-	153.4	153.5	-0.1
7	4.17 (dd, 6.0, 3.6)	4.17 (dd, 5.9, 3.6)	0	28.8	29.1	-0.3
8	2.34 (m)	2.34 (m)	0	32.1	32.3	-0.2
9	0.65 (d, 6.8)	0.66 (d, 6.8)	-0.01	15.8	16.0	-0.2
10	0.90 (d, 6.9)	0.90 (d, 6.9)	0	20.1	20.2	-0.2
1'	3.66 (d, 6.0)	3.66 (d, 5.9)	0	45.5	45.8	-0.3
2'	-	-	-	204.9	205.2	-0.3
3'	-	-	-	56.2	56.3	-0.1
4'	-	-	-	212.0	212.2	-0.2
5'	-	-	-	54.9	55.1	-0.2
6'	-	-	-	100.0	100.2	-0.2
7'	-	-	-	210.3	210.5	-0.2
8'	3.89 (m)	3.90 (m)	-0.01	39.9	40.0	-0.1
9'	1.21 (d, 6.8)	1.21 (d, 7.0)	0	20.5	20.6	-0.1
10'	1.16 (d, 6.8)	1.17 (d, 6.6)	-0.01	17.8	18.0	-0.2
11'	1.58 (s)	1.58 (s)	0	25.3	25.6	-0.3
12'	1.28 (s)	1.29 (s)	-0.01	24.0	24.2	-0.2
13'	1.54 (s)	1.55 (s)	-0.01	24.8	25.0	-0.2
14'	1.39 (s)	1.40 (s)	-0.01	19.1	19.3	-0.2
1"	-	-	-	111.3	111.5	-0.2
2"	-	-	-	197.6	197.8	-0.2
3"	-	-	-	56.2	57.9	-1.7
4"	-	-	-	214.0	214.4	-0.4
5"	-	-	-	47.4	47.5	-0.1
6"	-	-	-	167.4	167.6	-0.2
7"	4.33 (d, 3.3)	4.35 (d, 3.1)	-0.02	31.8	32.0	-0.2
8"	2.00 (m)	2.01 (m)	-0.01	34.1	34.2	-0.1
9"	0.70 (d, 6.9)	0.71 (d, 6.8)	-0.01	19.5	19.7	-0.2
10"	0.74 (d, 6.9)	0.75 (d, 6.8)	-0.01	18.2	18.3	-0.1
11"	1.41 (s)	1.41 (s)	0	26.8	27.0	-0.2
12"	1.31 (s)	1.33 (s)	-0.02	22.0	22.2	-0.2
13"	1.38 (s)	1.40 (s)	-0.02	25.2	25.4	-0.2
14"	1.61 (s)	1.62 (s)	-0.01	24.3	24.4	-0.1

Note: (1) The structure of synthetic (+)-myrtucommulone D (**1**) was confirmed by X-ray diffraction analysis. The ^1H and ^{13}C NMR data of the synthesized **1** was assigned by ^1H-^1H COSY, HSQC, HMBC, NOESY spectra. Compared NMR data between our synthetic **1** and the isolated **1** reported by Shaheen, F. and co-workers[3] as well as Ni, W. and co-workers,[4] they are the same compound. Thus, the ^1H NMR data of H9″ and ^{13}C NMR data of C11″ first reported by Shaheen, F. and co-workers needs to be revised slightly, respectively. The data of ^{13}C NMR data of C3″ reported by Ni, W. and co-workers needs to be revised slightly (56.2 was listed two times by mistake).

(2) Treatment of (+)-myrtucommulone D (**1**; 16.3 mg, 1.0 equiv.) with NaOH (2.0 equiv.) and NIS (1.05 equiv.) was in THF (2 mL) at −40 °C or −20 °C or 0 °C or 25 °C (Supplementary Figure 75).

The resulting mixture was stirred for about 1-3 h. The mixture was quenched with saturated aqueous sodium sulphite (5 mL), and extracted with ethyl acetate (5 mL). The combined organic layers were dried over Na_2SO_4, filtered and concentrated *in vacuo*. The residue was purified by flash-column chromatography on silica gel (1.5%-5% ethyl acetate/*n*-hexane) to afford **1a**, but not observed its isomer. The structure of **1a** was confirmed by X-ray diffraction analysis. The mechanism of stereoselective transformation was proposed in the Scheme S9. Of note, exposure of **1** to NaOH without NIS could recover the starting martial. This result suggested that *retro*-hemiketalization indeed probably occurred.

Compound **1a**

$[\alpha]_D^{26}$ = −45.8 (*c* = 0.1 in CH_2Cl_2); **R_f** = 0.41 (ethyl acetate/*n*-hexane = 1/10); **^{1}H NMR** (600 MHz, $CDCl_3$) δ 13.66 (s, 1H), 4.37 (d, *J* = 3.4 Hz, 1H), 4.00 (d, *J* = 2.4 Hz, 1H), 3.57 (m, 1H), 2.34 (m, 1H), 2.03 (m, 1H), 1.58 (s, 3H), 1.57 (s, 3H), 1.46 (s, 3H), 1.43 (s, 3H), 1.42 (s, 3H), 1.35 (s, 3H), 1.33 (s, 3H), 1.29 (s, 3H), 1.22 (d, *J* = 7.0 Hz, 3H), 1.16 (d, *J* = 6.5 Hz, 3H), 1.12 (d, *J* = 7.0 Hz, 3H); **^{13}C NMR** (151 MHz, $CDCl_3$) δ 212.0, 209.1, 208.4, 202.4, 201.7, 197.4, 167.5, 162.8, 157.3, 153.2, 112.4, 107.9, 104.5, 103.4, 100.2, 59.6, 56.5, 56.4, 47.9, 47.4, 38.9, 34.4, 32.1, 29.4, 25.6, 25.6, 24.8, 24.5, 24.4, 23.7, 23.0, 22.8, 22.7, 20.5, 19.6, 18.8, 17.8, 17.2; **HRMS** (ESI-TOF) m/z: $[M-H]^-$ calcd. for $C_{38}H_{49}O_9$ 649.3371; found, 649.3376.

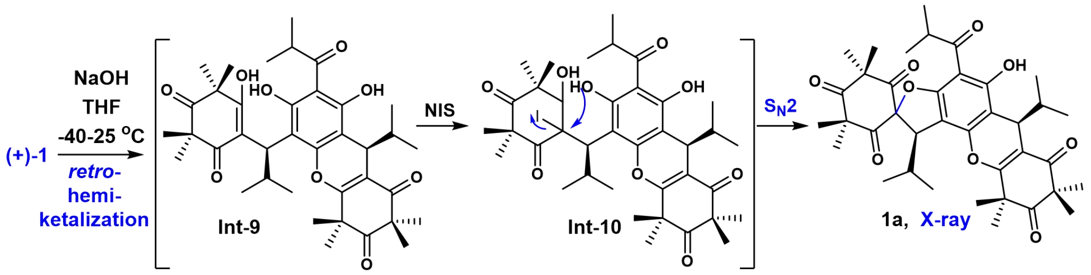

Supplementary Figure 75. Synthesis of 1a.

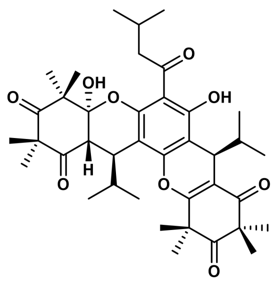

(+)-callistenone D (2)

Compound (+)-callistenone D (**2**): 108.3 mg, 62% yield, 5 h (120 mg scale), white solids; **$[\alpha]_D^{26}$** = +179.6 (c = 0.1 in CH_2Cl_2); Compound (-)-callistenone D (**2**): 111.8 mg, 64% yield, 5 h (120 mg scale), white solids; **$[\alpha]_D^{26}$** = −185.3 (c = 0.1 in CH_2Cl_2); **R_f** = 0.45 (ethyl acetate/*n*-hexane = 1/8); **IR (KBr)**: 3176, 2958, 2873, 1703, 1613, 1467, 1367, 1264, 1120, 1001 cm^{-1}; **^{1}H NMR** (400 MHz, $CDCl_3$) δ 13.46 (s, 1H), 4.36 (d, *J* = 2.8 Hz, 1H), 4.18 (d, J = 3.9 Hz, 1H), 3.67 (d, *J* = 5.9 Hz, 1H), 3.14 (dd, *J* = 17.1, 7.0 Hz, 1H), 2.88 (dd, *J* = 17.0, 6.6 Hz, 1H), 2.36 (m, 1H), 2.30 (m, 1H), 2.01 (m, 1H), 1.63 (s, 3H), 1.62 (s, 3H), 1.58 (s, 3H), 1.48 (s, 3H), 1.42 (s, 6H), 1.36 (s, 3H), 1.32 (s, 3H), 1.00 (s, 3H), 0.98 (d, *J* = 6.4 Hz, 3H), 0.93 (d, *J* = 6.9 Hz, 3H), 0.76 (d, *J* = 7.3 Hz, 3H), 0.74 (d, *J* = 6.8 Hz, 3H), 0.68 (d, *J* = 6.8 Hz, 3H); **^{13}C NMR** (101 MHz, $CDCl_3$) δ 213.9, 212.0, 205.5, 205.0, 197.6, 167.4, 160.8, 153.5, 151.0, 111.5, 110.0, 108.2, 106.8, 100.1, 57.8, 56.2, 54.9, 53.8, 47.4, 45.7, 34.2, 32.1, 31.8, 28.9, 26.8, 25.3, 25.3, 24.8, 24.7, 24.4, 24.2, 22.8, 22.7, 22.1, 20.2, 19.5, 19.4, 18.3, 16.0; **HRMS** (ESI-TOF) m/z: $[M-H]^-$ calcd. for $C_{39}H_{51}O_9$ 663.3533; found, 663.3538.

Supplementary Table 4. Compared NMR data [$CDCl_3$] between our synthetic callistenone D (**2**) and the isolated natural product reported by Carroll, A. R. and and co-workers.[5]

	^{1}H & ppm (J)			*^{13}C & ppm*		
position	isolated (600M)	synthesized (400M)	error (iso. - syn.)	isolated (151M)	synthesized (101M)	error (iso. - syn.)
1	-	-	-	106.7	106.8	-0.1
2	-	-	-	151.2	151.0	0.2
3	-	-	-	109.8	110.0	-0.2
4-OH	-	13.46*	-	160.9	160.8	0.1
5	-	-	-	108.3	108.2	0.1
6	-	-	-	153.1	153.5	-0.4
7	-	-	-	32.3	32.1	0.2
8	4.17 (dd, 6.3, 3.9)	4.18 (d, 3.9)	-0.01			
	2.36-2.43 (m)	2.36 (m)	-	16.0	16.0	0
9	0.68 (d, 6.9)	0.68 (d, 6.8)	0	20.1	20.2	-0.1
10	0.92 (d, 6.9)	0.93 (d, 6.9)	-0.01	46.3	45.7	0.6
1'	0.99 (d, 6.9)	1.00 (s)	-0.01	205.0	205.0	0
2'	3.68 (d, 6.3)	3.67 (d, 5.9)	0.01	57.1	57.8	-0.7
3'	-	-	-	213.5	213.9	0.4
4'	-	-	-	56.0	54.9	-1.1
5'	-	-	-	100.0	100.1	-0.1
6'	-	-	-	28.8	28.9	-0.1
7'	-	-	-	205.5	205.5	0
8'	3.14 (dd, 16.8, 6.9)	3.14 (dd, 17.1, 7.0)	0	54.3	53.8	0.5
9'	2.89 (dd, 16.8, 6.9)	2.88 (dd, 17.0, 6.6)	0.01	24.4	24.7	-0.3
10'	2.31-2.39 (m)	2.30 (m)	-	22.1	22.7	-0.6
11'	0.98 (d, 6.9)	0.98 (d, 6.4)	0	22.1	22.8	-0.7
12'	1.32 (s)	1.32 (s)	0.01	22.1	22.1	0
13'	1.43 (s)	1.42 (s)	0.01	26.7	26.8	-0.1
14'	1.47 (s)	1.48 (s)	-0.01	19.4	19.4	0
15'	1.62 (s)	1.62 (s)	0	24.5	24.4	0.1
1"	-	-	-	111.4	111.5	-0.1
2"	-	-	-	197.4	197.6	-0.2
3"	-	-	-	56.3	56.2	0.1
4"	-	-	-	212.0	212.0	0
5"	-	-	-	47.0	47.4	-0.4
6"	-	-	-	167.4	167.4	0
7"	4.39 (d, 3.3)	4.36 (d, 2.8)	0.03	32.1	31.8	-0.3
8"	2.00-2.12 (m)	2.01 (m)	-	34.0	34.2	-0.2
9"	0.74, (d, 3.9)	0.74 (d, 6.8)	0	18.8	18.3	0.5
10"	0.77 (d, 3.9)	0.76 (d, 7.3)	0.01	19.0	19.5	-0.5
11"	1.38 (s)	1.36 (s)	0.02	24.2	24.2	0
12"	1.44 (s)	1.42 (s)	0.02	25.0	24.8	0.2
13"	1.64 (s)	1.63 (s)	0.01	25.4	25.3	0.1
14"	1.60 (s)	1.58 (s)	0.02	25.2	25.3	-0.1

Supplementary Table 5. Compared NMR data [$CDCl_3$] between our synthetic callistenone D (**2**) and the isolated natural product reported by Ni, W. and co-workers.[4]

	^1H & ppm (J)			^{13}C & ppm		
position	isolated (600M)	synthesized (400M)	error (iso. - syn.)	isolated (151M)	synthesized (101M)	error (iso. - syn.)
1	-	-	-	106.7	106.8	-0.1
2	-	-	-	150.9	151.0	-0.1
3	-	-	-	109.9	110.0	-0.1
4-OH	13.50*	13.46*	0.04	160.8	160.8	0
5	-	-	-	108.2	108.2	0
6	-	-	-	153.5	153.5	0
7	-	-	-	205.4	205.5	-0.1
8	4.17 (dd, 5.9, 3.6)	4.18 (d, 3.9)	-0.01	32.1	32.1	0
	2.37 (m)	2.36 (m)	0.01			-
9	0.68 (d, 6.9)	0.68 (d, 6.8)	0	15.9	16.0	-0.1
10	0.92 (d, 6.9)	0.93 (d, 6.9)-	-0.01	20.1	20.2	-0.1
1'	0.99 (d, 7.0)	1.00 (s)	-0.01	205.0	205.0	0
2'	3.68 (d, 6.0)	3.67 (d, 5.9)	0	57.9	57.8	0.1
3'	-	-	-	213.9	213.9	0
4'	-	-	-	54.9	54.9	0
5'	-	-	-	100.1	100.1	0
6'	-	-	-	28.9	28.9	0
7'	-	-	-	22.0	22.1	-0.1
8'	3.14 (dd, 17.1, 7.0)	3.14 (dd, 17.1, 7.0)	0	53.8	53.8	0
9'	2.89 (dd, 17.1, 6.7)	2.88 (dd, 17.0, 6.6)	0.01	24.6	24.7	-0.1
10'	2.31 (m)	2.30 (m)	0.01	22.7	22.8	-0.1
11'	0.98 (d, 7.0)	0.98 (d, 6.4)	0	22.8	22.8	0
12'	1.32 (s)	1.32 (s)	0	45.6	45.7	-0.1
13'	1.42 (s)	1.42 (s)	0	26.8	26.8	0
14'	1.47 (s)	1.48 (s)	-0.01	19.4	19.4	0
15'	1.60 (s)	1.62 (s)	-0.02	24.4	24.4	0
1"	-	-	-	111.5	111.5	0
2"	-	-	-	197.6	197.6	0
3"	-	-	-	56.2	56.2	0
4"	-	-	-	212.0	212.0	0
5"	-	-	-	47.4	47.4	0
6"	-	-	-	167.4	167.4	0
7"	4.38 (d, 3.2)	4.36 (d, 2.8)	0.02	31.8	31.8	0
8"	2.03 (m)	2.01 (m)	0.02	34.2	34.2	0
9"	0.74, (d, 6.9)	0.74 (d, 6.8)	0	18.3	18.3	0
10"	0.77 (d, 6.9)	0.76 (d, 7.3)	0.01	19.4	19.5	-0.1
11"	1.37 (s)	1.36 (s)	0.01	24.3	24.2	0.1
12"	1.43 (s)	1.42 (s)	0.01	24.7	24.8	-0.1
13"	1.63 (s)	1.63 (s)	0	25.3	25.3	0
14"	1.58 (s)	1.58 (s)	0	25.4	25.3	0.1

Note: The structure of synthetic callistenone D (**2**) was confirmed by X-ray diffraction analysis. The ^1H and ^{13}C NMR data of the synthesized **2** was assigned by ^1H-^1H COSY, HSQC, HMBC, NOESY spectra. Compared NMR data between our synthetic **2** and the isolated **2** reported by Carroll, A. R. and co-workers[5] as well as Ni, W. and co-workers,[4] respectively, they are the same compound. Therefore, the ^{13}C NMR data reported by Carroll, A. R. and co-workers needs to be revised slightly. The reason may be that the relative scarcity of this compound (1.8 mg) results in an unclear signs of the carbon in **2**.

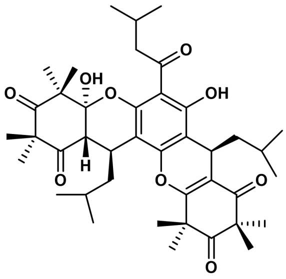

(+)-rhodomyrtosone R (3)

Compound (+)-rhodomyrtosone R (**3**): 51.2 mg, 58% yield, 5 h (60 mg scale), white solids; **$[\alpha]_D^{26}$** = +150.6 (*c* = 0.1 in CH_2Cl_2); Compound (-)-rhodomyrtosone R (**3**): 50.4 mg, 57% yield, 5 h, (60 mg scale), white solids; **$[\alpha]_D^{26}$** = −163.3 (*c* = 0.1 in CH_2Cl_2); **R_f** = 0.46 (ethyl acetate/*n*-hexane = 1/8); **IR (KBr)**: 3450, 2952, 2870, 1725, 1660, 1603, 1470, 1369, 1295, 1163, 1038 cm^{-1}; **^1H NMR** (600 MHz, $CDCl_3$) δ 13.78 (s, 1H), 4.27 (t, *J* = 6.0 Hz, 1H), 3.86 (dd, *J* = 12.3, 4.7 Hz, 1H), 3.33 (d, *J* = 6.0, 1H), 2.77 (dd, *J* = 18.2, 6.1 Hz, 1H), 2.65 (dd, *J* = 18.2, 7.2 Hz, 1H), 2.26 (m, 1H), 2.19 (m, 1H), 1.87 (m, 1H), 1.72 (m, 1H), 1.62 (s, 3H), 1.54 (s, 3H), 1.51 (s, 3H), 1.39 (m, 2H), 1.40 (m, 1H), 1.39 (s, 3H), 1.37 (s, 3H), 1.34 (s, 6H), 1.16 (d, *J* = 6.5 Hz, 3H), 1.01 (d, *J* = 6.5 Hz, 3H), 0.95 (d, *J* = 6.7 Hz, 3H), 0.91 (s, 3H), 0.88 (d, *J* = 6.7 Hz, 3H), 0.87 (d, *J* = 6.6 Hz, 3H), 0.85 (d, *J* = 6.4 Hz, 3H); **^{13}C NMR** (151 MHz, $CDCl_3$) δ 212.2, 211.0, 210.8, 205.7, 197.5, 166.8, 161.6, 153.7, 150.8, 114.8, 109.4, 108.6, 104.5, 100.8, 56.3, 55.0, 54.5, 54.2, 47.3, 46.5, 45.9, 42.0, 30.4, 26.6, 25.6, 25.3, 25.1, 25.1, 24.9, 24.8, 24.7, 24.3, 24.2, 24.1, 23.4, 23.1, 22.8, 22.5, 22.2, 21.0, 16.0; **HRMS** (ESI-TOF) m/z: $[M+H]^+$ calcd. for $C_{41}H_{57}O_9$ 693.3997; found, 693.3977.

Supplementary Table 6. Compared NMR data [$CDCl_3$] between our synthetic rhodomyrtosone R (**3**) and the isolated natural product.[6]

	^1H & ppm (J)			*^{13}C & ppm*		
position	isolated (600M)	synthesized (600M)	error (iso. - syn.)	isolated (151M)	synthesized (151M)	error (iso. - syn.)
1	-	-	-	109.3	109.4	-0.1
2	-	-	-	153.7	153.7	0
3	-	-	-	104.5	104.5	0
4-OH	13.78*(s)	13.78*(s)	0	150.7	150.8	-0.1
5	-	-	-	108.6	108.6	0
6	-	-	-	161.5	161.6	-0.1
7'	3.86 (dd, 12, 4.4)	3.86 (dd, 12.3, 4.7)	0	30.3	30.4	-0.1
8'	2.19 (m)	2.19 (m)	0	42.0	42.0	0
	1.73 (m)	1.72 (m)	0.01			
9'	1.87 (m)	1.87 (m)	0	25.3	25.3	0
10'	1.16 (d, 5.2)	1.16 (d, 6.5)	0	20.9	21.0	-0.1
11'	1.00 (d, 5.2)	1.01 (d, 6.5)	-0.01	22.7	22.8	-0.1
1'	3.33 (d, 6.0)	3.33 (m, 6.0)	0	45.9	45.9	0
2'	-	-	-	210.9	211.0	-0.1
3'	-	-	-	55.0	55.0	0
4'	-	-	-	210.7	210.8	-0.1
5'	-	-	-	54.5	54.5	0
6'	-	-	-	100.7	100.8	-0.1
7	-	-	-	205.7	205.7	0
8	2.78 (dd, 16.8, 6.0)	2.77 (dd, 18.2, 6.1)	0.01	54.2	54.3	-0.1
	2.65 (dd, 16.8, 6.0)	2.65 (dd, 18.2, 7.2)	0			
9	2.26 (m)	2.26 (m)	0	24.2	24.2	0
10	0.88 (d, 6.4)	0.88 (d, 6.7)	0	23.1	23.1	0
11	0.95 (d, 5.2)	0.95 (d, 6.7)	0	22.5	22.5	0
12'	0.91 (s)	0.91 (s)	0	24.3	24.3	0
13'	1.34 (s)	1.34 (s)	0	22.2	22.2	0
14'	1.54 (s)	1.54 (s)	0	15.9	16.0	-0.1
15'	1.37 (s)	1.37 (s)	0	24.6	24.7	-0.1
1"	-	-	-	114.8	114.8	0
2"	-	-	-	197.4	197.5	-0.1
3"	-	-	-	56.3	56.3	0
4"	-	-	-	212.2	212.2	0
5"	-	-	-	47.3	47.3	0
6"	-	-	-	166.8	166.8	0
7"	4.27 (t,6.0)	4.27 (t, 6.0)	0	25.1	25.1	0
8"	1.39 (m)	1.39 (m)	0	46.4	46.5	-0.1
9"	1.40 (m)	1.40 (m)	0	25.6	25.6	0
10"	0.85 (d, 6.4)	0.85 (d, 6.4)	0	23.4	23.4	0
11"	0.86 (d, 6.4)	0.87 (d, 6.6)	-0.01	23.1	23.1	0
12"	1.39 (s)	1.39 (s)	0	25.1	25.1	0
13"	1.34 (s)	1.34 (s)	0	26.6	26.6	0
14"	1.62 (s)	1.62 (s)	0	24.9	24.9	0
15"	1.51 (s)	1.51 (s)	0	24.7	24.8	-0.1

3.7 Synthesis of (+)- and (-)-5-7

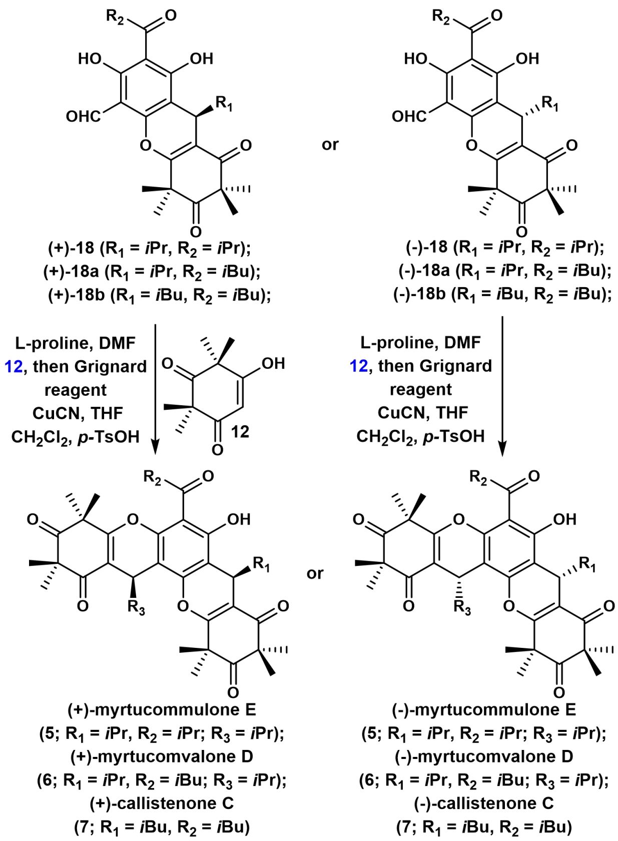

Supplementary Figure 76. Synthesis of (+)- and (-)-5-7.

To a solution of (+)-**18** or (+)-**18a-18b** or (-)-**18** or (-)-**18a-18b** (1.0 equiv.) in dimethyl formamide (DMF) under argon ere added L-proline (1.0 equiv.) and **12**. The resulting mixture was stirred for 12 h at 25 ºC. The mixture was poured into hexane and directly filtered, then concentrated *in vacuo* to afford crude.

To a solution of the crude in tetrahydrofuran-dichloromethane (THF-CH_2Cl_2; v/v =1:1; 0.05M) under argon were added copper(I) cyanide (CuCN, 1.1 equiv.) and Grignard reagent (*i*PrMgBr or *i*BuMgBr; 3.5 equiv.) at −78 °C. The resulting mixture was allowed to warm up to −50 °C. After the reaction was finished according to TLC, the water (4.0 equiv.) and *p*-TsOH (3.0 equiv.) were added. The resulting mixture was allowed to warm up to 40 °C and stirred for 2 h. The mixture was quenched with saturated aqueous sodium bicarbonate, and extracted with ethyl acetate. The combined organic layers were dried over Na_2SO_4, filtered and concentrated *in vacuo*. The residue was purified by flash-column chromatography on silica gel (1%-4% ethyl acetate/*n*-hexane) to

afford the corresponding products (+)- and (-)-**5**-**7**.

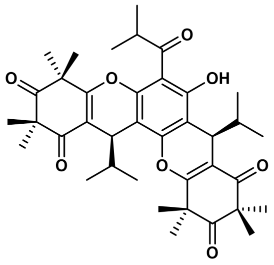

(+)-myrtucommulone E (5)

Compound (+)-myrtucommulone E (**5**): 99.5 mg, 58% yield, 5 h (120 mg scale), white solids; **[*α*]** = +158.1 (*c* = 0.3 in CH_2Cl_2); Compound (-)-myrtucommulone E (**5**): 99.5 mg, 58% yield, 5 h (120 mg scale), white solids; $[\alpha]^{26}_{D}$ = −152.3 (*c* = 0.1 in CH_2Cl_2); **R_f** = 0.60 (ethyl acetate/*n*-hexane = 1/8); **IR (KBr)**: 3691, 2964, 2923, 2873, 1717, 1660, 1616, 1467, 1383, 1090 cm^{-1}; **^{1}H NMR** (400 MHz, $CDCl_3$) δ 13.26 (s, 1H), 4.39 (d, *J* = 3.3 Hz, 1H), 4.35 (d, *J* = 3.4 Hz, 1H), 3.90 (m, 1H), 2.03 (m, 1H), 1.91 (m, 1H), 1.63 (s, 3H), 1.60 (s, 3H), 1.48 (s, 3H), 1.42 (s, 3H), 1.41 (s, 3H), 1.40 (s, 3H), 1.36 (s, 3H), 1.33 (s, 3H), 1.27 (s, 3H), 1.25 (d, *J* = 6.9 Hz, 3H), 0.89 (d, *J* = 6.9 Hz, 3H), 0.79 (d, *J* = 7.0 Hz, 3H), 0.79 (d, *J* = 7.0 Hz, 3H), 0.77 (d, *J* = 6.9 Hz, 3H); **^{13}C NMR** (101 MHz, $CDCl_3$) δ 211.8, 211.5, 209.5, 197.7, 197.5, 167.7, 167.2, 161.0, 153.2, 151.0, 111.9, 111.7, 110.3, 106.2, 103.0, 56.2, 56.1, 47.5, 47.3, 40.2, 35.3, 34.3, 32.2, 31.9, 25.4, 25.2, 25.1, 25.0, 24.9 (2 carbons overlapped), 24.0, 23.9, 20.7, 19.7, 19.4, 18.5, 18.5, 17.7; **HRMS** (ESI-TOF) m/z: $[M+H]^+$ calcd. for $C_{38}H_{49}O_8$ 633.3422; found, 633.3407.

Supplementary Table 7. Compared NMR data [$CDCl_3$] between our synthetic myrtucommulone E (**5**) and the isolated natural product.[3]

position	^1H & ppm (J)			^{13}C & ppm		
	isolated (400M)	synthesized (400M)	error (iso. - syn.)	isolated (101M)	synthesized (101M)	error (iso. - syn.)
1	-	-	-	103.2	103.0	0.2
2	-	-	-	151.0	151.0	0
3	-	-	-	106.1	106.2	0.1
4-OH	-	13.26*	-	160.9	161.0	-0.1
5	-	-	-	110.3	110.3	0
6	-	-	-	153.3	153.2	0.1
7	4.38 (d, 3.3)	4.39 (d, 3.3)	-0.01	32.2	32.2	0
8	1.98 (m)	2.03 (m)	-0.05	35.4	35.3	0.1
9	0.81 (d, 3.6)	0.79 (d, 7.0)	0.02	18.5	18.5	0
10	0.94 (d, 6.9)	0.89 (d, 6.9)	0.05	19.4	19.4	0
1'	-	-	-	111.9	111.9	0
2'	-	-	-	197.6	197.7	-0.1
3'	-	-	-	56.2	56.2	0
4'	-	-	-	211.5	211.5	0
5'	-	-	-	47.2	47.3	0.1
6'	-	-	-	167.2	167.2	0
7'	-	-	-	209.5	209.5	0
8'	3.83 (m)	3.90 (m)	-0.07	40.2	40.2	0
9'	0.83 (d, 7.1)	0.79 (d, 7.0)	0.04	19.7	19.7	0
10'	1.26 (d, 6.0)	1.25 (d, 6.9)	0.01	17.7	17.7	0
11'	1.57 (s)	1.60 (s)	0.03	23.8	23.9	-0.1
12'	1.29 (s)	1.33 (s)	-0.04	25.2	25.2	0
13'	1.56 (s)	1.48 (s)	0.08	25.5	25.4	0.1
14'	1.40 (s)	1.40 (s)	0	24.9	24.9	0
1"	-	-	-	110.3	111.7	1.4
2"	-	-	-	197.4	197.5	-0.1
3"	-	-	-	56.1	56.1	0
4"	-	-	-	211.5	211.8	-0.3
5"	-	-	-	47.5	47.5	0
6"	-	-	-	167.2	167.7	-0.5
7"	4.40 (d, 3.4)	4.35 (d, 3.4)	0.05	31.9	31.9	0
8"	1.95 (m)	1.91 (m)	0.04	34.3	34.3	0
9"	1.26 (d, 6.0)	1.27 (d, 7.0)	-0.01	20.7	20.7	0
10"	0.79 (d, 6.9)	0.77 (d, 6.9)	0.02	18.5	18.5	0
11"	1.41 (s)	1.41 (s)	0	24.9	24.9	0
12"	1.32 (s)	1.36 (s)	-0.04	23.9	24.0	0
13"	1.77 (s)	1.42 (s)	0.35	25.1	25.1	0
14"	1.64 (s)	1.63 (s)	0.01	25.0	25.0	0

Note: (1) The structure of synthetic **5** was confirmed by X-ray diffraction analysis. The ^{1}H and ^{13}C NMR data of the synthesized **5** was assigned by ^{1}H-^{1}H COSY, HSQC and HMBC spectra. Thus, the NMR data of H13″ and ^{13}C NMR data of C1″, C4″, C6″ reported by Shaheen, F. and co-workers[3] needs to be revised slightly, respectively (110.3, 211.5 and 167.2 were listed two times in the reference by mistake).

(2) According to the above procedure, a total of 190 mg of compounds (+)-**5** was prepared readily after 2 simple parallel operations.

(3) We expected that treatment of (+)-**5** with NaOH or KOH could give (+)-**1** (Supplementary Figure 77). Disappointedly, **1** and **28** would not be obtained, but an undesired **27**. The reason was probably that the double Michael addition reaction proceeded under NaOH or KOH condition, but then this reaction was prone to proceed to a *retro*-hemiketalization reaction to give ring-opened **27**.

Therefore, conversion of (+)-**5** to alcohol (+)-**1** was nontrivial by this strategy.

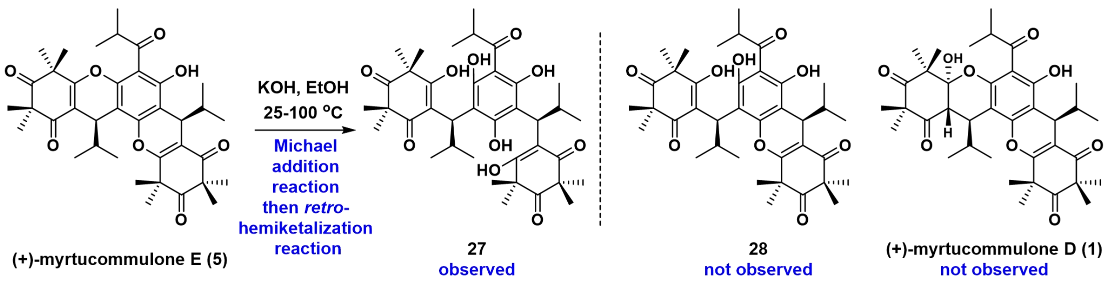

Supplementary Figure 77. Attempts to asymmetric syntheses of 1.

Note: The ^{1}H spectra was complex, which was because of the presence of rotamers and keto-enol tautomers.

Compound **27**

$[\alpha]_{D}^{26}$ = +36.5 (c = 0.1 in MeOH); **R$_f$** = 0.30 (ethyl acetate/n-hexane = 1/3); **IR (KBr):** 3161, 2970, 2934, 2872, 1719, 1616, 1468, 1385, 1261, 1184, 1016 cm^{-1}; **HRMS** (ESI-TOF) m/z: [M+H]$^{+}$ calcd. for $C_{38}H_{53}O_{10}$ 669.3633; found, 669.3623.

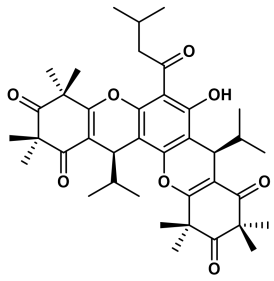

(+)-myrtucomvalone D (6)

Compound (+)-myrtucomvalone D (**6**): 106.0 mg, 58.5% yield, 5 h (120 mg scale), white solids; $[\alpha]_{D}^{26}$ = +147.6 (c = 0.3 in CH_2Cl_2); Compound (-)-myrtucomvalone D (**6**): 109.6 mg, 60.5% yield, 5 h (120 mg scale), white solids; $[\alpha]_{D}^{26}$ = −138.9 (c = 0.1 in CH_2Cl_2); **R$_f$** = 0.61 (ethyl acetate/n-hexane = 1/8); **IR (KBr)**: 3176, 2958, 2873, 1703, 1613, 1467, 1367, 1264, 1120, 1001 cm^{-1}; **^{1}H NMR** (400 MHz, $CDCl_3$) δ 13.43 (s, 1H), 4.40 (d, J = 3.3 Hz, 1H), 4.35 (d, J = 3.4 Hz, 1H), 3.20 (dd, J = 17.1, 7.3 Hz, 1H), 2.98 (dd, J = 17.1, 6.3 Hz, 1H), 2.36 (m, 1H), 2.04 (m, 1H), 1.91 (m, 1H), 1.66 (s, 3H), 1.64 (s, 3H), 1.48 (s, 3H), 1.44 (s, 3H), 1.43 (s, 3H), 1.42 (s, 3H), 1.36

(s, 3H), 1.34 (s, 3H), 1.02 (d, J = 7.4 Hz, 3H), 1.00 (d, J = 8.4 Hz, 3H), 0.90 (d, J = 6.8 Hz, 3H), 0.81 (d, J = 6.8 Hz, 3H), 0.80 (d, J = 6.5 Hz, 3H), 0.78 (d, J = 6.5 Hz, 3H); **^{13}C NMR** (101 MHz, $CDCl_3$) δ 212.0, 211.7, 204.8, 197.8, 197.6, 167.8, 167.4, 160.9, 153.5, 151.6, 112.1, 111.9, 110.2, 107.8, 103.3, 56.4, 56.3, 54.0, 47.7, 47.5, 35.6, 34.5, 32.3, 32.1, 25.6, 25.5, 25.4, 25.3, 25.2, 25.2, 24.9, 24.2, 24.0, 23.0, 22.9, 19.8, 19.6, 18.8 (2 carbons overlapped); **HRMS** (ESI-TOF) m/z: $[M-H]^-$ calcd. for $C_{39}H_{51}O_9$ 663.3533; found, 663.3538.

Supplementary Table 8. Compared NMR data [$CDCl_3$] between our synthetic myrtucomvalone D (**6**) and the isolated natural product.[7]

	^{1}H & ppm (J)			^{13}C & ppm		
position	isolated (400M)	synthesized (400M)	error (iso. - syn.)	isolated (101M)	synthesized (101M)	error (iso. - syn.)
1	-	-	-	103.3	103.3	0
2	-	-	-	151.5	151.6	-0.1
3	-	-	-	107.8	107.8	0
4-OH	13.44*	13.43*	0.01	160.8	160.9	-0.1
5	-	-	-	110.2	110.2	0
6	-	-	-	153.5	153.5	0
7	4.36 (d, 3.6)	4.35 (d, 3.4)	0.01	32.0	32.1	0
8	1.89-1.95 (m)	1.91 (m)	-	35.5	35.6	-0.1
9	0.81 (d, 6.6)	0.80 (d, 6.5)	0.01	18.7	18.8	-0.1
10	0.91 (d, 7.2)	0.90 (d, 6.8)	0.01	19.5	19.6	-0.1
1'				111.9	111.9	0
2'	-	-	-	197.6	197.6	0
3'	-	-	-	56.4	56.3	-0.1
4'	-	-	-	211.7	211.7	0
5'	-	-	-	47.5	47.5	0
6'	-	-	-	167.4	167.4	0
7'	-	-	-	204.8	204.8	0
8'	3.21 (dd,16.8, 7.2)	3.20 (dd,17.1, 7.3)	0.01	54.0	54.0	0
	2.99 (dd,16.8, 6.6)	2.98 (dd,17.1, 6.3)	0.01			
9'	2.34-2.42 (m)	2.36 (m)	-	25.1	25.2	-0.1
10'	1.04 (d, 6.6)	1.02 (d,7.4)	0.02	23.0	23.0	0
11'	1.02 (d, 6.6)	1.00 (d, 8.4)	0.02	22.8	22.9	-0.1
12'	1.44 (s)	1.43 (s)	0.01	25.2	25.2	0
13'	1.36 (s)	1.34 (s)	0.02	24.0	24.0	0
14'	1.50 (s)	1.48 (s)	0.02	24.9	24.9	0
15'	1.65 (s)	1.64 (s)	0.01	25.3	25.3	0
1''	-	-	-	112.1	112.1	0
2''	-	-	-	197.8	197.8	0
3''	-	-	-	56.3	56.4	-0.1
4''	-	-	-	212.0	212.0	0
5''	-	-	-	47.6	47.7	-0.1
6''	-	-	-	167.8	167.8	0
7''	4.41 (d, 3.6)	4.40 (d, 3.3)	0.01	32.3	32.3	0
8''	2.03-2.09 (m)	2.04 (m)	-	34.5	34.5	0
9''	0.82 (d, 7.2)	0.81 (d, 6.8)	0.01	19.8	19.8	0
10''	0.80 (d, 7.2)	0.78 (d, 6.5)	0.01	18.7	18.8	-0.1
11''	1.44 (s)	1.42 (s)	0.02	25.1	25.2	-0.1
12''	1.38 (s)	1.36 (s)	0.02	24.1	24.2	-0.1
13''	1.67 (s)	1.66 (s)	0.01	25.6	25.6	0
14''	1.45 (s)	1.44 (s)	0.01	25.4	25.4	0

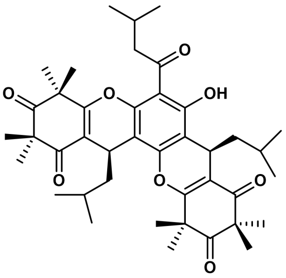

(+)-callistenone C (7)

Compound (+)-callistenone C (**7**): 57.6 mg, 54% yield, 5 h (70 mg scale), white solids; $[\alpha]_D^{26}$ = +141.8 (c = 0.1 in CH_2Cl_2); Compound (-)-callistenone C (**7**): 54.4 mg, 51% yield, 5 h (70 mg scale), white solids; $[\alpha]_D^{26}$ = −129.3 (c = 0.1 in CH_2Cl_2); **R*f*** = 0.53 (ethyl acetate/*n*-hexane = 1/10); **IR (KBr)**: 3461, 2955, 2871, 1722, 1660, 1620, 1464, 1381, 1285, 1156, 1038 cm^{-1}; **^{1}H NMR** (400 MHz, $CDCl_3$) δ 13.46 (s, 1H), 4.36 (t, *J* = 5.8 Hz, 1H), 4.32 (t, *J* = 5.4 Hz, 1H), 3.20 (dd, *J* = 17.3, 7.2 Hz, 1H), 2.99 (dd, *J* = 17.3, 6.3 Hz, 1H), 2.37 (m, 1H), 1.63 (s, 3H), 1.61 (s, 3H), 1.50 (m, 2H), 1.49 (s, 3H), 1.48 (m, 2H), 1.45 (s, 3H), 1.41 (s, 3H), 1.39 (s, 3H), 1.37 (s, 3H), 1.33 (s, 3H), 1.02 (d, *J* = 5.2, 3H), 1.01 (d, *J* = 5.1, 3H), 0.95 (d, *J* = 6.2 Hz, 3H), 0.87 (d, *J* = 5.8 Hz, 3H), 0.81 (d, *J* = 6.0 Hz, 3H), 0.80 (d, *J* = 5.8 Hz, 3H); **^{13}C NMR** (101 MHz, $CDCl_3$) δ 211.7, 211.5, 204.7, 197.6, 197.5, 166.9, 166.8, 160.7, 152.5, 150.6, 114.4, 113.6, 111.8, 107.7, 105.8, 56.3, 56.1, 54.0, 47.4, 47.4, 46.9, 45.6, 25.7, 25.5, 25.4, 25.3, 25.2, 25.2, 25.1, 25.0, 24.8, 24.6, 24.5, 24.3, 23.9, 23.9, 23.5, 23.4, 23.4, 22.9, 22.8; **HRMS** (ESI-TOF) m/z: $[M+H]^+$ calcd. for $C_{41}H_{55}O_8$ 675.3891; found, 675.3888.

Supplementary Table 9. Compared NMR data [$CDCl_3$] between our synthetic callistenone C (**7**) and the isolated natural product reported by Mahabusarakam, W. and co-workers.[8]

	^1H & ppm (J)			*^{13}C & ppm*		
position	isolated (600M)	synthesized (400M)	error (iso. - syn.)	isolated (151M)	synthesized (101M)	error (iso. - syn.)
1	-	-	-	105.7	105.8	-0.1
2	-	-	-	150.5	150.6	-0.1
3	-	-	-	107.6	107.7	-0.1
4	13.50*(s)	13.46*(s)	0.04	160.6	160.7	-0.1
5	-	-	-	107.8	111.8	-4
6	-	-	-	152.4	152.5	-0.1
7	4.39 (t, 5.4)	4.36 (t, 5.8)	0.03	25.2	25.3	-0.1
8	1.50 (m)	1.50 (m)	0	46.8	46.9	-0.1
9	1.50 (m)	1.50 (m)	0	25.0	25.2	-0.2
10	0.84 (d, 6.0)	0.81 (d, 6.0)	0.03	23.4	23.5	-0.1
11	0.98 (d, 6.0)	0.95 (d, 6.2)	0.03	23.8	23.9	-0.1
1'	-	-	-	113.5	113.6	-0.1
2'	-	-	-	197.6	197.6	0
3'	-	-	-	56.2	56.3	-0.1
4'	-	-	-	211.4	211.5	-0.1
5'	-	-	-	47.3	47.4	-0.1
6'	-	-		166.8	166.9	-0.1
7'	-	-	-	24.2	24.3	-0.1
8'	3.23 (dd, 17.4,6.6)	3.20 (dd, 17.3,7.2)	0.03	204.6	204.7	-0.1
	2.99 (dd, 17.4,6.6)	2.99 (dd, 17.6,6.3)	0	53.9	54.0	-0.1
9'	2.40 (m)	2.37 (m)	0.03			
10'	1.05 (d, 6.6)	1.02 (d, 5.2)	0.03	24.5	24.6	-0.1
11'	1.04 (d, 6.6)	1.00 (d, 5.1)	0.04	22.8	22.9	-0.1
12'	1.44 (s)	1.41 (s)	0.03	22.6	22.8	-0.1
13'	1.42 (s)	1.39 (s)	0.03	23.8	23.9	-0.1
14'	1.66 (s)	1.63 (s)	0.03	25.3	25.4	-0.1
15'	1.52 (s)	1.49 (s)	0.03	24.9	25.0	-0.1
1"	-	-	-	114.3	114.4	-0.1
2"	-	-	-	197.4	197.5	-0.1
3"	-	-	-	56.0	56.1	-0.1
4"	-	-	-	211.6	211.7	-0.1
5"	-	-	-	47.2	47.4	-0.2
6"	-	-	-	166.7	166.8	-0.1
7"	4.35 (t, 5.7)	4.32 (t, 5.4)	-0.03	25.6	25.7	-0.1
8"	1.50 (m)	1.48 (m)	0.02	45.4	45.6	-0.2
9"	1.50 (m)	1.48 (m)	002	25.3	25.5	-0.2
10"	0.83 (d, 6.0)	0.80 (d, 5.8)	0.03	23.3	23.4	-0.1
11"	0.90 (d, 6.0)	0.87 (d, 5.8)	0.03	23.3	23.4	-0.1
12"	1.40 (s)	1.37 (s)	0.03	24.9	25.1	-0.2
13"	1.37 (s)	1.33 (s)	0.04	24.4	24.5	-0.1
14"	1.64 (s)	1.61 (s)	0.03	24.7	24.8	-0.1
15"	1.48 (s)	1.45 (s)	0.03	25.0	25.2	-0.2

Supplementary Table 10. Compared NMR data [$CDCl_3$] between our synthetic callistenone C (**7**) and the isolated natural product reported by Wang, J.[6]

	^{1}H & ppm (J)			^{13}C & ppm		
position	isolated (600M)	synthesized (400M)	error (iso. - syn.)	isolated (151M)	synthesized (101M)	error (iso. - syn.)
1	-	-	-	105.8	105.8	0
2	-	-	-	150.6	150.6	0
3	-	-	-	107.7	107.7	0
4	13.46*(s)	13.46*(s)	0	160.7	160.7	0
5	-	-	-	111.8	111.8	0
6	-	-	-	152.5	152.5	0
7	4.37 (t, 5.6)	4.36 (t, 5.8)	0.01	25.3	25.3	0
8	1.50 (m)	1.50 (m)	0	46.9	46.9	0
9	1.50 (m)	1.50 (m)	0	25.1	25.2	-0.1
10	0.82 (d, 6.4)	0.81 (d, 6.0)	0.01	23.5	23.5	0
11	0.95 (d, 6.4)	0.95 (d, 6.2)	0	23.9	23.9	0
1'	-	-	-	204.7	204.7	0
2'	-	-	-	113.6	113.6	0
3'	-	-	-	197.6	197.6	0
4'	-	-	-	56.3	56.3	0
5'	-	-	-	211.5	211.5	0
6'	-	-	-	47.3	47.4	-0.1
7'	-	-	-	166.9	166.9	0
8'	3.20 (dd, 17.6,7.2)	3.20 (dd, 17.3,7.2)	0	54.0	54.0	0
	2.99 (dd, 17.6,7.2)	2.99 (dd, 17.6,6.3)				
			0			
9'	2.37 (m)	2.37 (m)	0	24.6	24.6	0
10'	1.03 (d, 5.2)	1.02 (d, 5.2)	0.01	22.9	22.9	0
11'	1.01 (d, 5.2)	1.00 (d, 5.1)	0.01	22.7	22.8	-0.1
12'	1.42 (s)	1.41 (s)	0.01	24.3	24.3	0
13'	1.40 (s)	1.39 (s)	0.01	23.9	23.9	0
14'	1.63 (s)	1.63 (s)	0	25.4	25.4	0
15'	1.50 (s)	1.49 (s)	0.01	25.0	25.0	0
1"	-	-	-	114.4	114.4	0
2"	-	-	-	197.5	197.5	0
3"	-	-	-	56.1	56.1	0
4"	-	-	-	211.7	211.7	0
5"	-	-	-	47.4	47.4	0
6"	-	-	-	166.9	166.8	0
7"	4.31 (t, 5.2)	4.32 (t, 5.4)	-0.01	25.7	25.7	0
8"	1.48 (m)	1.48 (m)	0.	45.6	45.6	0
9"	1.48 (m)	1.48 (m)	0	25.5	25.5	0
10"	0.81 (d, 6.4)	0.80 (d, 5.8)	0.01	23.4	23.4	0
11"	0.88 (d, 6.4)	0.87 (d, 5.8)	0.01	23.4	23.4	0
12"	1.37 (s)	1.37 (s)	0	25.0	25.1	-0.1
13"	1.34 (s)	1.33 (s)	0.01	24.5	24.5	0
14"	1.61 (s)	1.61 (s)	0	24.8	24.8	0
15"	1.46 (s)	1.45 (s)	0.01	25.1	25.2	-0.1

Note: Compared NMR data between our synthetic **7** and the isolated natural product reported by Mahabusarakam, W.[8] and co-workers as well as Wang, J.[6], respectively, they are the same compound. Thus, the ^{13}C NMR data of C5 reported by Mahabusarakam, W. and co-workers needs to be revised slightly.

3.8 X-ray crystal structures of (+)-1, 1a, (+)-2, (+)-5, *rac*-5a, (-)-8, (+)-14, (-)-14, 15, (-)-17n, (-)-17o

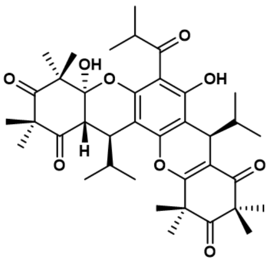

(+)-myrtucommulone D (1)

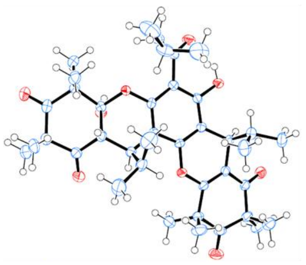

X-ray structure of 1
CCDC 2248854

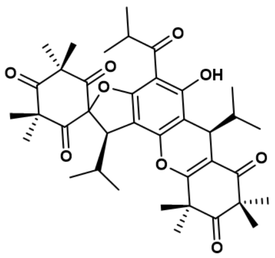

1a

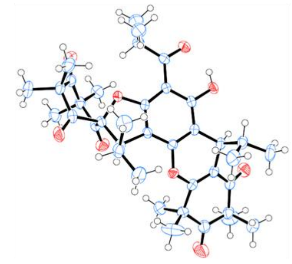

X-ray structure of 1a
CCDC 2248859

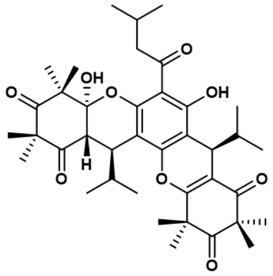

(+)-callistenone D (2)

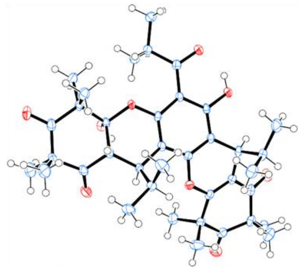

X-ray structure of 2
CCDC 2248856

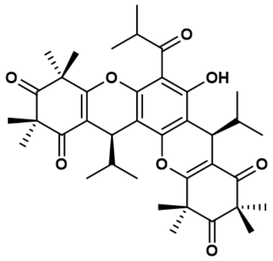

(+)-myrtucommulone E (5)

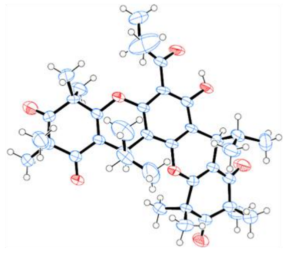

X-ray structure of 5
CCDC 2131874

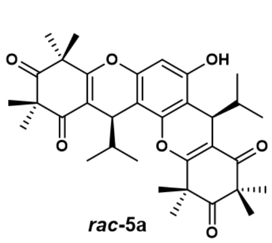

***rac*-5a**

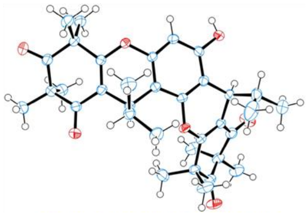

X-ray structure of *rac*-5a
CCDC 2248867

Supplementary Figure 78. X-ray crystal structures of (+)-1, 1a, (+)-2, (+)-5, *rac*-5a.

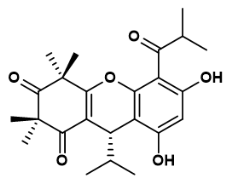

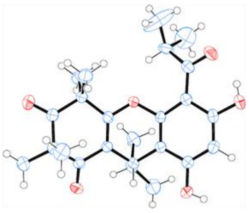

(-)-8

X-ray structure of (-)-8
CCDC 2248864

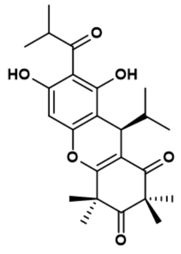

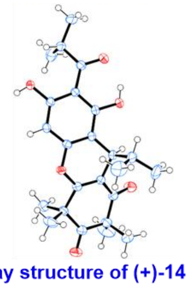

(+)-14

X-ray structure of (+)-14
CCDC 2248861

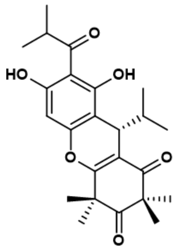

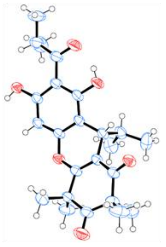

(-)-14

X-ray structure of (-)-14
CCDC 2248865

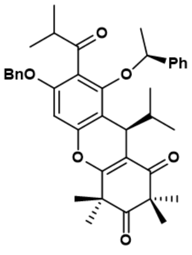

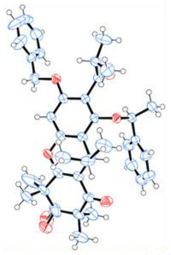

15

X-ray structure of 15
CCDC 2248863

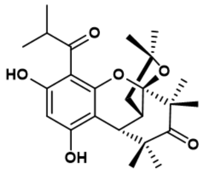

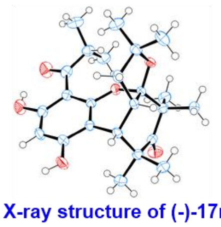

(-)-17n

X-ray structure of (-)-17n
CCDC 2248858

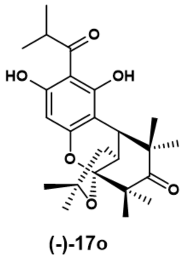

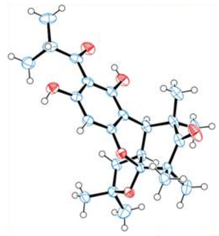

(-)-17o

X-ray structure of *ent*-17o
CCDC 2248866

Supplementary Figure 79. X-ray crystal structures of (-)-8, (+)-14, (-)-14, 15, (-)-17n, (-)-17o.

3.9 Computational details

In order to gain further insight into the origin of high diastereoselectivity of the the cascade reaction, density functional theory (DFT) calculations were carried out using Gaussian 16 software package.[9] Geometrical optimization was performed using the M06-2X[10] hybrid functional. For C, H, O and Br atoms, the 6-31G(d)[11] basis set was used, and the SDD[12] effective core potential basis set was used for Cu and Mg. Frequency analysis were carried out at the same level of theory. Intrinsic reaction coordinate (IRC) calculations were performed on the transition structures to either verify that they indeed connect to the starting and product complexes or to obtain suitable initial guess structure inputs for them. To obtain more accurate Gibbs free energies, single point energy calculations were performed with M06-2X functional and 6-311++G**[13] basis set. All the Gibbs energies shown in this article were calculated at 1 atm and 298.150 K.

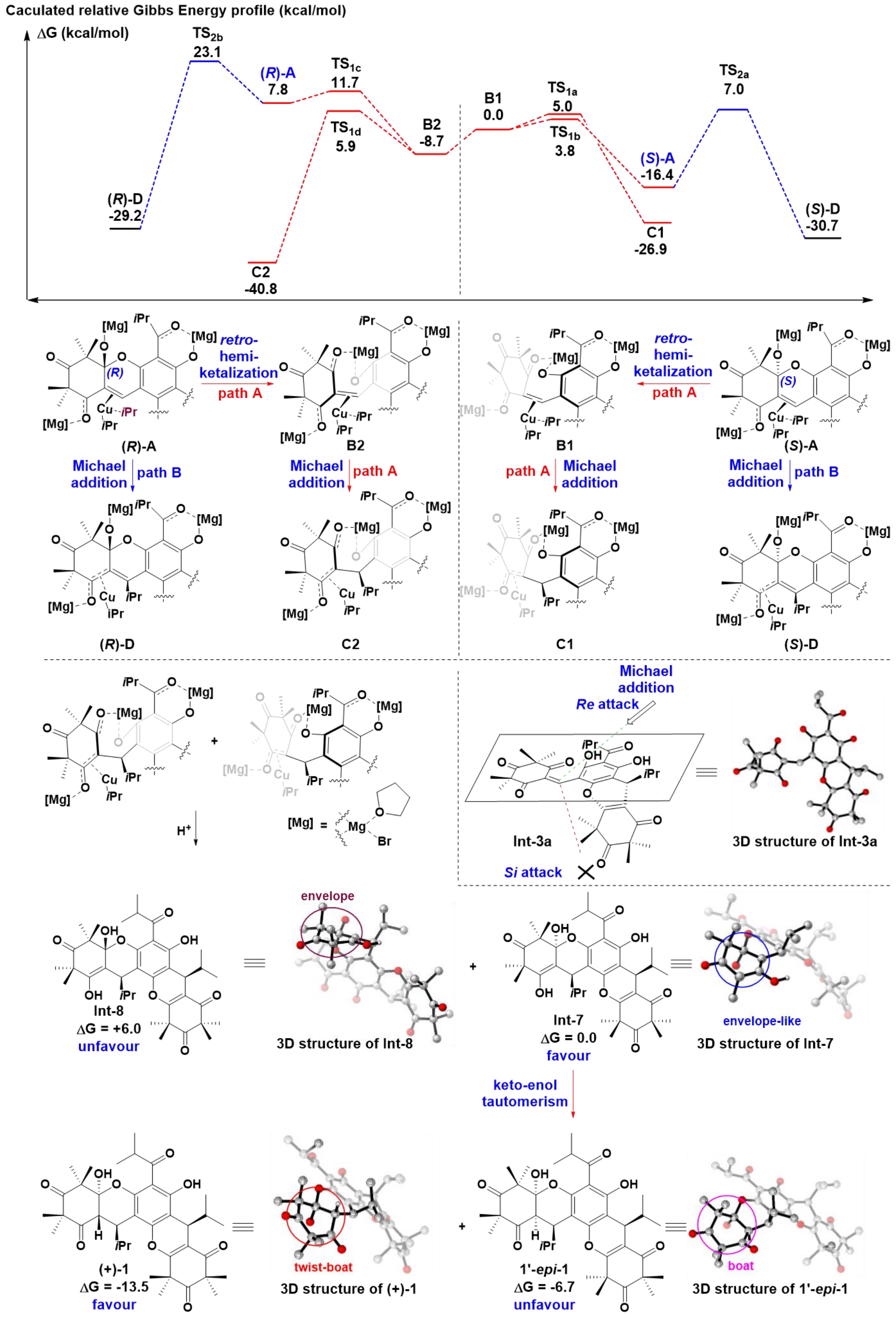

Supplementary Figure 80. The proposed and computational studies to understand mechanism of diastereoselectivity of the cascade reaction.

Note: (1) Since the Mg(II) and Cu(I) could probably play a crucial role in *retro*-hemiketalization and Michael addition reactions, the computational model was established in the Supplementary Figure 80.

(2) The *retro*-hemiketalization of (*R*)-**A** and (*S*)-**A** with a barrier of 3.9 and 20.2 kcal/mol provided **B2** and **B1**, respectively. However, Michael addition of (*R*)-**A** and (*S*)-**A** with a greater barrier of 15.3 and 23.4 kcal/mol gave (*R*)-**D** and (*S*)-**D**, respectively. Therefore, path A is more likely the pathway. Furthermore, Michael addition of **B2** and **B1** with moderate or small barrier of 14.6 and 5.0 kcal/mol afforded **C2** and **C1**, suggesting that this step will readily occur.

3.10 Synthetic ^{1}H, ^{13}C and 2D-NMR spectra

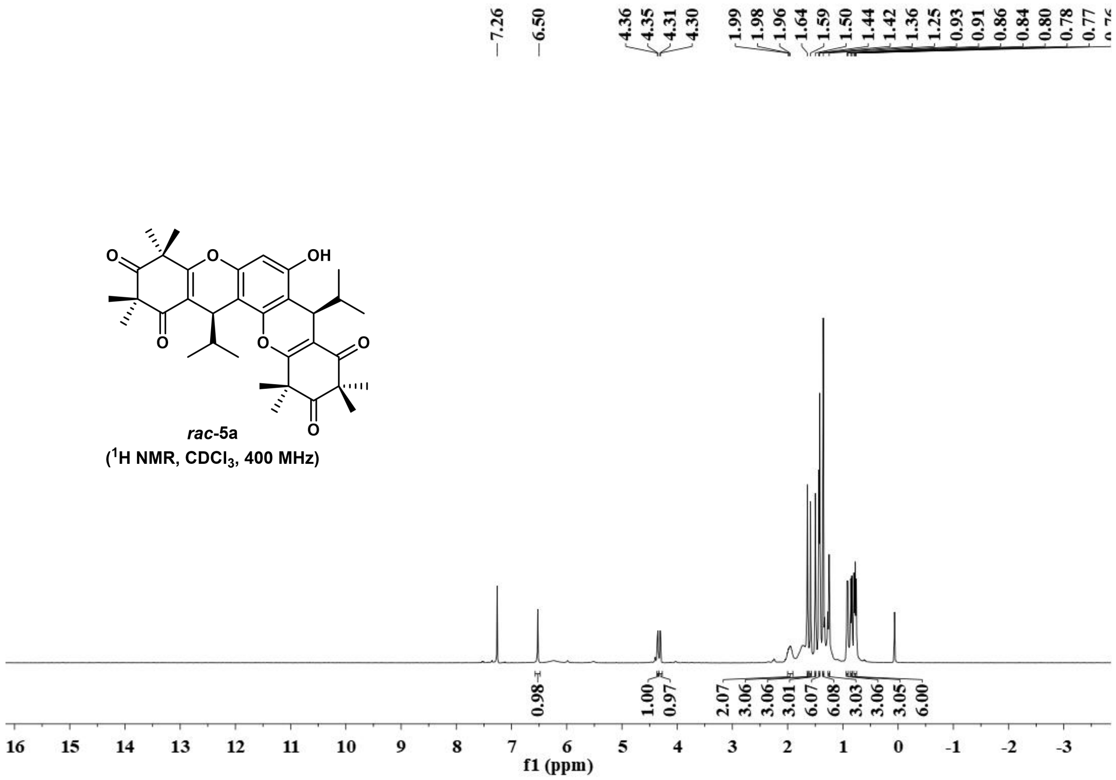

Supplementary Figure 81. ^{1}**H NMR spectra of** ***rac*****-5a.**

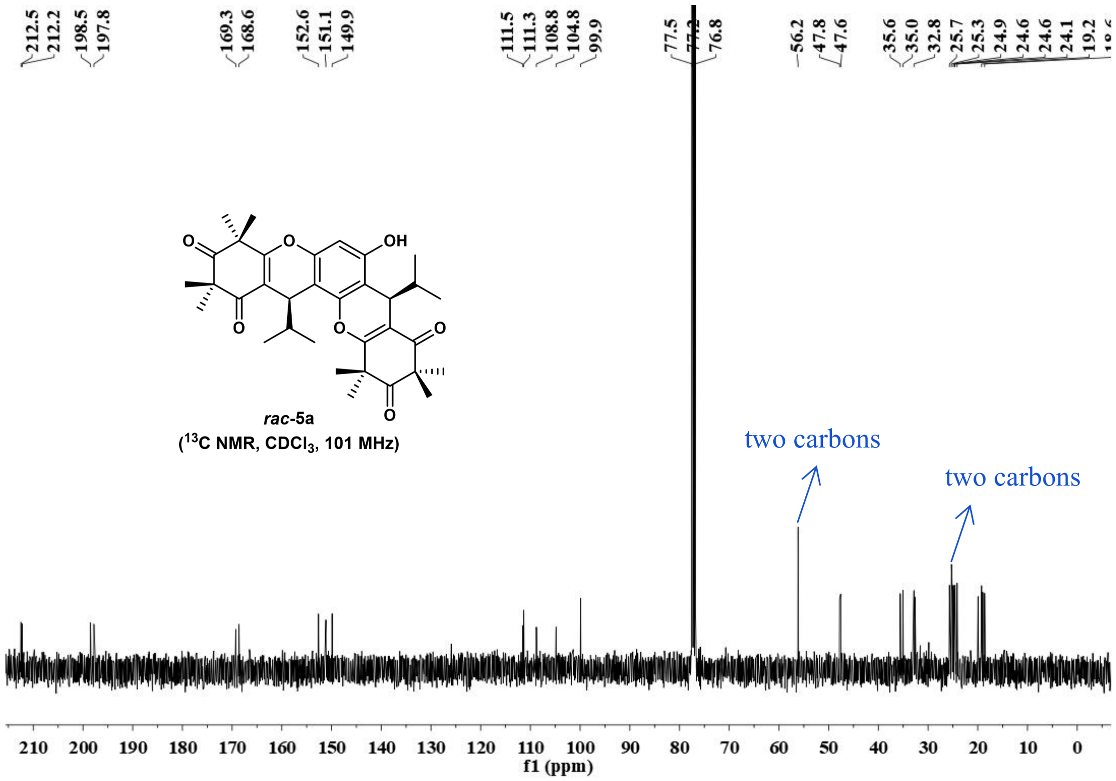

Supplementary Figure 82. ^{13}**C NMR spectra of** ***rac*****-5a.**

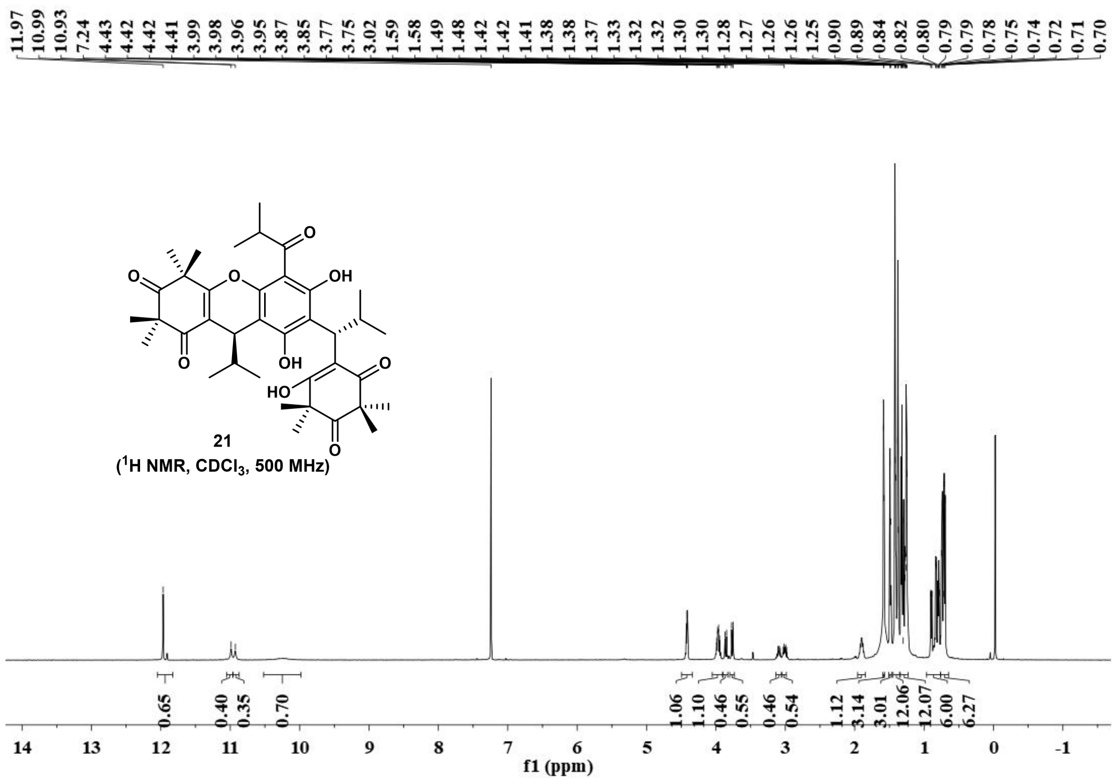

Supplementary Figure 83. ^{1}H NMR spectra of 21.

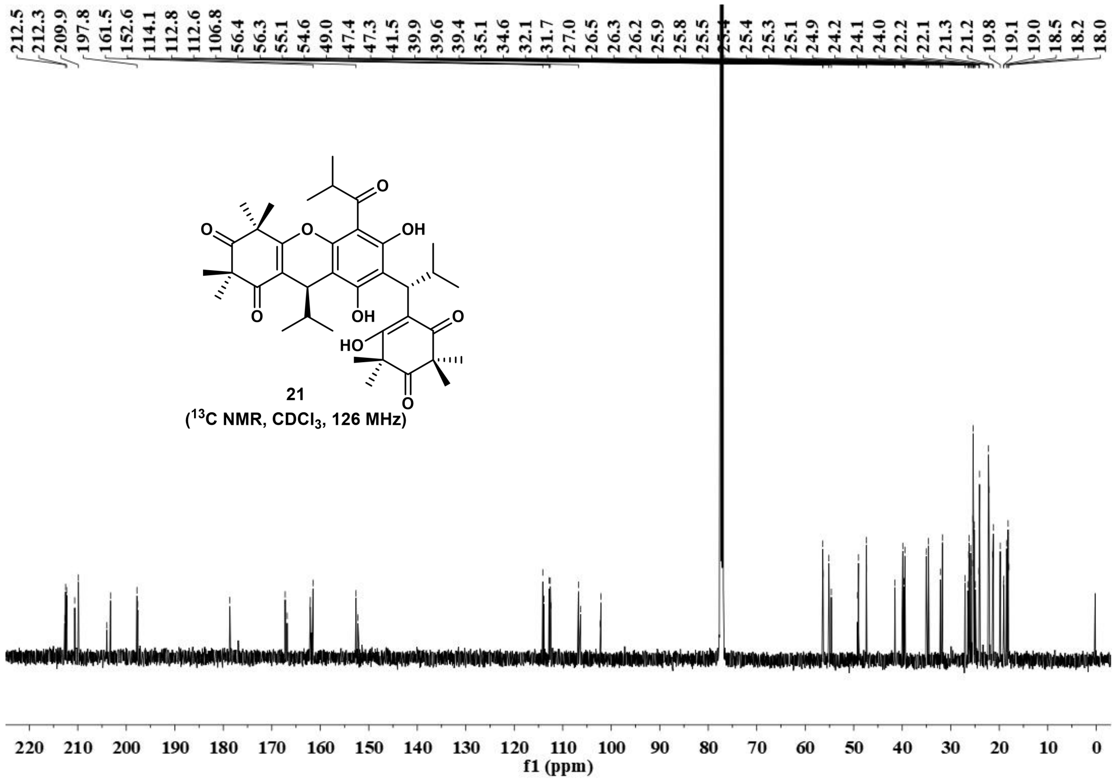

Supplementary Figure 84. ^{13}C NMR spectra of 21.

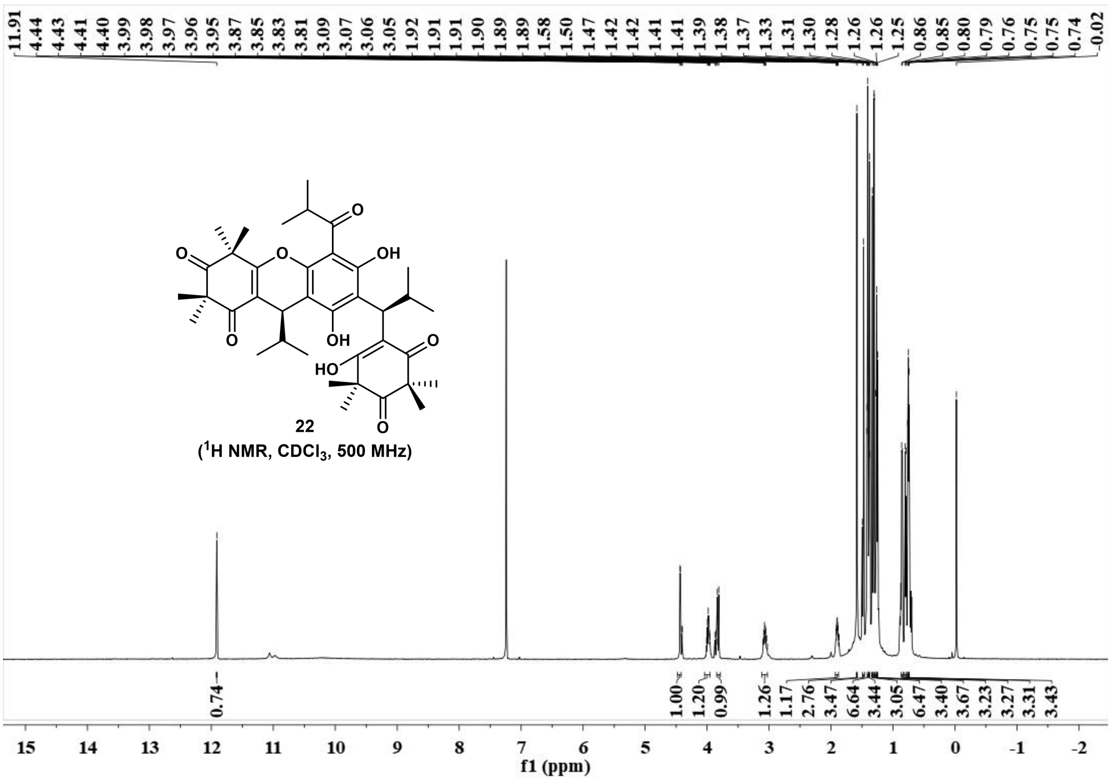

Supplementary Figure 85. ^{1}H NMR spectra of 22.

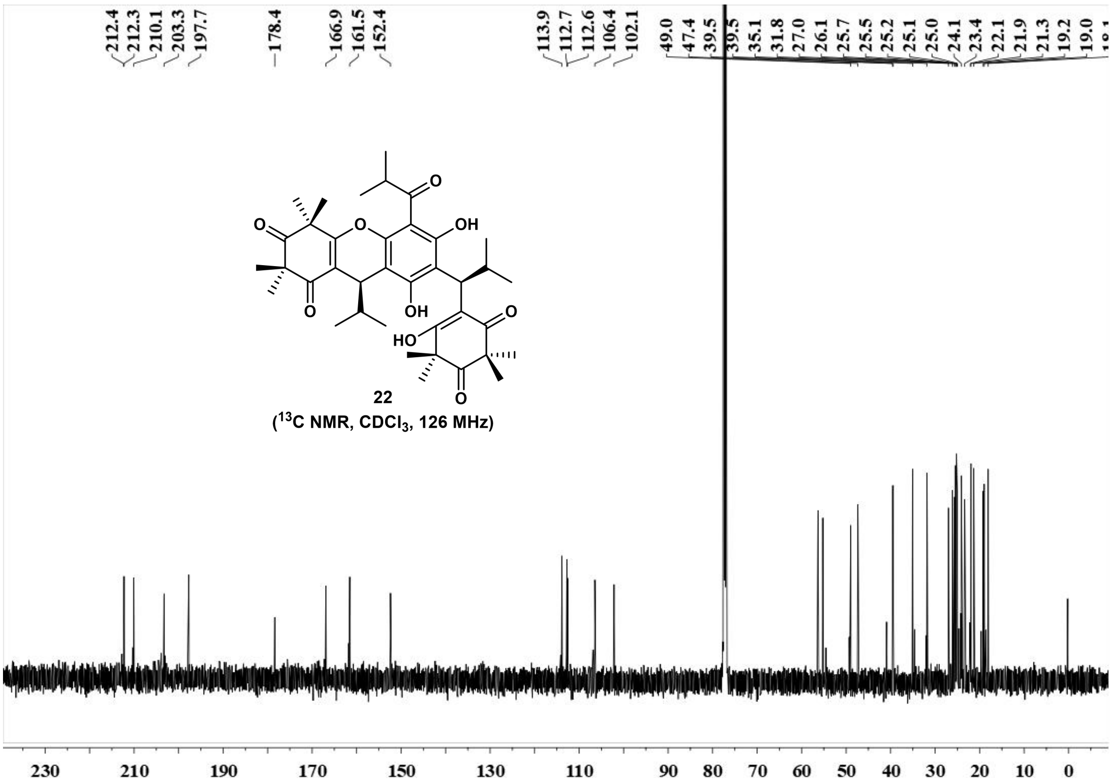

Supplementary Figure 86. ^{13}C NMR spectra of 22.

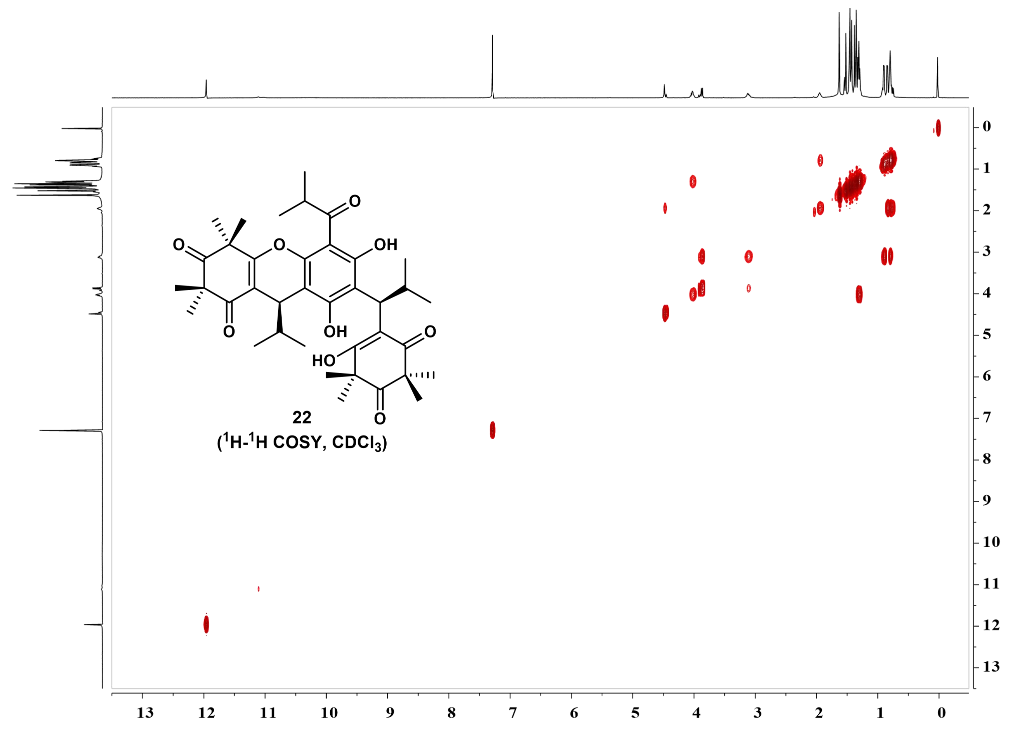

Supplementary Figure 87. ^{1}H-^{1}H COSY spectra of 22.

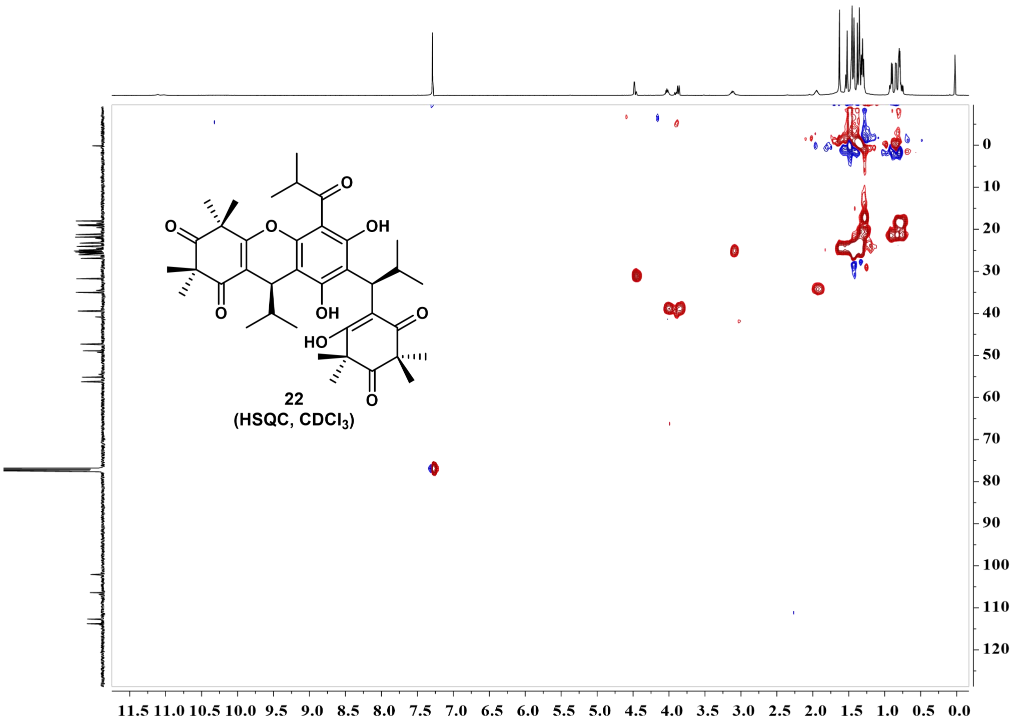

Supplementary Figure 88. HSQC spectra of 22.

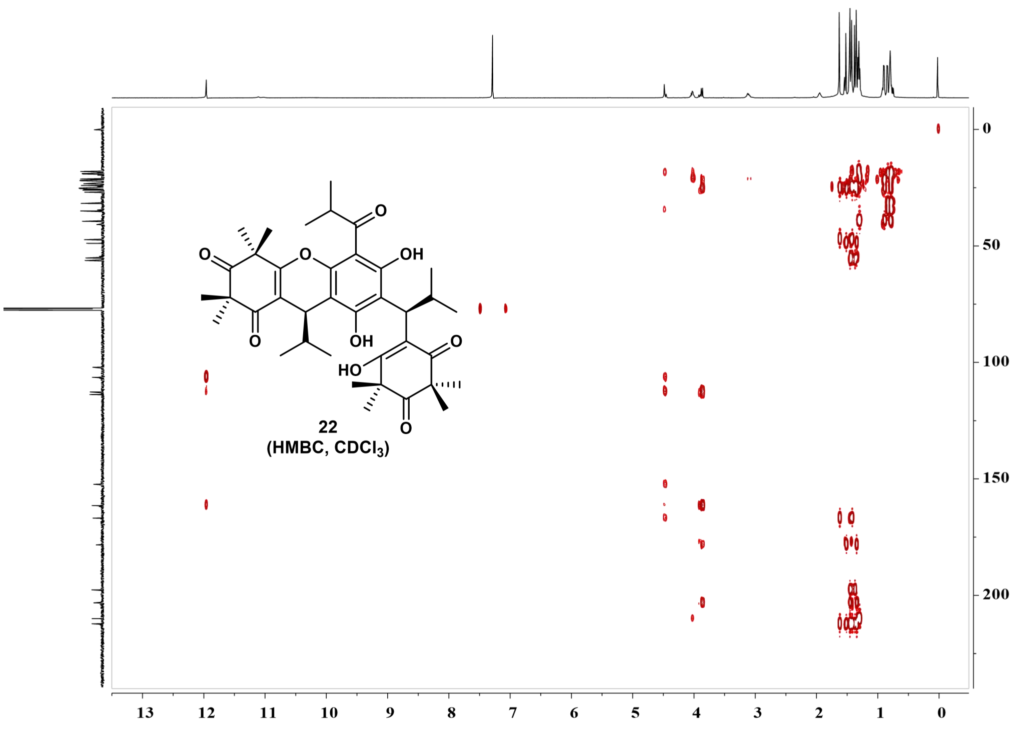

Supplementary Figure 89. HMBC spectra of 22.

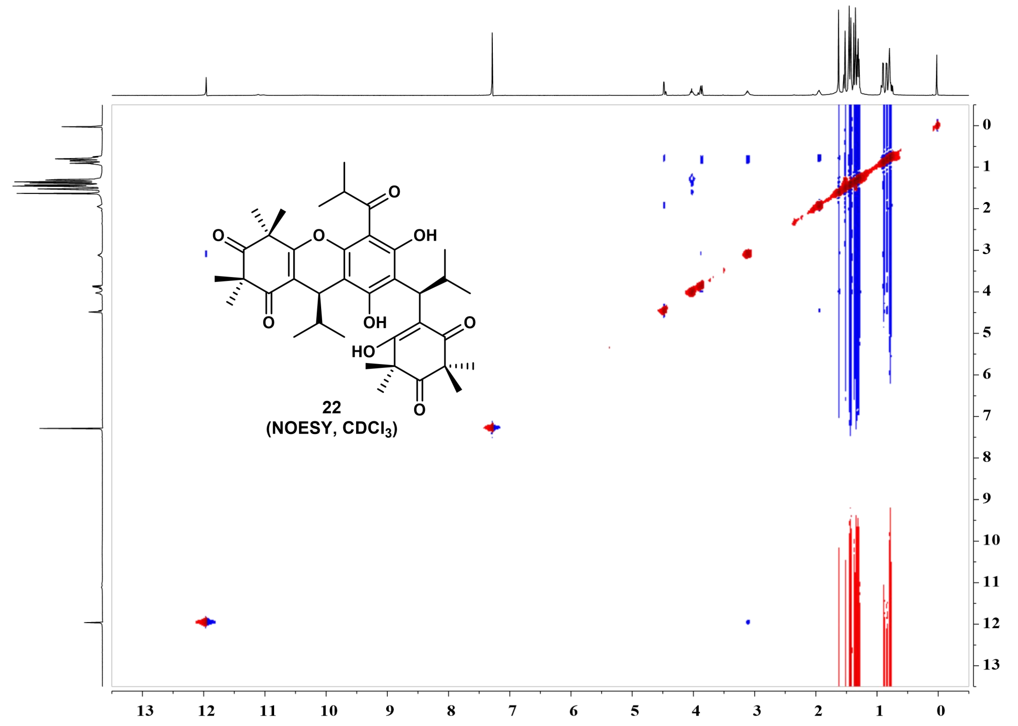

Supplementary Figure 90. NOESY spectra of 22.

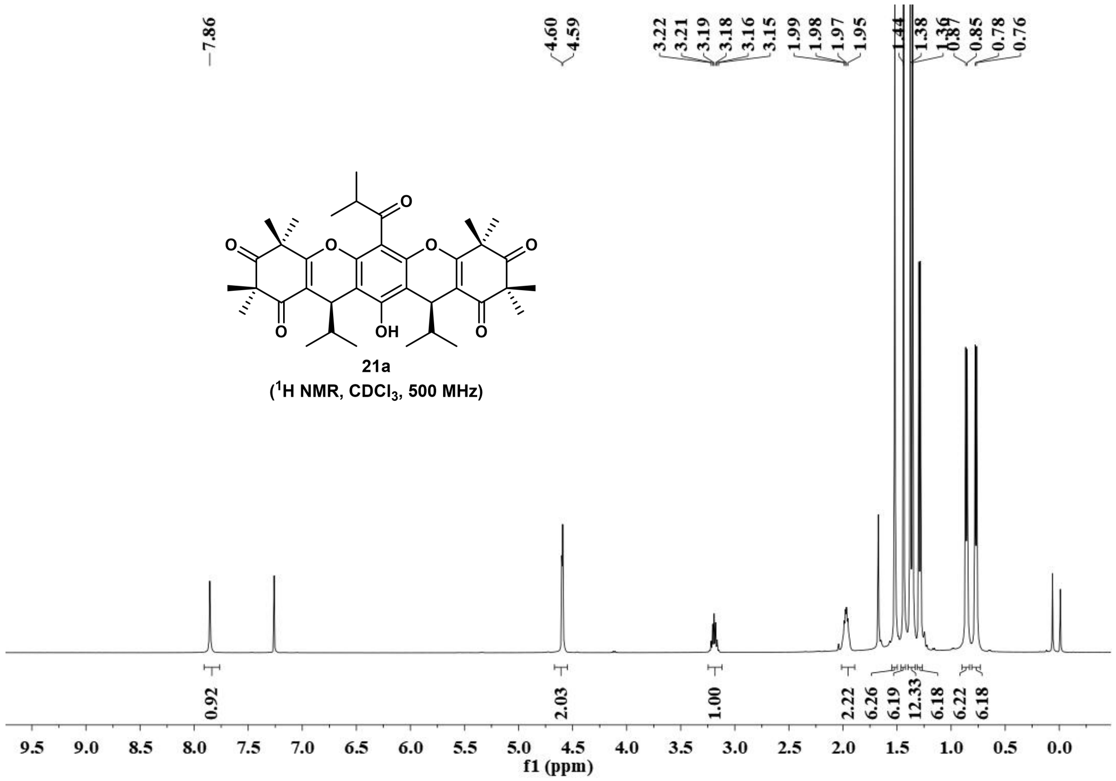

Supplementary Figure 91. ^{1}H NMR spectra of 21a.

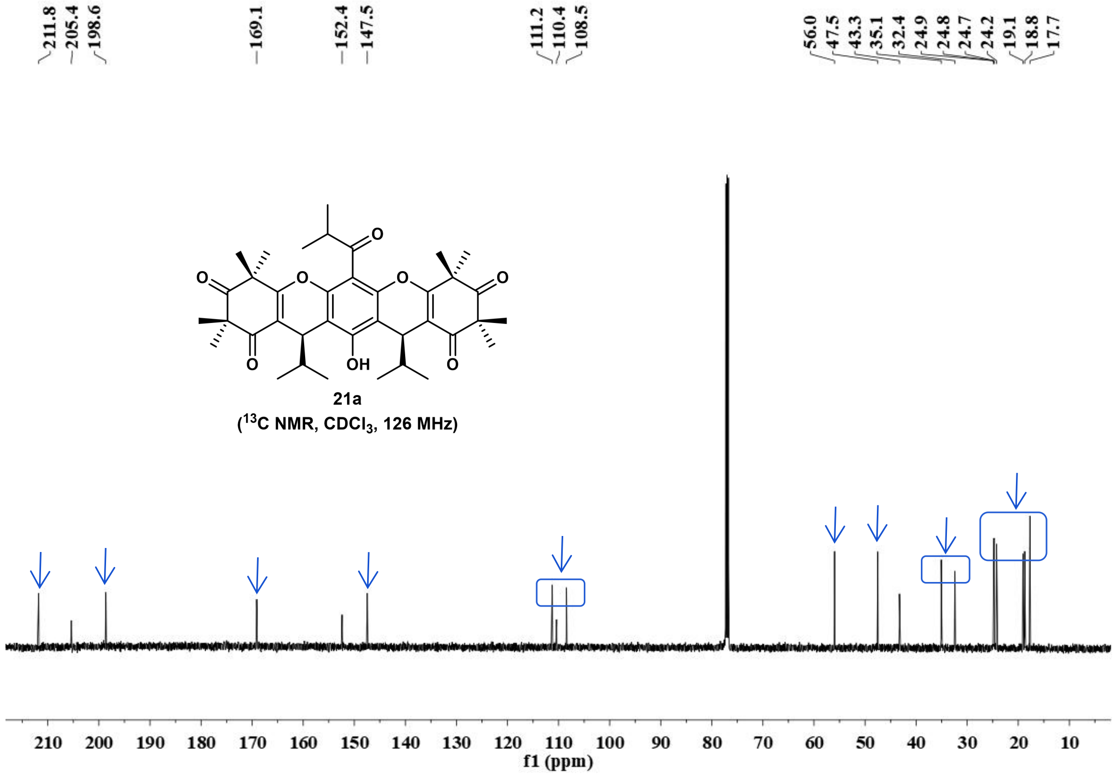

Supplementary Figure 92. ^{13}C NMR spectra of 21a.

Note: There are overlapping carbon signals including 211.8 (2 carbons overlapped), 198.6 (2

carbons overlapped), 169.1 (2 carbons overlapped), 147.5 (2 carbons overlapped), 111.2 (2 carbons overlapped), 108.5 (2 carbons overlapped), 56.0 (2 carbons overlapped), 47.5 (2 carbons overlapped), 35.1 (2 carbons overlapped), 32.4 (2 carbons overlapped), 24.9 (2 carbons overlapped), 24.8 (2 carbons overlapped), 24.7 (2 carbons overlapped), 24.2 (2 carbons overlapped), 19.1 (2 carbons overlapped), 18.8 (2 carbons overlapped), 17.7 (2 carbons overlapped).

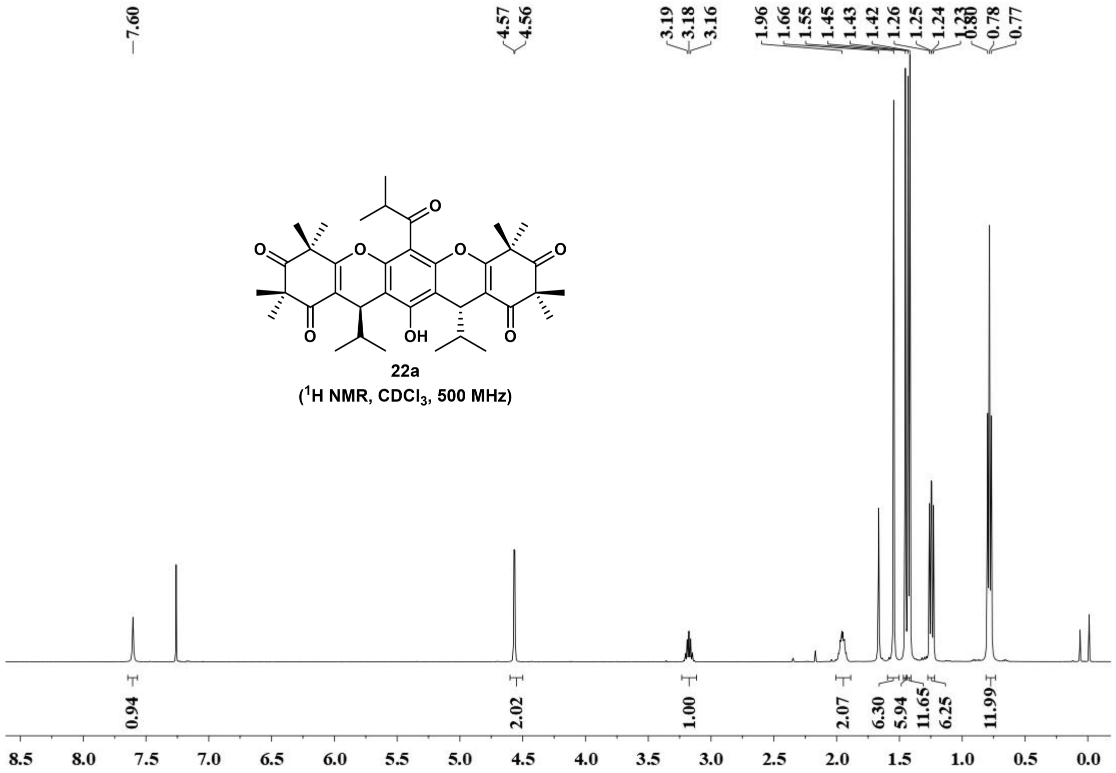

Supplementary Figure 93. ^{1}H NMR spectra of 22a.

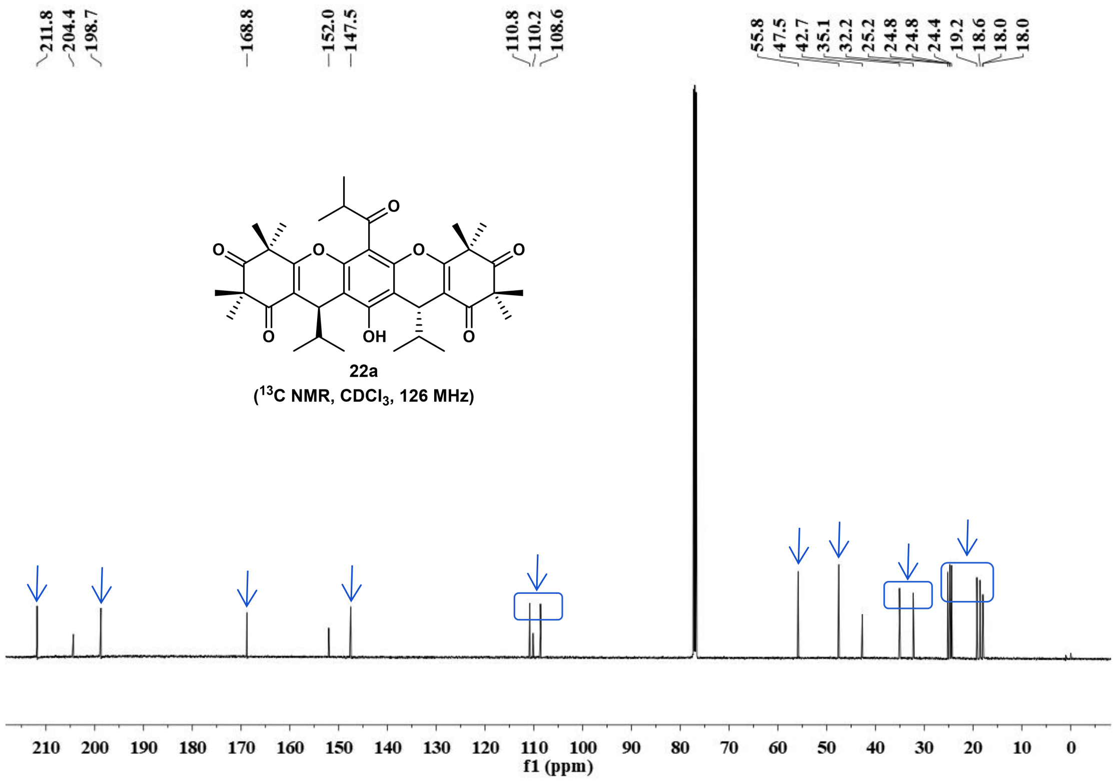

Supplementary Figure 94. ^{13}C NMR spectra of 22a.

Note: There are overlapping carbon signals including 211.8 (2 carbons overlapped), 198.7 (2 carbons overlapped), 168.8 (2 carbons overlapped), 147.5 (2 carbons overlapped), 110.8 (2 carbons overlapped), 108.6 (2 carbons overlapped), 55.8 (2 carbons overlapped), 47.5 (2 carbons overlapped), 35.7 (2 carbons overlapped), 32.2 (2 carbons overlapped), 25.2 (2 carbons overlapped), 24.8 (2 carbons overlapped), 24.8 (2 carbons overlapped), 24.4 (2 carbons overlapped), 19.2 (2 carbons overlapped), 18.6 (2 carbons overlapped), 18.0 (2 carbons overlapped).

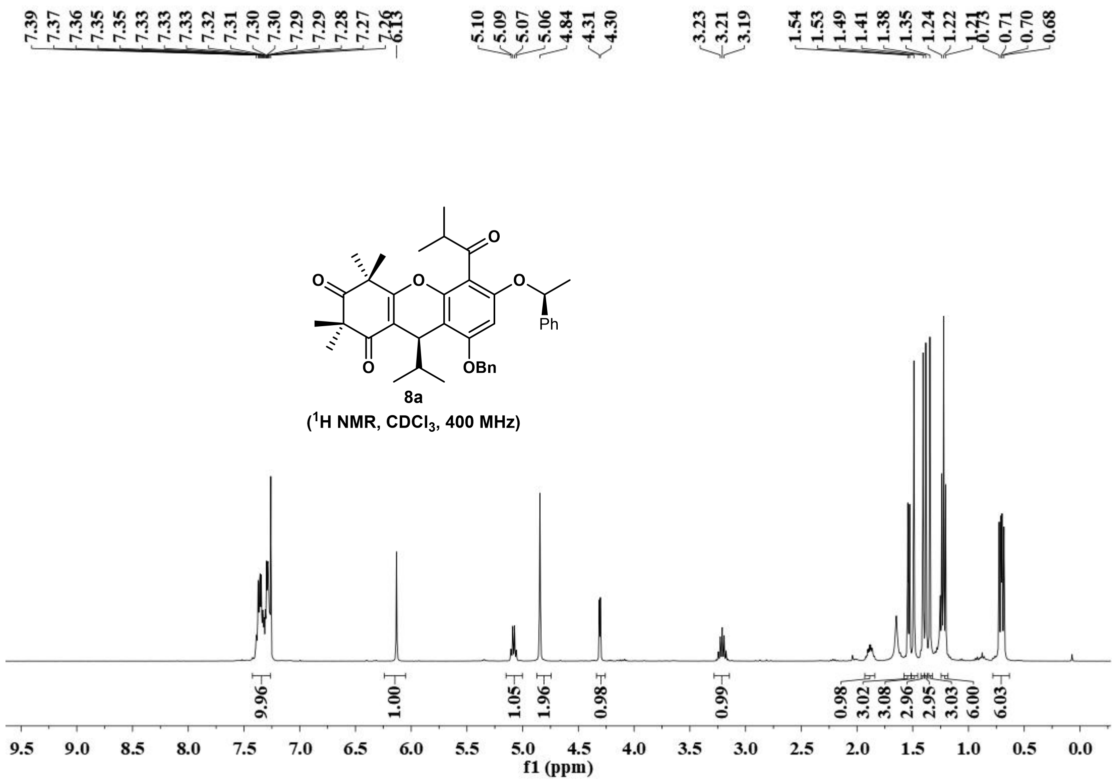

Supplementary Figure 95. ^{1}H NMR spectra of 8a.

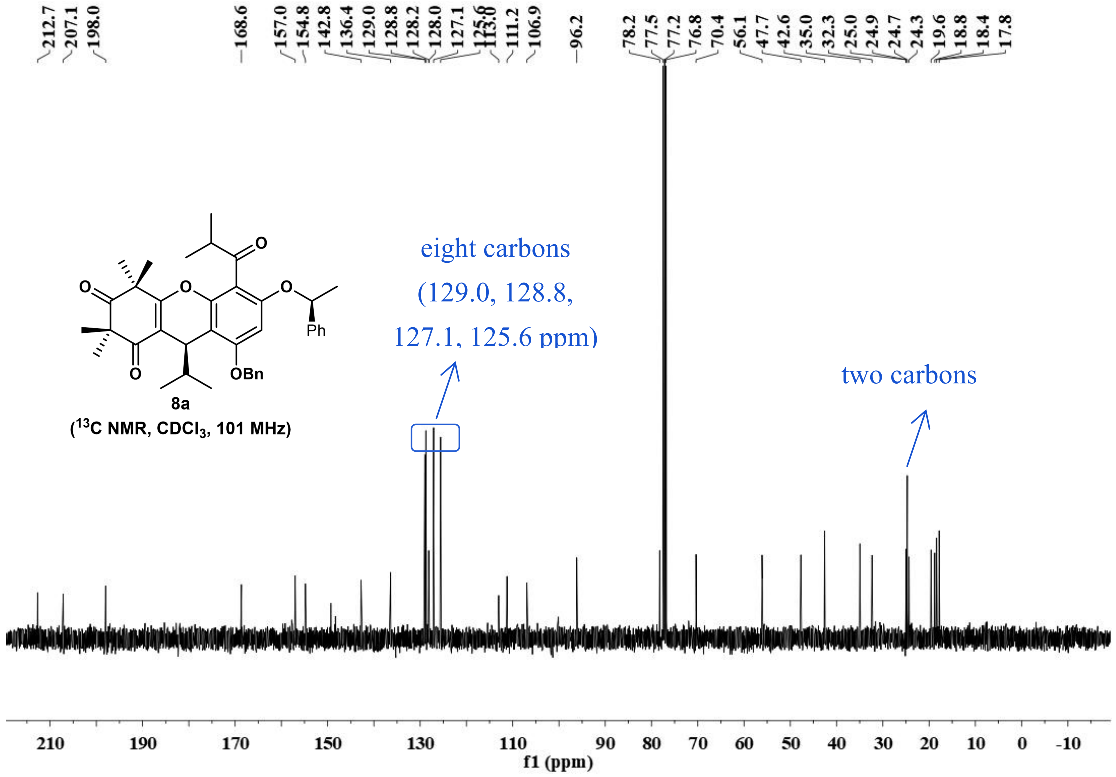

Supplementary Figure 96. ^{13}C NMR spectra of 8b.

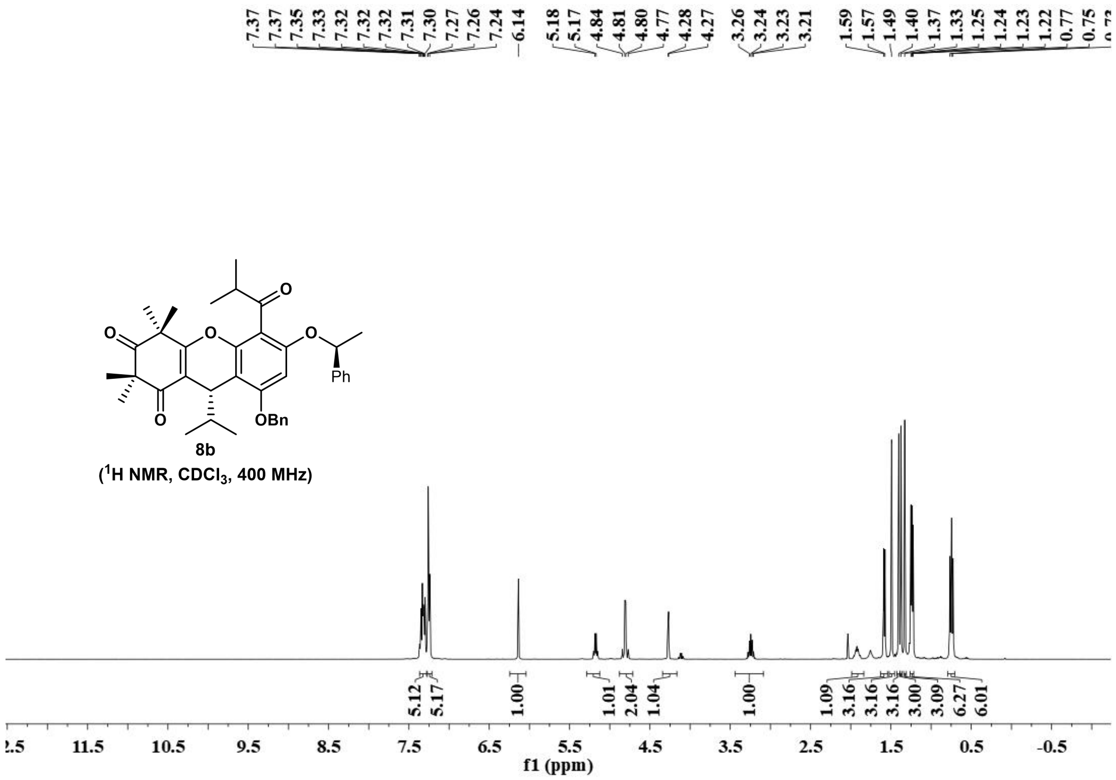

Supplementary Figure 97. ^{1}H NMR spectra of 8b.

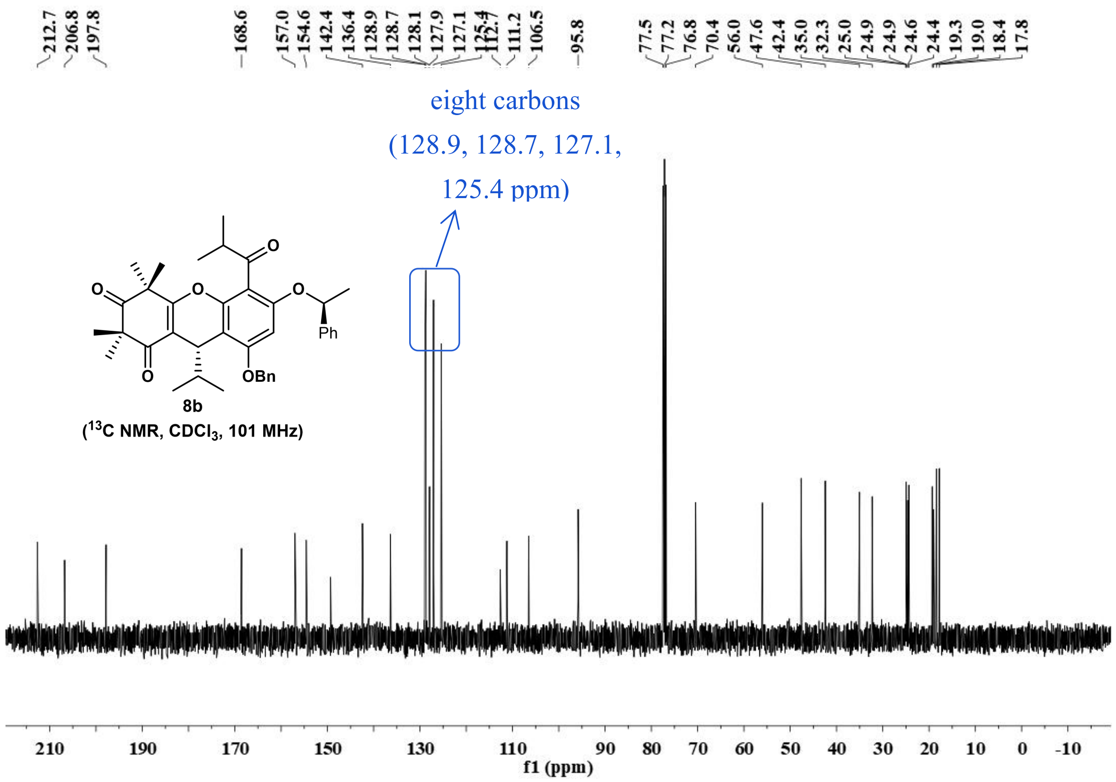

Supplementary Figure 98. ^{13}C NMR spectra of 8b.

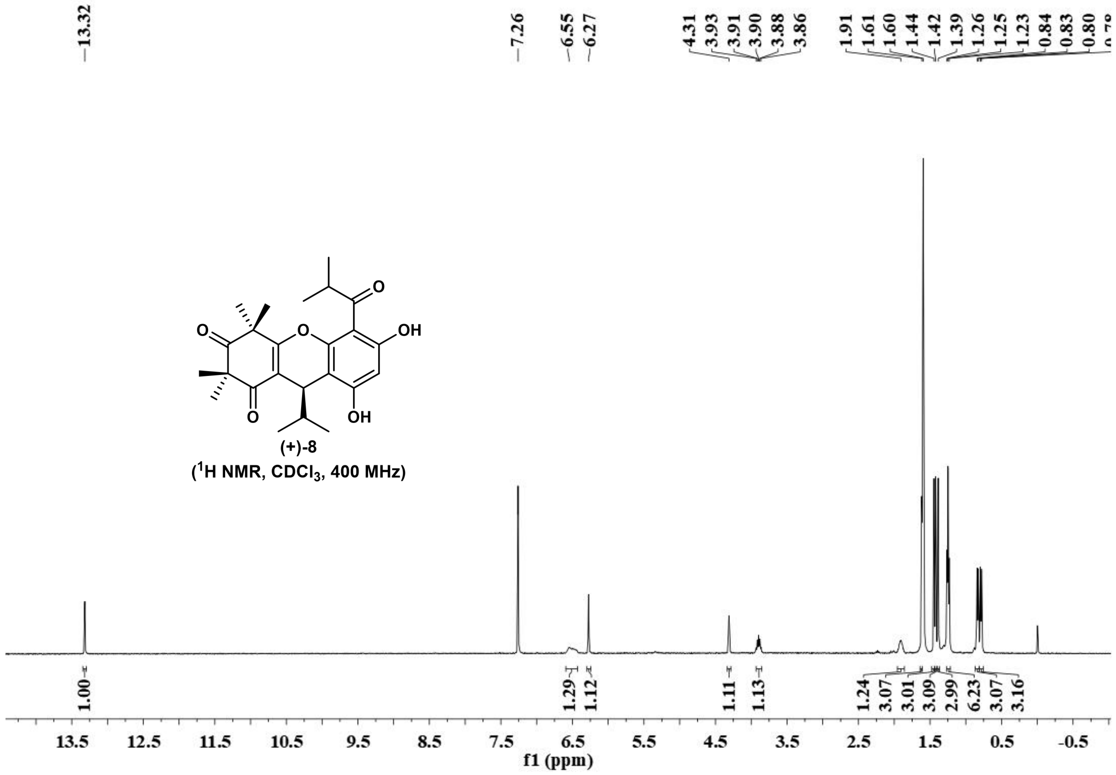

Supplementary Figure 99. ^{1}H NMR spectra of (+)-8.

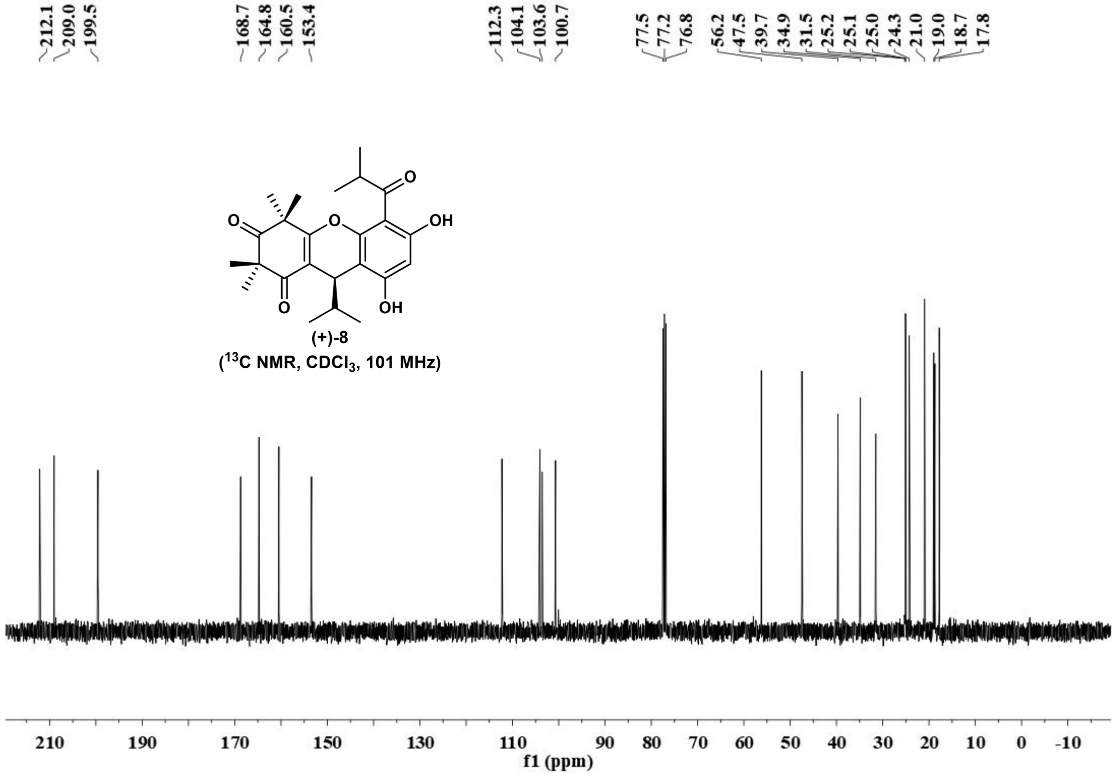

Supplementary Figure 100. ^{13}C NMR spectra of (+)-8.

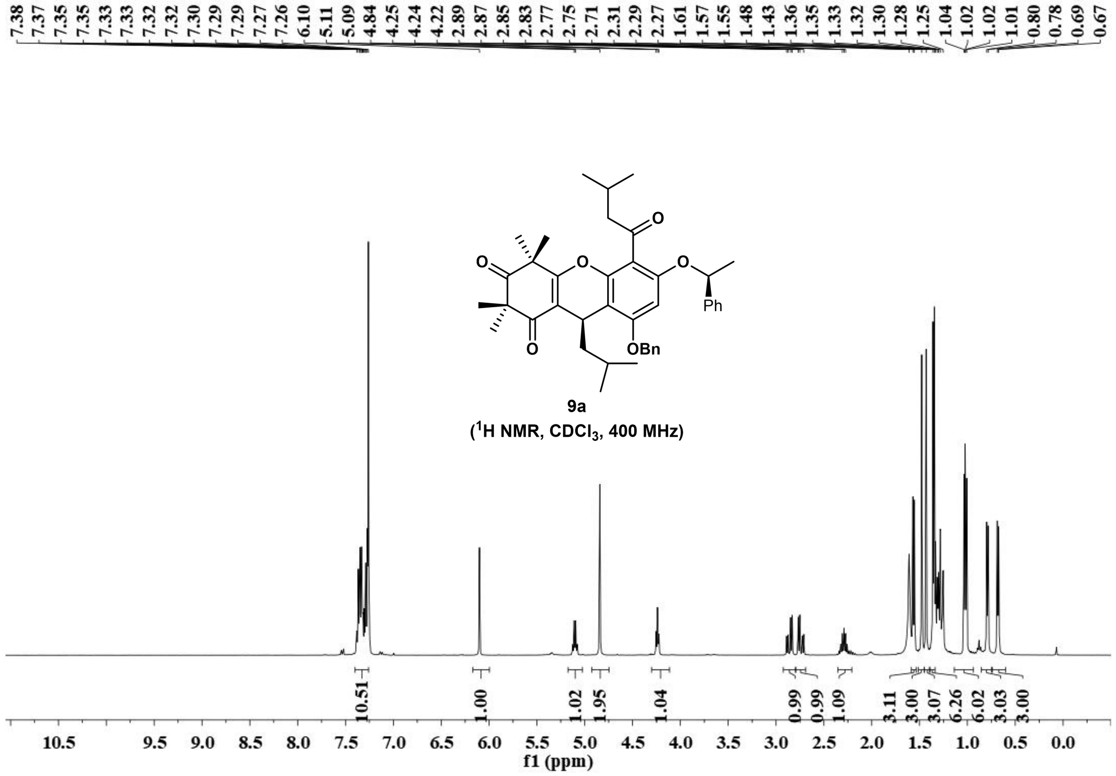

Supplementary Figure 101. ^{1}H NMR spectra of 9a.

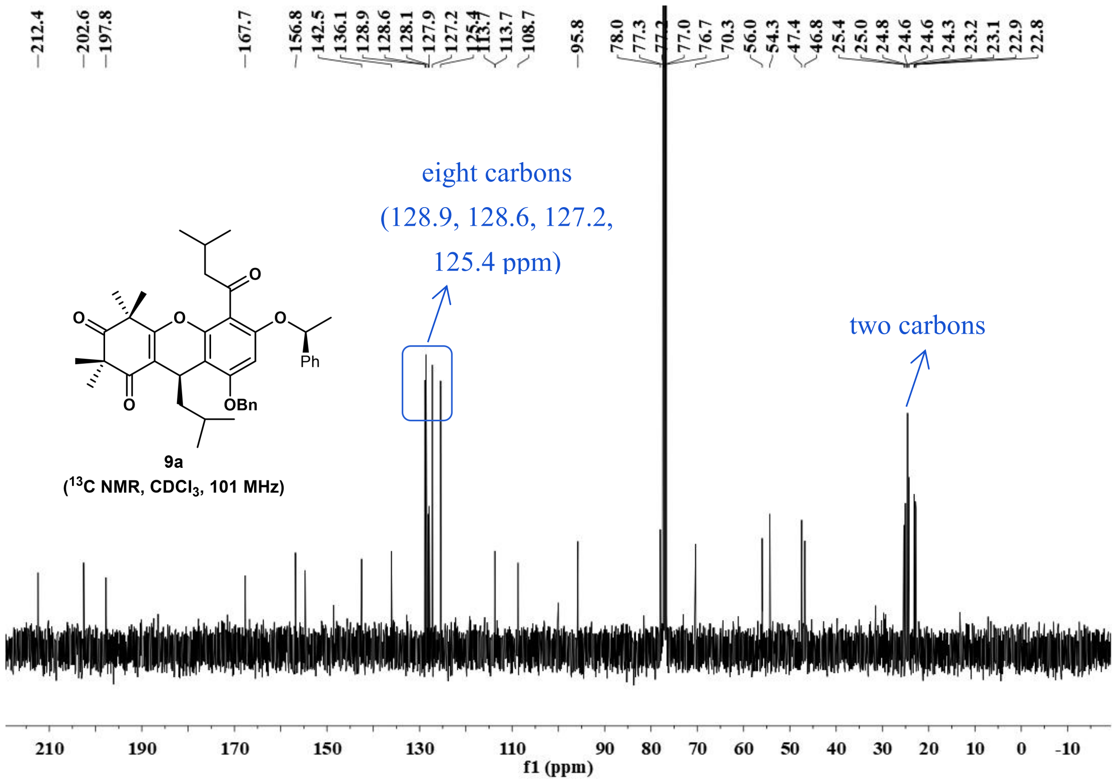

Supplementary Figure 102. ^{13}C NMR spectra of 9a.

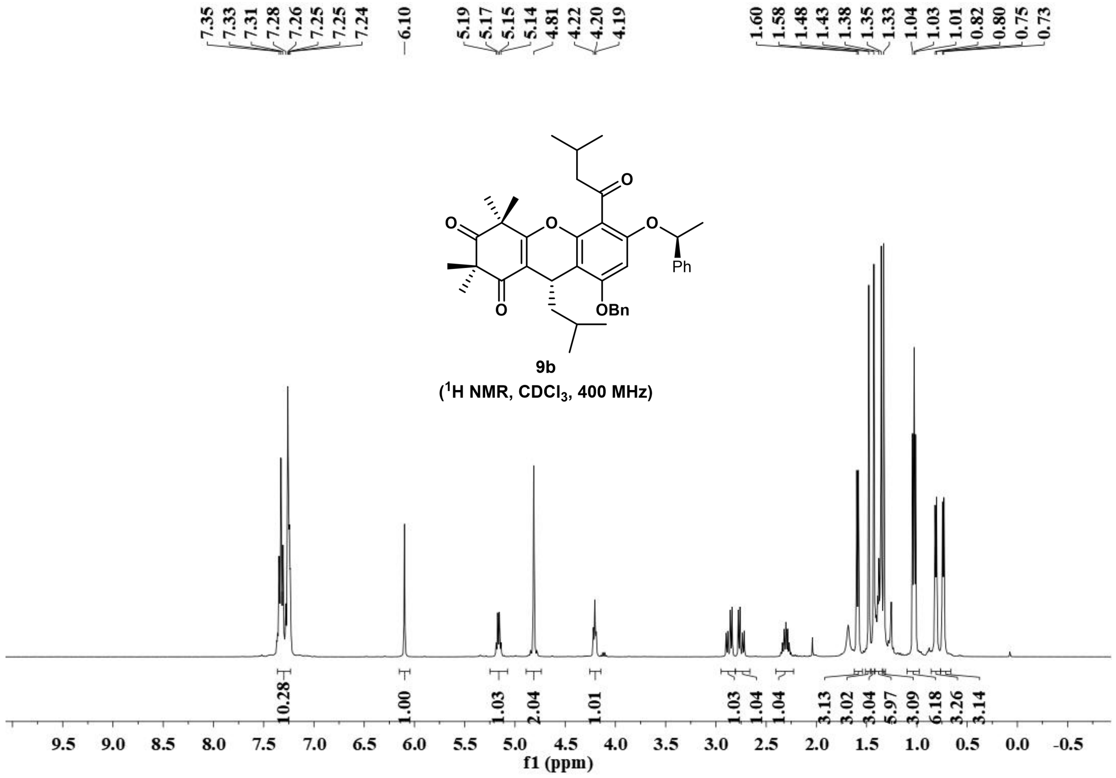

Supplementary Figure 103. ^{1}H NMR spectra of 9b.

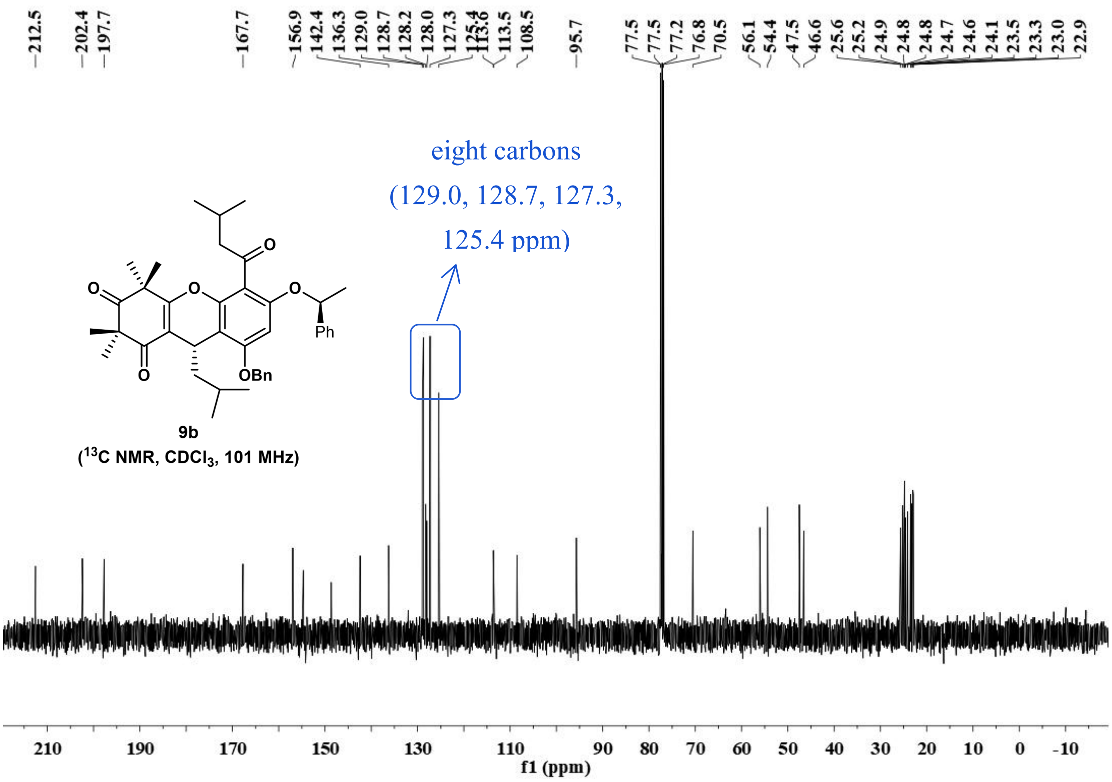

Supplementary Figure 104. ^{13}**C NMR spectra of 9b.**

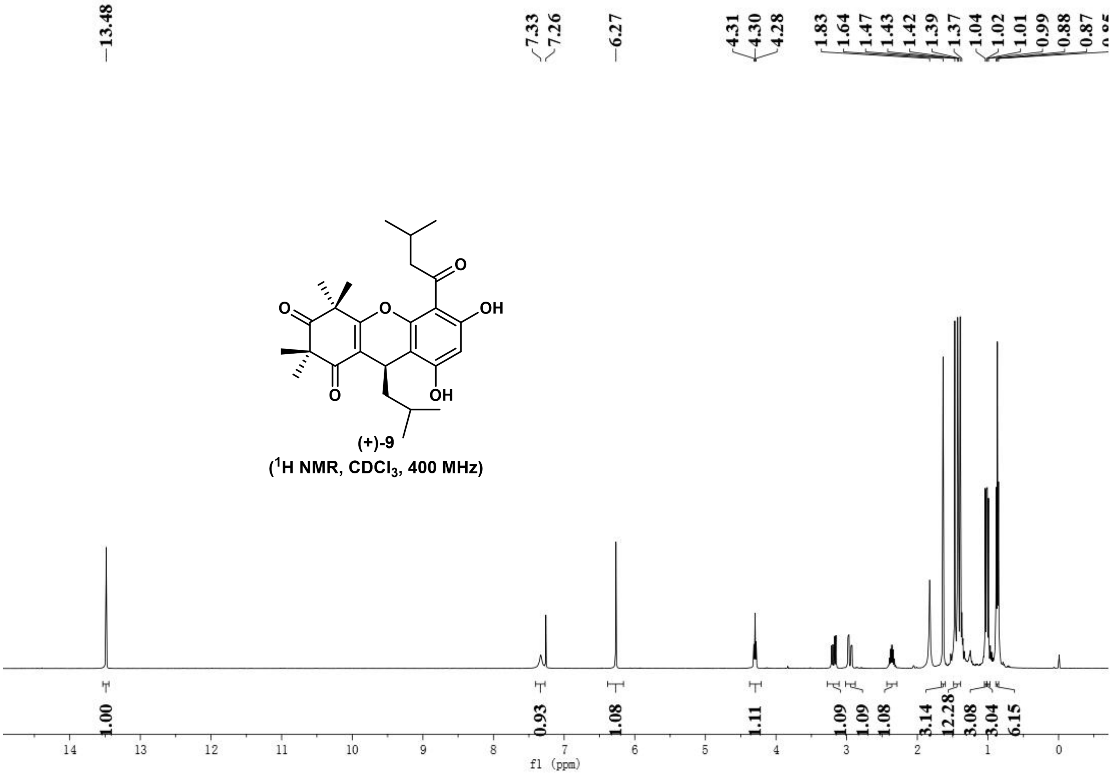

Supplementary Figure 105. ^{1}**H NMR spectra of (+)-9.**

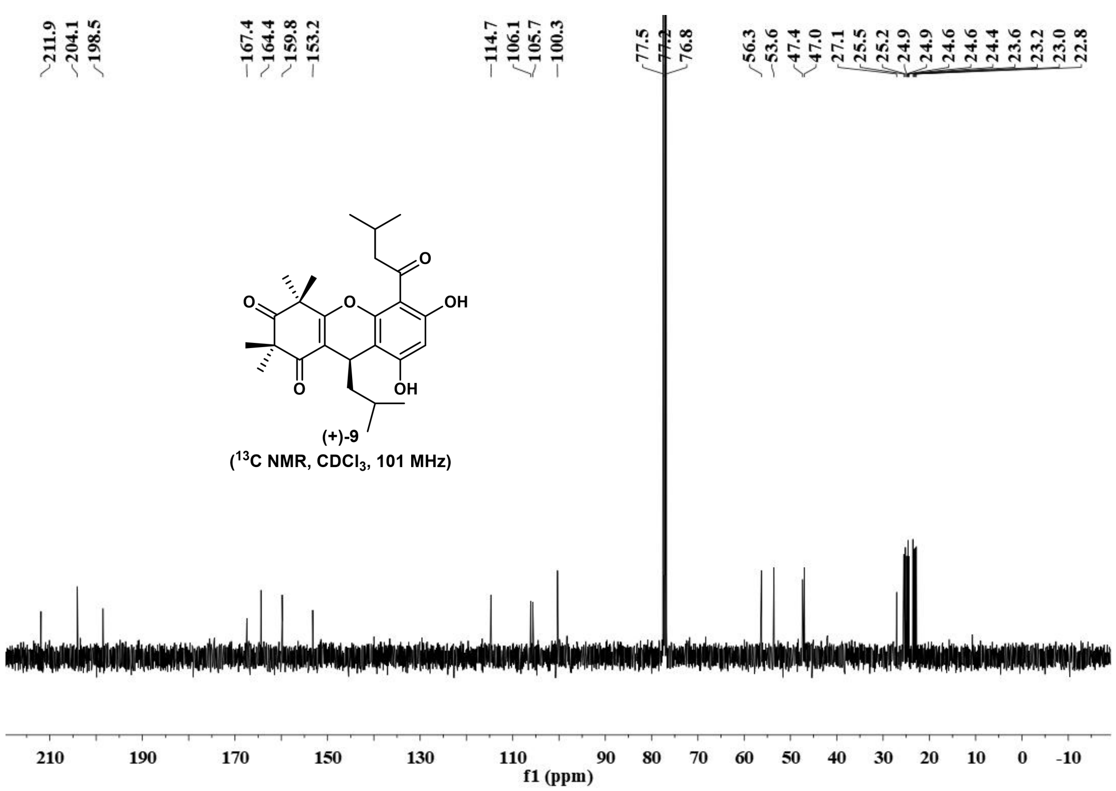

Supplementary Figure 106. ^{13}C NMR spectra of (+)-9.

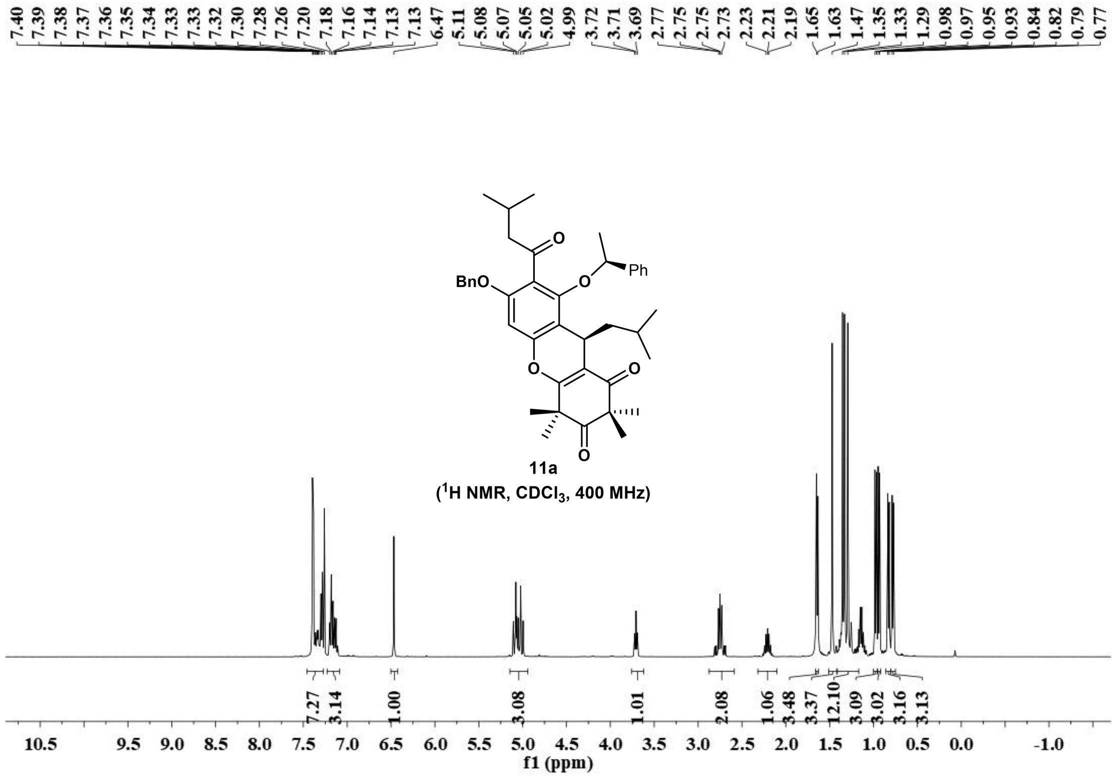

Supplementary Figure 107. ^{1}H NMR spectra of 11a.

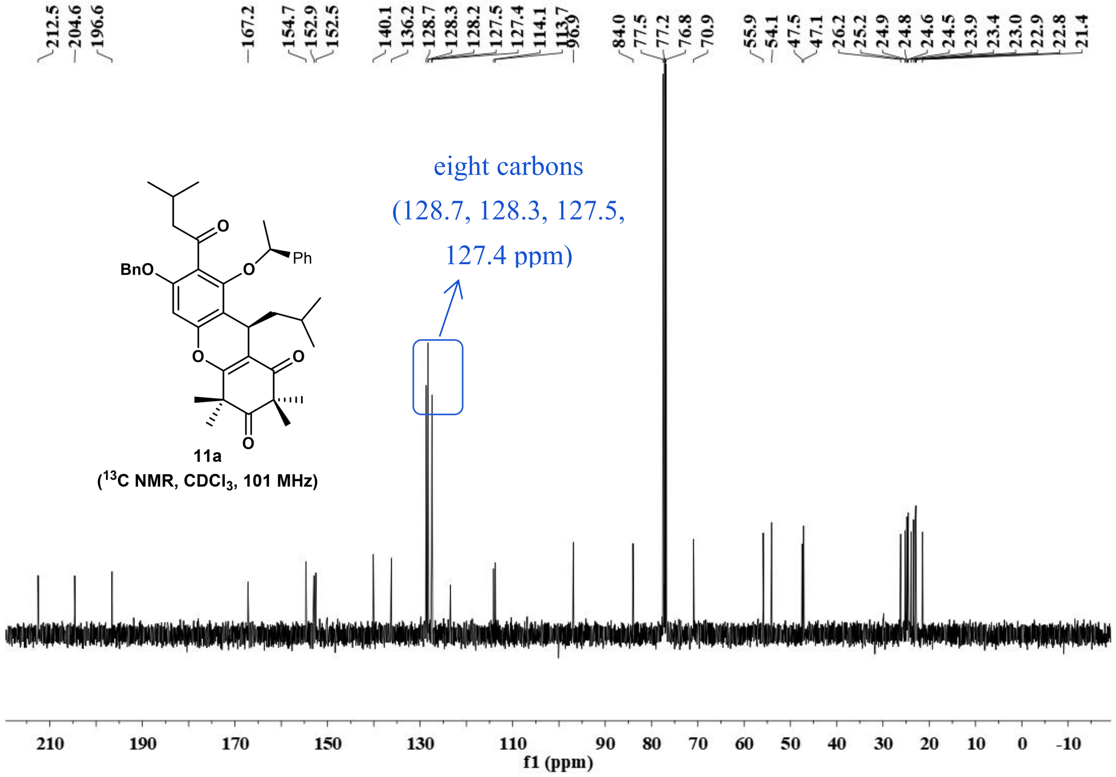

Supplementary Figure 108. ^{13}**C NMR spectra of 11a.**

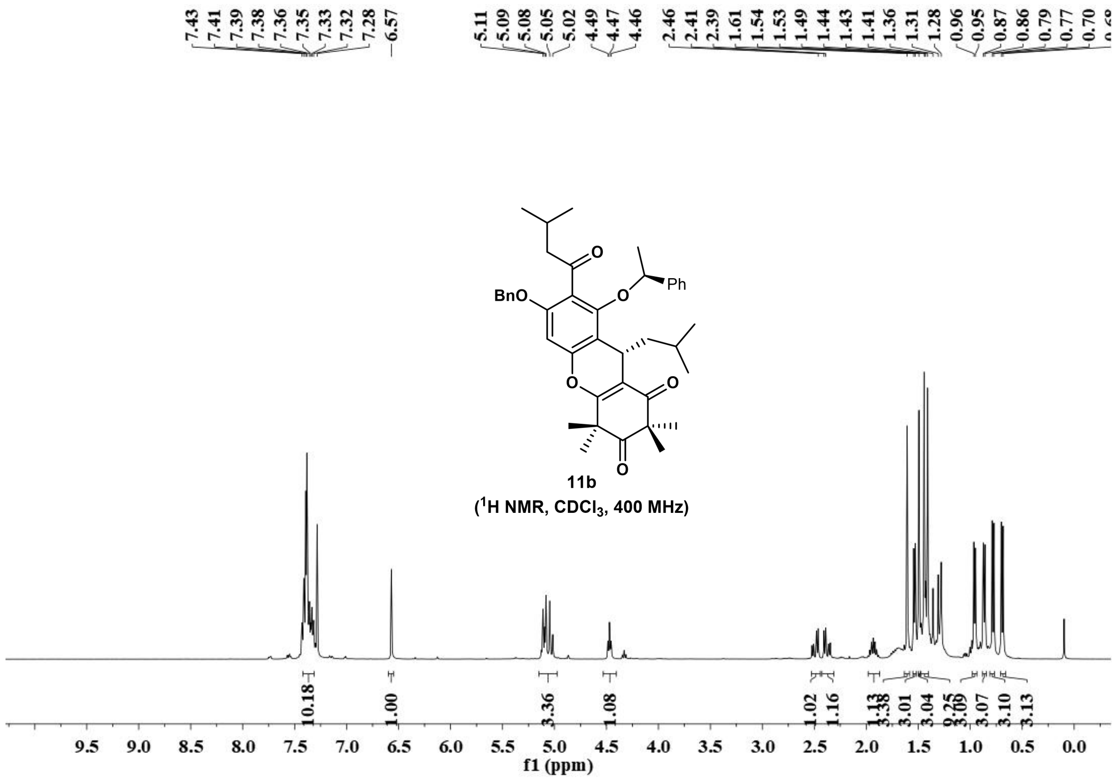

Supplementary Figure 109. ^{1}**H NMR spectra of 11b.**

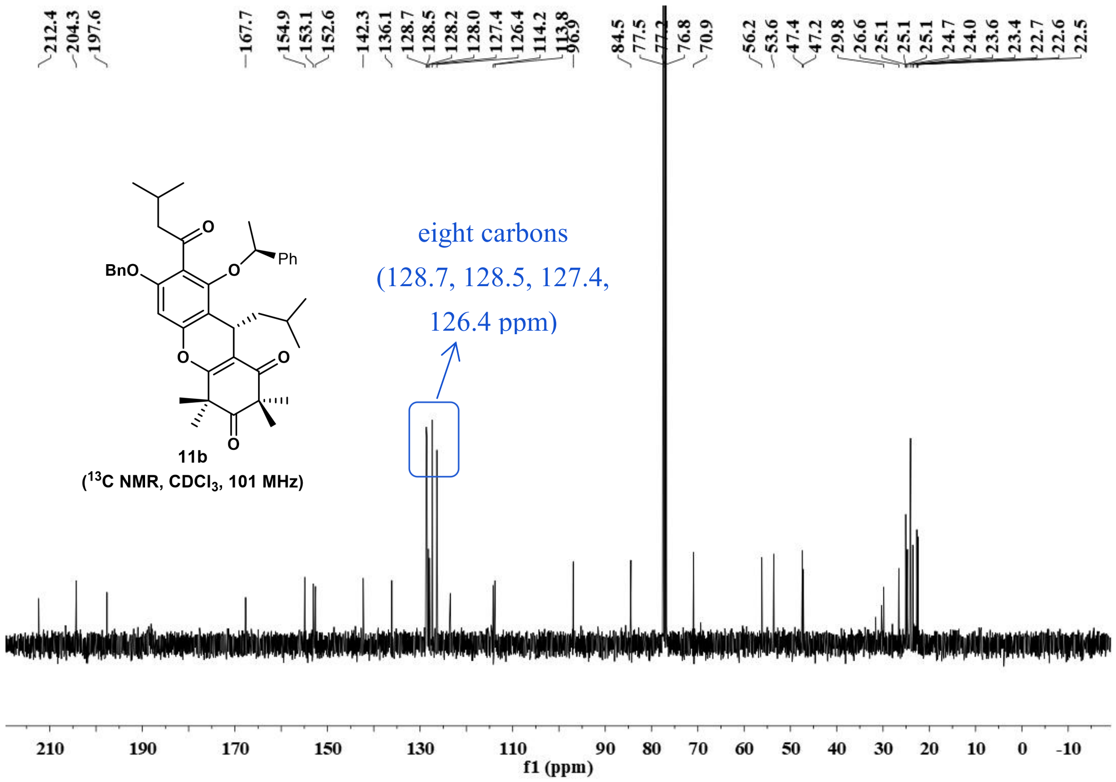

Supplementary Figure 110. ^{13}C NMR spectra of 11b.

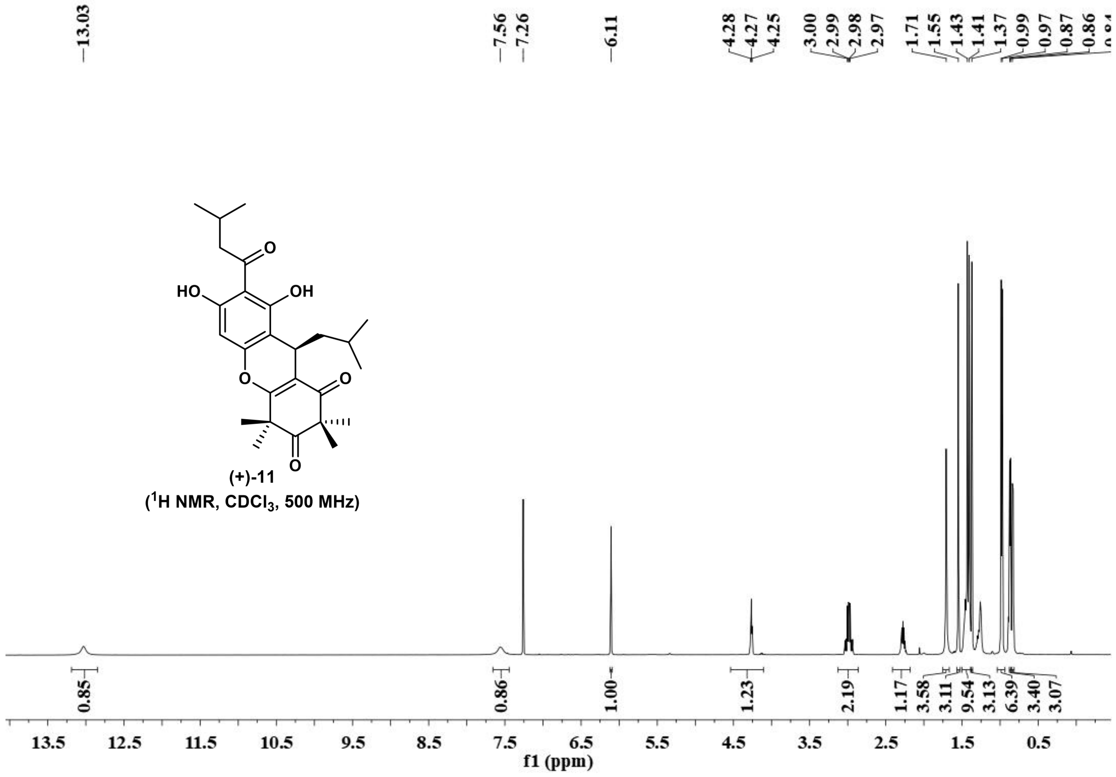

Supplementary Figure 111. ^{1}H NMR spectra of (+)-11.

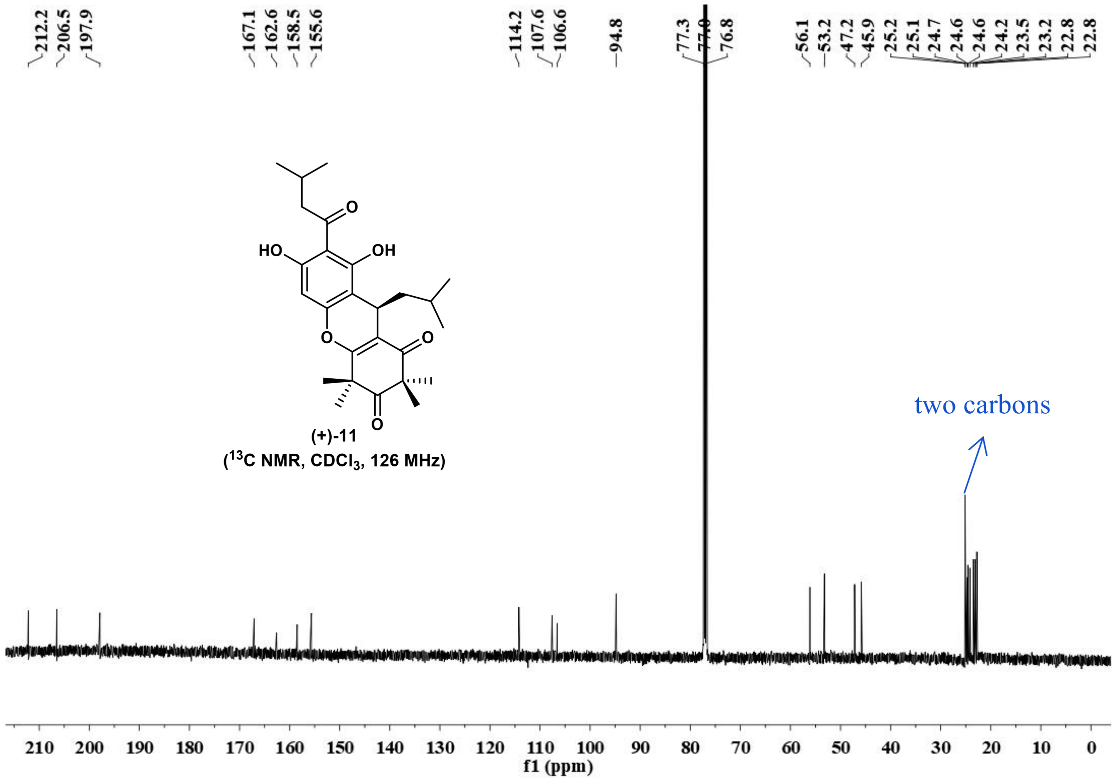

Supplementary Figure 112. ^{13}C NMR spectra of (+)-11.

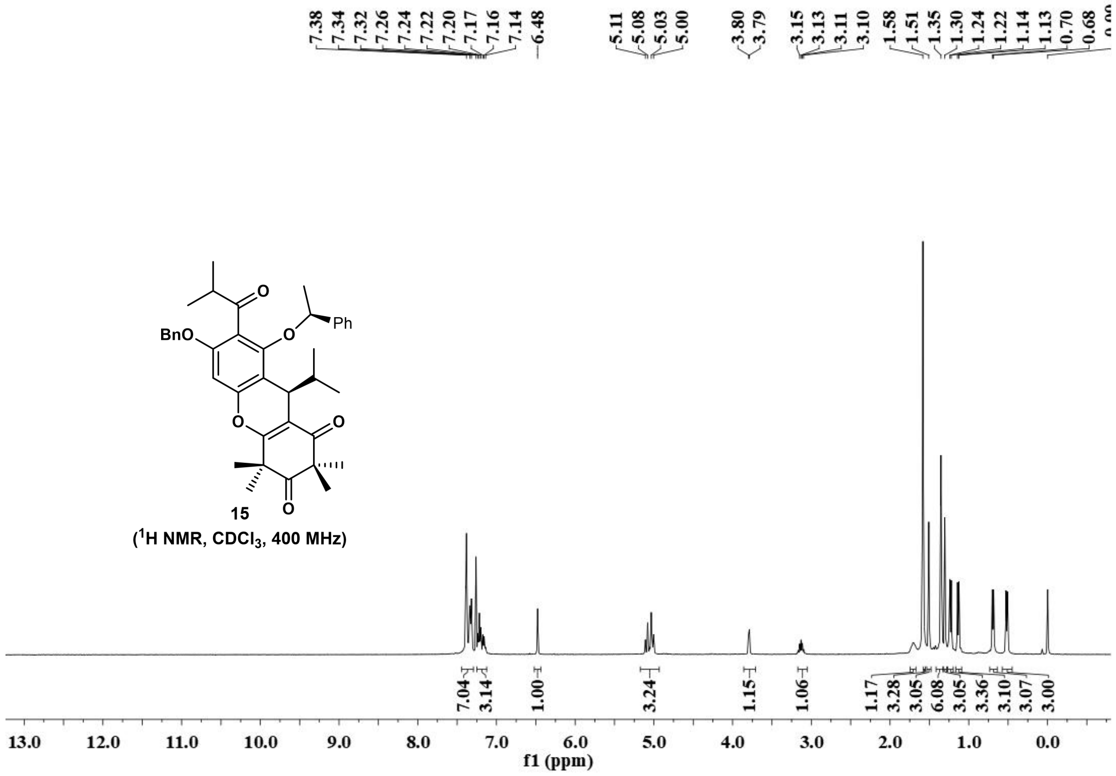

Supplementary Figure 113. ^{1}H NMR spectra of 15.

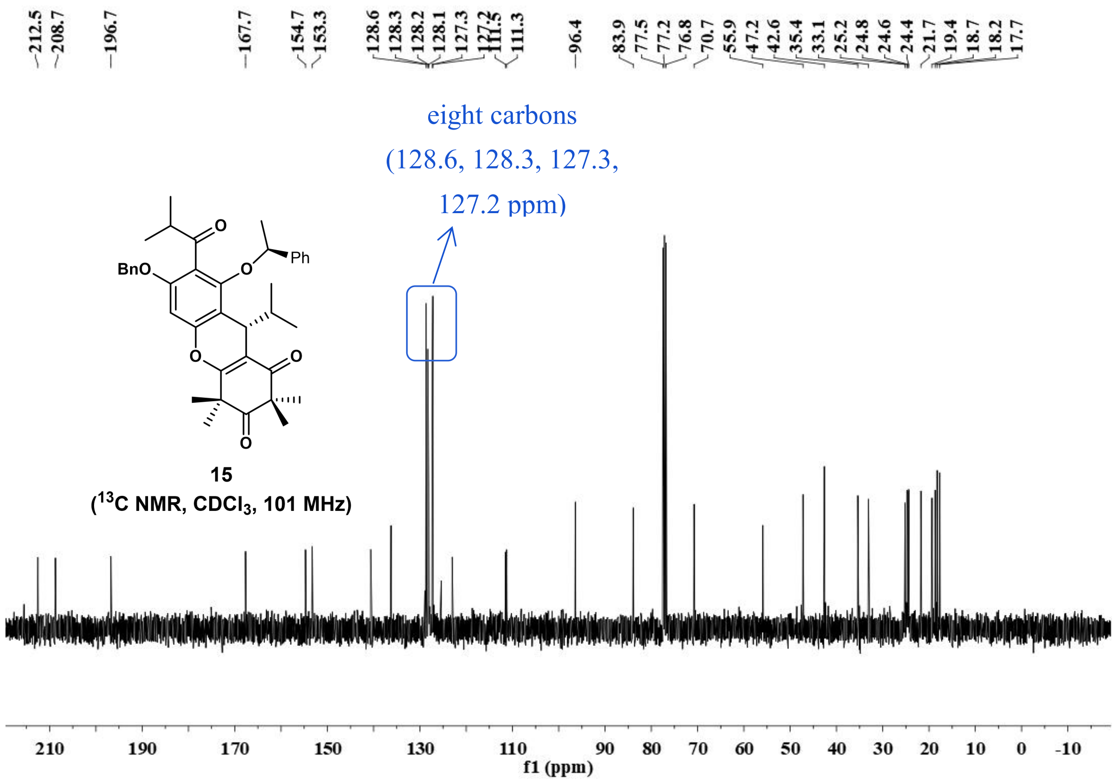

Supplementary Figure 114. ^{13}C NMR spectra of 15.

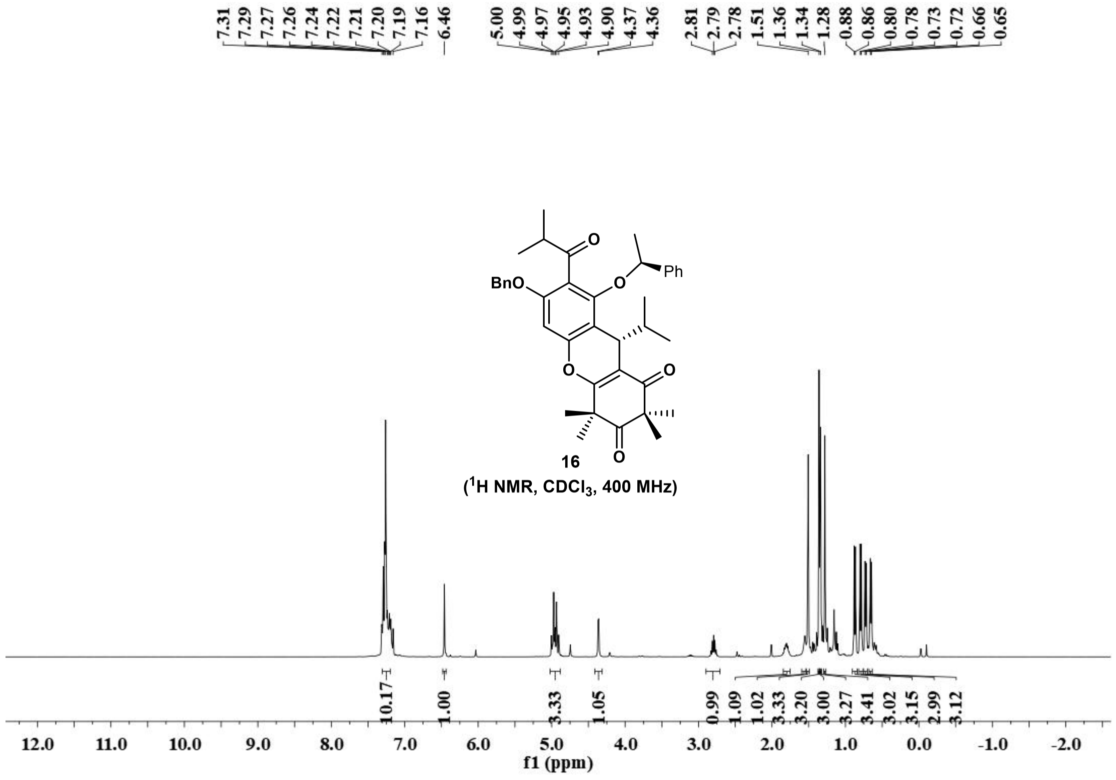

Supplementary Figure 115. ^{1}H NMR spectra of 16.

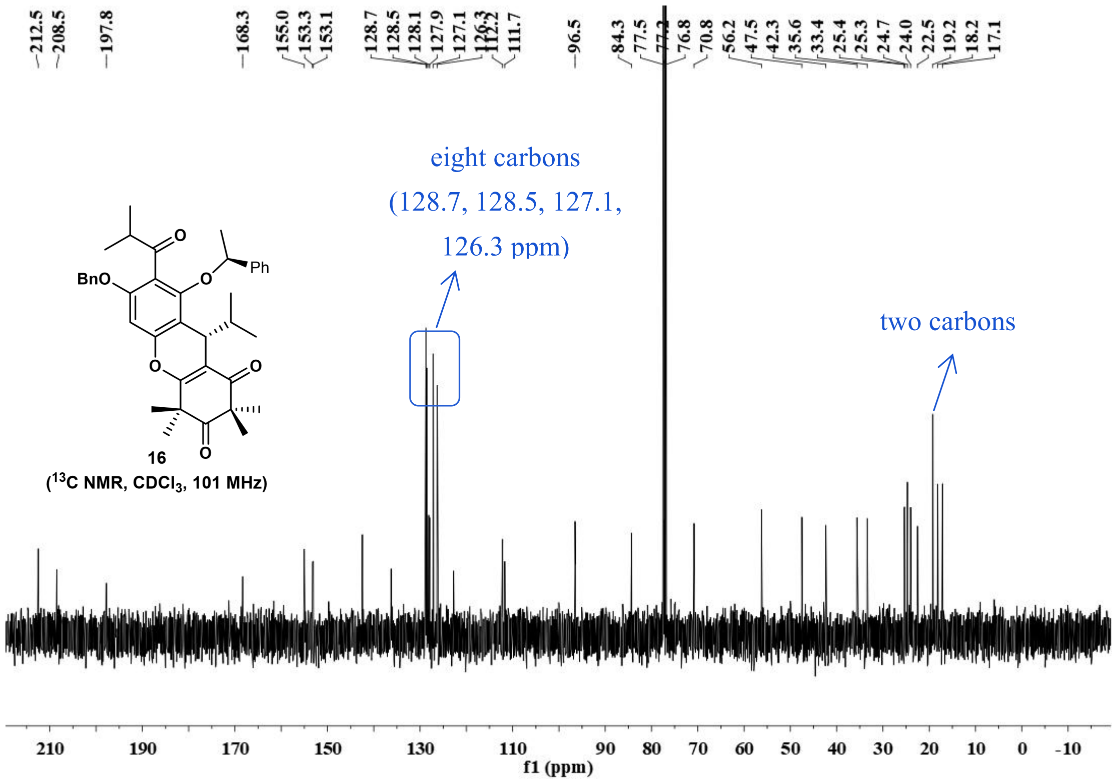

Supplementary Figure 116. ^{13}C NMR spectra of 16.

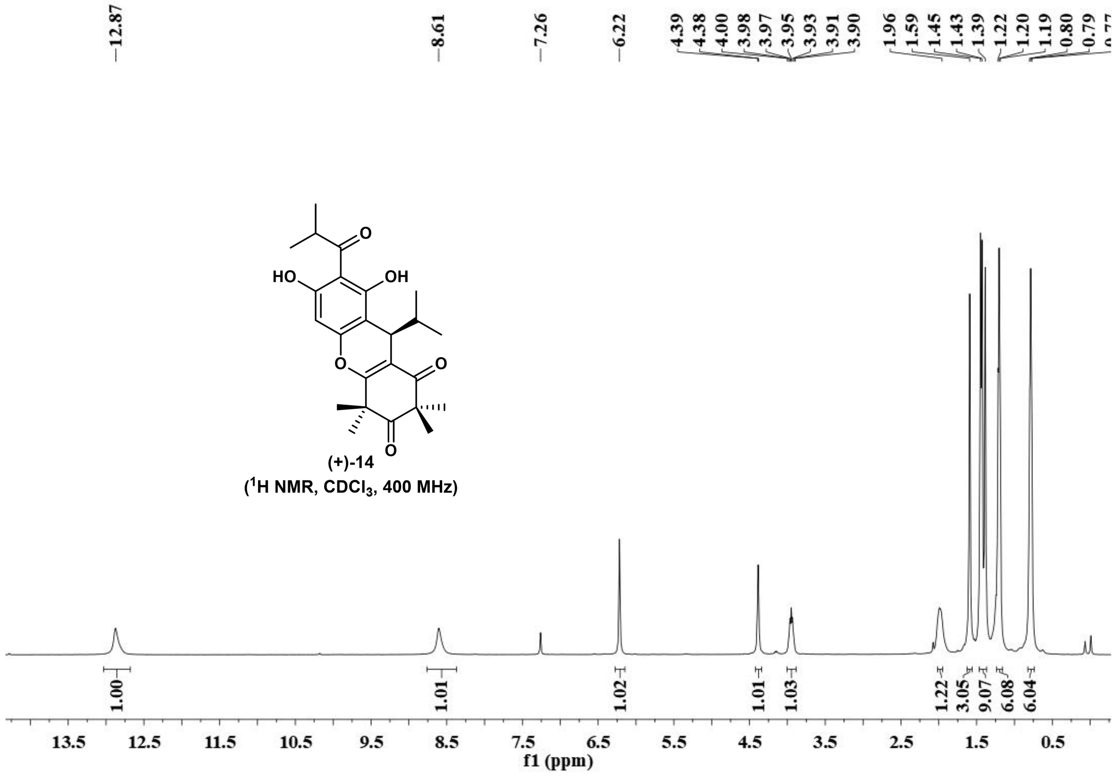

Supplementary Figure 117. ^{1}H NMR spectra of (+)-14.

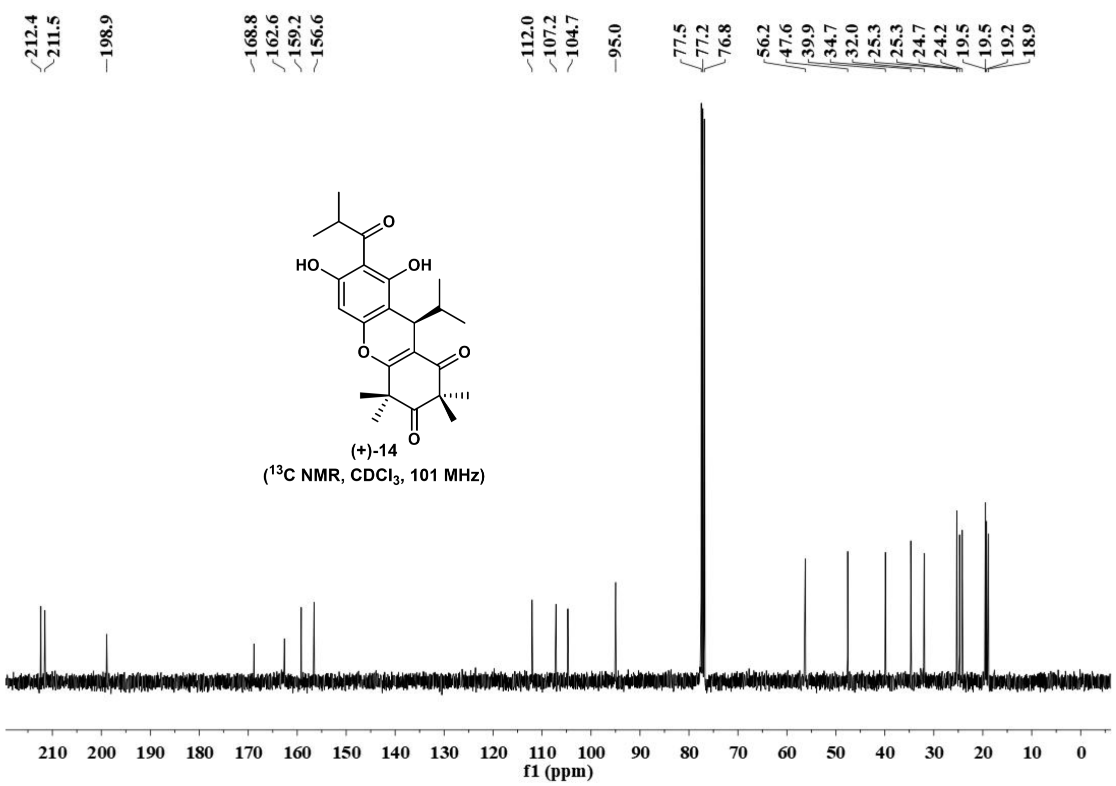

Supplementary Figure 118. ^{13}C NMR spectra of (+)-14.

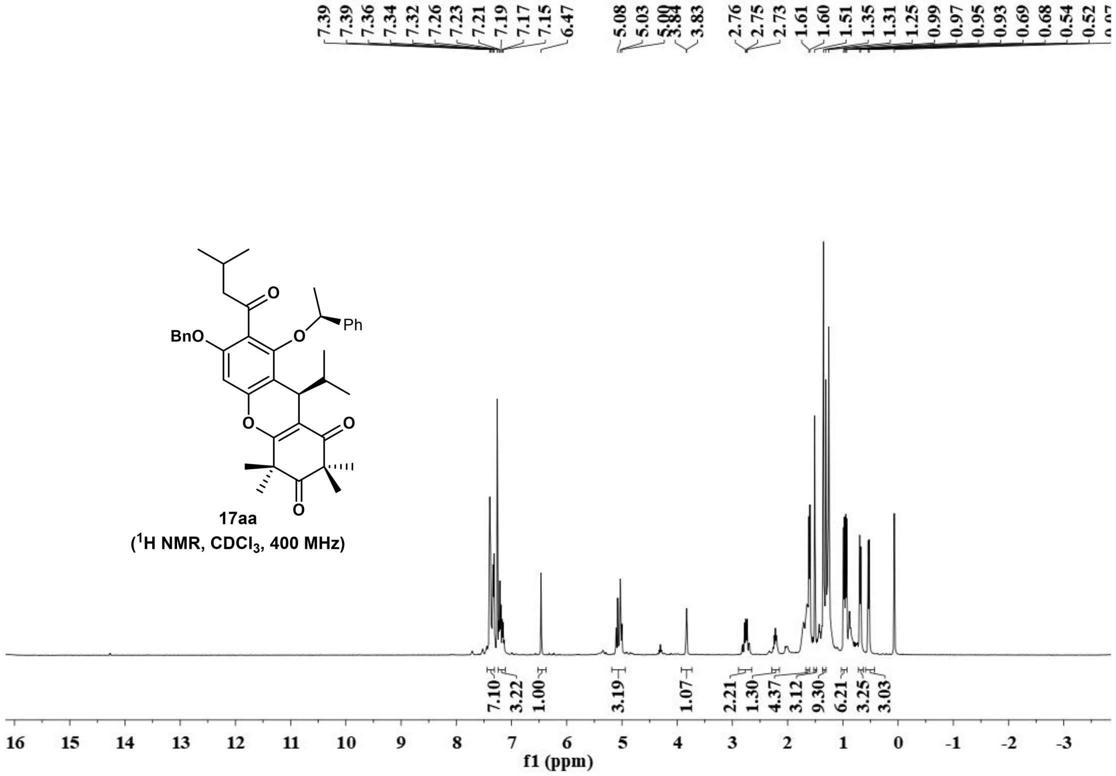

Supplementary Figure 119. ^{1}H NMR spectra of 17aa.

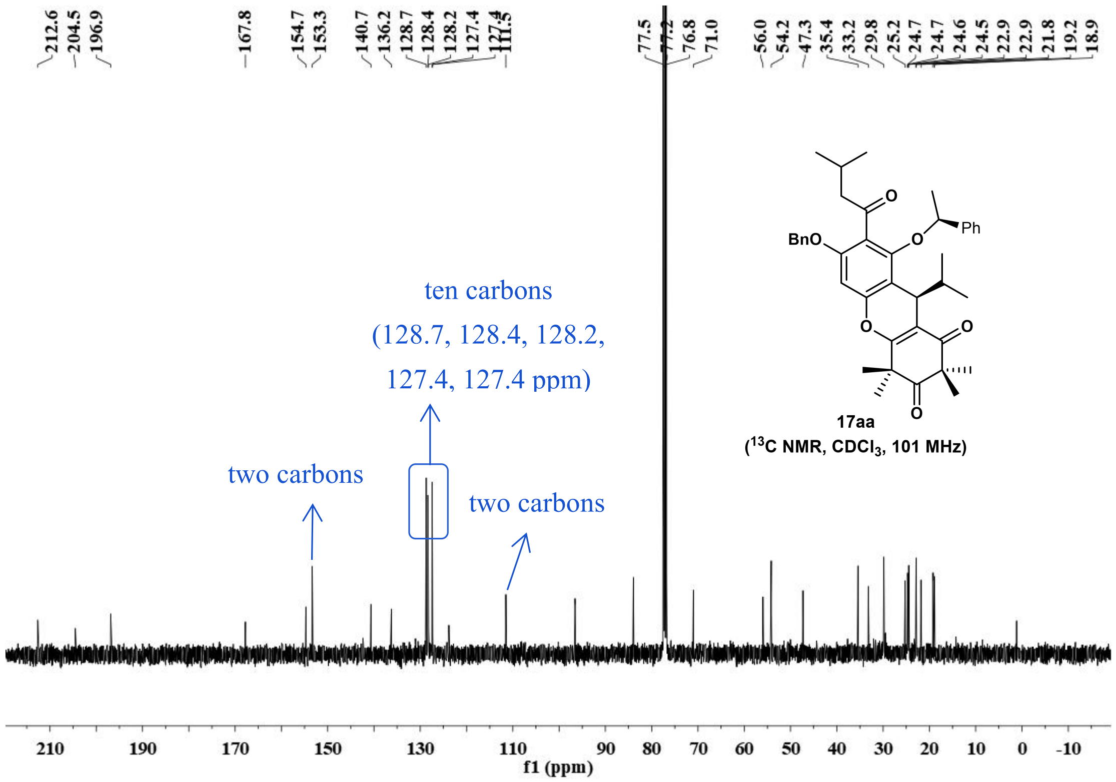

Supplementary Figure 120. ^{13}C NMR spectra of 17aa.

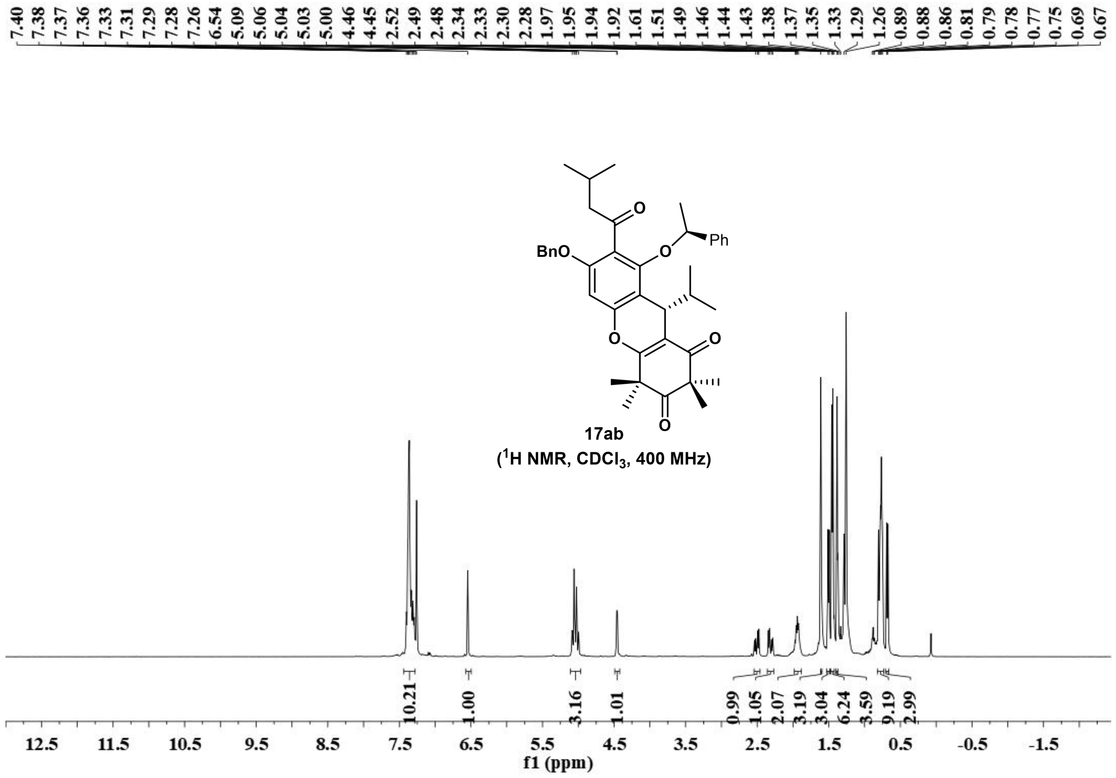

Supplementary Figure 121. ^{1}H NMR spectra of 17ab.

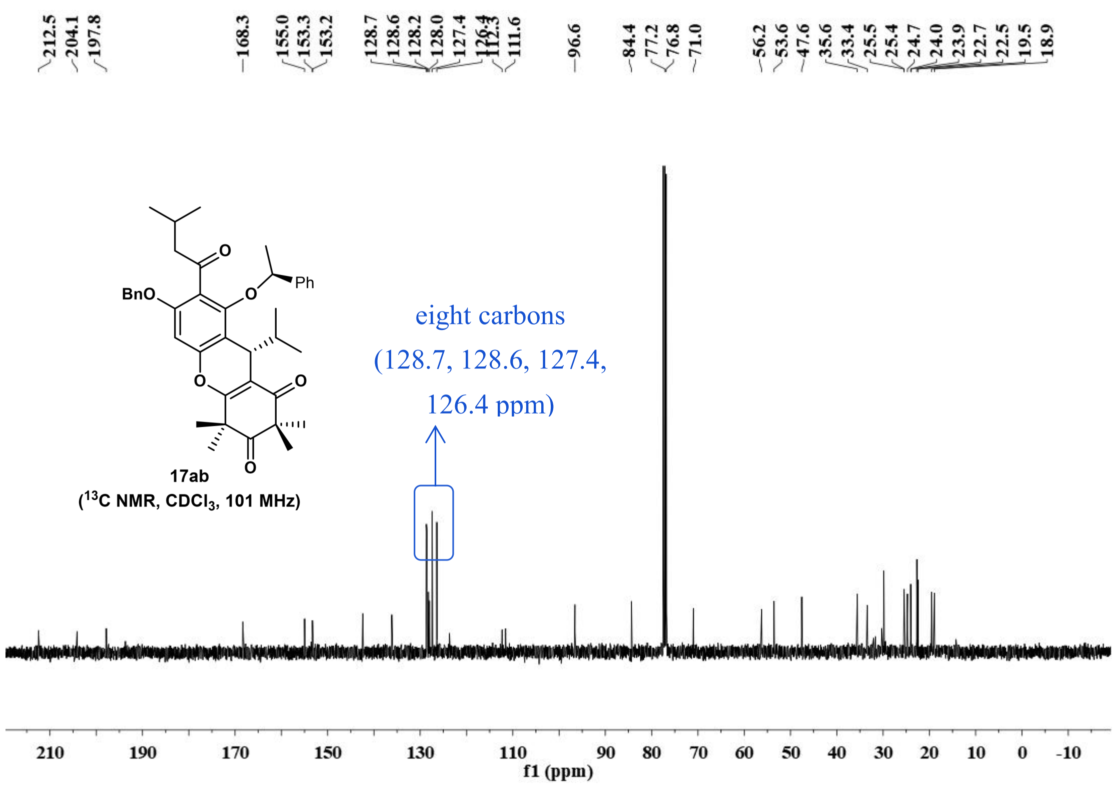

Supplementary Figure 122. ^{13}**C NMR spectra of 17ab.**

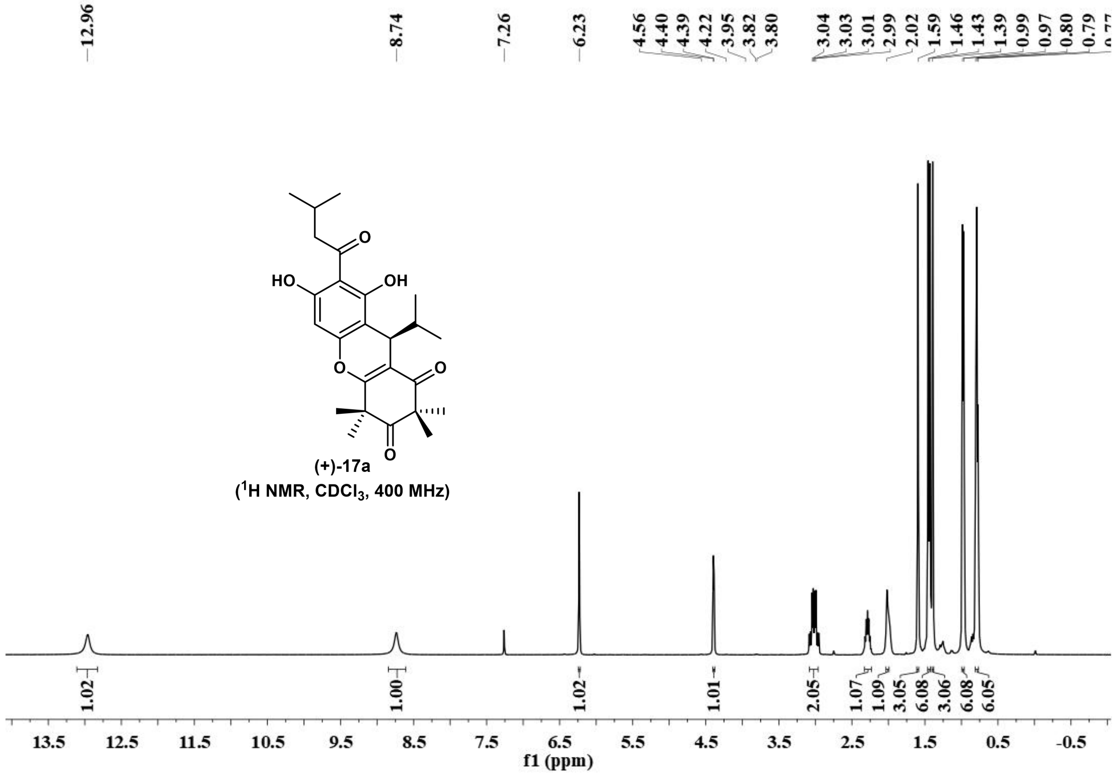

Supplementary Figure 123. ^{1}**H NMR spectra of (+)-17a.**

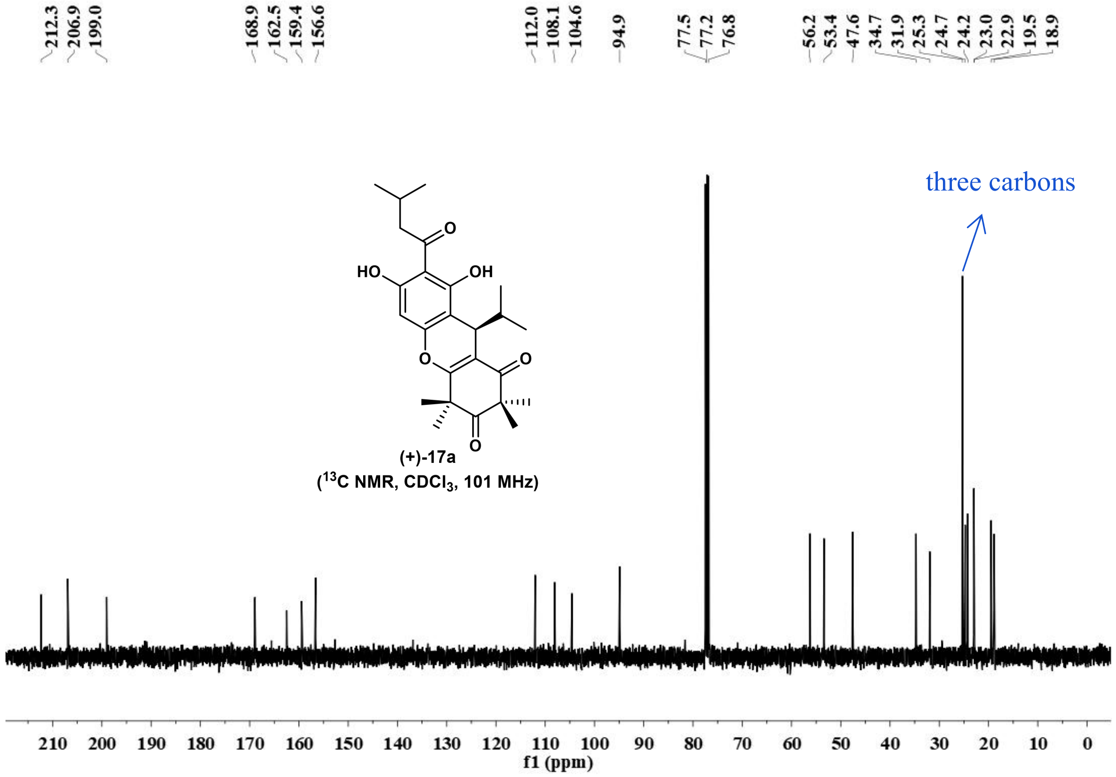

Supplementary Figure 124. ^{13}C NMR spectra of (+)-17a.

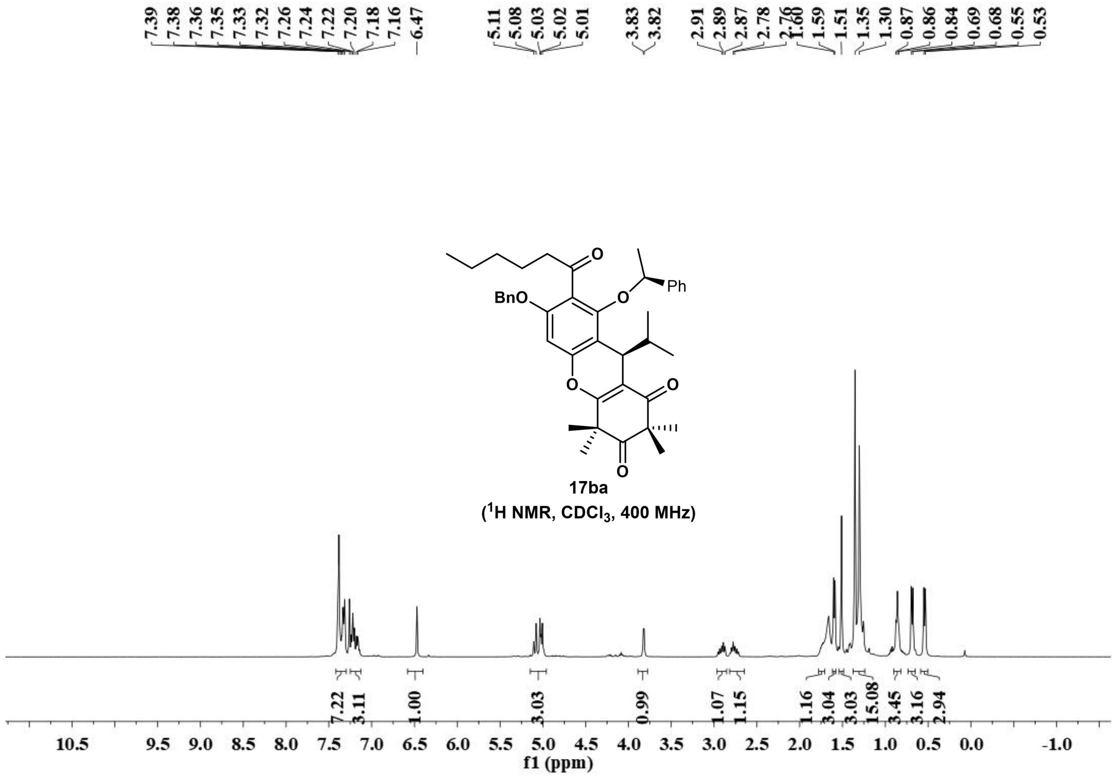

Supplementary Figure 125. ^{1}H NMR spectra of 17ba.

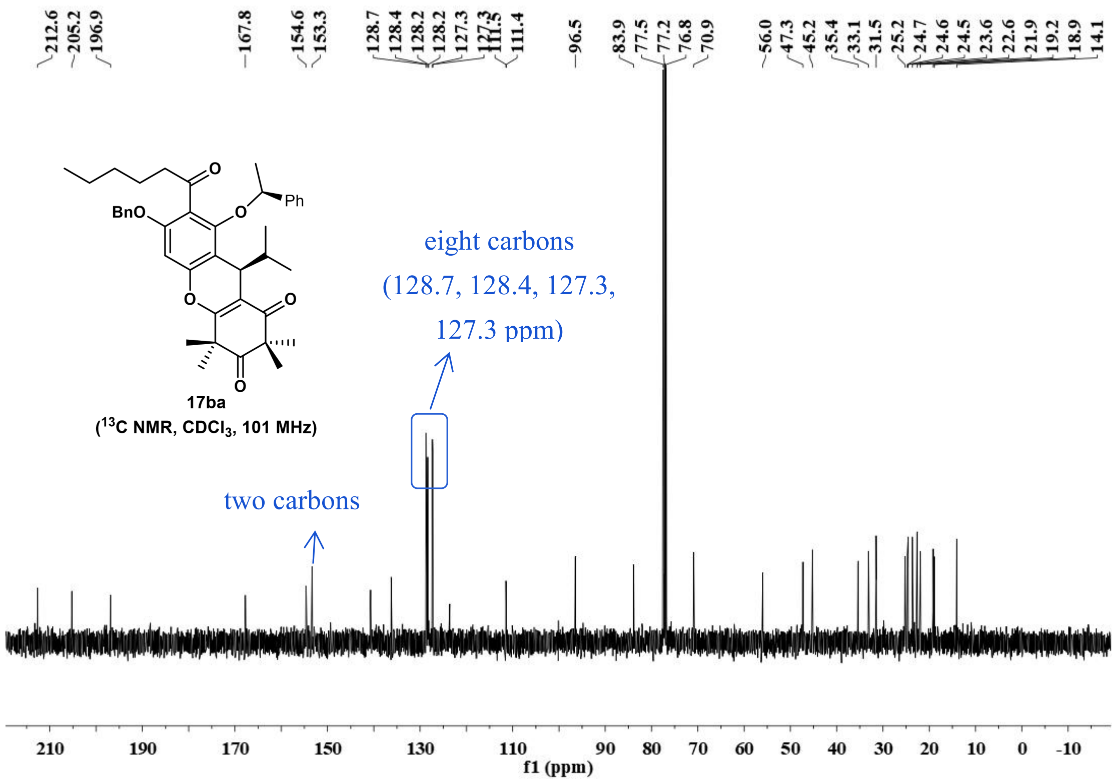

Supplementary Figure 126. ^{13}**C NMR spectra of 17ba.**

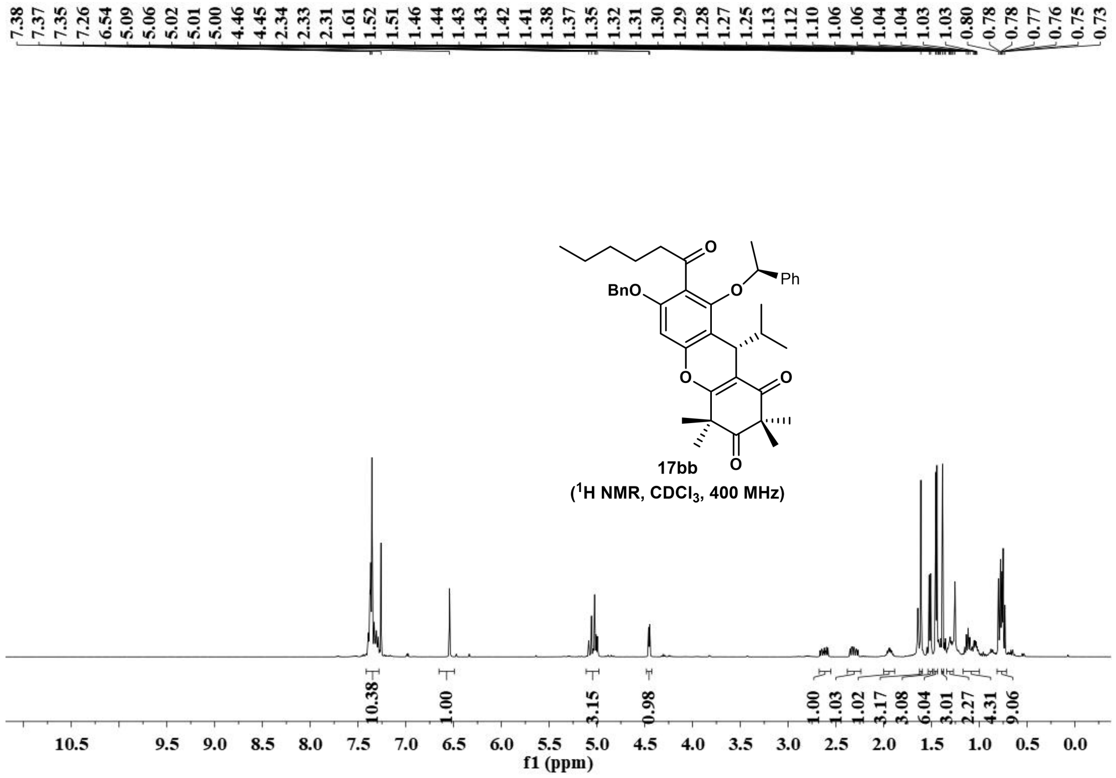

Supplementary Figure 127. ^{1}**H NMR spectra of 17bb.**

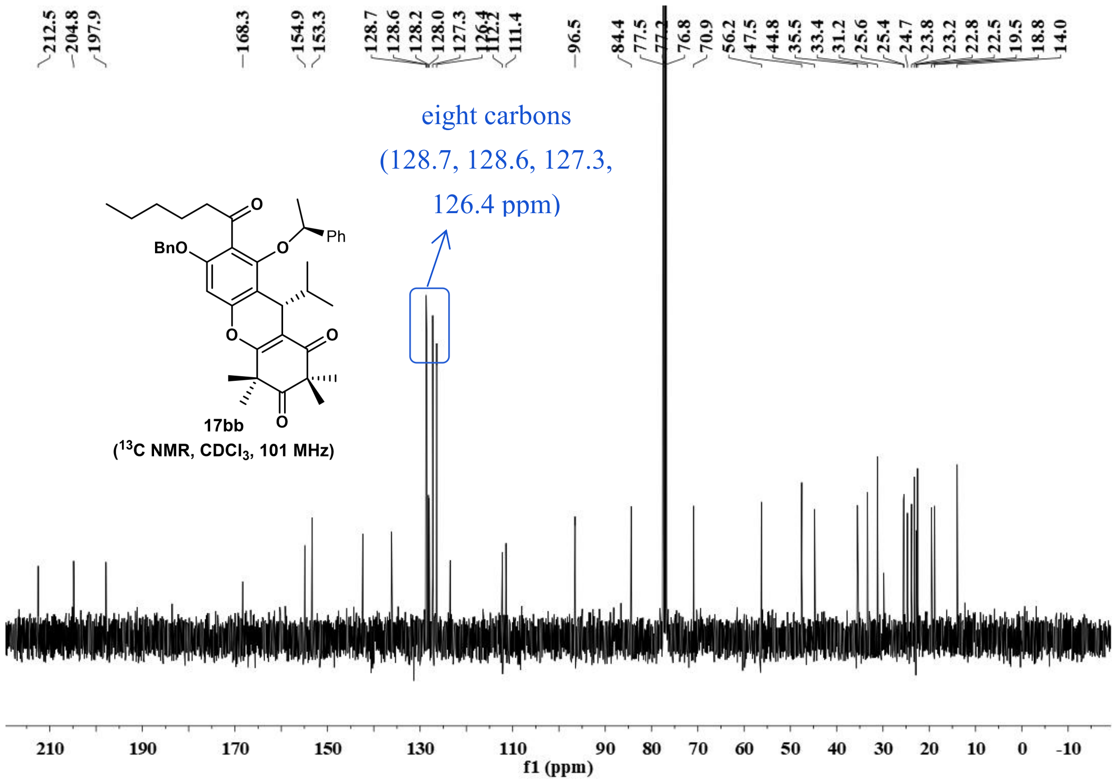

Supplementary Figure 128. ^{13}**C NMR spectra of 17bb.**

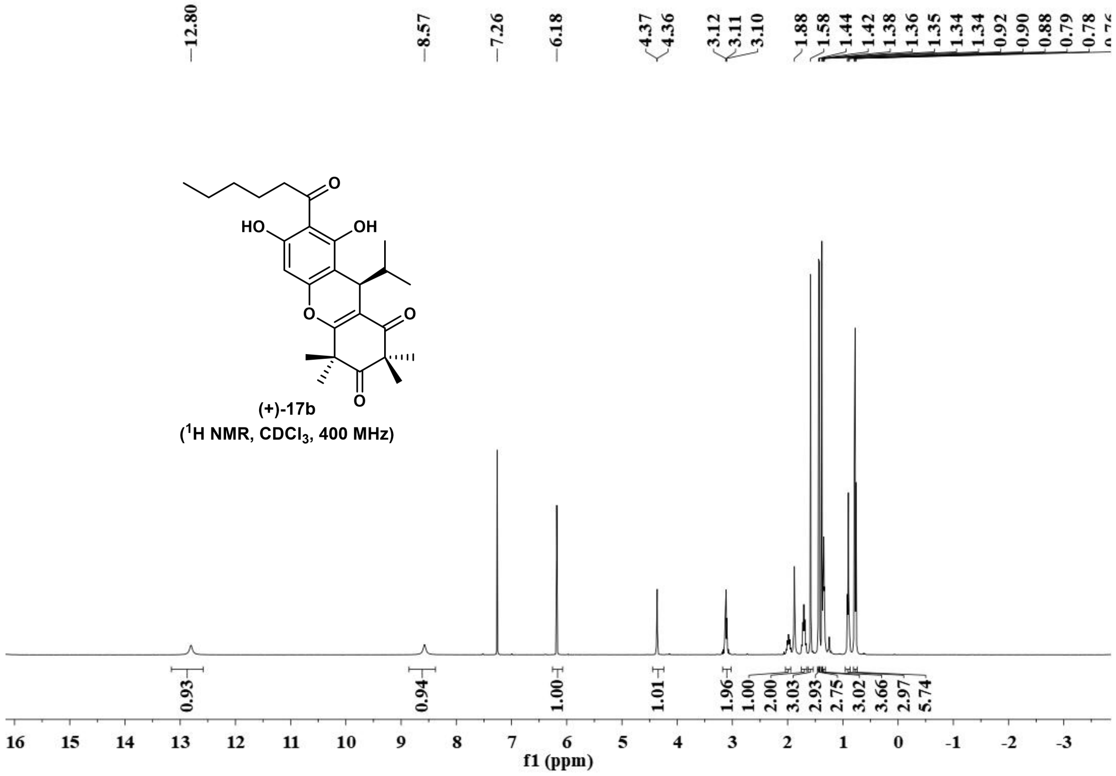

Supplementary Figure 129. ^{1}**H NMR spectra of (+)-17b.**

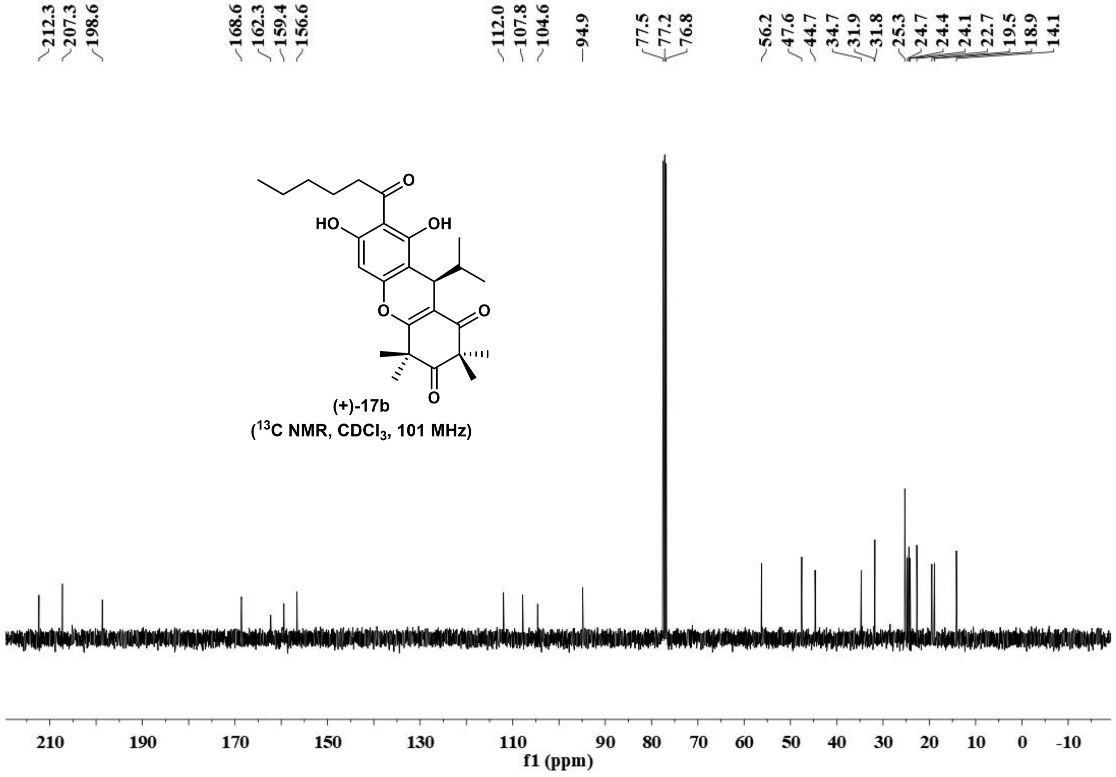

Supplementary Figure 130. ^{13}C NMR spectra of (+)-17b.

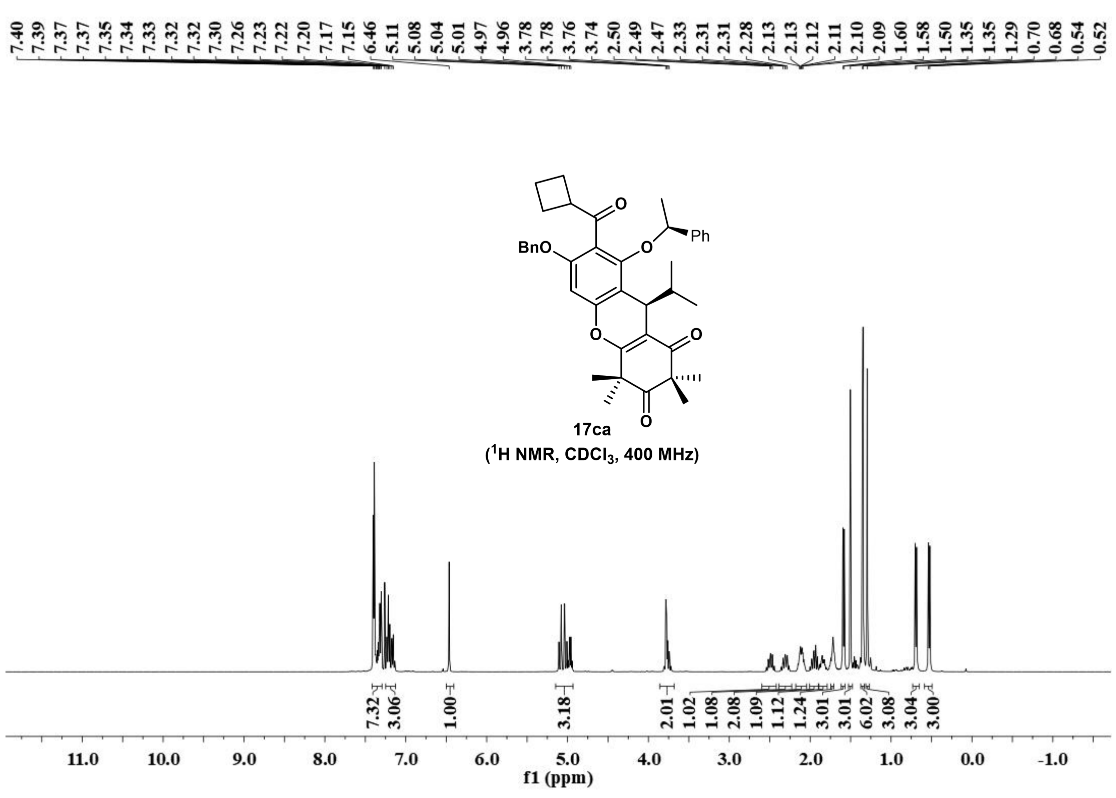

Supplementary Figure 131. ^{1}H NMR spectra of 17ca.

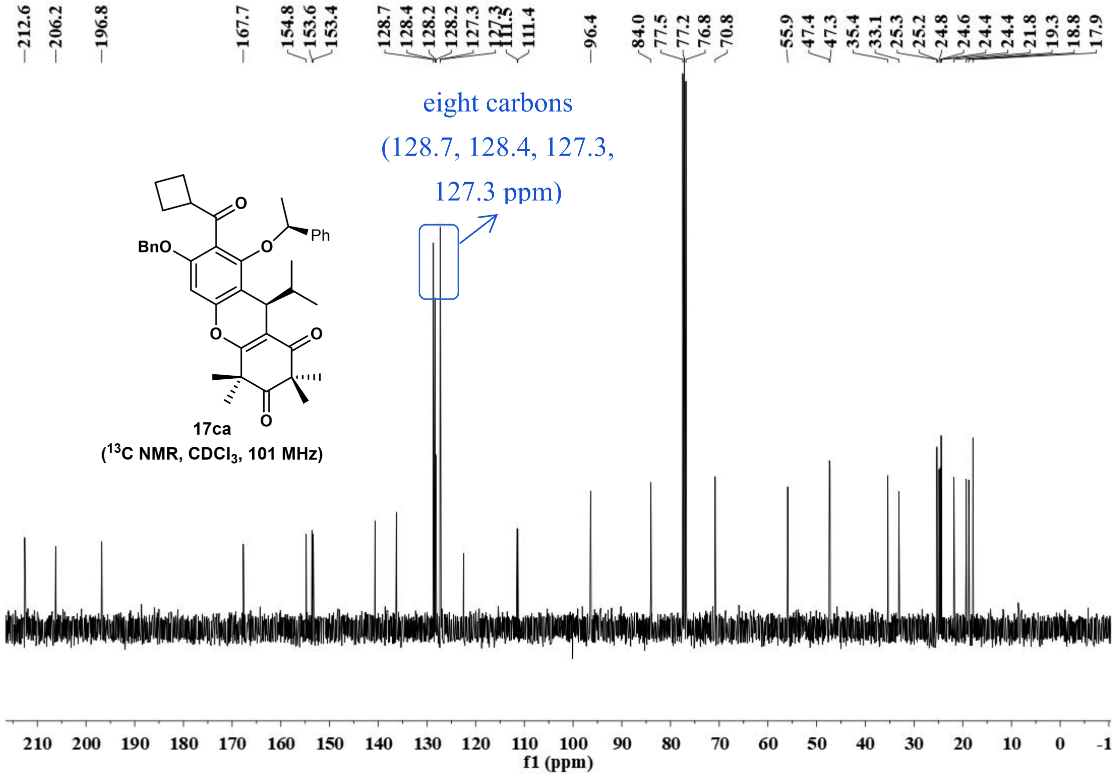

Supplementary Figure 132. ^{13}**C NMR spectra of 17ca.**

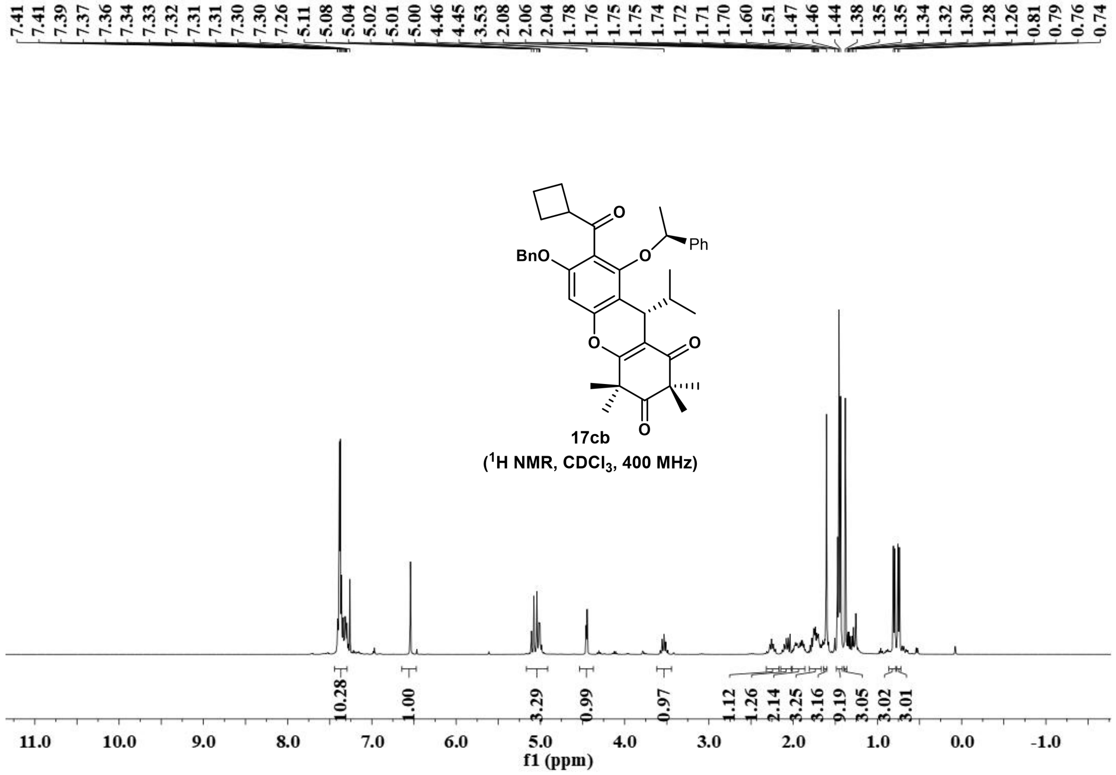

Supplementary Figure 133. ^{1}**H NMR spectra of 17cb.**

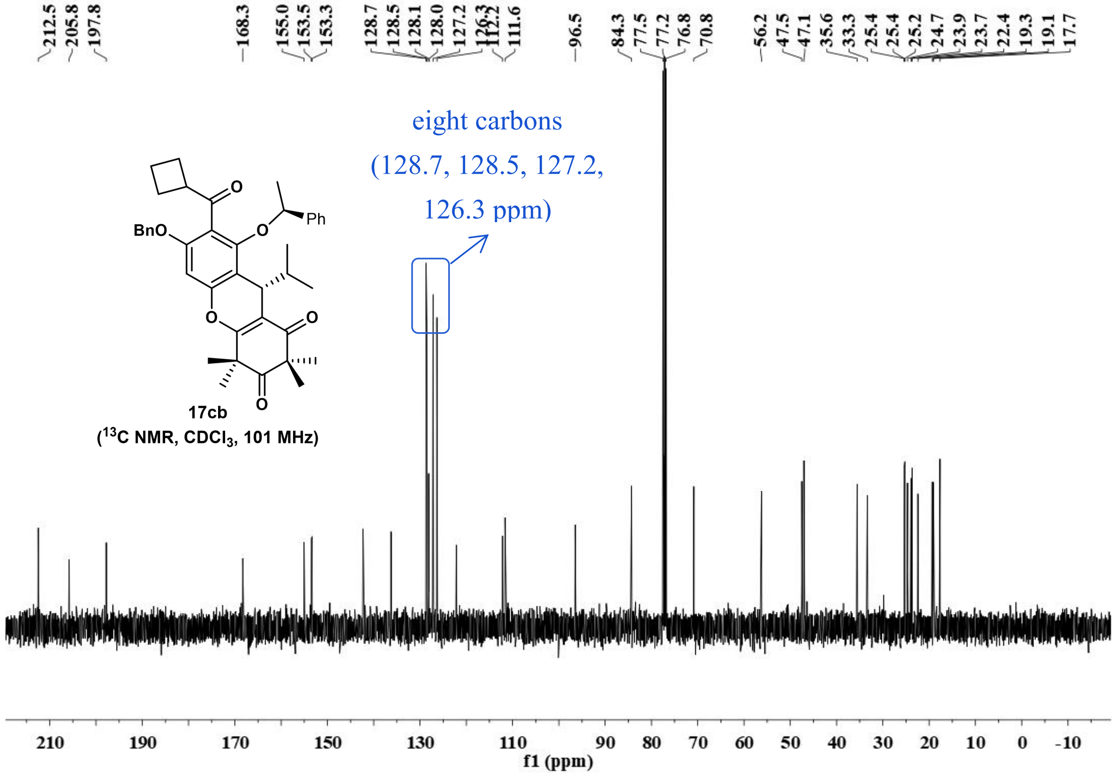

Supplementary Figure 134. ^{13}C NMR spectra of 17cb.

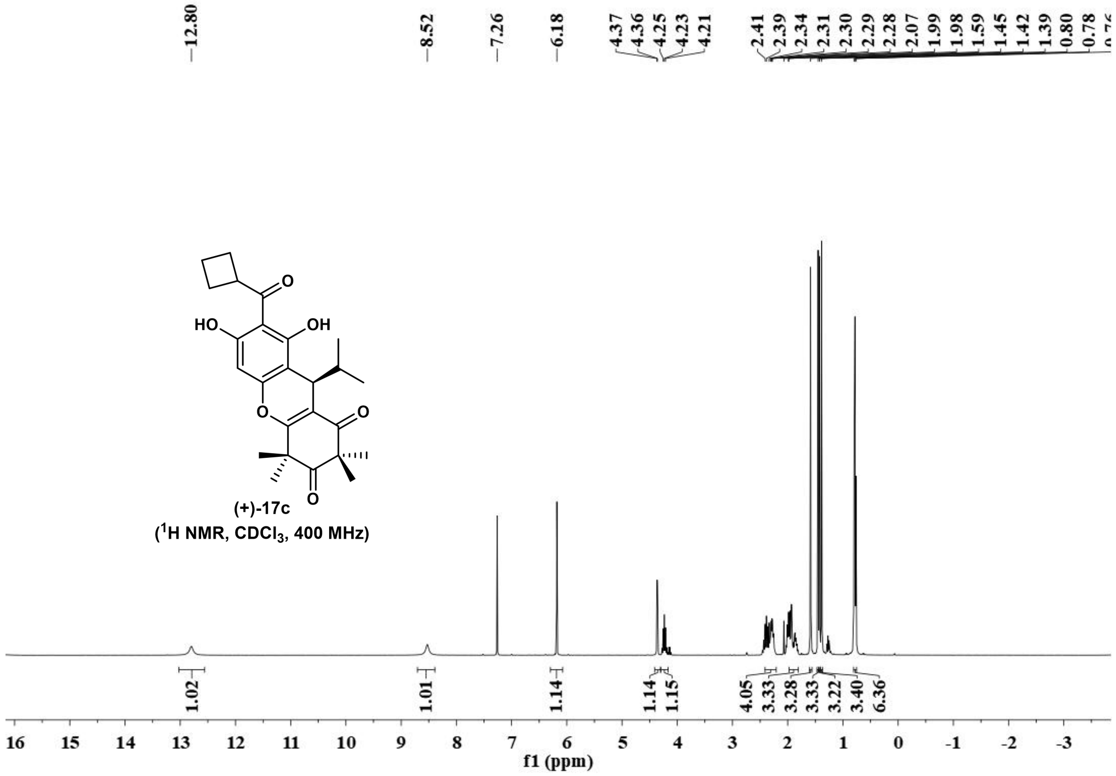

Supplementary Figure 135. ^{1}H NMR spectra of (+)-17c.

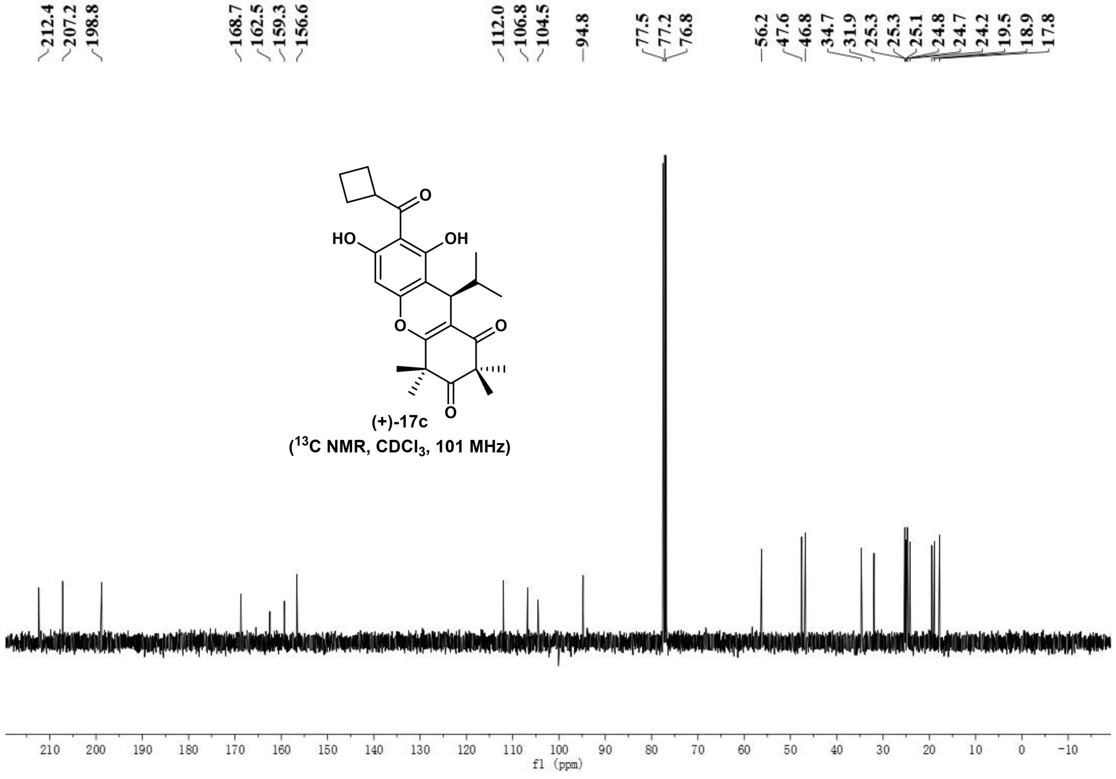

Supplementary Figure 136. ^{13}C NMR spectra of (+)-17c.

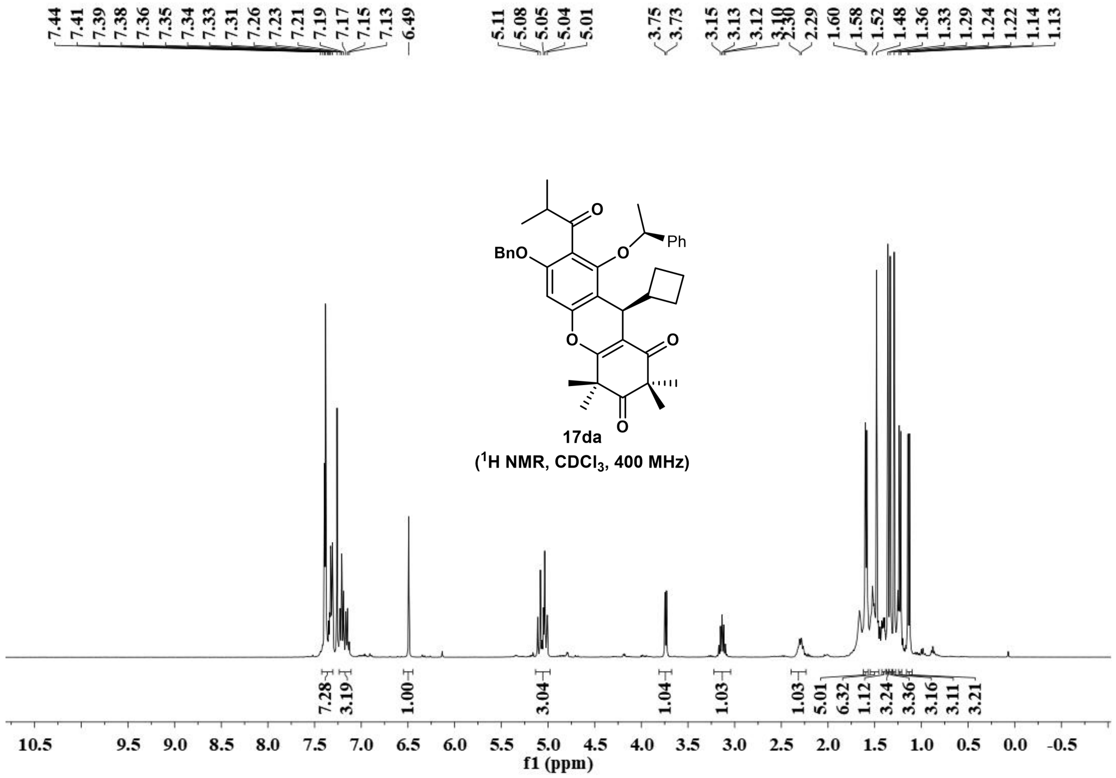

Supplementary Figure 137. ^{1}H NMR spectra of 17da.

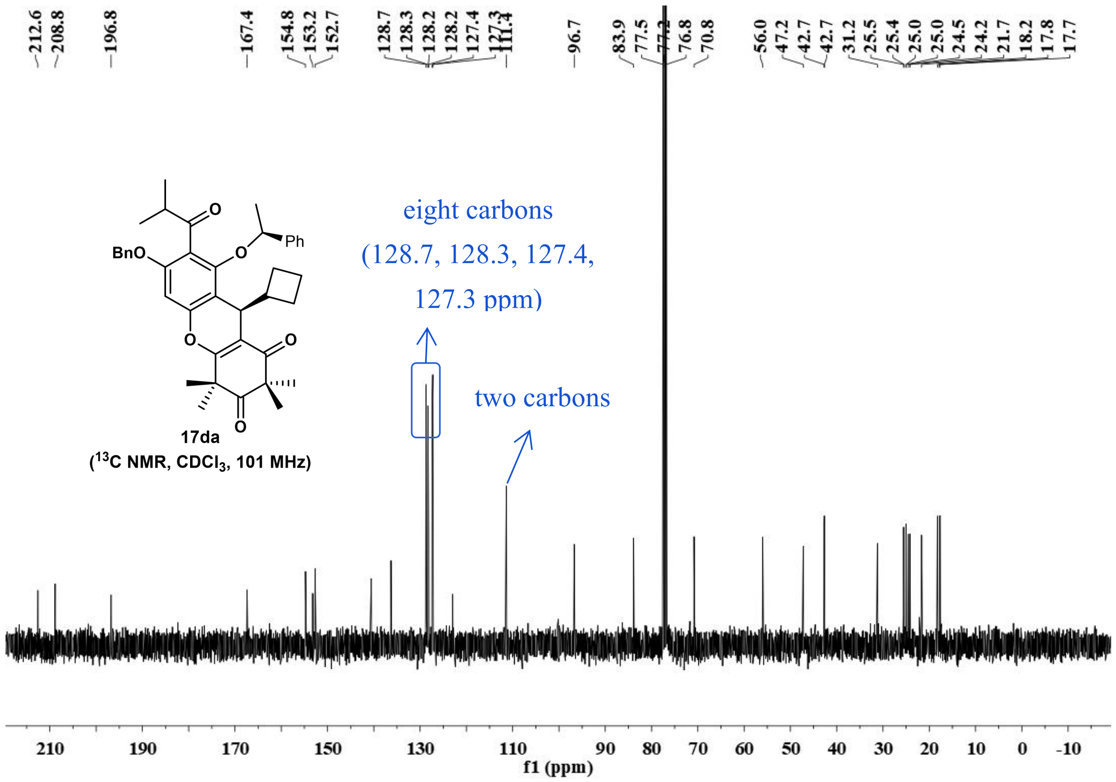

Supplementary Figure 138. ^{13}C NMR spectra of 17da.

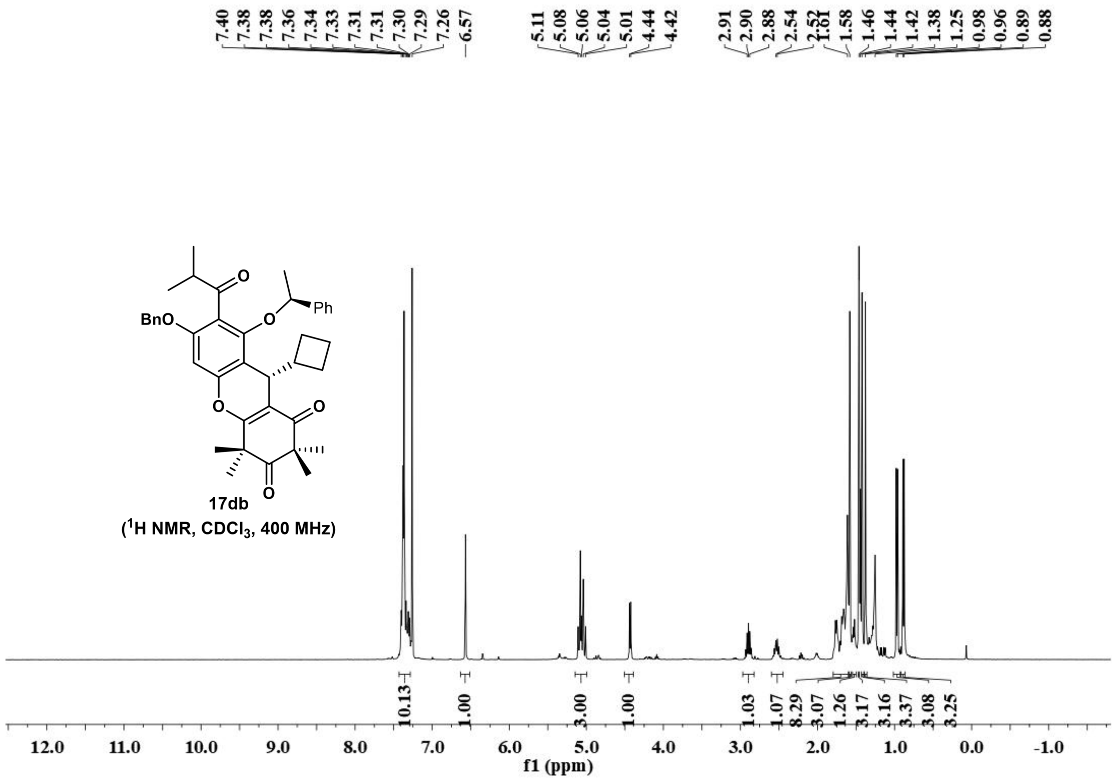

Supplementary Figure 139. ^{1}H NMR spectra of 17db.

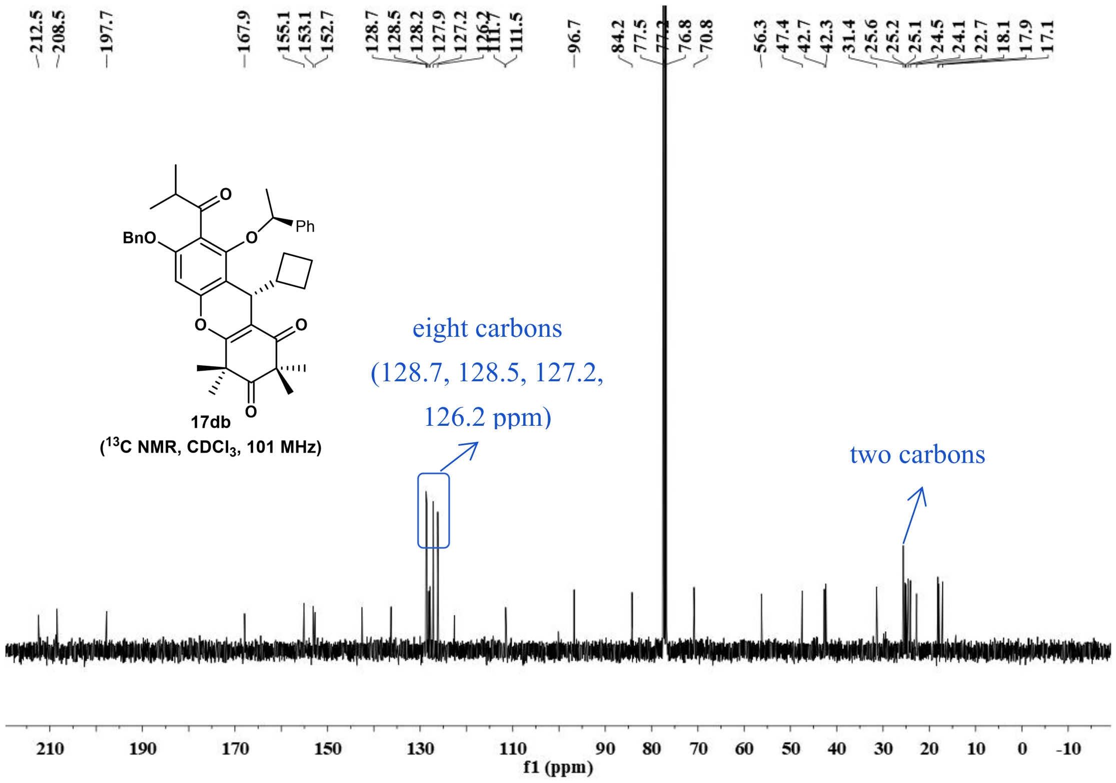

Supplementary Figure 140. ^{13}C NMR spectra of 17db.

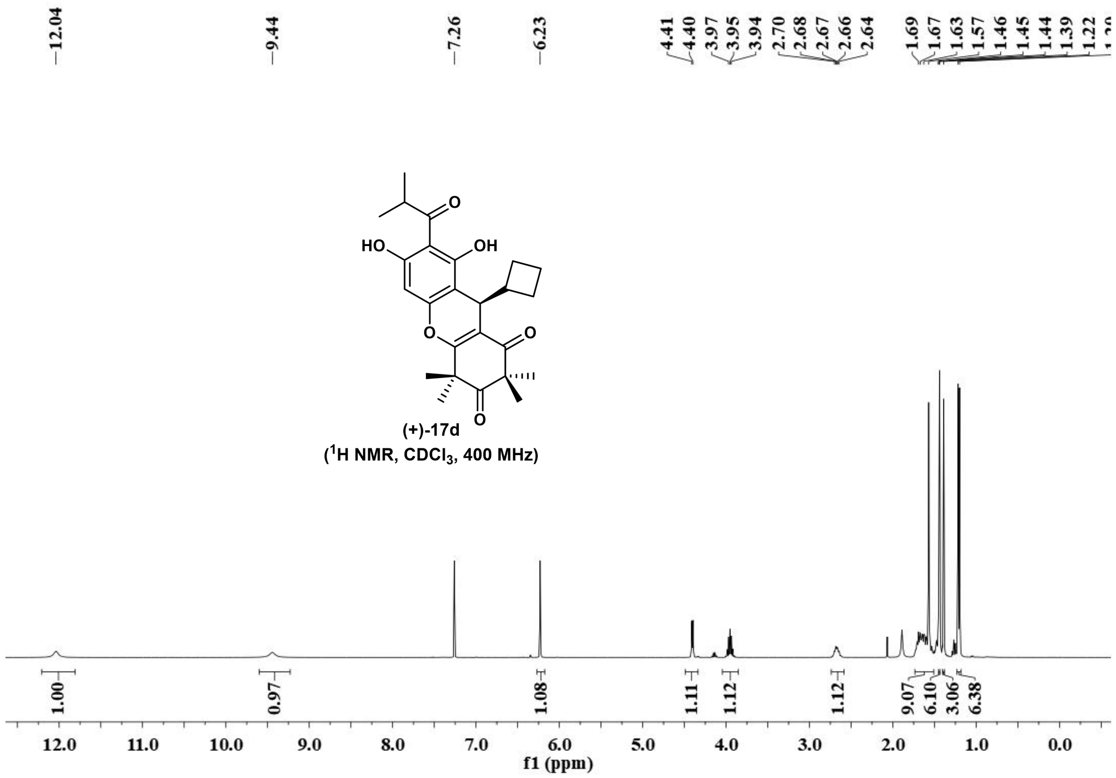

Supplementary Figure 141. ^{1}H NMR spectra of (+)-17d.

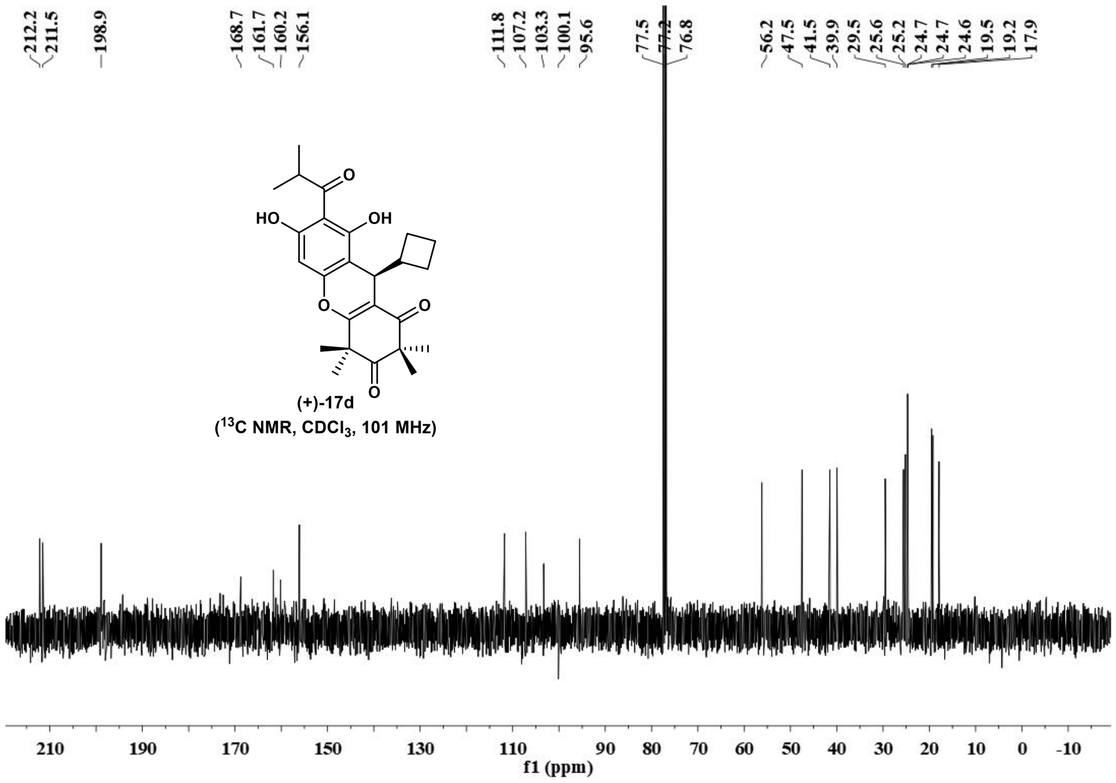

Supplementary Figure 142. ^{1}H NMR spectra of (+)-17d.

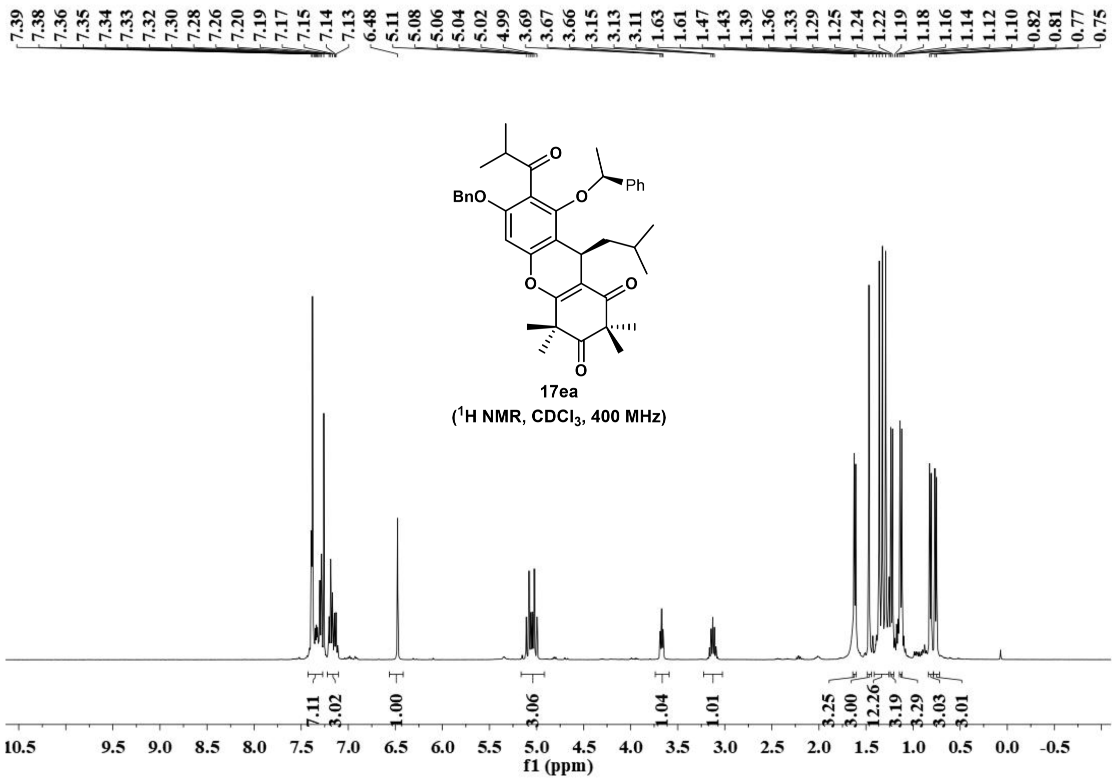

Supplementary Figure 143. ^{1}H NMR spectra of 17ea.

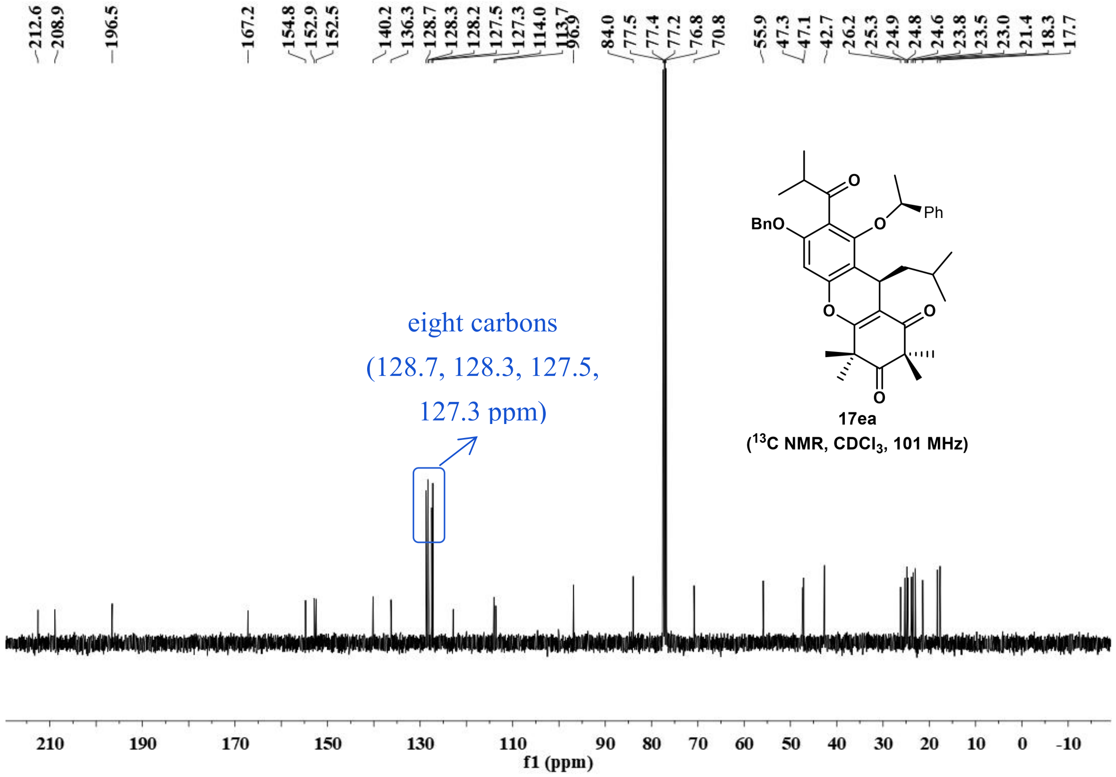

Supplementary Figure 144. ^{13}C NMR spectra of 17ea.

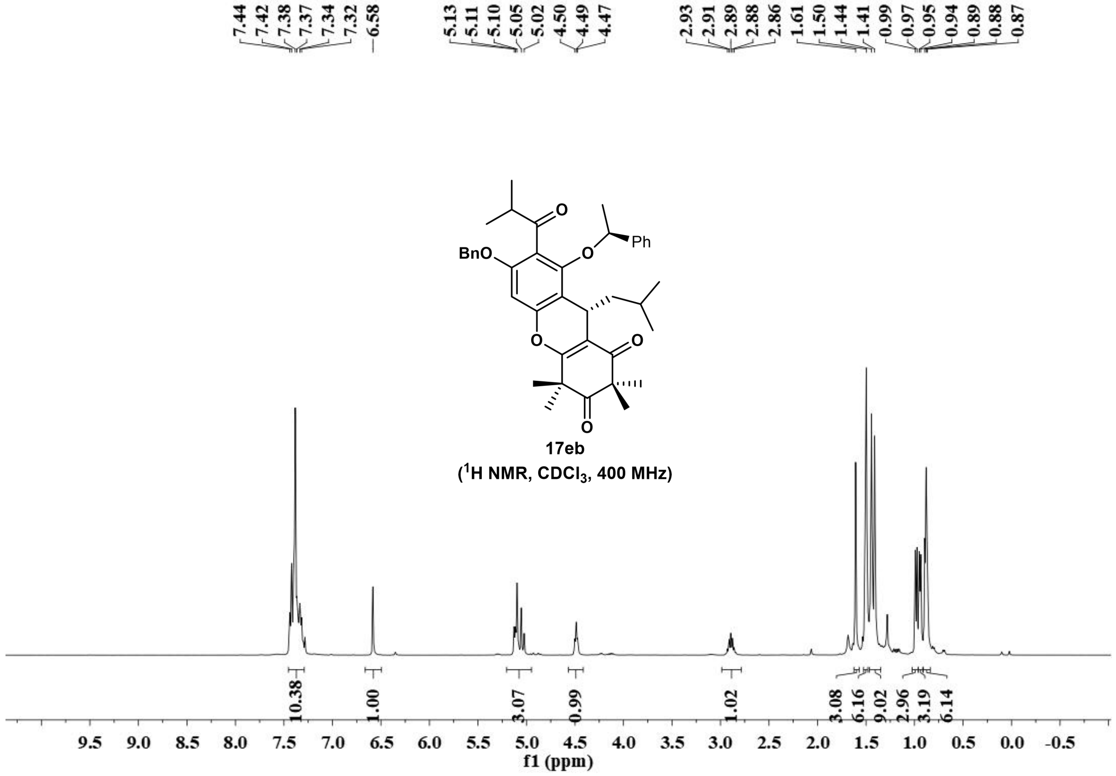

Supplementary Figure 145. ^{1}H NMR spectra of 17eb.

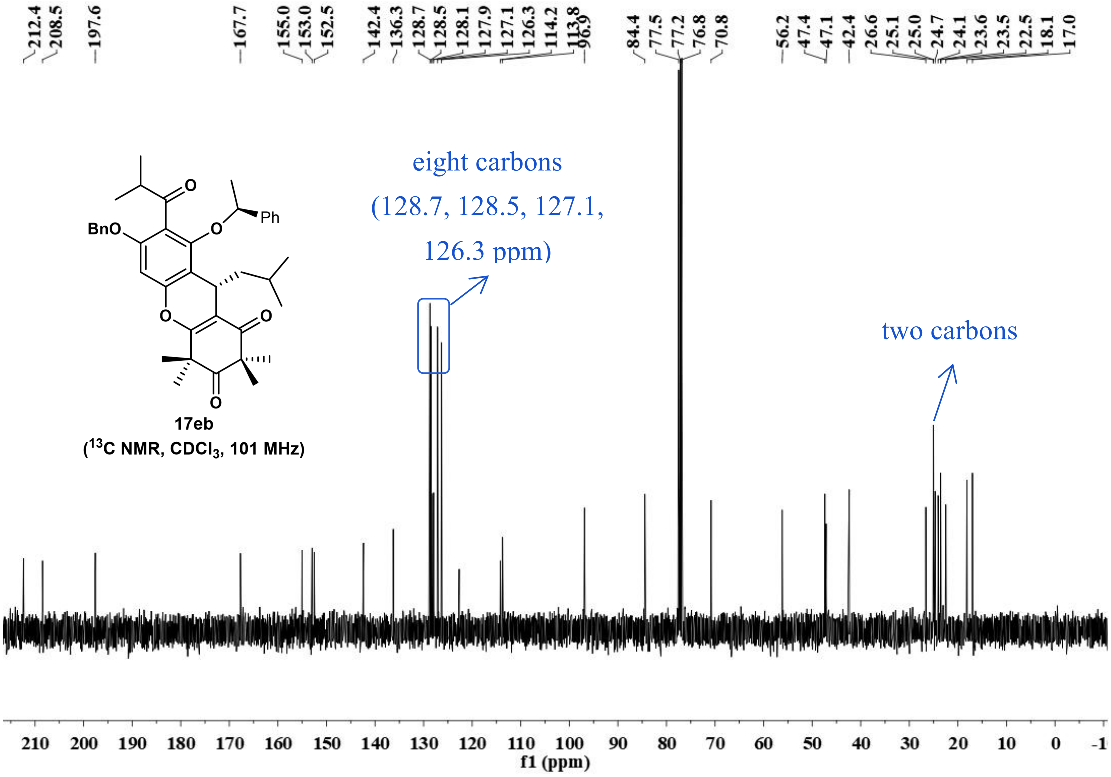

Supplementary Figure 146. ^{13}C NMR spectra of 17eb.

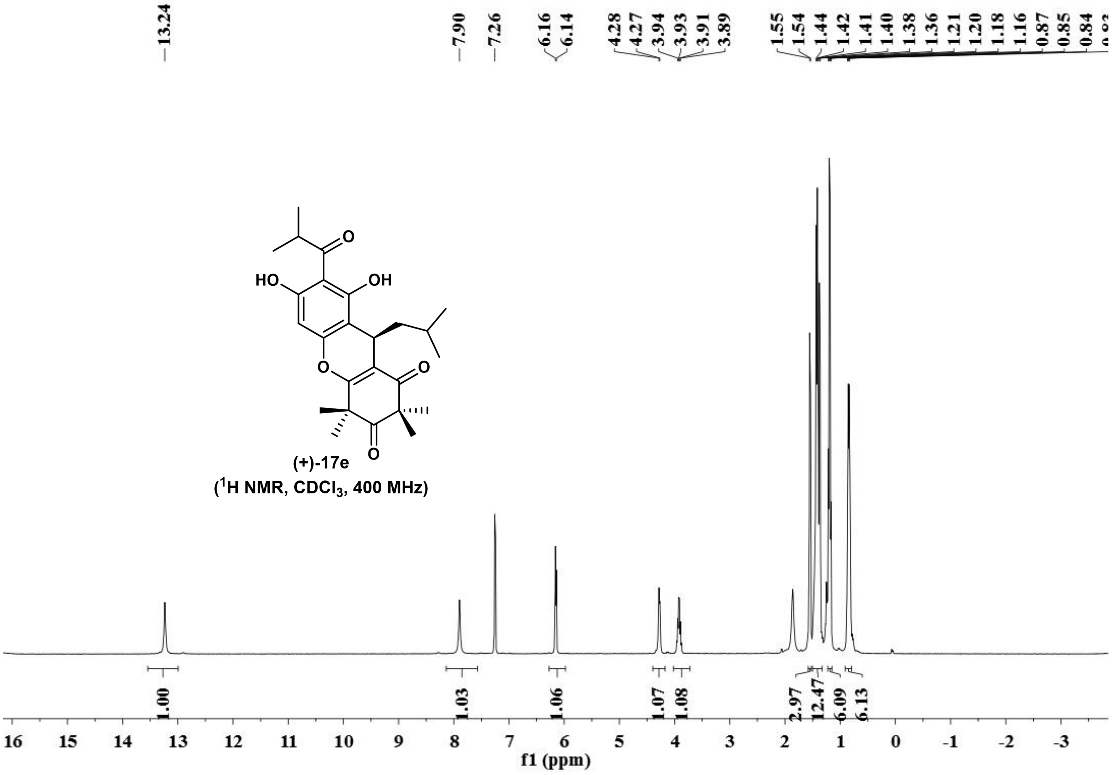

Supplementary Figure 147. ^{1}H NMR spectra of (+)-17e.

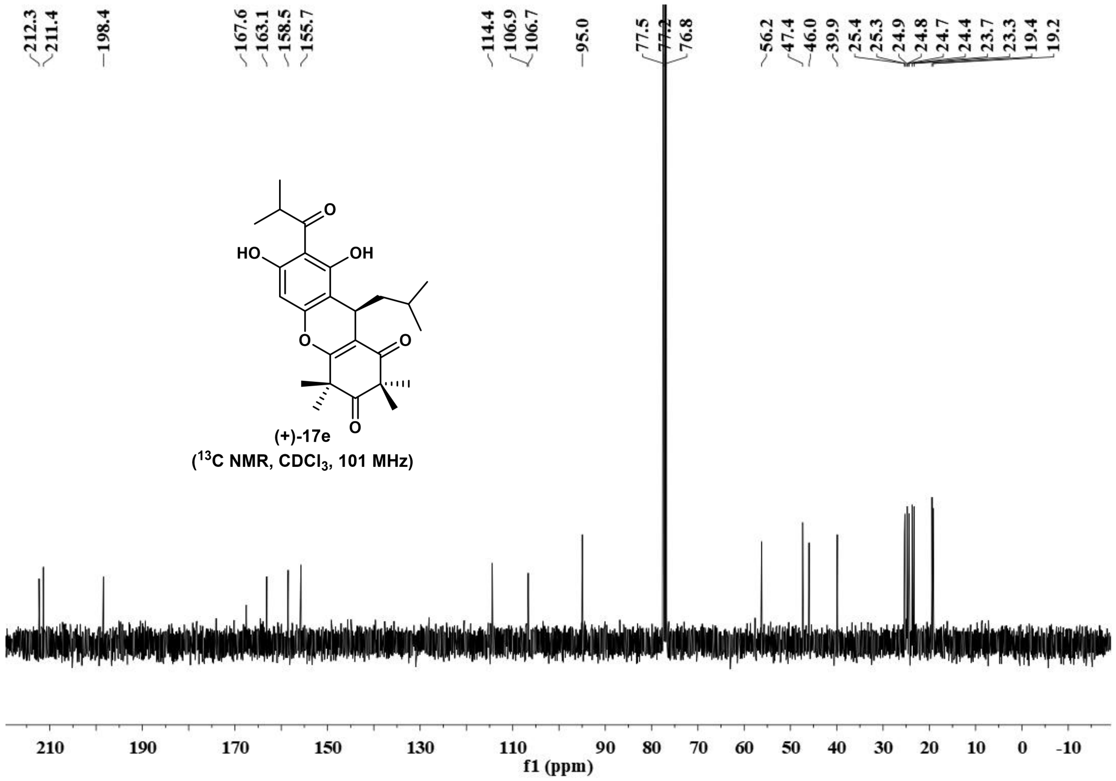

Supplementary Figure 148. ^{13}C NMR spectra of (+)-17e.

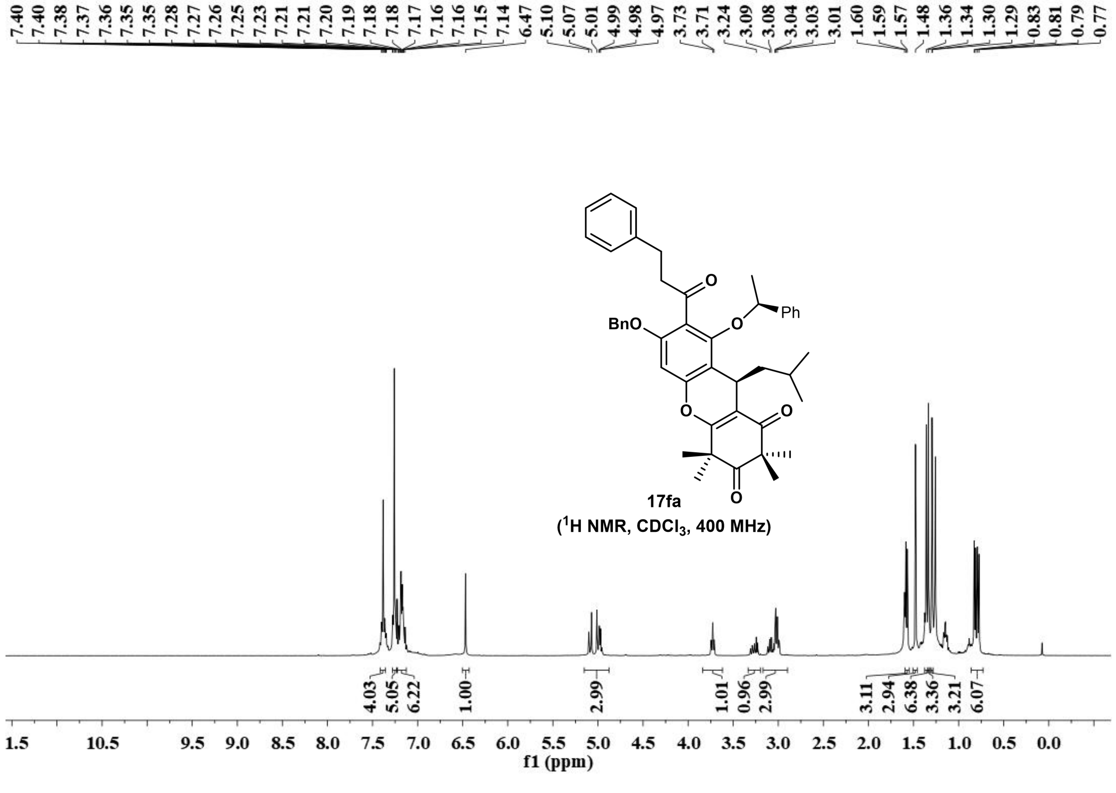

Supplementary Figure 149. ^{1}H NMR spectra of 17fa.

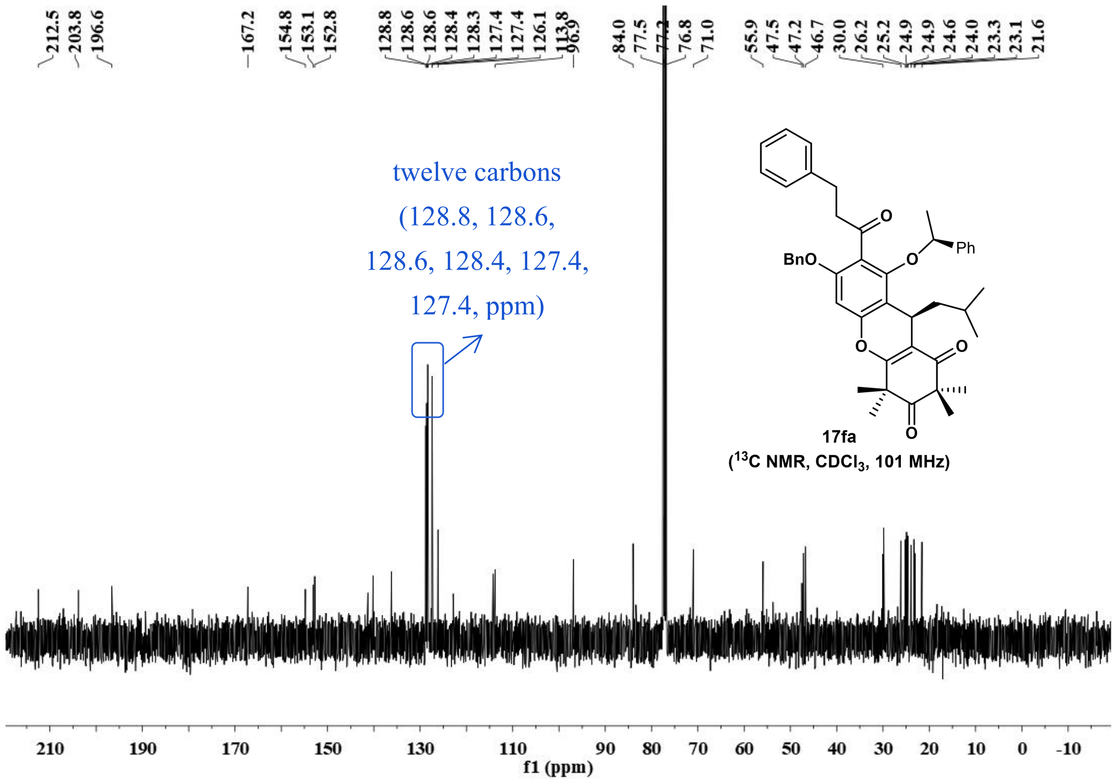

Supplementary Figure 150. ^{13}C NMR spectra of 17fa.

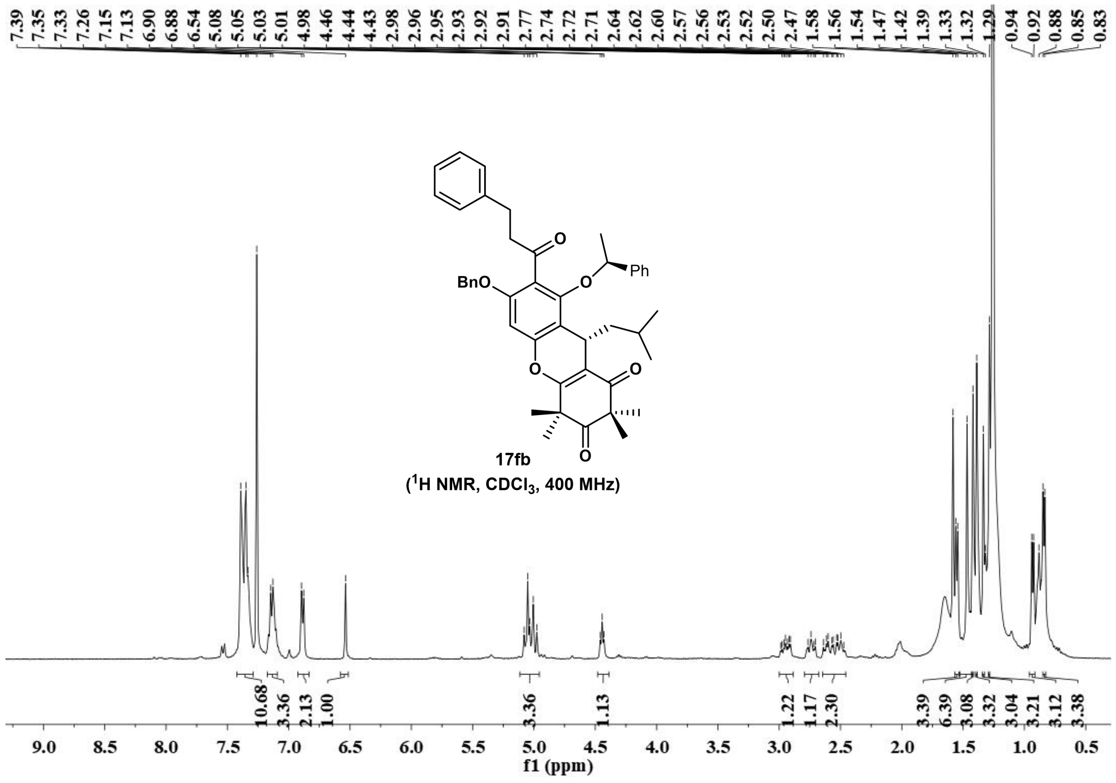

Supplementary Figure 151. ^{1}H NMR spectra of 17fb.

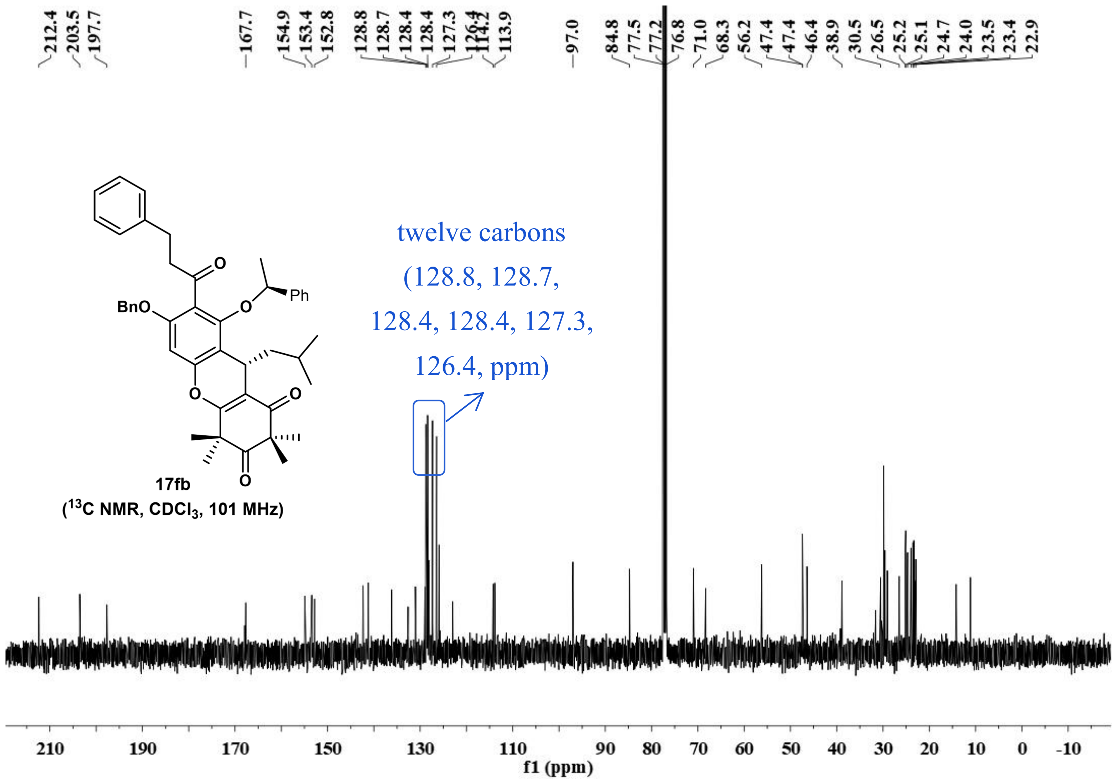

Supplementary Figure 152. ^{13}**C NMR spectra of 17fb.**

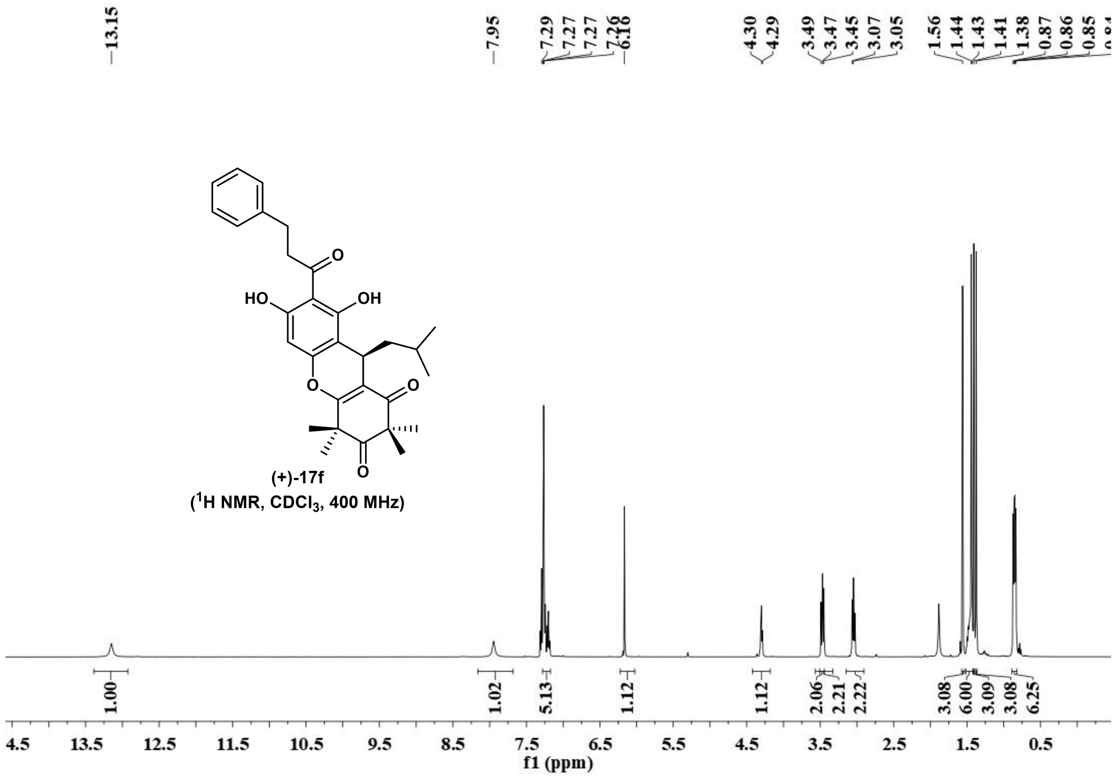

Supplementary Figure 153. ^{1}**H NMR spectra of (+)-17f.**

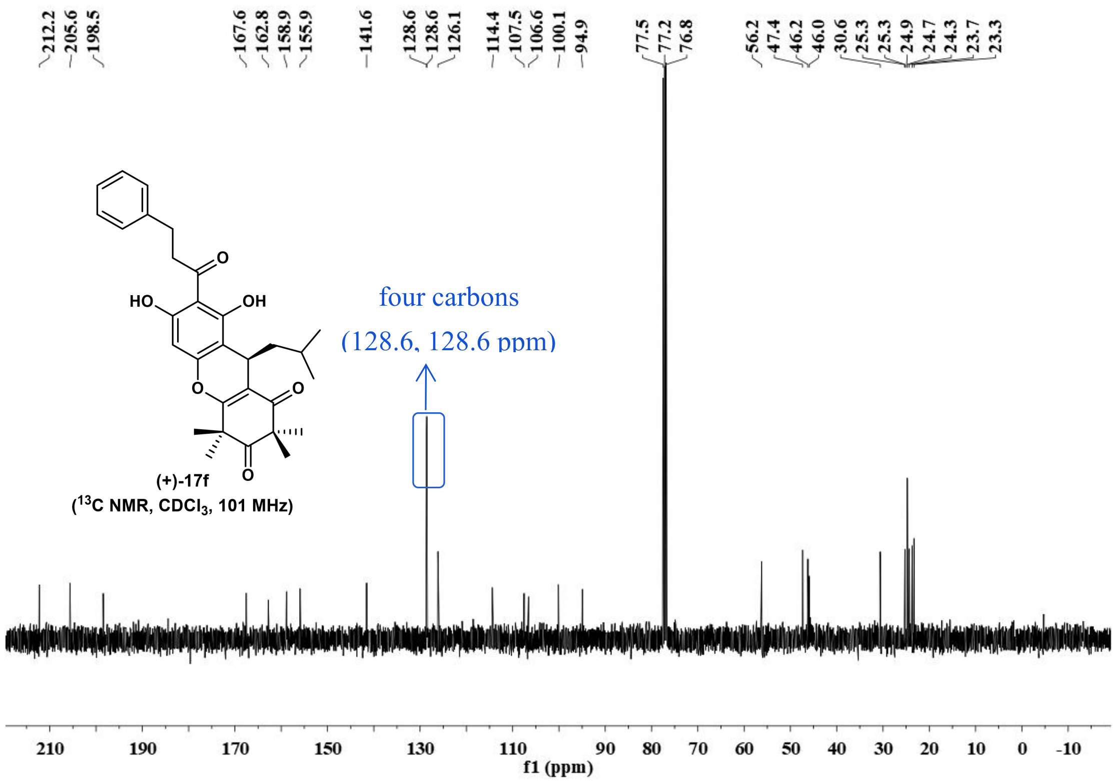

Supplementary Figure 154. ^{13}**C NMR spectra of (+)-17f.**

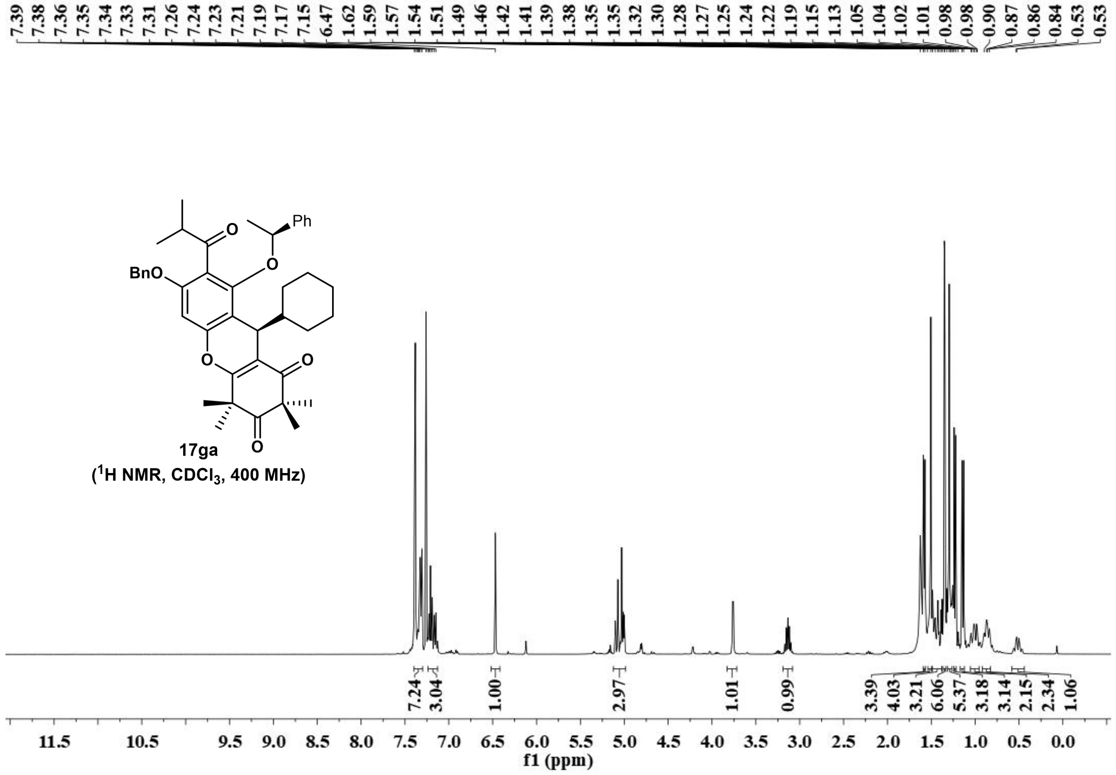

Supplementary Figure 155. ^{1}**H NMR spectra of 17ga.**

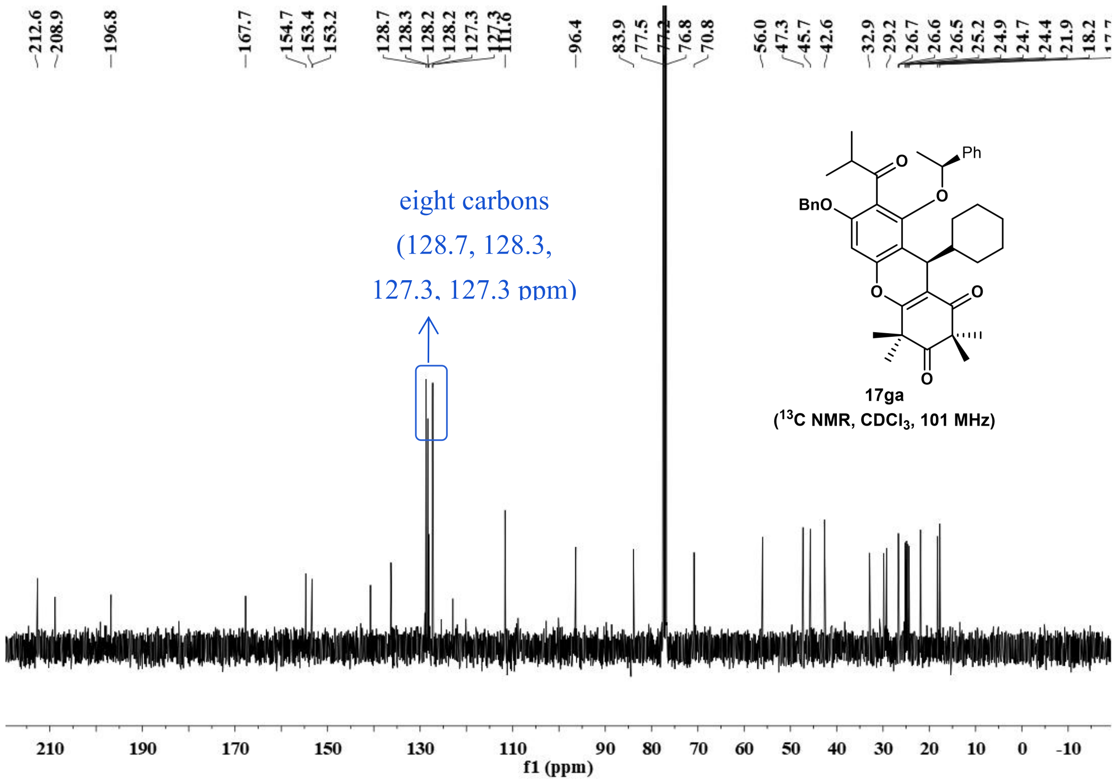

Supplementary Figure 156. ^{13}C NMR spectra of 17ga.

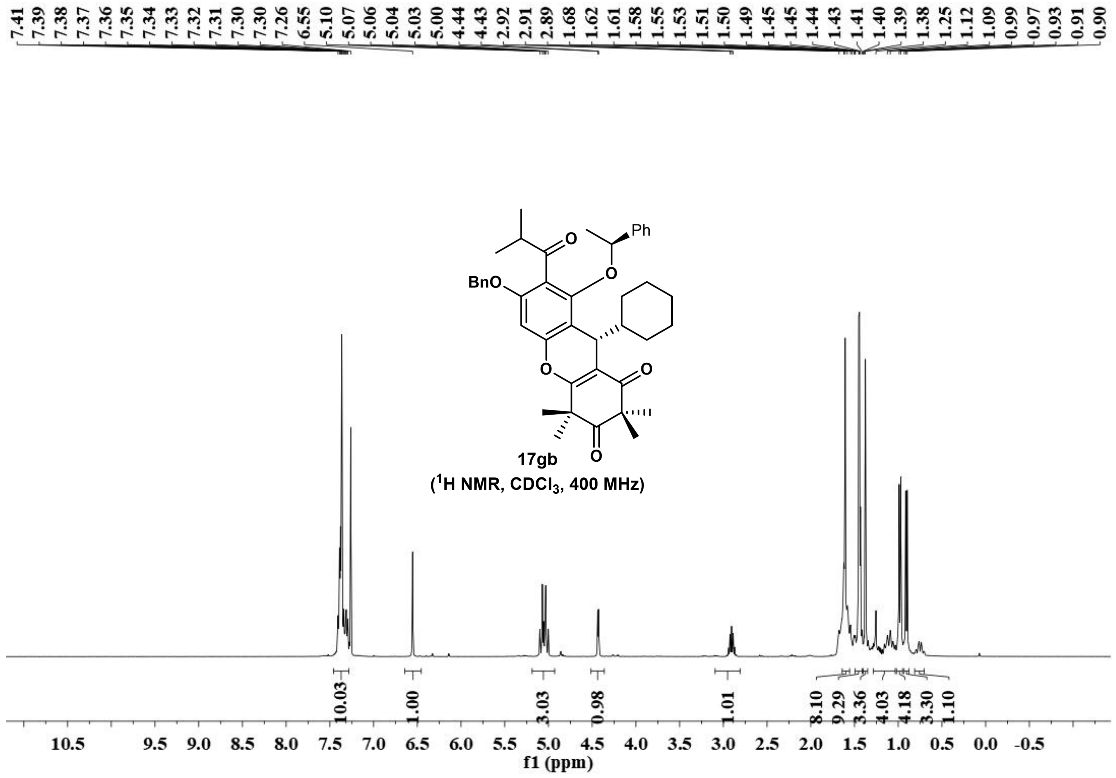

Supplementary Figure 157. ^{1}H NMR spectra of 17gb.

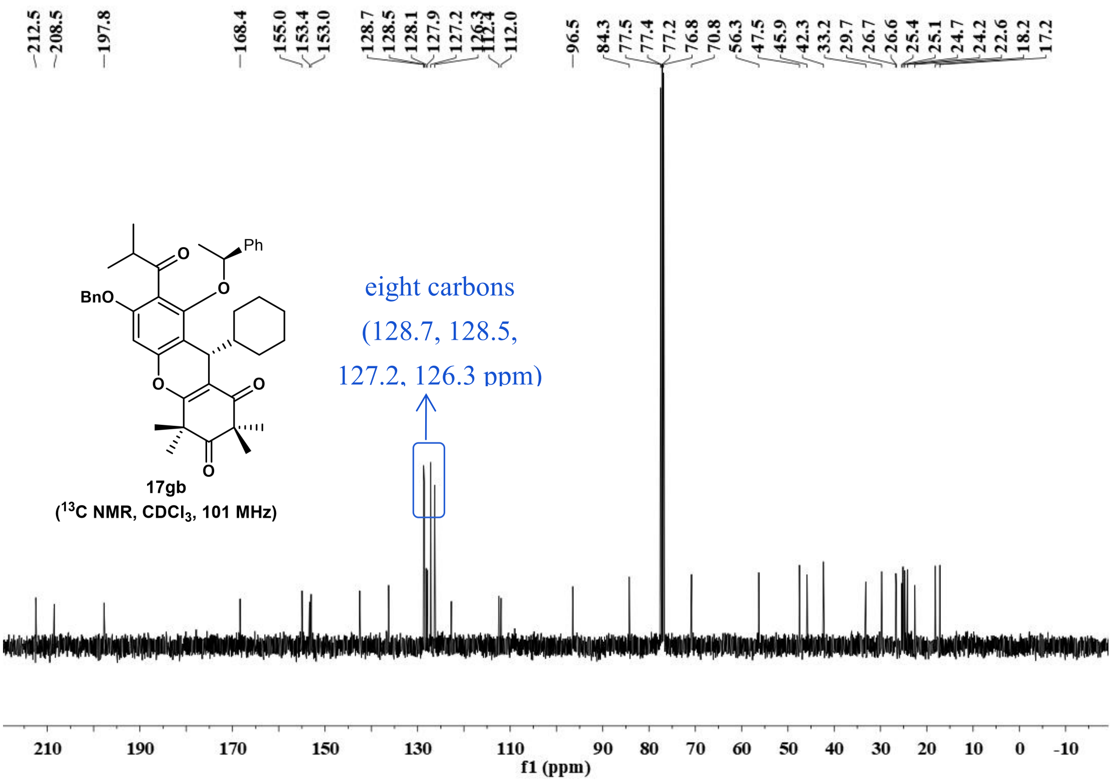

Supplementary Figure 158. ^{13}C NMR spectra of 17gb.

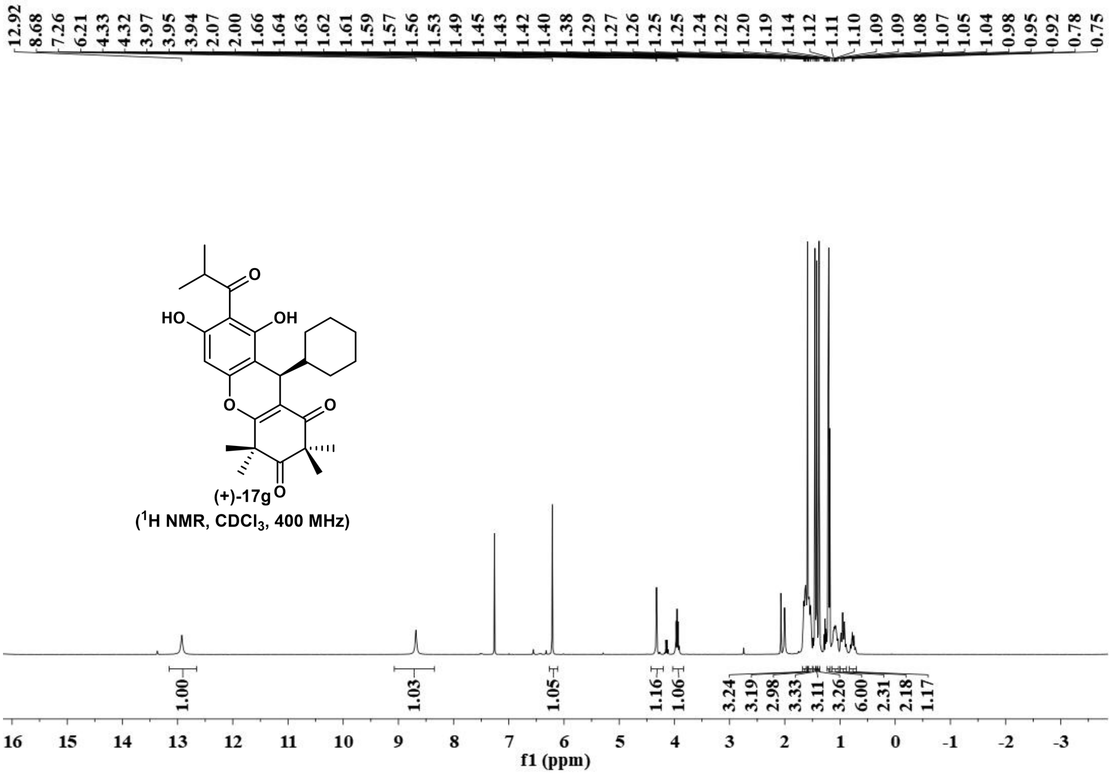

Supplementary Figure 159. ^{1}H NMR spectra of (+)-17g.

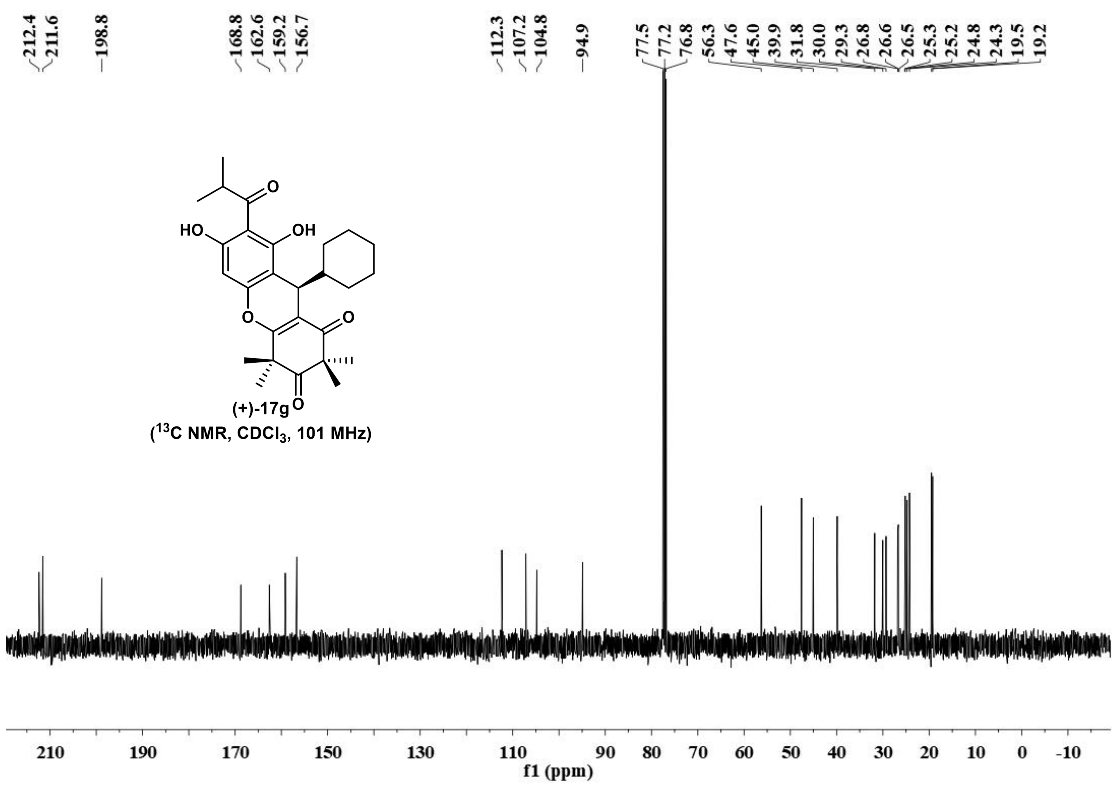

Supplementary Figure 160. ^{13}C NMR spectra of (+)-17g.

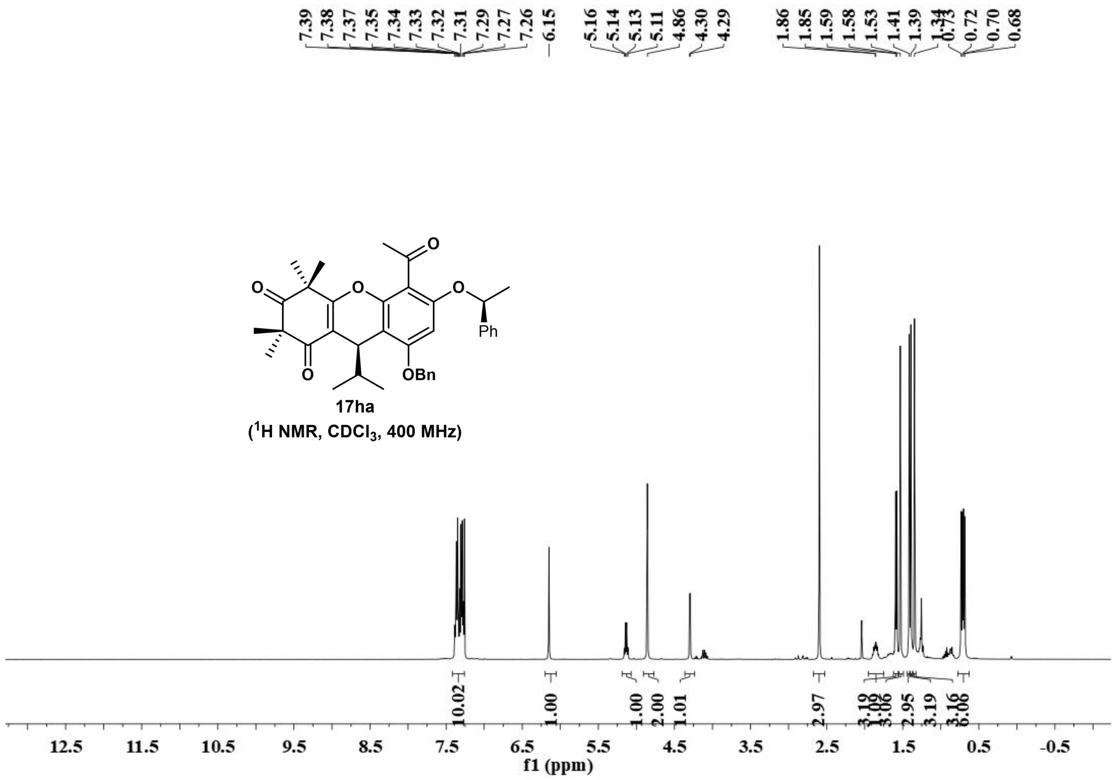

Supplementary Figure 161. ^{1}H NMR spectra of 17ha.

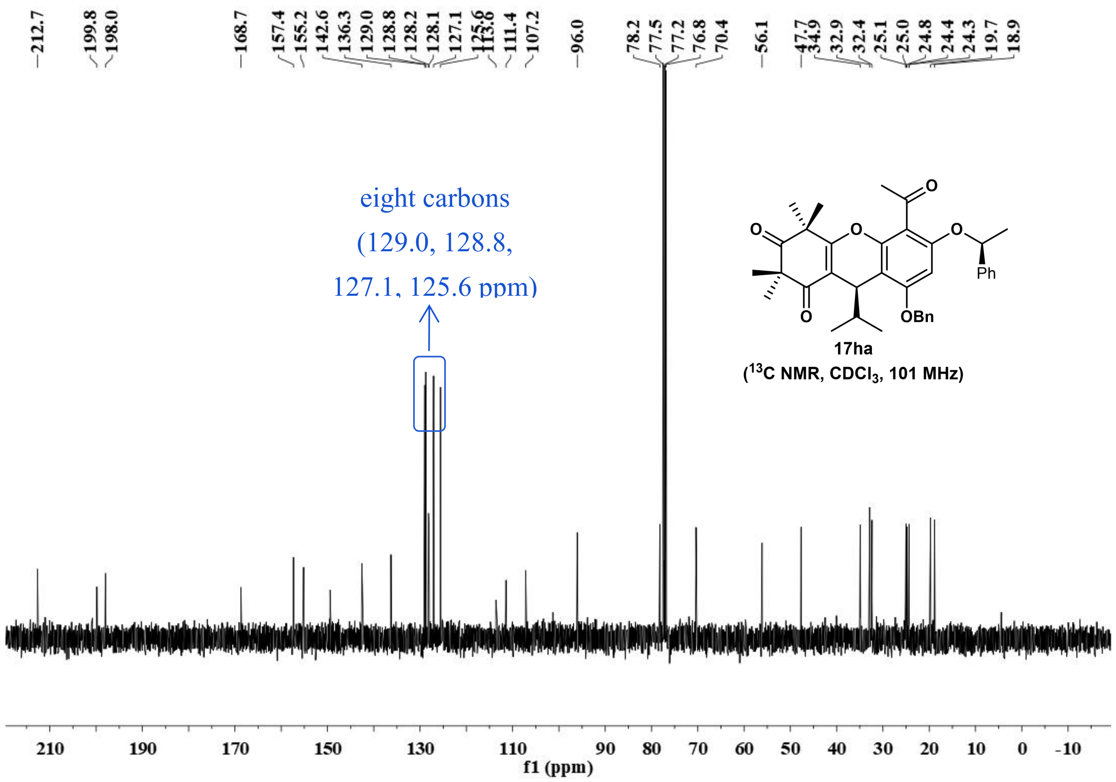

Supplementary Figure 162. ^{13}C NMR spectra of 17ha.

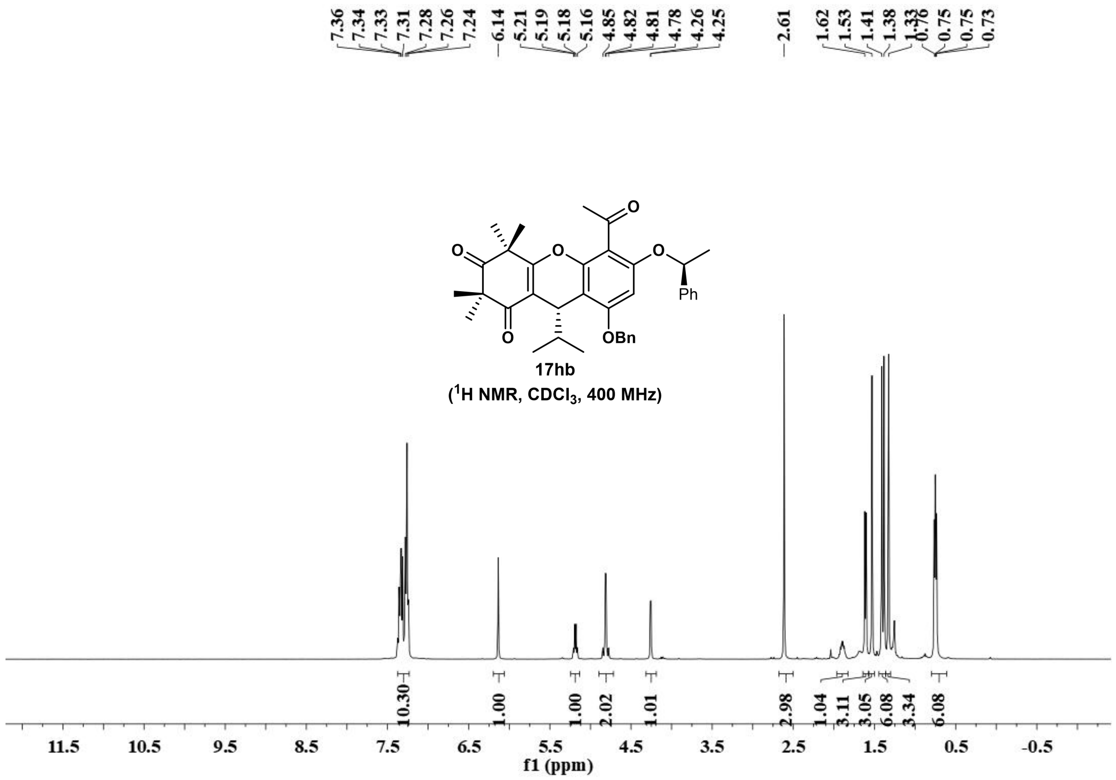

Supplementary Figure 163. ^{1}H NMR spectra of 17hb.

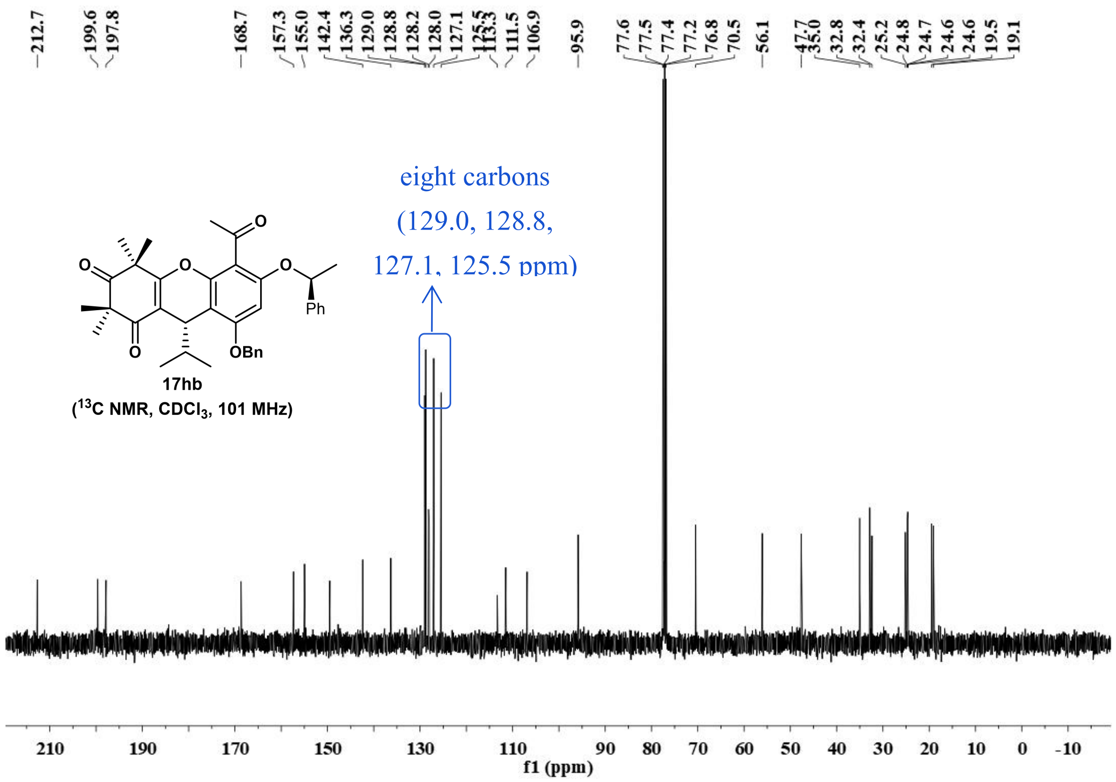

Supplementary Figure 164. ^{13}C NMR spectra of 17hb.

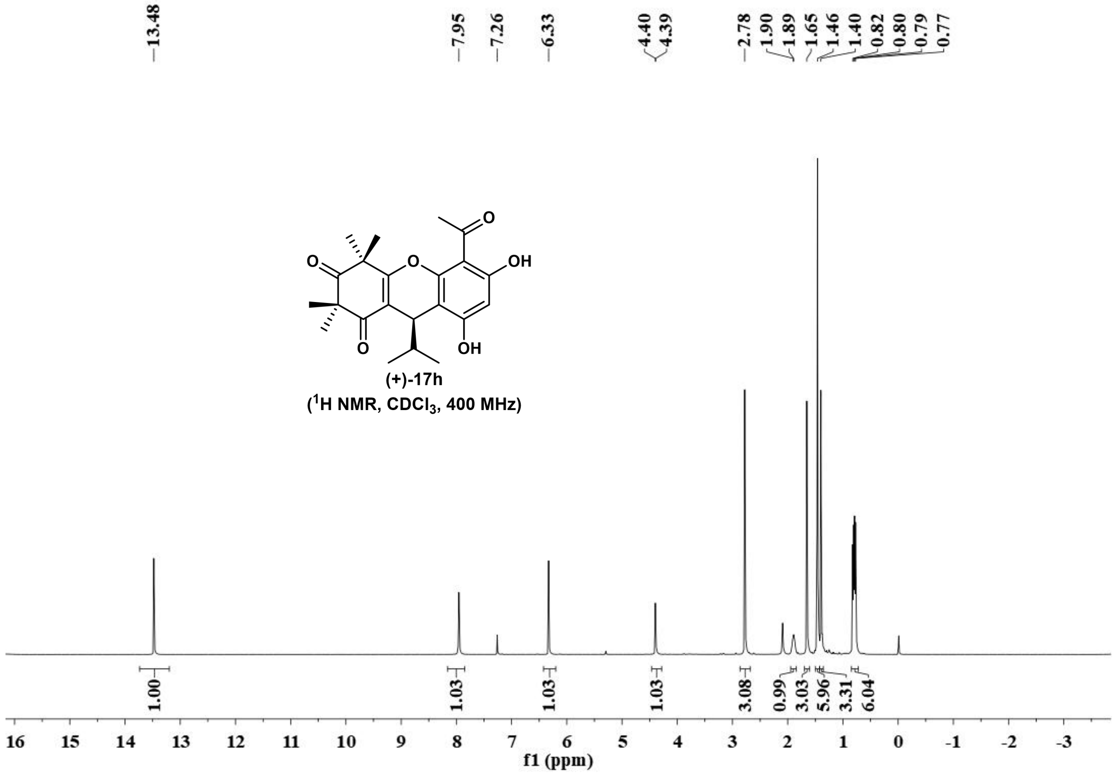

Supplementary Figure 165. ^{1}H NMR spectra of (+)-17h.

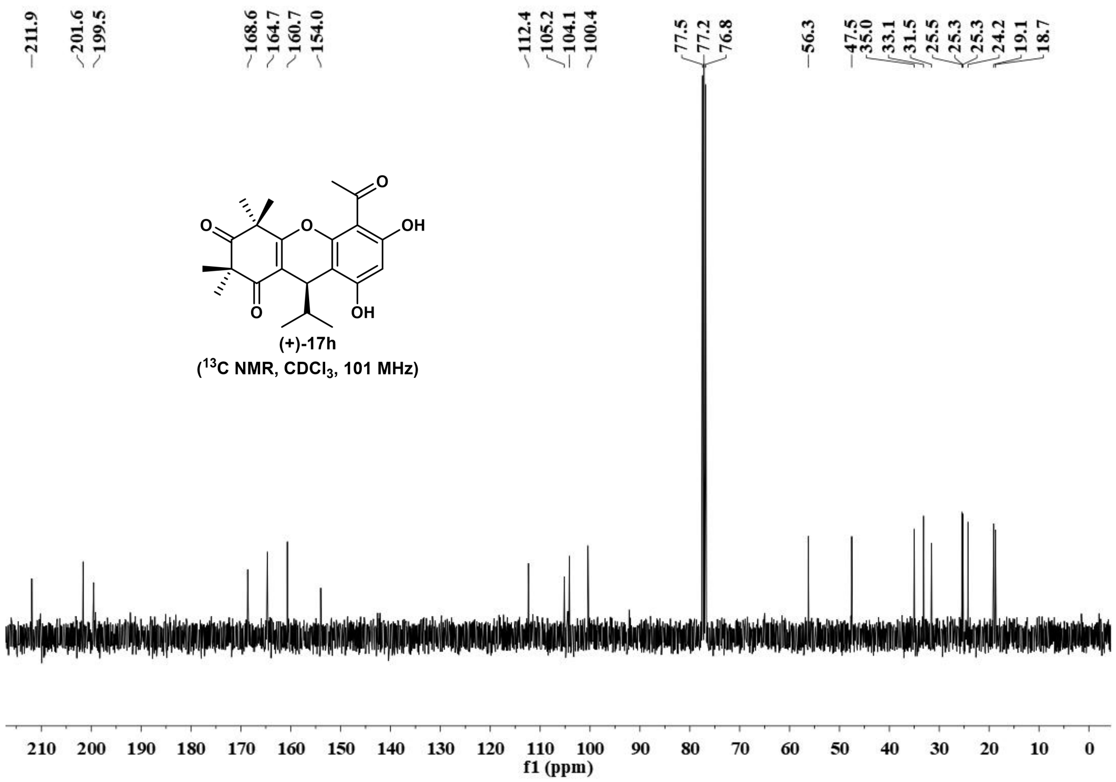

Supplementary Figure 166. ^{13}C NMR spectra of (+)-17h.

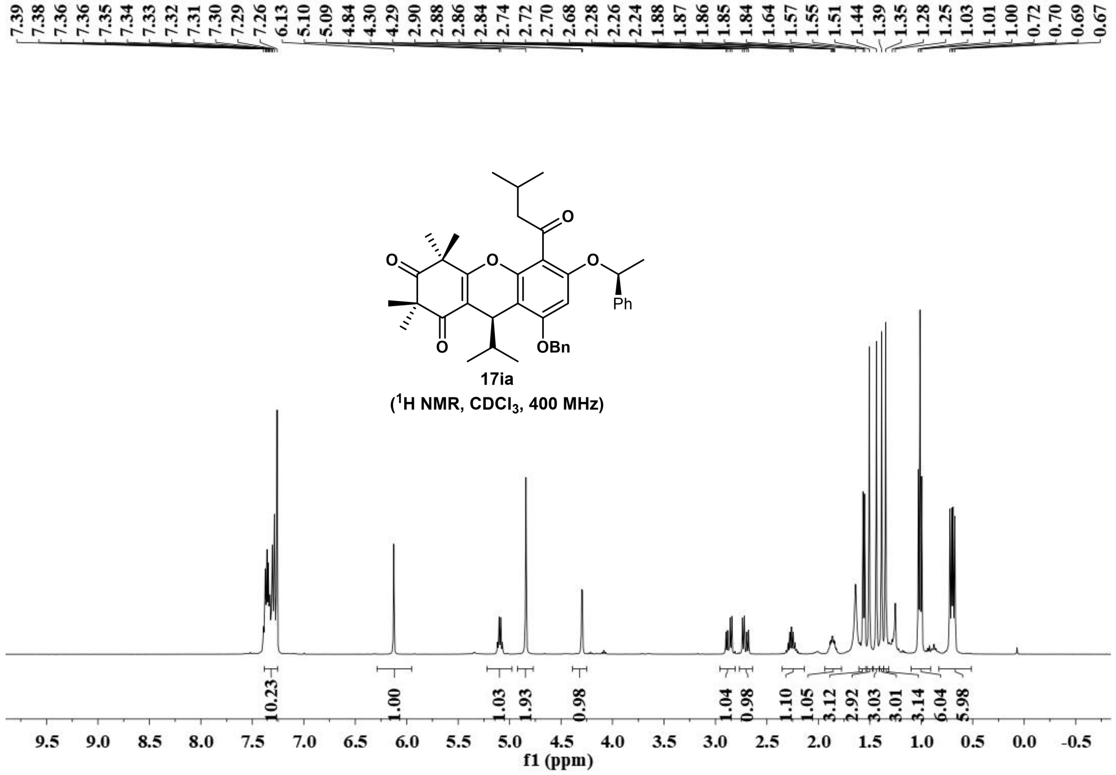

Supplementary Figure 167. ^{1}H NMR spectra of 17ia.

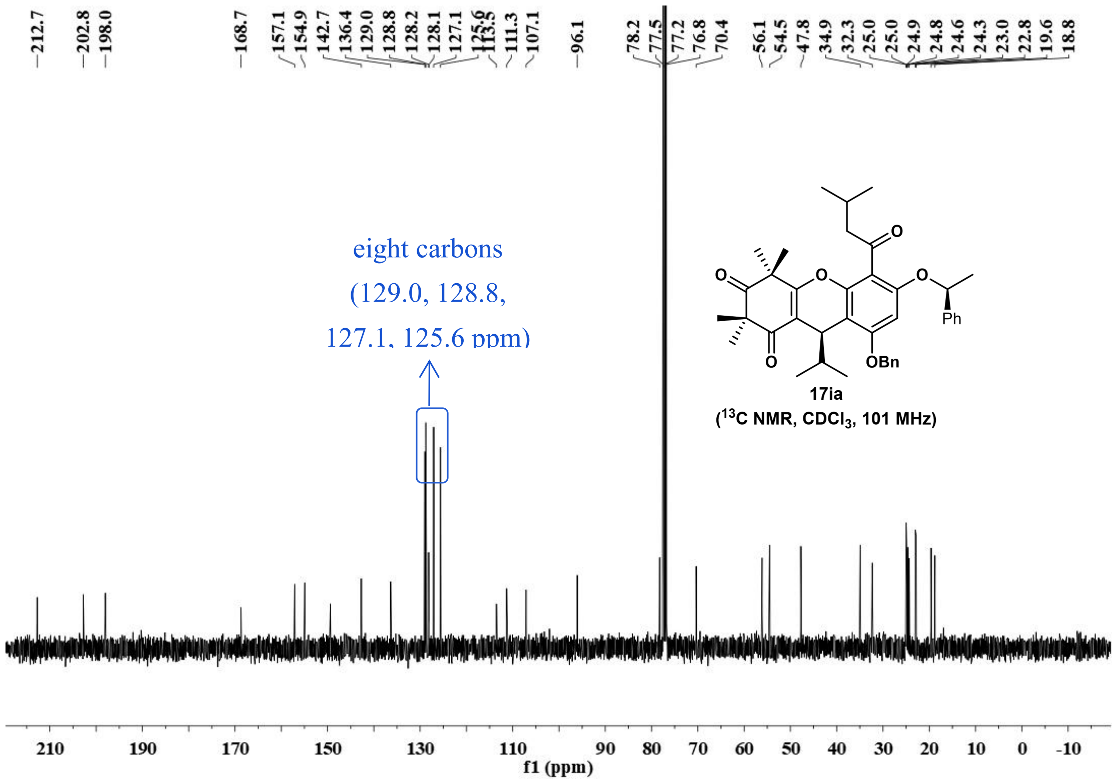

Supplementary Figure 168. ^{13}C NMR spectra of 17ia.

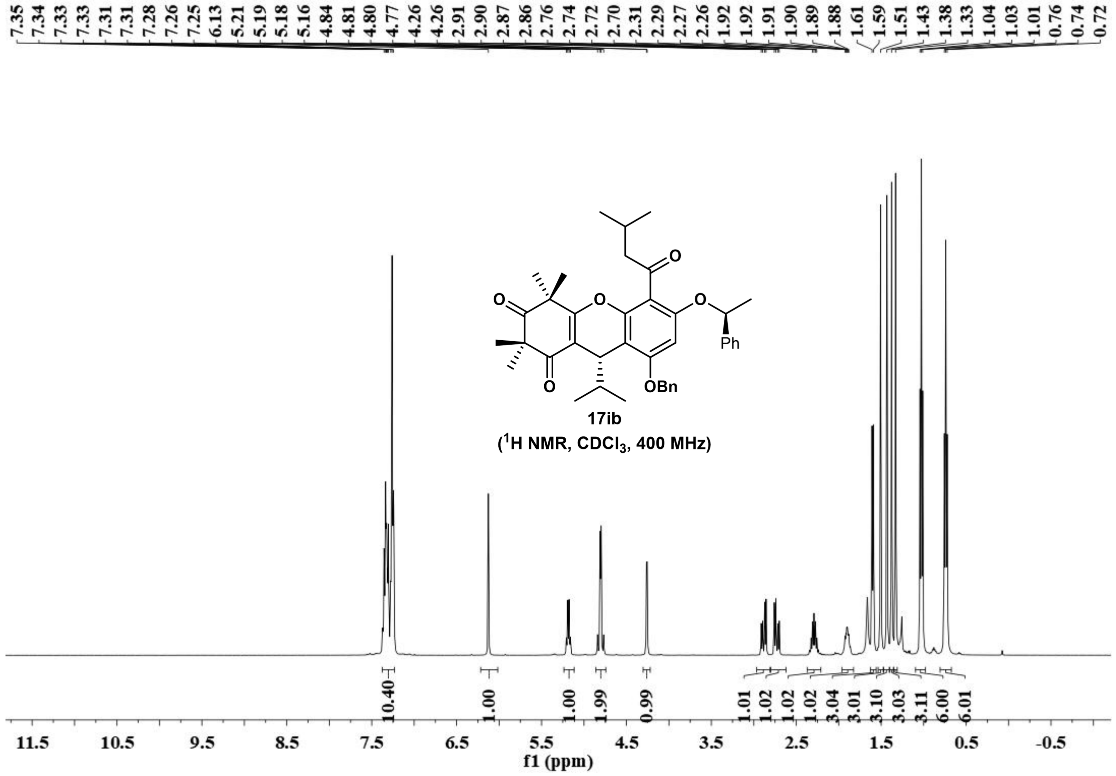

Supplementary Figure 169. ^{1}H NMR spectra of 17ib.

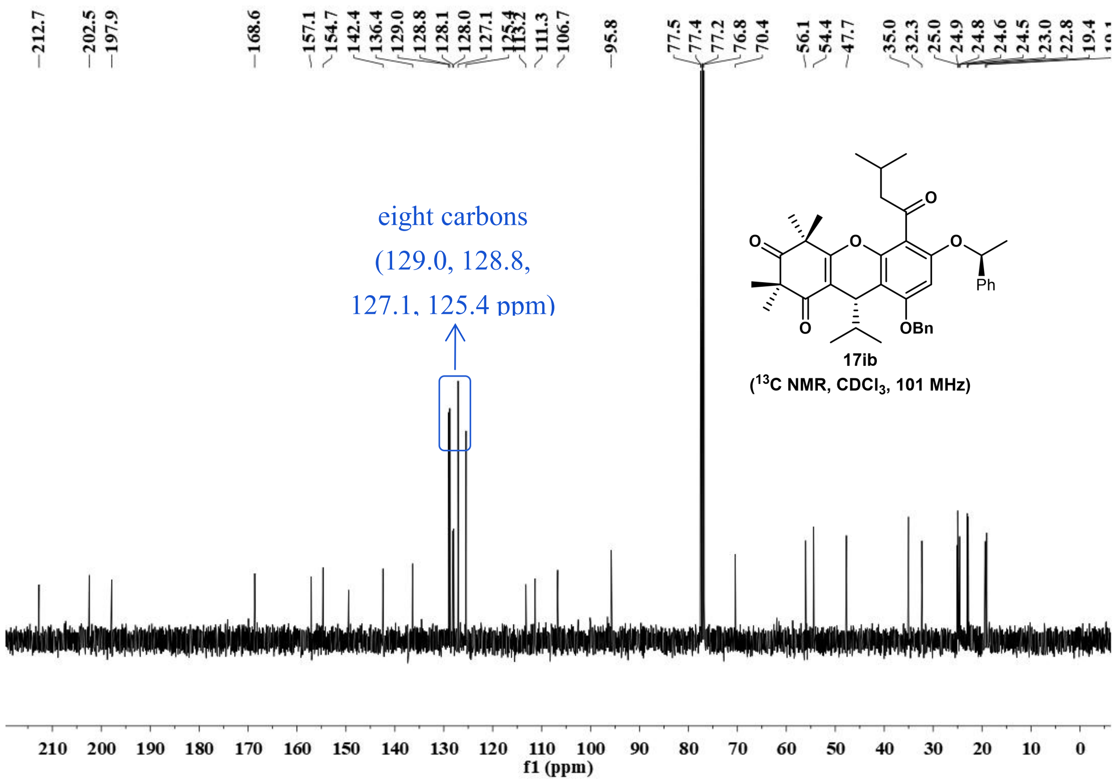

Supplementary Figure 170. ^{13}**C NMR spectra of 17ib.**

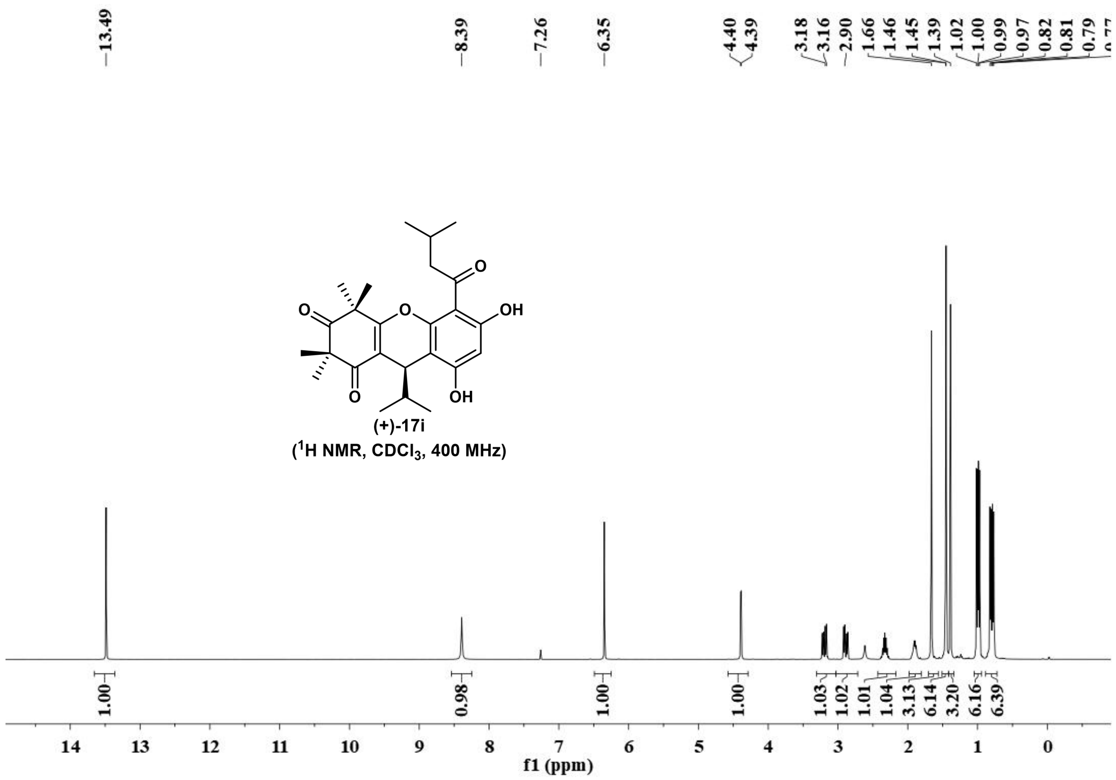

Supplementary Figure 171. ^{1}**H NMR spectra of (+)-17i.**

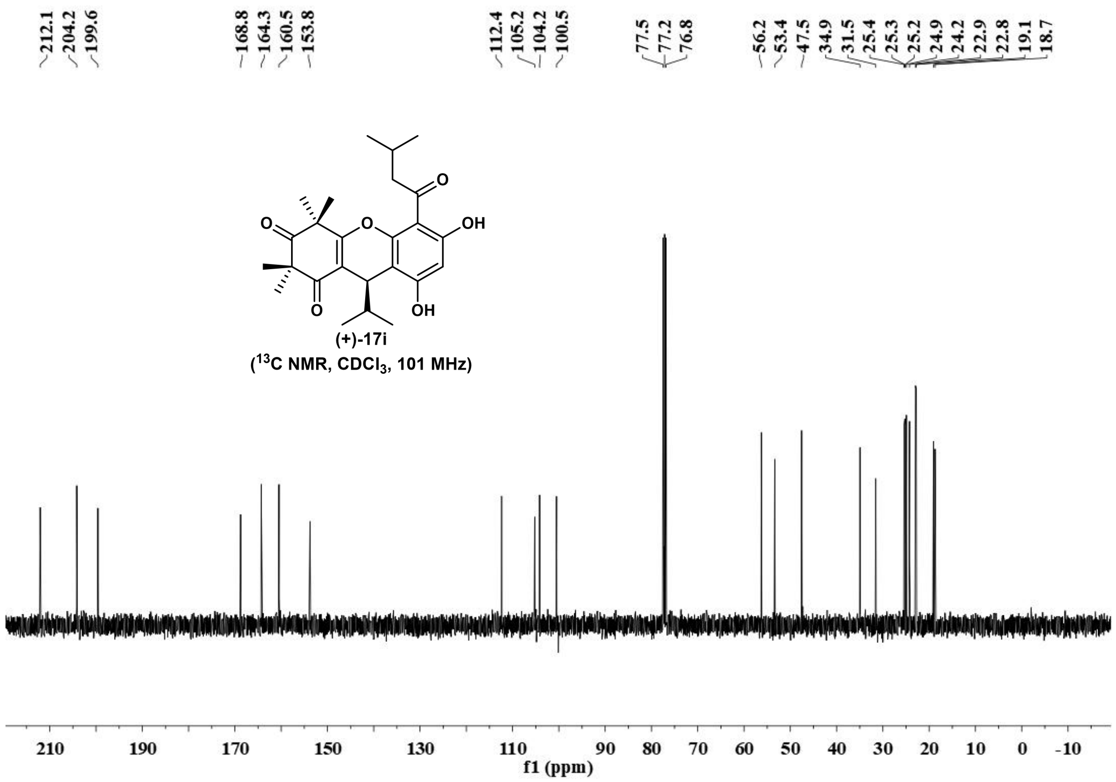

Supplementary Figure 172. ^{13}**C NMR spectra of (+)-17i.**

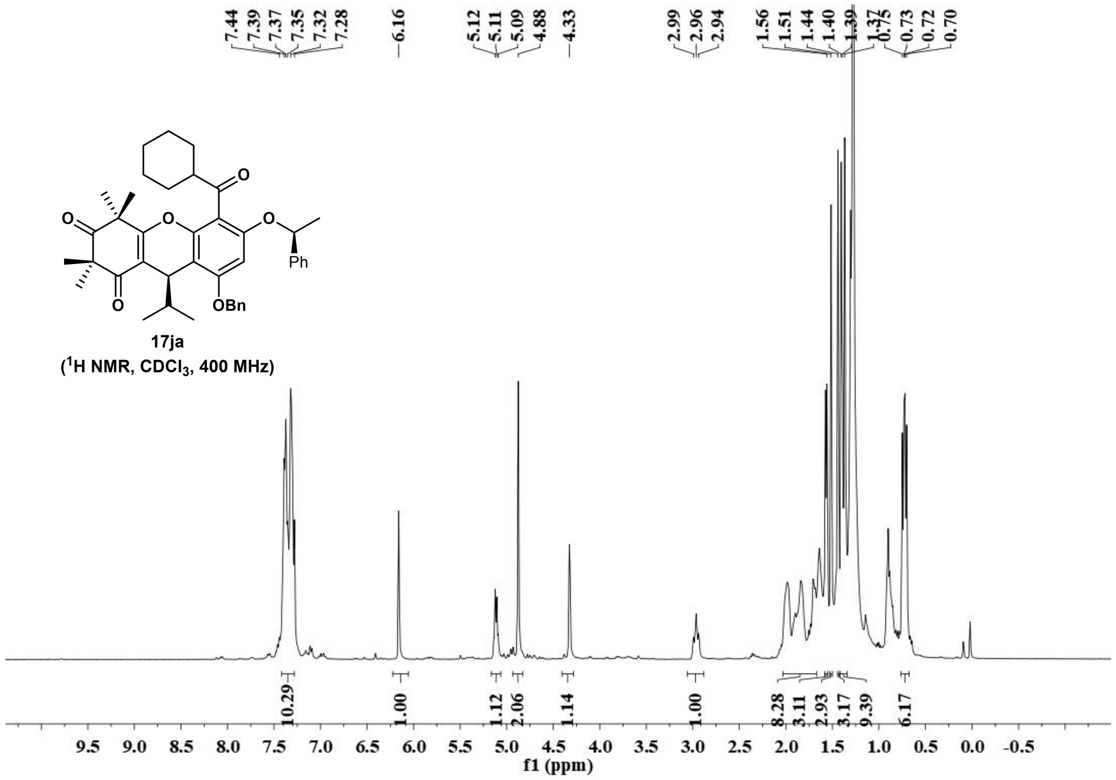

Supplementary Figure 173. ^{1}**H NMR spectra of 17ja.**

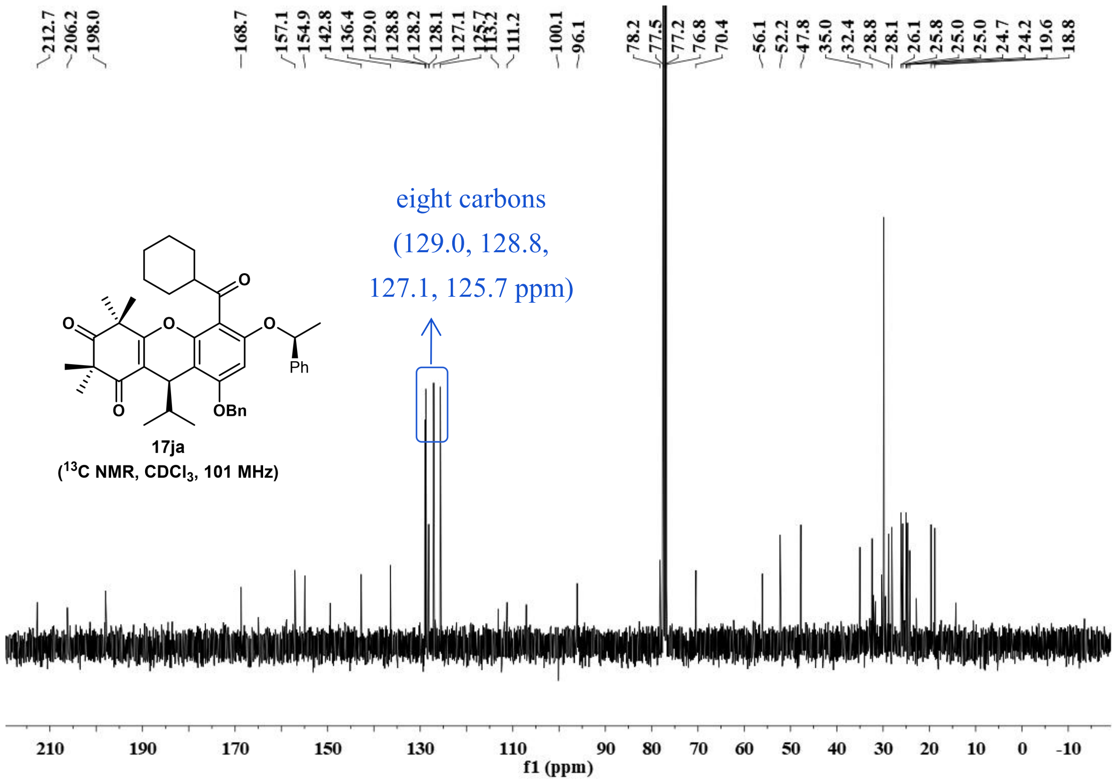

Supplementary Figure 174. ^{13}C NMR spectra of 17ja.

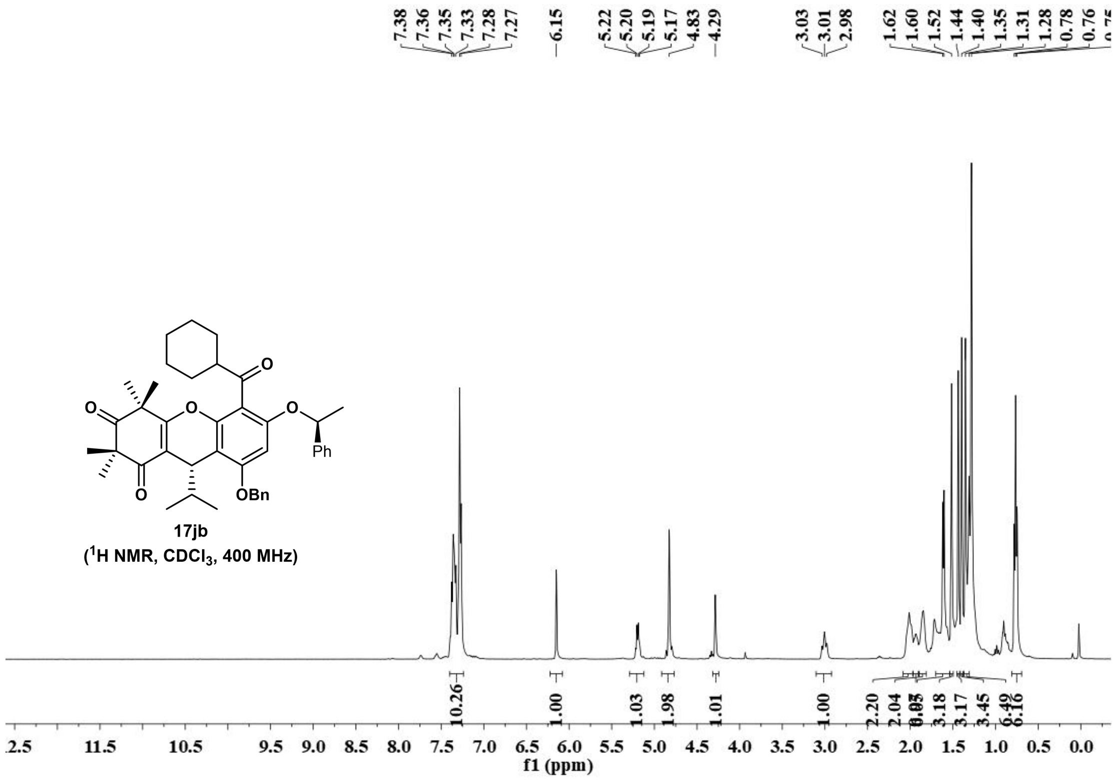

Supplementary Figure 175. ^{1}H NMR spectra of 17jb.

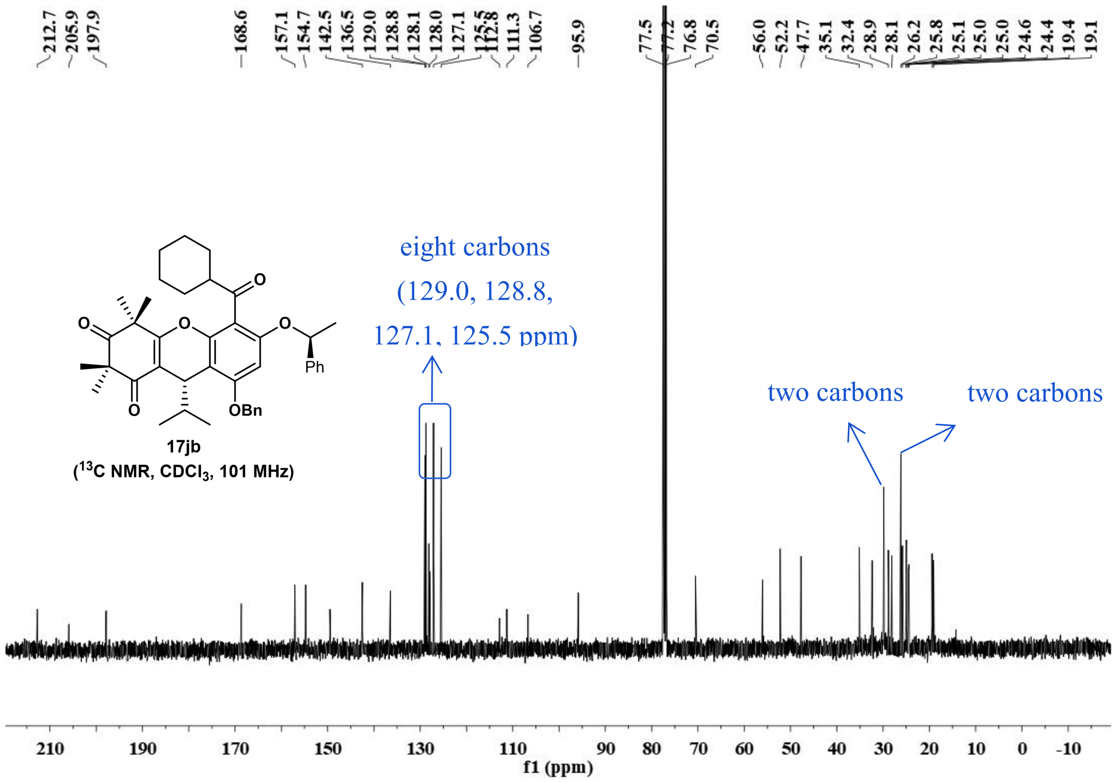

Supplementary Figure 176. ^{13}C NMR spectra of 17jb.

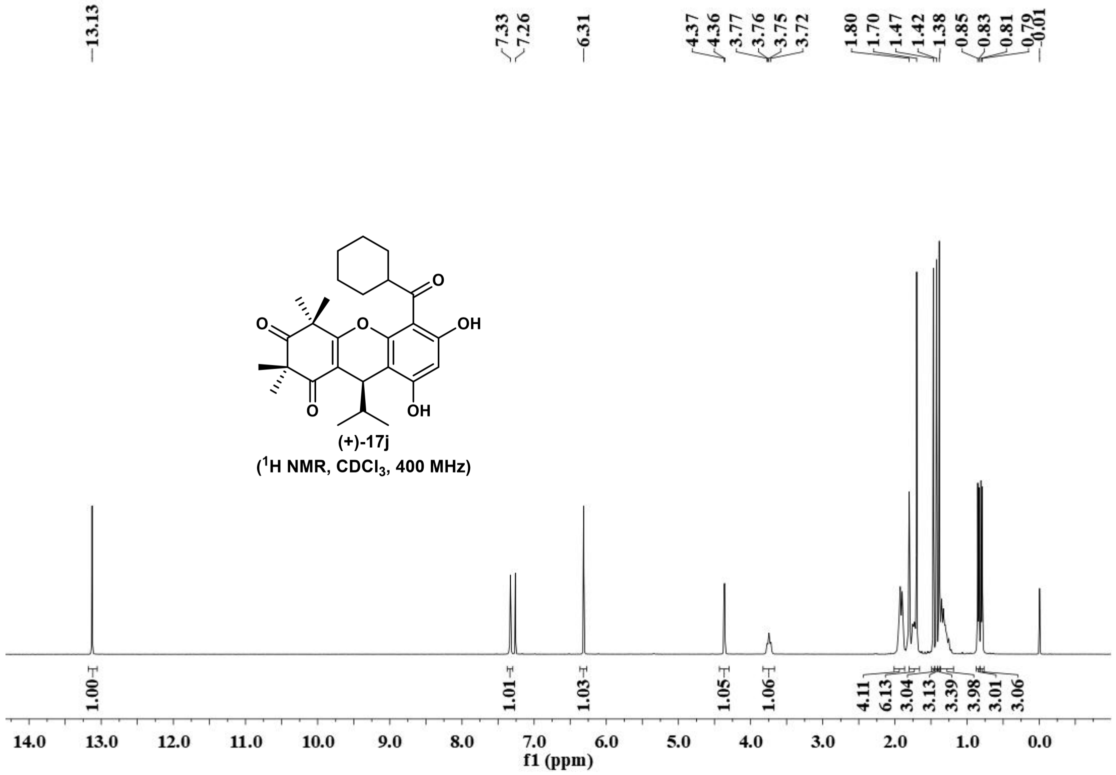

Supplementary Figure 177. ^{1}H NMR spectra of (+)-17j.

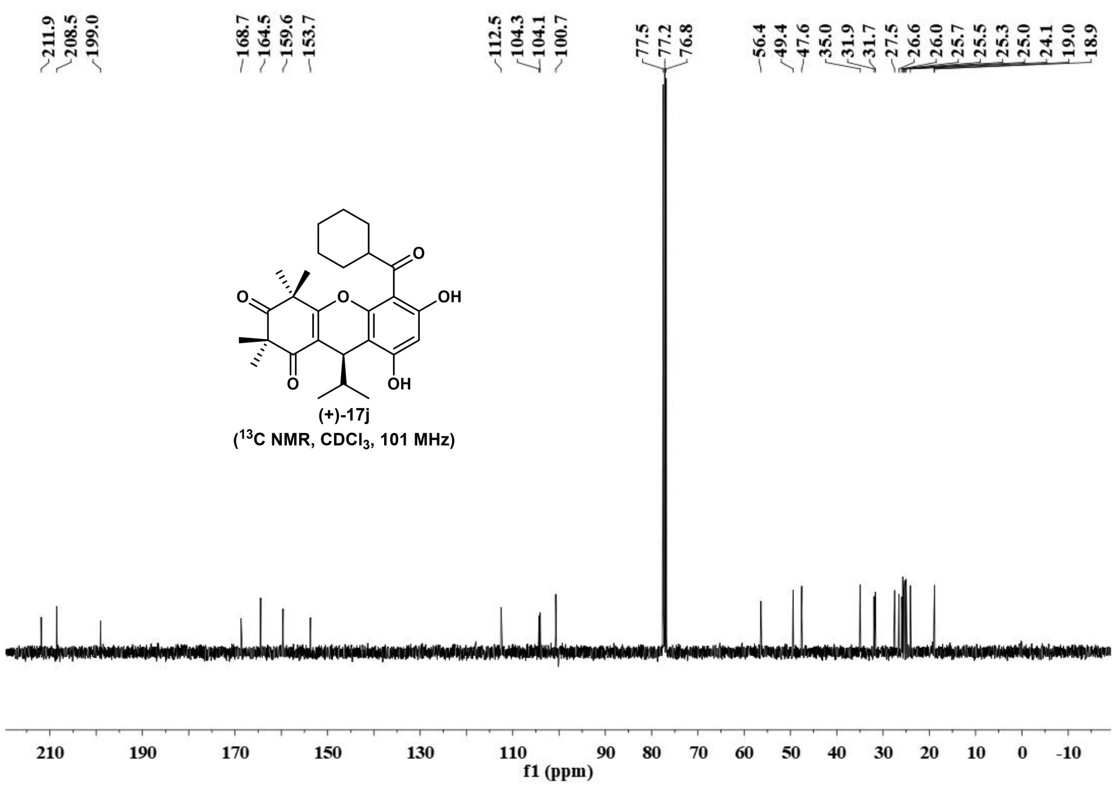

Supplementary Figure 178. ^{13}C NMR spectra of (+)-17j.

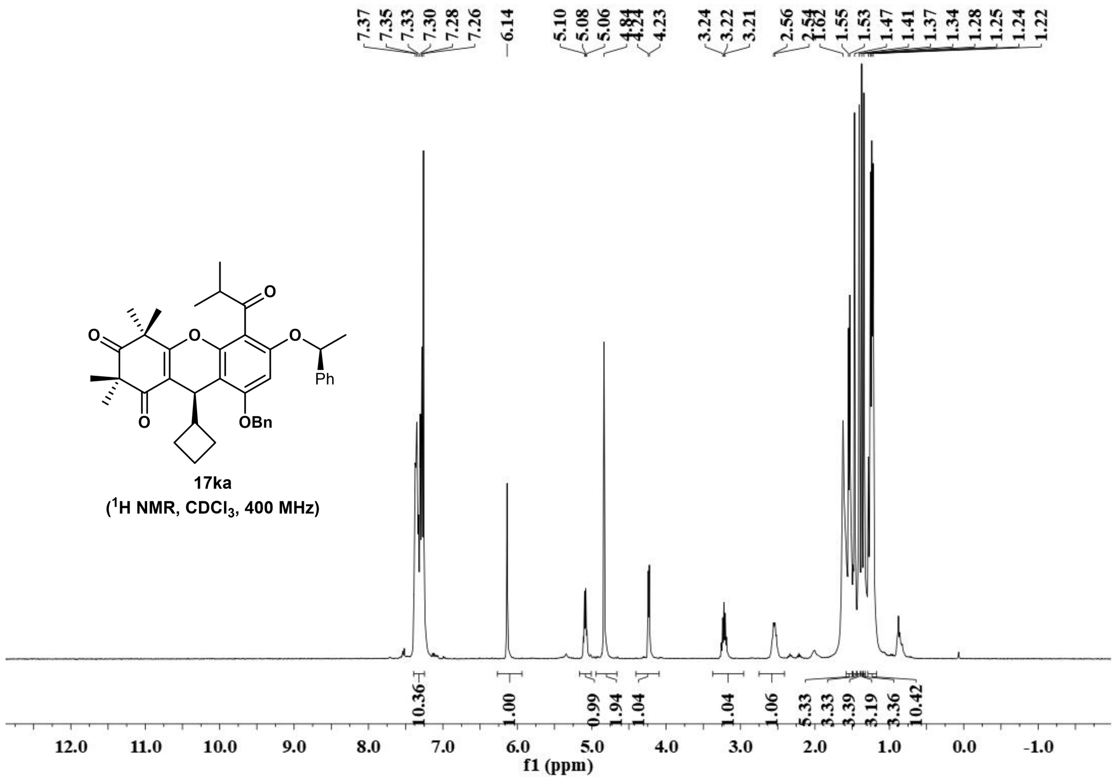

Supplementary Figure 179. ^{1}H NMR spectra of 17ka.

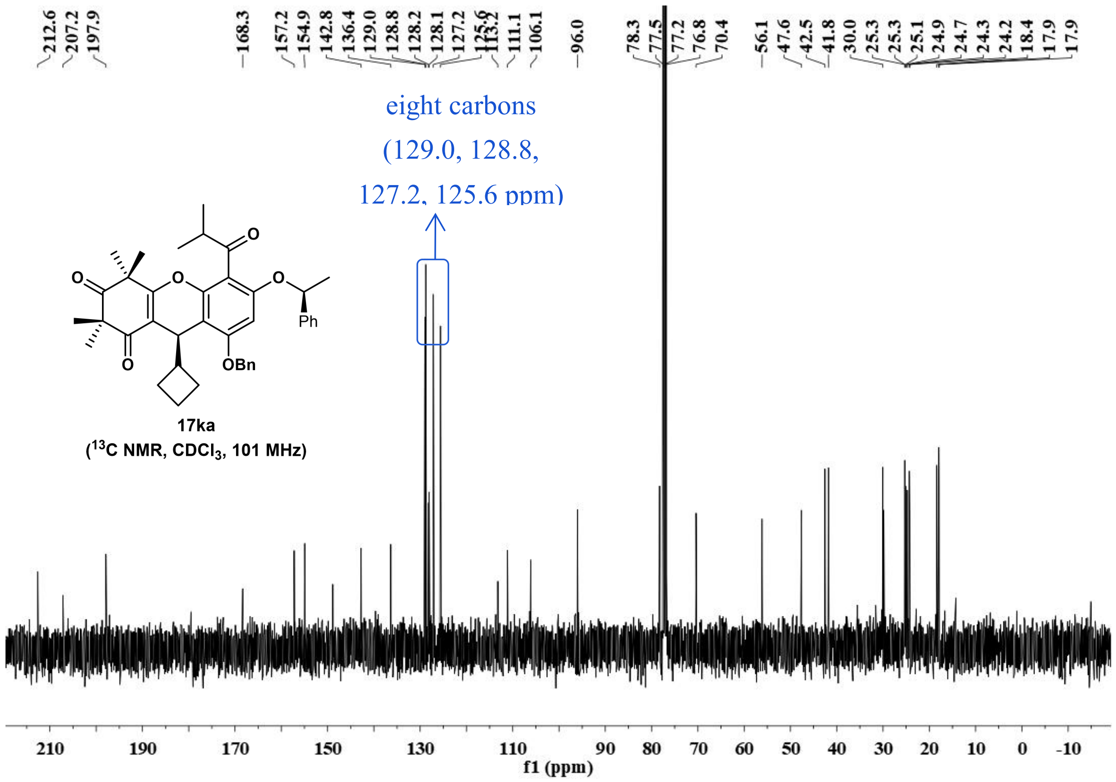

Supplementary Figure 180. ^{13}C NMR spectra of 17ka.

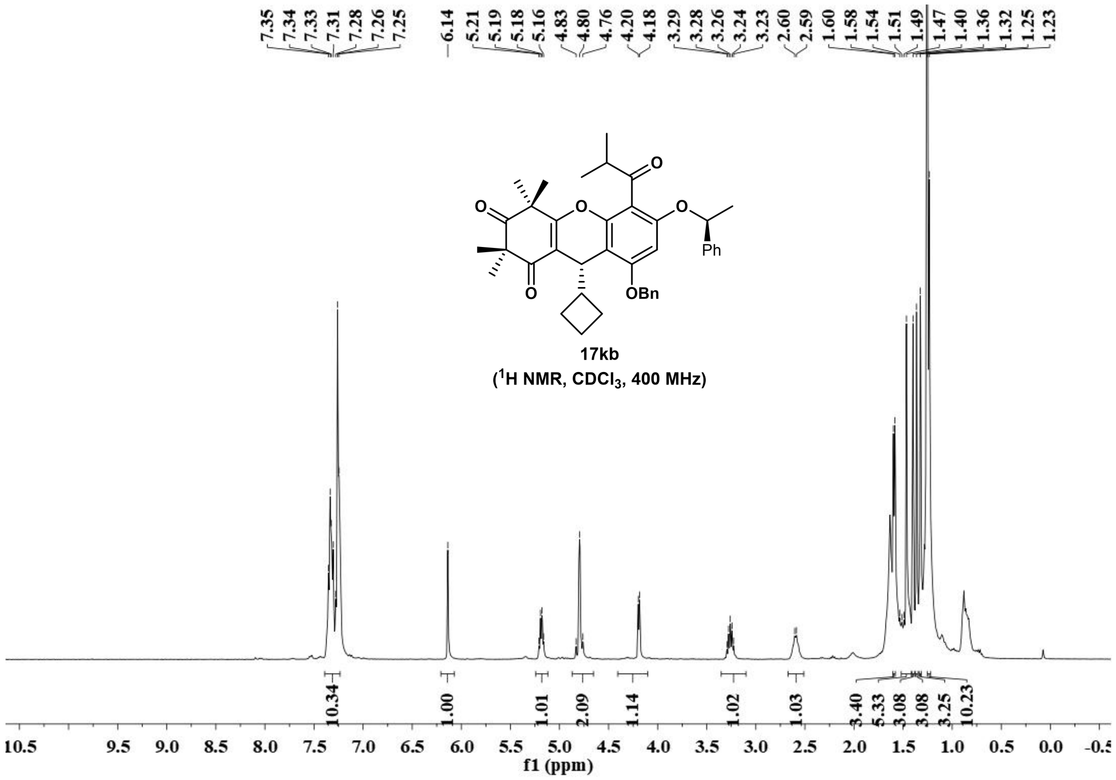

Supplementary Figure 181. ^{1}H NMR spectra of 17kb.

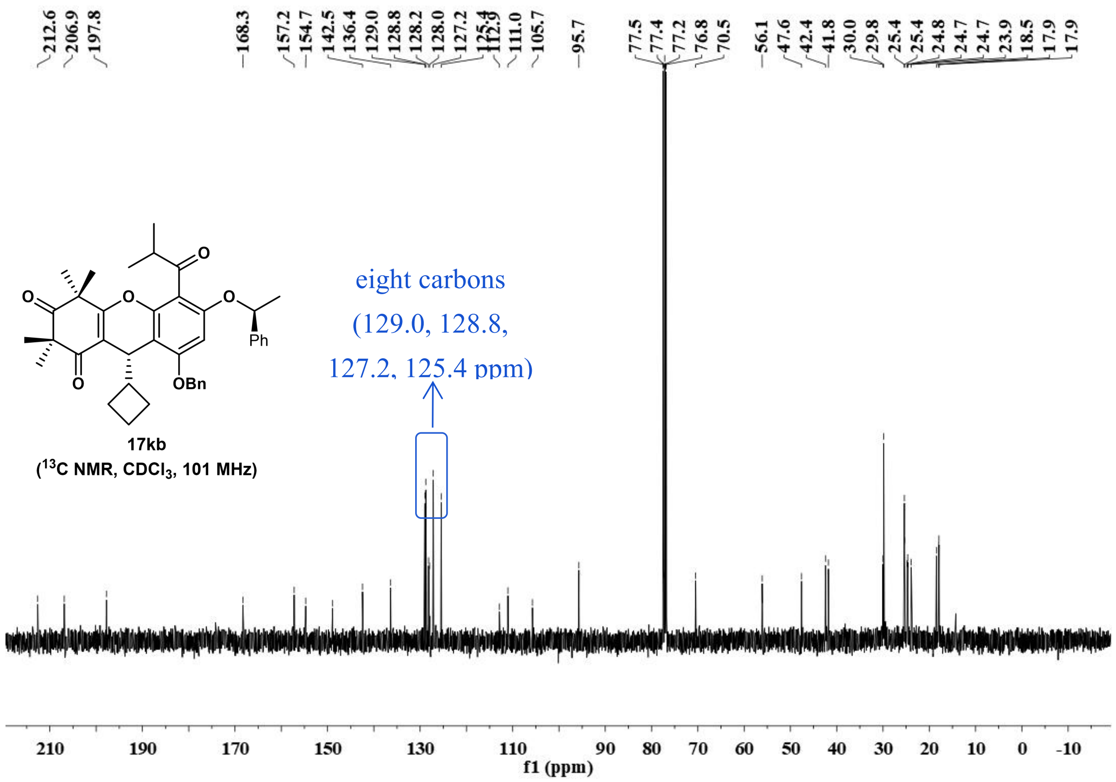

Supplementary Figure 182. ^{13}C **NMR spectra of 17kb.**

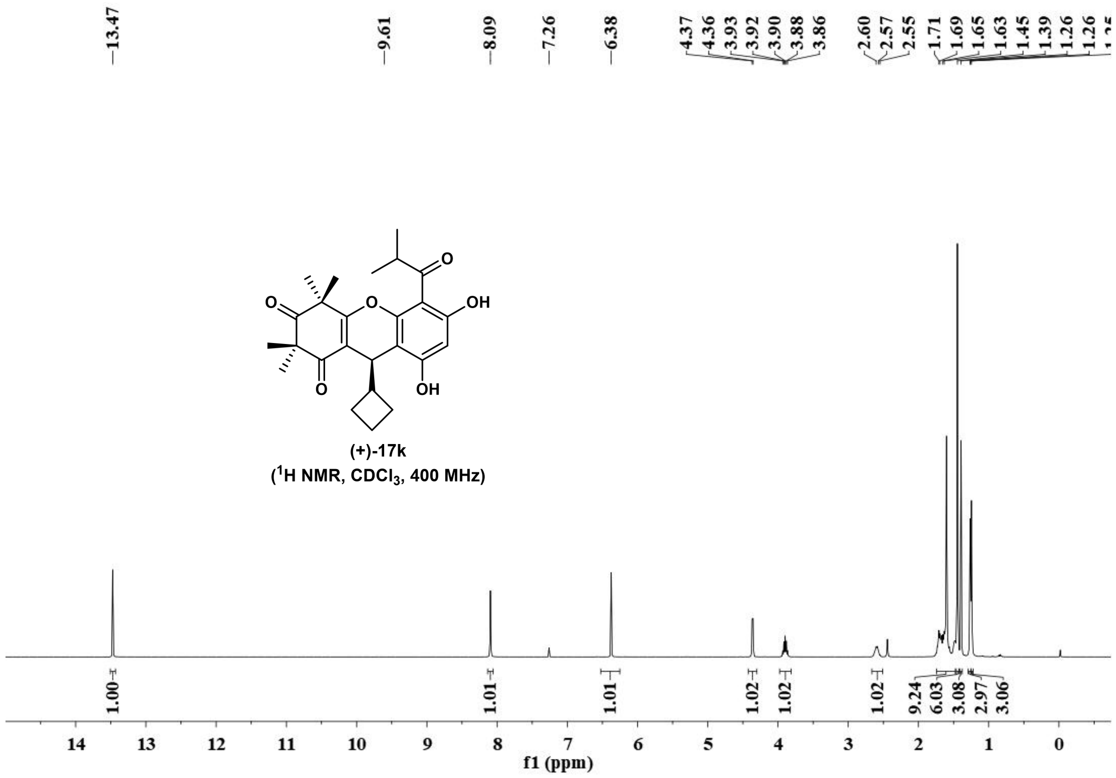

Supplementary Figure 183. ^{1}H **NMR spectra of (+)-17k.**

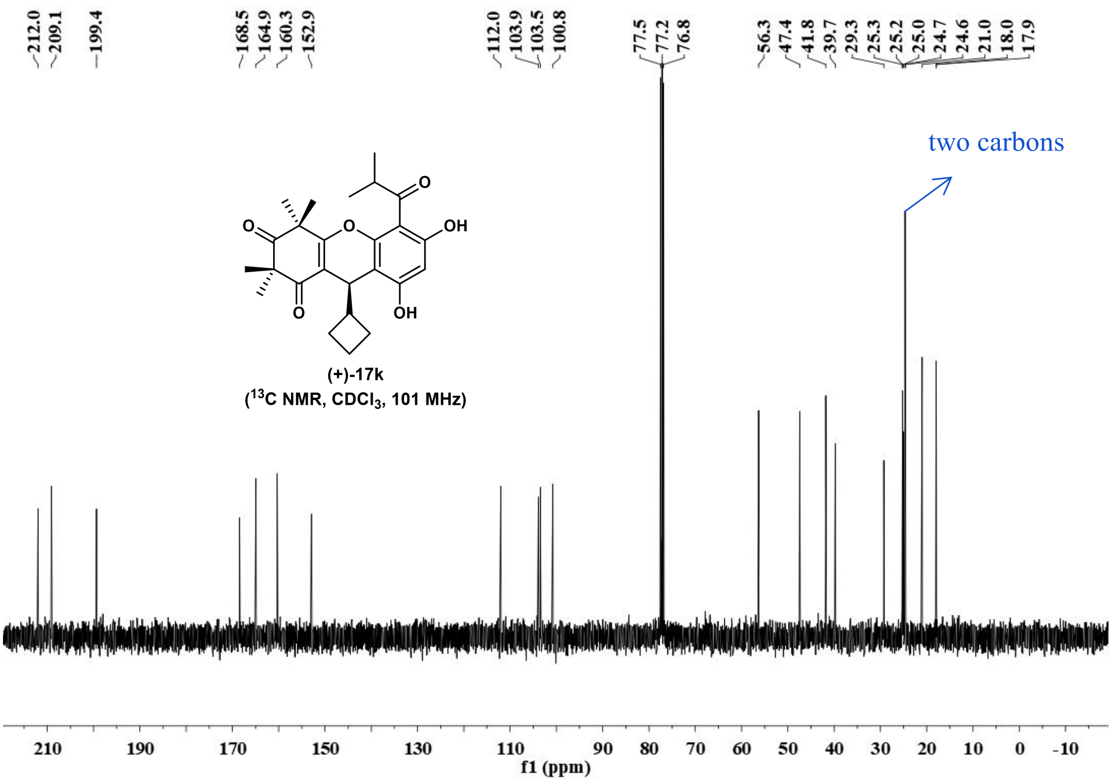

Supplementary Figure 184. ^{13}C NMR spectra of (+)-17k.

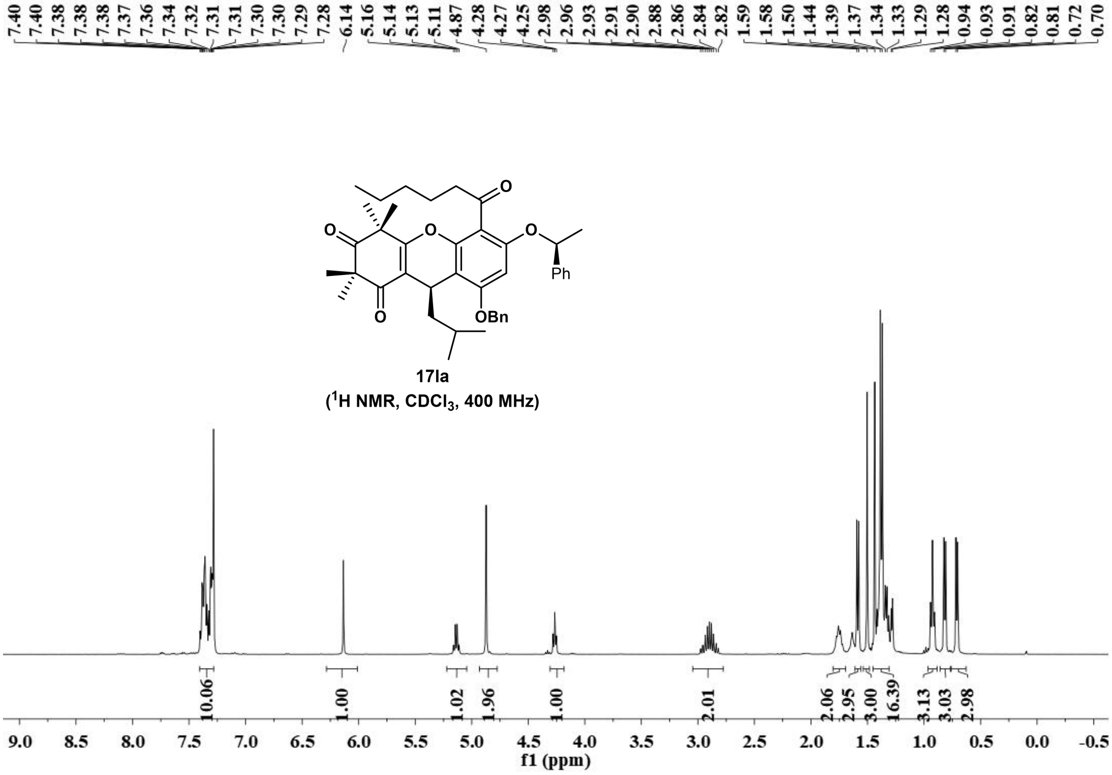

Supplementary Figure 185. ^{1}H NMR spectra of 17la.

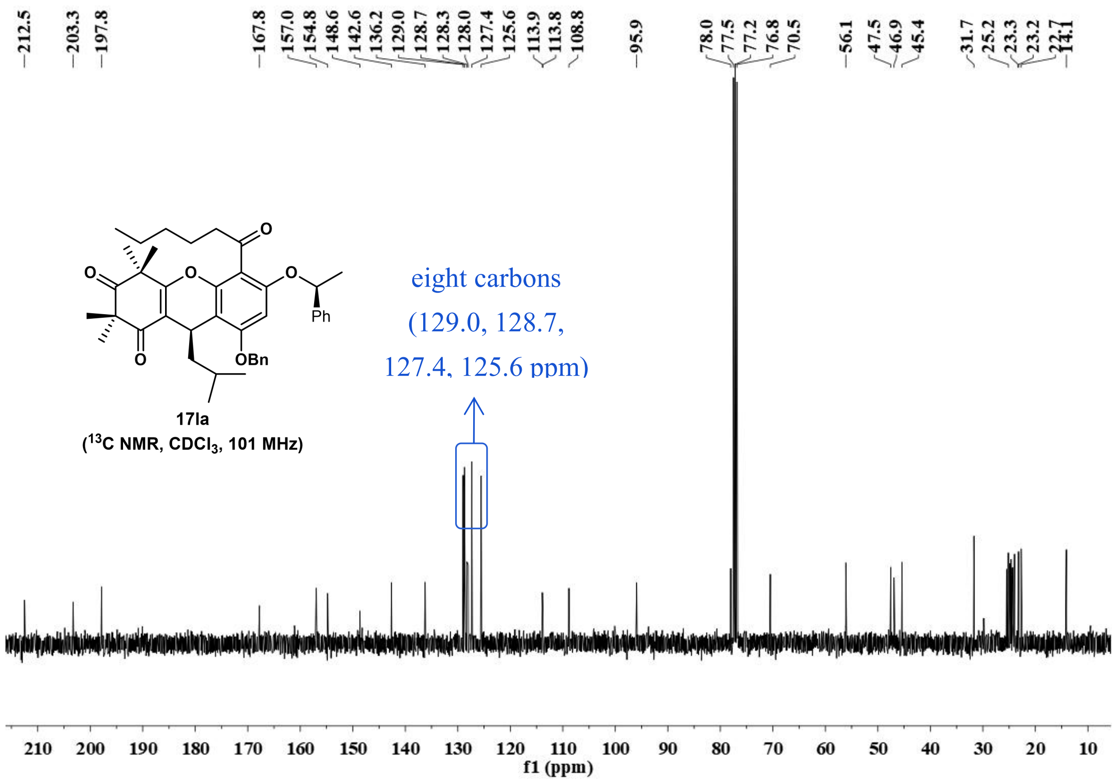

Supplementary Figure 186. ^{13}C NMR spectra of 17la.

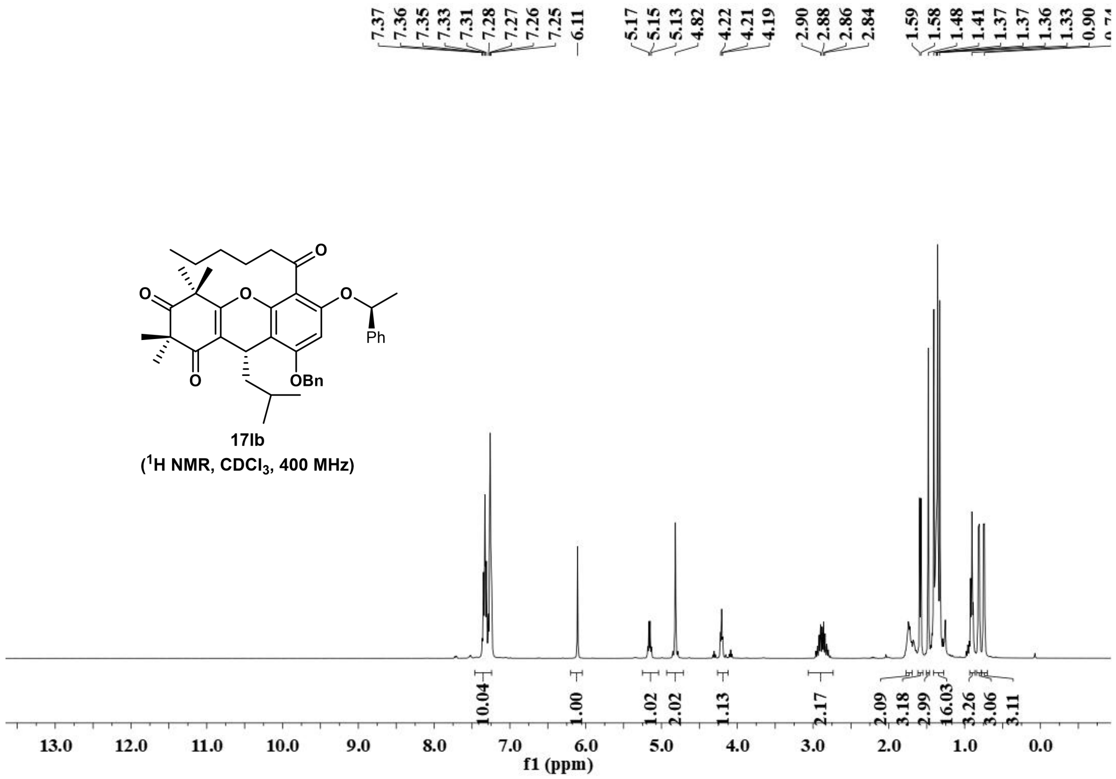

Supplementary Figure 187. ^{1}H NMR spectra of 17lb.

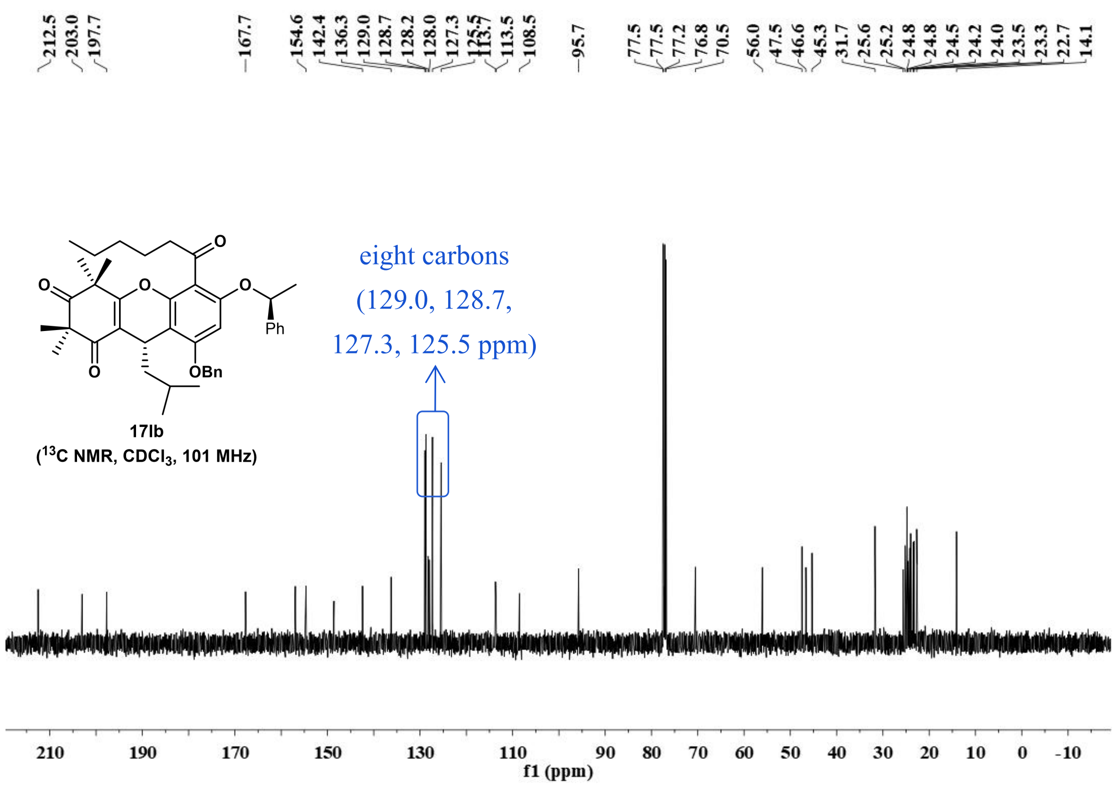

Supplementary Figure 188. ^{13}C NMR spectra of 17lb.

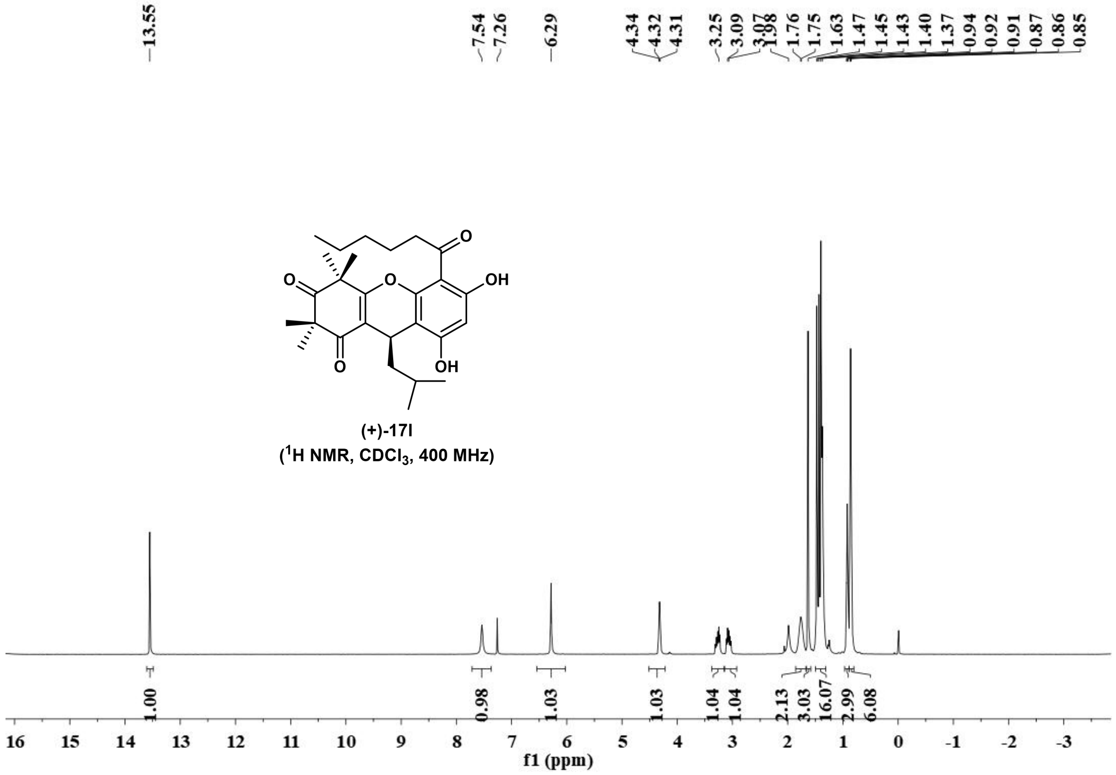

Supplementary Figure 189. ^{1}H NMR spectra of (+)-17l.

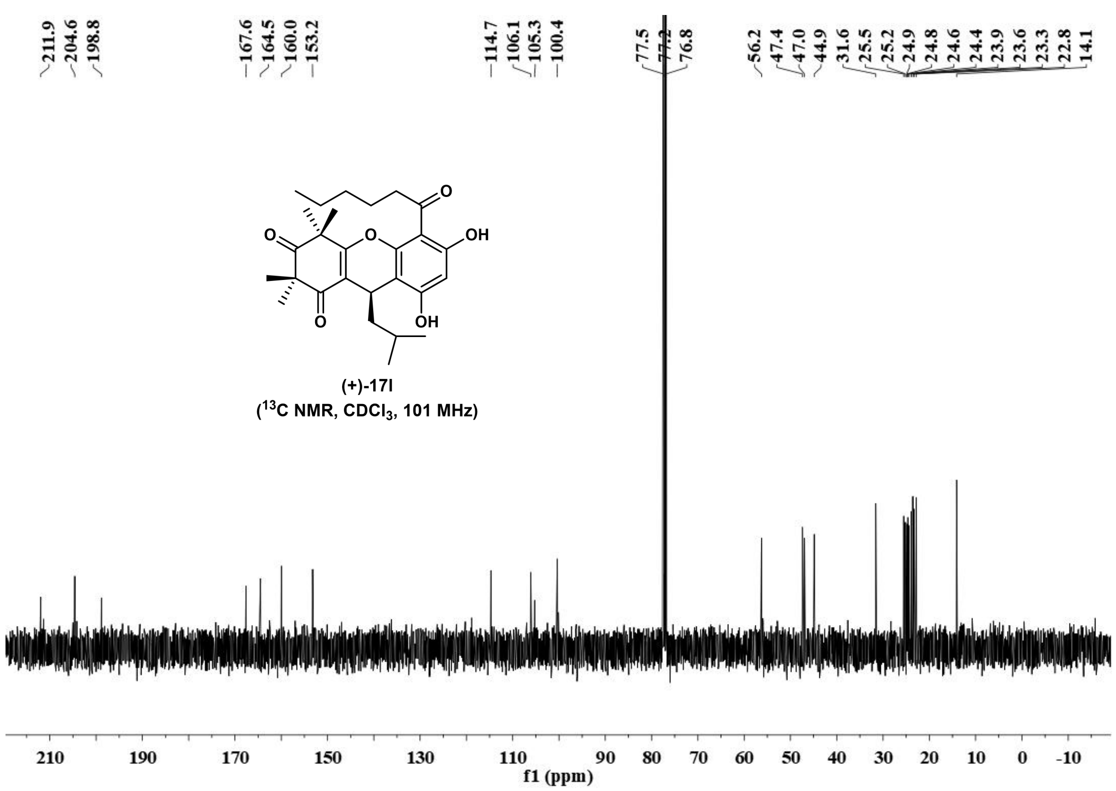

Supplementary Figure 190. ^{13}C NMR spectra of (+)-17l.

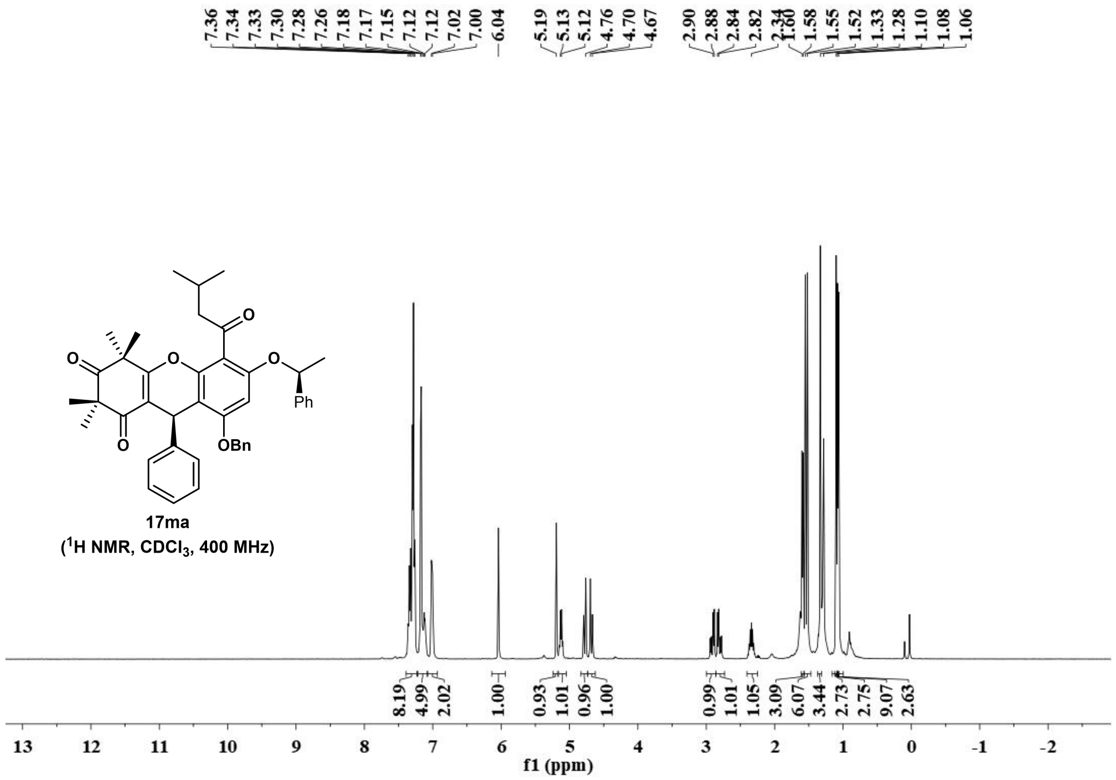

Supplementary Figure 191. ^{1}H NMR spectra of 17ma.

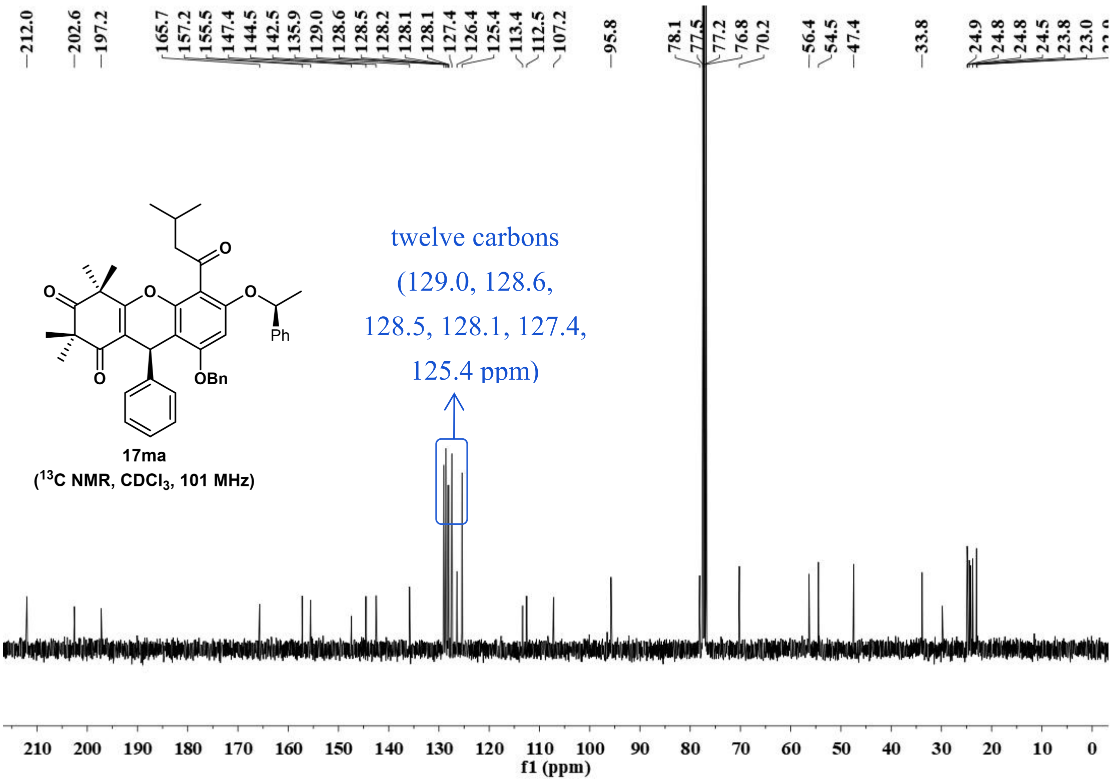

Supplementary Figure 192. ^{13}C NMR spectra of 17ma.

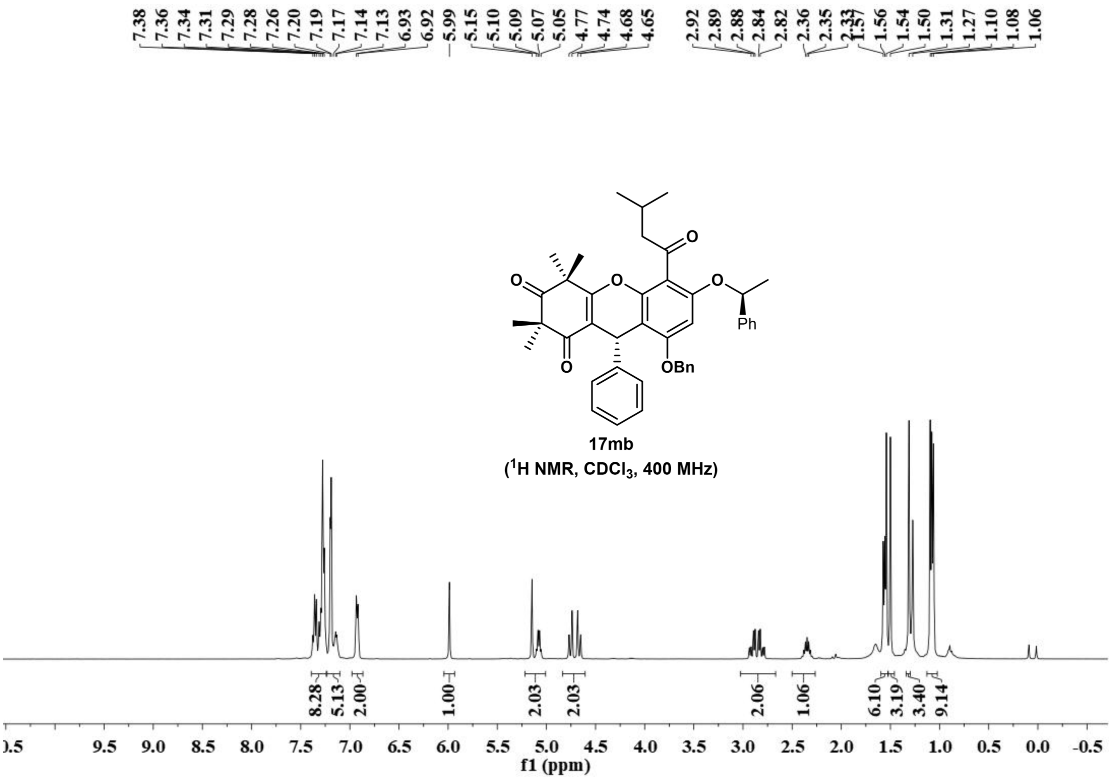

Supplementary Figure 193. ^{1}H NMR spectra of 17mb.

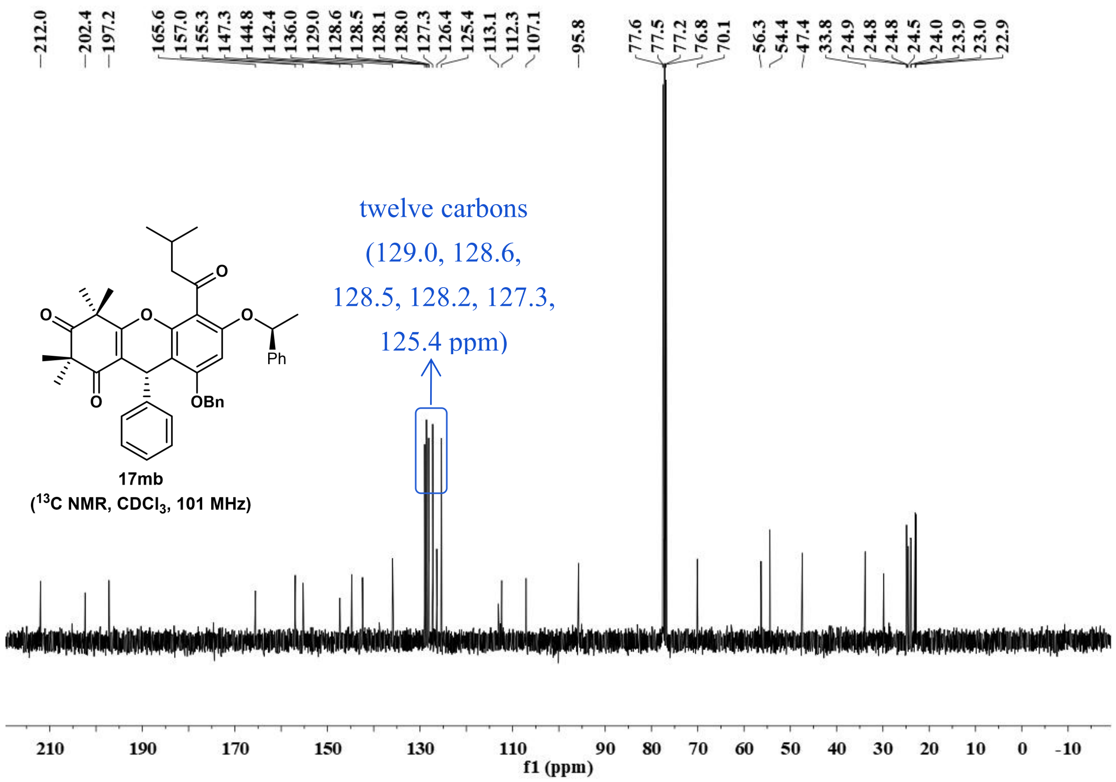

Supplementary Figure 194. ^{13}C NMR spectra of 17mb.

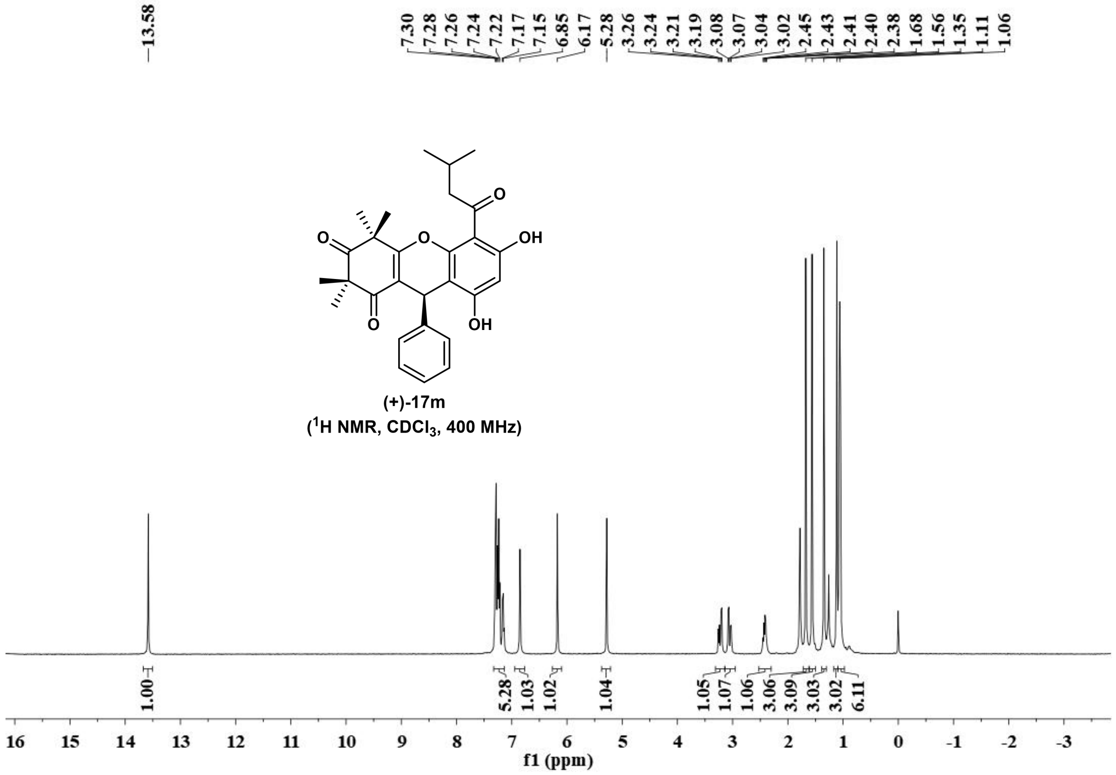

Supplementary Figure 195. ^{1}H NMR spectra of (+)-17m.

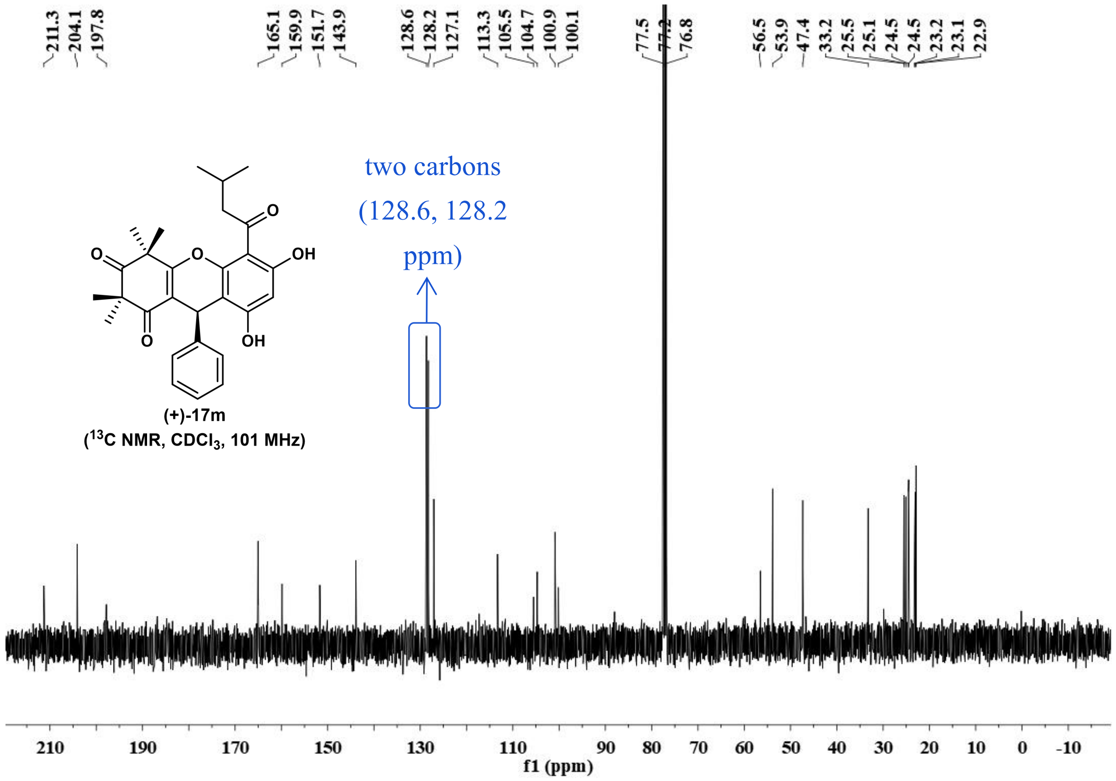

Supplementary Figure 196. ^{13}C NMR spectra of (+)-17m.

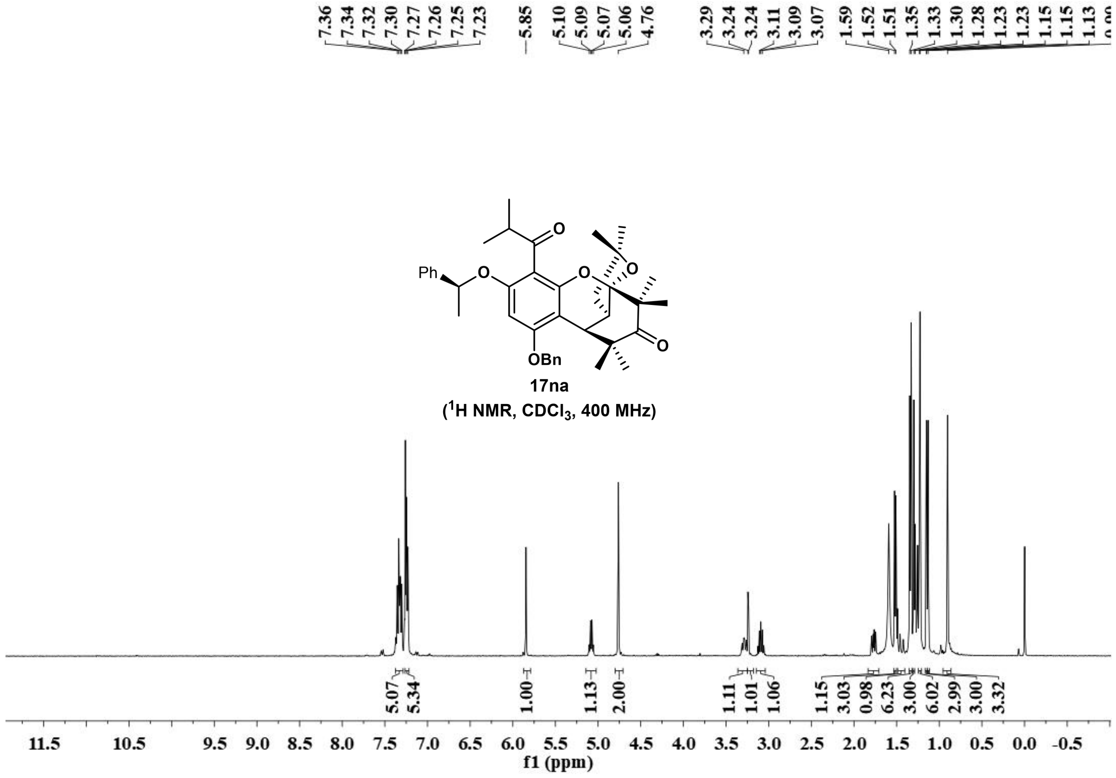

Supplementary Figure 197. ^{1}H NMR spectra of 17na.

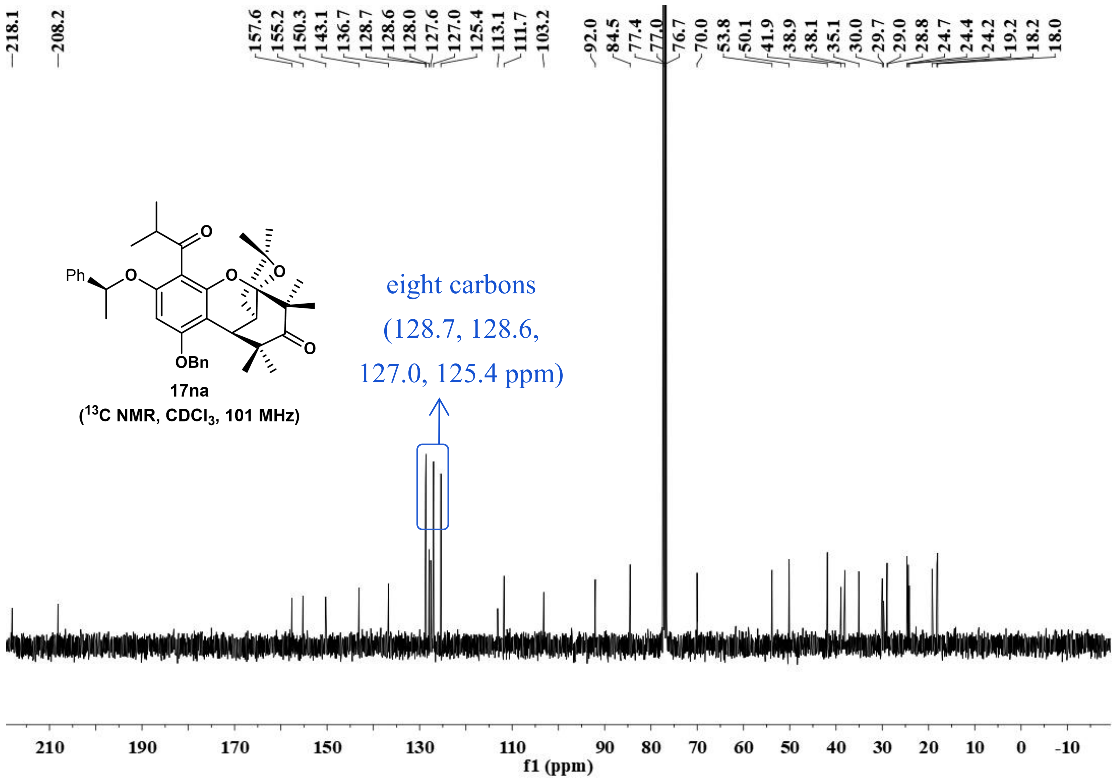

Supplementary Figure 198. ^{13}**C NMR spectra of 17na.**

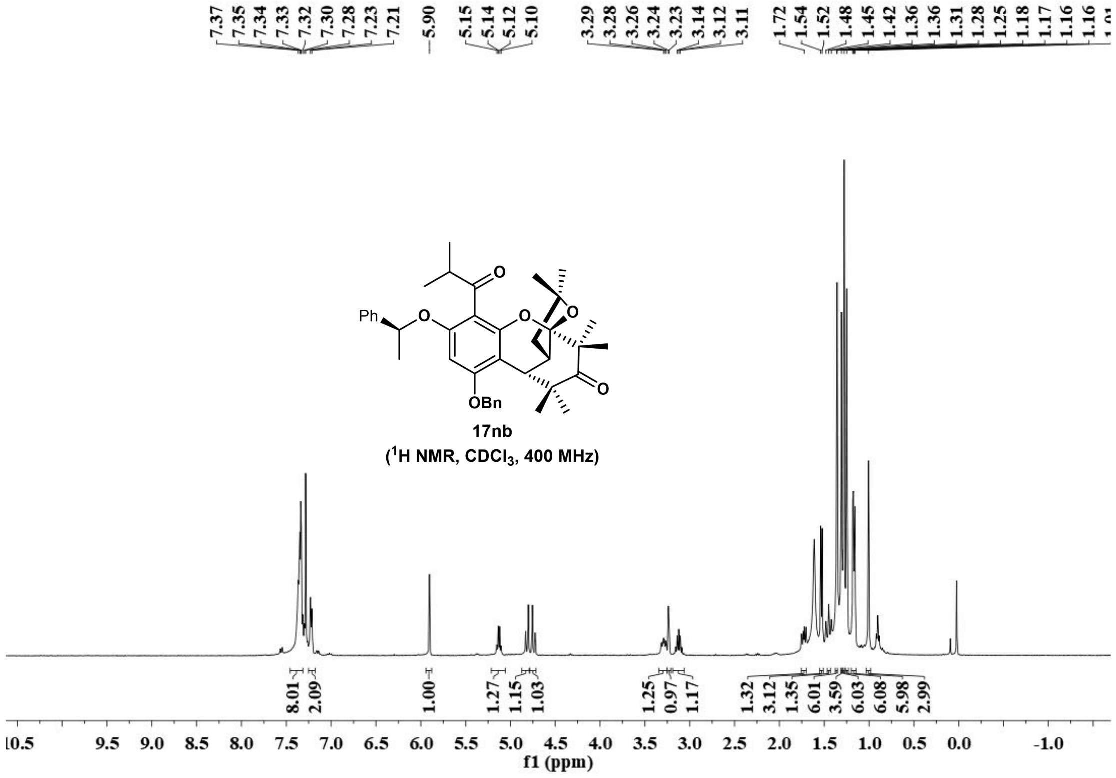

Supplementary Figure 199. ^{1}**H NMR spectra of 17nb.**

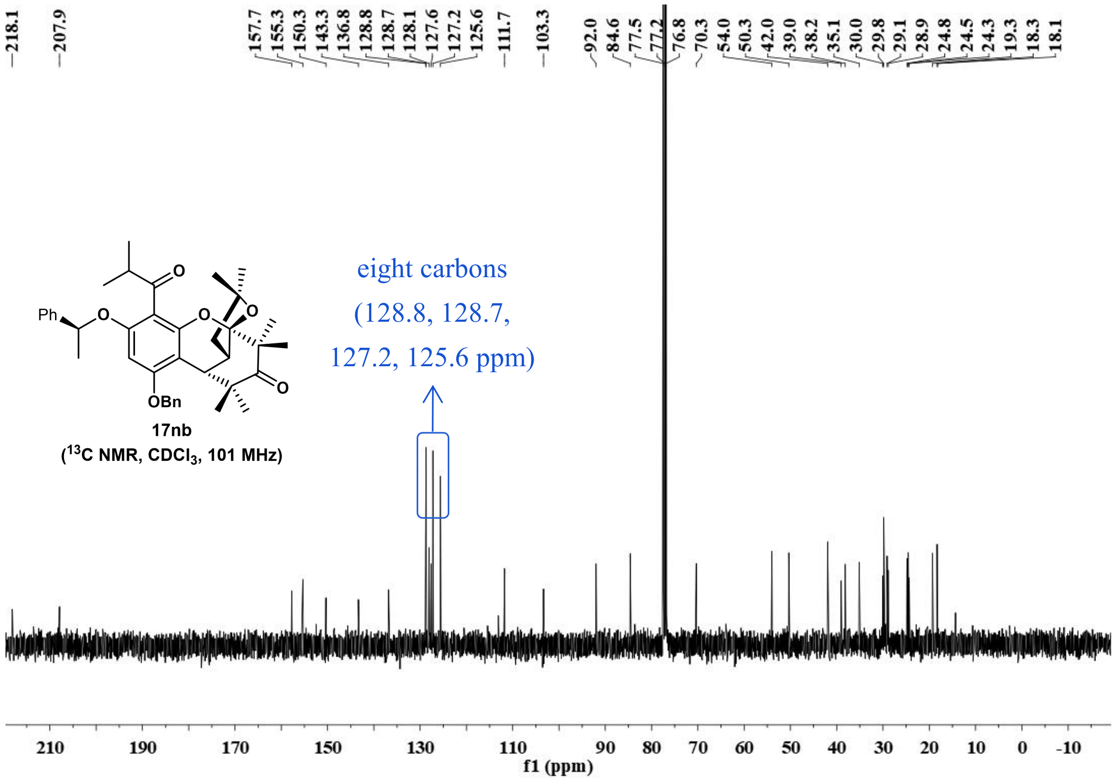

Supplementary Figure 200. ^{13}**C NMR spectra of 17nb.**

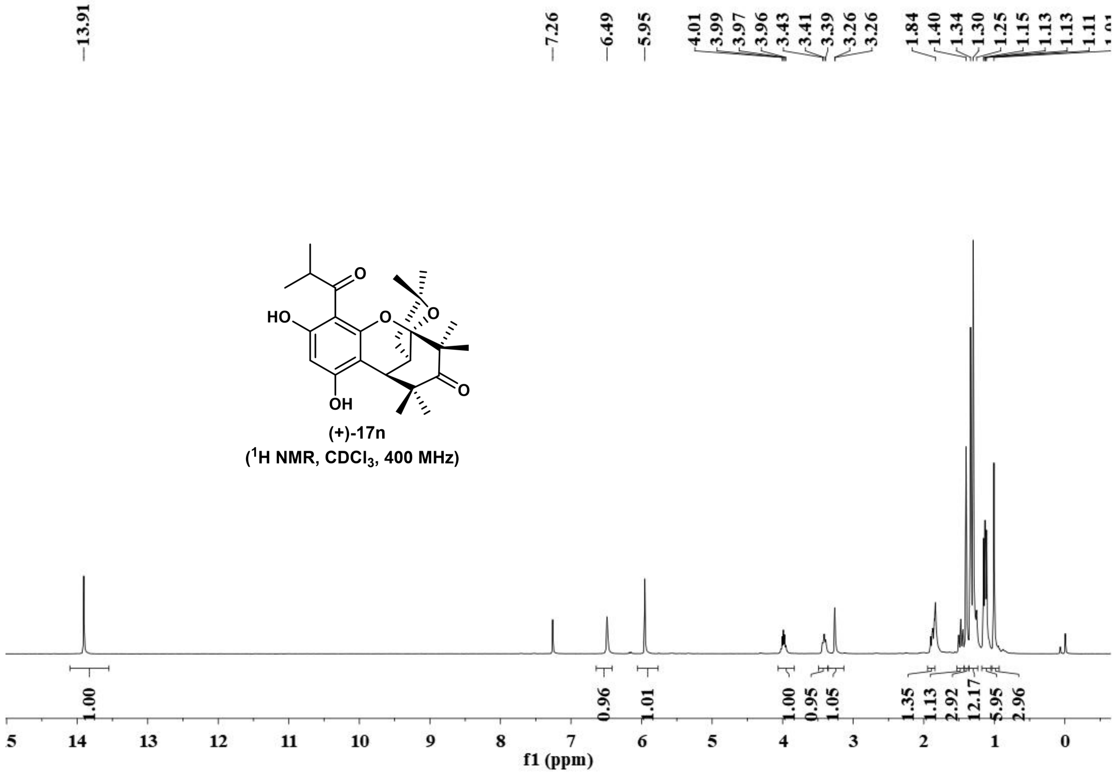

Supplementary Figure 201. ^{1}**H NMR spectra of (+)-17n.**

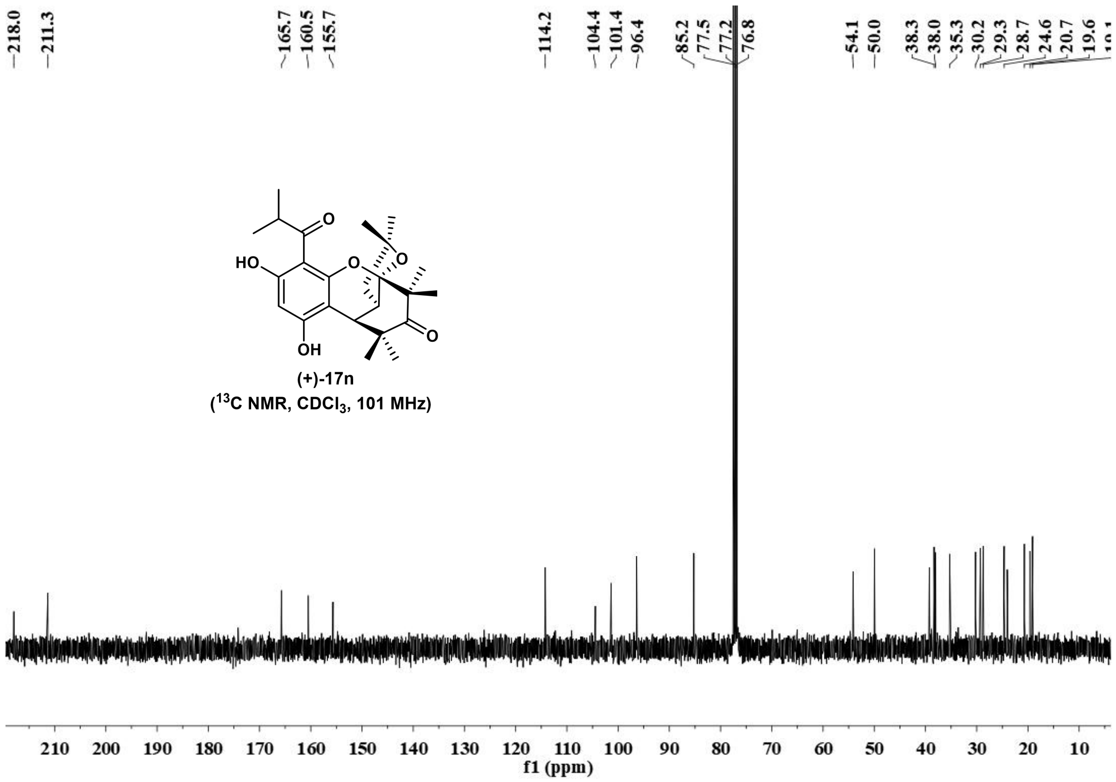

Supplementary Figure 202. ^{13}C NMR spectra of (+)-17n.

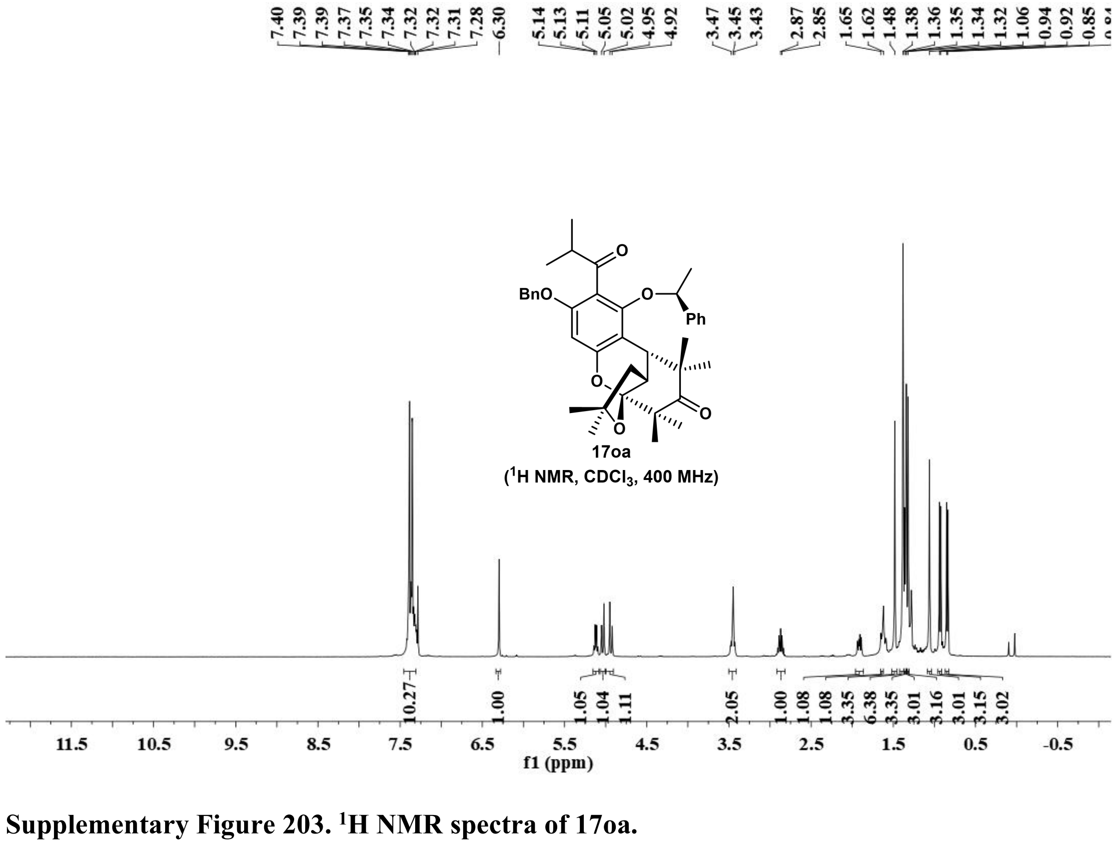

Supplementary Figure 203. ^{1}H NMR spectra of 17oa.

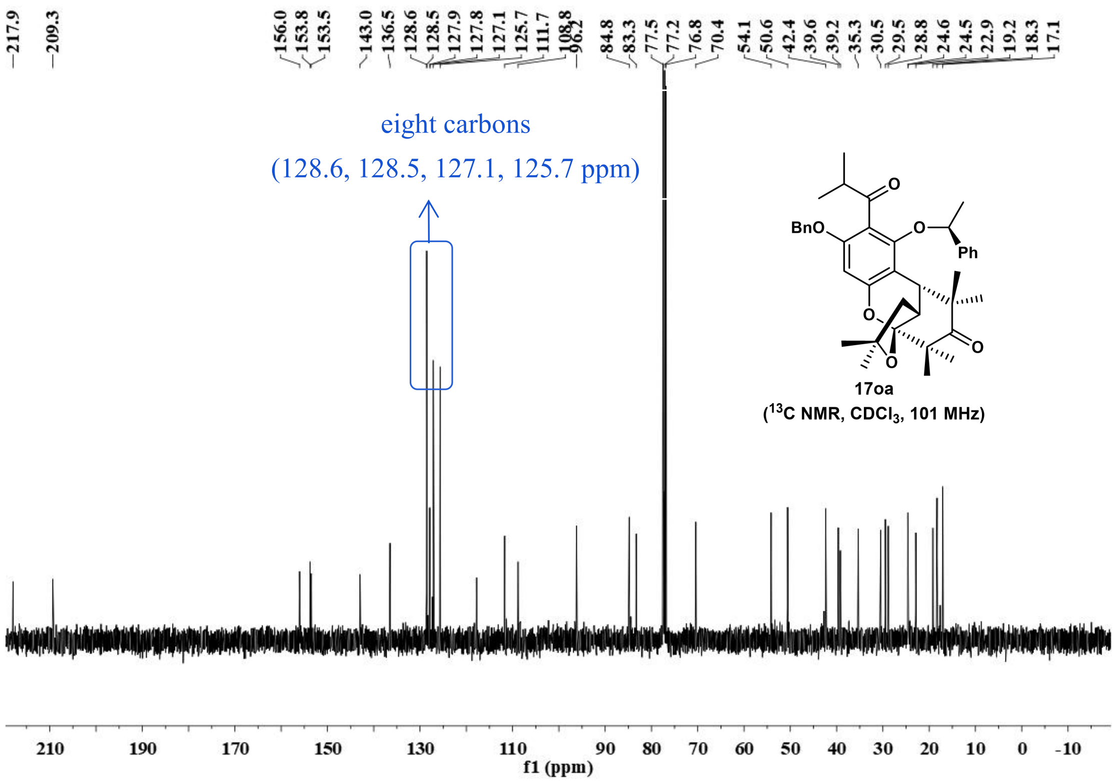

Supplementary Figure 204. ^{13}C NMR spectra of 17oa.

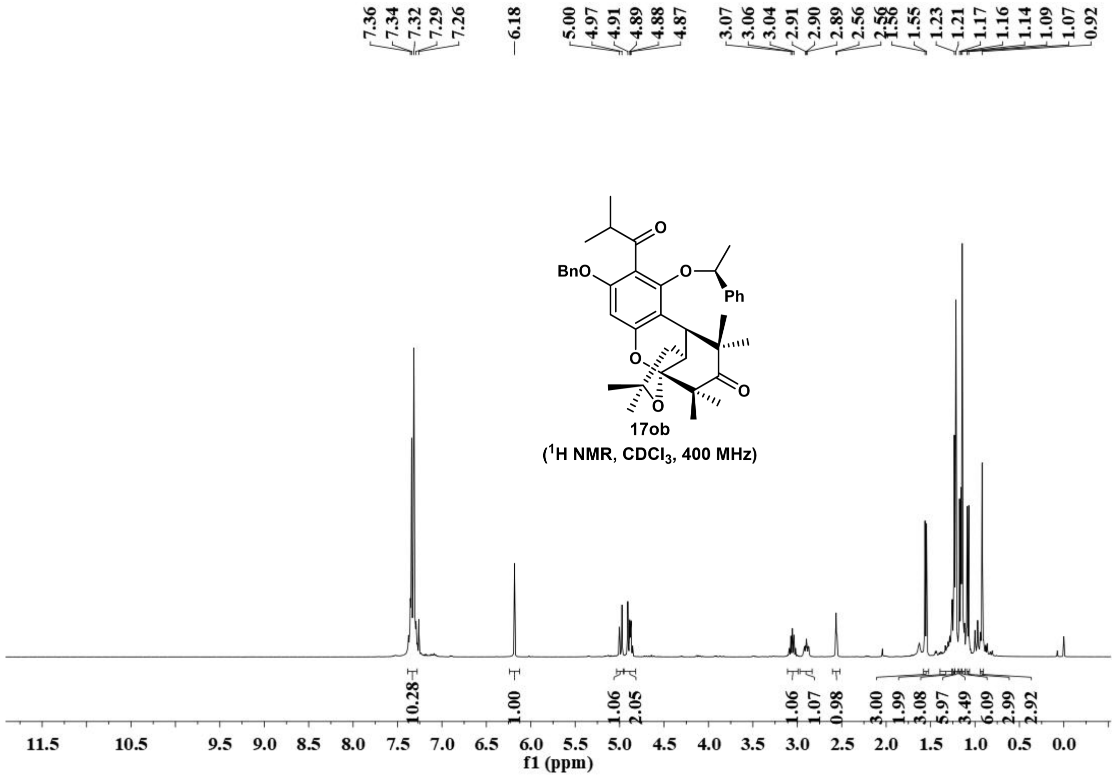

Supplementary Figure 205. ^{1}H NMR spectra of 17ob.

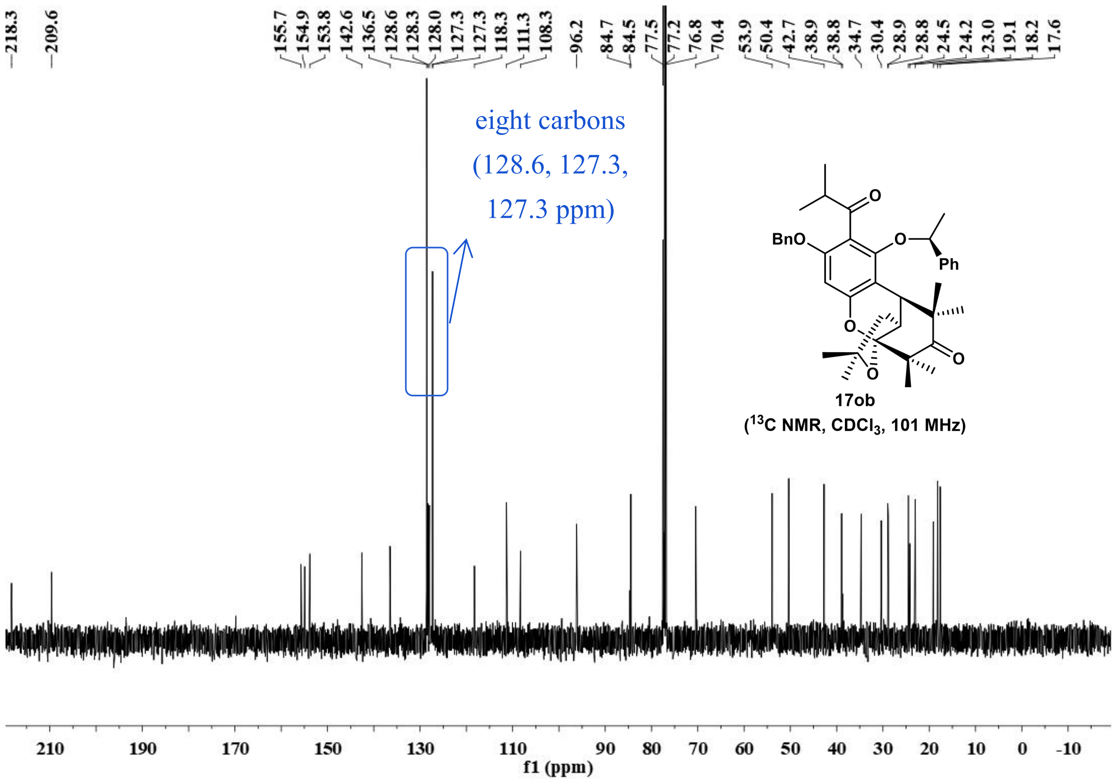

Supplementary Figure 206. ^{13}**C NMR spectra of 17ob.**

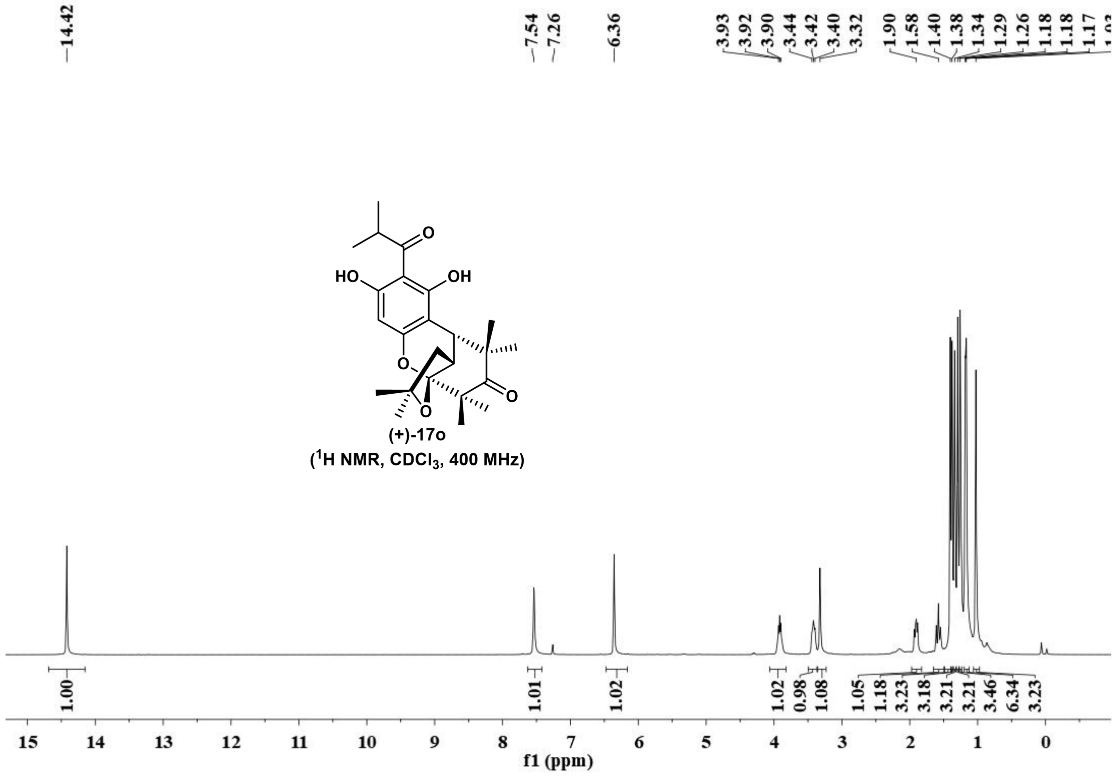

Supplementary Figure 207. ^{1}**H NMR spectra of (+)-17o.**

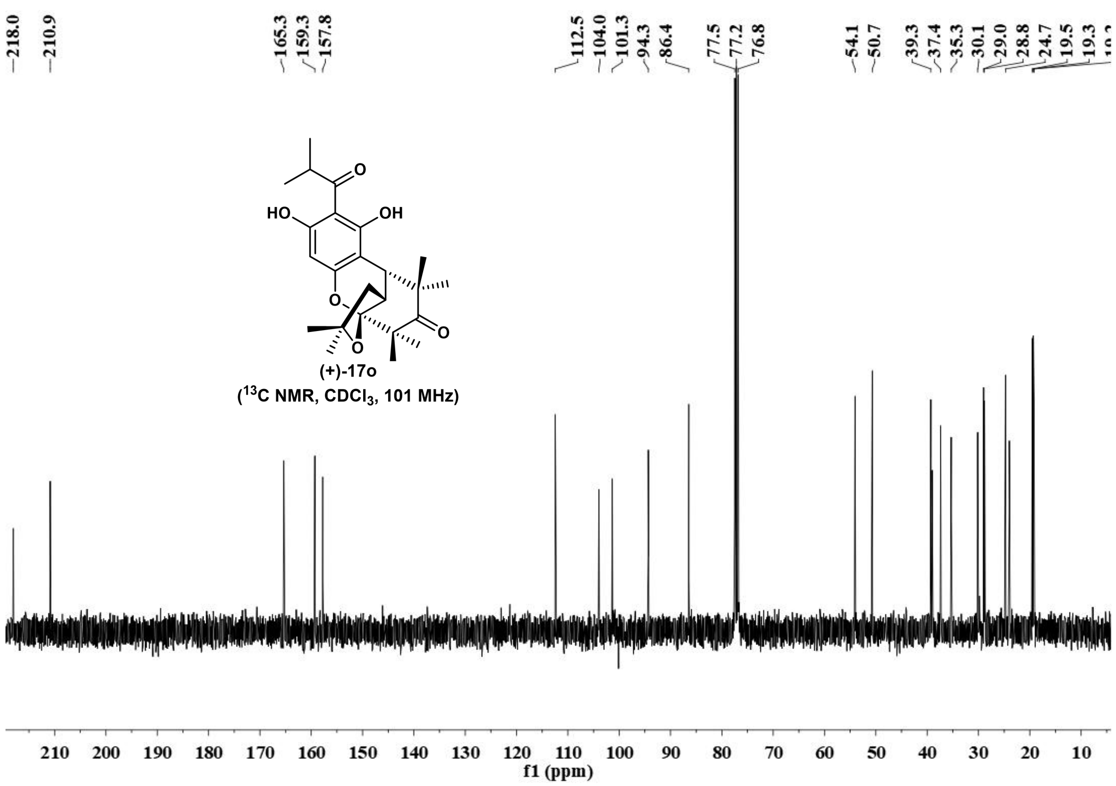

Supplementary Figure 208. ^{13}**C NMR spectra of (+)-17o.**

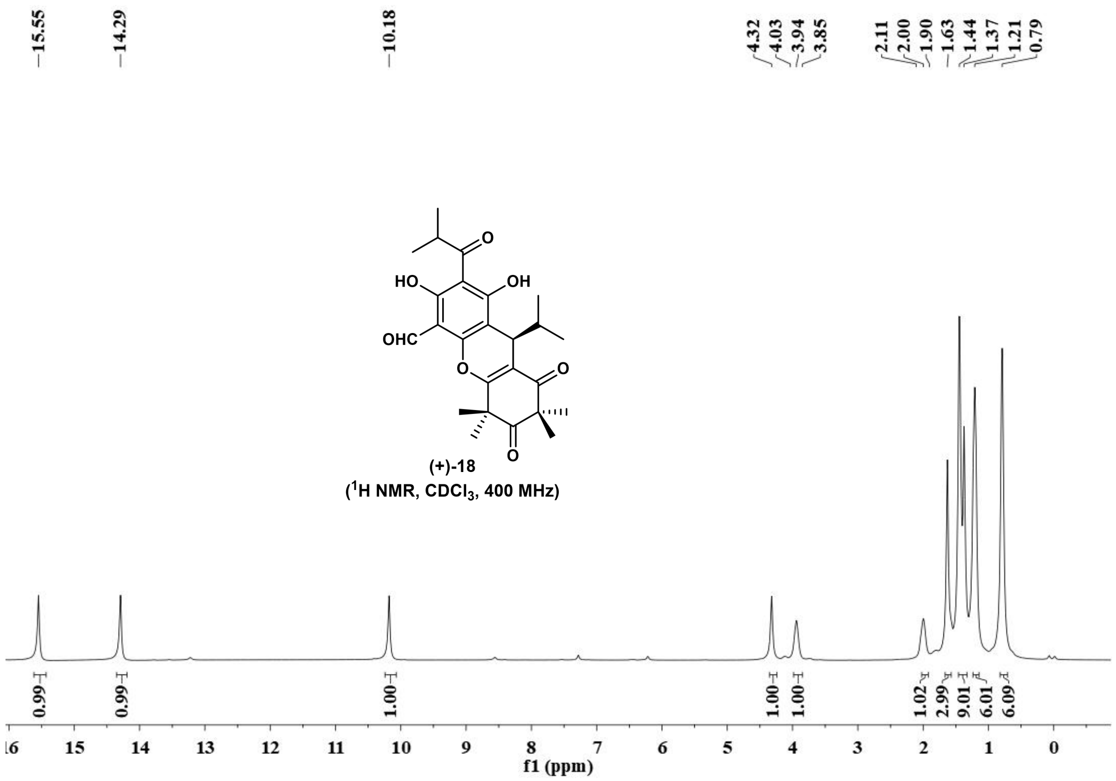

Supplementary Figure 209. ^{1}**H NMR spectra of (+)-18.**

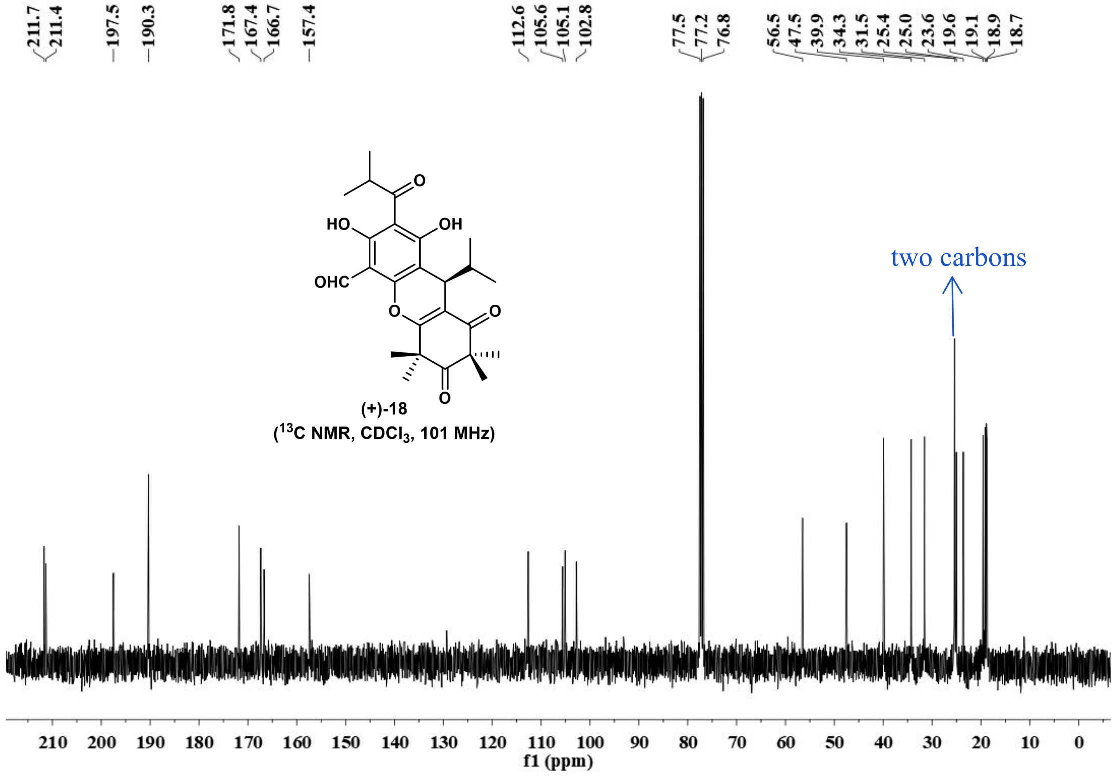

Supplementary Figure 210. ^{13}C NMR spectra of (+)-18.

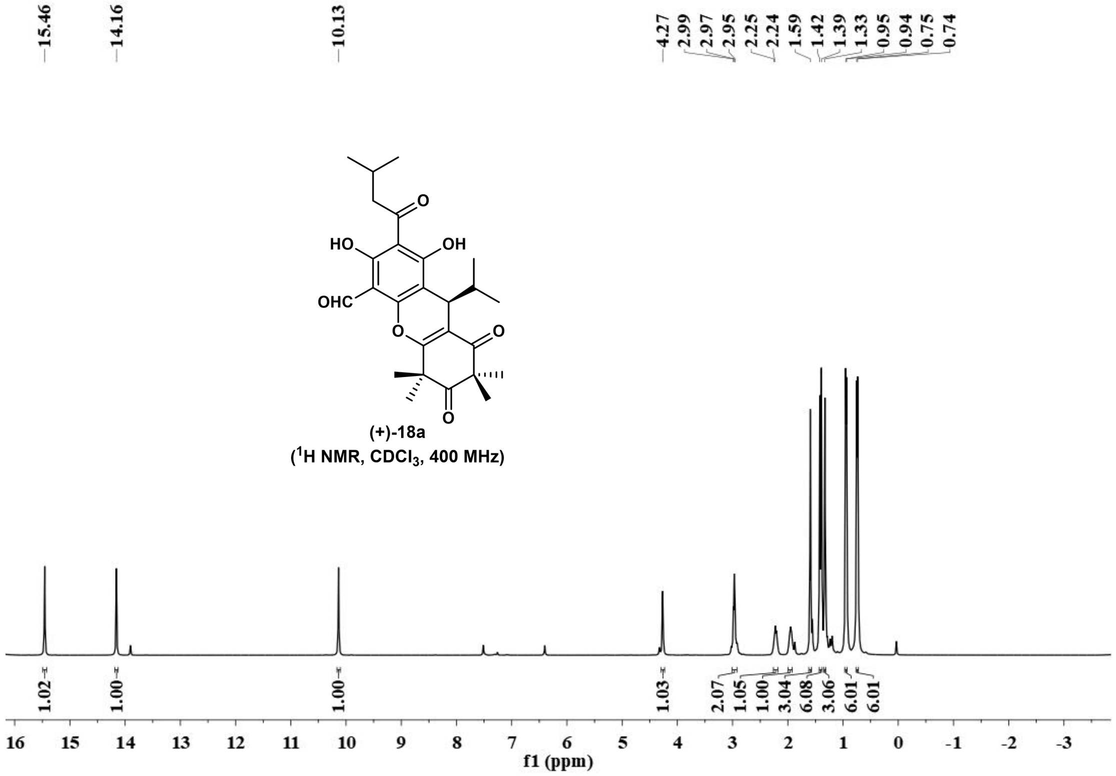

Supplementary Figure 211. ^{1}H NMR spectra of (+)-18a.

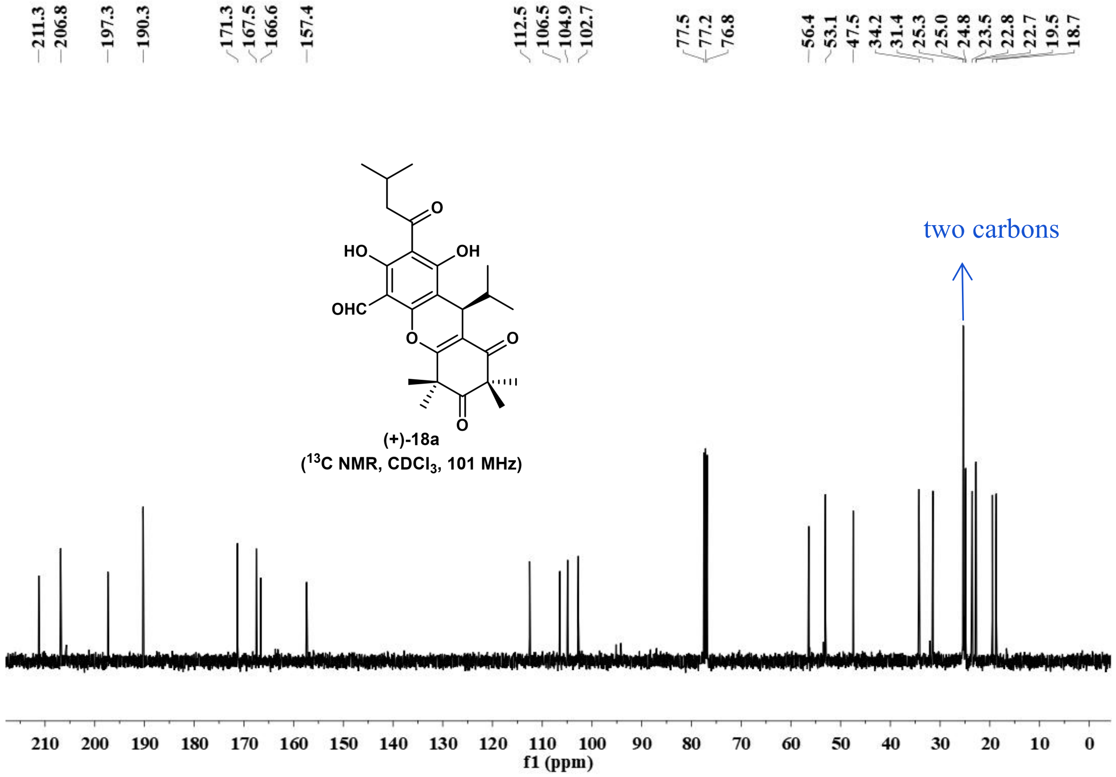

Supplementary Figure 212. ^{13}C NMR spectra of (+)-18a.

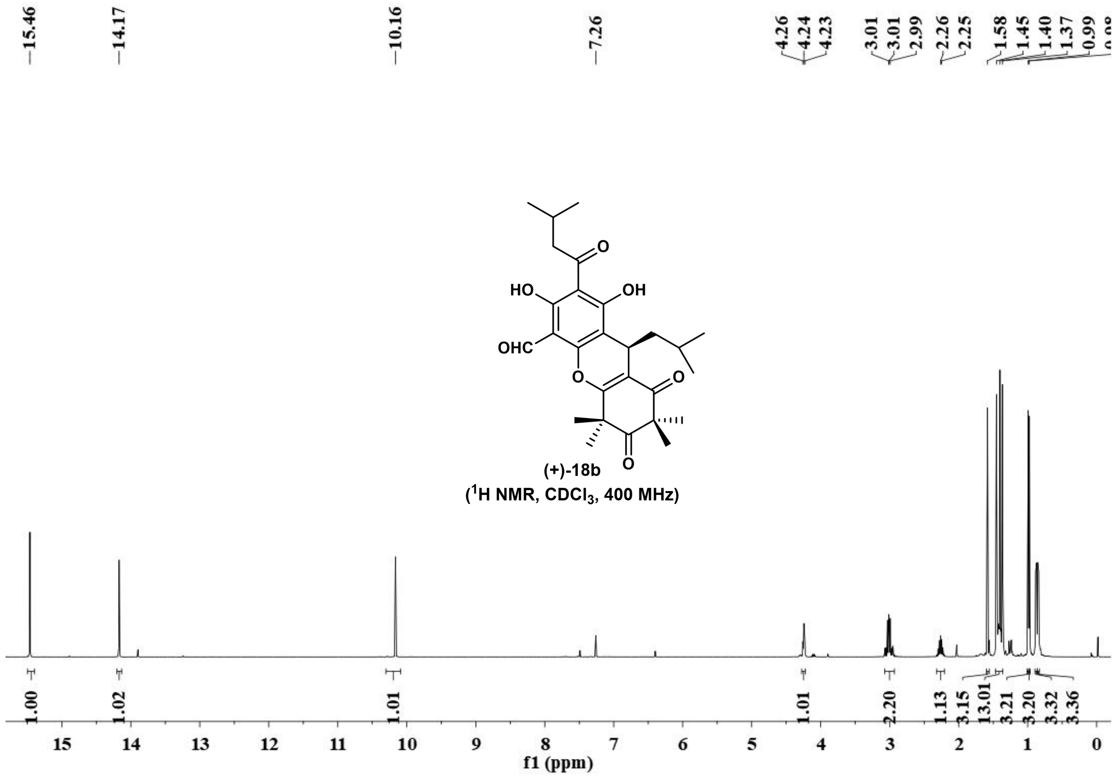

Supplementary Figure 213. ^{1}H NMR spectra of (+)-18b.

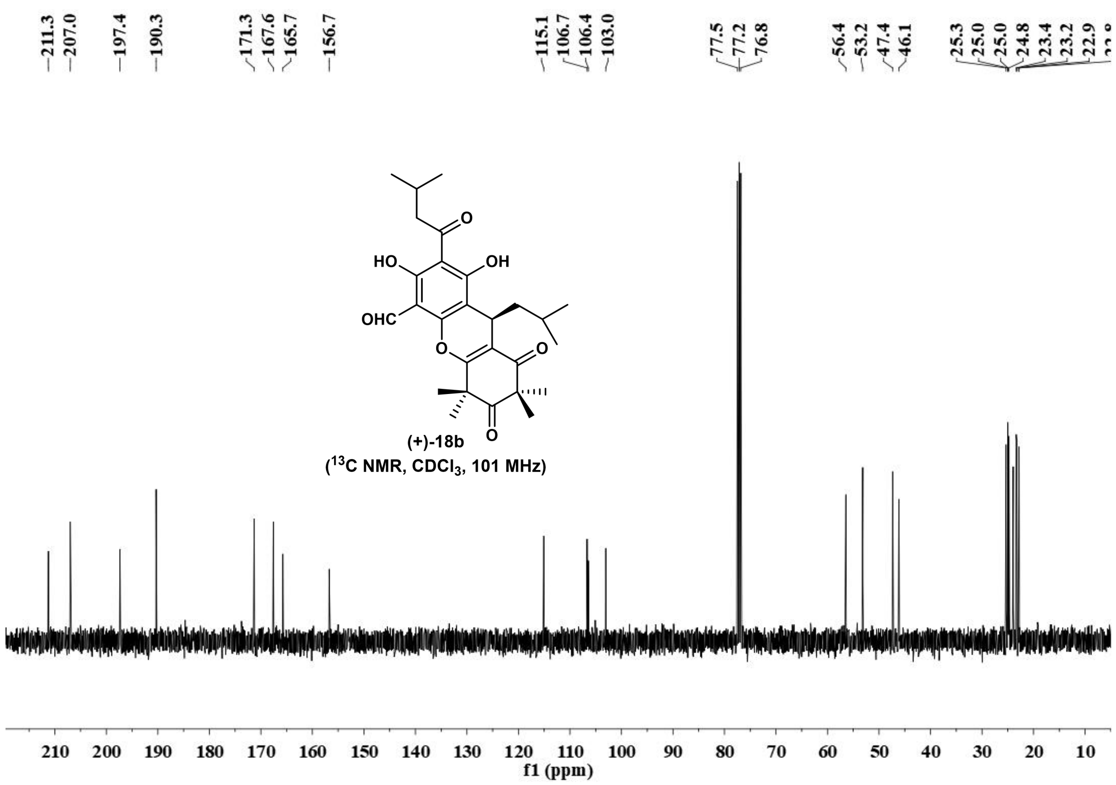

Supplementary Figure 214. ^{13}C NMR spectra of (+)-18b.

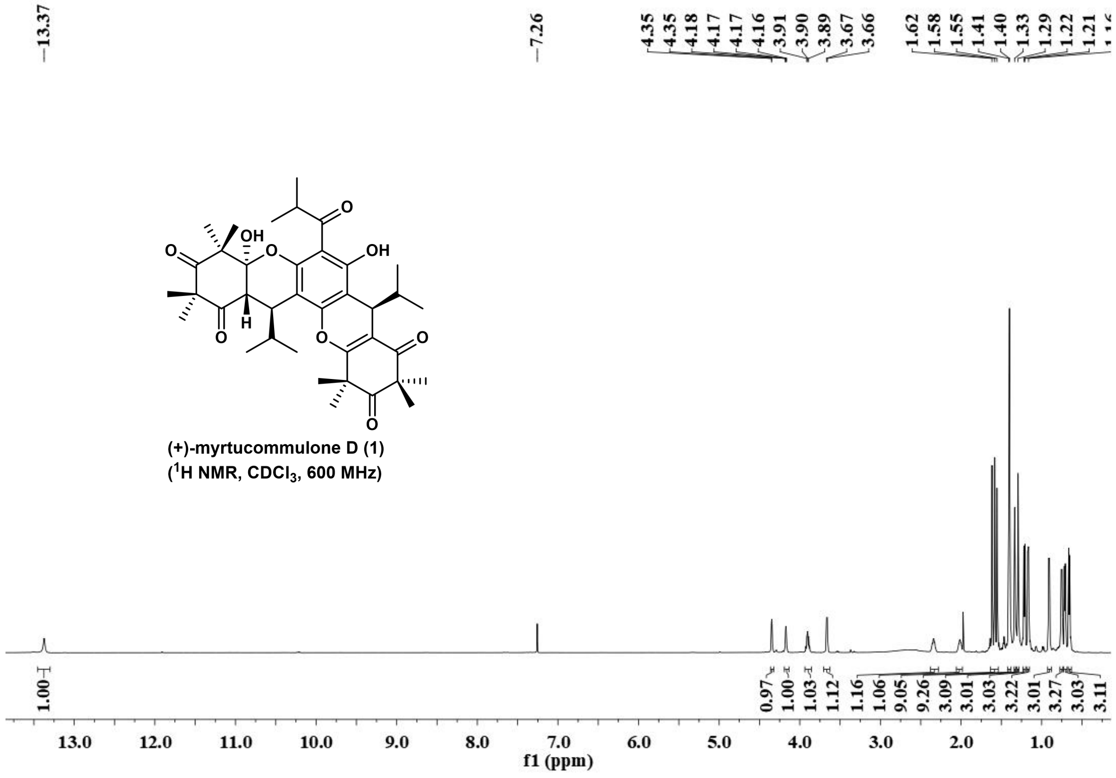

Supplementary Figure 215. ^{1}H NMR spectra of (+)-1.

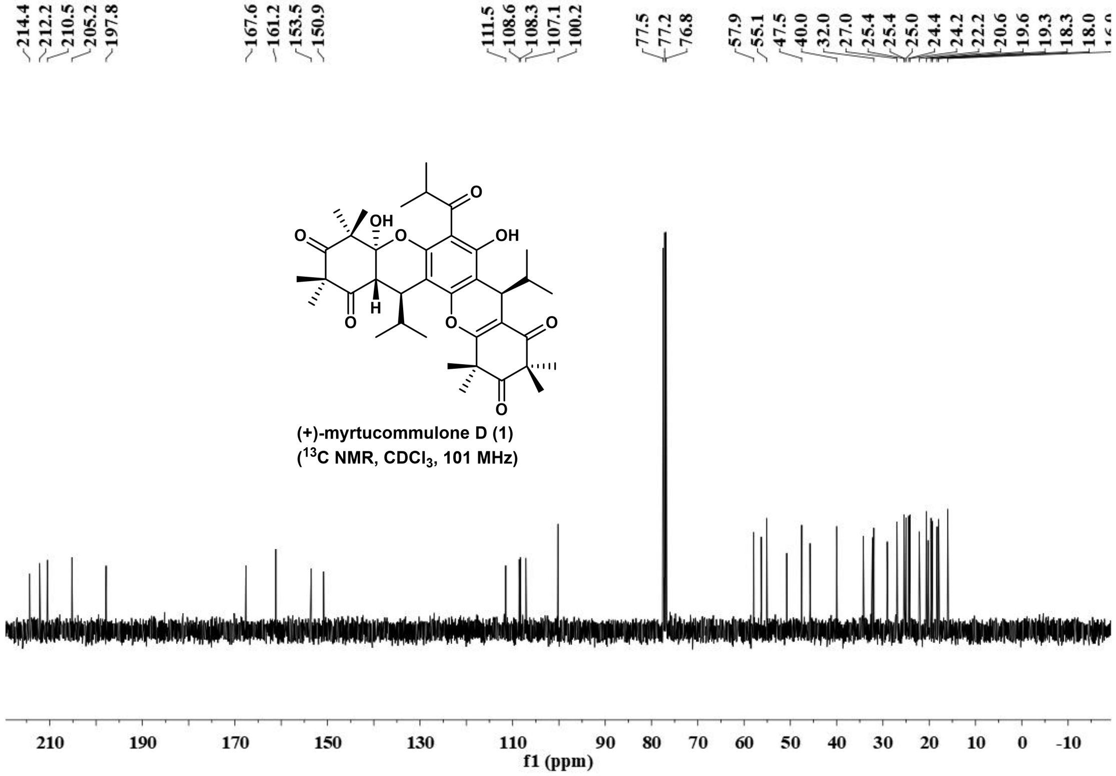

Supplementary Figure 216. ^{13}C NMR spectra of (+)-1.

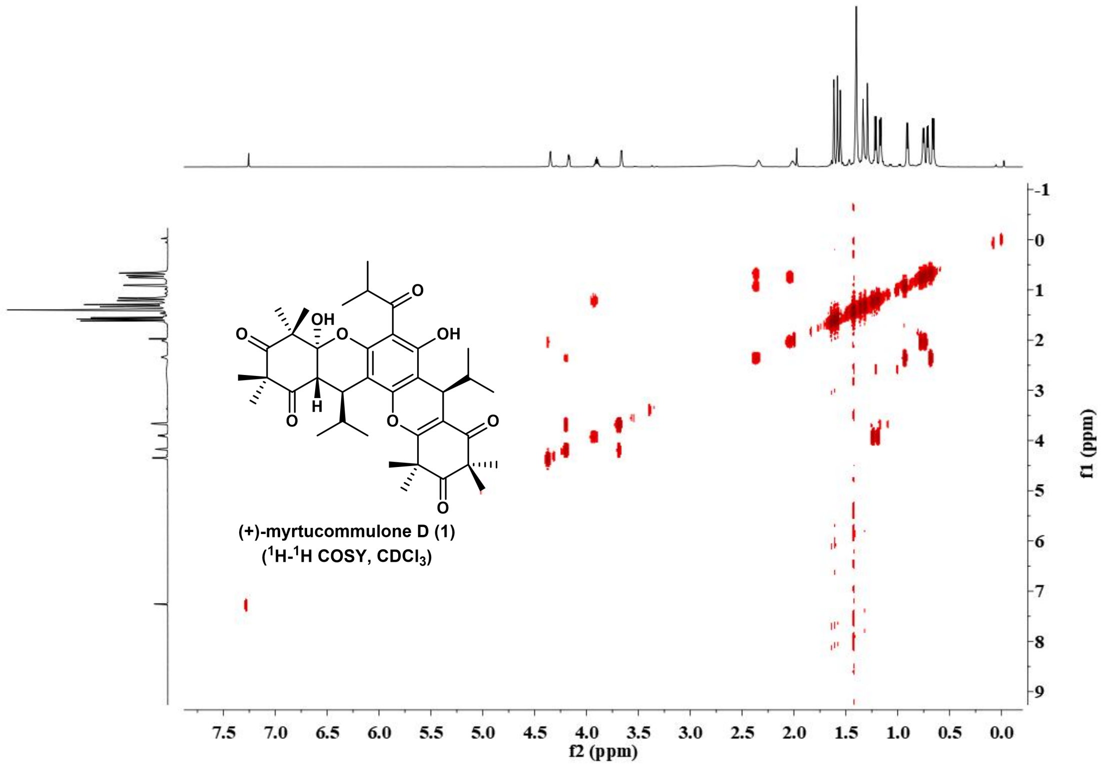

Supplementary Figure 217. ^{1}H-^{1}H COSY spectra of (+)-1.

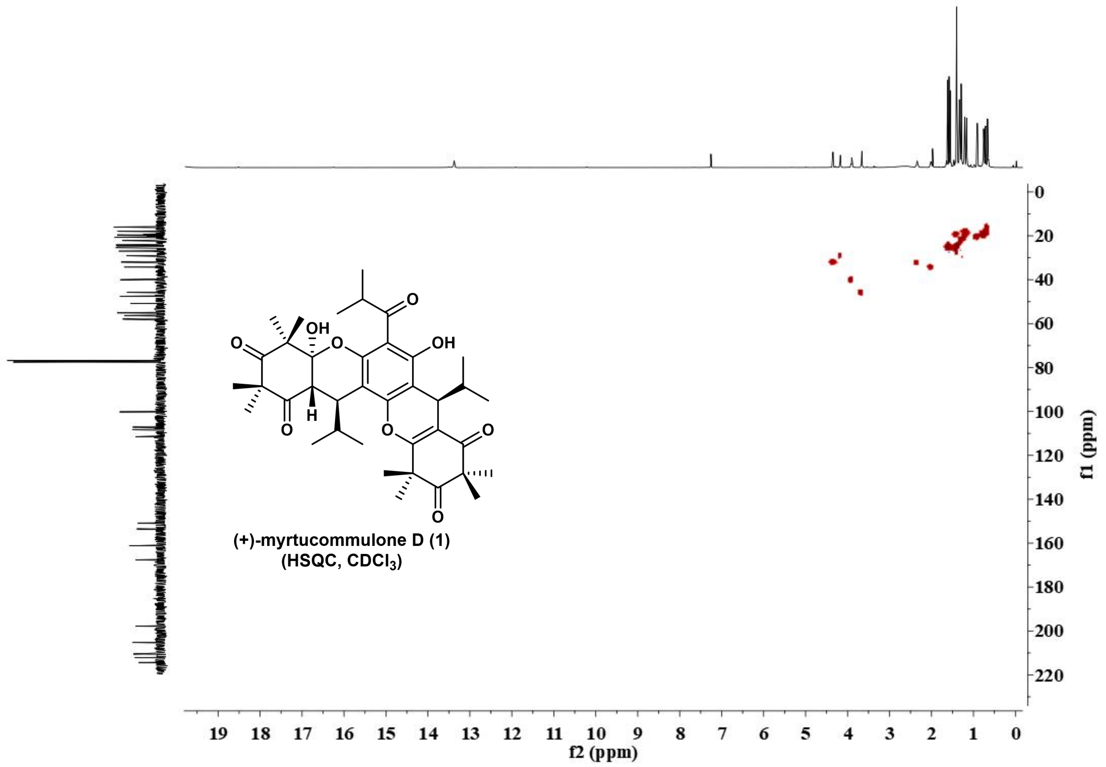

Supplementary Figure 218. HSQC spectra of (+)-1.

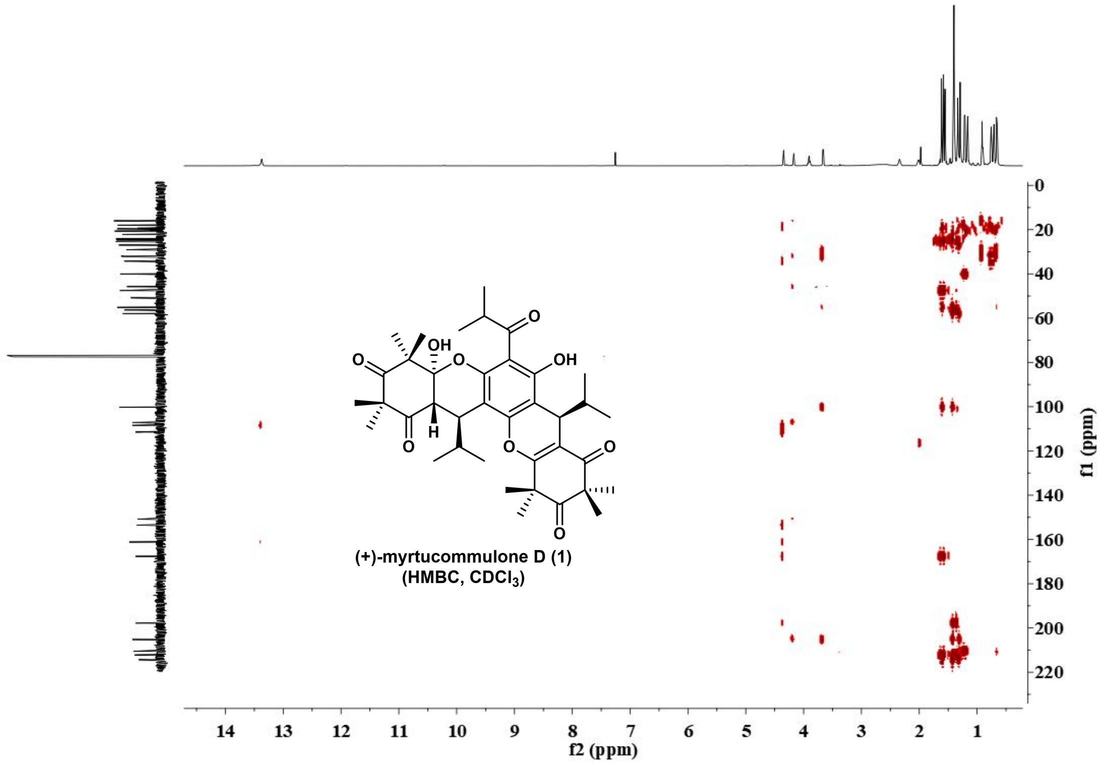

Supplementary Figure 219. HMBC spectra of (+)-1.

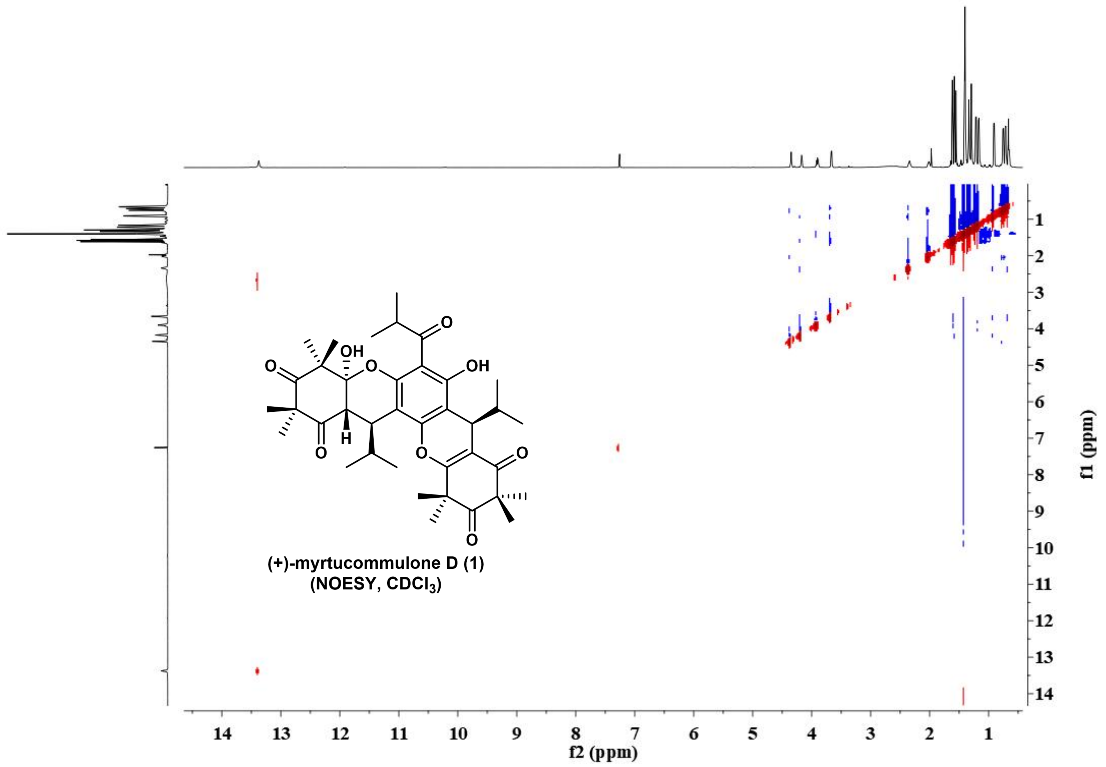

Supplementary Figure 220. NOESY spectra of (+)-1.

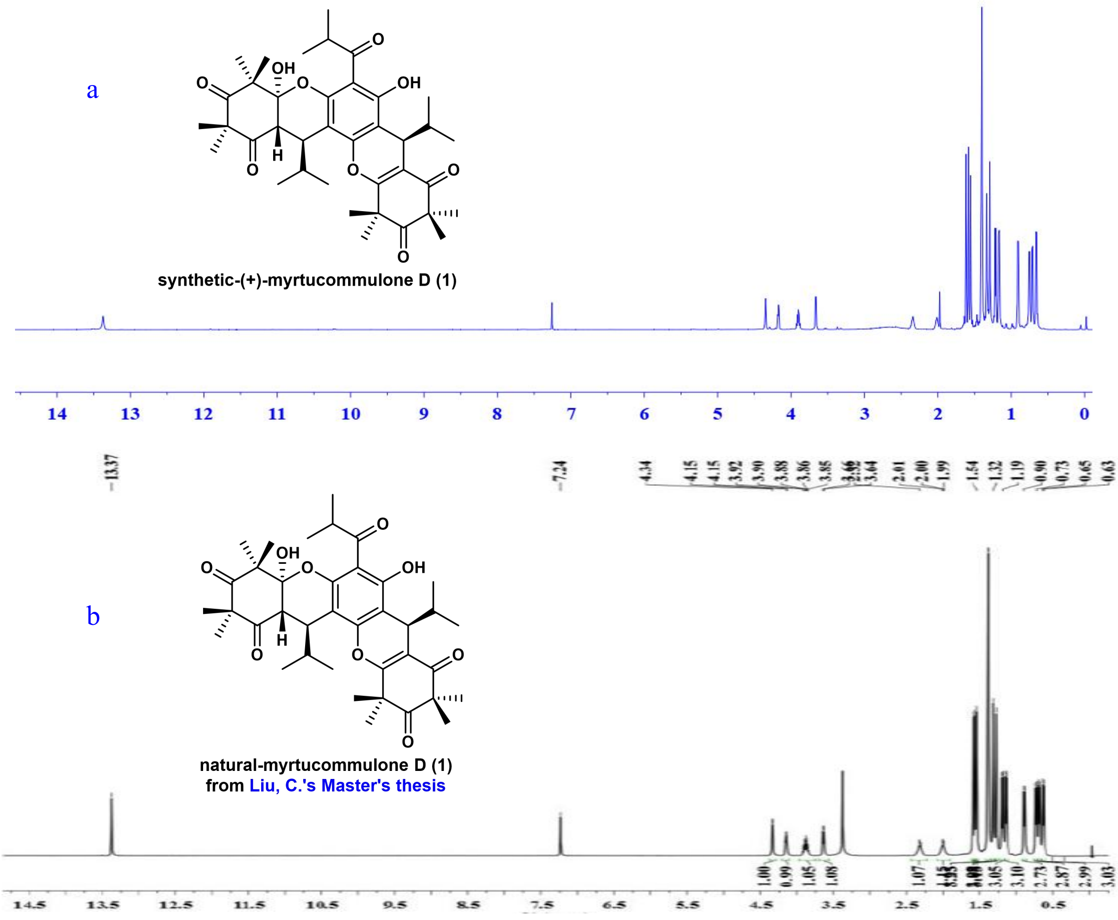

Supplementary Figure 221. Comparison of ^{1}H NMR spectra between synthetic and the

isolated 1. a, ^{1}H NMR spectra of synthetic 1. b, ^{1}H NMR spectra of isolated 1.

Note: 1. Since the ^{13}C spectra image was not provided in the reported literature, which includes Shaheen, F.'s[3], and Ni, W.'s work[4], and only the ^{1}H spectra image from [Liu, C. *Studies on Phloroglucinol Derivatives from the Twigs and Leaves of Myrtus communis (Chinese version, Master's thesis)*, Jinan University (2016).] was presented, we have included a comparative ^{1}H spectra image between our synthetic myrtucommulone D (**1**) and natural **1**.

2. Supplementary Figure 221b is reproduced from Liu, C.'s Master's thesis.

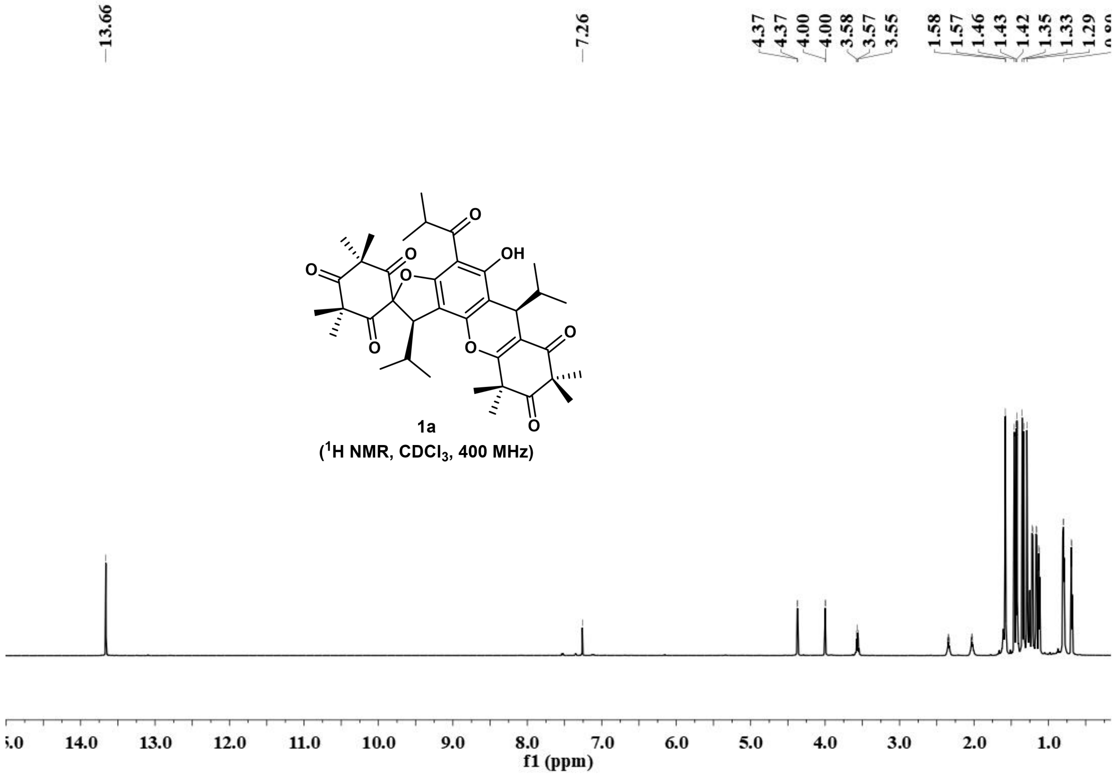

Supplementary Figure 222. ^{1}H NMR spectra of 1a.

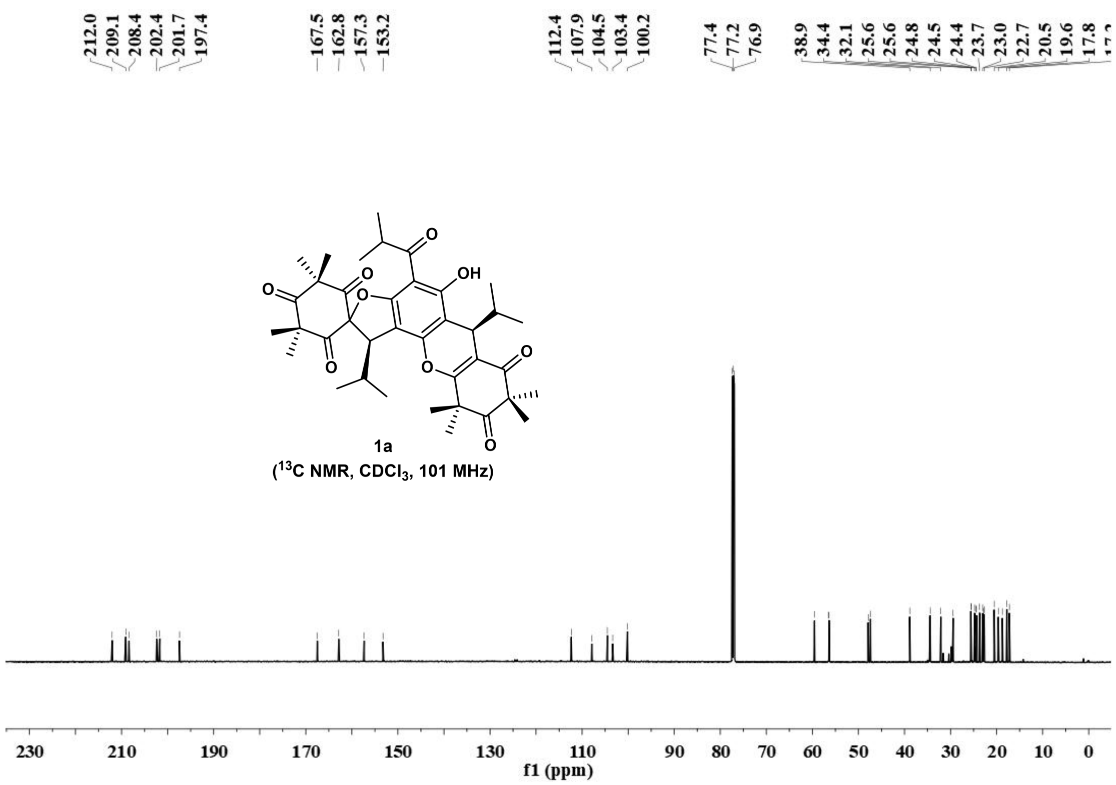

Supplementary Figure 223. ^{13}**C NMR spectra of 1a.**

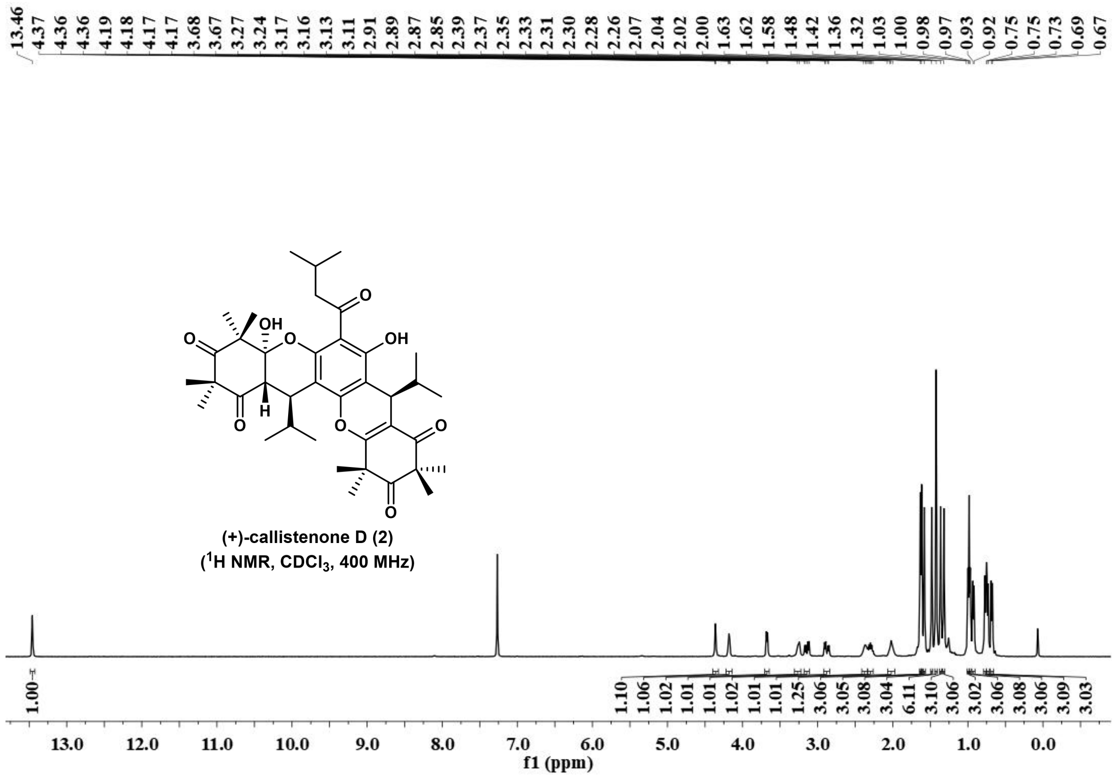

Supplementary Figure 224. ^{1}**H NMR spectra of (+)-2.**

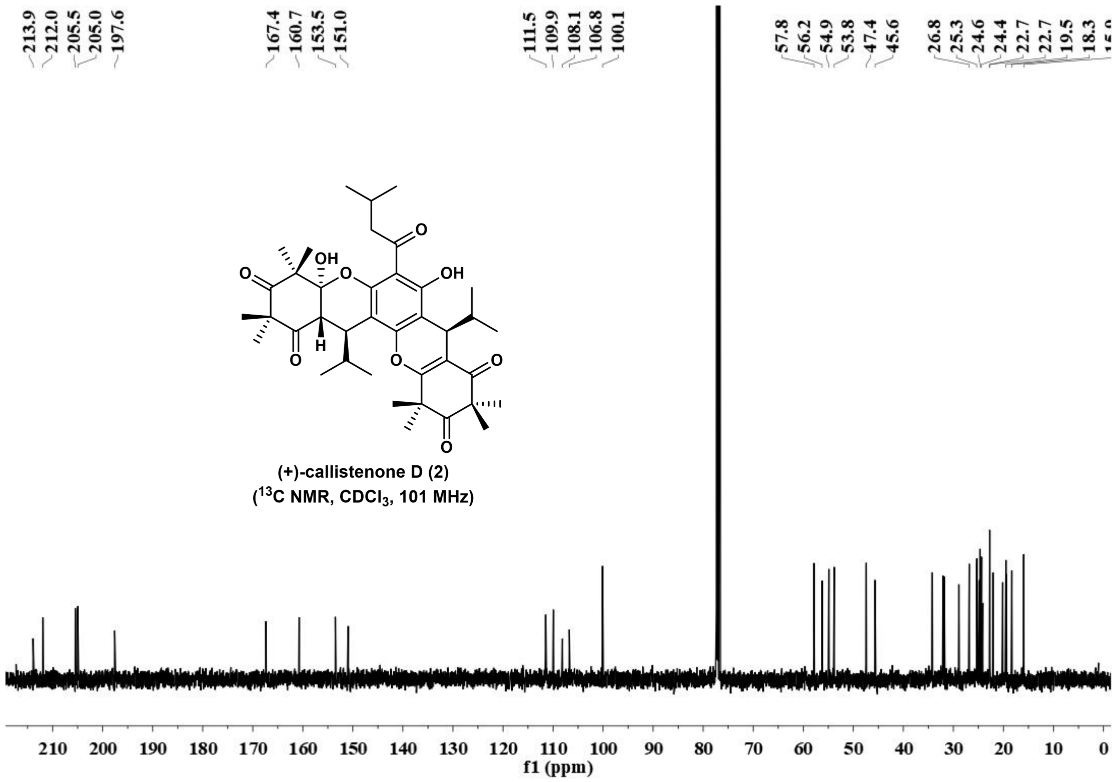

Supplementary Figure 225. ^{13}**C NMR spectra of (+)-2.**

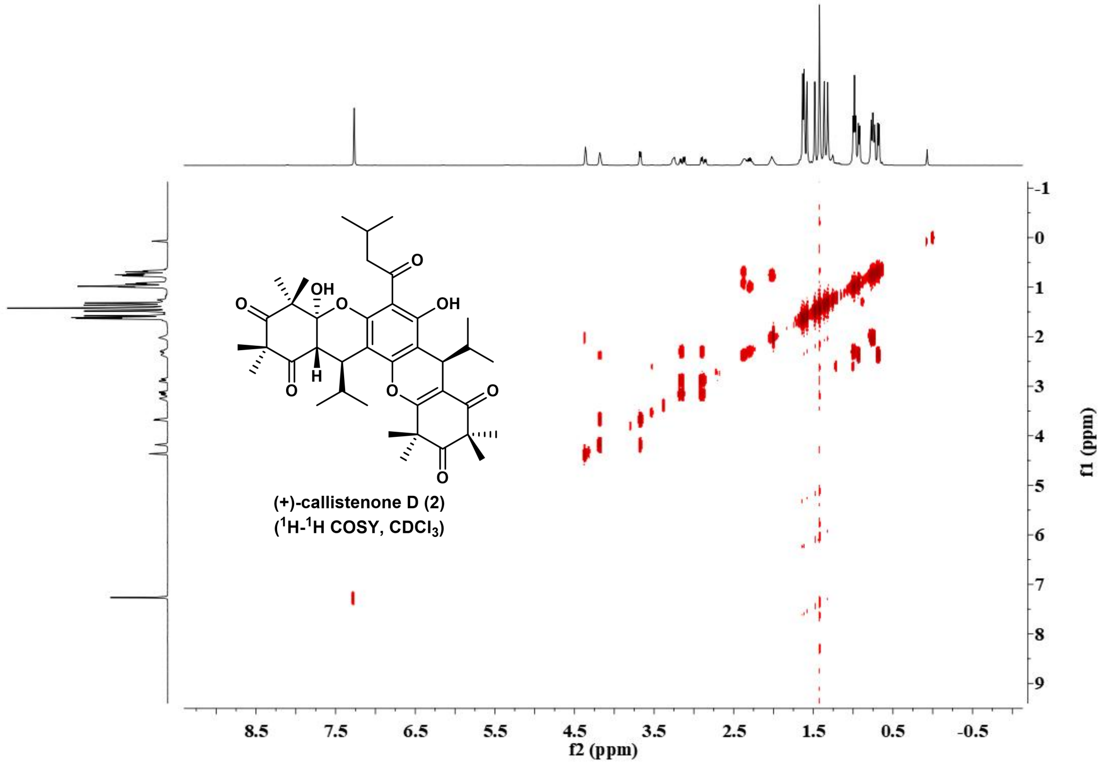

Supplementary Figure 226. ^{1}**H-**^{1}**H COSY spectra of (+)-2.**

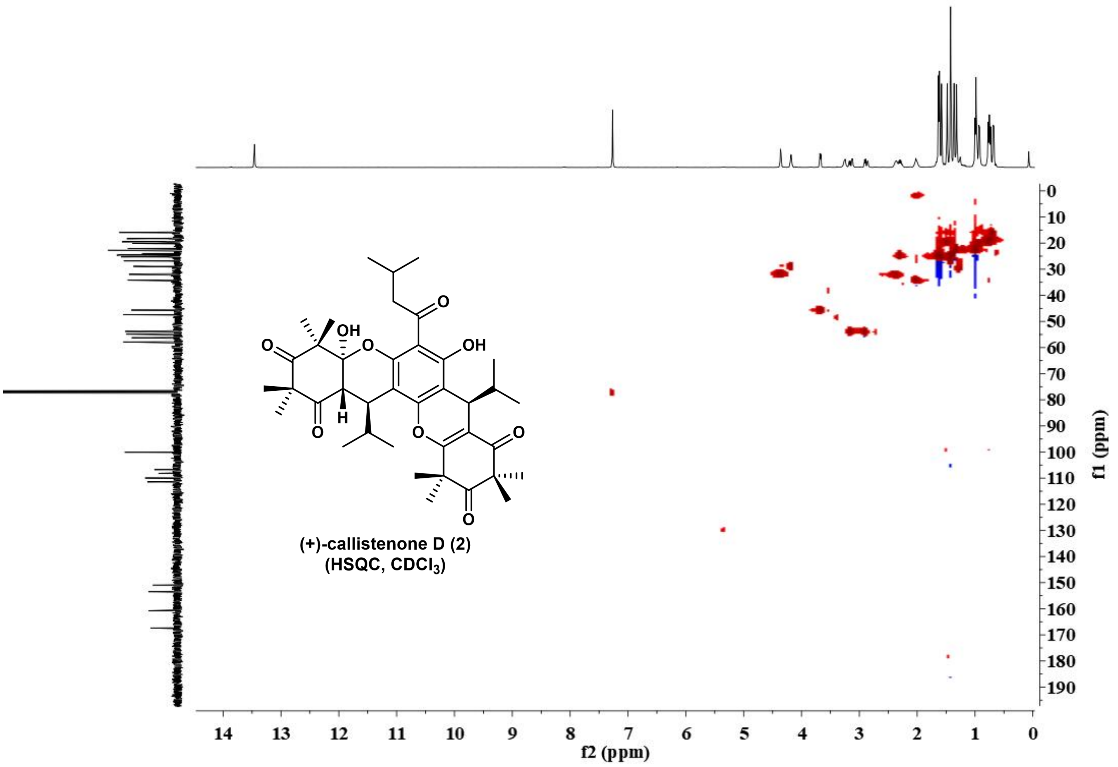

Supplementary Figure 227. HSQC spectra of (+)-2.

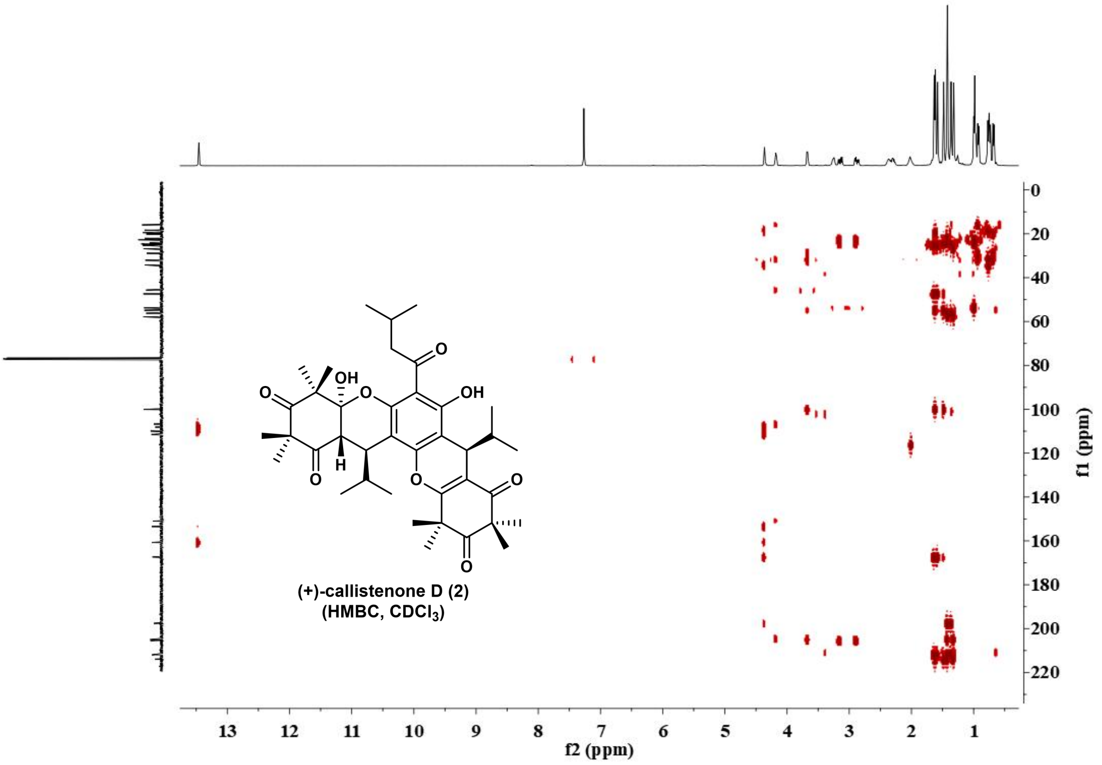

Supplementary Figure 228. HMBC spectra of (+)-2.

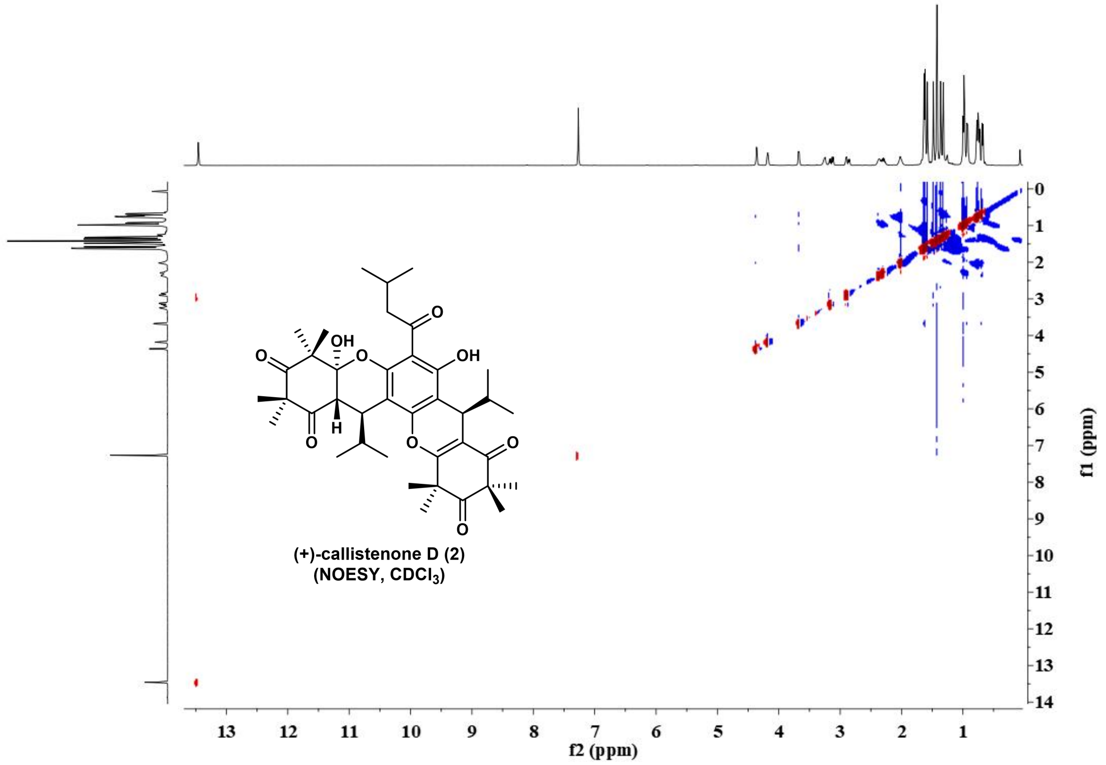

Supplementary Figure 229. NOESY spectra of (+)-2.

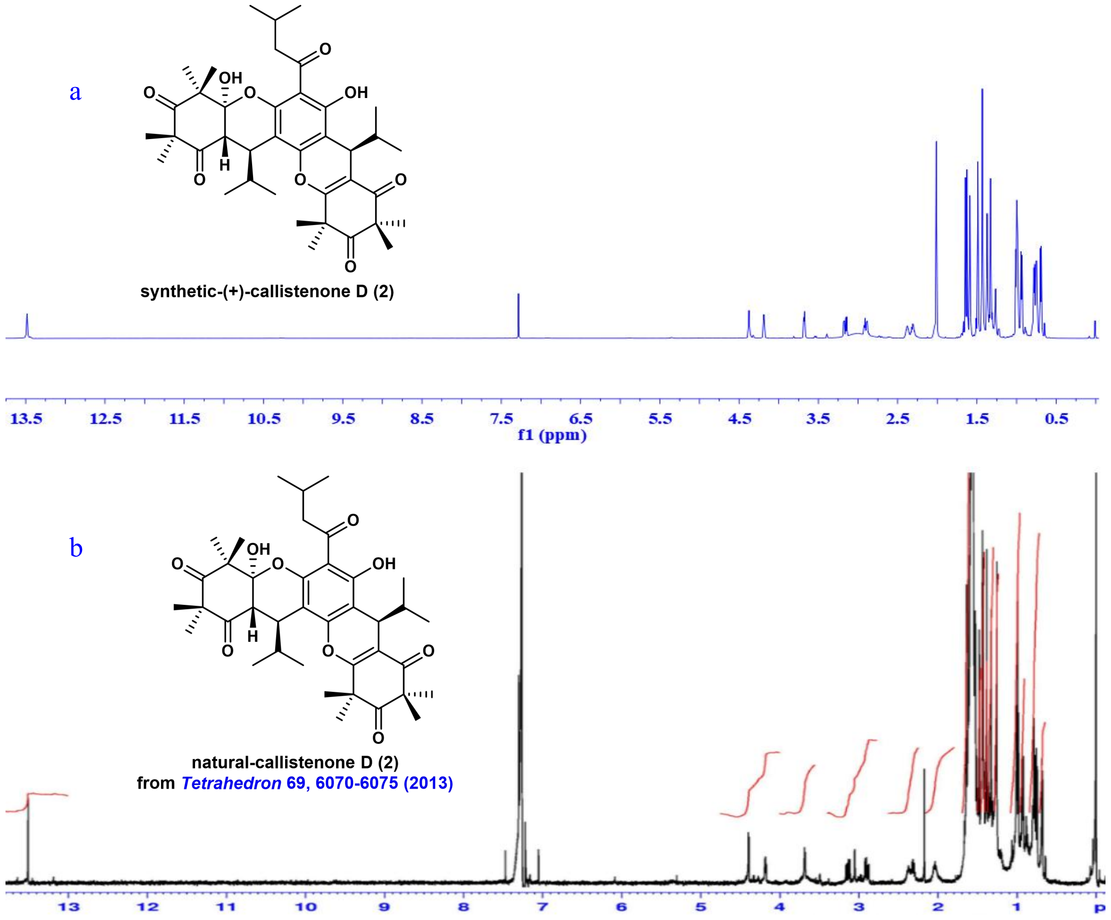

Supplementary Figure 230. Comparison of ^{1}H NMR spectra between synthetic and the

isolated 2. a, ^{1}H NMR spectra of synthetic 2. b, ^{1}H NMR spectra of isolated 2.

Note: 1. Since the ^{13}C spectra image was not provided in the reported literature, which includes Ni, W.'s[4] and Carroll, A. R.'s[5] work, and only the ^{1}H spectra image from Carroll, A. R.'s work was presented, possibly due to the compound's relative scarcity in nature (1.8 mg) , we have included a comparative ^{1}H spectra image between our synthetic callistenone D (**2**) and natural **2**.

2. Supplementary Figure 230b is reproduced from *Tetrahedron* 69, 6070-6075 (2013).

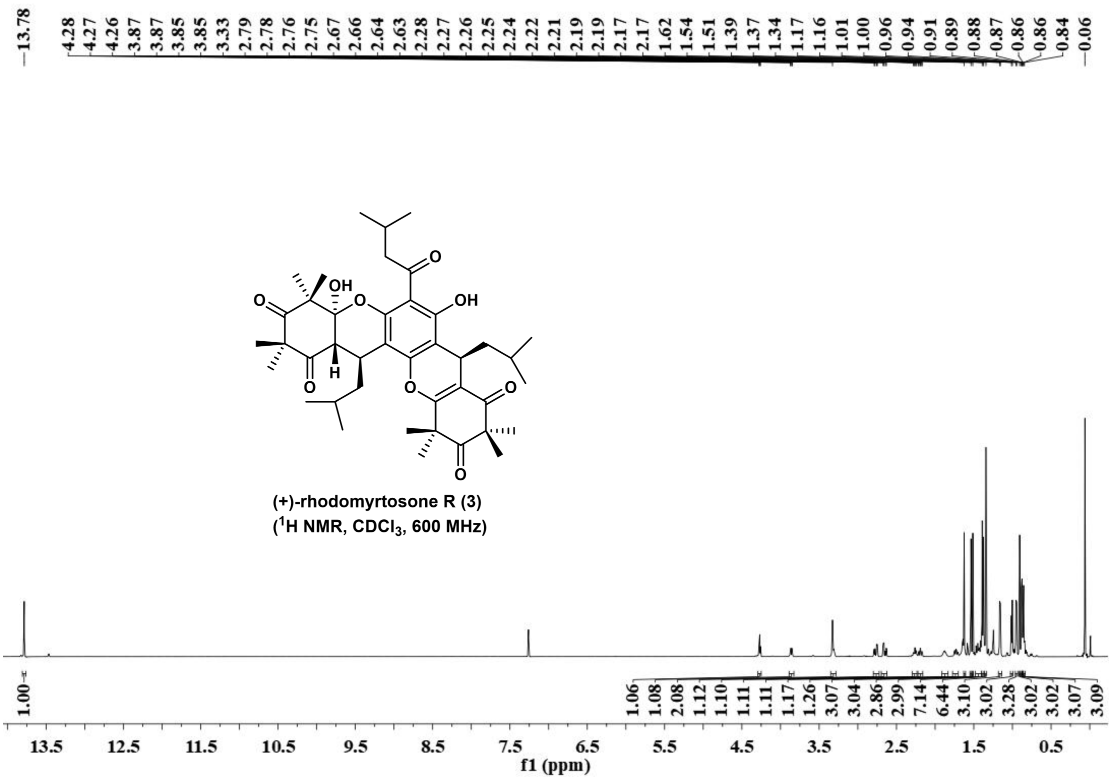

Supplementary Figure 231. ^{1}H NMR spectra of (+)-3.

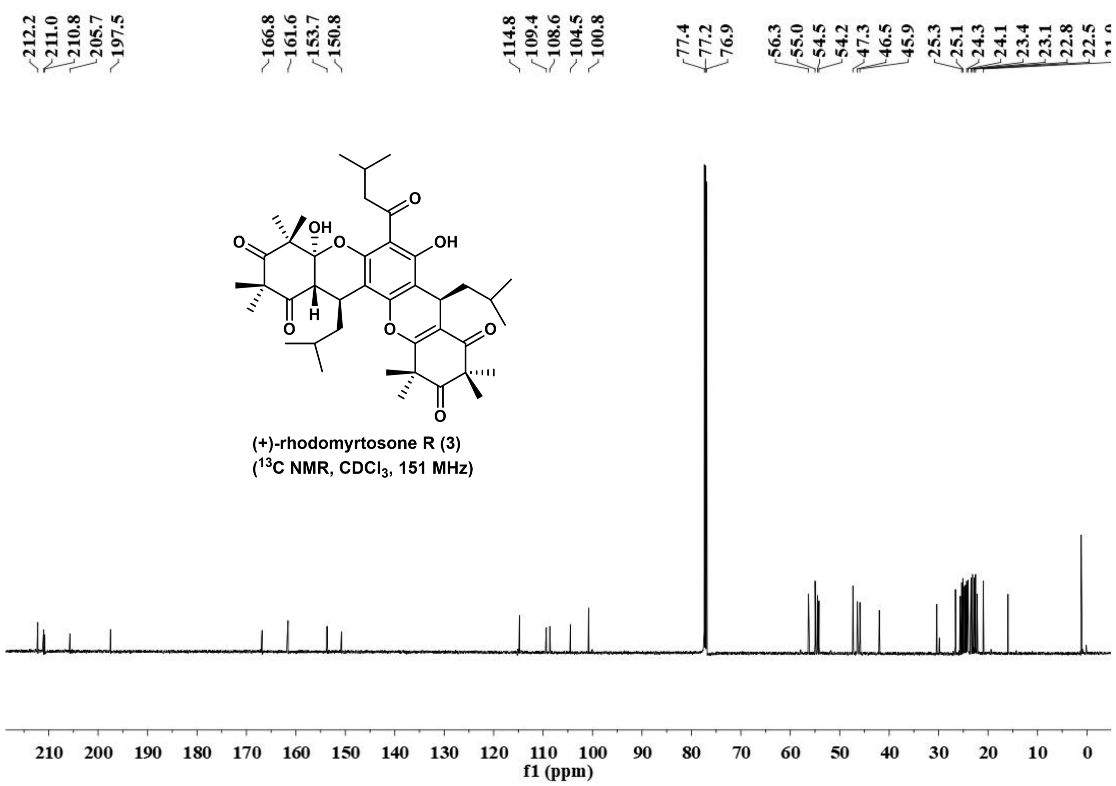

Supplementary Figure 232. ^{13}**C NMR spectra of (+)-3.**

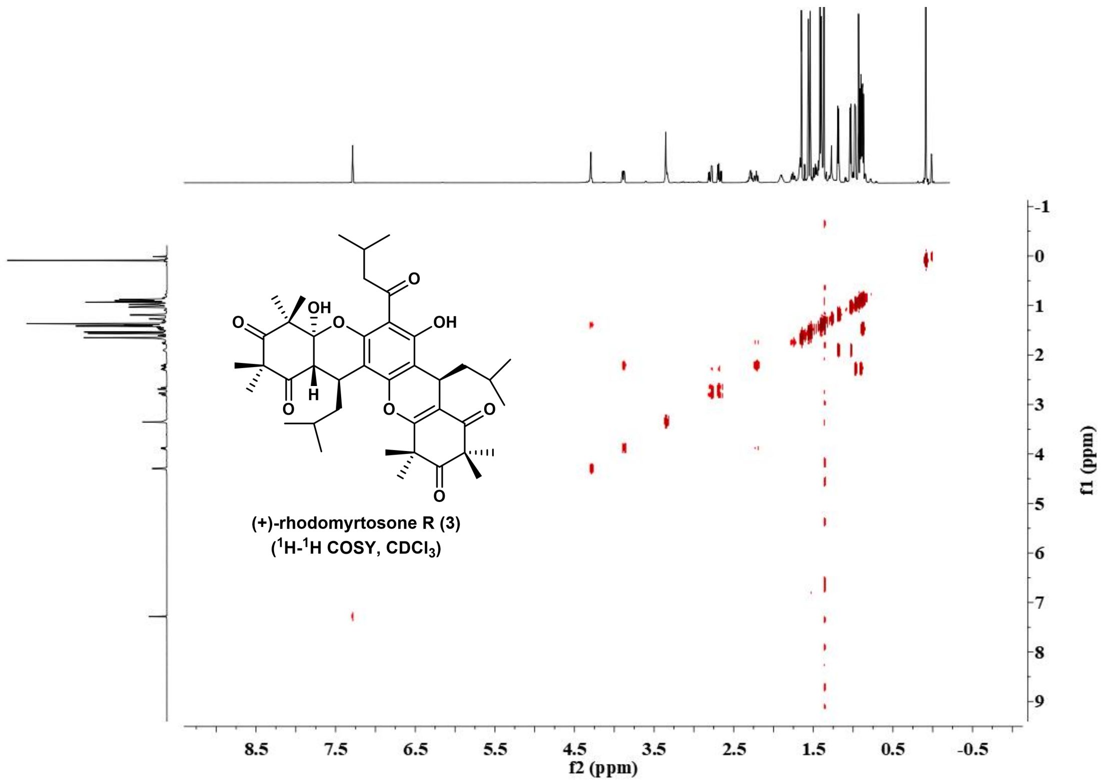

Supplementary Figure 233. ^{1}**H-**^{1}**H spectra of (+)-3.**

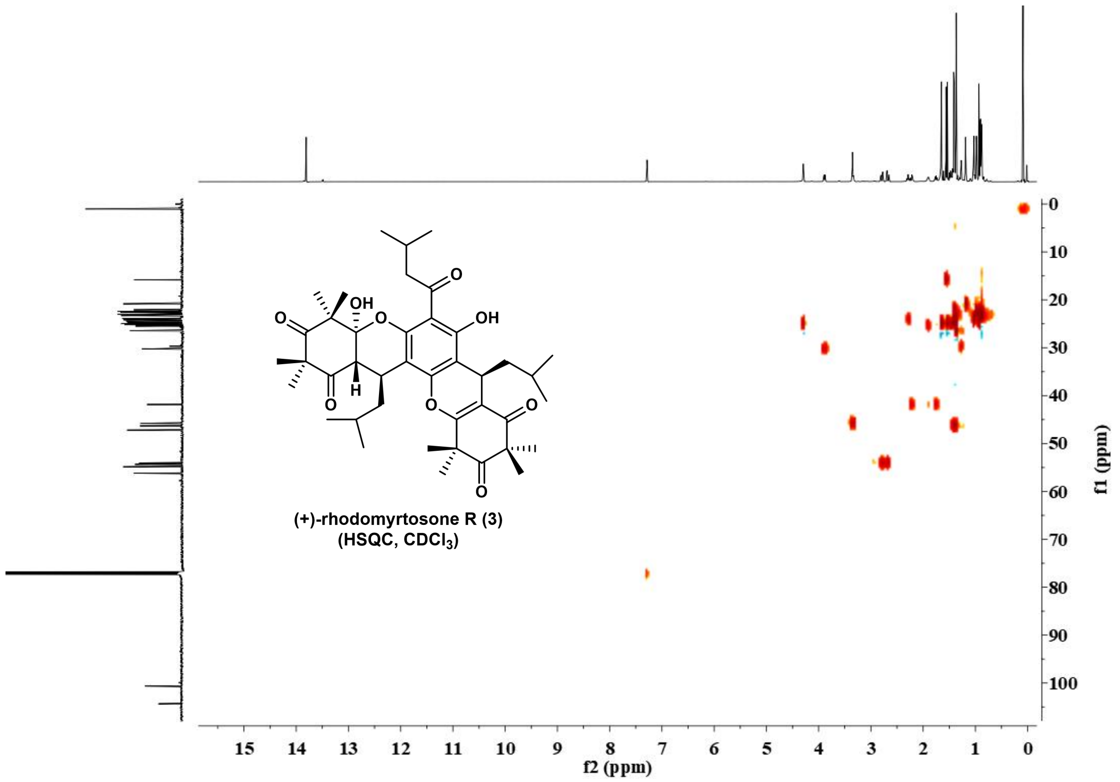

Supplementary Figure 234. HSQC spectra of (+)-3.

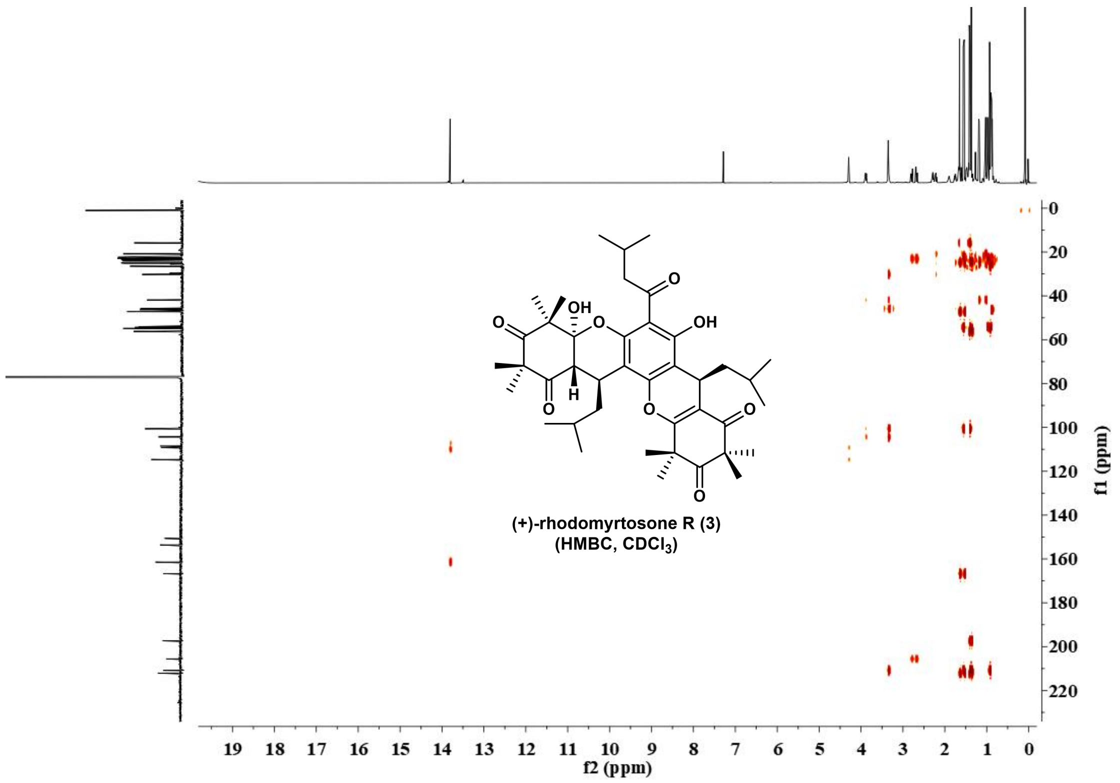

Supplementary Figure 235. HMBC spectra of (+)-3.

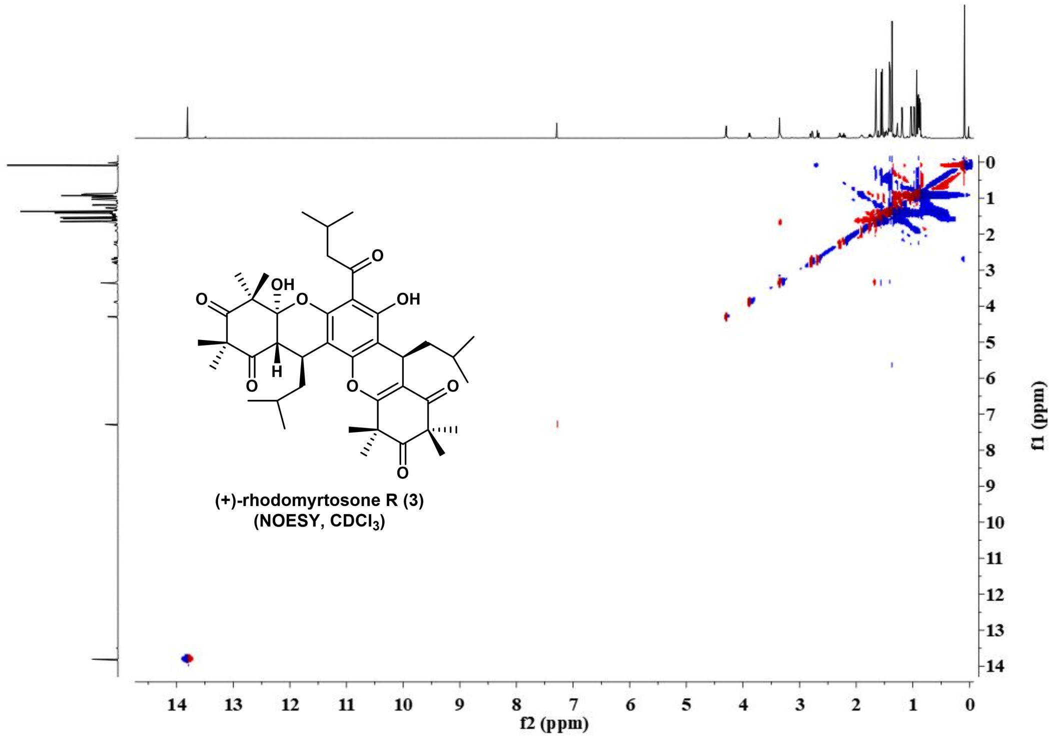

Supplementary Figure 236. NOESY spectra of (+)-3.

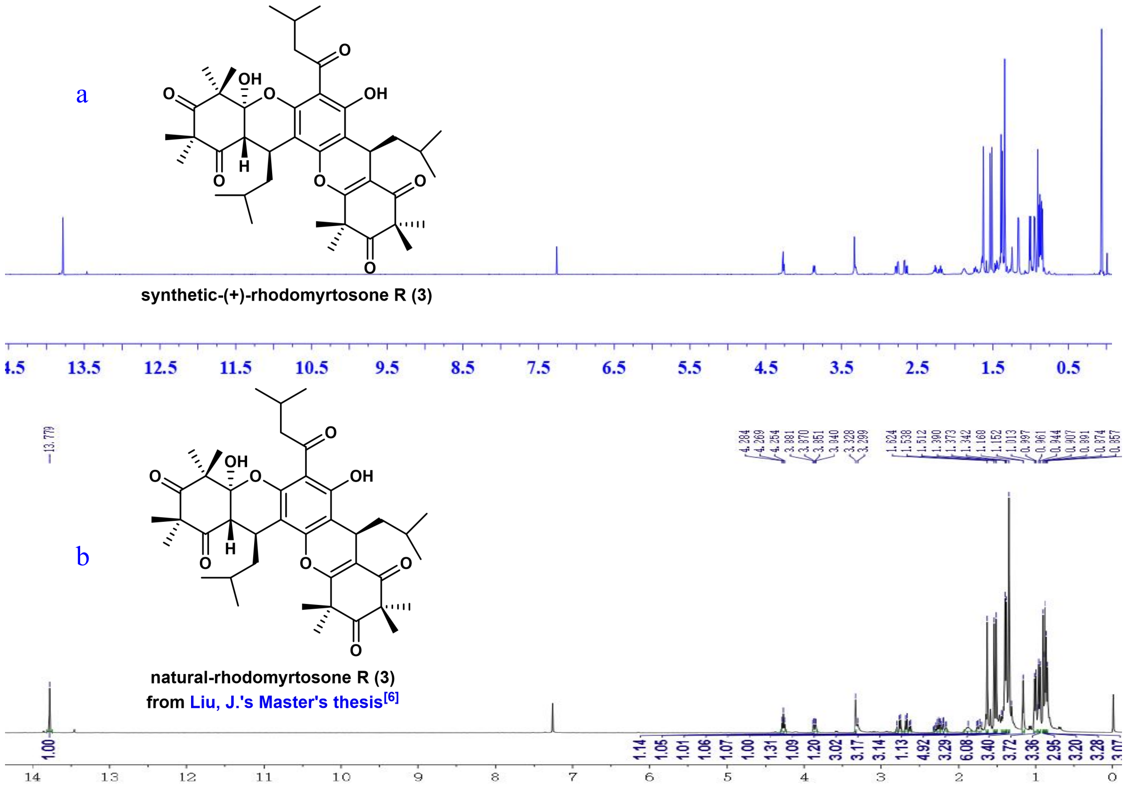

Supplementary Figure 237. Comparison of ^{1}H NMR spectra between synthetic and the isolated 3. a, ^{1}H NMR spectra of synthetic 3. b, ^{1}H NMR spectra of isolated 3.

Note: Supplementary Figure 237b is reproduced from Liu, J.'s Master's thesis[6].

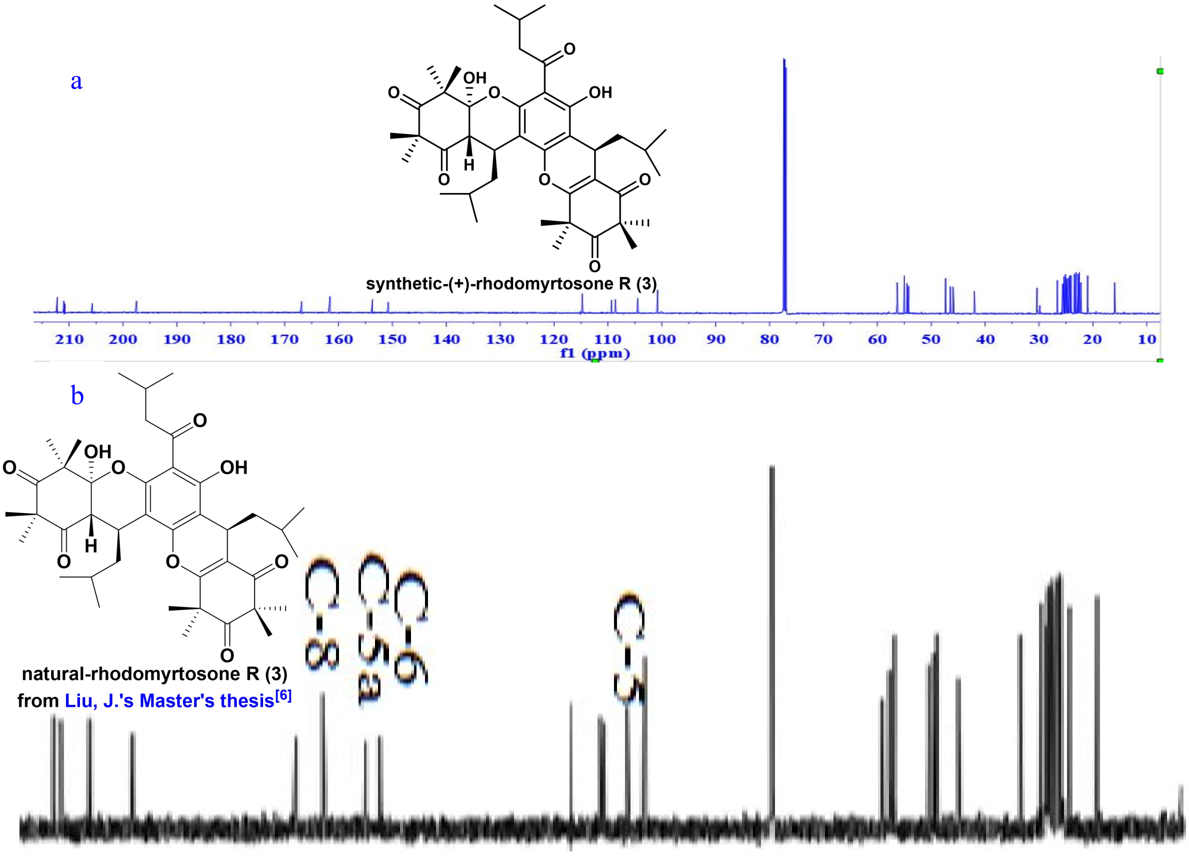

Supplementary Figure 238. Comparison of ^{13}C NMR spectra between synthetic and the isolated 3. a, ^{13}C NMR spectra of synthetic 3. b, ^{13}C NMR spectra of isolated 3.

Note: Supplementary Figure 238b is reproduced from Liu, J.'s Master's thesis[6].

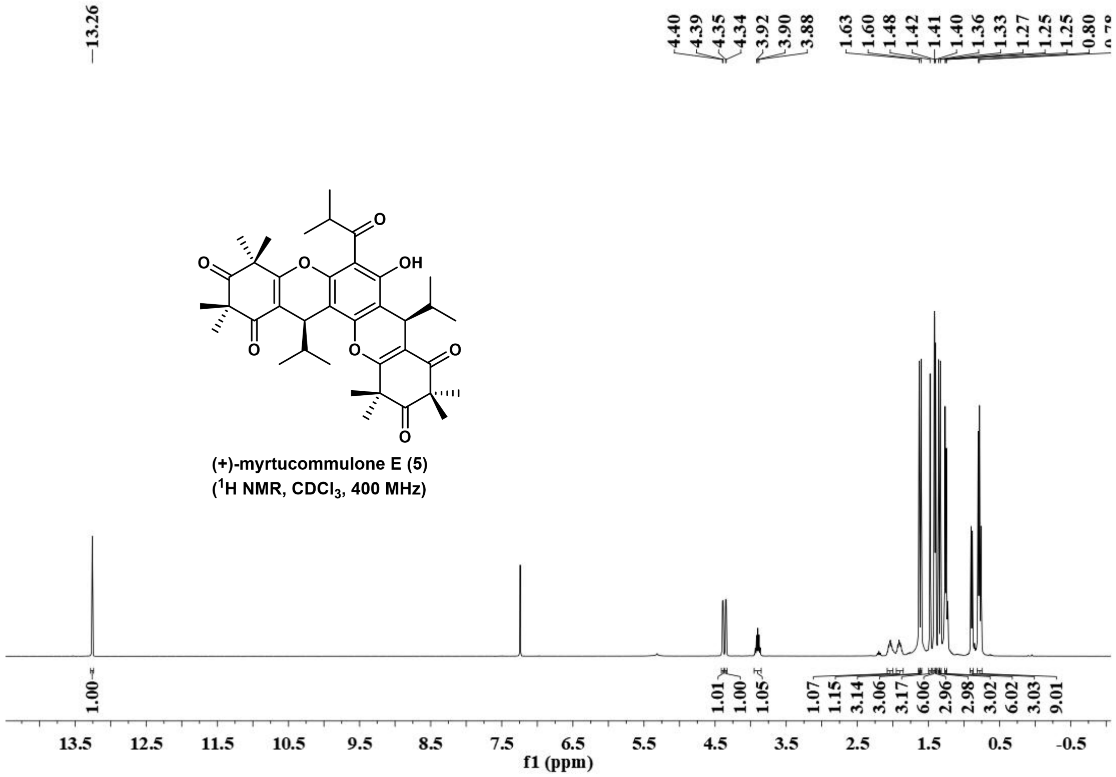

Supplementary Figure 239. ^{1}H spectra of (+)-5.

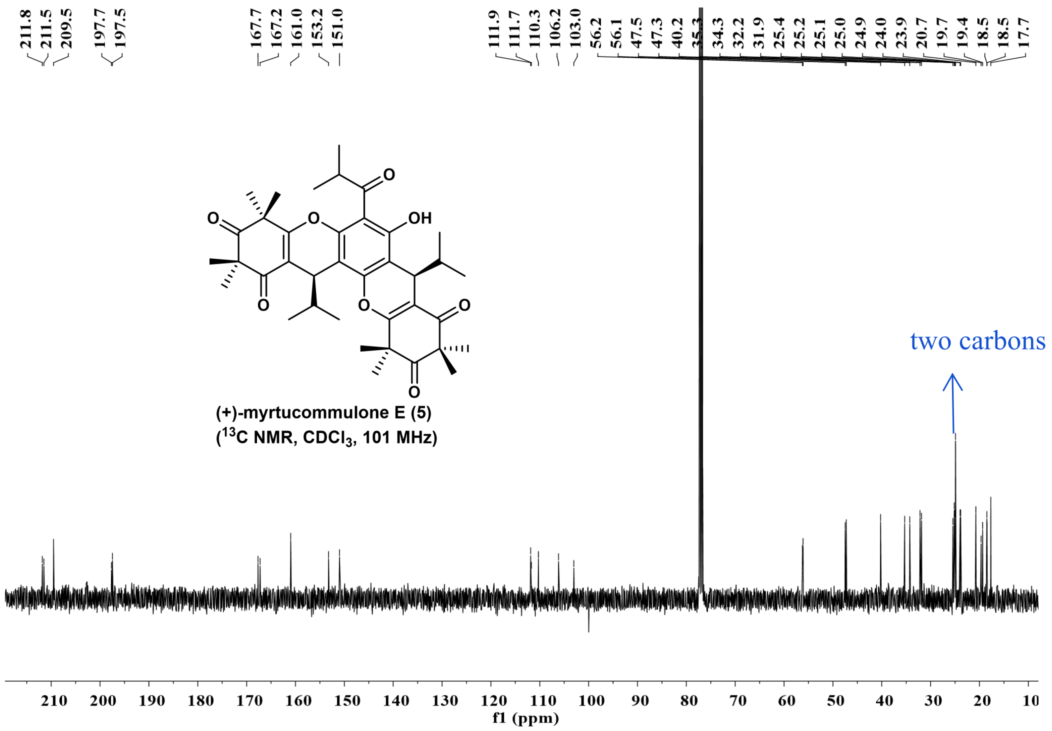

Supplementary Figure 240. ^{13}C spectra of (+)-5.

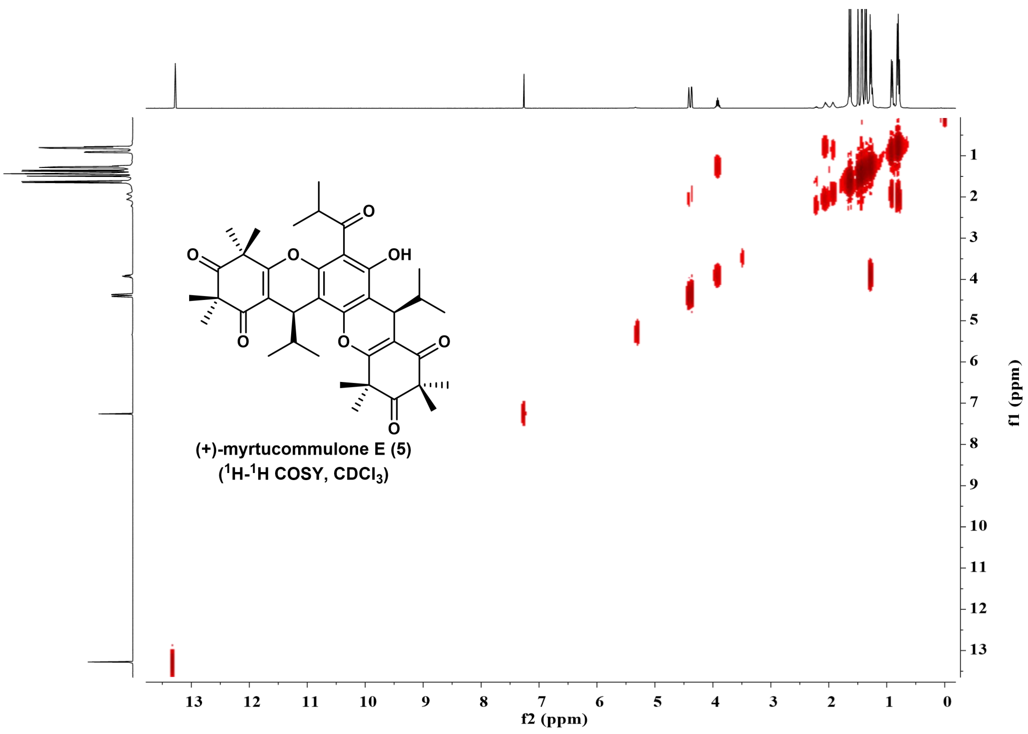

Supplementary Figure 241. ^{1}H-^{1}H COSY spectra of (+)-5.

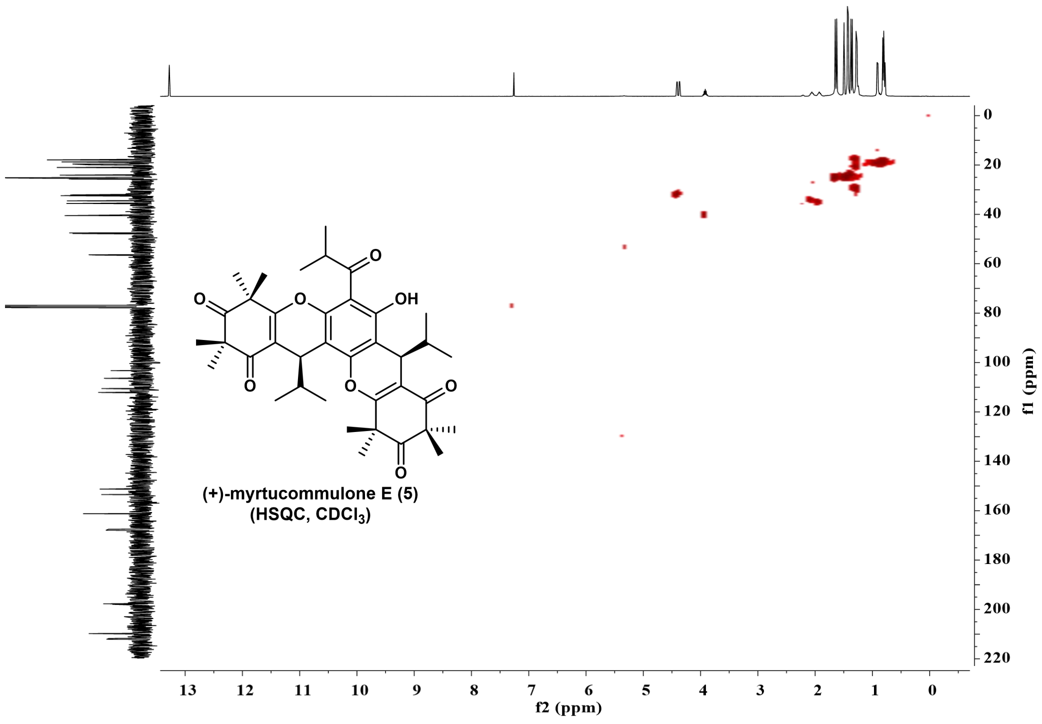

Supplementary Figure 242. HSQC spectra of (+)-5.

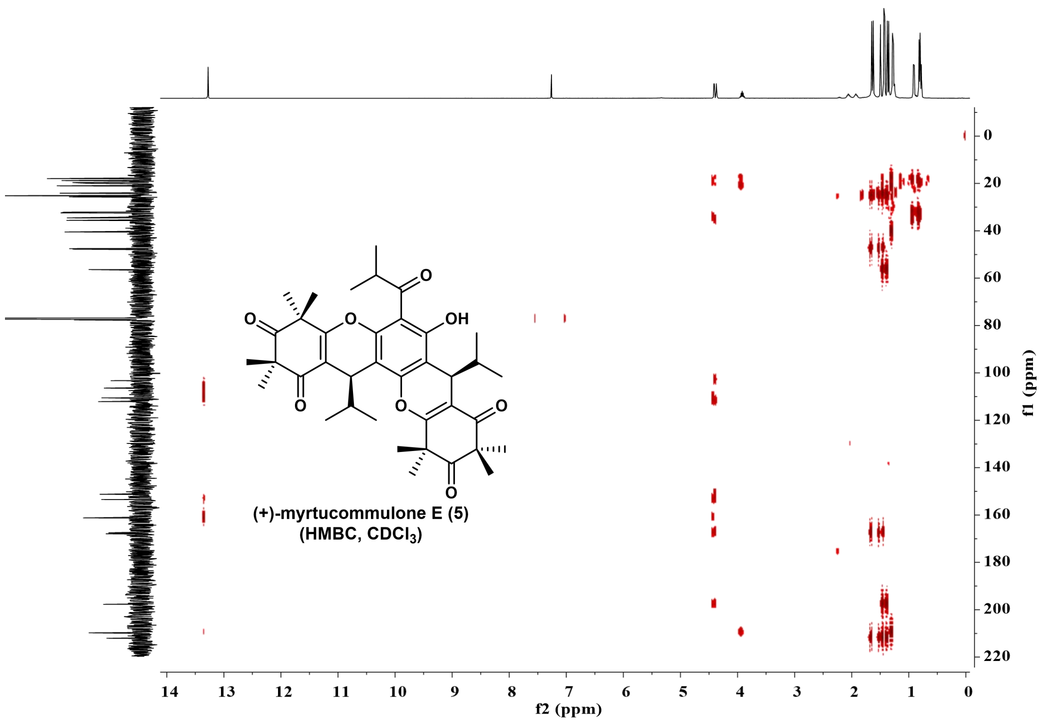

Supplementary Figure 243. HMBC spectra of (+)-5.

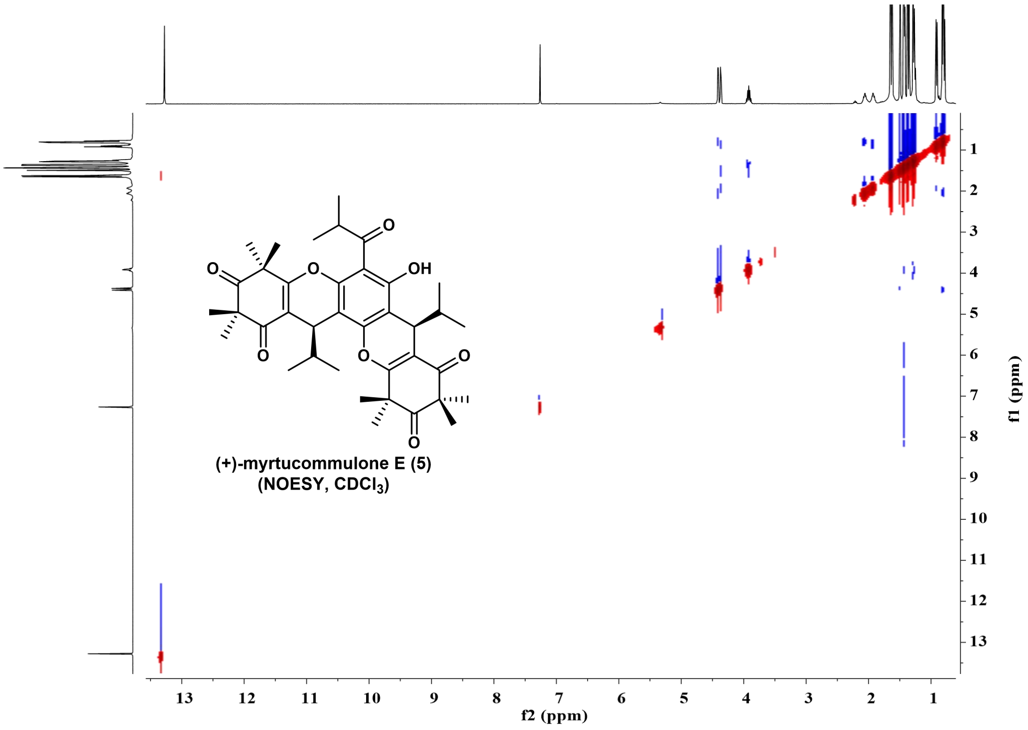

Supplementary Figure 244. NOESY spectra of (+)-5.

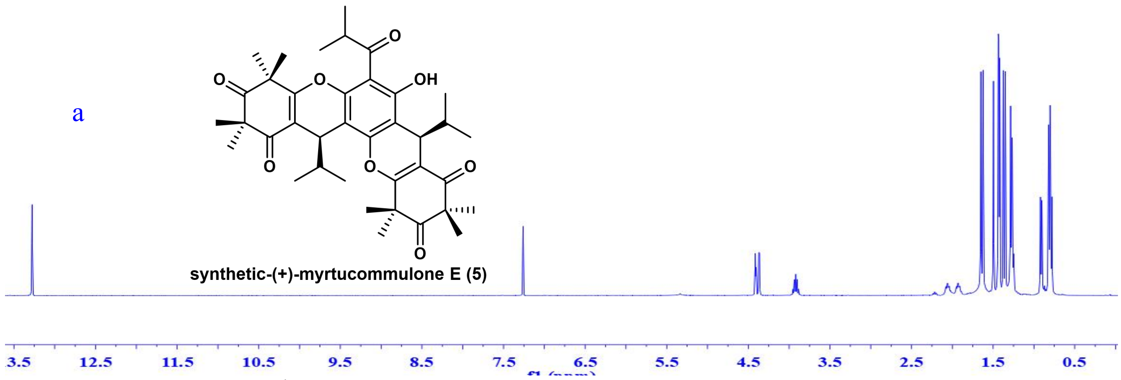

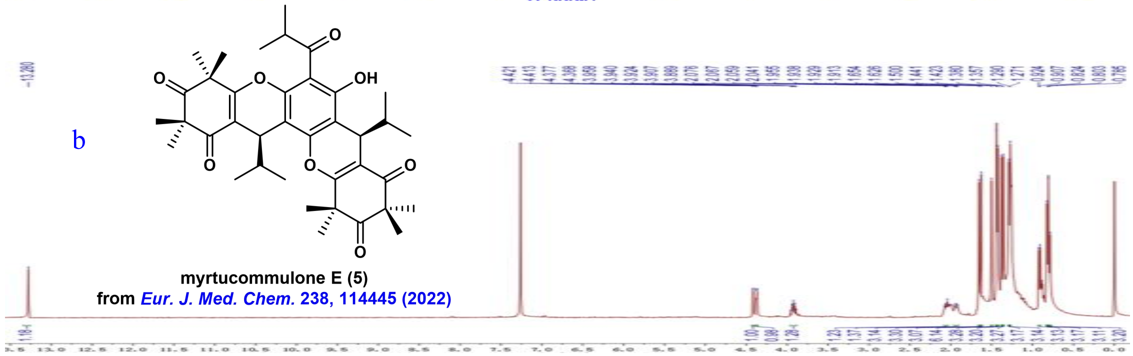

Supplementary Figure 245. Comparison of ^{1}H NMR spectra between synthetic and the reported 5. a, ^{1}H NMR spectra of synthetic 5. b, ^{1}H NMR spectra of reported 5.

Note: Supplementary Figure 245b is reproduced from Zhang, Y. et al. Discovery, enantioselective synthesis of myrtucommulone E analogues as tyrosyl-DNA phosphodiesterase 2 inhibitors and their biological activities. *Eur. J. Med. Chem.* **238**, 114445 (2022).

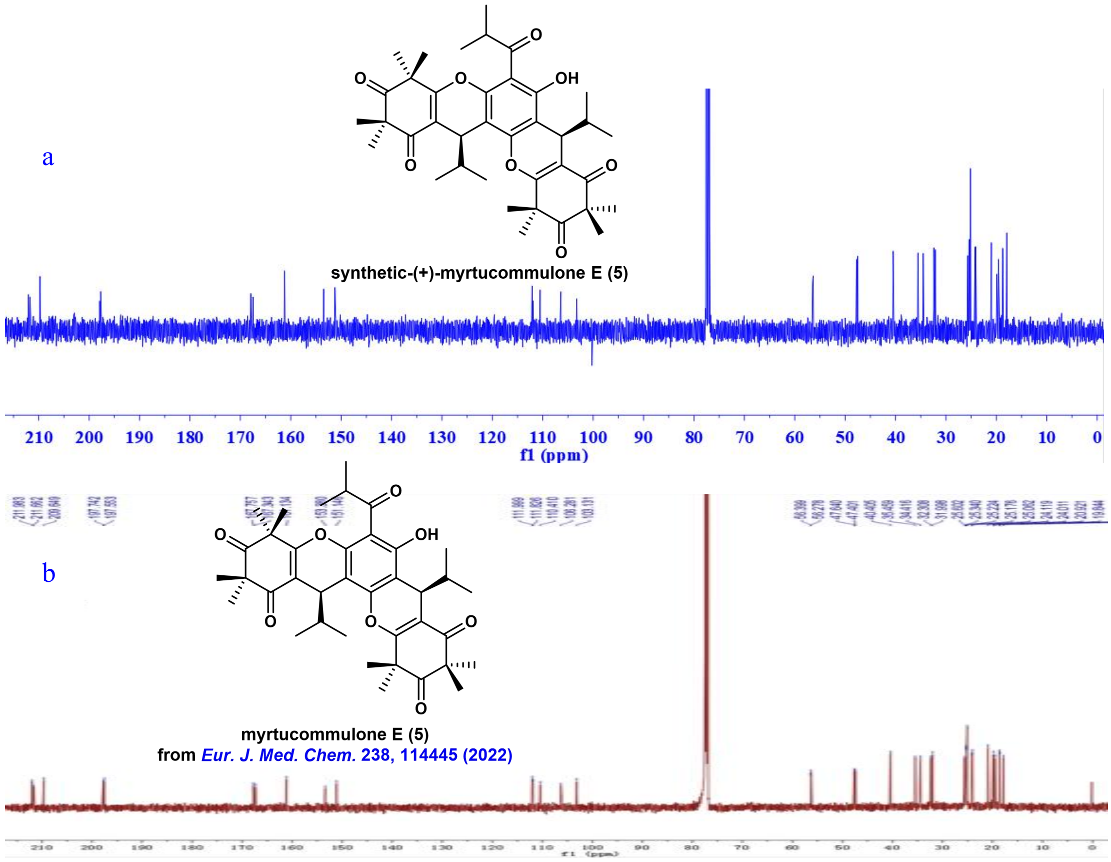

Supplementary Figure 246. Comparison of ^{13}C NMR spectra between synthetic and the reported 5. a, ^{13}C NMR spectra of synthetic 5. b, ^{13}C NMR spectra of reported 5.

Note: Supplementary Figure 246b is reproduced from Zhang, Y. et al. Discovery, enantioselective synthesis of myrtucommulone E analogues as tyrosyl-DNA phosphodiesterase 2 inhibitors and their biological activities. *Eur. J. Med. Chem.* **238**, 114445 (2022).

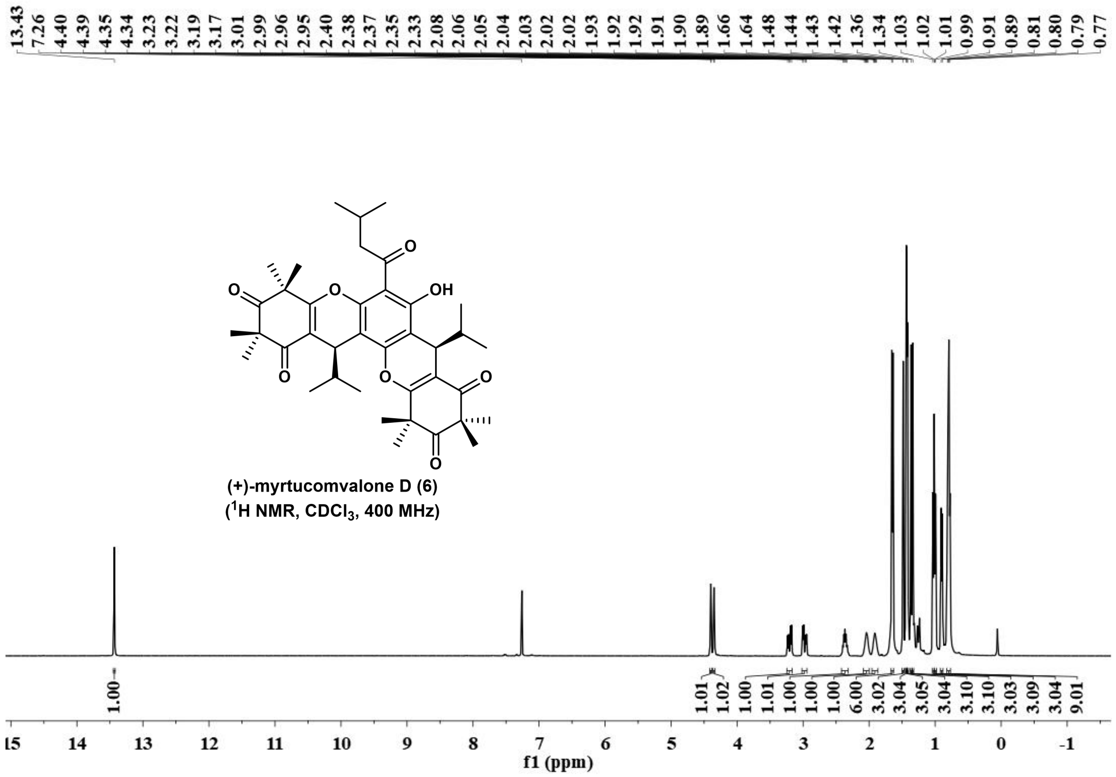

Supplementary Figure 247. ^{1}H NMR spectra of (+)-6.

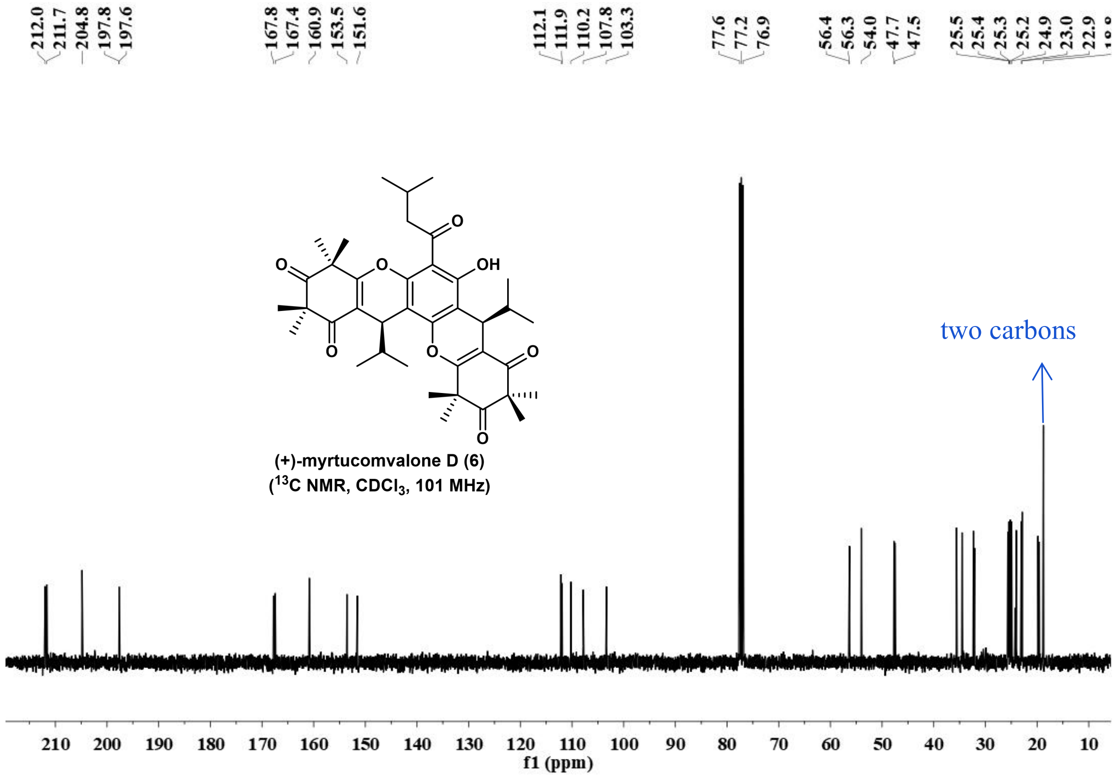

Supplementary Figure 248. ^{13}C NMR spectra of (+)-6.

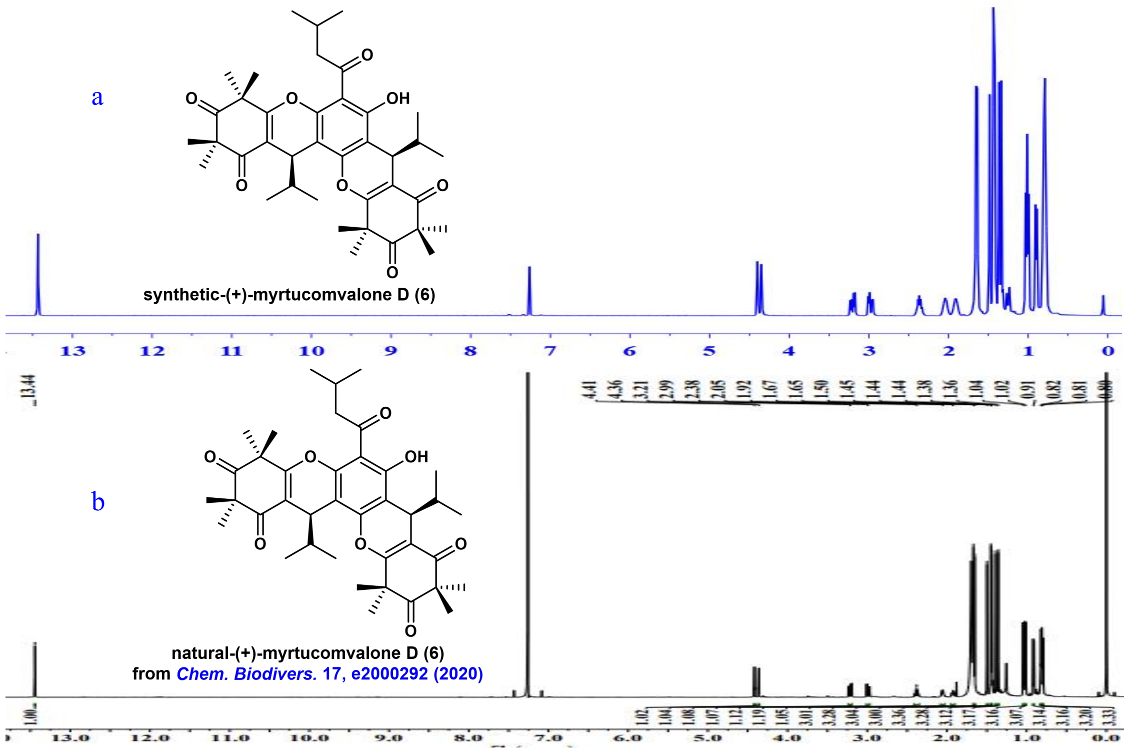

Supplementary Figure 249. Comparison of ^{1}H NMR spectra between synthetic and the isolated 6. a, ^{1}H NMR spectra of synthetic 6. b, ^{1}H NMR spectra of isolated 6.

Note: Supplementary Figure 249b is reproduced from Wu, Y. et al. Phloroglucinol derivatives from *Myrtus communis* ‘Variegata’and their antibacterial activities. *Chem. Biodivers.* **17**, e2000292 (2020).

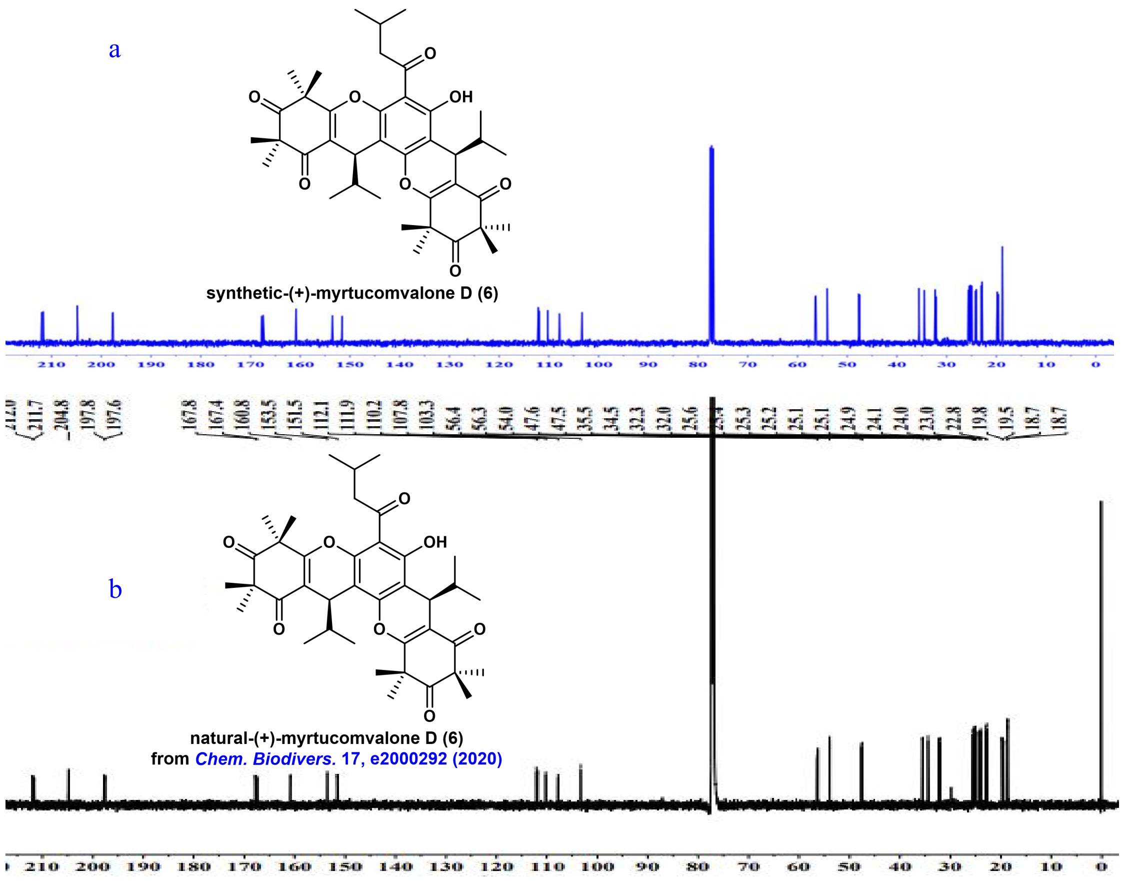

Supplementary Figure 250. Comparison of ^{13}C NMR spectra between synthetic and the isolated 6. a, ^{13}C NMR spectra of synthetic 6. b, ^{13}C NMR spectra of isolated 6.

Note: Supplementary Figure 250b is reproduced from Wu, Y. et al. Phloroglucinol derivatives from *Myrtus communis* 'Variegata'and their antibacterial activities. *Chem. Biodivers.* **17**, e2000292 (2020).

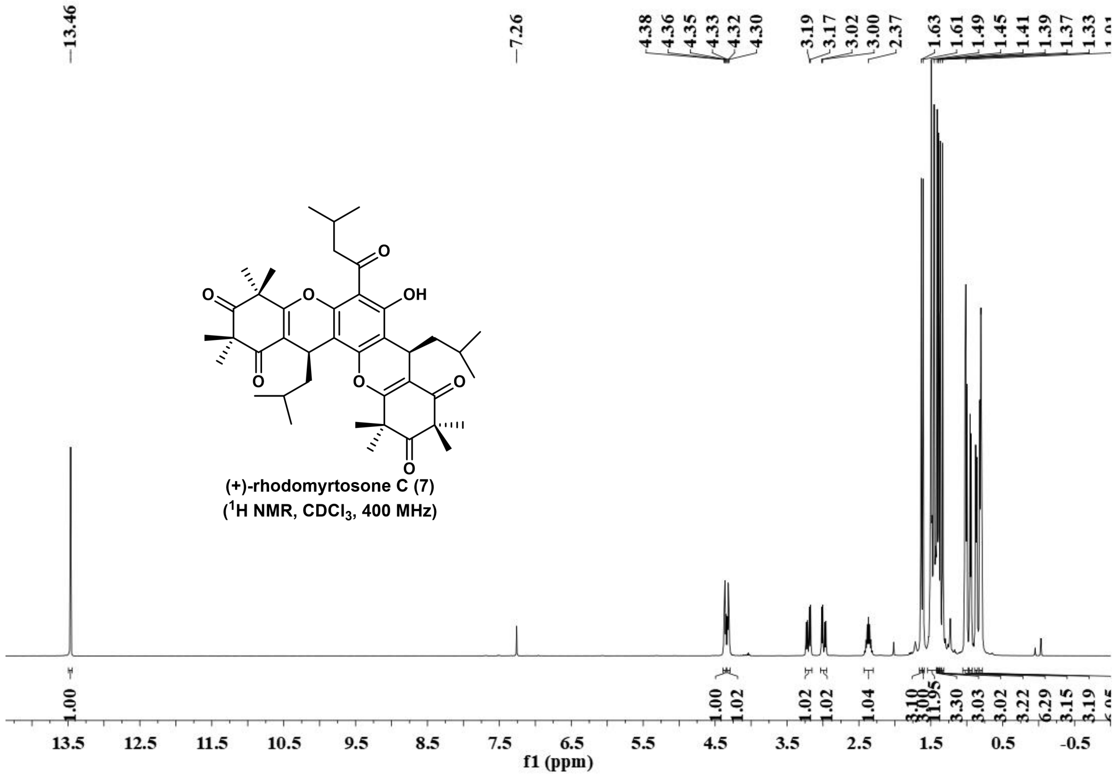

Supplementary Figure 251. ^{1}H NMR spectra of (+)-7.

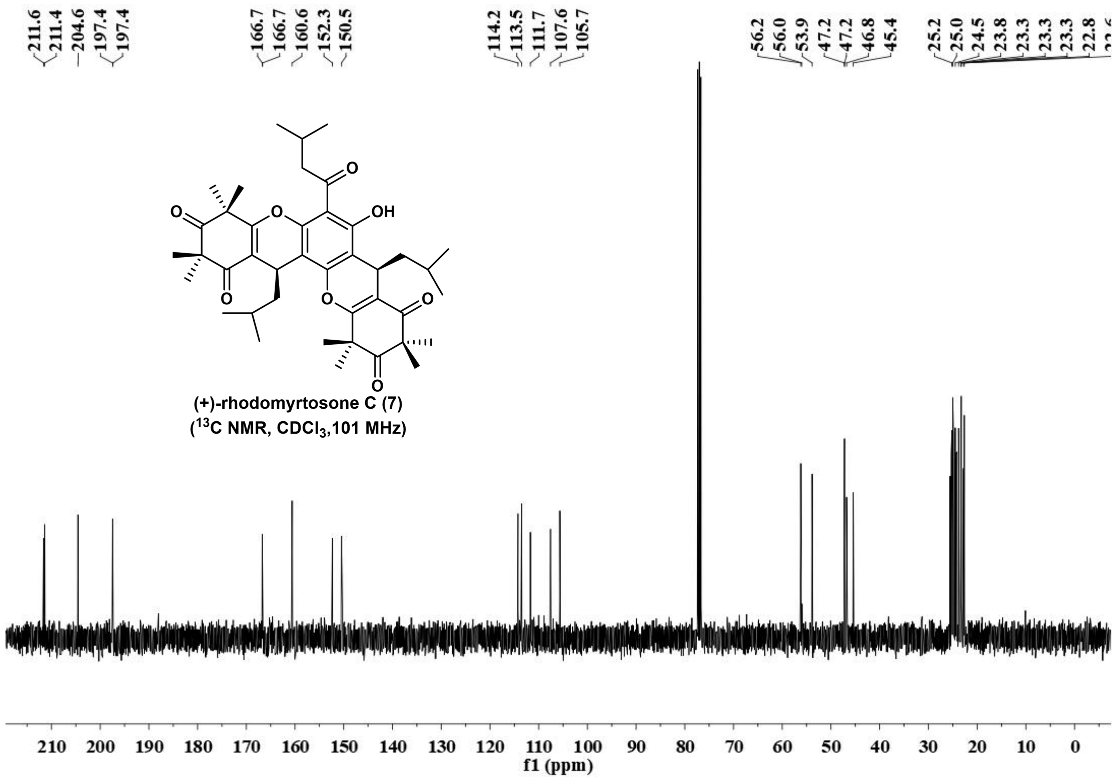

Supplementary Figure 252. ^{13}C NMR spectra of (+)-7.

Note: Since no pictures of ^{1}H, ^{13}C spectra were provided in the reported literature, which includes

Wang, J.'s[6], and Mahabusarakam, W.'s work[8], we are currently unable to present comparative spectra images of ^{1}H, ^{13}C between our synthetic **7** and natural **7**.

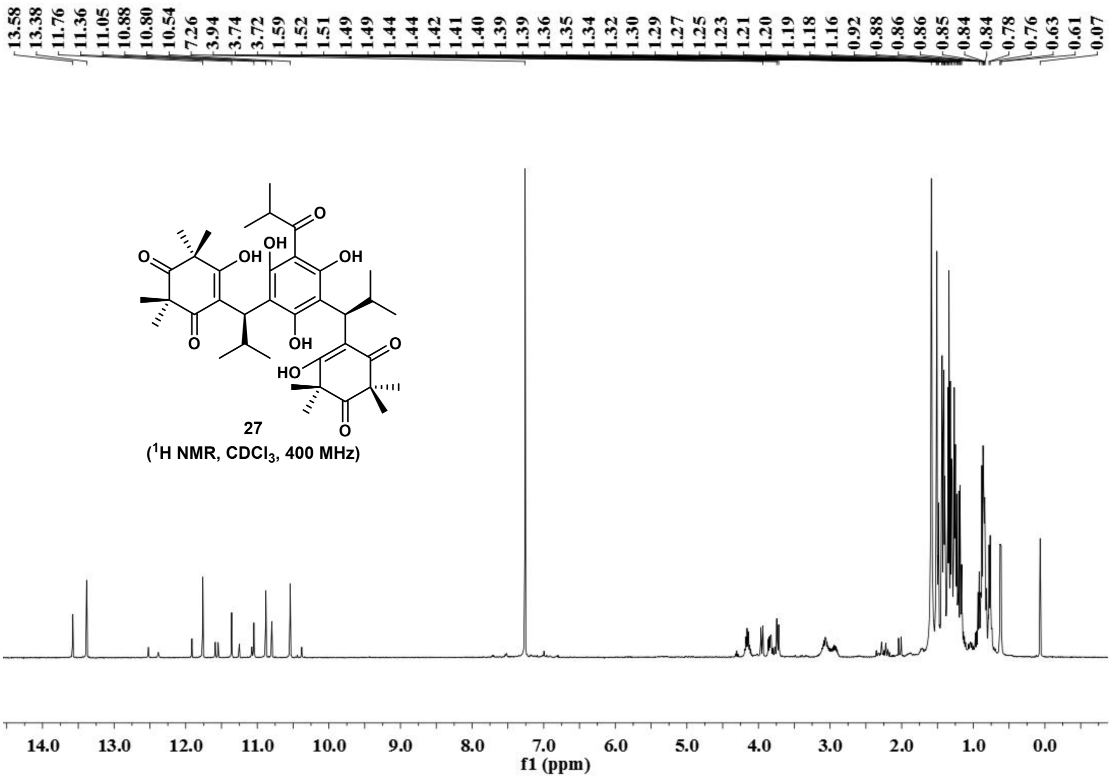

Supplementary Figure 253. ^{1}H NMR spectra of 27.

3.11 The tables and figures of antibacterial activity assay

Supplementary Table 11. MICs of 66 synthetic compounds (μg/mL).

Compounds	*S. aureus* ATCC 29213	*S. aureus* (MSSA) CC 49050	*S. aureus* (MRSA) MRSA 252	*S. aureus* (MRSA) CC 48973	*E. faecalis* ATCC 29212	*E. faecium* (VRE) CC 42266	*S. pneumoniae* (PSSP) CC F3993	*P. aeruginosa* ATCC 27853	*E. coli* ATCC 25922	*K. pneumoniae* ATCC 13883
(+)-**1**	2	2	1	1	32	8	16	>128	>128	>128
(-)-**1**	2	1	1	1	32	8	8	>128	>128	>128
**1a**	>128	>128	>128	>128	>128	>128	>128	>128	>128	>128
(+)-**2**	2	2	1	1	16	8	32	>128	>128	>128
(-)-**2**	1.5	1	1	1	8	4	16	>128	>128	>128
(+)-**3**	2	2	4	4	8	4	16	>128	>128	>128
(-)-**3**	2	4	4	4	8	8	16	>128	>128	>128
(+)-**5**	>128	>128	>128	>128	>128	>128	>128	>128	>128	>128
(-)-**5**	>128	>128	>128	>128	>128	>128	>128	>128	>128	>128
rac-**5a**	>128	>128	>128	>128	>128	>128	>128	>128	>128	>128
(+)-**6**	>128	>128	>128	>128	>128	>128	>128	>128	>128	>128
(-)-**6**	>128	>128	>128	>128	>128	>128	>128	>128	>128	>128
(+)-**7**	>128	>128	>128	>128	>128	>128	>128	>128	>128	>128
(-)-**7**	>128	>128	>128	>128	>128	>128	>128	>128	>128	>128
(+)-**8**	4	8	4	4	8	4	16	>128	>128	>128
(-)-**8**	4	16	4	4	8	4	16	>128	>128	>128
(+)-**9**	4	8	2	2	1	2	32	>128	>128	>128
(-)-**9**	2	4	2	2	2	1	4	>128	>128	>128
(+)-**11**	0.25	0.5	0.5	0.5	4	2	16	>128	>128	>128
(-)-**11**	0.25	0.5	0.5	0.5	1	1	8	>128	>128	>128
(+)-**14**	0.5	1	1	1	2	2	32	>128	>128	>128
(-)-**14**	0.5	0.5	1	1	2	4	16	>128	>128	>128
rac-**14a**	>128	>128	>128	>128	>128	>128	>128	>128	>128	>128
(+)-**17a**	0.25	2	0.5	0.5	1	2	8	>128	>128	>128
(-)-**17a**	0.5	0.5	0.5	0.5	2	2	8	>128	>128	>128
(+)-**17b**	0.5	0.5	0.5	0.5	1	1	16	>128	>128	>128
(-)-**17b**	0.5	0.5	0.5	0.5	16	2	16	>128	>128	>128
(+)-**17c**	1	4	1	1	2	2	16	>128	>128	>128
(-)-**17c**	0.5	0.5	1	1	2	2	16	>128	>128	>128
(+)-**17d**	1	1	1	1	4	2	32	>128	>128	>128
(-)-**17d**	1	1	1	1	2	4	16	>128	>128	>128
(+)-**17e**	0.5	0.5	1	1	2	2	16	>128	>128	>128

(-)-**17e**	0.5	0.5	1	1	1	2	8	>128	>128	>128
(+)-**17f**	8	4	8	8	64	4	32	>128	>128	>128
(-)-**17f**	8	8	8	8	32	4	32	>128	>128	>128
(+)-**17g**	0.5	0.5	0.5	0.5	1	2	8	>128	>128	>128
(-)-**17g**	0.5	0.5	0.5	0.5	1	1	8	>128	>128	>128
(+)-**17h**	16	16	16	16	32	32	32	>128	>128	>128
(-)-**17h**	64	64	64	64	32	64	>128	>128	>128	>128
(+)-**17i**	4	4	4	4	4	4	16	>128	>128	>128
(-)-**17i**	4	8	4	4	4	4	16	>128	>128	>128
(+)-**17j**	4	8	2	2	4	4	16	>128	>128	>128
(-)-**17j**	4	4	4	4	8	4	16	>128	>128	>128
(+)-**17k**	8	8	8	8	8	4	32	>128	>128	>128
(-)-**17k**	8	8	8	8	8	8	64	>128	>128	>128
(+)-**17l**	2	4	2	2	2	64	8	>128	>128	>128
(-)-**17l**	2	2	2	2	4	>128	8	>128	>128	>128
(+)-**17m**	16	16	8	8	8	8	32	>128	>128	>128
(-)-**17m**	8	16	8	8	8	8	16	>128	>128	>128
(+)-**17n**	>128	>128	>128	>128	>128	>128	>128	>128	>128	>128
(-)-**17n**	>128	>128	>128	>128	>128	>128	>128	>128	>128	>128
(+)-**17o**	>128	>128	>128	>128	>128	>128	>128	>128	>128	>128
(-)-**17o**	>128	>128	>128	>128	>128	>128	>128	>128	>128	>128
(+)-**18**	>128	>128	>128	>128	>128	>128	>128	>128	>128	>128
(-)-**18**	>128	>128	>128	>128	>128	>128	>128	>128	>128	>128
(+)-**18a**	>128	>128	>128	>128	>128	>128	>128	>128	>128	>128
(-)-**18a**	>128	>128	>128	>128	>128	>128	>128	>128	>128	>128
(+)-**18b**	>128	>128	>128	>128	>128	>128	>128	>128	>128	>128
(-)-**18b**	>128	>128	>128	>128	>128	>128	>128	>128	>128	>128
**21**	2	1	1	1	2	2	2	>128	>128	>128
**21a**	>128	>128	>128	>128	>128	>128	>128	>128	>128	>128
**22**	0.5	0.5	0.5	0.5	1	1	1	>128	>128	>128
**22a**	>128	>128	>128	>128	>128	>128	>128	>128	>128	>128
rac-**24a**	>128	>128	>128	>128	>128	>128	>128	>128	>128	>128
rac-**24b**	>128	>128	>128	>128	>128	>128	>128	>128	>128	>128
**27**	2	2	2	2	8	4	8	>128	>128	>128
Polymyxin B	-	-	-	-	-	-	-	0.5	0.5	0.5
Vancomycin	1	1	1	1	4	32	4	-	-	-
Daptomycin	1	4	4	8	8	32	2	-	-	-
Oxacillin	0.5	0.5	>128	>128	-	-	-	-	-	-

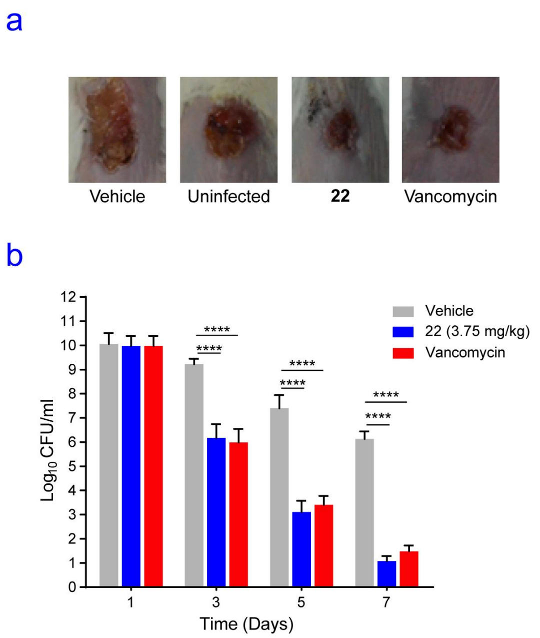

Supplementary Figure 254. *In vivo* activity against MRSA 252 of **22**. **a**, The open wound area, animals were grouped randomly into 4 groups (n = 3 per group). **b**, Local bacterial load of mice infected with 50 µL of MRSA 252 at a concentration of 2 × 10^9 CFU mL^{-1} after **22** and vancomycin treatment, animals were grouped randomly into 3 groups (n = 20 per group, n = 5 per time point). The mean is shown, and error bars represent the s.d. *P* values were determined using a nonparametric one-way ANOVA. $^{****}P < 0.0001$. Source data are provided as a Source Data file.

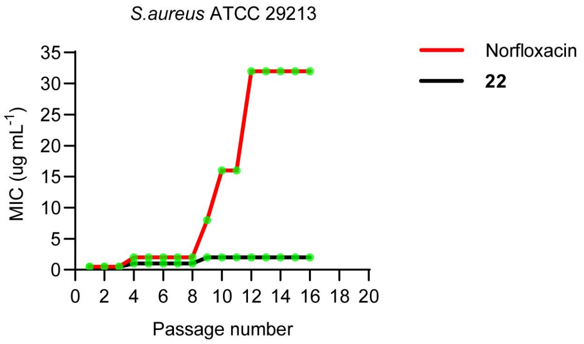

Supplementary Figure 255. Bacterial resistance studies of compound **22**, norfloxacin against *S. aureus* ATCC 29213.Source data are provided as a Source Data file.

Supplementary Table 12. Antibacterial activity of **22** against *Staphylococcus aureus* ATCC29213, spontaneous resistant mutant ($SA_{22\text{-}SR}$) and $SA_{WalK(R86C)}$ (μg/mL).

Bacterial species	Number of strains	Range of MICs for multiple strains	Median MIC
Staphylococcus aureus, ATCC29213 (SA_{29213})	1	0.5	—
Staphylococcus aureus, spontaneous resistant mutant ($SA_{22\text{-}SR}$)	4	4	4
$SA_{WalK(R86C)}$	1	2	—

Supplementary Table 13. MICs (μg/mL) of antibiotics in **22-**susceptible and **22-**resistant *Staphylococcus aureus.*

Antibiotics	SA_{29213}	$SA_{22\text{-}SR}$
Vancomycin	1	1
Ofloxacin	0.2	0.2
Linezolid	2.5	2.5
Kanamycin	10	5
Meropenem	0.3125	0.156
Tigecycline	0.3125	0.3125

Supplementary Table 14. Whole-genome sequencing identified in spontaneous resistant mutant ($\text{SA}_{\mathbf{22}\text{-SR}}$).

Strains[£]	Locus_Tag[*]	Gene	Codon position	Amino acid position	Product
$\text{SA}_{\mathbf{22}\text{-SR-1}}$ to $\text{SA}_{\mathbf{22}\text{-SR-4}}$	NWMN_RS13105	*Hypothetical*	1102 A > C	368 Asn > His	YhgE/Pip domain-containing protein
$\text{SA}_{\mathbf{22}\text{-SR-1}}$ to $\text{SA}_{\mathbf{22}\text{-SR-4}}$	NWMN_RS13110	*Hypothetical*	361 G > A	121 Glu > Lys	TetR/AcrR family transcriptional regulator
$\text{SA}_{\mathbf{22}\text{-SR-1}}$ to $\text{SA}_{\mathbf{22}\text{-SR-4}}$	NWMN_RS00030	*gyrA*	82 A > G	28 Ser > Gly	DNA gyrase subunit A
$\text{SA}_{\mathbf{22}\text{-SR-1}}$ to $\text{SA}_{\mathbf{22}\text{-SR-4}}$	NWMN_RS00105	*walK*	256 C > T	86 Arg > Cys	Sensor protein kinase
$\text{SA}_{\mathbf{22}\text{-SR-1}}$ to $\text{SA}_{\mathbf{22}\text{-SR-4}}$	NWMN_RS08735	*valS*	2533 G > T	845 Ala > Ser	Valine-tRNA ligase
$\text{SA}_{\mathbf{22}\text{-SR-1}}$ to $\text{SA}_{\mathbf{22}\text{-SR-4}}$	NWMN_RS08100	*Hypothetical*	72 dup A	25 Leu > frameshift	Hypothetical protein
$\text{SA}_{\mathbf{22}\text{-SR-1}}$ to $\text{SA}_{\mathbf{22}\text{-SR-4}}$	NWMN_RS07135	*plsY*	110 G > A	37 Gly > Asp	G3P acyltransferase
$\text{SA}_{22\text{-SR-1}}$ to $\text{SA}_{\mathbf{22}\text{-SR-4}}$	NWMN_RS06795	*glpK*	1282 del T	428 Ser > frameshift	Glycerol kinase
$\text{SA}_{\mathbf{22}\text{-SR-1}}$ to $\text{SA}_{\mathbf{22}\text{-SR-4}}$	NWMN_RS02345	*Hypothetical*	19 G > T	7 Val > Phe	hypothetical protein
$\text{SA}_{\mathbf{22}\text{-SR-2}}$ to $\text{SA}_{\mathbf{22}\text{-SR-4}}$	NWMN_RS06410	*fakA*	1434-1439 del CAGCCA	478 Ser-480 Gln > Arg	Fatty acid kinase catalytic subunit
$\text{SA}_{\mathbf{22}\text{-SR-2}}$ to $\text{SA}_{\mathbf{22}\text{-SR-4}}$	NWMN_RS02905	*nusG*	229-242 dup ACAGATGAATCATG	81 Trp > frameshift	Transcription termination/antitermination protein
$\text{SA}_{\mathbf{22}\text{-SR-3}}$ to $\text{SA}_{\mathbf{22}\text{-SR-4}}$	NWMN_RS01075	*tarS*	335-338 del ATCA	112 His > frameshift	poly(ribitol-phosphate) beta-N-acetylglucosaminyltransferase
$\text{SA}_{\mathbf{22}\text{-SR-4}}$	NWMN_RS13105	*Hypothetical*	473 C > T	158 Thr > Ile	Hypothetical protein

£ Strains carrying the mutation in the corresponding line.

* Newman strain (NC_009641) is used as the reference for the mutation annotation.

dup and del represent duplication and deletion, respectively.

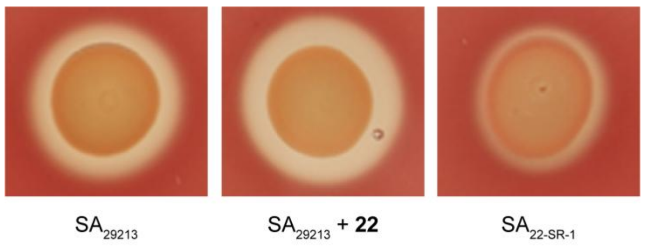

Supplementary Figure 256. The haemolysis activity of *Staphylococcus aureus* ATCC29213 (SA_{29213}), spontaneously resistant strain ($\text{SA}_{\mathbf{22}\text{-SR-1}}$) and SA_{29213} after **22** (0.1 μg/mL) treatment on sheep blood agar. Source data are provided as a Source Data file.

Supplementary Table 15. Primers used in this study.

Sequences	Description
TACTTCCAATCCAATGACCAGCACAAGACACTGGAA	*walK* and flanks amplification-forward
TTATCCACTTCCAATGCCATTTTACGCGTACGTGCC	*walK* and flanks amplification-reverse
AGGGCTCCGTAAATGCACAA	Mutation verification (*walK*)-forward
CAGACACGGTCCTTACCACC	Mutation verification (*walK*)-reverse
TCTTGCCAGCTTTCCCCTTC	*pyJ335*-forward
GGTGTAGAGCAGCCTACATTGTATTG	*pyJ335*-reverse
AAGCAGCTCTAATGCGCTGT	Recombination verification-forward
TGACGGTTGGCATACTCACT	Recombination verification-reverse
ACGTGGATAACCTACCTATAAGACTGGGAT	*16SrRNA* transcription-forward
TACCTTACCAACTAGCTAATGCAGCG	*16SrRNA* transcription-reverse
AACAGCACCAACGGATTAC	*atlA* transcription-forward
CATAGTCAGCATAGTTATTCATTG	*atlA* transcription-reverse
CAGCAACAGCAGGAGATAAC	*lytM* transcription-forward
ATAATTGACCTTTCCATTACCATC	*lytM* transcription-reverse
AGAGATTCTTGGAACCCGGT	*hla* transcription-forward
ACTGTAGCGAAGTCTGGTGA	*hla* transcription-reverse
GCAGTAGGTTTAGGAATCGTAGCAGGAAAT	*sceD* transcription-forward
CTGATTCAAAGTGATAAGTAAACCCTTCAT	*sceD* transcription-reverse
CCGTACTGGTGGTTTAGGTGCAAGCTACAG	*ssaA* transcription-forward
GCATTGCCCCAAGTTGAACCGATTTTACCA	*ssaA* transcription-reverse
CTTCTACACAACATACAGTACAAT	*SA0620* transcription-forward
TCAACTGAAACACCATATCTGC	*SA0620* transcription-reverse
GATTCACAGTAAATCATACACCTTC	*SA2097* transcription-forward
TGATGACATATGTACTAGAATTAAG	*SA2097* transcription-reverse
CATGATGCACAAGCCGCAGA	*SA2353* transcription-forward
GATGCATTGTTATAACTATA	*SA2353* transcription-reverse
AATTATATTCATACAATCCTGGTG	*SA0710* transcription-forward
GGTGCTTGCTTAACTACTAC	*SA0710* transcription-reverse
GGTACTACATGGTCATGGAGCTATGAAGC	*isaA* transcription-forward
CTCACTGAACTTGAAGTAGTTGAAGTGCTG	*isaA* transcription-reverse
TGCTTCTGCCGCTTCAAAAC	*ebpS* transcription-forward
TACTTTGGCCATGCCACCTT	*ebpS* transcription-reverse
TTGCCAACGCCTTTTTCTCC	*sdrD* transcription-forward
TAACCAAAGTGGCGGAGCTG	*sdrD* transcription-reverse

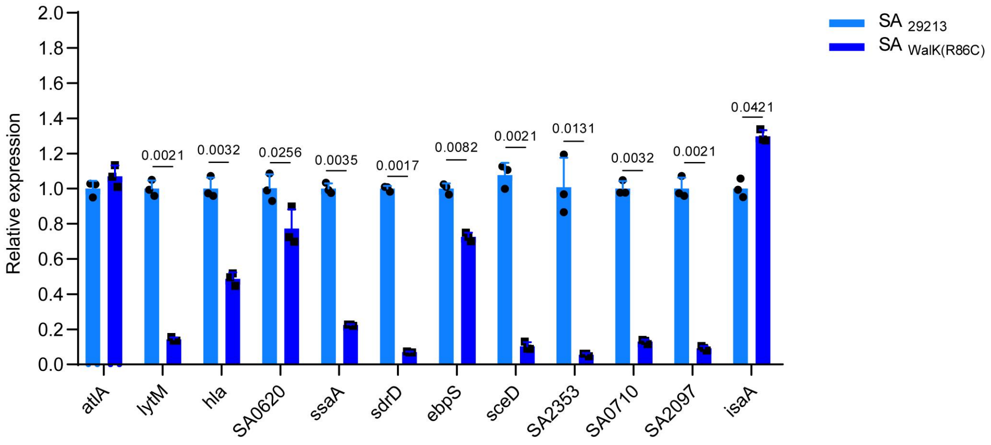

Supplementary Figure 257. Transcription levels of genes regulated by WalKR in *Staphylococcus aureus* ATCC 29213 and its WalK(R86C) mutant $SA_{WalK(R86C)}$. The mean is shown, and error bars represent the s.d. *P* values were determined using an unpaired t-test.

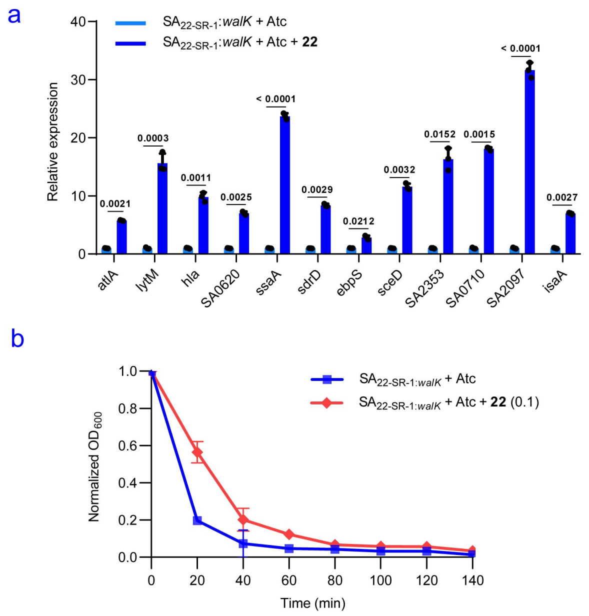

Supplementary Figure 258. a, Transcription levels of genes regulated by WalKR in the presence or absence of **22** (0.025 μg/mL) in *walK* overexpression strain based on **22** spontaneously resistant strain ($SA_{22\text{-}SR\text{-}1}$). The mean is shown, and error bars represent the s.d. *P* values were determined using an paired t-test. **b**, Lysostaphin-induced lysis process in the presence or absence of **22** (0.1 μg/mL) in *walK* overexpression strain based on **22** spontaneously resistant strain ($SA_{22\text{-}SR\text{-}1}$). Anhydrotetracycline (Atc) (100 ng/mL) was used to induce transcription. Source data are provided as a Source Data file.

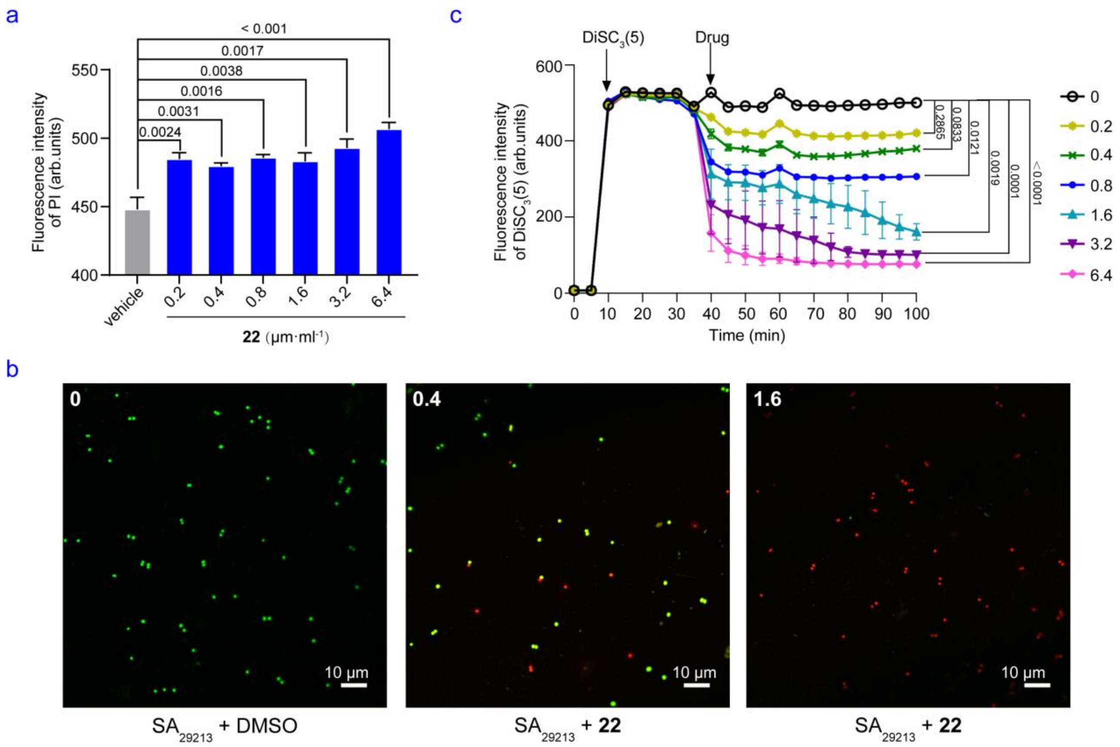

Supplementary Figure 259. a, Permeability of the membrane probed with PI. **b**, Confocal images of SA_{29213} cells treated with **22** (0, 0.4 and 1.6 µg/mL) overnight. Viable bacterial cells were stained green by SYTO9, whereas dead cells were stained red by PI. **c**, Potential of the membrane probed with 3,3-dipropylthiadicarbocyanine iodide $DiSC_3(5)$. Three biologically independent experiments were performed in **a-c**. The mean ± s.d. is shown. *P* values were determined using an unpaired, two-tailed Student's *t*-test. Source data are provided as a Source Data file.

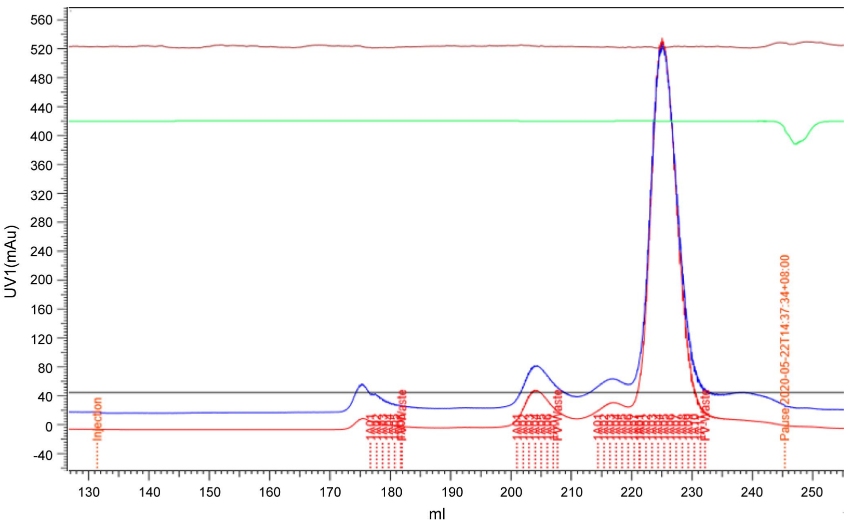

Supplementary Figure 260. Gel filtration results of erWalK$_{R86C}$ protein. Source data are provided as a Source Data file.

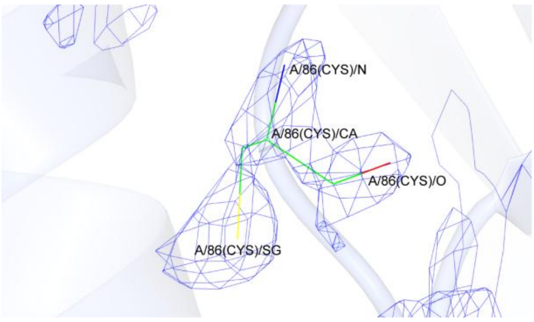

Supplementary Figure 261. Fo-Fc electron density showing the Cys86 residue. Source data are provided as a Source Data file.

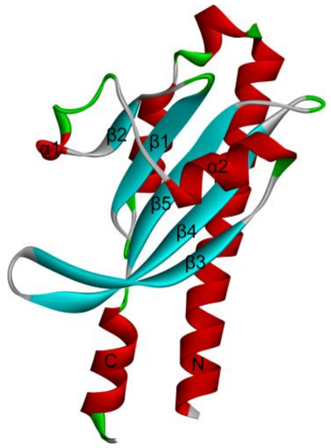

Supplementary Figure 262. Overall structure of erWalK$_{R86C}$ with each part appropriately labeled. N and C label the termini. Source data are provided as a Source Data file.

Supplementary Table 16. Summarizing structural and refinement statistics.

Data collection	erWalKR$_{86C}$
Space group	P32
Cell dimensions	
a, b, c (Å)	66.63, 66.63, 80.08
a, b, g (Å)	90.00, 90.00, 120.00
Wavelength (Å)	0.979
Resolution (Å)	21.05-1.95 (1.98-1.95)
R_{merge}	0.096 (0.573)
R_{pim}	0.033 (0.184)
$I/\sigma I$	23.9 (2.8)
Completeness (%)	100 (100)
Multiplicity	10.2 (9.0)
Refinement	
Resolution (Å)	21.05-1.95 (1.98-1.95)
No. reflections	28906 (1412)
R_{work}/R_{free}	0.197/0.268
No. atoms	
Protein	2368
Ligand/ion	/
Water	159
No. residues	147
B-factors (Å^2)	
Protein	35.5
Ligand/ion	/
Water	41.5
R.m.s deviations	
Bond lengths (Å)	0.01
Bond angles (°)	1.682
Ramachandran	
Favoured (%)	97.9
Allowed (%)	2.1
Outlier (%)	0
PDB ID	7DUD

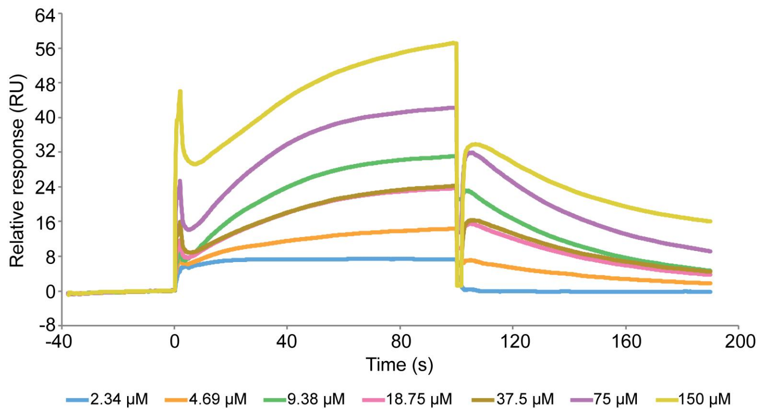

Supplementary Figure 263. SPR analysis demonstrated the marked decrease in the affinity of erWalK$_{R86C}$ (K_D = 7.38 × 10^{-5} M) with **22**. Source data are provided as a Source Data file.

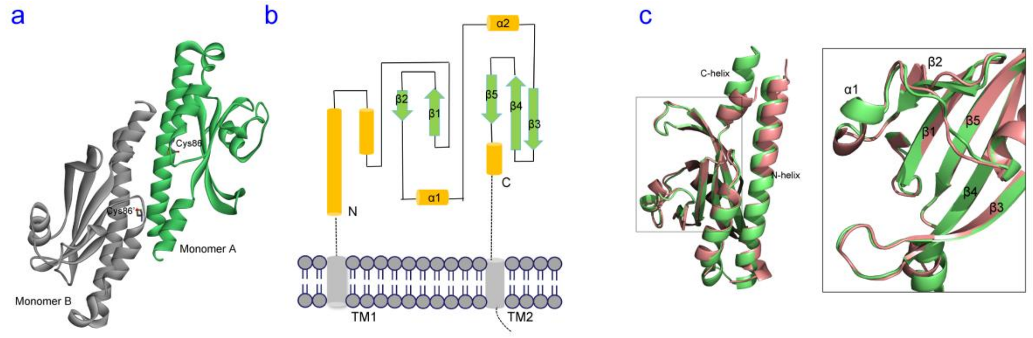

Supplementary Figure 264. a, Overall structure of the erWalK$_{R86C}$ dimer. **b**, The schematic topology diagram shows the secondary structure of erWalK$_{R86C}$. The orange cylinders and green arrows mark the α-helices and β-strands, respectively; the grey lines mark the loops and turns. This figure was generated by PDB sum (http://www.ebi.ac.uk/pdbsum)[14]. **c**, Structure alignment of erWalK (wheat) and erWalK$_{R86C}$ (green) to show the structure rearrangements of the erWalK$_{R86C}$ long α-helice close to the β-strand region and of the α1 and β2. Source data are provided as a Source Data file.

Note: We docked **22** into the wild-type structure of WalK. Consistent with the observation that the R86C mutant was inert to **22**, the ligand was found to interact with WalK in the α1 and β2 regions where the mutant structure differs most from the wild-type structure. In detail, **22** forms 5 hydrogen bonds within the β-sheet pocket formed by β1-5 and α1 (Supplementary Figure 264). As calculated using AutoDock Tools, **22** has a high affinity of 2.01 × 10^{-5} M for erWalK, which is consistent with the results obtained with OpenSPR. The three residues that form hydrogen bonds with **22** are Arg93, Asp139, and Asn109. In addition, the hydrocarbon potion of Lys106, and

residues Leu112, Ile136, and Tyr140 interacts with **22** by hydrophobic interactions (Supplementary Table 17). In contrast, **22** docked poorly to the same pocket in erWalK$_{R86C}$ with a predicted affinity of 6.56 × 10^{-5} M. The local structural rearrangements in R86C diminished most of the interactions, leaving only Lys138 for hydrogen bonding, and Leu112 and Try165 for hydrophobic interactions (Supplementary Table 18).

Supplementary Table 17. Hydrogen bonds and hydrophobic bonds formed between **22** and erWalK.

Bond	Distance (Å)	Category	Types
A:ARG93:NH2-X:MCR:O1	3.19139	Hydrogen Bond	Conventional Hydrogen Bond
X: MCR:H10-A:ASP139:O	1.96384	Hydrogen Bond	Conventional Hydrogen Bond
X:MCR:H8-A:ASN109:OD1	2.5165	Hydrogen Bond	Conventional Hydrogen Bond
X:MCR:H30-A:ASP139:O	2.39415	Hydrogen Bond	Conventional Hydrogen Bond
A:LYS138:NZ-X:MCR	4.01839	Hydrogen Bond; Electrostatic	Pi-Donor Hydrogen Bond
X:MCR:C30-A:ILE136	4.04101	Hydrophobic	Alkyl
X:MCR:C21-A:LYS106	5.42549	Hydrophobic	Alkyl
X:MCR:C3-A:LEU112	4.57127	Hydrophobic	Alkyl
X:MCR:C1-A:LEU112	4.39344	Hydrophobic	Alkyl
A:TYR140-X:MCR:C21	4.95014	Hydrophobic	Pi-Alkyl
X:MCR-A:LYS138	5.48067	Hydrophobic	Pi-Alkyl

Supplementary Table 18. Hydrogen bonds and hydrophobic bonds formed between **22** and erWalK$_{R86C}$.

Bond	Distance (Å)	Category	Types
X:MCR:H8-X:MCR:O5	2.33035	Hydrogen Bond	Conventional Hydrogen Bond
B:LYS138:NZ-X:MCR	3.83483	Hydrogen Bond; Electrostatic	Pi-Donor Hydrogen Bond
X:MCR:C16-X:MCR	3.74395	Hydrophobic	Pi-Sigma
X:MCR:C34-B:LYS138	3.96272	Hydrophobic	Alkyl
X:MCR:C13-B:LEU112	4.67576	Hydrophobic	Alkyl
X:MCR:C38-B:LEU112	4.49271	Hydrophobic	Alkyl
B:TYR165-X:MCR:C21	4.83934	Hydrophobic	Pi-Alkyl

4. Supplementary References

1. Cheng, M. J. et al. Catalytic asymmetric total syntheses of myrtucommuacetalone, myrtucommuacetalone B, and callistrilones A, C, D and E. *Chem. Sci.* **9**, 1488-1495 (2018).
2. Hans, M. et al. Stereoisomeric composition of natural myrtucommulone A. *J. Nat. Prod.* **78**, 2381-2389 (2015).
3. Shaheen, F. et al. New α-glucosidase inhibitors and antibacterial compounds from *Myrtus communis* L. *Eur. J. Org. Chem.* **2006**, 2371-2377 (2006).
4. Ni, W. et al. Acylphloroglucinol oligomer, its preparation method, and application in treatment of Alzheimer's disease and complications thereof. CN Patent 113461702 (2022).
5. Rattanaburi, S., Mahabusarakam, W., Phongpaichit, S. & Carroll, A. R. Acylphloroglucinols from *Callistemon lanceolatus* DC. *Tetrahedron* **69**, 6070-6075 (2013).
6. Liu, J. *Phloroglucinol derivatives from the twigs and leaves of Rhodomyrtus tomentosa (Chinese version*, *Master's thesis)*, Jinan University (2016).
7. Wu, Y. et al. Phloroglucinol derivatives from *Myrtus communis* 'Variegata'and their antibacterial activities. *Chem. Biodiversity* **17**, e2000292 (2020).
8. Hiranrat, A. & Mahabusarakam, W. New acylphloroglucinols from the leaves of *Rhodomyrtus tomentosa*. *Tetrahedron* **64**, 11193-11197 (2008).
9. Frisch, M. et al. Fox. Gaussian 16 Rev. A.03, Wallingford, CT (2016).
10. Zhao, Y. & Truhlar, D. The M06 suite of density functionals for main group thermochemistry, thermochemical kinetics, noncovalent interactions, excited states, and transition elements: two new functionals and systematic testing of four M06-class functionals and 12 other functionals. *Theor. Chem. Acc.* **120**, 215-241 (2008).
11. Ditchfifield, R., Hehre, W. & Pople, J. Self-consistent molecular-orbital methods. IX. An extended Gaussian-type basis for molecular-orbital studies of organic molecules. *J. Chem. Phys.* **54**, 724-728 (1971).
12. Fuentealba, P., Preuss, H., Stoll, H. & Szentpály, L. A proper account of core-polarization with pseudopotentials: single valence-electron alkali compounds. *Chem. Phys. Lett.* **89**, 418-422 (1982).
13. Wiberg, K. B. Basis set effects on calculated geometries:6-311++G** vs. aug-cc-pVDZ. *J. Comput. Chem.* **25**, 1342-1346 (2004).

14. de Beer, T. A., Berka, K., Thornton, J. M. & Laskowski, R. A. PDBsum additions. *Nucleic Acids Res.* **42**, D292-296 (2014).
