# Supplementary material for: Asymmetric total synthesis of polycyclic xanthenes and discovery of a WalK activator active against MRSA
Source: Nat Commun. 2024 Jul 13;15:5879. doi: 10.1038/s41467-024-49629-8 (PMC11245619; doi:10.1038/s41467-024-49629-8)
Supplement: Supplementary file 3 — Description of Additional Supplementary Files [file 41467_2024_49629_MOESM3_ESM.pdf]

Description of Additional Supplementary Files

Supplementary Data 1: Coordinates of all located intermediates and transition structures
